# Clustered sparsity and Poisson-gap sampling

**DOI:** 10.1007/s10858-021-00385-7

**Published:** 2021-11-05

**Authors:** Paweł Kasprzak, Mateusz Urbańczyk, Krzysztof Kazimierczuk

**Affiliations:** 1 Centre of New Technologies, University of Warsaw, Banacha 2C, 02-097 Warsaw, Poland; 2 Faculty of Physics, University of Warsaw, Pasteura 5, 02-093 Warsaw, Poland; 3 Institute of Physical Chemistry, Polish Academy of Sciences, Kasprzaka 44/52, 01-224 Warsaw, Poland

**Keywords:** Poisson-gap sampling, Compressed sensing, Non-uniform sampling, Blue-noise sampling, Iterative soft thresholding, Clustered sparsity

## Abstract

Non-uniform sampling (NUS) is a popular way of reducing the amount of time taken by multidimensional NMR experiments. Among the various non-uniform sampling schemes that exist, the Poisson-gap (PG) schedules are particularly popular, especially when combined with compressed-sensing (CS) reconstruction of missing data points. However, the use of PG is based mainly on practical experience and has not, as yet, been explained in terms of CS theory. Moreover, an apparent contradiction exists between the reported effectiveness of PG and CS theory, which states that a “flat” pseudo-random generator is the best way to generate sampling schedules in order to reconstruct sparse spectra. In this paper we explain how, and in what situations, PG reveals its superior features in NMR spectroscopy. We support our theoretical considerations with simulations and analyses of experimental data from the Biological Magnetic Resonance Bank (BMRB). Our analyses reveal a previously unnoticed feature of many NMR spectra that explains the success of ”blue-noise” schedules, such as PG. We call this feature “clustered sparsity”. This refers to the fact that the peaks in NMR spectra are not just sparse but often form clusters in the indirect dimension, and PG is particularly suited to deal with such situations. Additionally, we discuss why denser sampling in the initial and final parts of the clustered signal may be useful.

**Supplementary Information:**

The online version contains supplementary material available at 10.1007/s10858-021-00385-7.

## Introduction

The main limiting factor in multidimensional NMR spectroscopy is the need for extensive sampling of indirect time dimensions. The distance between sampling points is imposed by the Nyquist–Shannon sampling theorem (Nyquist 1928), and often thousands of sampling points are needed in order to achieve evolution times that provide the desired spectral resolution (Szántay 2008). Collecting this data may take many days for spectra with three dimensions or more, and even with 2D spectra tens of hours are sometimes needed to reach sufficient peak dispersion (Misiak et al. 2013).

Among the numerous strategies that have been used to alleviate the problem of lengthy experiments, non-uniform sampling (NUS) is the most common. In NUS we skip a large proportion of the data points during the signal’s acquisition and reconstruct them afterwards using various mathematical approaches. These reconstruction techniques include maximum entropy (Hoch 1985), multidimensional decomposition (Orekhov and Jaravine 2011), direct FT of zero-augmented data (Kazimierczuk et al. 2012), variants of the CLEAN algorithm (Barna et al. 1988; Coggins and Zhou 2008; Stanek et al. 2010; Ying et al. 2017), and methods based on minimizing the spectrum’s $\ell_p$-norm (Drori 2007; Kazimierczuk and Orekhov 2011; Holland et al. 2011; Hyberts et al. 2012a; Sun et al. 2015).

The strategies based on minimizing the spectrum’s $\ell_p$-norm listed above are known as compressed-sensing (or compressive sampling, CS) methods. They are based on a robust mathematical theory that states that if: a) spectrum ${\mathbf{x}}$ of size *N* is *K*-sparse (has *K* significant elements, at least approximately); b) measurement matrix ${\mathbf{A}}$ is sufficiently *incoherent*; and c) the number of sampling points is of the order $\sim K log(N)$ (usually much lower than the size of the full grid *N*), then: d) strictly sparse ${\mathbf{x}}$ can be recovered exactly (Candes et al. 2006); and e) if ${\mathbf{x}}$ is not *K*-sparse, its *K* highest points can still be recovered (Candès et al. 2006a, b). Spectrum is recovered by solving the optimization problem:

1 $$\begin{aligned} {\mathbf{x}}={{\,\mathrm{arg\,min}\,}}_{{\mathbf{z}}} \left( \Vert {\mathbf{A}}{\mathbf{z}}-{\mathbf{y}}\Vert_{\ell_{2}}+\lambda \cdot \Vert {\mathbf{z}}\Vert_{\ell_{p}}\right) \end{aligned}$$

where $\ell_p$-norm is defined as $\Vert {\mathbf{z}}\Vert_{p}=\root p \of {|z_{1}|^{p}+|z_{2}|^{p}+\ldots +|z_{n}|^{p}}$ and $\lambda$ is the constant balancing between the sparsity of the reconstructed ${\mathbf{x}}$ and its accordance with the measured data ${\mathbf{y}}.$ The measurement matrix **A** is the inverse Fourier transform (FT) matrix with rows removed according to the NUS schedule. The most popular CS reconstruction algorithm, namely ”iterative soft thresholding” (IST) (Drori 2007; Stern et al. 2007; Kazimierczuk and Orekhov 2011; Hyberts et al. 2012a; Sun et al. 2015), uses $p=1,$ but some others, such as “iteratively reweighted least squares” (IRLS) (Kazimierczuk and Orekhov 2011, 2012), allow the use of other norms as well.

Let us focus on condition (b), that is to say, (in)coherence of the FT matrix. The coherence of a matrix ${\mathbf{A}}$ can be defined as a maximum among scalar products of all pairs of its columns (see also Definition 5.1 in Foucart and Rauhut 2010). It is known that if ${\mathbf{A}}$ is a FT matrix, then the lowest coherence (highest incoherence) is achieved by purely random sampling, that is to say, it can be generated using a pseudo-random generator with time-independent probability (Candès 2006); below, we use the term “unweighted random” to describe this. The opposite of this statement is also true, that is to say, every ”regularity” in the sampling schedule results in increased coherence (Kazimierczuk et al. 2007; Hoch et al. 2008).

In multidimensional NMR, by contrast, we rarely use this unweighted random approach. The NMR signal usually decays over time (except for dimensions sampled in a constant-time mode), so the signal-to-noise ratio (SNR) also decays. Thus, it is beneficial to sample the beginning of the signal more extensively, in other words to match the sampling density to the signal’s envelope by using a decaying sampling density (Barna et al. 1987; Kumar et al. 1991; Rovnyak et al. 2011). As has been demonstrated elsewhere (Kazimierczuk et al. 2014), relaxation-matched sampling improves SNR, but it worsens the aforementioned CS reconstruction condition b), that is, the incoherence of ${\mathbf{A}}$. Besides relaxation matching, the sampling density can be matched with J-modulation, for example, resulting from $\hbox {C}_\alpha$-$\hbox {C}_\beta$ couplings in $\hbox {C}_\alpha$ dimensions (Jaravine et al. 2006; Kazimierczuk et al. 2020).

Apart from matching the sampling density to the signal’s envelope, several solutions have been proposed to achieve less non-uniform coverage of the evolution time space. These include jittered sampling (Kazimierczuk et al. 2007; Mobli 2015), quantile sampling (Craft et al. 2018), Poisson-disk sampling (Kazimierczuk et al. 2008), and Poisson-gap (PG) sampling (Hyberts et al. 2010). Together, these solutions are often referred to as “blue-noise sampling”, especially in non-NMR literature (Tang et al. 2009; Correa et al. 2016; Lanaro et al. 2020). As shown in Fig. 2, the term “blue noise” refers to the *point spread function*, that is, the fact that noise-like NUS artifacts are stronger at a greater distance from the peak (see also the discussion in Mobli and Miljenović 2019). It has been reported (Hyberts et al. 2010, 2014), based on empirical studies, that blue-noise sampling in NMR provides better reconstruction quality than both weighted and unweighted NUS. The reconstruction quality is also less dependent on the value of the seed in a pseudo-random number generator (Hyberts et al. 2010; Aoto et al. 2014; Mobli 2015). This dependence has been completely removed in deterministic sampling schemes (Worley and Powers 2015; Worley 2016). Jittered schemes with reduced seed variability have also been proposed (Mobli 2015; Craft et al. 2018). Mobli and Miljenović (Mobli and Miljenović 2019) also suggest “red-noise” (or “red-shift”) sampling, which is in a certain sense the opposite of blue-noise sampling. With red-noise sampling, stronger NUS artifacts appear in the middle of the PSF, while low-artifact regions are present at high frequencies.

Despite the widespread use of blue-noise sampling schemes, the theoretical basis for applying them to NMR spectra has never been fully formulated (Worley and Powers 2015). In fact, their effectiveness appears to contradict CS theory, which states that it is not possible to design any better sampling scheme than a fully random scheme, assuming the spectrum is sparse (Candès 2006). Surprisingly, however, it has been reported that PG schemes are particularly well suited for CS reconstruction using the hmsIST algorithm (Hyberts et al. 2012a), for example, but not so much for other methods (see analysis in Ying et al. 2017; Karunanithy and Hansen 2020). How then, we must ask, does CS theory explain the superiority of PG sampling in NMR? When we choose PG sampling, are we actually making any other tacit assumptions? Also, how can we explain the need for gap-size modulation: Is it only a matter of SNR? And finally, does the PG sampling generator always generate schemes with the Poisson distribution of gap size?

In this paper we attempt to answer these questions by presenting certain theoretical considerations, numerical simulations, and experimental analyses using 2D and 3D spectra. We show that PG, particularly with sinusoidal gap modulation, is superior to both weighted and unweighted random NUS when the spectrum is not just sparse but reveals *clustered sparsity*, that is to say, significant spectral points form a closely-spaced group. Our analysis of thousands of BMRB datasets reveals that this is often the case for the NMR spectra used for backbone assignment, which explains the empirically-observed superiority of PG and other similar sampling schemes. Clearly, the benefits of using PG sampling are more pronounced for some spectra (such as ${^13}$C HSQC) than for others that are less “clustered”. As reported by other investigators, reconstructions of some other (non-clustered) spectra can be even worse when using PG (Bostock et al. 2012; Mobli and Miljenović 2019; Roginkin et al. 2020). We believe that the understanding of relation between spectral clustering and time-domain sampling allows a more rational use of PG sampling schemes.

**Fig. 1:**
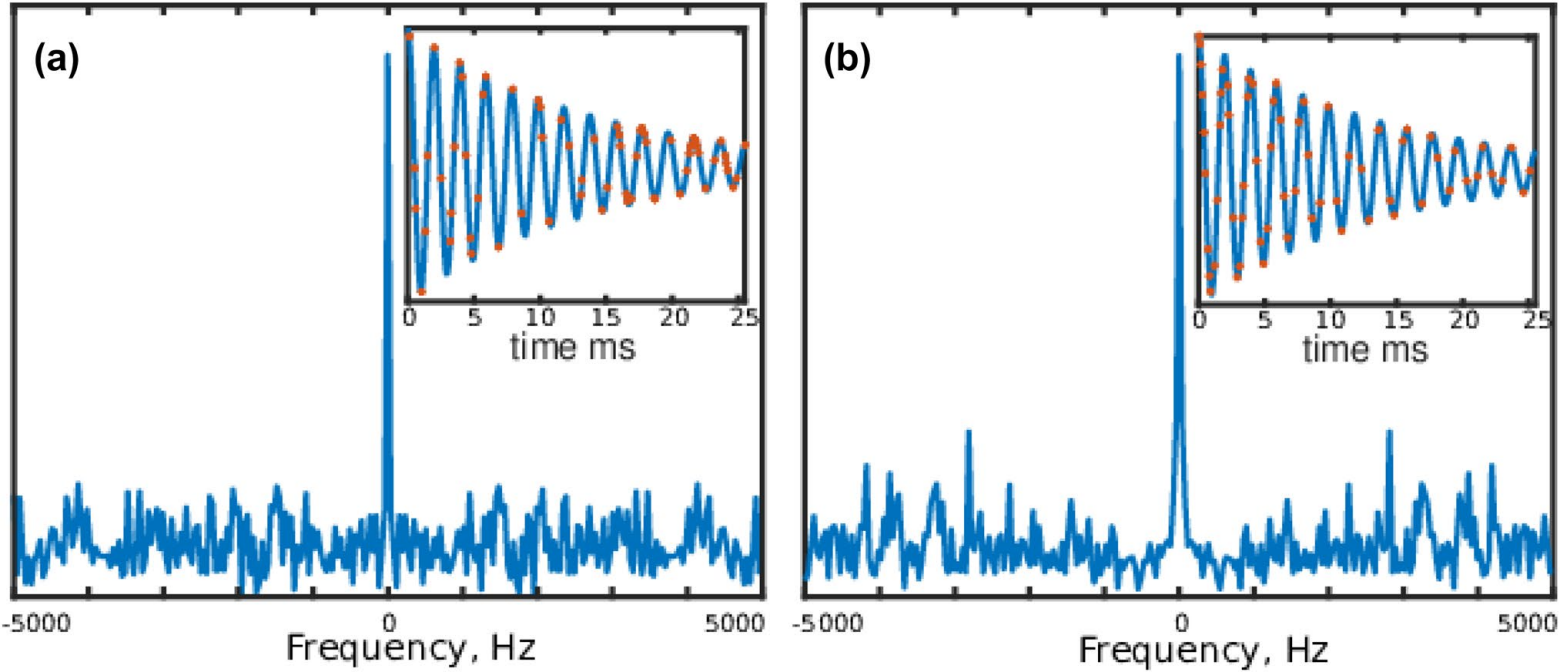
The point spread functions for unweighted random sampling (**a**) and an example of blue-noise sampling obtained using a Poisson-gap schedule generator with sinusoidal gap modulation (**b**) (Hyberts et al. 2010). The insets present the sampling of a typical FID signal. The large plots present the point-spread functions (PSFs), that is, the Fourier transforms of the sampling schedules. The blue-noise sampling “covers” the FID signal more evenly than unweighted random sampling. Consequently, the corresponding PSF has a non-uniform distribution of artifacts that are small in the peak’s vicinity and larger at greater distances from the peak (justifying the term “blue noise” or “high-frequency noise”)

## Theory

### Coherence of the FT matrix and the point spread function (PSF)

Let ${\mathbf{A}}\in \mathsf {Mat}_{m\times n}$ be a partial inverse FT obtained by removing $n-m$ rows of the full FT matrix according to the sampling schedule $(\iota_1,\iota_2,\ldots ,\iota_m)$

$$\begin{aligned}{\mathbf{A}}_{jk} = \frac{1}{\sqrt{n}}\exp \left( \frac{2\pi \imath }{n}(\iota_j-1)(k-1)\right).\end{aligned}$$

The quality (in terms of CS reconstruction) of the sampling schedule is reflected in the measurement matrix ${\mathbf{A}}$ and can be quantified in a number of different, but related, ways. The coherence $\mu ({\mathbf{A}})$ plays an important role here, and is defined as

$$\begin{aligned}\mu ({\mathbf{A}}) = \max_{k\ne k^{\prime}}|{\mathbf{a}}_k^\dagger {\mathbf{a}}_{k^{\prime}}|\end{aligned}$$

where ${\mathbf{a}}_k^\dagger$ denotes the conjugate transpose of the *k*th column of ${\mathbf{A}}$ and ${\mathbf{a}}_k^\dagger {\mathbf{a}}_{k^{\prime}}$ is the scalar product of the corresponding columns. The scalar product ${\mathbf{a}}_k^\dagger {\mathbf{a}}_{k^{\prime}}$ can be computed as

$$\begin{aligned} {\mathbf{a}}_k^\dagger {\mathbf{a}}_{k^{\prime}}&=\sum_{j=1}^m\overline{{\mathbf{A}}_{jk}}{\mathbf{A}}_{jk^{\prime}}\\ {}&=\frac{1}{n}\sum_{j=1}^m\exp \left( \frac{2\pi \imath }{n}(\iota_j-1)(k^{\prime}-k)\right) \end{aligned}$$

and related with the point spread function assigned to the sampling schedule $(\iota_1,\iota_2,\ldots ,\iota_m)$. The latter is defined as

$$\begin{aligned} \mathsf {PSF} = {\mathbf{A}}^\dagger {\mathbf{1}}_m\in {\mathbb {C}}^n\end{aligned}$$

where ${\mathbf{1}}_m$ is the vector of size *m* with all coordinates equal 1. Equivalently, $\mathsf {PSF}$ is the FT of the characteristic function of the sampling schedule, and we have

$$\begin{aligned}\mathsf {PSF}_k = \frac{1}{\sqrt{n}}\sum_{j=1}^m\exp \left( -\frac{2\pi \imath }{n}(\iota_j-1)(k-1)\right).\end{aligned}$$

Noting that

$$\begin{aligned}&\frac{1}{\sqrt{n}}\mathsf {PSF}_k={\mathbf{a}}_1^\dagger {\mathbf{a}}_{k} \\ &{\mathbf{a}}_k^\dagger {\mathbf{a}}_{k^{\prime}}=\frac{1}{\sqrt{n}}\mathsf {PSF}_{k^{\prime \prime}}\;\text{where}\; k^{\prime \prime} = (k-k^{\prime}+1)\mod n \end{aligned}$$

we conclude that $\mathsf {PSF}$ contains complete information about the correlations of columns of ${\mathbf{A}}$ and can serve as a visualization the of coherence. In fact, a number of researchers have examined the PSF concept, using the term ”peak-to-side-lobe ratio” when discussing the artifact level or, indeed, the coherence (see Table 1 in Mobli et al. 2012).

A typical spectrum obtained by the FT of the NUS data consists of many peaks, each generating its own artifact pattern. For this reason, the coherence is a coarse quantifier of the CS efficiency of the measurement matrix ${\mathbf{A}}$. Indeed, the artifact patterns overlap and only the sum of them reflects the difficulty of carrying out the reconstruction using CS algorithms.

A subtler and more fundamental quantity that takes into account the presence of multiple components in the reconstructed spectrum is the Restricted Isometry Property (RIP) constant. The matrix ${\mathbf{A}}$ is said to satisfy $(K,\delta )$-RIP if

2 $$\begin{aligned} (1-\delta )\Vert {\mathbf{x}}\Vert ^2\le \Vert \mathbf{Ax}\Vert ^2\le (1+\delta )\Vert {\mathbf{x}}\Vert ^2.\end{aligned}$$

for every *K*-sparse vector ${\mathbf{x}}$. For a given matrix ${\mathbf{A}}$, the RIP condition is, on the one hand, NP-difficult to check, but probabilistically generic on the other. If the RIP constant is sufficiently small and the vector ${\mathbf{x}}$ sufficiently sparse (for example, $\delta <\frac{1}{3}$ and $\Vert {\mathbf{x}}\Vert_0 \le {\frac{K}{2}}$, see Foucart and Rauhut 2010, Theorem 6.8), then ${\mathbf{x}}$ can be recovered as a unique solution of the CS problem:

$$\begin{aligned} {\mathbf{x}} = {{\,\mathrm{arg\,min}\,}}\{\Vert {\mathbf{z}}\Vert_1:\mathbf{Az} = \mathbf{Ax}\}.\end{aligned}$$

RIP takes into account the interference of artifact patterns originating in different spectral peaks and considers a worse-case scenario for a given spectral sparsity level. Thus, it is very useful for theoretical considerations, but in practice yields rather ”pessimistic” conditions, and perfect reconstruction often occurs even if RIP is not fulfilled. Coherence as defined above has a similar feature, especially in the context of NMR signals.

Finally, regarding the use of CS in the context of NMR, we note that: a) spectra are not strictly sparse, as even a single Lorentzian peak is built of all frequency-domain points; and b) the signal-to-noise ratio decays with evolution time. The latter point means that NUS schedules of decaying density are preferable. However, both coherence and RIP ignore SNR issues.

### Poisson-gap sampling

A random variable *X* is described by the Poisson distribution with a parameter $\lambda >0$ and we write $X\sim \text{Pois}(\lambda )$ if for $k=0,1,2,\ldots$, the probability that $X=k$ is given by

$$\begin{aligned}\text{Pr}(X=k) = \frac{\lambda ^ke^{-\lambda }}{k!}.\end{aligned}$$

The mean value $\text{E}(X)$ of *X* and its variation $\text{Var}(X)$ are both equal $\lambda$.

**Fig. 2:**
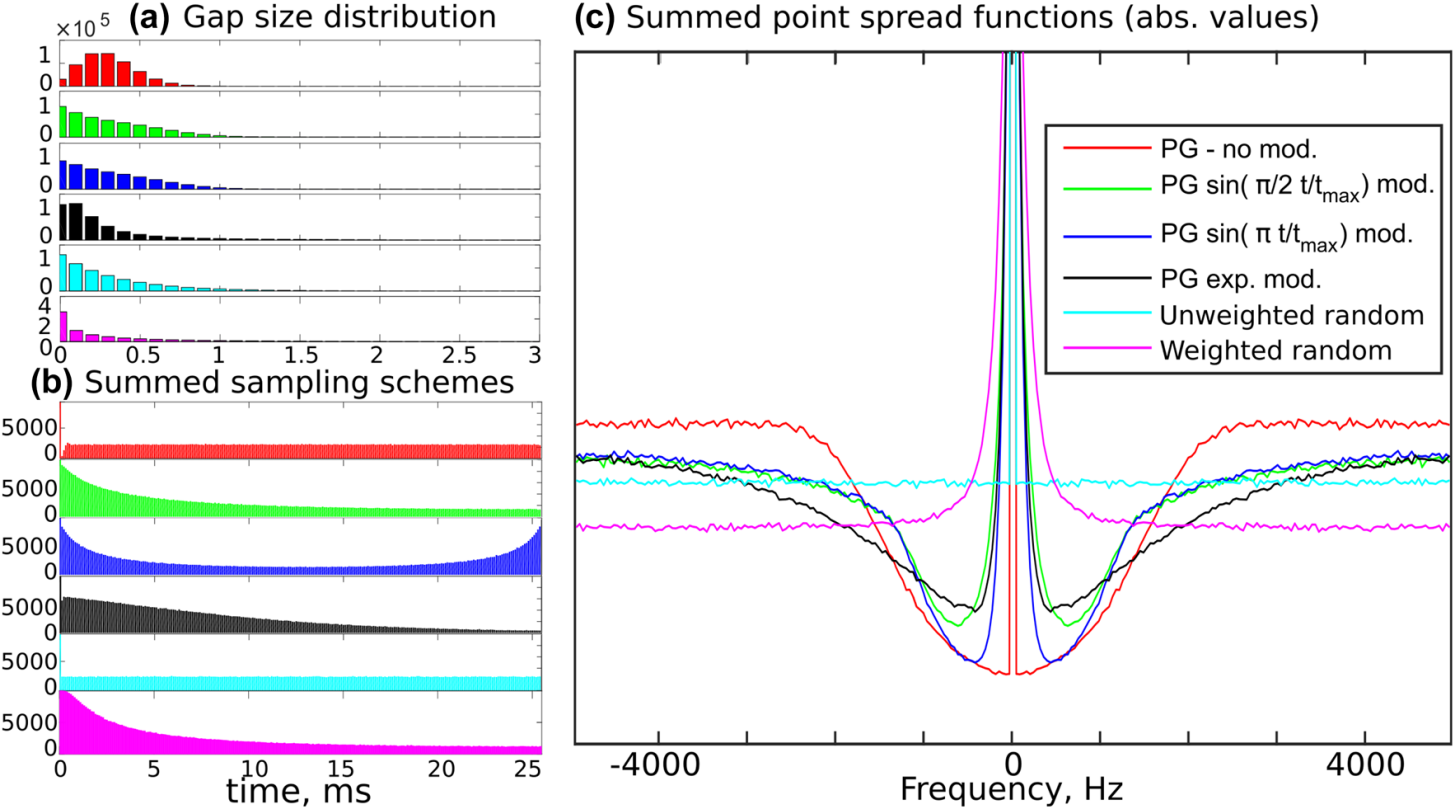
The sampling scheme statistics and artifact distributions of Poisson-gap sampling without gap size modulation (red), $\sin \left( \frac{\pi }{2}\frac{t}{t_{\text{max}}}\right)$ modulation (green), $\sin \left( \pi \frac{t}{t_{\text{max}}}\right)$ modulation (dark blue), exponential modulation (black), unweighted random sampling scheme (light blue), and random weighted using $\sin ^{-1}\left( \frac{\pi }{2}\frac{t}{t_{\text{max}}}\right)$ sampling density (magenta). The panels show the summed results of 10,000 sampling schemes (64 points from the grid of 256) obtained using different seeds from a pseudo-random number generator: **a** gap size distributions, **b** summed sampling schemes (note that all schedules always contain 0th increment), and **c** averaged absolute values of PSFs (only the bottom part of a PSF is shown). The gap-size distribution (**a**) is related to the artifact distribution in (**c**), while the distribution of points (**b**) affects the linewidth

In PG sampling we want the distribution of gaps between sampling points to follow the Poisson distribution. Figure 2a) shows that the distribution does indeed differ significantly from that of fully-random NUS.

**Fig. 3:**
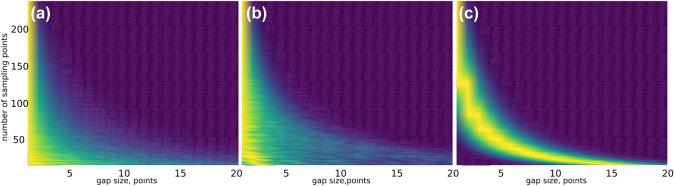
The series of histograms of gap size in NUS schedules at different sampling levels up to full sampling (256 points). Histograms were obtained by averaging 25 schedules. **a** Unweighted random sampling, **b** Poisson-gap sampling with $\sin \left( \frac{\pi }{2}\frac{t}{t_{\text{max}}}\right)$ gap-size modulation, and **c** Poisson-gap sampling without gap-size modulation. Above an approximately 50% sampling level there is no significant difference between schedules in terms of gap-size distribution

In practice, the PG sampling schedule is generated using Knuth’s algorithm (Knuth 1997). An obvious observation—although one that is rarely made—is that the schedule roughly follows Poisson distribution only for a certain range of sampling levels. The histograms in Fig. 3 show that, over a certain sampling level (approximately 50% in the case shown), PG is almost indistinguishable from random sampling in terms of gap distribution, and that gap-size modulation amplifies this effect.

The original work on the application of PG in NMR states that avoiding large gaps between sampling points is particularly important in the initial and final parts of the signal (Hyberts et al. 2010). Usually, due to the signal’s decay and apodization, we ignore the final part of the signal and the gap size is modulated with a quarter of sine, which results in a denser sampling of the initial part only (Hyberts et al. 2012b). While Hyberts et al. propose sinusoidal gap modulation (Hyberts et al. 2010), a classical, exponentially decaying density has been also used for this kind of sampling scheme (Kazimierczuk et al. 2008). Obviously, any modulation causes the sampling scheme to deviate from the Poisson distribution (see Fig. 2a) and 3). However, as shown below, it may improve its effectiveness in the case of clustered spectra.

Before PG became popular, off-grid Poisson-disk sampling (Kazimierczuk et al. 2008), known from image processing (Mitchell 1987), was used in NMR. The use of Poisson distribution in image processing is to some extent justified by the natural Poisson-disk distribution of the receptors in the retina (Yellott 1982). Moreover, it results from the methods used to generate Poisson-disk schedules, for example, variants of the dart-throwing algorithm (Lagae and Dutré 2008), even if the Poisson distribution of gaps is not explicitly implemented in the code.

### Blue-noise point spread function

Poisson-gap sampling can be thought of as a type of NUS whose properties are somewhere between unweighted random NUS and conventional regular sampling. Other variants include jittered sampling (Kazimierczuk et al. 2007; Mobli 2015), quantile sampling (Craft et al. 2018), sine-gap sampling (Worley and Powers 2015) and Poisson-disk sampling (Kazimierczuk et al. 2008). Their common feature is that the coverage of the sampled time domain is more even than in the case of unweighted random NUS. This is reflected in the distribution of the gaps between sampling points. As demonstrated by (Kazimierczuk et al. 2008), the sharper distribution leads to smaller artifacts near the peak (see Fig. 2). At higher frequencies, the artifacts in the PSF (that is, coherence) become larger, generally exceeding those found in unweighted random PSF. These high-frequency artifacts justify the name “blue noise”, which describes all kinds of pseudo-random schedules with a restricted distance between sampling points.

The fact that increased artifacts occupy the high-frequency region in the PSF does not mean that they appear in the high-frequency region of the spectrum, of course. Each peak in the spectrum obtained by a direct FT of the NUS data (with missing points replaced by zeros) is a perfect, usually Lorentzian spectral line convoluted with the PSF.

High artifacts in the PSF (high coherence) indicate possible difficulties with the reconstruction. This can be easily understood from the block diagrams of the NUS reconstruction algorithms (see figures in Shchukina et al. 2017): The reconstruction algorithms usually start from the direct FT of the NUS data, that is, the spectrum convoluted with PSF, and then, in an iterative fashion, deconvolve PSF from the peaks (or their fractions, as in the iterative soft thresholding method). Higher artifacts may cause reconstruction imperfections, especially if they overlap with peaks. However, there are other factors determining the reconstruction quality, apart from the artifacts’ level as generated by the sampling schedule. One such factor is the decay of the signal, which promotes a decaying sampling density, even though it worsens coherence (Kazimierczuk et al. 2014). Another factor is clustered sparsity, which promotes various forms of blue-noise sampling, as discussed in this paper.

### Clustered sparsity in NMR spectra

**Fig. 4:**
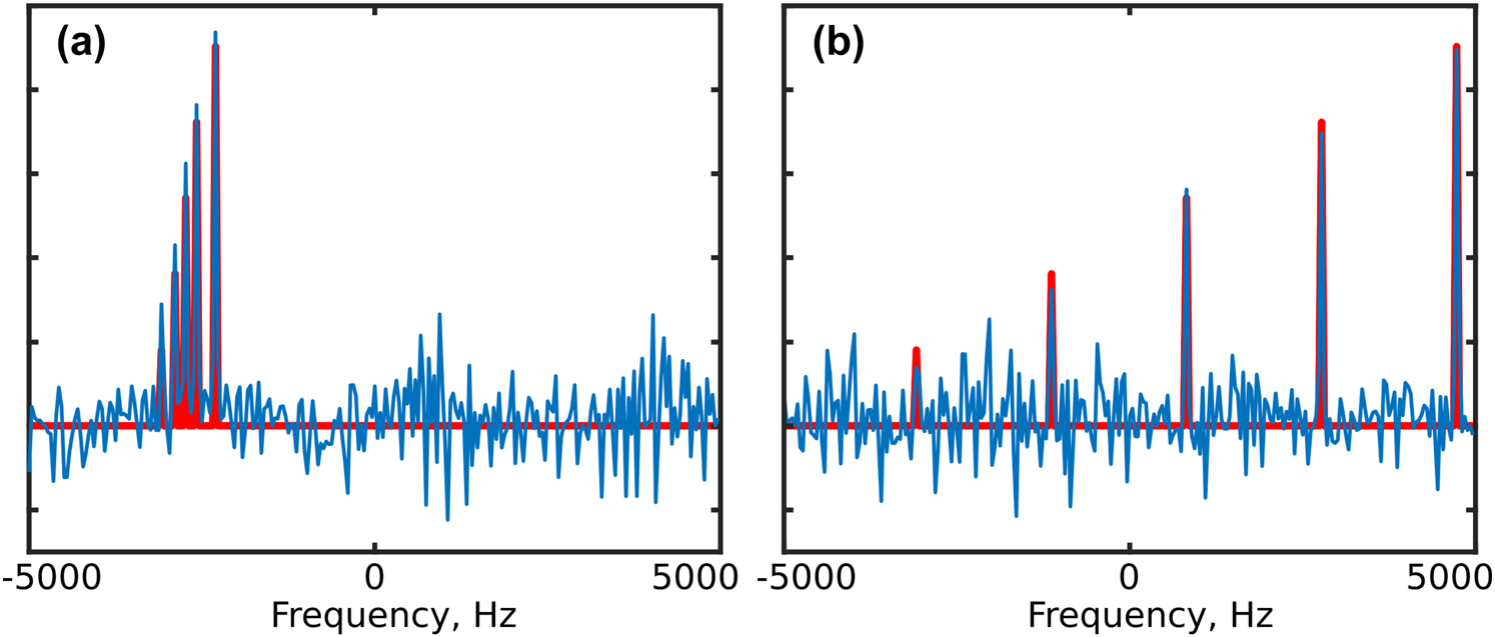
Different kinds of sparsity and a blue-noise sampling. Both spectra were obtained by means of a Fourier transform of a NUS signal sampled using the PG schedule as in Fig. 1b. The spectrum obtained using full sampling is shown in red. Five peaks are convoluted with a blue-noise PSF, and are thus less covered by the artifacts when their positions are clustered (as in (**a**)) than when they are uniformly spread over the whole frequency band (as in (**b**)). In **b** the smallest peak on the left completely disappears under the artifacts. As the FT of the NUS data is the starting point for most reconstruction algorithms, the artifact distribution is a good indicator of the difficulty of the reconstruction

The shape of blue-noise PSF (Figs. 1b and 2c) suggests when (that is, in which cases) blue-noise sampling may be superior to weighted or unweighted random schemes—namely, cases where the peaks form closely-spaced groups and thus occupy each other’s low-artifact regions. The results of the simulation shown in Fig. 4 illustrate this nicely: Both spectra have the same sparsity and the same measurement matrix (and thus, the same coherence of ${\mathbf{A}}$). However, they present a different difficulty level for the reconstruction algorithm, which would deal with the clustered case much more easily (see simulations below).

**Fig. 5:**
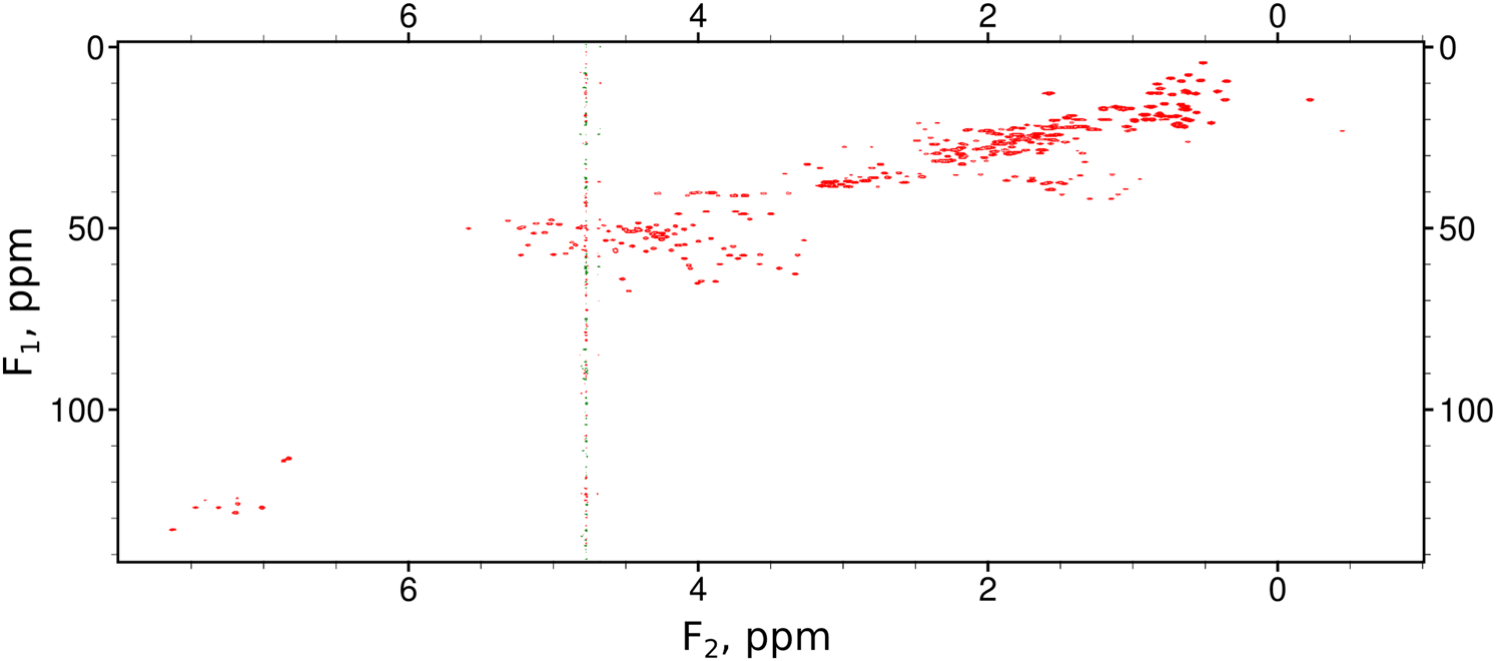
Example of clustered sparsity: ^13^C HSQC of human ubiquitin. Each column of the data matrix (each point on the direct dimension) contains peaks that are (1) sparse, and (2) clustered (that is, they occupy a narrow range compared to the full $F_1$ band). While condition (1) makes ^13^C HSQC suitable for NUS reconstruction, condition (2) makes blue-noise sampling (such as PG sampling) preferable to weighted or unweighted random sampling

A natural question that might arise regarding the clustered case would be: Why not just reduce the spectral width during the acquisition of the signal? While this would be justified in the case of one-dimensional signals, the situation is different in cases of two or more dimensions. For example, in a typical ^13^C HSQC of a protein (see Fig. 5), peaks form groups in an indirect dimension, but the position of each group is different for each point of the direct dimension. Aromatic protons (6.5-8 ppm) are correlated with aromatic carbons (120-160 ppm), aliphatic protons (0-3 ppm) with aliphatic carbons (0-50 ppm), and so on. The clustering relative to the full spectral width is additionally enhanced by the practice of setting the sampling rate to a slightly higher level than the Nyquist rate, which leads to peak-free margins on both sides of the spectrum. However, a 2D signal consisting of all correlations is measured at once, so in a standard HSQC we cannot set different excitation bands for different spectral points on the direct dimension. In this sense, multidimensional NMR spectroscopy is exceptional among the areas where CS can be applied, in that the NMR spectrum contains only *K* significant points (in other words, it is *K*-sparse), and *in addition* we know that these points probably form a closely-spaced group, but we do not know where that group is located. This special case of *K*-sparsity is not covered by the standard RIP characterization of the sampling schedules.

### Null space property corresponding to clustered sparsity

The mathematical theory of compressed sensing focuses on the properties of a measurement matrix ${\mathbf{A}}\in \mathsf {Mat}_{m\times n}$ (for example, a partial inverse Fourier matrix in the case of NMR) with respect to the class of sparse vectors ${\mathbf{x}}\in {\mathbb {C}}^n$. As $m<n$, there are at least $n-m$ independent directions in ${\mathbb {C}}^n$ for which the measurement yields no information whatsoever.

The null space property of ${\mathbf{A}}$ with respect to the supporting set $S\subset \{1,2,\ldots , n\}$ (see Foucart and Rauhut 2010, Definition 4.1) guaranties that no information is lost in the measurement of the *S*-supported elements ${\mathbf{x}}\in {\mathbb {C}}^n$. Remarkably, this property is equivalent to the uniqueness of the solution of the $\ell_1$-minimization problem

$$\begin{aligned} {\mathbf{x}} = {{\,\mathrm{arg\,min}\,}}\{\Vert {\mathbf{z}}\Vert_1:\mathbf{Az} = \mathbf{Ax}\}\end{aligned}$$

for vectors ${\mathbf{x}}\in {\mathbb {C}}^n$ supported on *S* (Foucart and Rauhut 2010, Theorem 4.4).

As we describe elsewhere in this article, the effectiveness of blue-noise sampling (for example, PG) is closely related to the clustering of the peaks. This motivates the following version of (Foucart and Rauhut 2010, Definition 4.1):

#### Definition 1

We say that a matrix ${\mathbf{A}}\in \mathsf {Mat}_{m\times n}$ has a $(\Lambda ,K)$-null space property if for every $\Lambda$-clustered subset $S\subset \{1,\ldots ,n\}$ of cardinality not greater then *K*, we have $\Vert {\mathbf{x}}_S\Vert_1<\Vert {\mathbf{x}}_{{\overline{S}}}\Vert_1$ whenever ${\mathbf{A}}{\mathbf{x}} = 0$, ${\mathbf{x}}\ne 0$ where ${\overline{S}}$ denotes the complement of *S* and ${\mathbf{x}}_S$ is the restriction of the vector ${\mathbf{x}}$ to the subset $S\subset \{1,\ldots ,n\}$.

In other words, a $(\Lambda ,K)$-null space space property means that whenever the measurement of ${\mathbf{x}}$ modeled by ${\mathbf{A}}$ yields no information, ${\mathbf{A}}{\mathbf{x}}=0$, then the vector ${\mathbf{x}}$ must be strongly unclustered, in the sense that the contribution to $\Vert {\mathbf{x}}\Vert_1$ of an arbitrary *K*-sparse cluster *S* of size $\Lambda$ is always smaller then the contribution of the complement ${\overline{S}}$ of *S*, $\Vert {\mathbf{x}}_S\Vert_1<\Vert {\mathbf{x}}_{{\overline{S}}}\Vert_1$. We should add that if a vector ${\mathbf{x}}$ is supported by a *K*-sparse cluster *S* of size $\Lambda$ (which can be written as $\Vert {\mathbf{x}}_{{\overline{S}}}\Vert_1=0$) and its measurement yields no information, ${\mathbf{A}}{\mathbf{x}}=0$, then ${\mathbf{x}}=0$. Indeed, if ${\mathbf{x}}\ne 0$, then using Definition 1 we would have $\Vert {\mathbf{x}}_S\Vert_1 <\Vert {\mathbf{x}}_{{\overline{S}}}\Vert_1 = 0$, which is a contradiction.

Repeating the reasoning yielding the proof of (Foucart and Rauhut 2010, Theorem 4.5) gives us the following:

#### Theorem 1

Given a matrix ${\mathbf{A}}\in \mathsf {Mat}_{m\times n}$, every $\Lambda$-clustered *K*-sparse vector ${\mathbf{x}}$ is the unique solution of the CS-problem

$$\begin{aligned} {\mathbf{x}} = {{\,\mathrm{arg\,min}\,}}\{\Vert {\mathbf{z}}\Vert_1:\mathbf{Az} = \mathbf{Ax}\} \end{aligned}$$

if and only if ${\mathbf{A}}$ has $(\Lambda ,K)$-null space property.

**Fig. 6:**
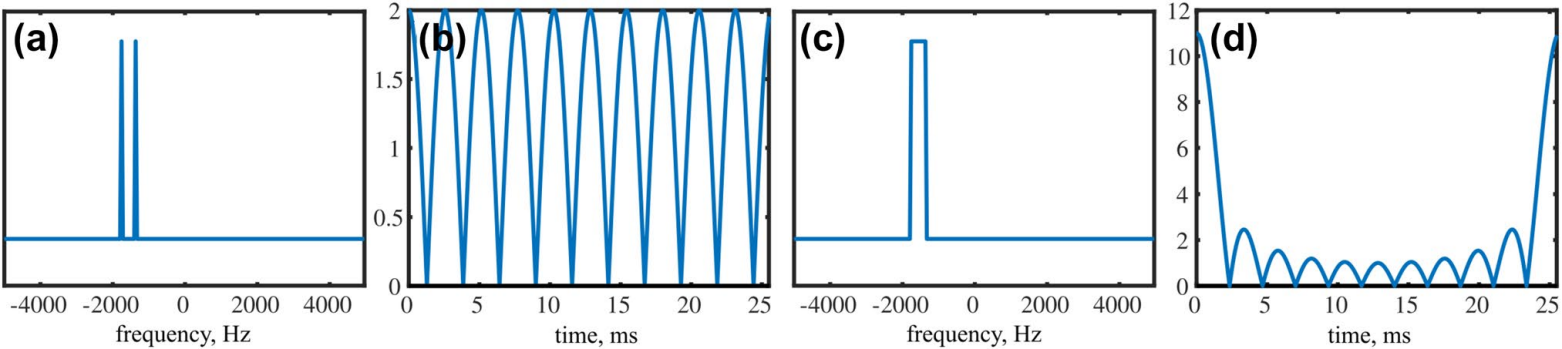
Amplitude modulations in clustered signals. Two extreme examples of time-domain signals with clustered spectra are shown. The figure shows the following: **a** a spectrum with two close peaks at frequencies separated by $\Delta \omega$; **b** the absolute value of the corresponding complex signal with an amplitude modulation of $\cos \left( \Delta \omega t \right)$ (“beating”); **c** the spectrum with a cluster of size $\Delta \omega$ filled with peaks; and **d** the absolute value of the corresponding complex signal. The shape of the time-domain signals suggests how we can avoid sampling close to zero-crossings, which is important due to SNR loss and the requirements of the compressed sensing theory (null-space property)

Proving that blue-noise sampling gives rise to the measurement matrix satisfying $(\Lambda ,K)$-null space property is beyond the scope of this article. Nevertheless, this assumption is useful for justifying the choice of Poisson sampling for recovering clustered spectra. Indeed, PG and other blue-noise schemes avoid a situation in which ${\mathbf{A}}{\mathbf{x}} \approx 0$, if ${\mathbf{x}}$ is clustered and if the average gap size is small enough. Figure 6 presents two examples of signals with clustered spectra. As can be seen from the examples, clustering generally results in the suppression of both real and imaginary parts of the signal at regular intervals (known as “amplitude modulation” or ”beating”).

How, then, can we avoid sampling that results in ${\mathbf{A}}{\mathbf{x}} \approx 0$, that is to say, how can we avoid taking samples close to the zeros of the amplitude modulation? If we restrict the distance between points (gap size), then the points will not gather around zero crossings, which explains why PG may work better for clustered signals than weighted or unweighted random NUS. However, this advantage is revealed only if the average gap is small enough compared to the amplitude modulation frequency, which is related to the inverse of the cluster size.

Figure 6d explains why $\sin \left( \pi \frac{t}{t_{\text{max}}}\right)$ gap modulation is particularly effective when the cluster is full of peaks, so to speak. Essentially, it provides denser sampling near the maxima of the amplitude modulation in the initial and final parts of the signal. Thus, the first iteration of a CS algorithm (for example, IST) generates an input that is close to the proper solution, that is to say, one with more experimental data near the beginning and the end, and zeros in the middle part, where the reconstructed signal should be close to zero anyway.

Of course, the sample signals in Fig. 6 are simplified compared to the real NMR signal, inasmuch as they are noiseless, do not decay, and have peaks of equal amplitude. Nevertheless, it is generally true that clustering leads to amplitude modulation of the resulting signal, which suggests that some form of constrained sampling may be better than unweighted random NUS. Moreover, the more peaks there are in the cluster, the closer we are to the situation in Fig. 6d, which justifies the use of denser sampling in the initial and final parts of the signal. The SNR is also important, as avoiding sampling of the zeros of the amplitude modulation enhances the sensitivity in the same way as sampling matched with J-coupling constants (Jaravine et al. 2006; Kazimierczuk et al. 2020) or chemical shifts (Schuyler et al. 2011; Shchukina et al. 2019).

## Simulations

**Fig. 7:**
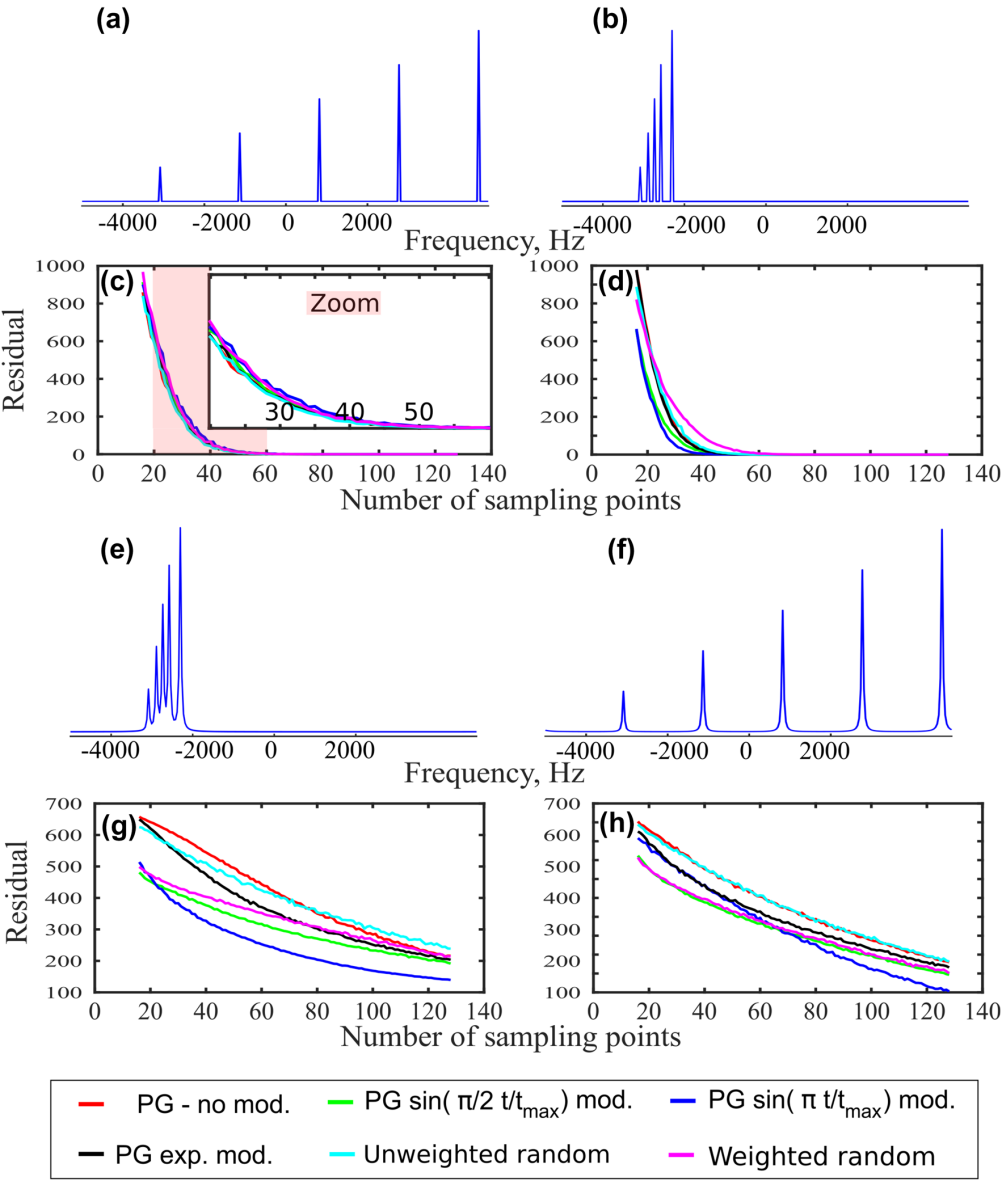
Efficiency of different sampling schemes for various kinds of sparsity. **a**, **b**, **e**, and **f** show fully-sampled spectra, used as a reference for calculating the reconstruction residuals presented in (**c**, **d**, **g**, and **h**). Reconstruction of the spectra was performed using iterative soft thresholding in a variant similar to hmsIST (Hyberts et al. 2012a), also referred to as IST-D (Sun et al. 2015; Shchukina et al. 2017). All signals contained five components with amplitudes in the ratio of 1 : 2 : 3 : 4 : 5. In **a** and **f** the signals were equally spread over the frequency domain, while in **b** and **e** they were clustered. In **e** and **f** the signals decayed, leading to Lorentzian peaks (approximating a sparse spectrum), while in **a** and **b** the signals did not decay, leading to single-point peaks (a strictly sparse spectrum). All signals were noiseless. Five hundred different random generator seeds were used for each sampling level. The number of points used was 16, 17 … 128 from the full grid of 256. The sampling scheme generators and colors are the same as in Fig. 2. The weighted random (magenta) is generated using $\sin ^{-1}\left( \frac{\pi }{2}\frac{t}{t_{\text{max}}}\right)$ sampling density and thus in a real experiment it would have the equivalent sensitivity to PG with $\sin \left( \frac{\pi }{2}\frac{t}{t_{\text{max}}}\right)$ gap size modulation (green). Averaged residuals are shown

To verify our hypothesis that clustered sparsity is a prerequisite for the successful use of PG sampling in CS reconstruction, we performed several sets of simulations. We simulated four noiseless signals, each consisting of five components. The signals differed in their relaxation rate and the relative positions of the peaks: The peaks were either broad (Lorentzian) or (infinitely) narrow, and either clustered or equally spread over the frequency domain. The signals were undersampled at different NUS levels, using from 16 to 128 points taken from a 256-point grid, according to weighted and unweighted random schemes and PG with various kinds of modulation, corresponding to Fig. 2. Five hundred random generator seeds were used. The fully-sampled spectra and the averaged reconstruction residuals are shown in Fig. 7.

We include the non-decaying signals in the simulations in order to link the conclusions with the theory of compressed sensing. They also correspond to the constant-time dimensions used in NMR experiments.

### Clustering analysis of BMRB data

We analyzed 8934 entries from BMRB (Ulrich et al. 2008) containing chemical shifts of ^15^N, ^13^C and ^1^H. For each entry we simulated a series of spectra: 2D ^13^C HSQC, 2D ^15^N HSQC, 3D HNCA, and 3D HNCACB.

For 2D spectra we assumed that each peak occupied the region of 0.02 ppm in the direct dimension and allocated the other chemical shifts accordingly to groups belonging to the same column of a 2D spectrum. We calculated the standard deviation of all peaks in each column and normalized it to the distance between the maximum and minimum indirect dimension chemical shifts for a given protein. Finally, we plotted the histograms of the distances.

For the 3D spectra, the procedure was very similar, but we analyzed the clustering using 2D cross-sections instead of 1D columns. We calculated the clustering as the square root of the sum of squared standard deviations for both indirect dimensions, normalized similarly as 2D spectra, as shown in the equation below:

3 $$\begin{aligned} H_j=\frac{2 \sqrt{\sum_{i=1}^{M} \sigma ^2\left( \mathbf{\delta }^j_i \right) }}{\sqrt{\sum_{i=1}^{M} \text{SW}_i^2}}, \end{aligned}$$

where $H_j$ describes the clustering of peaks on the cross-section corresponding to the *j*th point of the direct dimension; $\mathbf{\delta }^j=\left( \mathbf{\delta }_i^j\right)_{i=1,\ldots , M}$ is the vector of chemical shifts for peaks in the *i*th indirect dimension for a given *j*; *M* is the number of indirect dimensions; and $SW_i$ are spectral widths in the indirect dimensions tailored exactly to the extreme positions of peaks in the whole dataset. The histograms of the results are shown in Fig. 9.

## Experimental data

We analyzed a 2D ^13^C HSQC and a 2D ^15^N HSQC of a human ubiquitin, as well as 3D HNCA and 3D HNCACB of azurin. All spectra were collected using full sampling and artificial undersampling.

The 2D experiments were carried out using a Varian 700 MHz DDR2 spectrometer equipped with a triple-resonance HCN probe. Measurements were performed at 298 K using a sample of human ubiquitin (1 mM in 10:90 D2O/H2O, 50 mM phosphate buffer and 0.02% NaN3, pH 7.1, ^13^C,^15^N-labeled, obtained from Giotto Biotech). The indirect dimension sampling grid was 512 for ^13^C HSQC and 256 for ^15^N HSQC. We set the number of scans to four.

The 3D HNCACB and 3D HNCA were performed on the sample of azurin (Karlsson et al. 1989), which was also double-labeled with the concentration of 1 mM in the phosphate buffer sample (pH 5). The spectra were recorded using a Varian 800 MHz UNITY Inova spectrometer with a room-temperature HCN probe. The indirect dimensions were sampled according to the full grid of $150\times 55$ (3D HNCACB) in ^13^C dimensions and $55\times 75$ (3D HNCA) in ^15^N dimensions. We set the number of scans to four.

We performed the processing and analysis using a custom-made Python script (Helmus and Jaroniec 2013; Harris et al. 2020; Wes McKinney 2010), the *mddnmr* package (Orekhov et al. 2021), and a PG sampling generator taken from (Hyberts et al. 2010). We artificially generated NUS datasets from the full data for a broad range of sampling levels (26, 28,…, 100 points for ^15^N HSQC, 26, 28,…, 200 points for ^13^C HSQC, 300, 400,…, 1100 points for 3D HNCA, and 300, 400,…, 2200 points for 3D HNCACB), using two different sampling strategies: $\sin (\frac{\pi }{2} \frac{t}{t_{\text{max}}})$-modulated PG, and random with $\sin ^{-1}(\frac{\pi }{2} \frac{t}{t_{\text{max}}})$-weighted density of points. To generate schedules for 3D experiments we used the “add” strategy from (Hyberts et al. 2014). Generators did not allow repetitions of sampling points. We used ten NUS generator seeds for each sampling level.

**Fig. 8:**
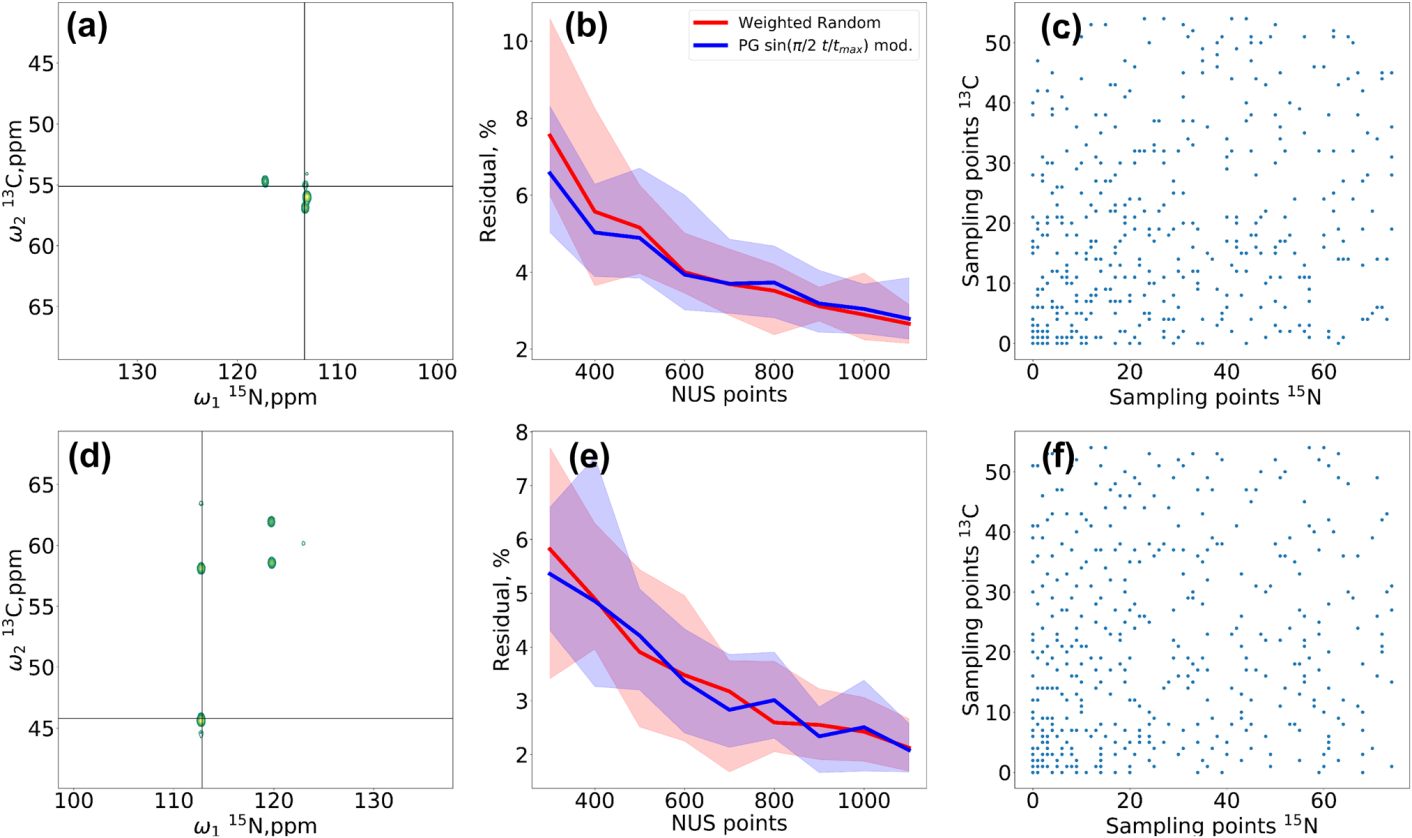
Two cross-sections from 3D HNCA spectrum of the azurin, differing by clustering level (**a**, **d**), and reconstruction residuals (**b**, **e**) for the marked peaks ($\pm \,3$ spectral points from the peak top) calculated at NUS levels up to 25% (300, 400...1100 points from a $55\times 75$ grid) using two different sampling schemes: PG with gap size modulated with $\sin \left( \frac{\pi }{2}\frac{t_1+t_2}{t_{\text{1max}}+t_{\text{2max}}}\right)$ (blue) and random sampling with density modulated by $\sin ^{-1}\left( \frac{\pi }{2}\frac{t_1+t_2}{t_{\text{1max}}+t_{\text{2max}}}\right)$ (thus, both having a similar time-domain SNR). Residuals were averaged over ten different schedule-generator seeds for each NUS level and shown as a percentage of the norm of a given peak from full sampled spectra. The colored bands represent the distribution of residuals for different seeds. The full sampling grid of $55\times 75$ was used in ^13^C and ^15^N dimensions. **c** is an example of a sampling schedule for weighted random, **f** is an example of a sampling schedule for PG

We reconstructed the missing data points using the IST algorithm with 200 iterations and a virtual-echo option (Mayzel et al. 2014). The residual of the reconstruction was calculated for each peak individually as the sum of the “local” differences ($\pm 3$ points from the peak top) between the fully-sampled and reconstructed spectra. Sample cross-sections from 3D HNCA are shown in Fig. 8. The residuals and clustering histograms, calculated in the same way as for BMRB data, are shown in Fig. 10. For indirect dimension cross-sections and residuals as a function of the sampling level for each peak of each spectrum, see Supplementary Information.

**Fig. 9:**
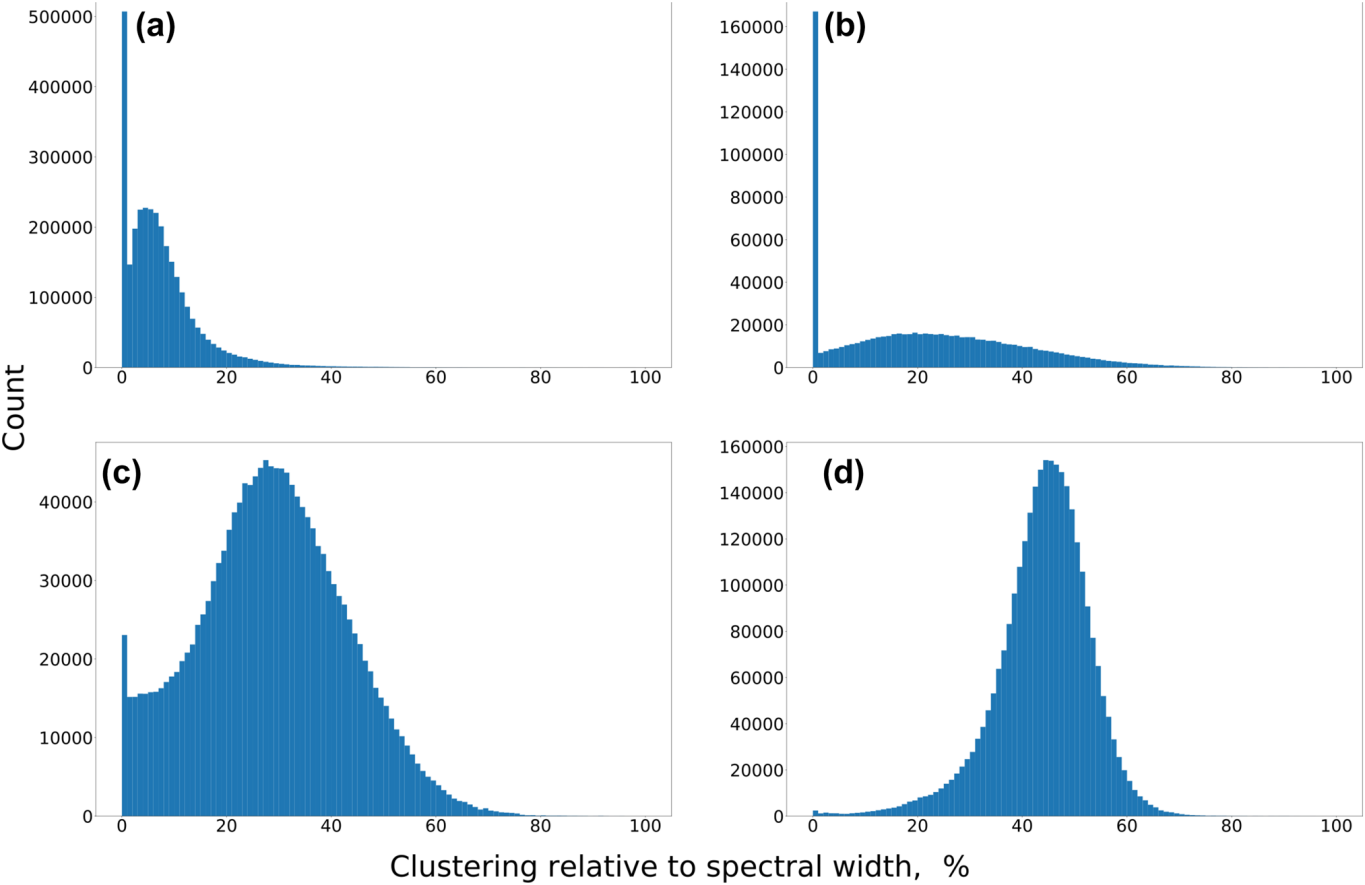
Analysis of peak clustering based on 8934 protein datasets from BMRB: **a** 2D ^13^C HSQC, **b** 2D ^15^N HSQC, **c** 3D HNCA, and **d** 3D HNCACB. The clustering is defined as a standard deviation of chemical shifts on a cross-section obtained for a given ^1^H coordinate, related to the spectral width, defined as the maximum distance between all peaks in the indirect dimensions for a given protein. The large value at 0 clustering corresponds to the cross-sections containing only a single peak

**Fig. 10:**
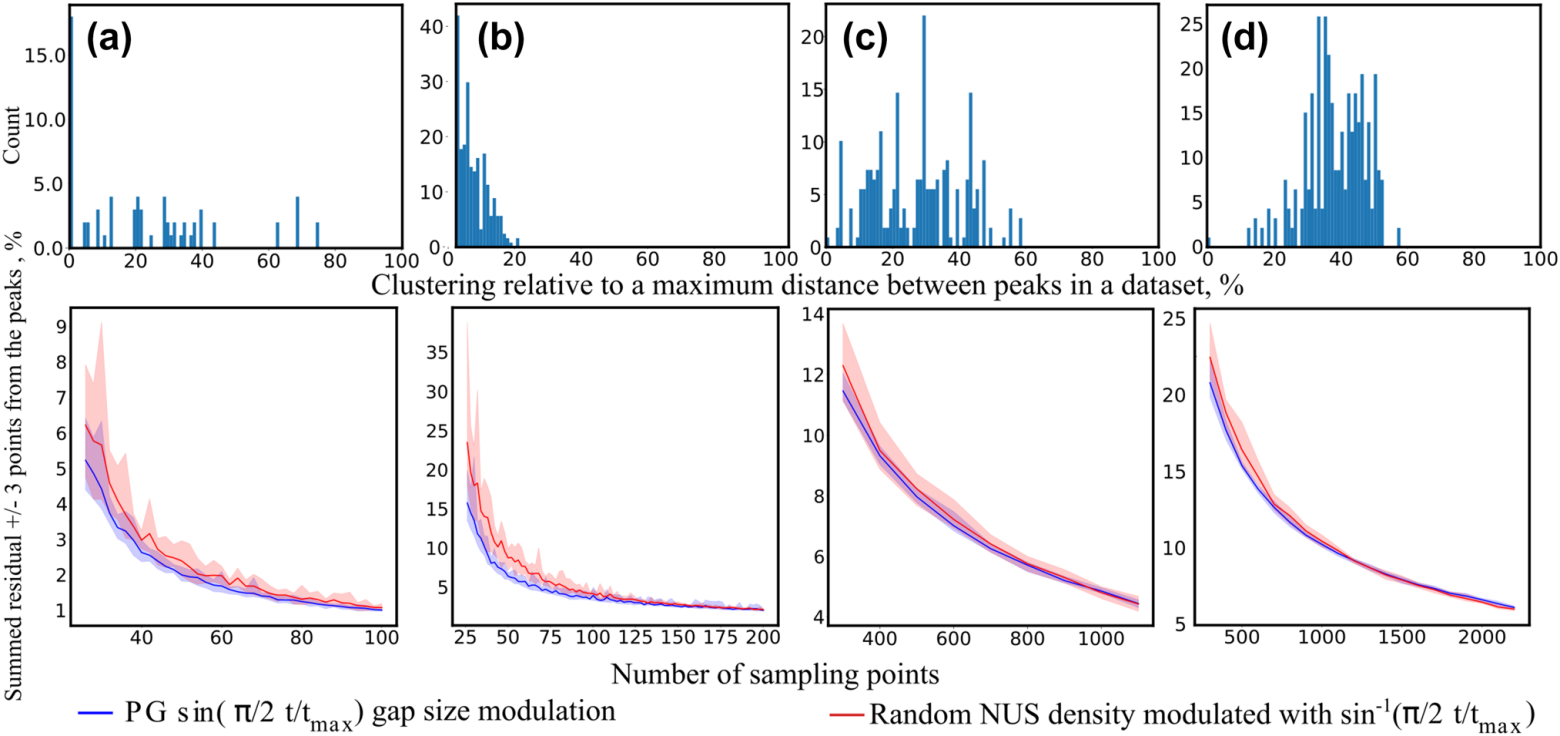
Peak clustering (upper panels) and residuals of CS reconstruction (lower panels) in various NMR spectra of ubiquitin (**a** and **b**) and azurin (**c** and **d**). We used two sampling schemes: PG with $\sin (\frac{\pi }{2}\frac{t}{t_{\text{max}}})$ (2D) or $\sin (\frac{\pi }{2}\frac{t_1+t_2}{t_{1\text{max}}+t_{2\text{max}}})$ (3D) gap size modulation (blue), and sensitivity-equivalent random NUS (blue) with sampling density decaying as $\sin ^{-1}(\frac{\pi }{2}\frac{t}{t_{\text{max}}})$ (2D) or $\sin ^{-1}(\frac{\pi }{2}\frac{t_1+t_2}{t_{1\text{max}}+t_{2\text{max}}})$ (3D). The colored bands show the distribution of the residuals among ten random generator seeds, while the lines show the average residual over ten seeds. The residuals were calculated as an $\ell_2$-norm of the difference between fully-sampled and reconstructed spectra in the peak regions, that is, the cubes of $\pm 3$ points around the tops of the peaks. They are shown as a percentage of the norm of a given peak from full sampled spectra. The clustering was calculated in the same way as in Fig. 9. We analyzed the following datasets: **a** 2D ^15^N HSQC (26, 28...100 points from a full sampling grid of 256 points), **b** 2D ^13^C HSQC (26, 28...200 points from a full sampling grid of 512 points), **c** 3D HNCA (300, 400...1100 points from a full sampling grid of $75\times 55$ points in ^15^N × ^13^C), and **d** 3D HNCACB (300, 400...2200 points from full a sampling grid of $55\times 150$ points in ^15^N × ^13^C). The average linewidths and spectral widths in the indirect dimensions were: **a** 11 Hz/2600 Hz, **b** 100 Hz/27,000 Hz, **c** ^13^C: 110 Hz/5900 Hz, ^15^N: 40 Hz/3200 Hz, and **d** ^13^C: 110 Hz/16,090 Hz, ^15^N: 55 Hz/3200 Hz. The tested sampling levels reached 39% in 2D and 25% in 3D spectra

## Results and discussion

Our analysis shows that the fundamental concepts of CS theory—RIP and coherence—do not explain the efficiency of blue-noise sampling schemes in NMR. Indeed, coherence, visualized as the highest artifact in the PSF, and RIP, as a worst-case overlap of artifact patterns originating from peaks located at arbitrary positions, are on average better in the case of unweighted random NUS than for any other kind of sampling, including PG. This is reflected in the quality of the CS reconstruction of strictly sparse spectra with arbitrary peak positions (Fig. 7a). Clearly, in this standard CS problem, there is no better sampling than that generated using an unweighted random scheme.

However, coherence and RIP ignore features of NMR spectra other than sparsity. Two of these features are ubiquitous, namely the signal’s decay (Lorentzian lineshape) and peak clustering. The signal’s decay would lead us to favor the use of NUS schemes with decaying density, while peak clustering would lead us to favor blue-noise sampling. Some spectroscopists have suggested that combining the two approaches—in other words using sinusoidally-modulated PG sampling—might be the optimal solution (Hyberts et al. 2010, 2012b). However, many other examples appear to contradict this (Bostock et al. 2012; Craft et al. 2018; Roginkin et al. 2020) and the theory-based recommendations about when to use PG are not clear.

Panels b and c in Fig. 2 confirm the fact, already established by (Tarczyński et al. 2004; Kazimierczuk et al. 2007), that the decay of the sampling density according to a certain function has a similar effect on the PSF as the corresponding apodization, that is to say, it broadens the spectral line. Of course, this broadening is not observed in the reconstructed spectrum if the missing points are successfully calculated.

On the other hand, as shown by panels a and c in Fig. 2, sharpness of the gap-size distribution is associated with a difference between the artifact level near the peak and far from it, as reported earlier for Poisson-disk sampling (Kazimierczuk et al. 2008). Thus, the choice of the gap-size modulation function should achieve a balance between the effects of clustering and the signal’s decay (SNR). Our simulations (Fig. 7) and experiments (Fig. 10) indicate that the choice of $\sin (\frac{\pi }{2} \frac{t}{t_{\text{max}}})$ achieves the necessary balance.

Interestingly, $\sin (\frac{\pi }{2} \frac{t}{t_{\text{max}}})$ and $\sin (\pi \frac{t}{t_{\text{max}}})$ gap-size modulations lead to identical gap-size histograms (green and blue in Fig. 2a). To explain this fact, we need to recall the procedure generating these histograms, in which, roughly speaking, we repeatedly choose the sampling points $0\le t\le t_{\text{max}}$ and then, using the Poisson distribution $\text{Pois}(\lambda )$, generate a new gap, where $\lambda$ is given by $\sin (\frac{\pi }{2} \frac{t}{t_{\text{max}}})$ or $\sin (\pi \frac{t}{t_{\text{max}}})$, depending on the case. Now, the equality of the gap-size histograms for both versions easily follows from the reflection symmetry of $\sin (\pi \frac{t}{t_{\text{max}}})$ with respect to $t= \frac{t_{\text{max}}}{2}$. Thus, the blue-noise artifact patterns of these two schemes are on average very similar, although the lineshape effect in the PSF differs.

It is interesting to consider the extreme cases of the two features of the schedule generator: gap-size distribution (Fig. 2a) and probability density (related to the point distribution in summed schedules, Fig. 2b). Enforcing even (or equal, in the extreme case) gap-size distribution pushes the resulting sampling towards uniform sub-Nyquist sampling. On the other hand, enforcing heavily decaying sampling density pushes it towards truncated uniform sampling. In sinusoidal PG, the density of sampling points is not weighted directly in the NUS generator but results from the gap-size modulation. Yet, as shown below, a resulting heavy bias towards early evolution times is the most important feature of PG, more meaningful than the interpoint distance restriction.

To properly analyze the experimental data, we attempted to separate the effect of blue-noise sampling from the sensitivity gain due to the decaying sampling density. As shown in Fig. 2b), the use of $\sin ^{-1}(\frac{\pi }{2} \frac{t}{t_{\text{max}}})$ density modulation for the weighted random case delivers an almost identical sampling density as the PG with quarter-sine gap-size modulation. There are only slight differences in the sampling of the initial part of the signal due to the lack of distance restriction in weighted random sampling, which is present in PG.

The results shown in Fig. 7 allows for some interesting conclusions. First, although the difference is very minor, unweighted random sampling works best for strictly sparse spectra with high peak dispersion. This is entirely in line with CS theory, which provides a framework for strictly sparse signals without any other specific structure. Second, for the clustered data, all blue-noise schemes work better than the weighted or unweighted random NUS, at least for sufficiently dense sampling schedules. However, as explained in our discussion of the null space property, if the cluster is full of peaks, so to speak, the amplitude modulation has maxima in the initial and final parts of the signal. The $\sin \left( \pi \frac{t}{t_{\text{max}}}\right)$-modulation is particularly well suited to this case. Even in the noiseless case, it provides a better starting-point for the IST with experimental data points in the time domain regions where the signal is strong, and zeros in the regions where it is close to zero. Additionally, the gap-size modulations introduced into blue-noise sampling are particularly useful when the peaks are broad (Lorentzian). As can be seen from Fig. 7h, this effect stems from the decaying sampling density rather than the distance restrictions in a time domain. The weighted random (magenta line) and PG of the same sampling density (quarter-sine, green line) have almost the same residual. For the clustered Lorentzian peaks (Fig. 7e), the PG is clearly better, as in addition to Lorentzian shapes we have clustering.

We suspect that the good performance of decaying sampling schemes for the reconstruction of spectra with Lorentzian peaks is associated with the line-broadening effect visible in Fig. 1c). This effect may compensate for the natural tendency of CS algorithms to artificially narrow or even split the spectral lines, which leads to enhanced sparsity (Stern et al. 2007; Shchukina et al. 2017; Qu et al. 2015).

Finally, the crossing of the red (non-modulated PG) and light blue (unweighted random) lines in Fig. 7g) deserves comment. The width of the low-artifact region in PSF (Fig. 2c) is approximately equal to the average PG sampling density, so for low sampling levels this region may be smaller than the size of a cluster. In fact, it needs to be ca. $2\times$ the width of the cluster in order to make non-modulated PG work better than the unweighted random approach. This only happens above a certain sampling level.

Our simulations employing noiseless signals confirm that the gap-size modulation with $\sin \left( {\pi } \frac{t}{t_{\text{max}}}\right)$ is the best of the tested schemes. However, the decaying signals in noisy experimental data mean that it is better to use a decaying sampling scheme with $\sin \left( \frac{\pi }{2} \frac{t}{t_{\text{max}}}\right)$ gap size modulation. It should be noted that we did not test all available variants of blue-noise sampling (Kazimierczuk et al. 2008; Mobli 2015; Craft et al. 2018), as this would be beyond the scope of this paper.

As can be seen from Fig. 10, the results of the reconstruction from PG sampling are only slightly better than for weighted-random NUS with equivalent density. This can be observed for low sampling levels, where, as shown in Fig. 3, the distribution of sampling points actually differs between the two modes. Moreover, the heavily-clustered ^13^C HSQC (Fig. 10b) gains the most from PG, which confirms our conclusion that PG sampling is effective for clustered spectra. Indeed, in ^15^N HSQC the gain is slightly lower, while in the 3D spectra with weak clustering it is hardly seen at all. The strong decay of the sampling density provides benefits, but this decay can also be achieved without PG. The benefit of PG sampling is that it is less dependent on the seed used (Hyberts et al. 2010; Mobli 2015; Aoto et al. 2014), as reflected in the narrower distribution of residuals (pale-colored bands in Fig. 10). This could be improved by using deterministic schedules (Worley and Powers 2015) or other approaches (Mobli 2015).

It is important to emphasize that the connection between clustering (as defined by Eq. (3)) and the efficiency of PG is a statistical effect. As such, it may be unobserved for certain individual peaks, spectral cross-sections, or sampling schedules generated by particular random generator seeds. This could be due to two factors. First, the clustering metric ($H_j$) introduced above to quantify the peak dispersion is a simplistic parameter and does not take into account the differences in peak intensities and thus in the artifacts generated by these peaks, for example. Second, a random generator can sometimes provide distance-restricted sampling schemes—theoretically, even in the extreme case of uniform sub-Nyquist sampling. In the Supplementary Information we provide residuals for individual peaks from the spectra shown in Fig. 10. As can be seen, some exceptions occur to our rule. Yet, the effect as averaged over the seeds and cross-sections clearly confirms the relation between the clustering level and the efficiency of PG sampling.

Is the gain from the use of PG sampling specific to CS algorithms? Previously, some authors have reported that the improvement is low or absent in spectra processed using iterative hard thresholding (Bostock et al. 2012), SMILE (Ying et al. 2017), or the deep learning method FID-Net (Karunanithy and Hansen 2020). By contrast, the original works on PG employed two different algorithms: forward maximum entropy with the distillation procedure (Hyberts et al. 2010), and iterative soft thresholding (Hyberts et al. 2012a). Both of these algorithms perform an $\ell_1$-norm minimization, and thus their principles are very well described by standard CS theory. Other blue-noise sampling schemes have been found to work well with maximum entropy (Mobli 2015; Craft et al. 2018).

In some cases we are particularly interested in individual peaks that are far from the cluster and can be heavily covered by artifacts. For example, for the far off-diagonal regions in the NOESY spectrum, Bostock et al. (see Fig. 8 in Bostock et al. 2012) report strong artifacts in the case of the PG schedule. With this in mind, Mobli and Miljenović proposed dedicated “red-noise” burst schemes for NOESY and TOCSY spectra (Mobli and Miljenović 2019). Thus, although it seems that PG may be safely used as a default sampling for protein backbone assignment experiments, in many contexts it may be sub-optimal. It has been pointed out that other blue-noise schemes provide more freedom for sensitivity optimization by shaping the distribution of points (Kazimierczuk et al. 2008; Zambrello et al. 2020; Roginkin et al. 2020). Nevertheless, we believe that the relationship between spectral clustering and blue-noise is not limited to PG.

It should be noted that the analysis of the clustering in the BMRB data (Fig. 9) and in the experiments (Fig. 10) underestimates its true level. In practice, the experimental spectral width is not exactly tailored to the extreme peak positions, but set with a certain margin. Thus, the actual clustering is actually slightly stronger than Figs. 9 and 10 suggest.

## Conclusions

To summarize, blue-noise sampling schemes may be the optimal choice for many protein backbone experiments. Apart from spectral sparsity, which to a certain extent underlies all NUS methods, they also exploit the clustering found in spectra. Clustering is common in protein NMR, but its level differs between different kinds of experiments. For example, ^13^C HSQC spectra of proteins are usually heavily clustered and thus blue-noise sampling is by far the best way of generating NUS schedules for them. In other cases, its advantages may be less pronounced.

It should be noted that peaks far from the cluster may be reconstructed less well when using PG. If it is precisely these peaks that are of interest, other sampling schemes should be considered (Mobli and Miljenović 2019).

As shown by many of the authors cited in this study, no single optimal NUS schedule exists for NMR. The choice of schedule will depend on a multitude of conditions: sensitivity, relaxation, dimensionality, line shapes, dynamic range of peak intensities, type of experiment, type of reconstruction algorithm, and the information one wishes to gain from the data. Our study suggests adding the presence of peak clusters to this list: Where peak clusters are present, it is worth considering blue-noise schedules, at least if certain algorithms will be used for the reconstruction.

We hope that the current paper shines some light on the problem of sampling choice and provides a more rational basis for the use of blue-noise NUS. We have shown that peak clusters may be expected in many protein backbone spectra; perhaps others will find them in data that is beyond the focus of this study. Although the observation might seem obvious to many spectroscopists, we would like to emphasize how meaningful it is in the context of non-uniform sampling.

## Supplementary Information

The Supplementary Information contains the reconstruction residuals for each individual peak (marked with *) in four experimental datasets: ^13^C HSQC and ^15^N HSQC of ubiquitin, and 3D HNCA and 3D HNCACB of azurin.

Below is the link to the electronic supplementary material.

Supplementary file1 (PDF 3695 kb)

Supplementary file2 (PDF 14484 kb)

Supplementary file3 (PDF 20244 kb)

Supplementary file4 (PDF 29610 kb)

## Data Availability

The datasets generated during and analysed during the current study are available from the corresponding author on reasonable request.
