# Supplementary material for: Clustered sparsity and Poisson-gap sampling
Source: J Biomol NMR. 2021 Nov 5;75(10-12):401–16. doi: 10.1007/s10858-021-00385-7 (PMC8642362; doi:10.1007/s10858-021-00385-7)

Supplementary Information:

Clustered sparsity and Poisson-gap sampling

Paweł Kasprzak[1,2], Mateusz Urbańczyk[1,3], and Krzysztof Kazimierczuk[1]

[1]Centre of New Technologies, University of Warsaw, Banacha 2C, 02-097 Warsaw, Poland
[2]Faculty of Physics, University of Warsaw, Pasteura 5, 02-093 Warsaw, Poland
[3]Institute of Physical Chemistry, Polish Academy of Sciences, Kasprzaka 44/52,01-224 Warsaw, Poland

Analysis of ^{15}N-HSQC spectrum of ubiquitin

Q2N-H

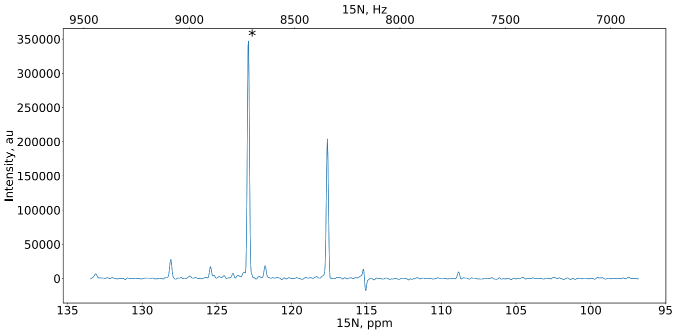

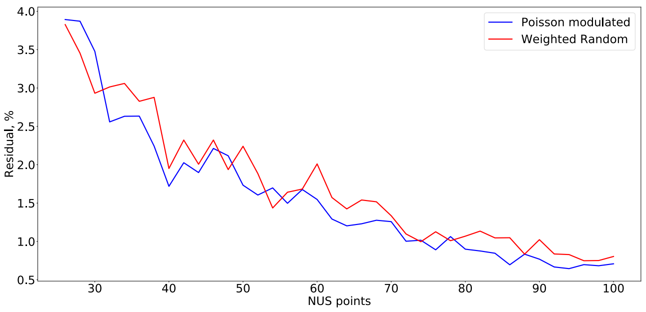

I3N-H

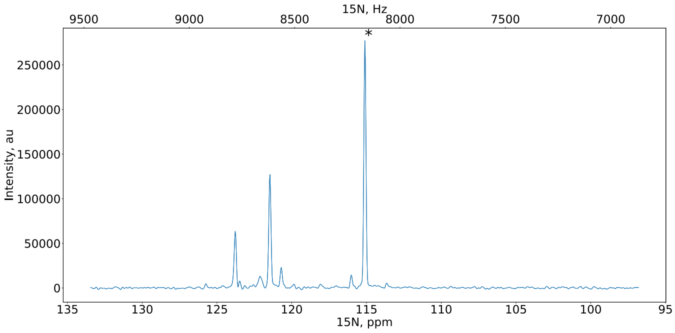

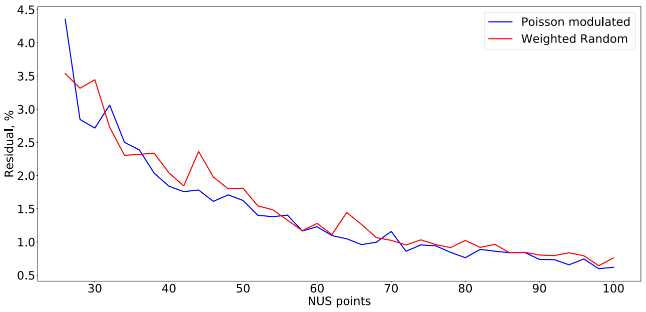

F4N-H

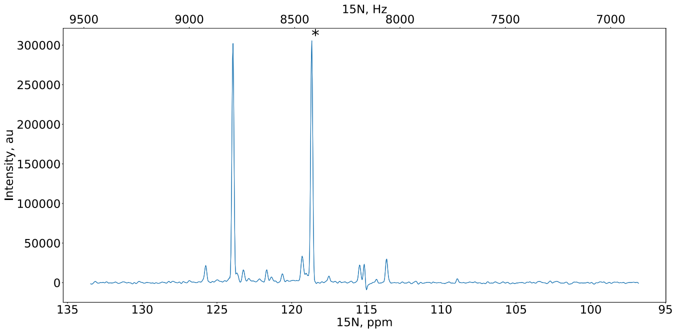

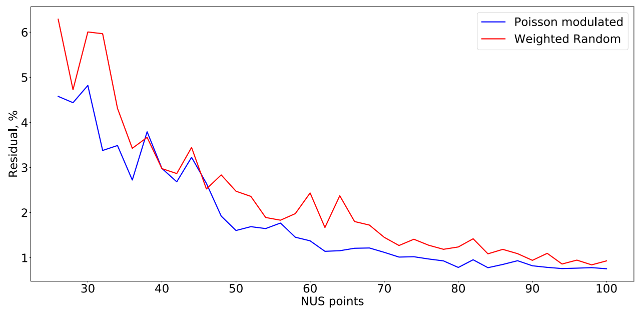

V5N-H

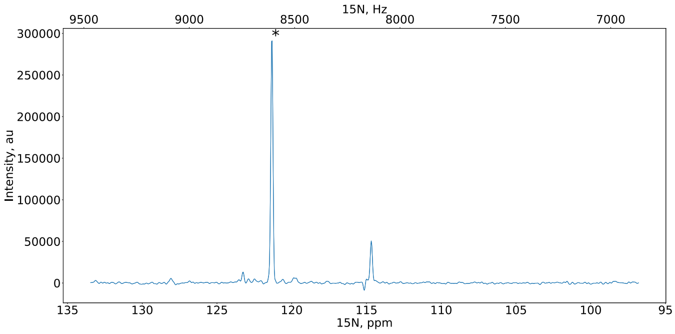

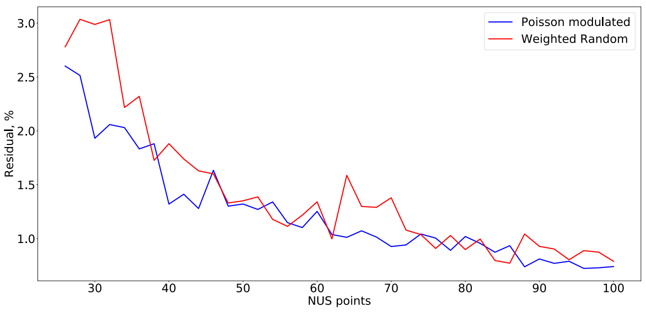

K6N-H

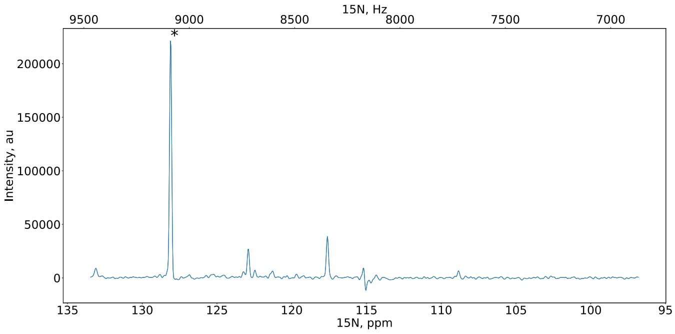

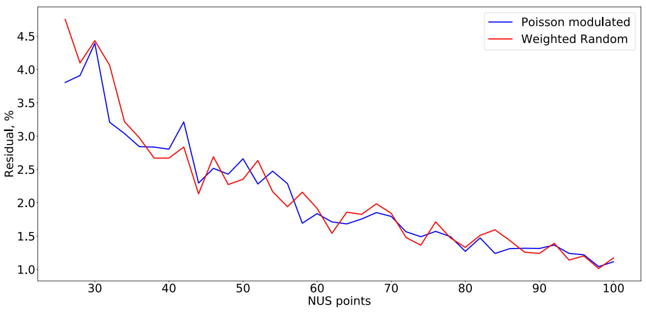

T7N-H

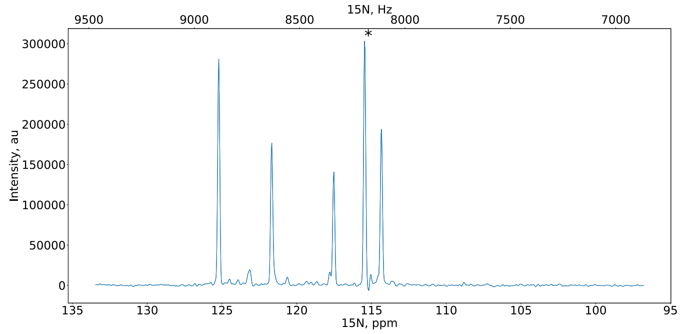

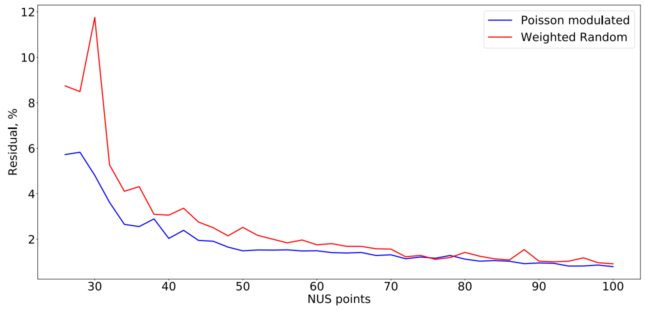

L8N-H

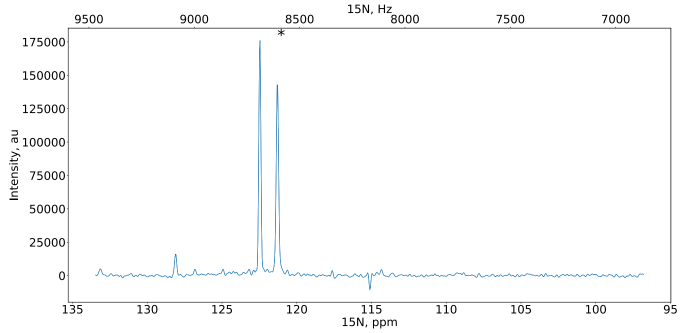

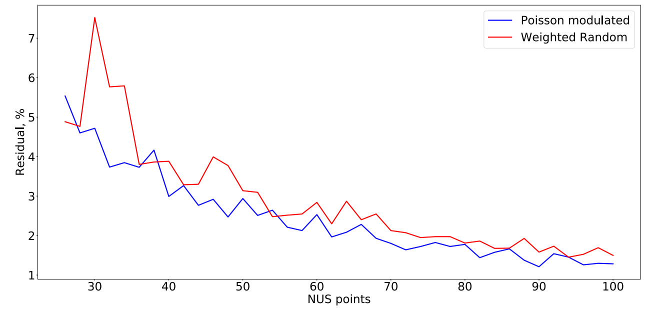

T9N-H

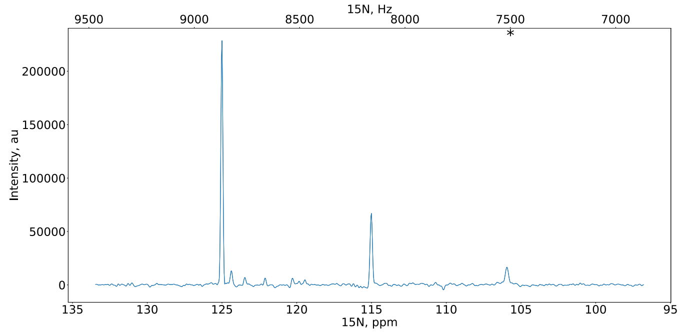

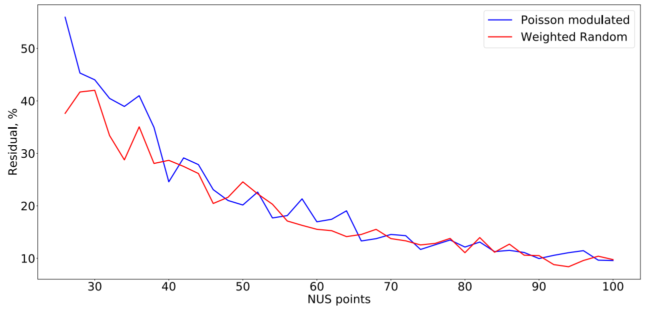

G10N-H

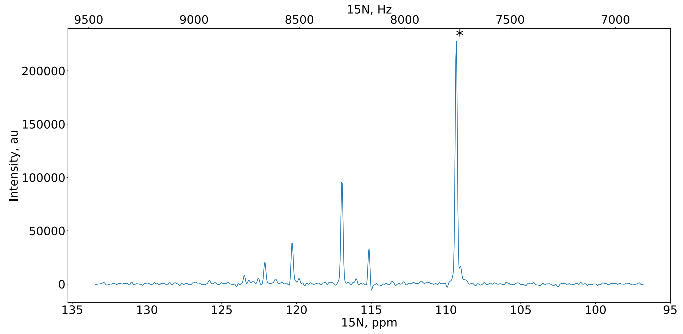

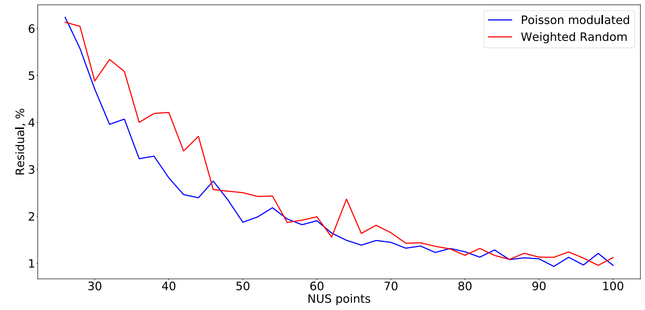

K11N-H

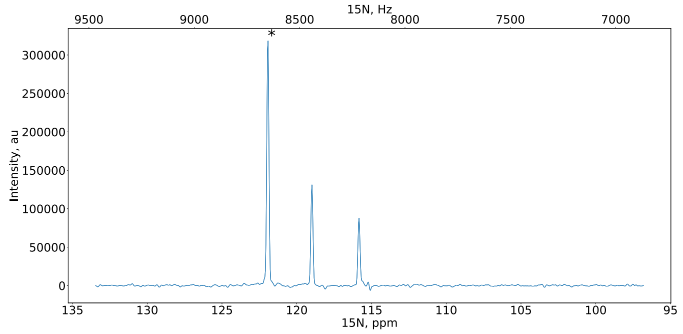

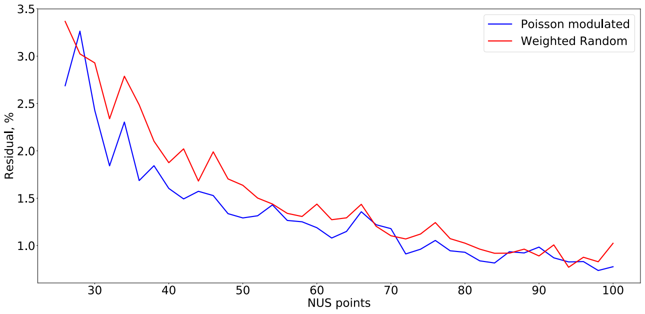

T12N-H

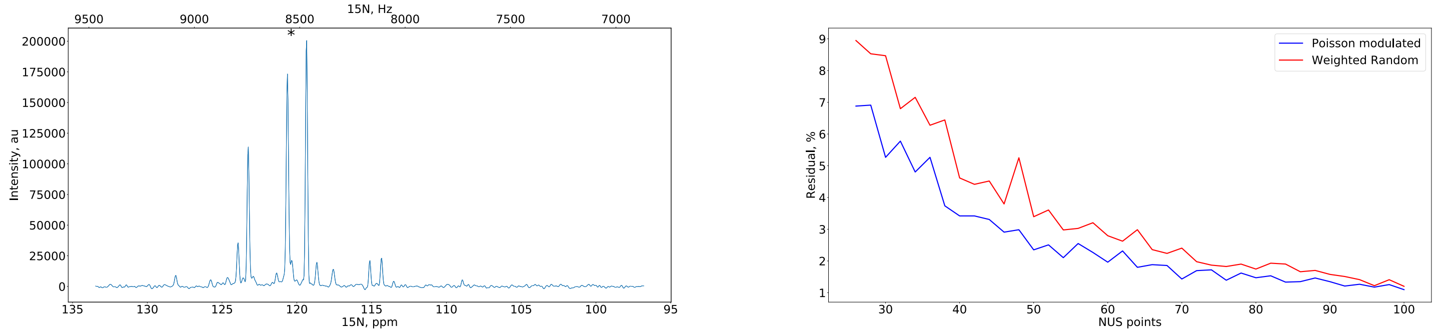

I13N-H

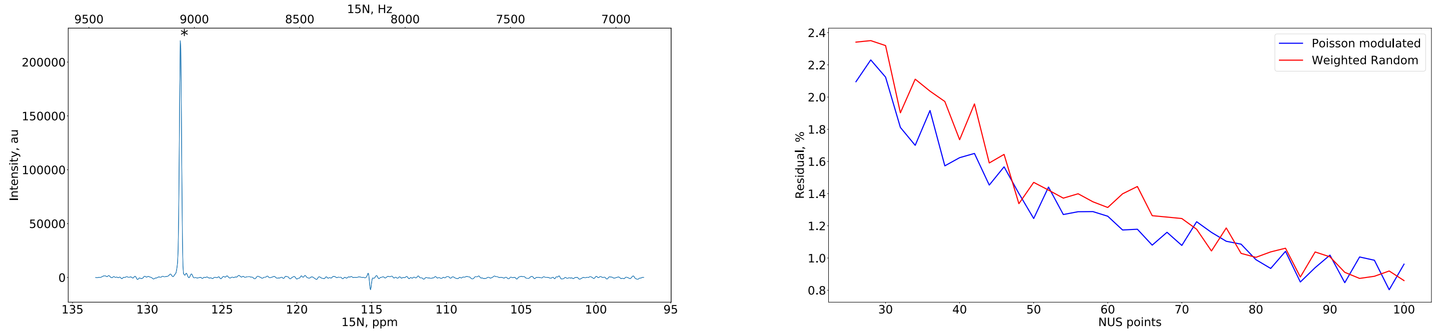

T14N-H

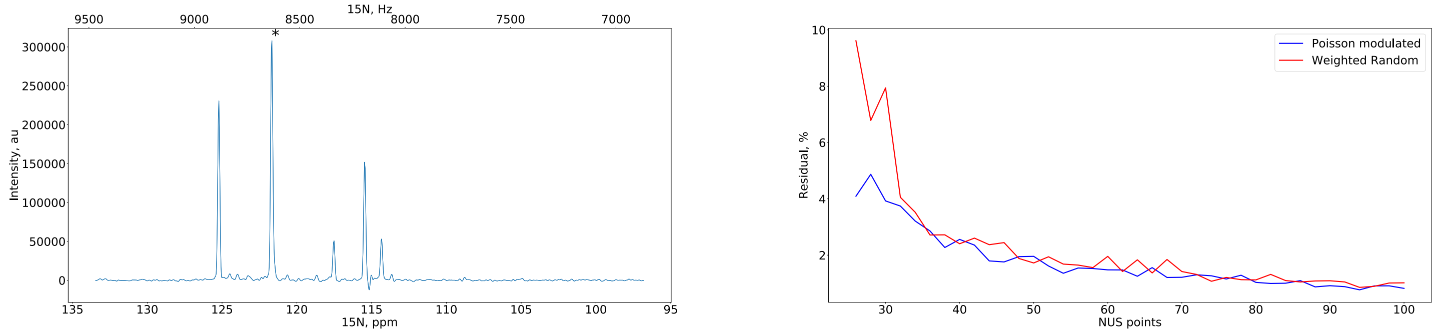

L15N-H

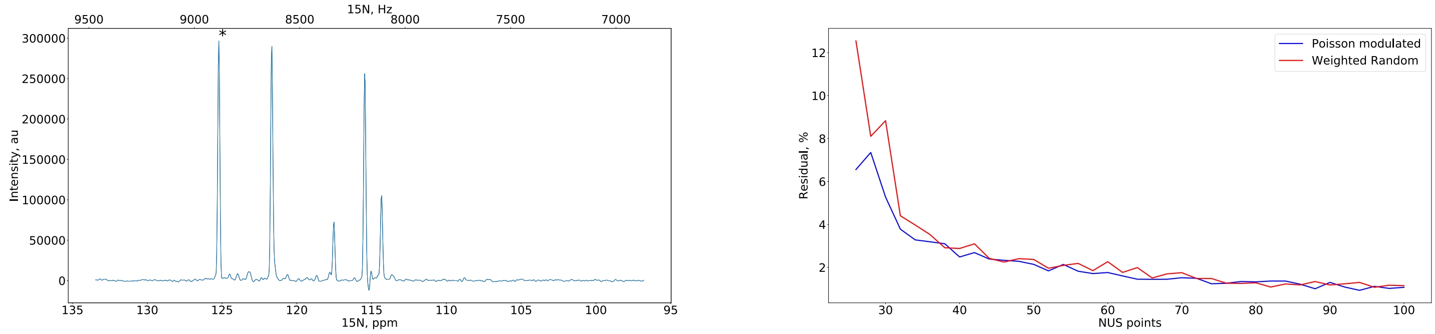

E16N-H

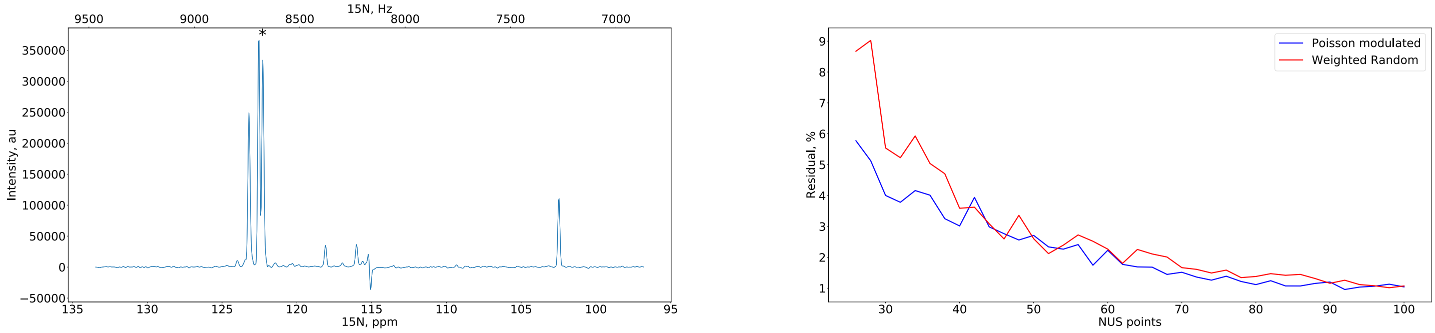

V17N-H

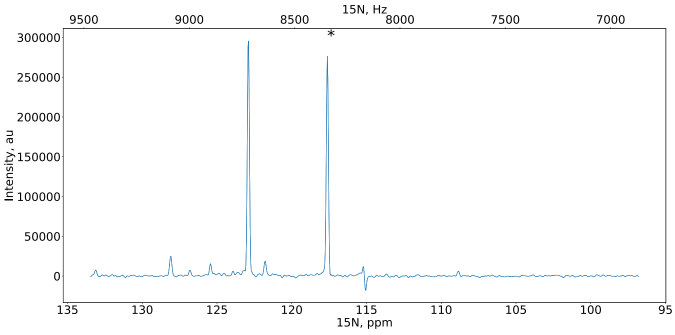

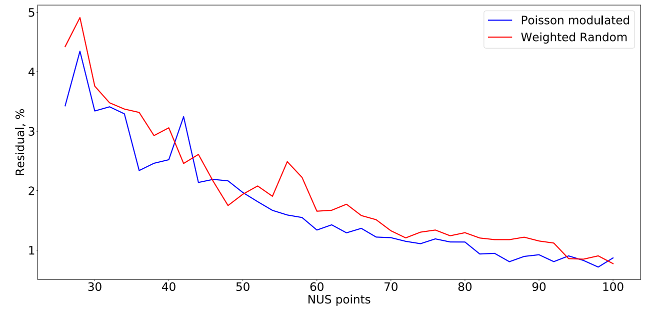

E18N-H

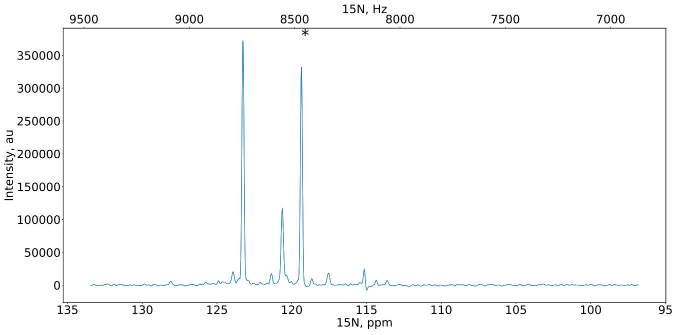

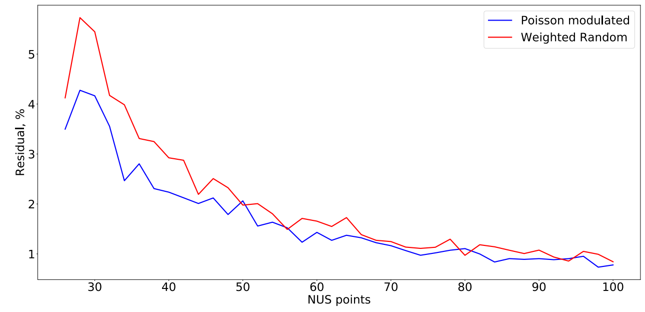

S20N-H

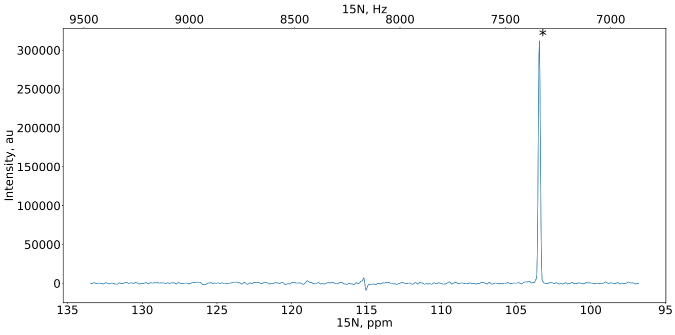

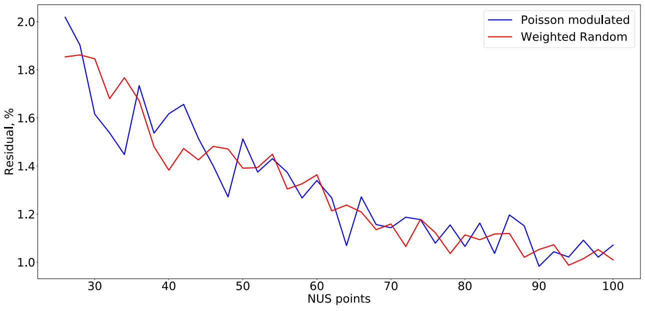

D21N-H

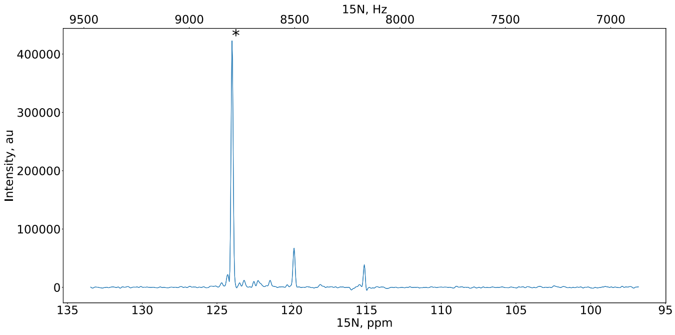

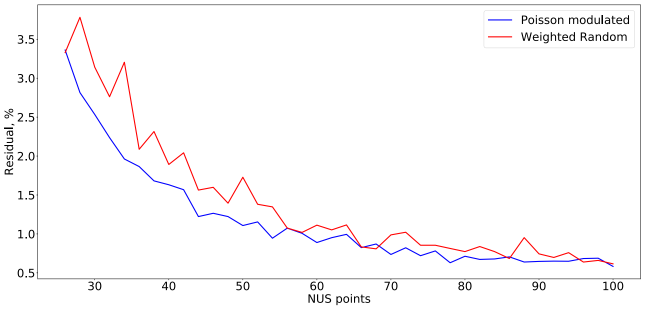

T22N-H

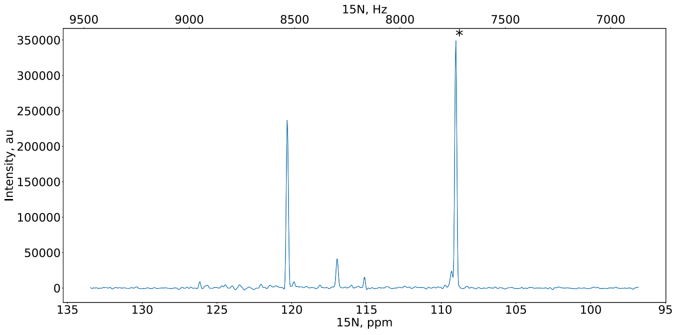

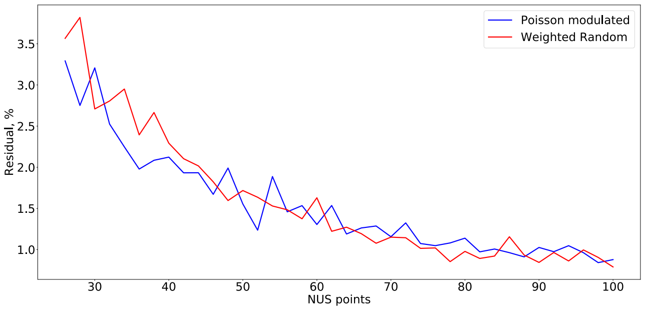

I23N-H

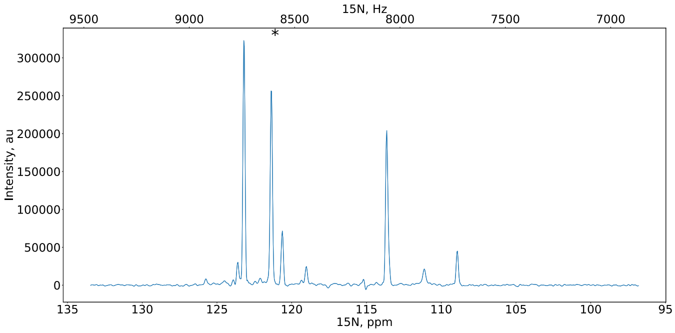

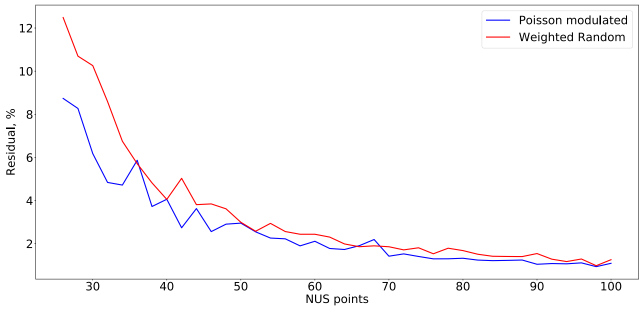

N25N-H

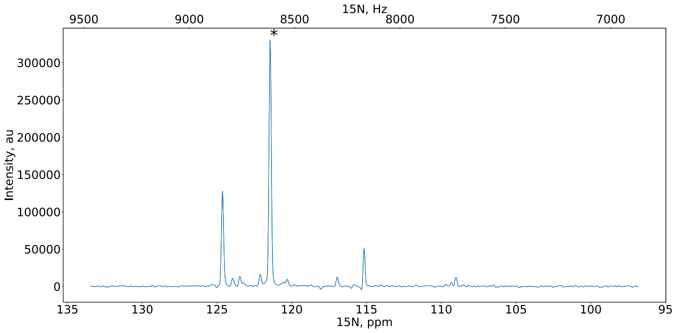

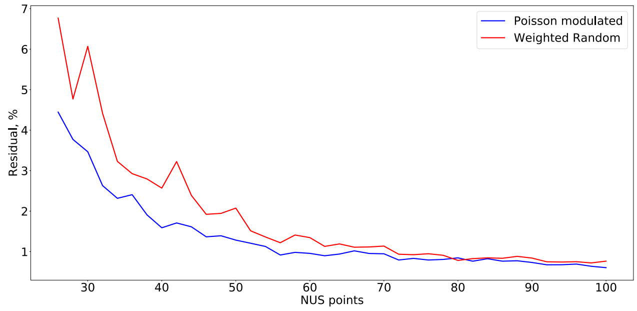

V26N-H

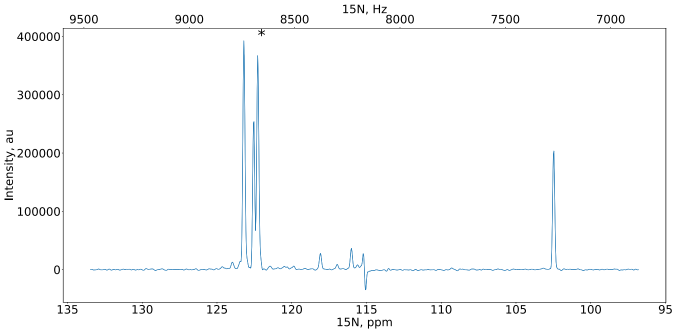

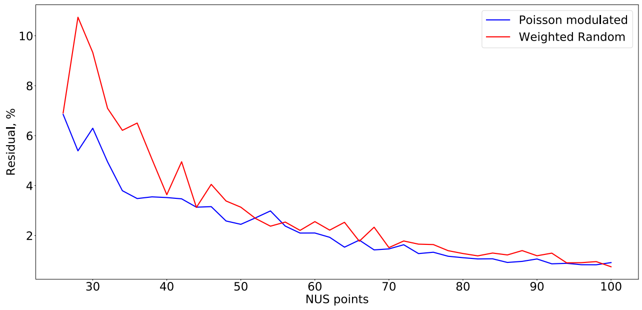

K27N-H

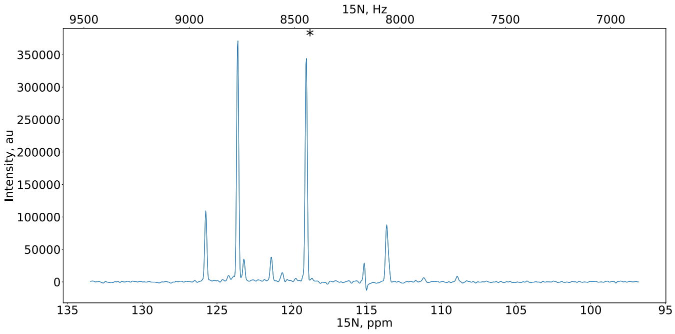

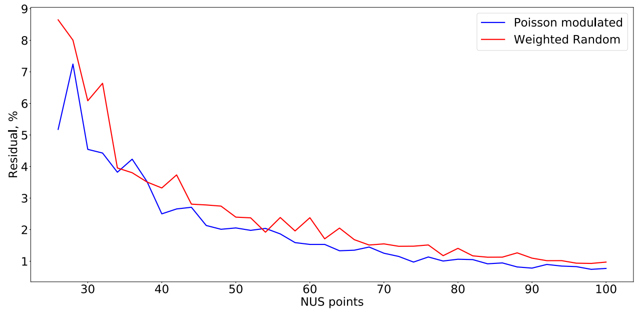

A28N-H

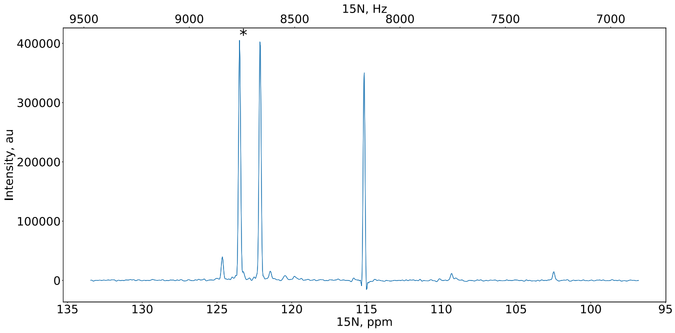

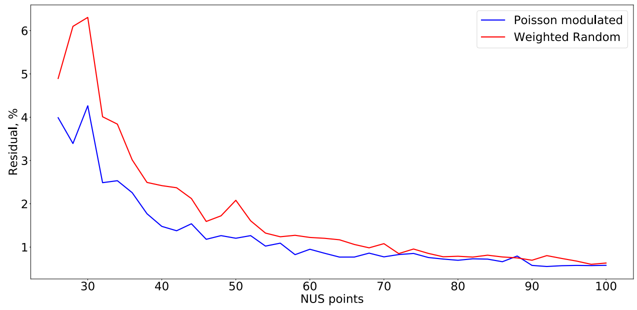

K29N-H

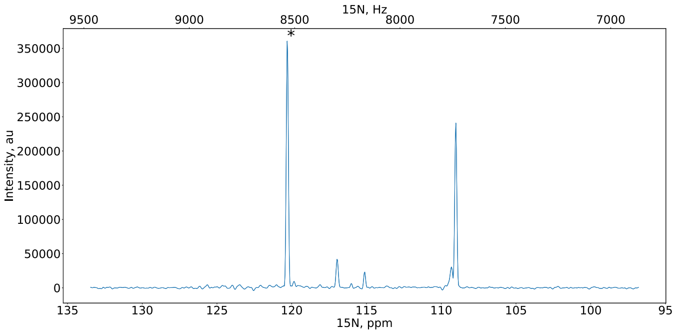

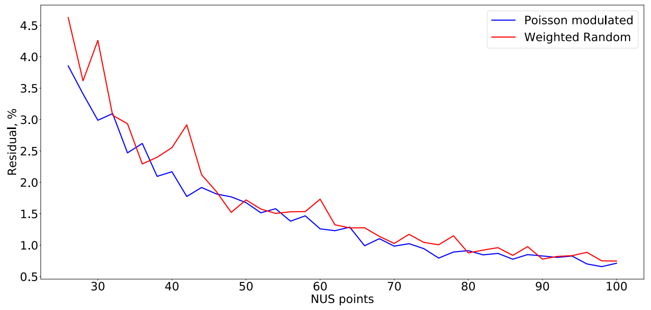

I30N-H

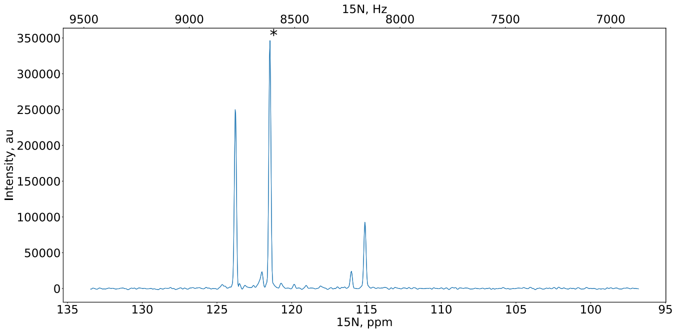

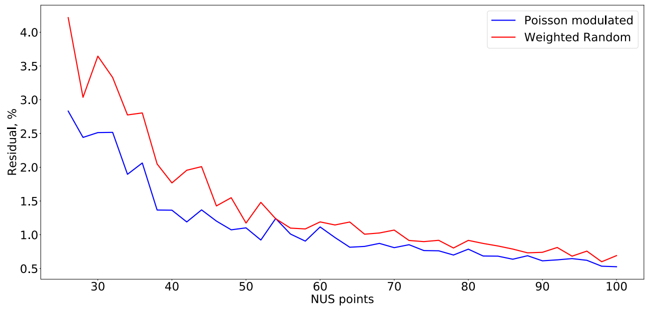

Q31N-H

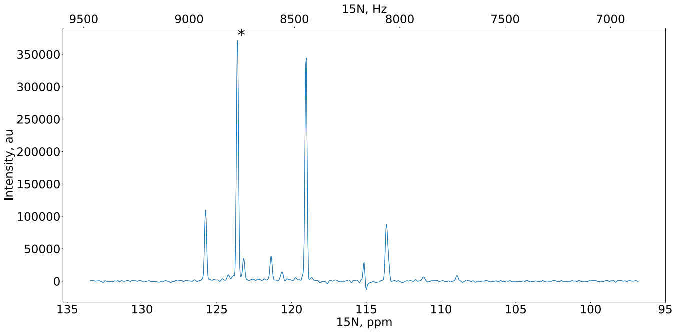

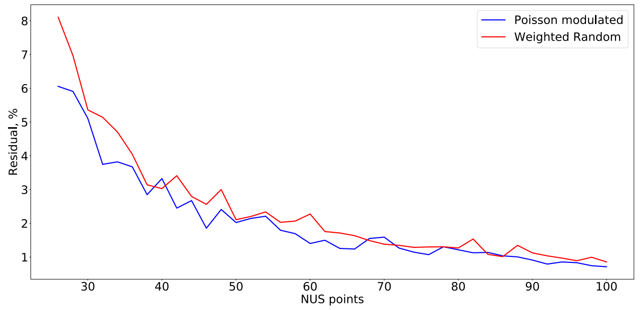

D32N-H

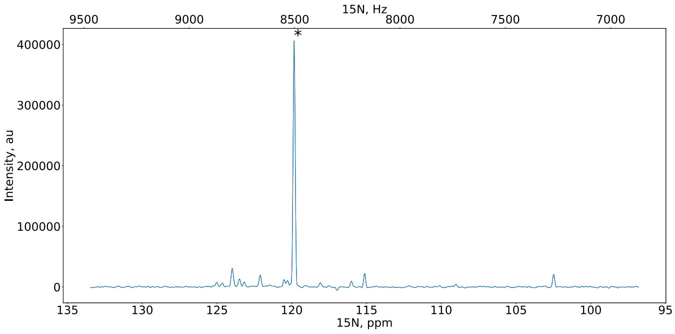

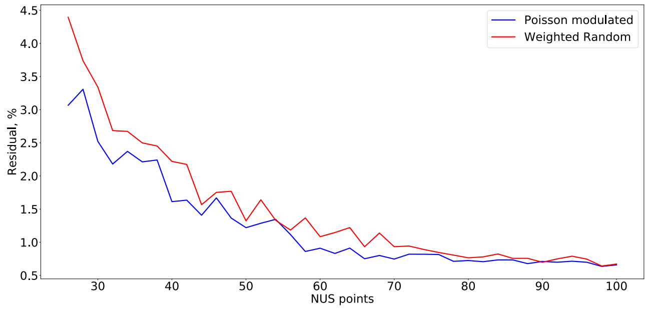

K33N-H

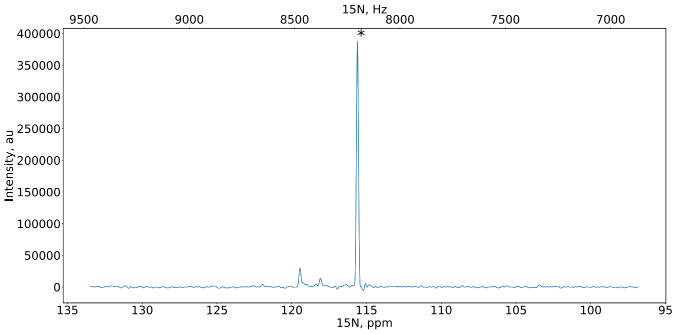

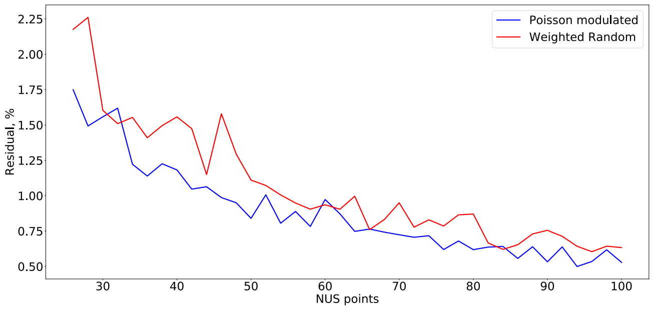

E34N-H

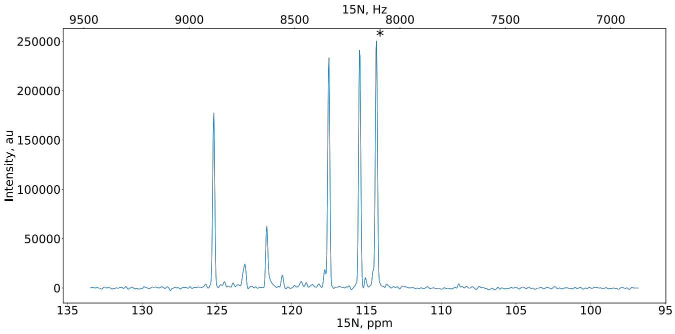

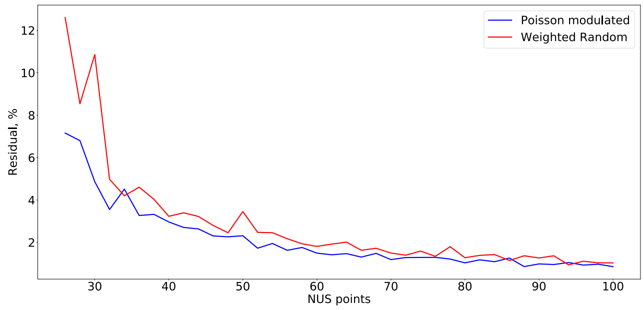

G35N-H

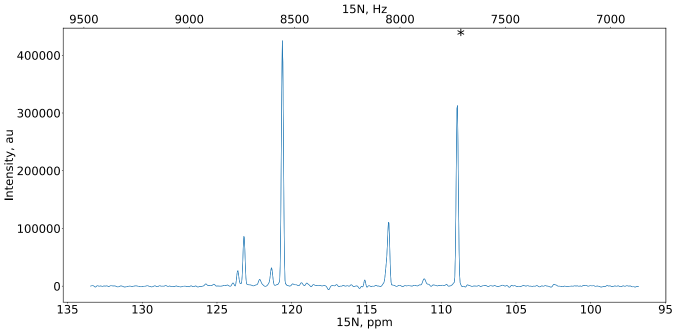

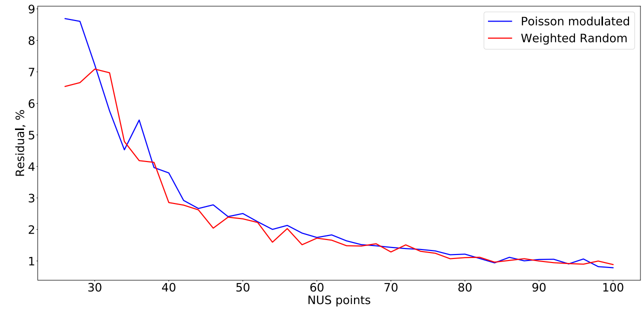

I36N-H

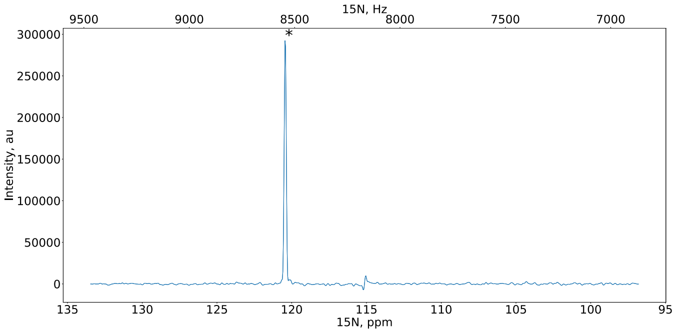

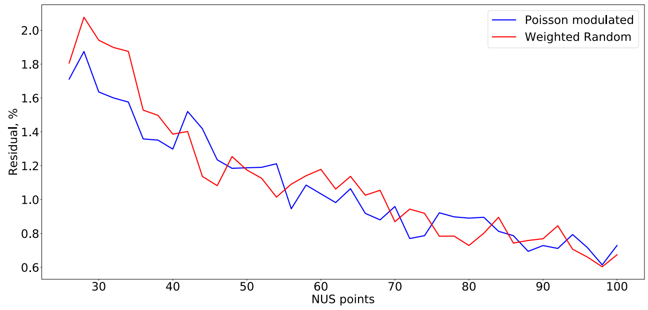

D39N-H

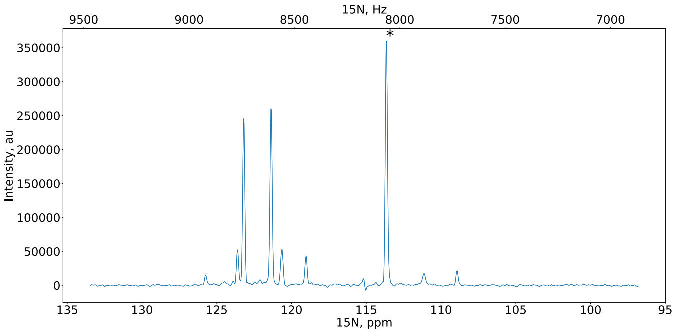

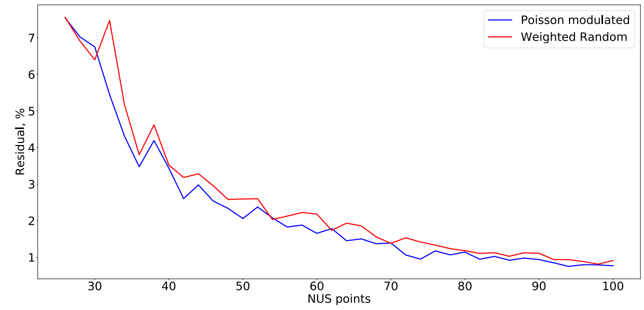

Q40N-H

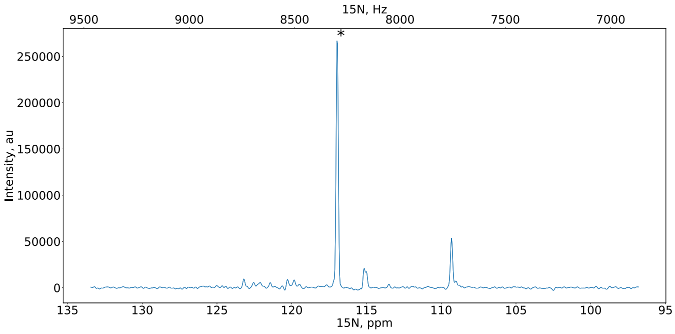

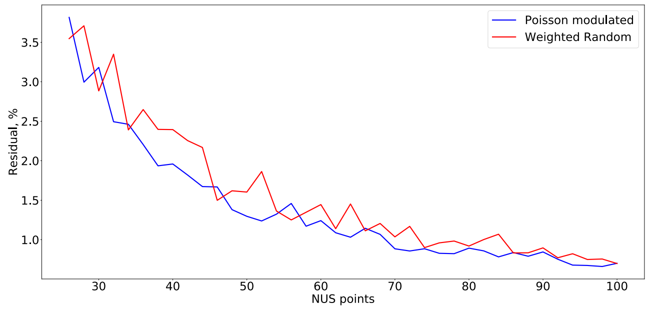

Q41N-H

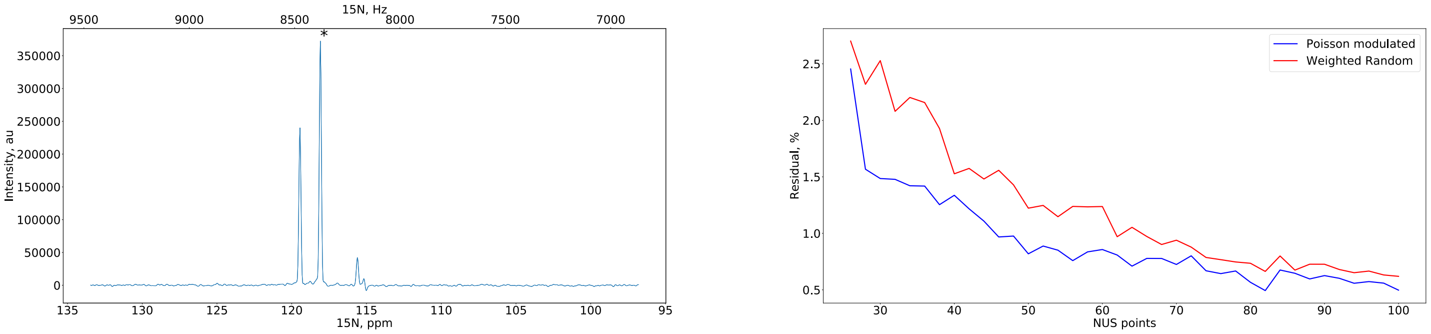

R42N-H

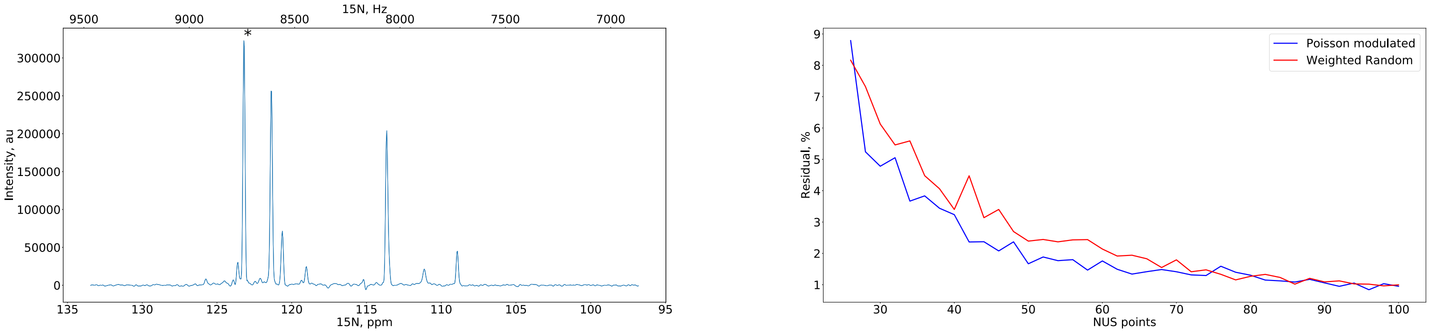

L43N-H

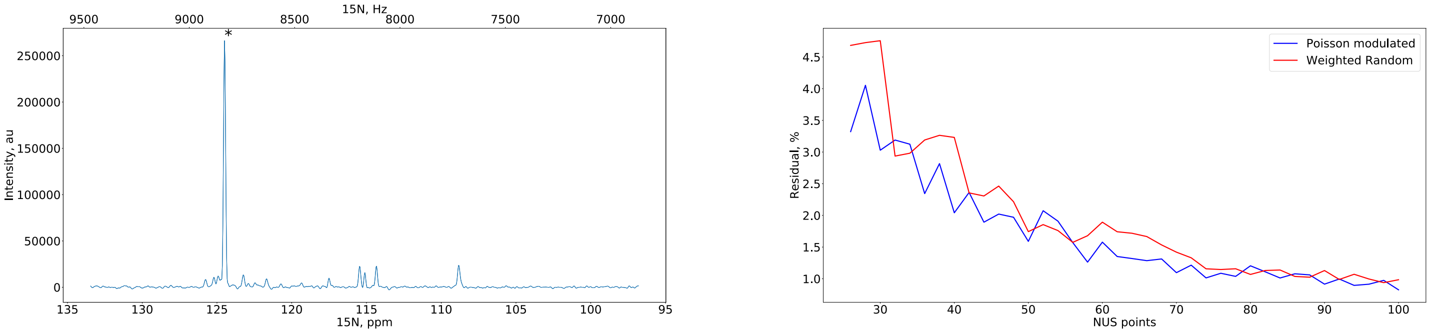

I44N-H

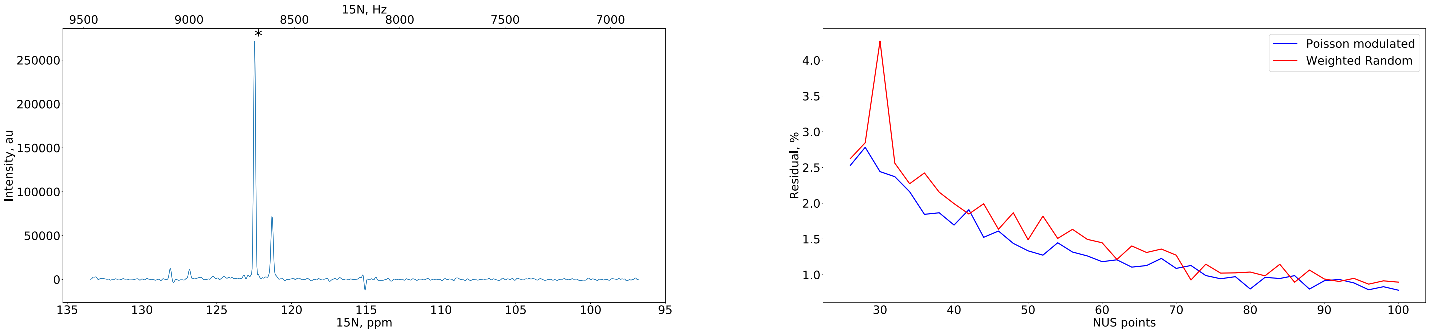

F45N-H

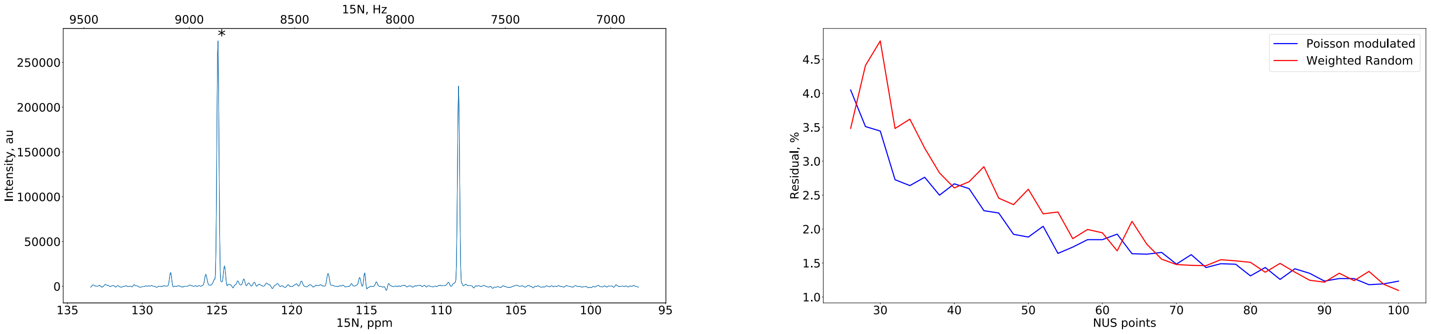

G47N-H

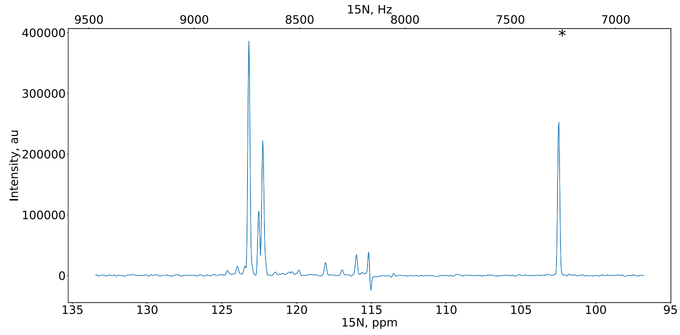

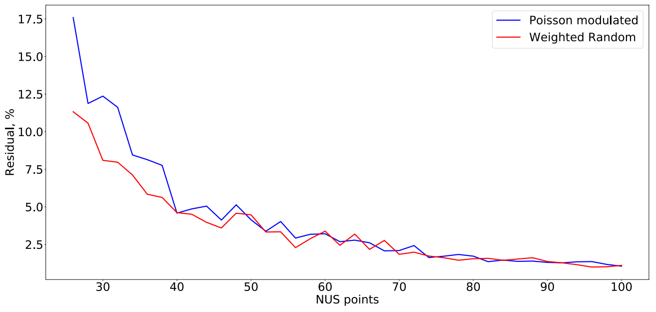

K48N-H

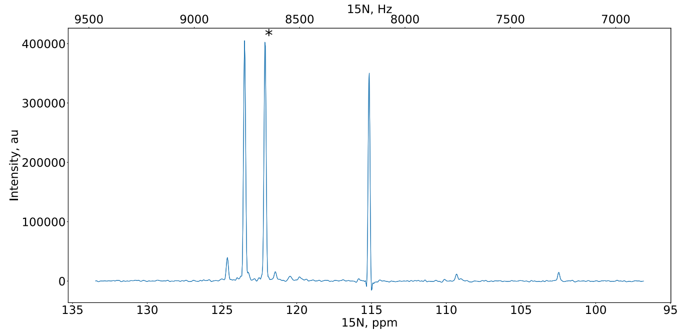

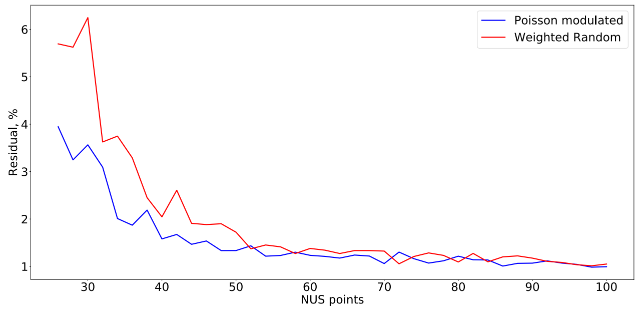

Q49N-H

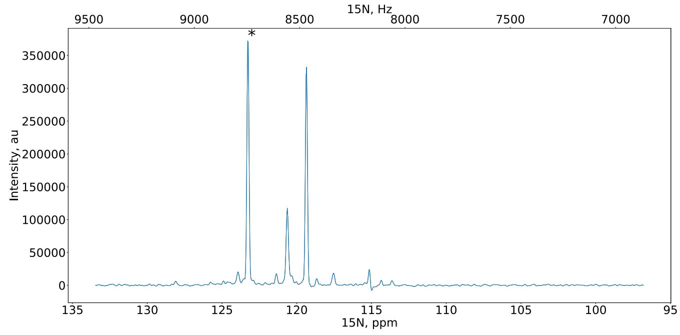

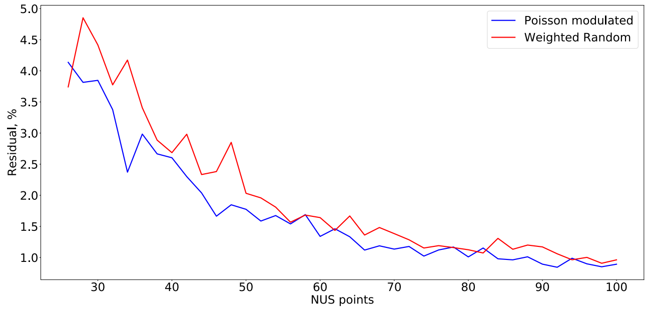

L50N-H

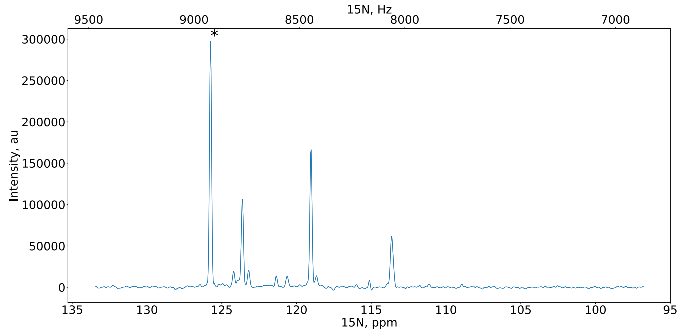

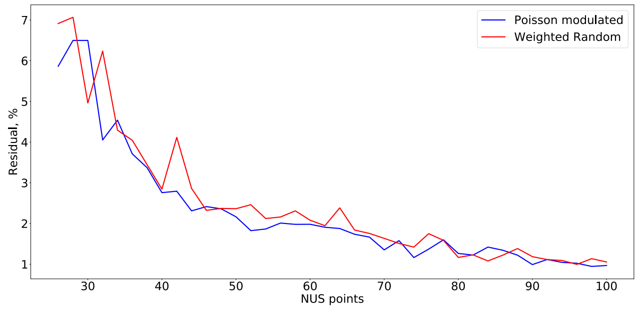

E51N-H

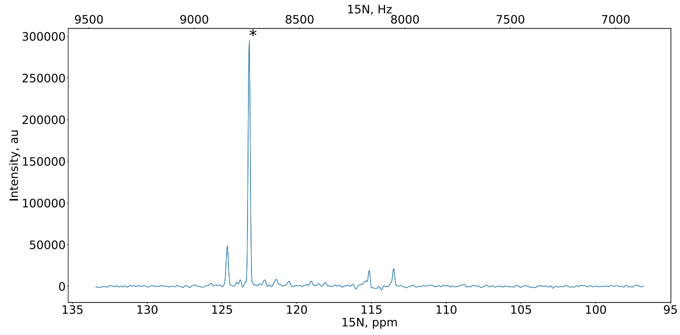

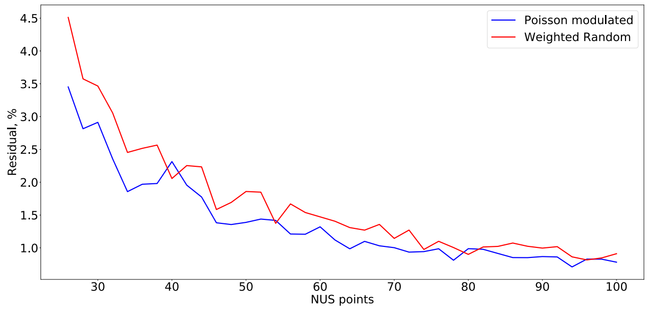

D52N-H

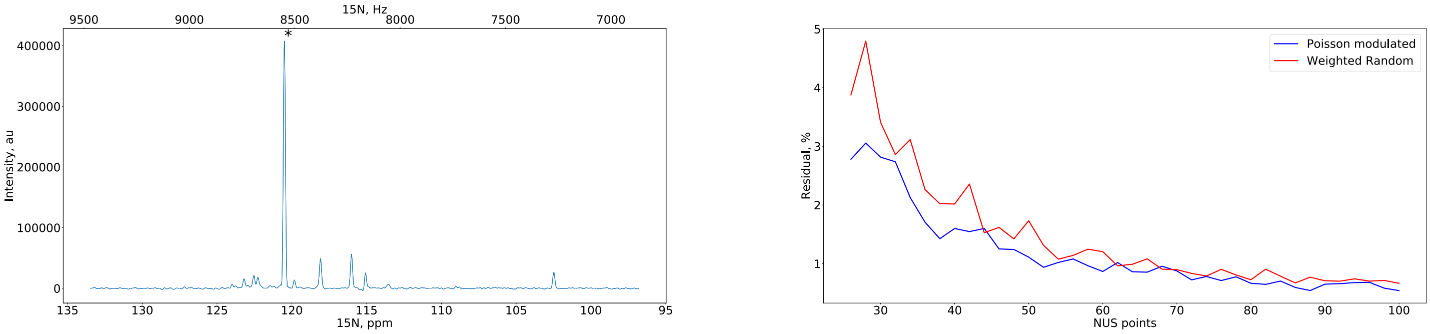

R54N-H

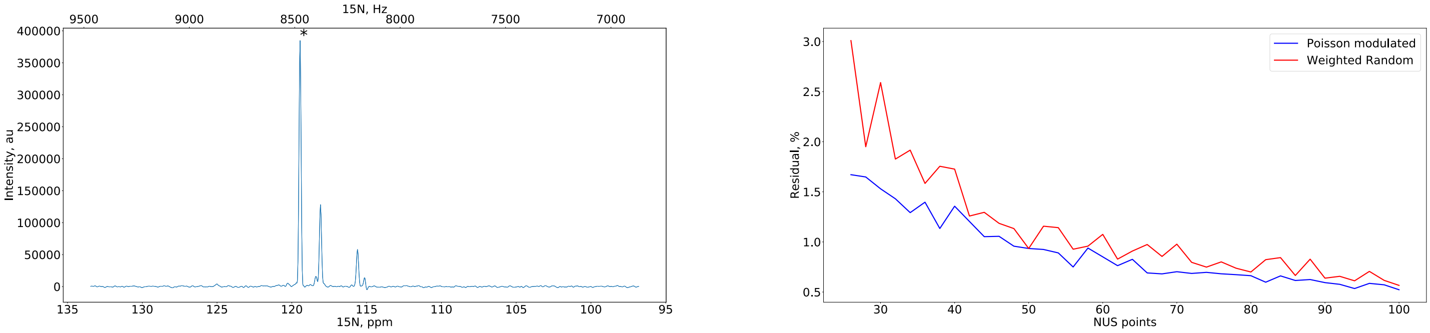

T55N-H

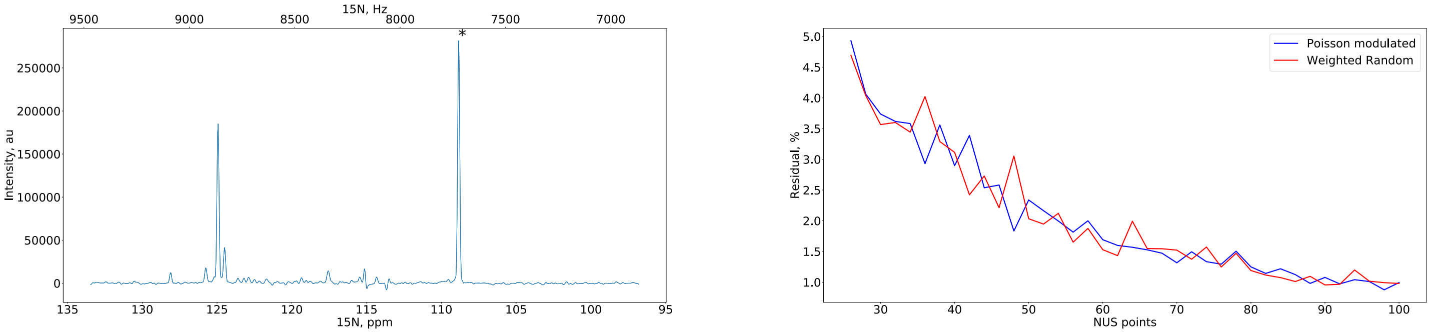

L56N-H

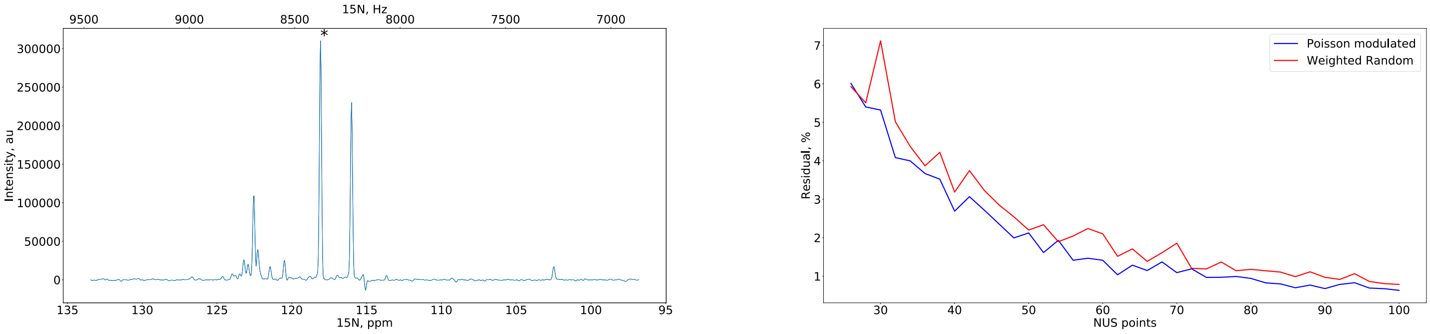

S57N-H

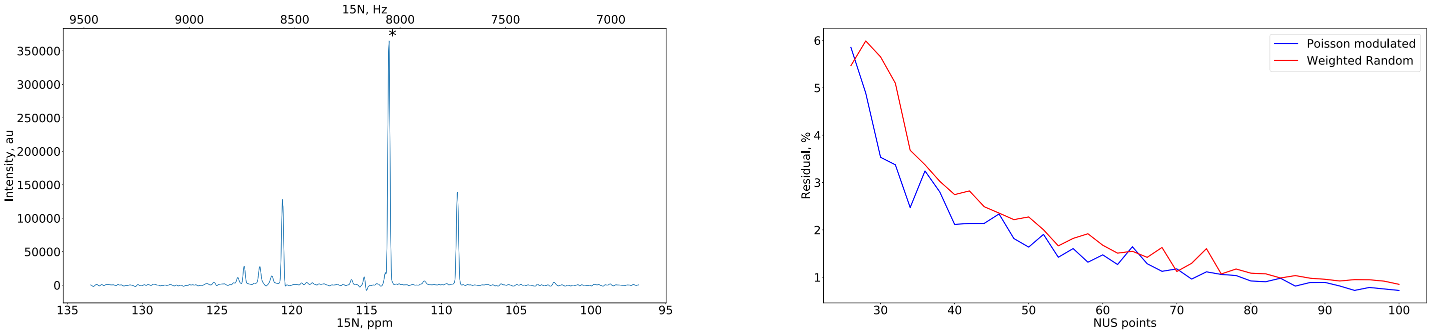

D58N-H

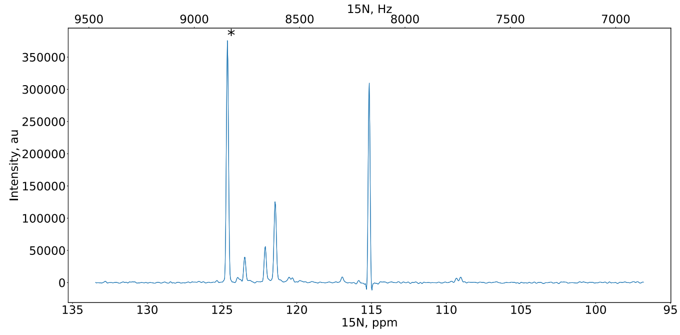

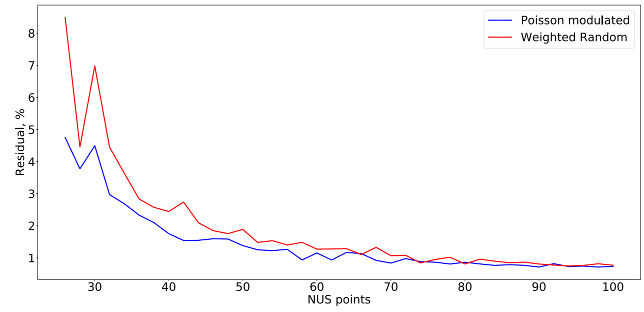

Y59N-H

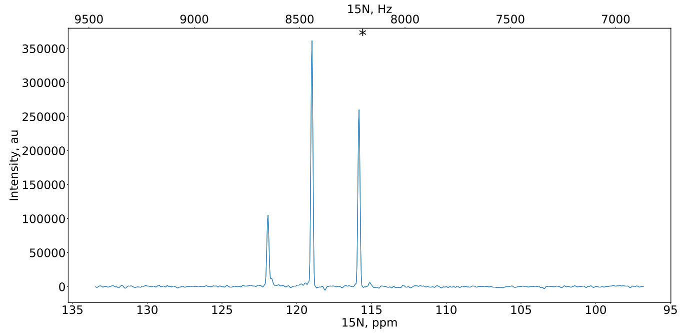

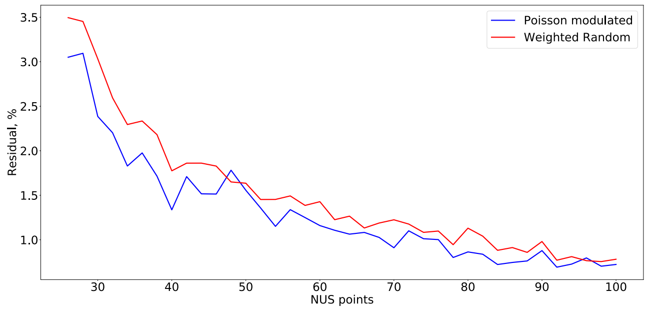

N60N-H

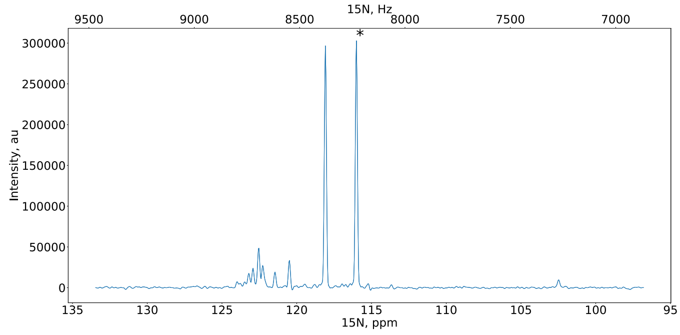

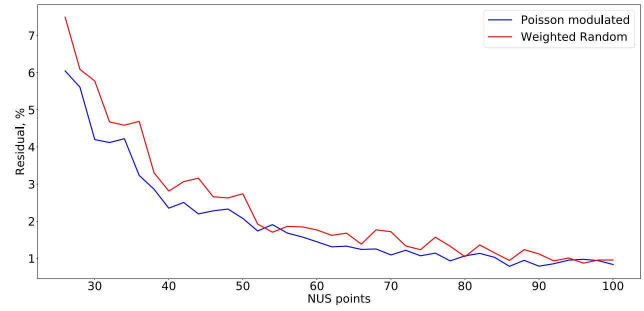

I61N-H

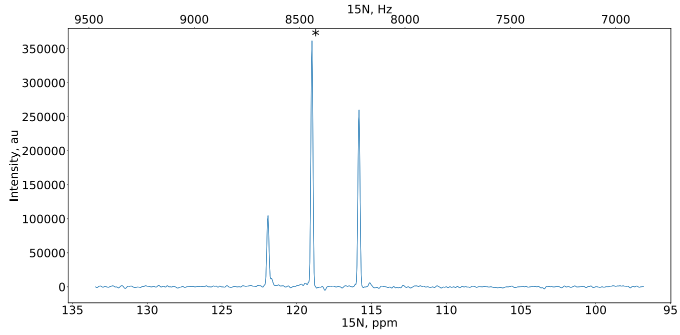

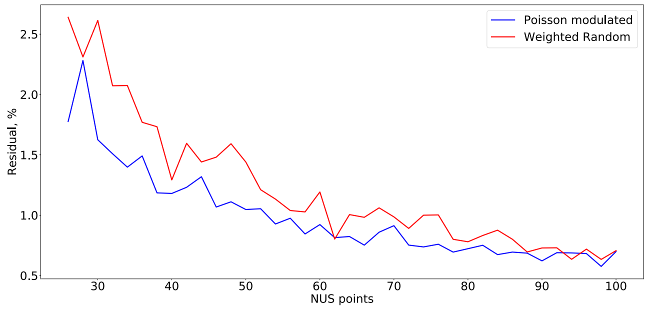

Q62N-H

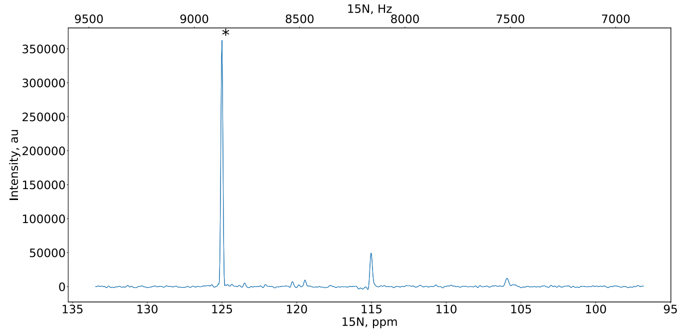

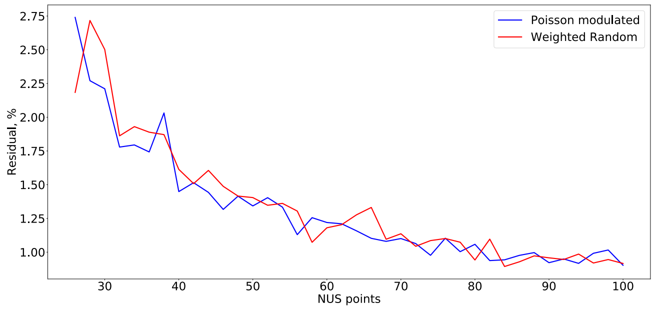

K63N-H

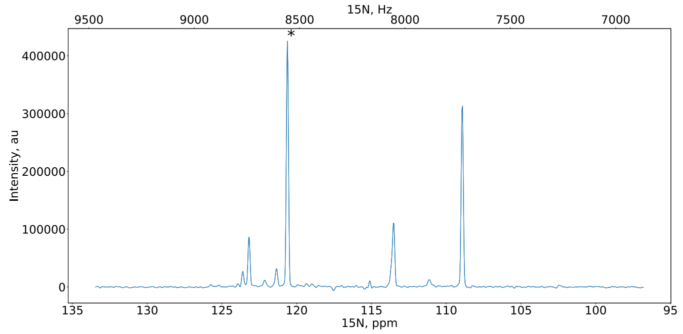

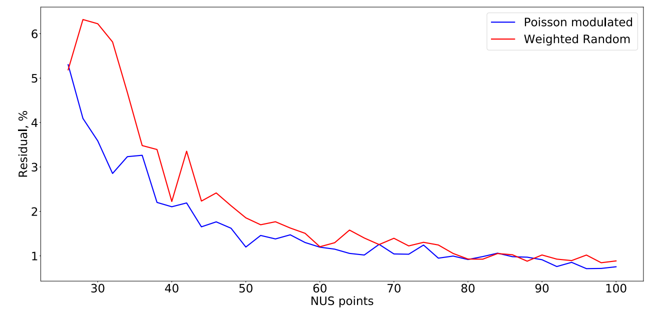

E64N-H

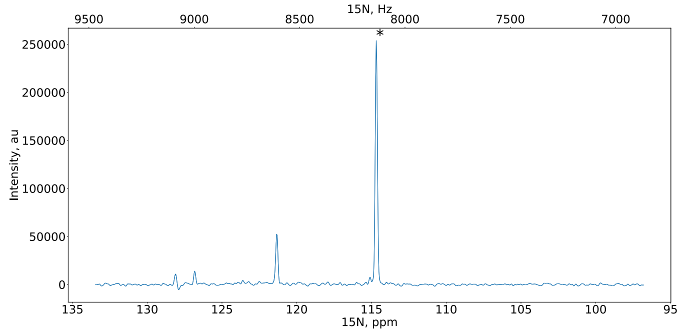

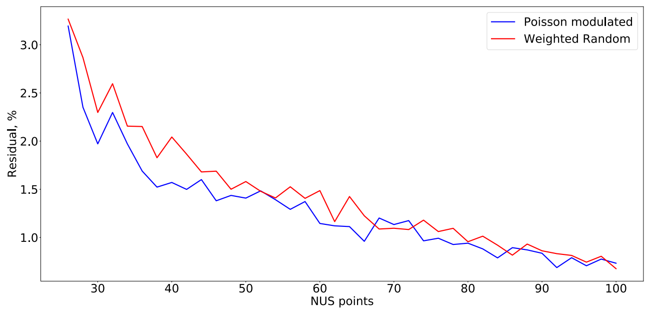

S65N-H

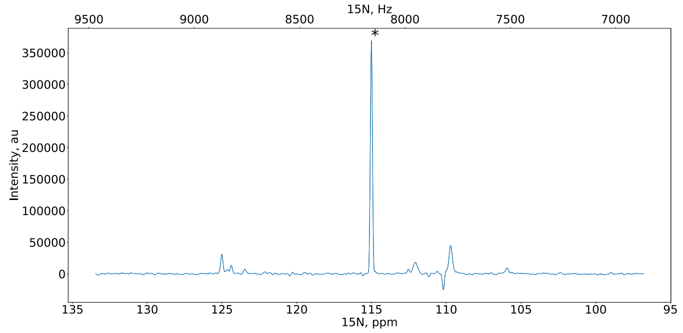

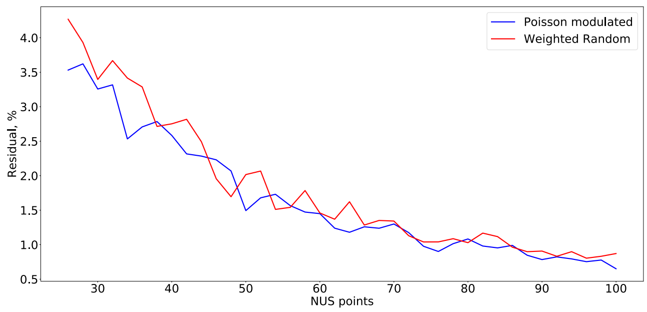

T66N-H

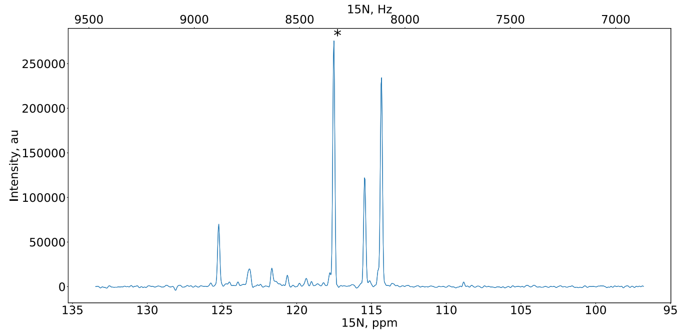

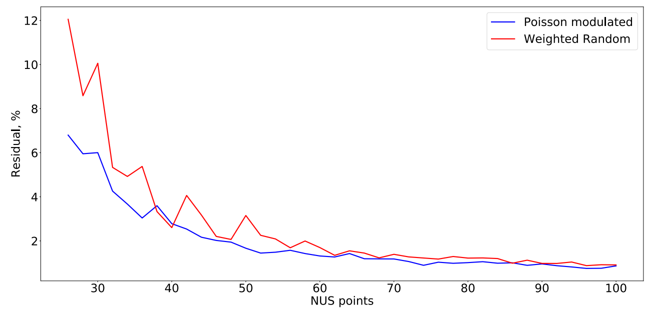

L67N-H

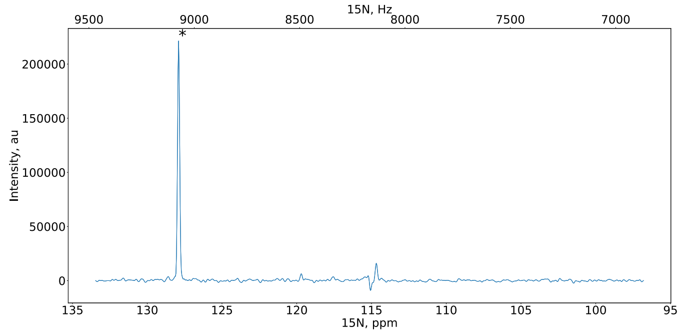

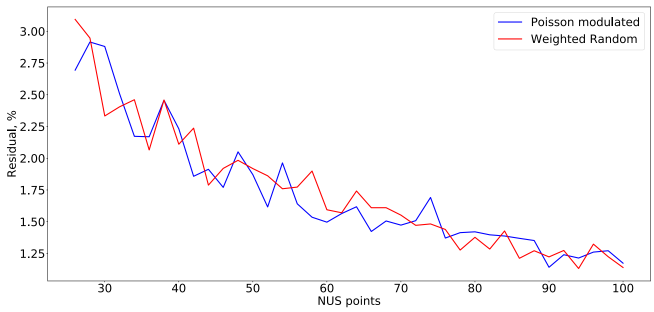

H68N-H

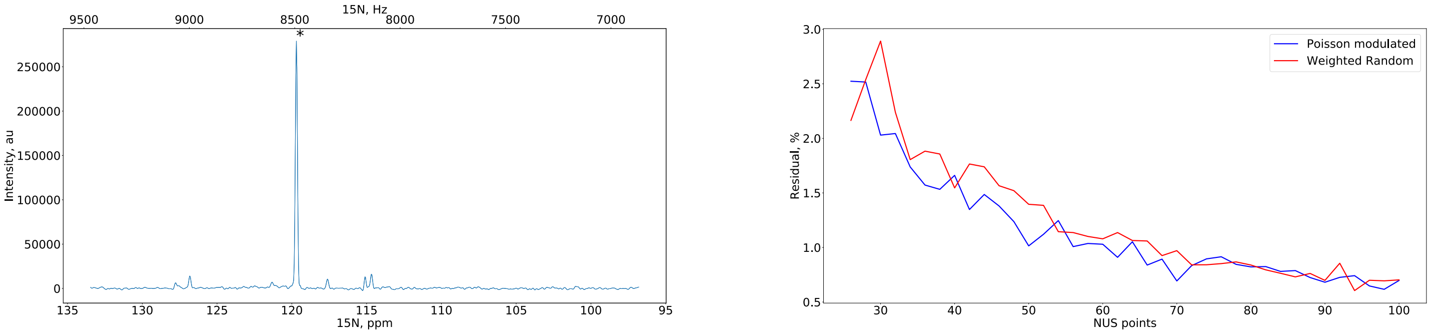

L69N-H

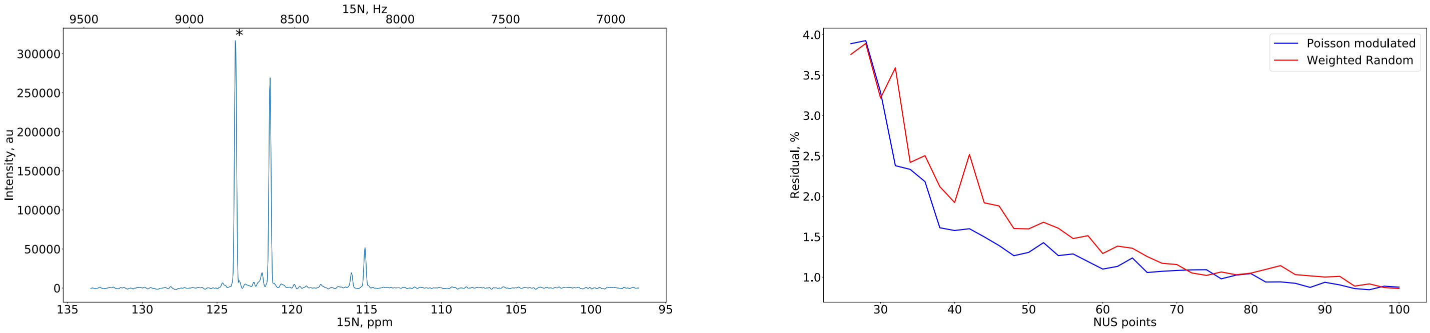

V70N-H

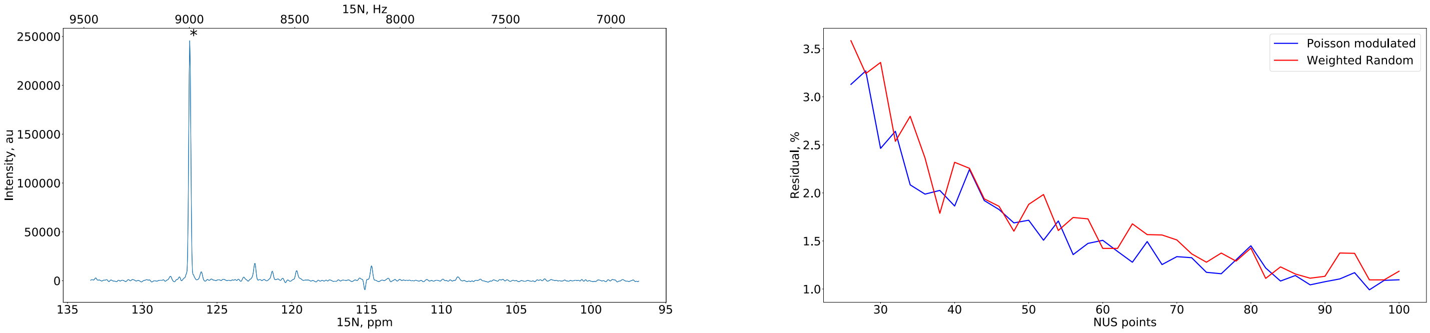

L71N-H

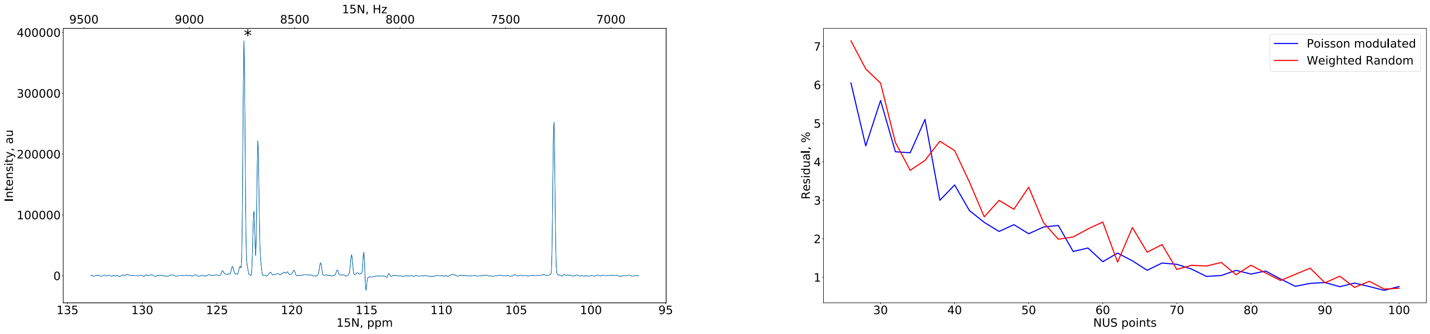

R72N-H

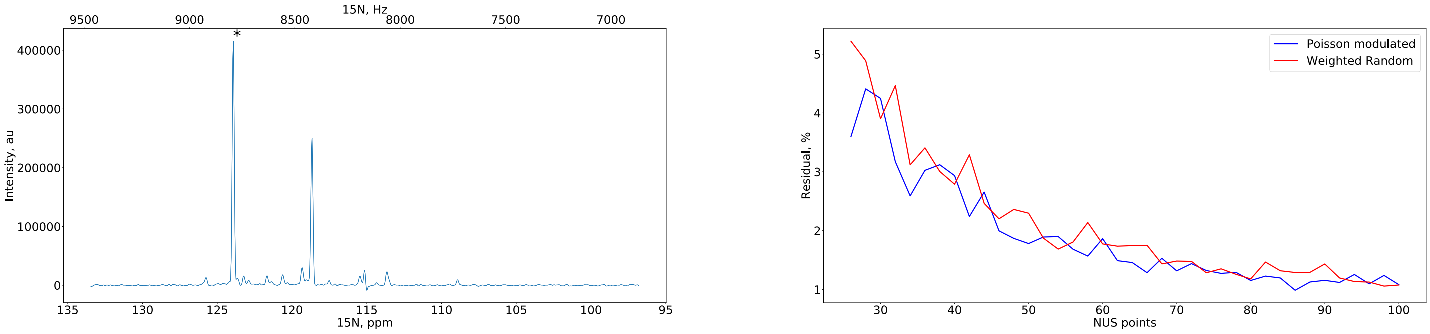

L73N-H

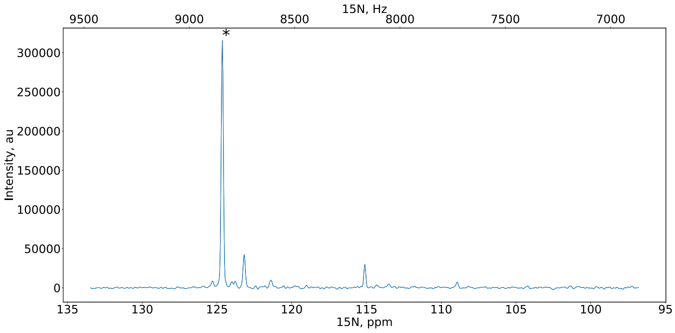

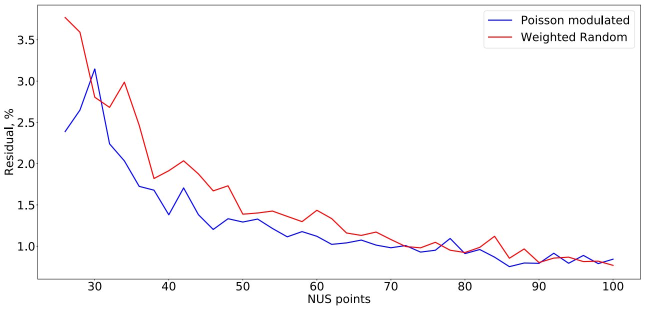

R74N-H

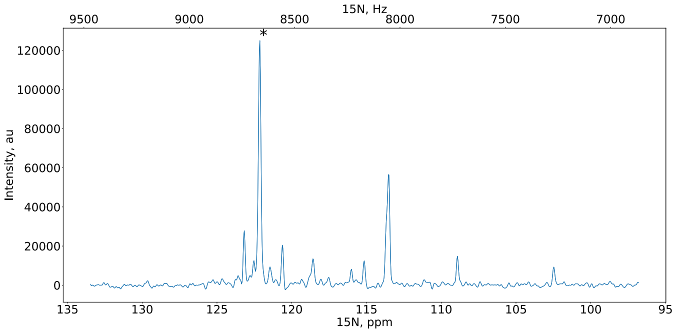

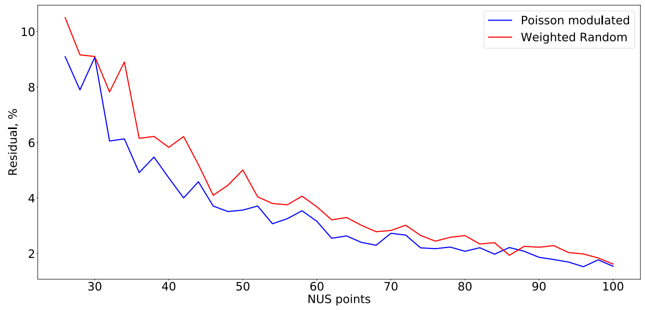

G75N-H

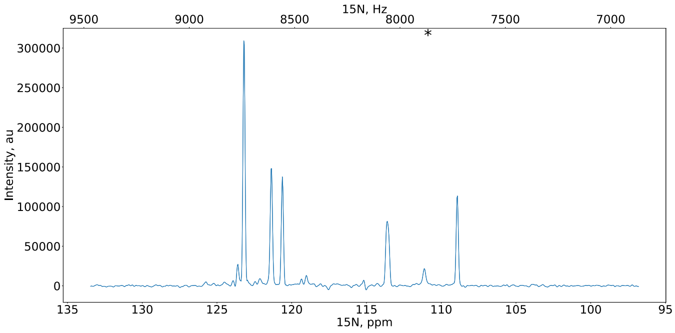

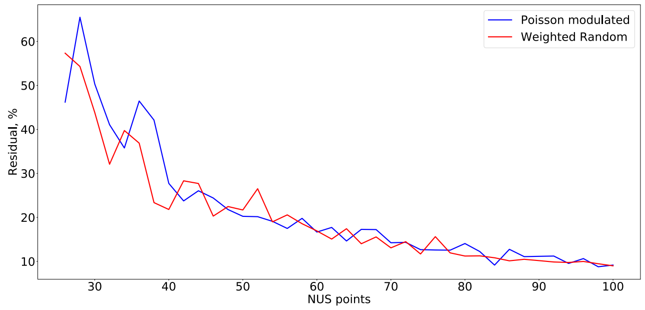

G76N-H

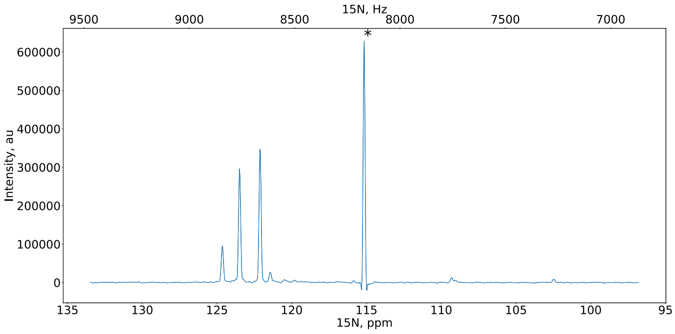

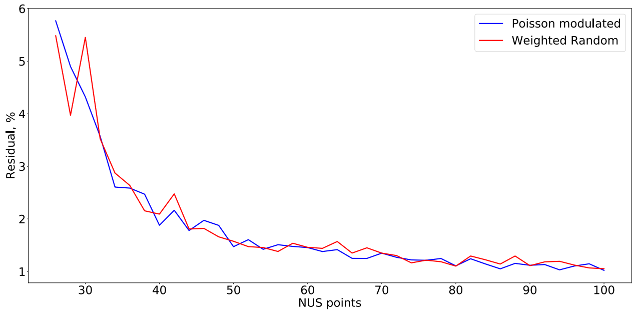

Supplement: Supplementary file 1 — Supplementary file1 (PDF 3695 kb) [file 10858_2021_385_MOESM1_ESM.pdf]
