# Supplementary material for: Clustered sparsity and Poisson-gap sampling
Source: J Biomol NMR. 2021 Nov 5;75(10-12):401–16. doi: 10.1007/s10858-021-00385-7 (PMC8642362; doi:10.1007/s10858-021-00385-7)

Supplementary Information:

Clustered sparsity and Poisson-gap sampling

Paweł Kasprzak[1,2], Mateusz Urbańczyk[1,3], and Krzysztof Kazimierczuk[1]

[1]Centre of New Technologies, University of Warsaw, Banacha 2C, 02-097 Warsaw, Poland
[2]Faculty of Physics, University of Warsaw, Pasteura 5, 02-093 Warsaw, Poland
[3]Institute of Physical Chemistry, Polish Academy of Sciences, Kasprzaka 44/52,01-224 Warsaw, Poland

Analysis of ^{13}C-HSQC spectrum of ubiquitin

Peak:1

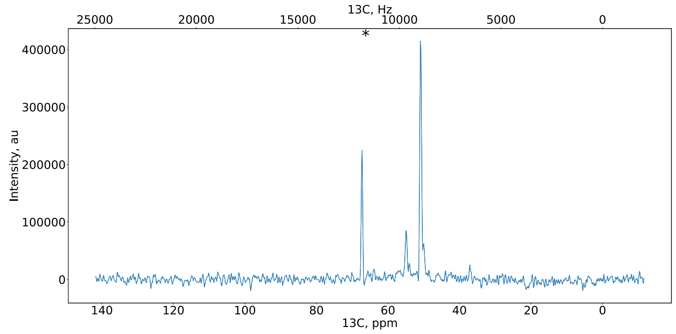

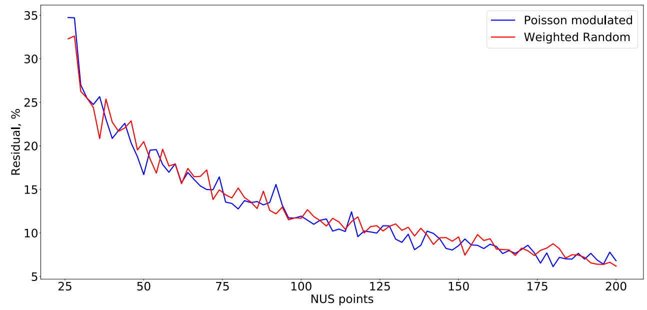

Peak:2

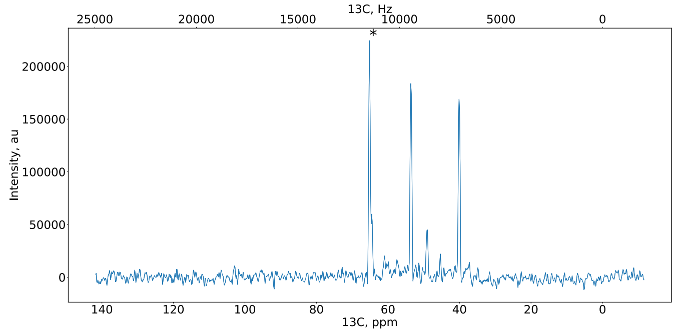

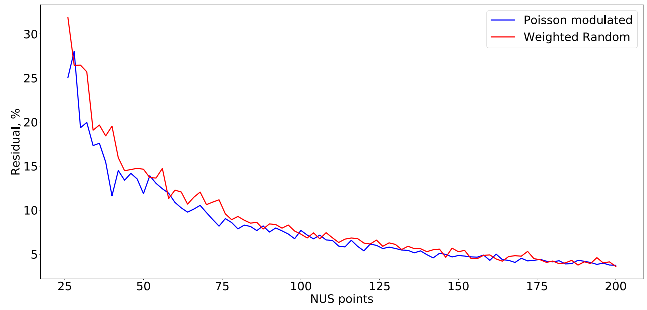

Peak:3

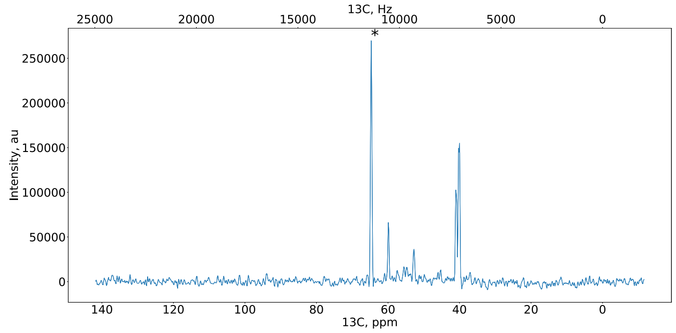

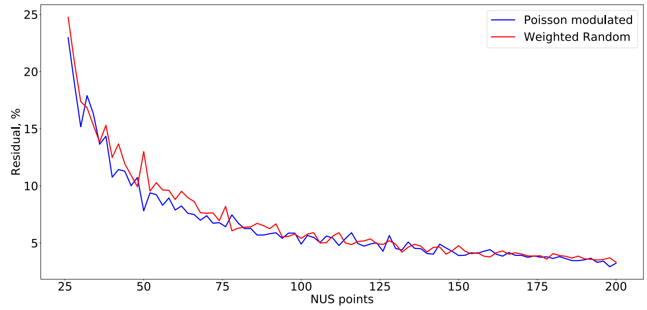

Peak:4

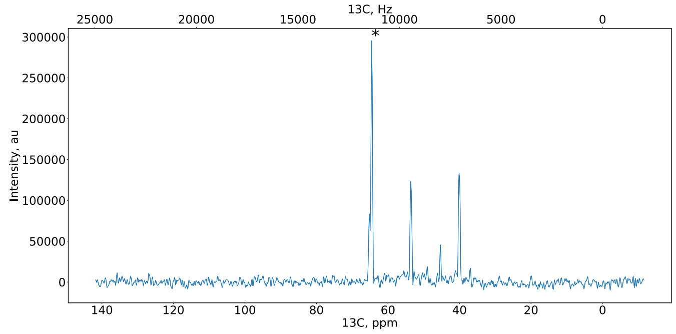

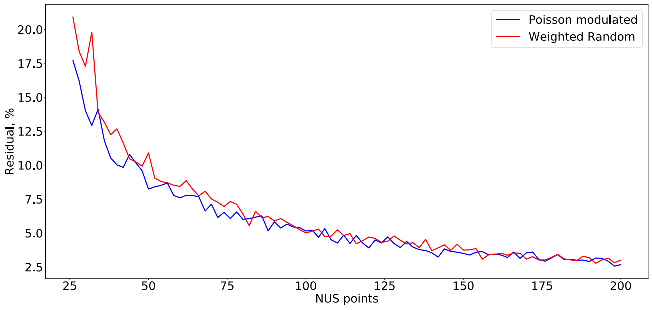

Peak:5

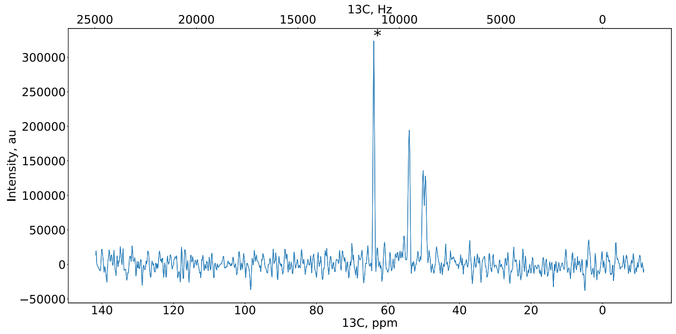

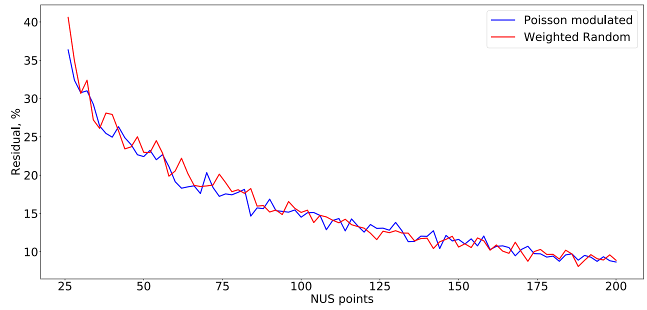

Peak:6

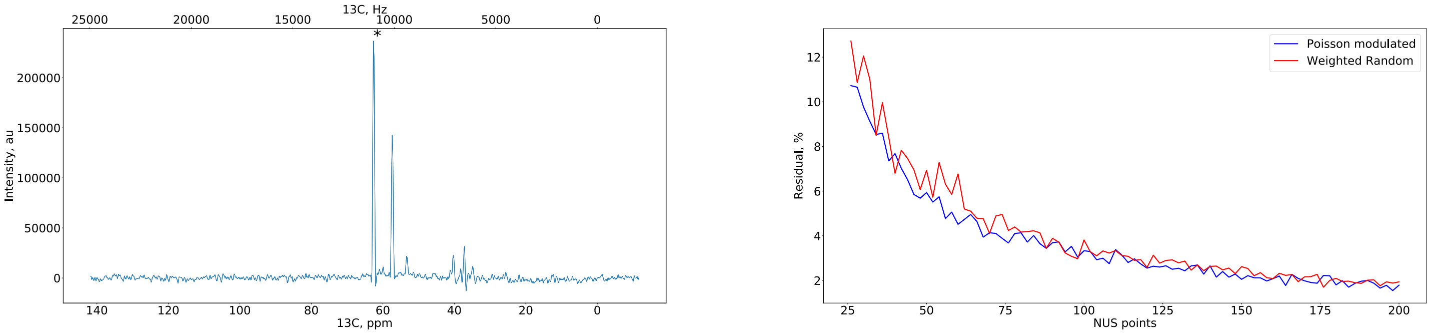

Peak:7

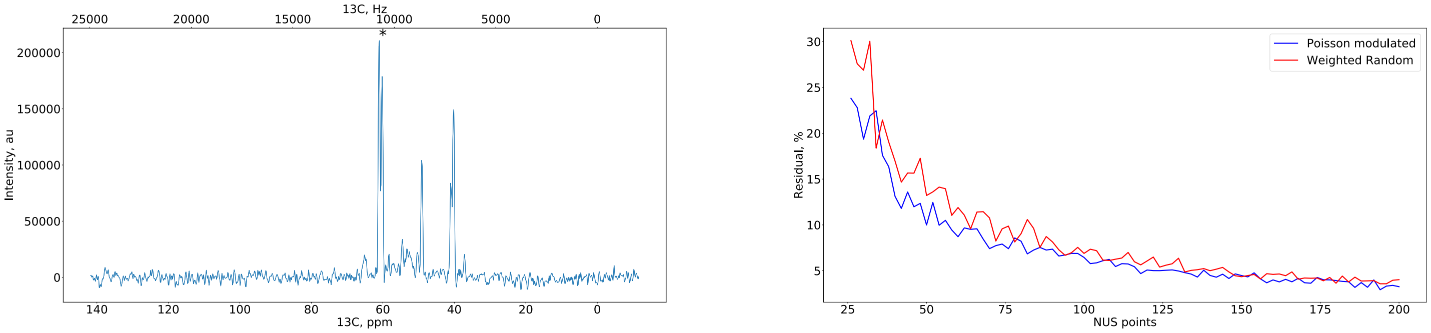

Peak:8

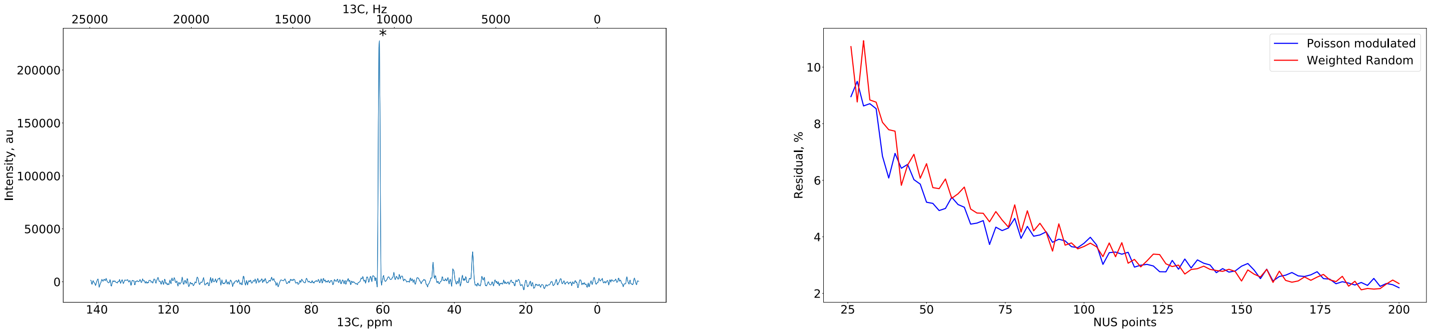

Peak:9

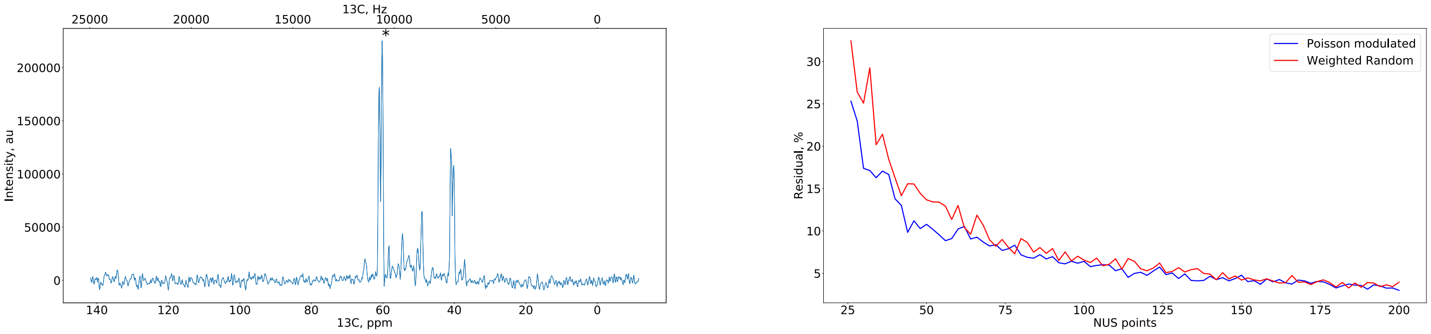

Peak:10

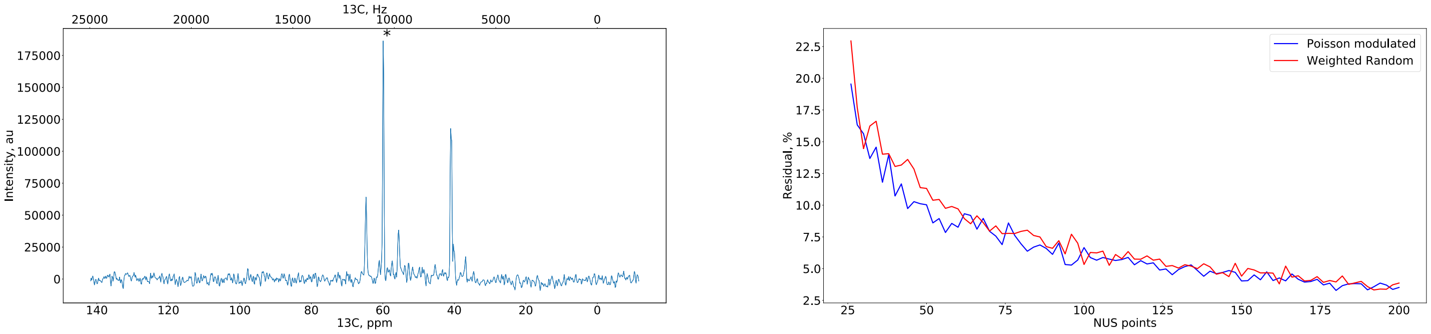

Peak:11

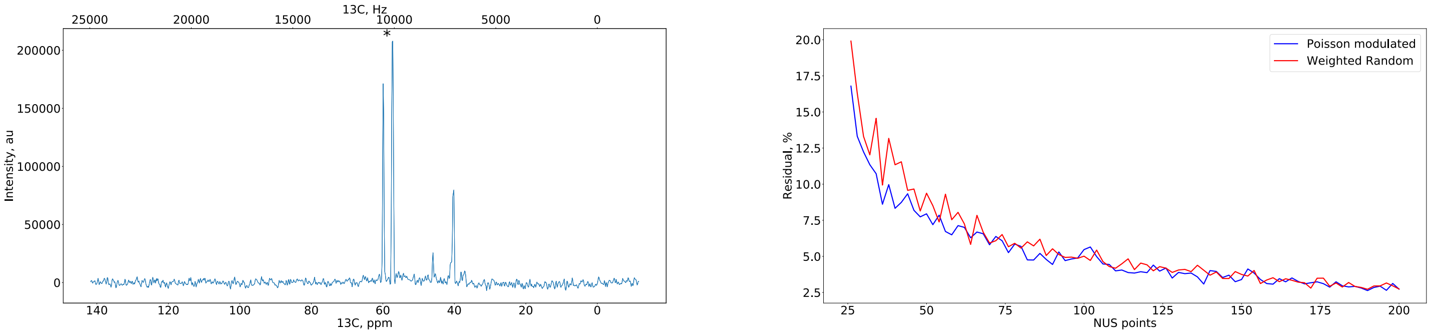

Peak:12

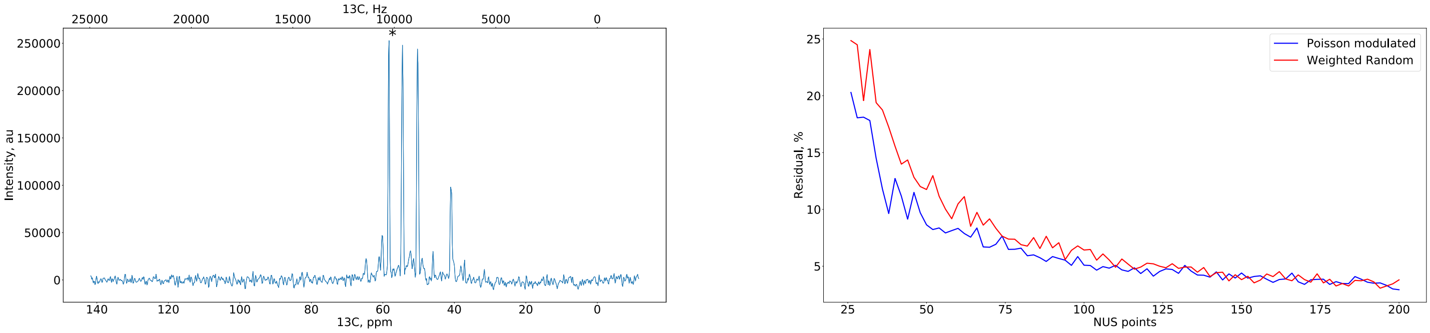

Peak:13

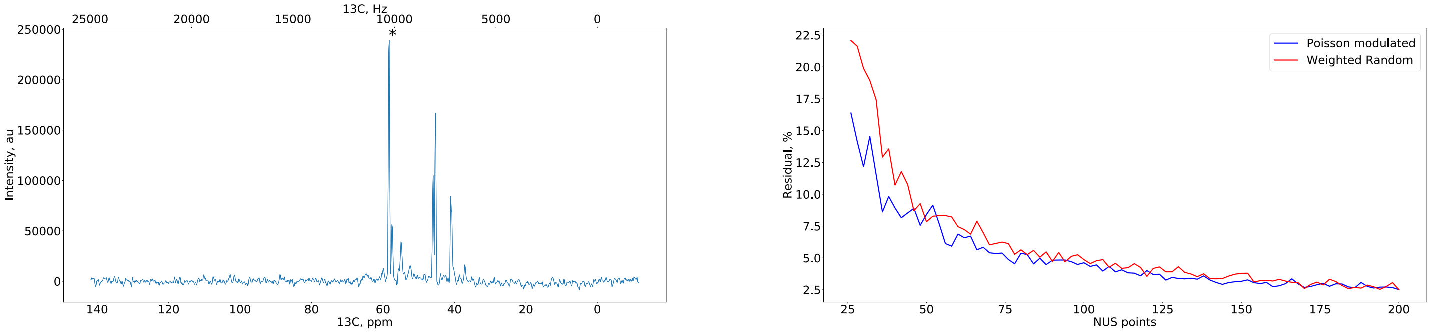

Peak:14

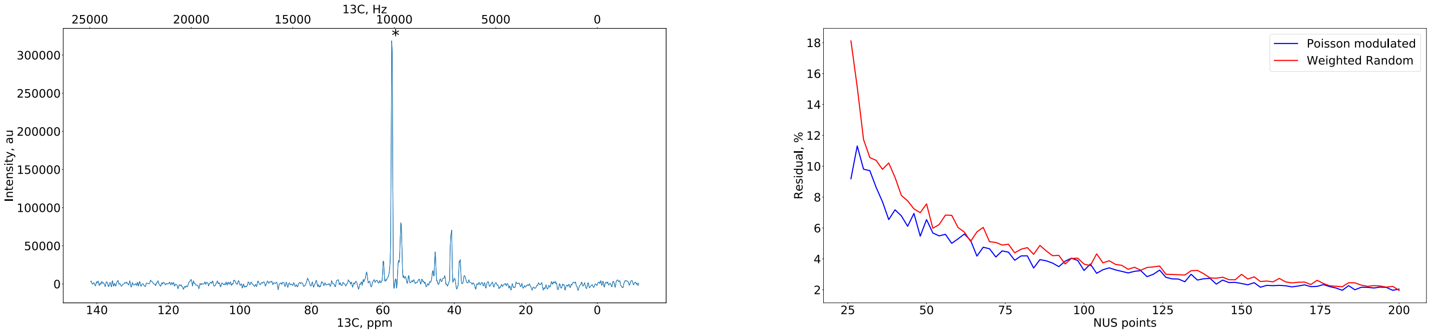

Peak:15

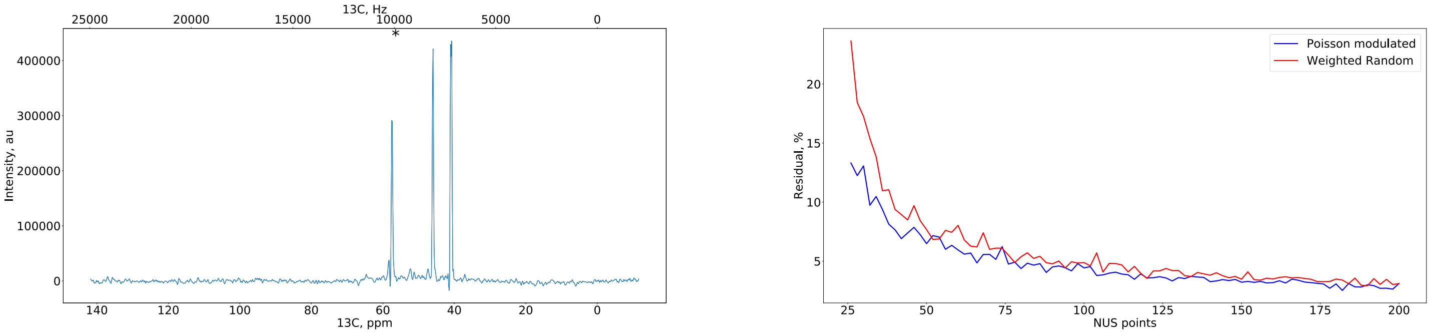

Peak:16

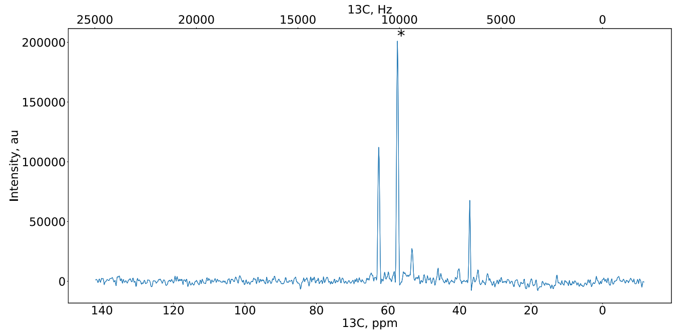

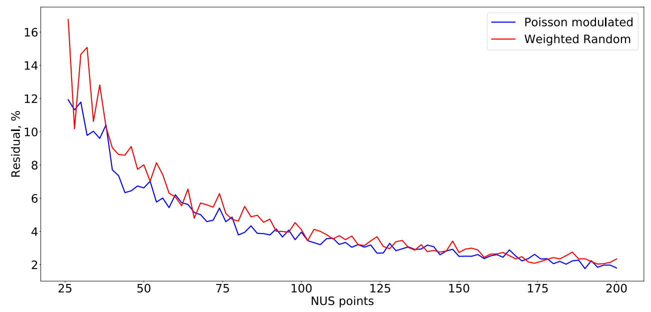

Peak:17

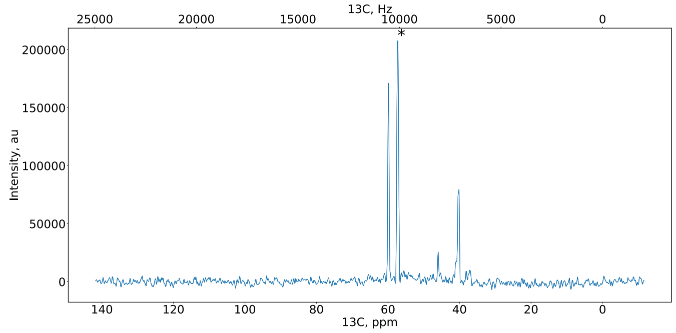

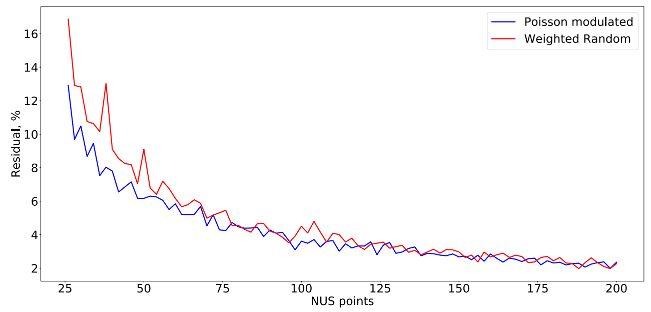

Peak:18

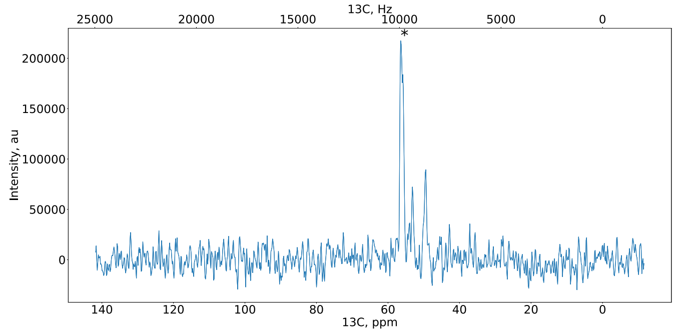

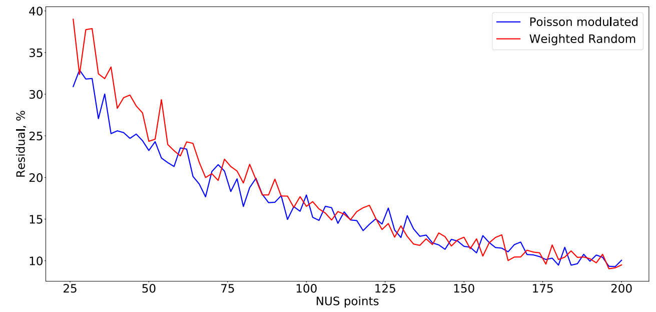

Peak:19

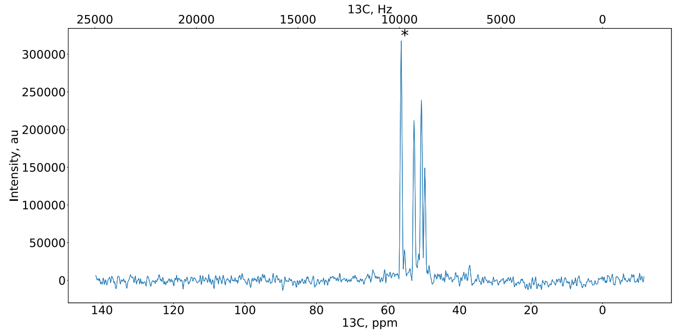

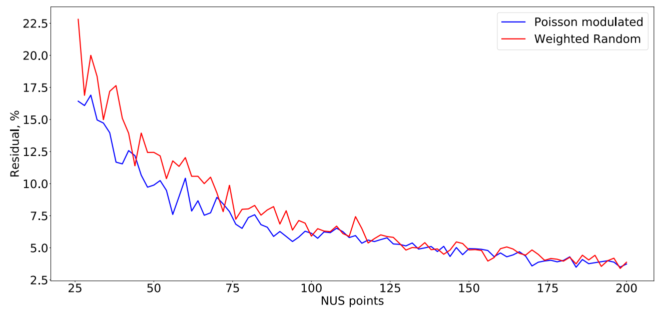

Peak:20

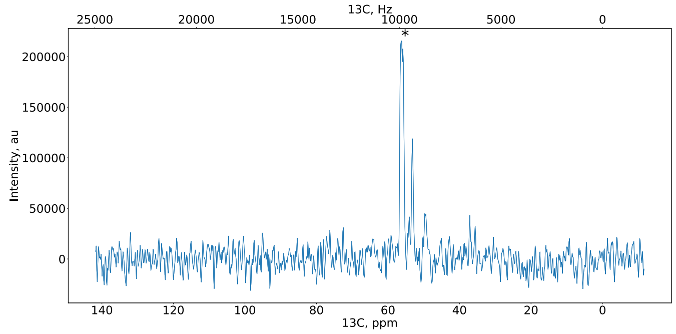

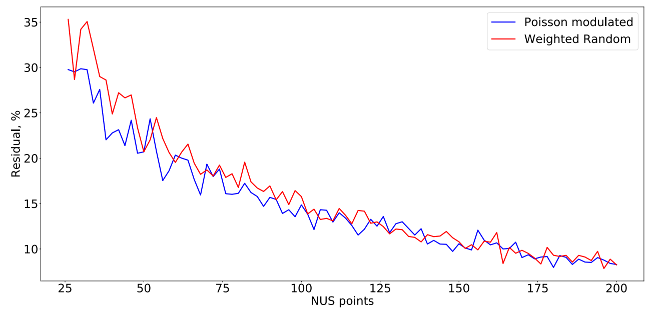

Peak:21

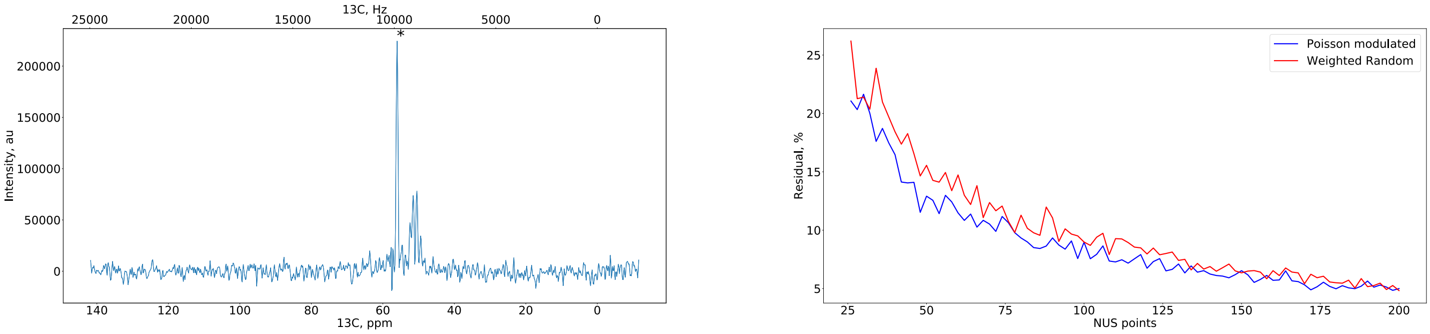

Peak:22

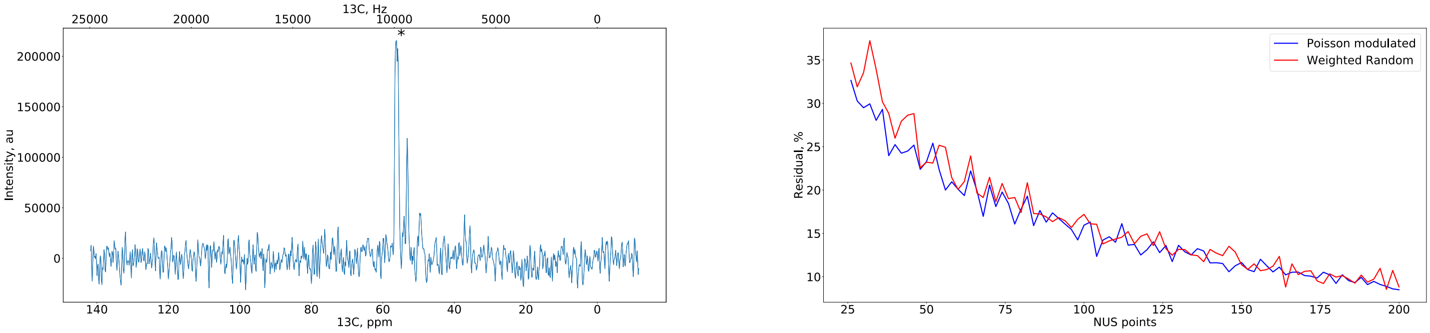

Peak:23

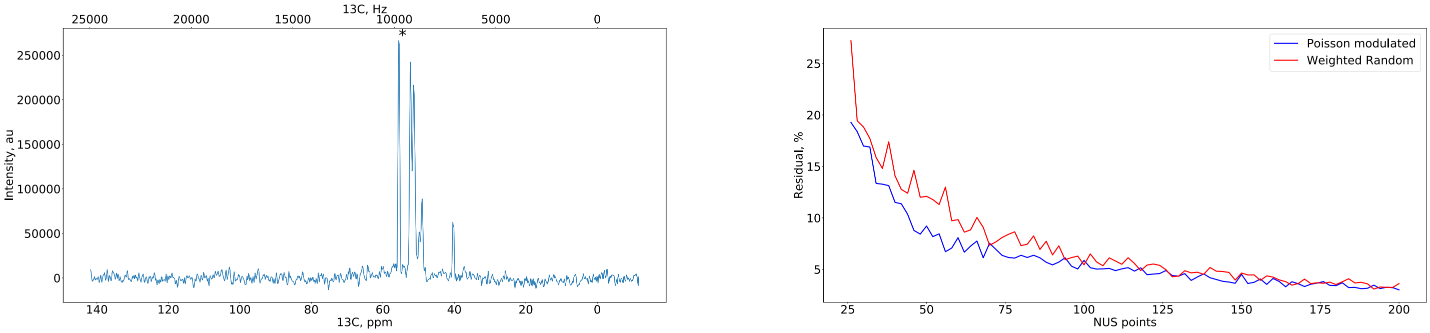

Peak:24

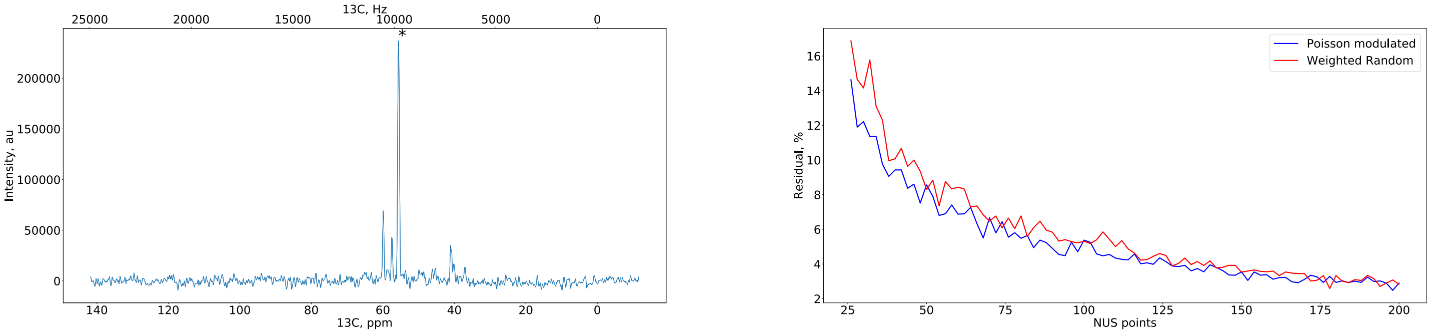

Peak:25

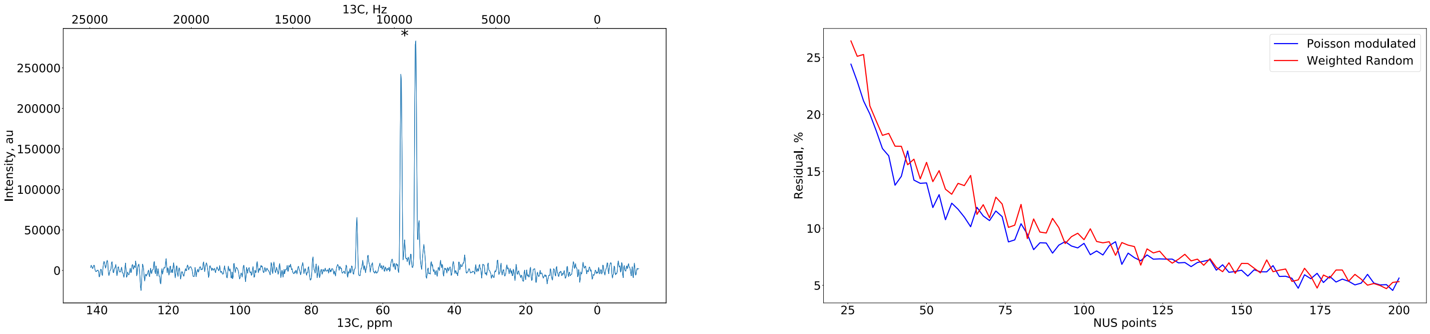

Peak:26

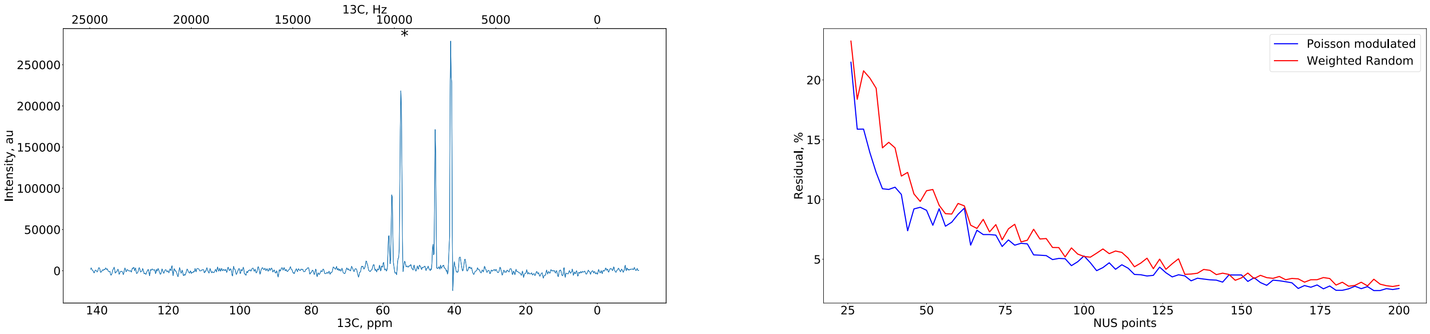

Peak:27

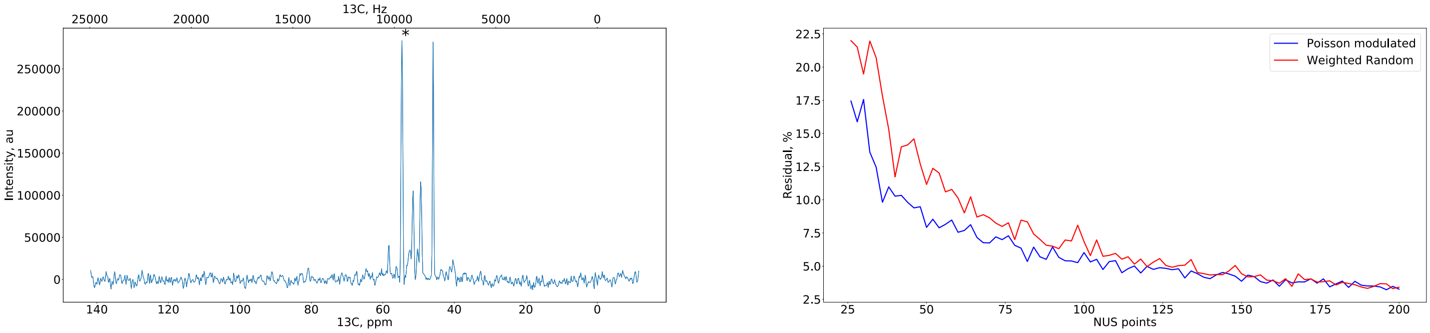

Peak:28

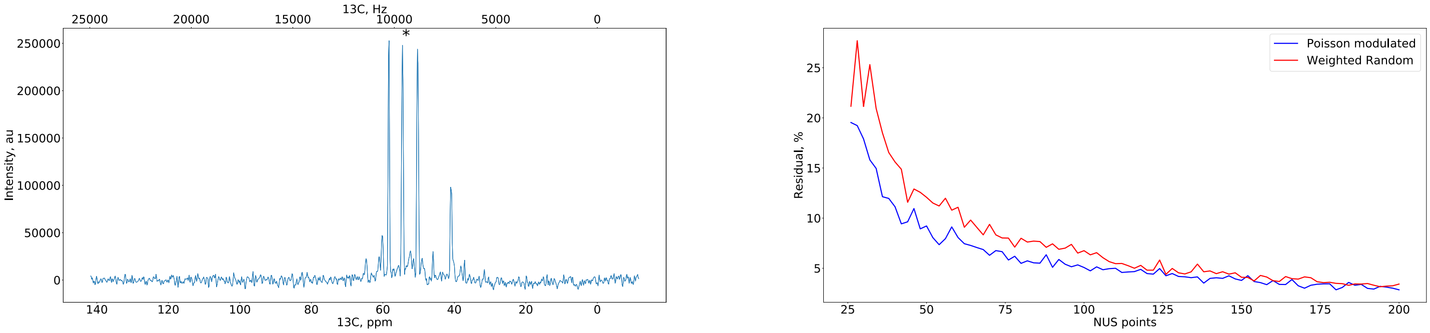

Peak:29

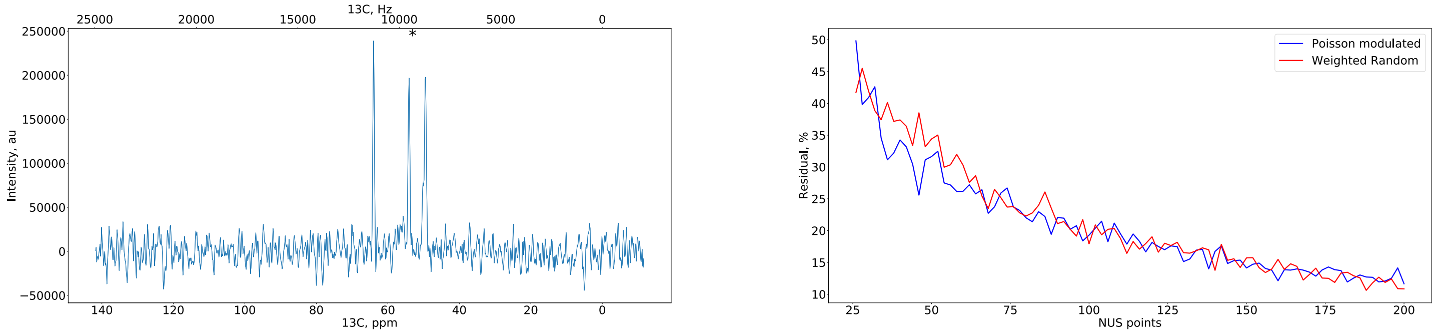

Peak:30

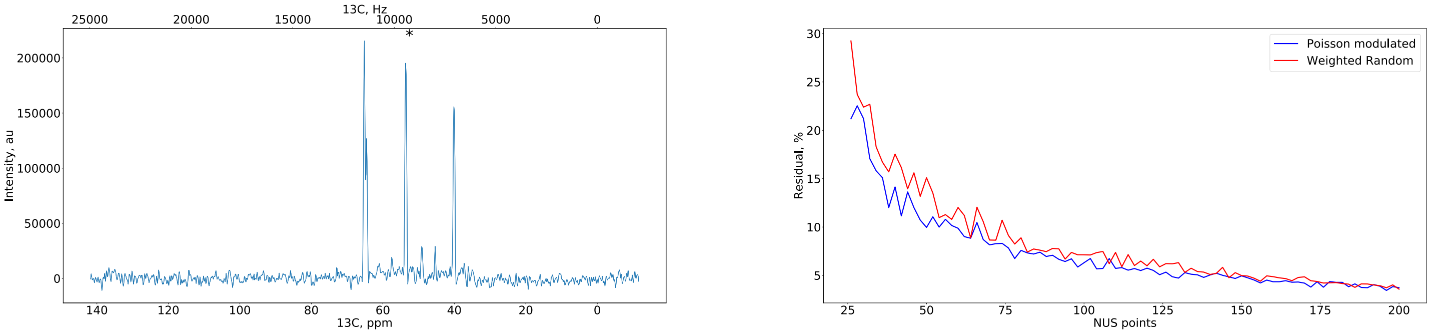

Peak:31

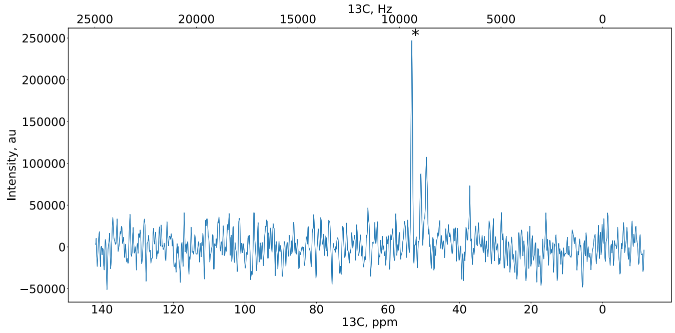

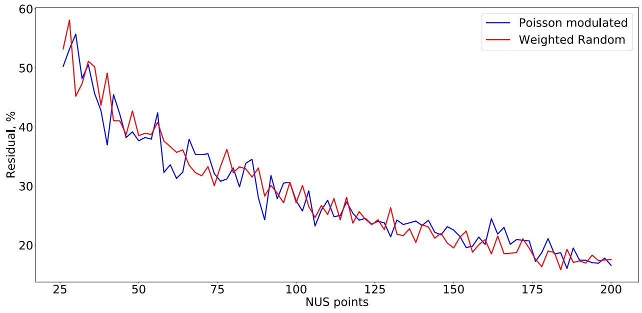

Peak:32

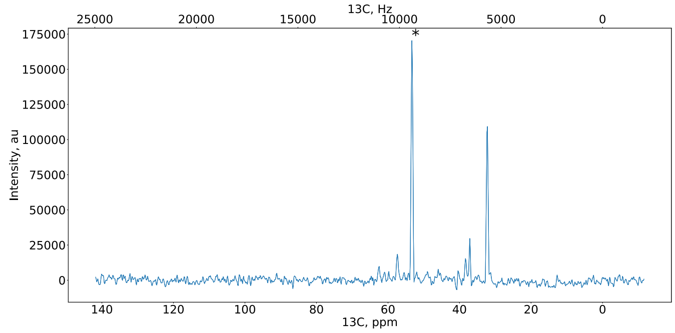

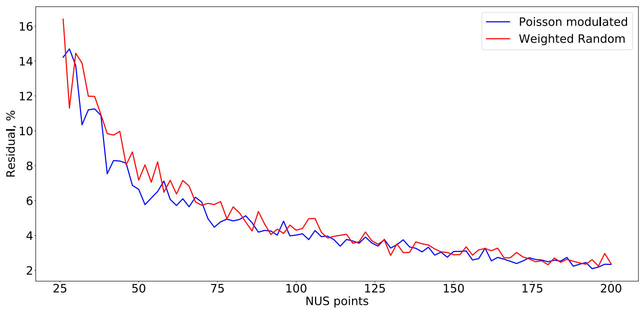

Peak:33

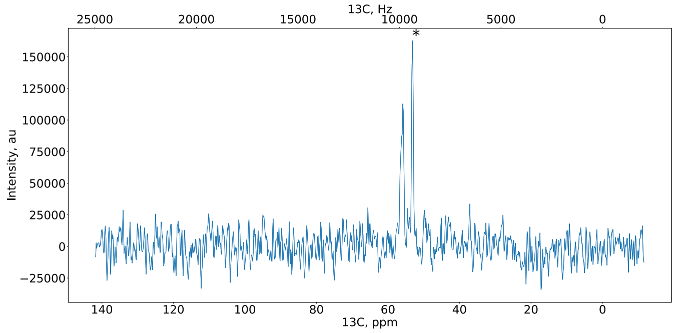

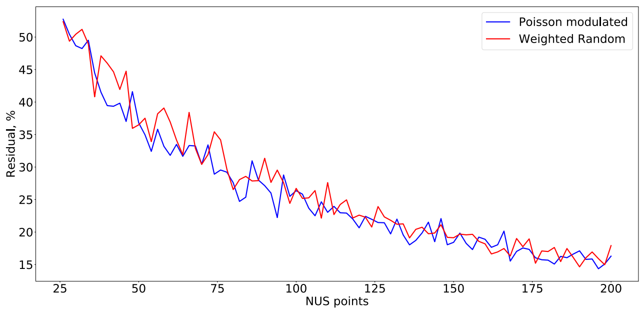

Peak:34

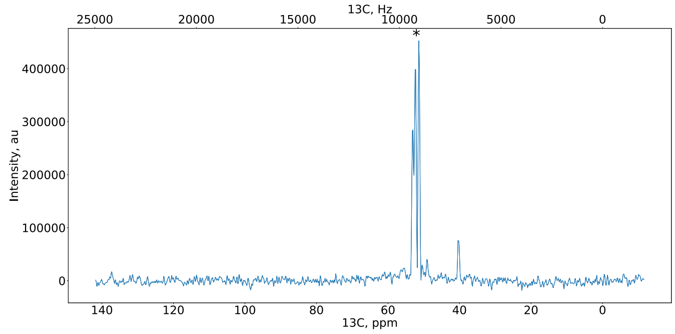

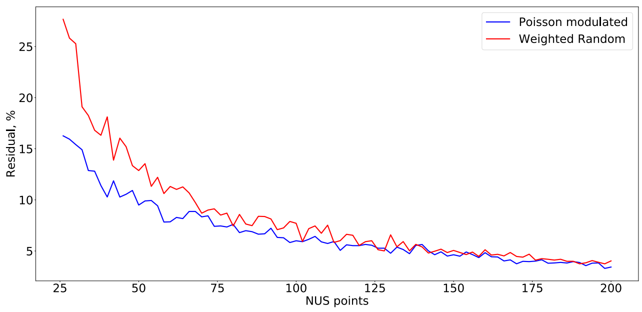

Peak:35

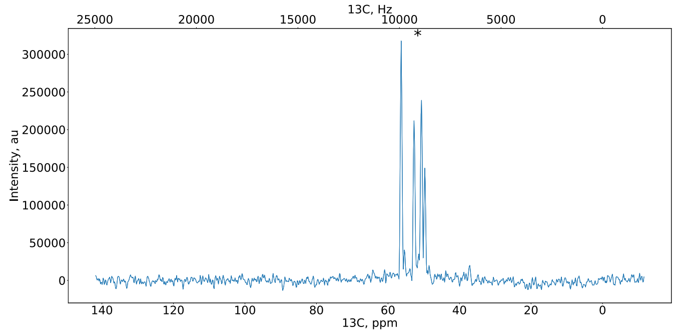

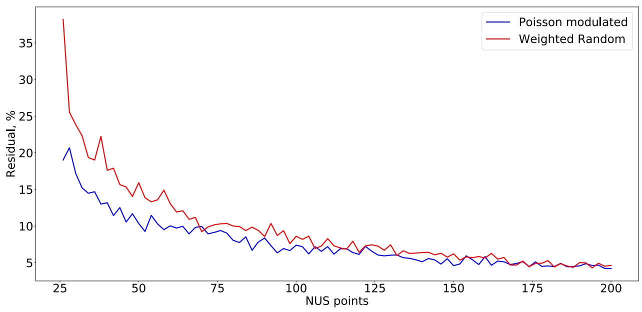

Peak:36

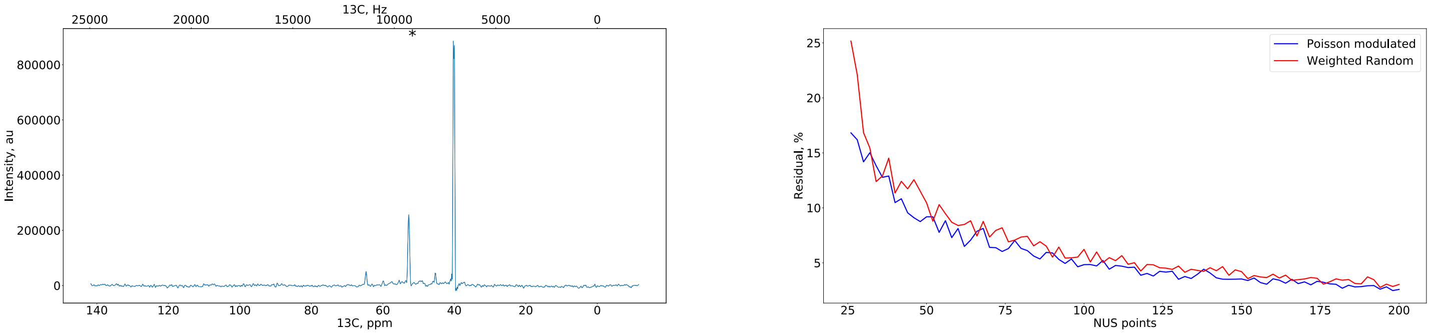

Peak:37

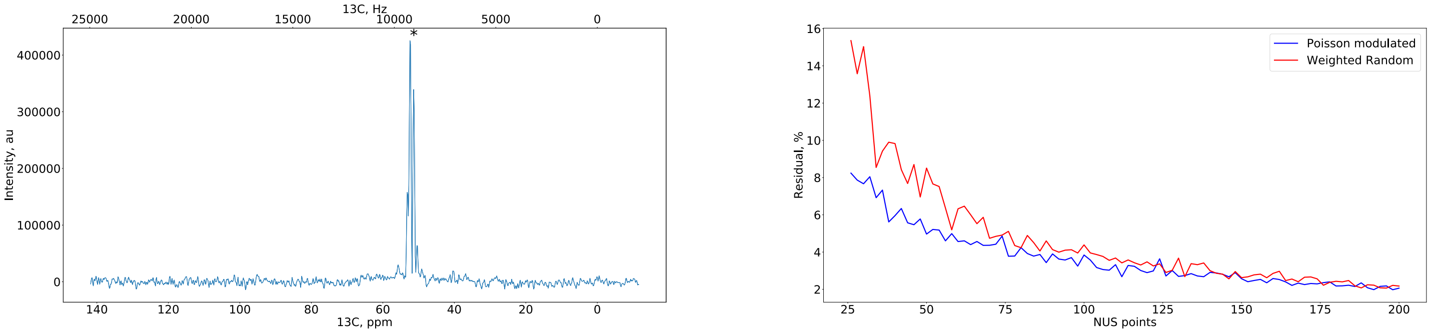

Peak:38

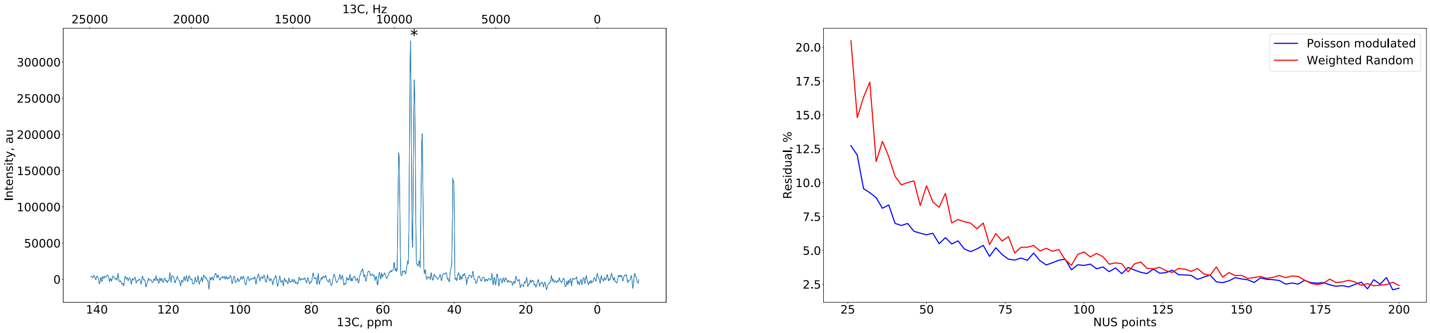

Peak:39

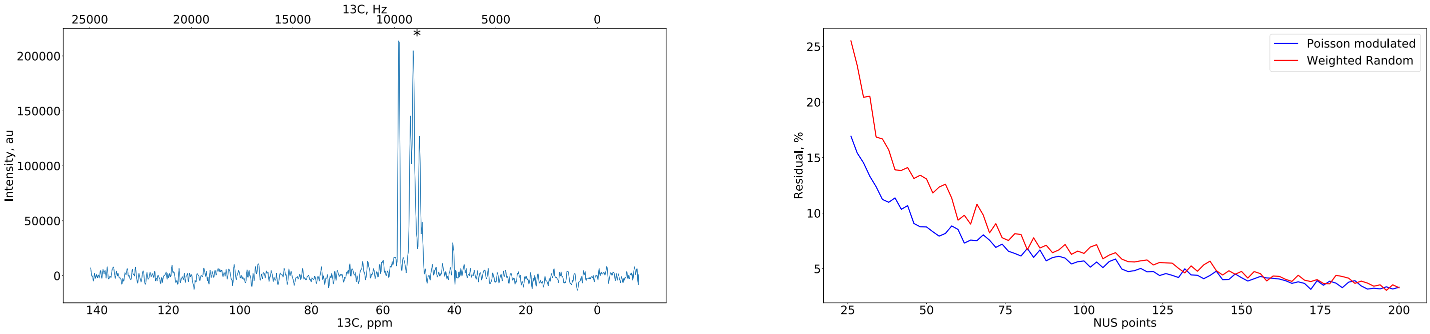

Peak:40

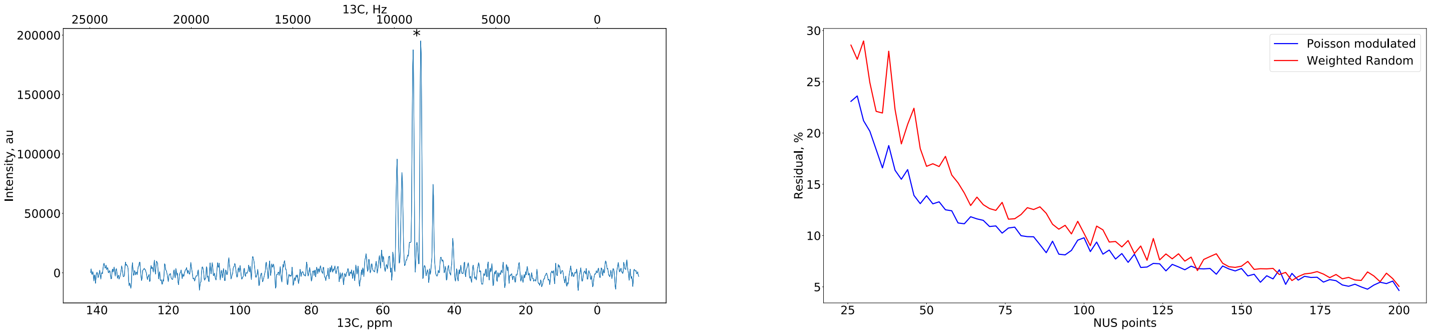

Peak:41

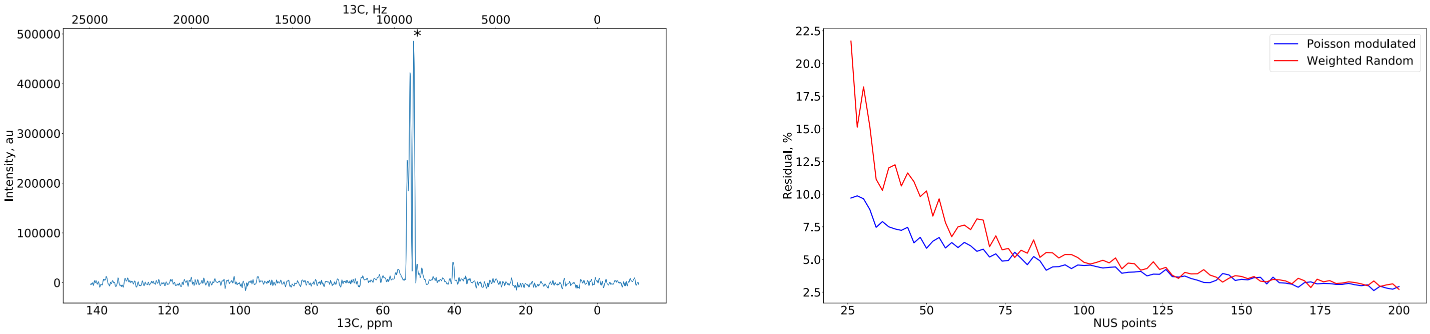

Peak:42

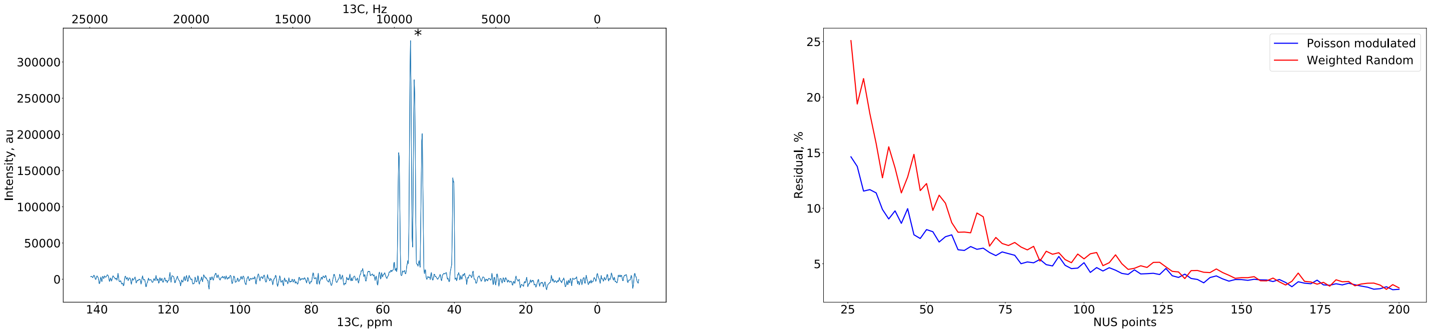

Peak:43

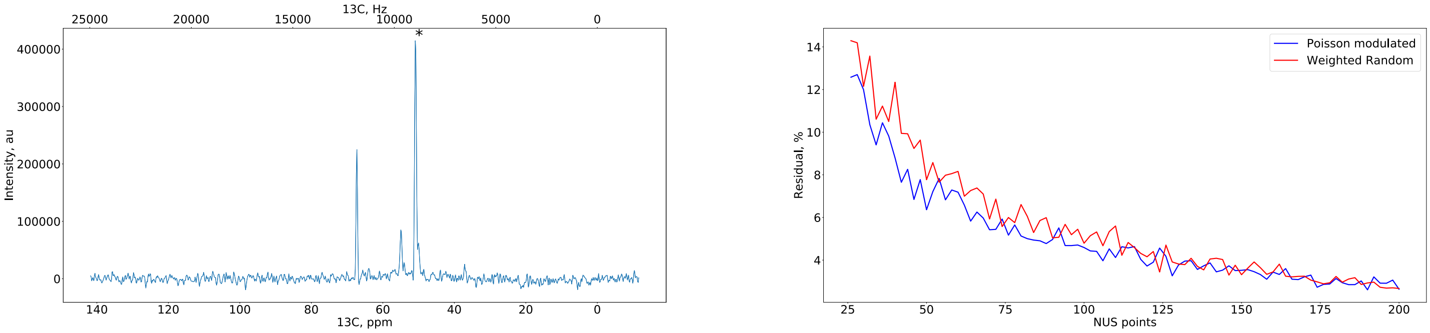

Peak:44

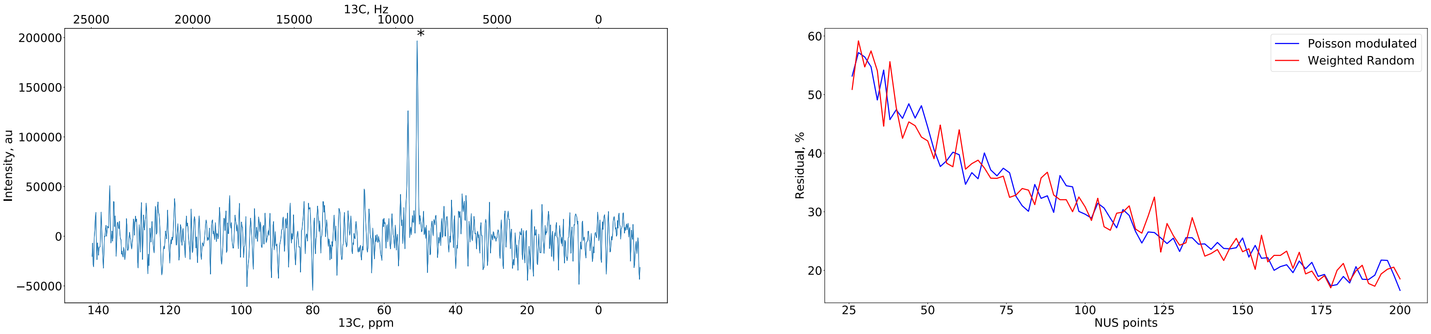

Peak:45

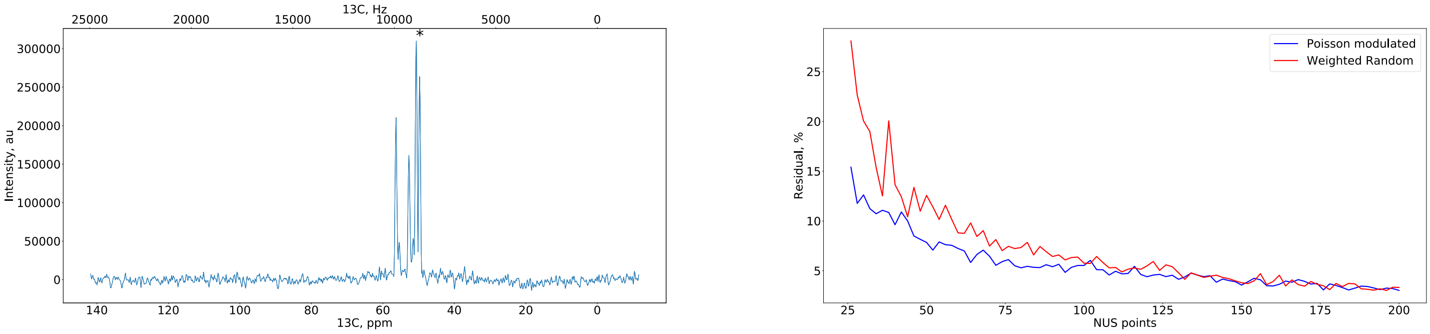

Peak:46

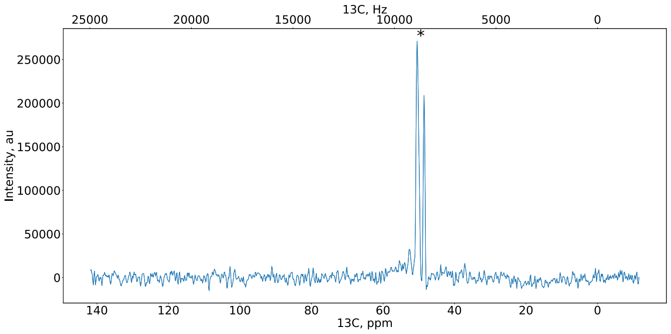

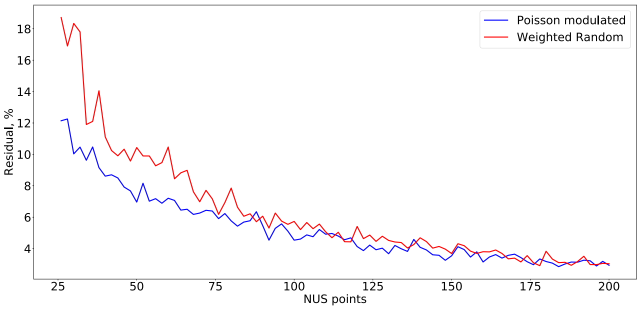

Peak:47

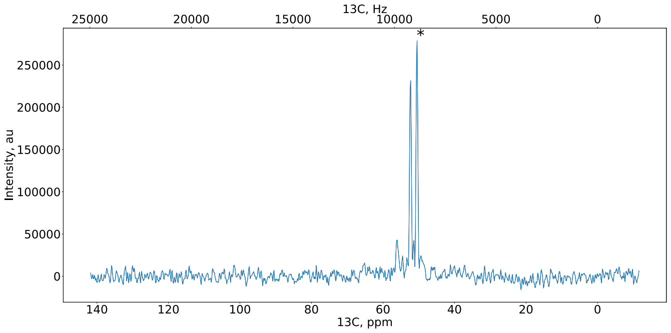

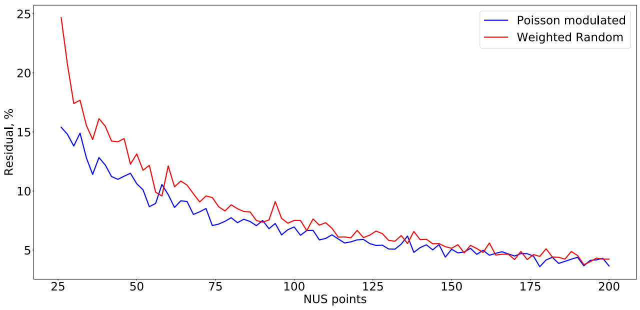

Peak:48

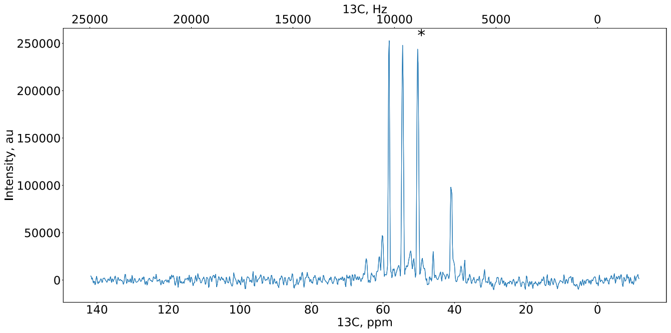

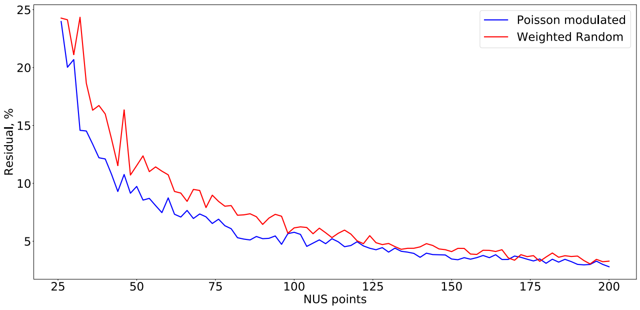

Peak:49

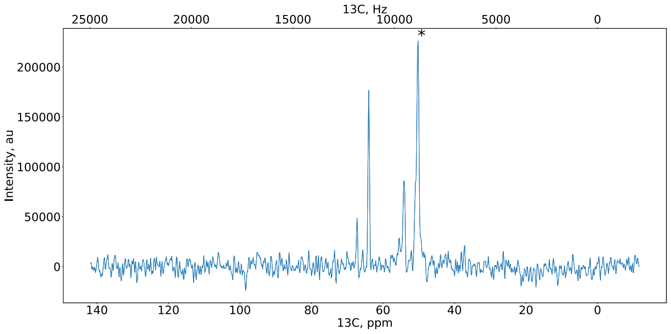

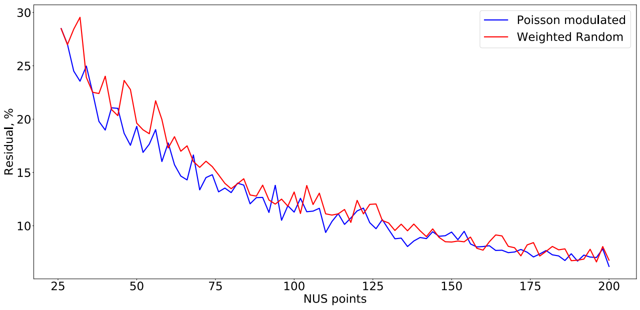

Peak:50

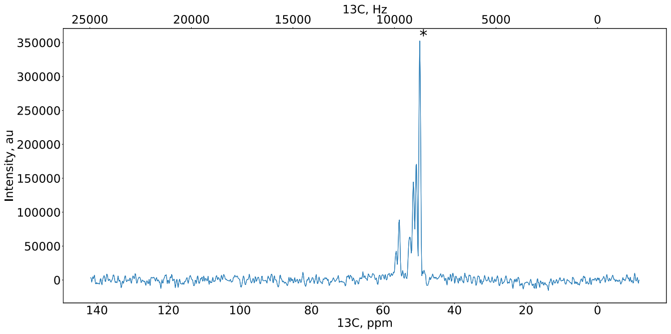

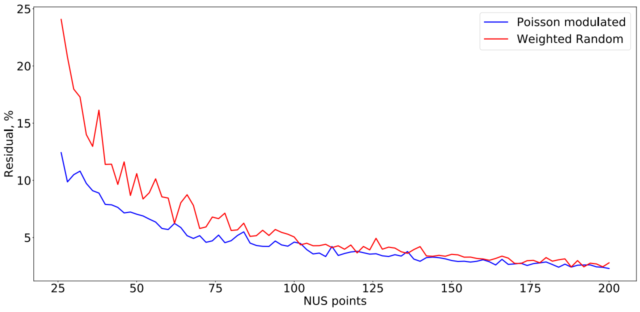

Peak:51

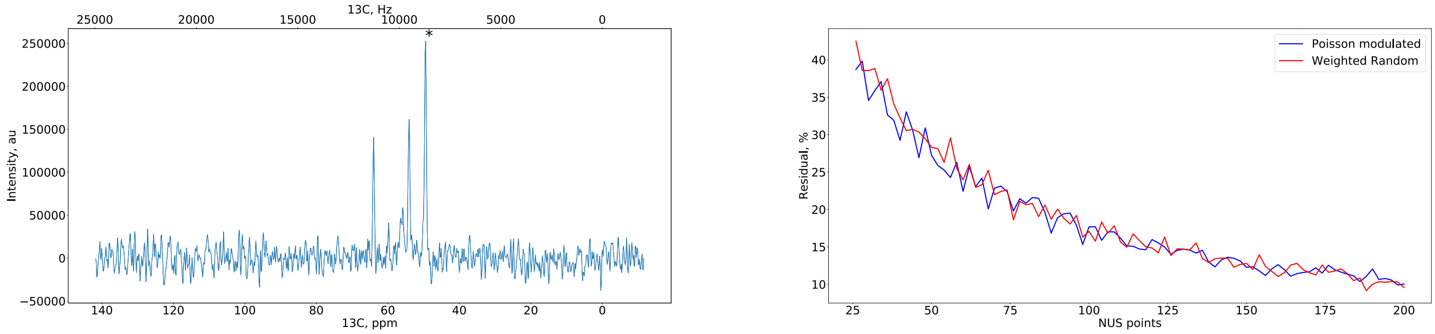

Peak:52

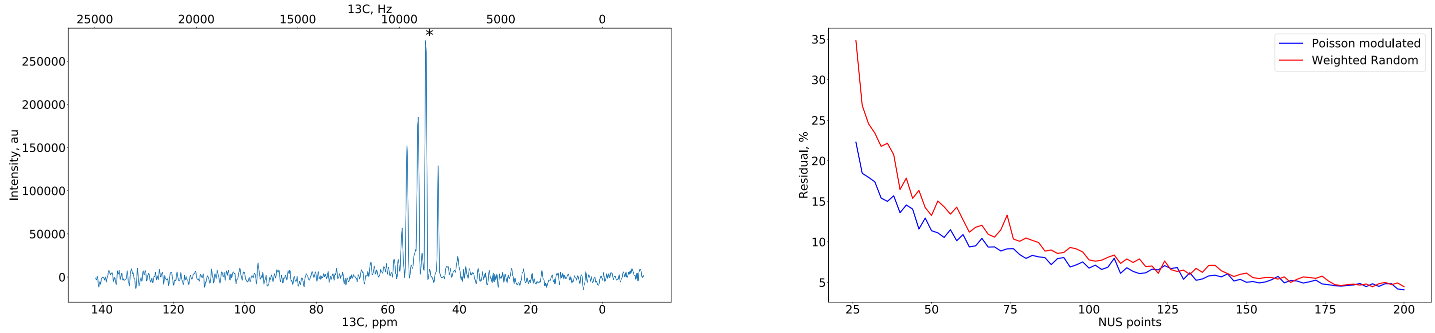

Peak:53

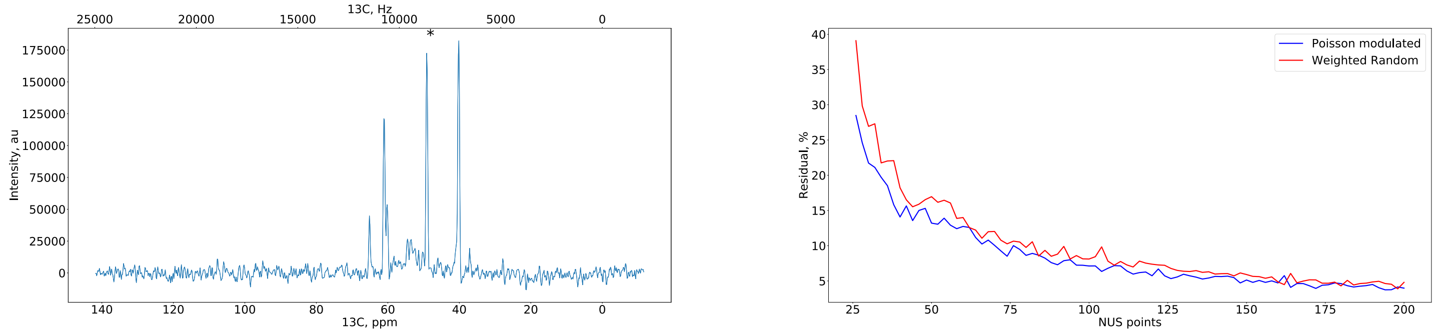

Peak:54

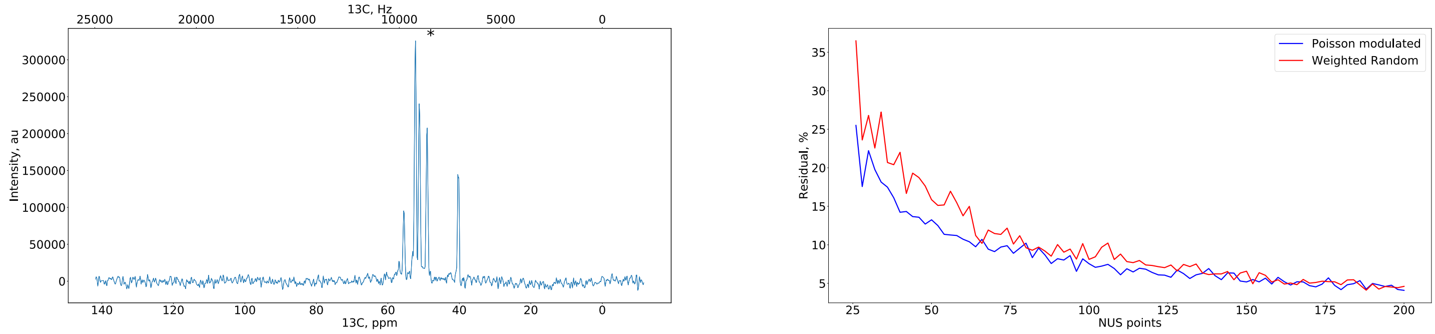

Peak:55

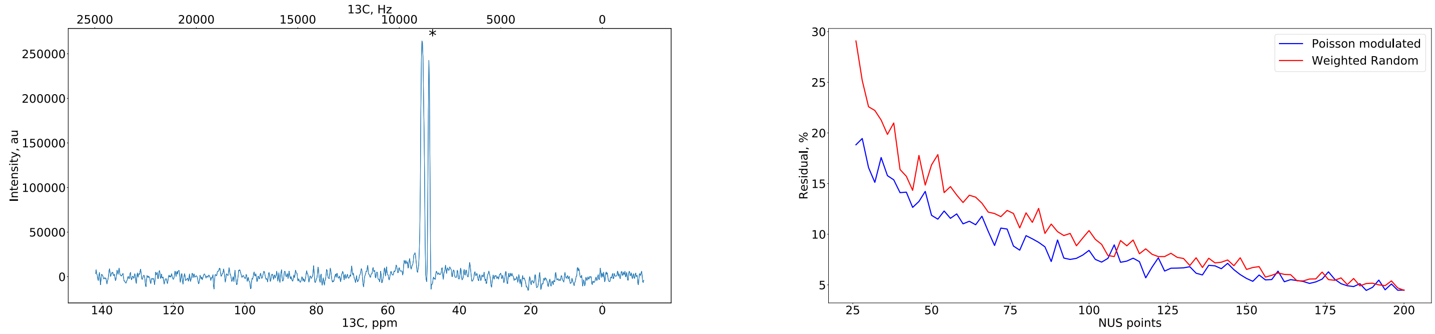

Peak:56

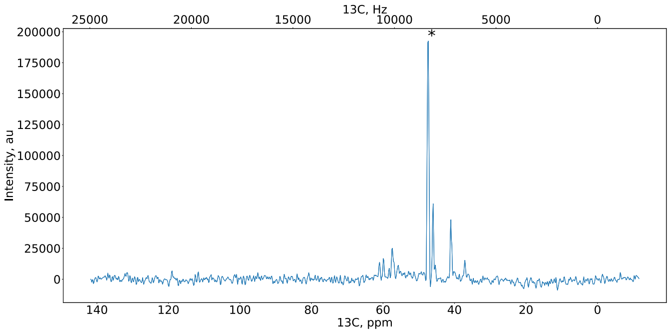

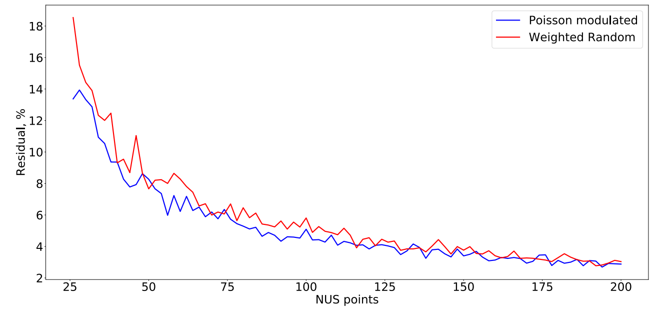

Peak:57

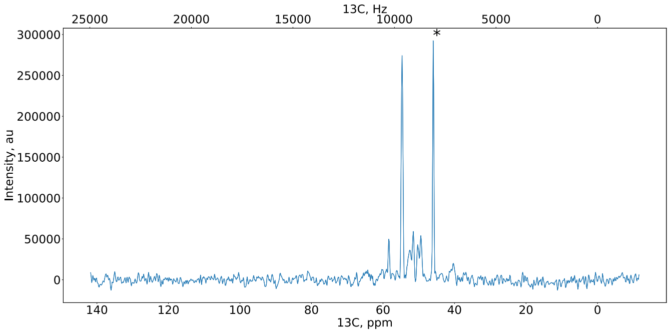

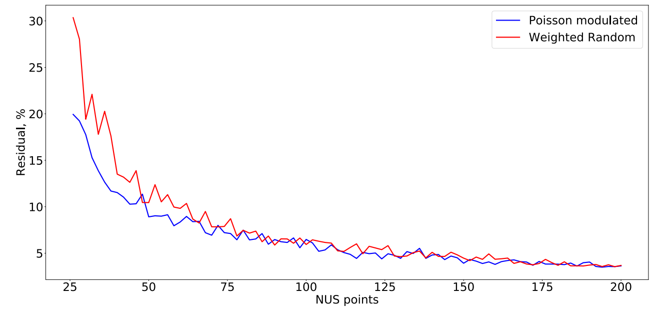

Peak:58

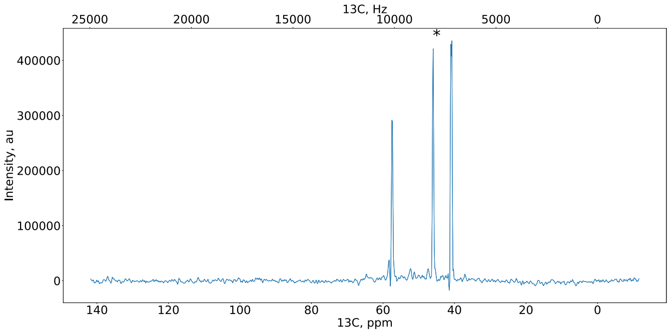

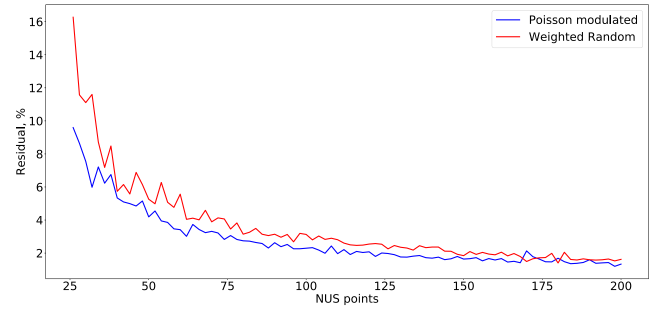

Peak:59

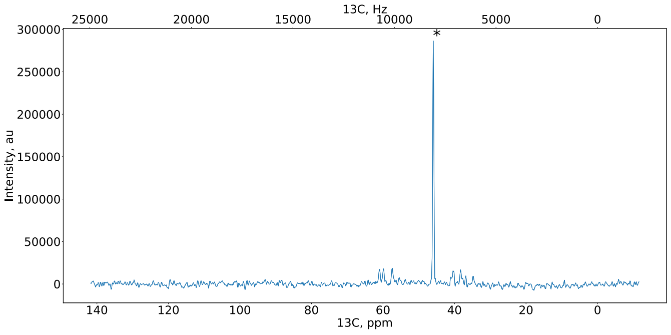

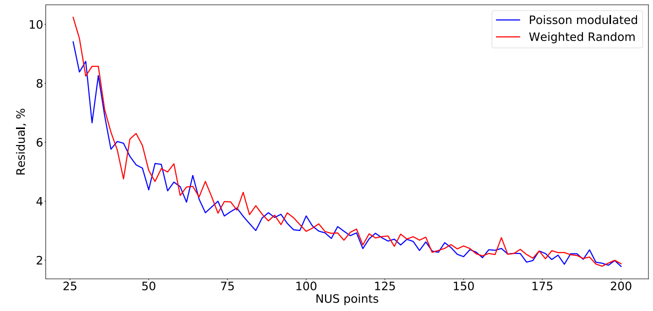

Peak:60

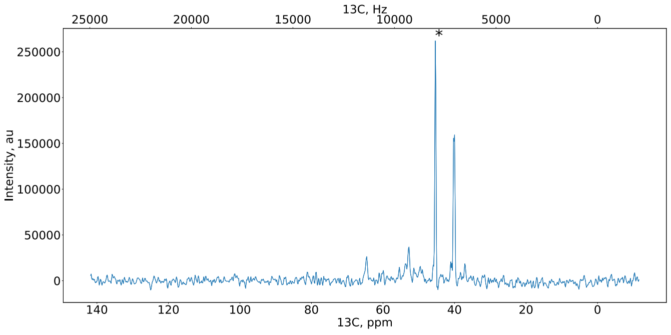

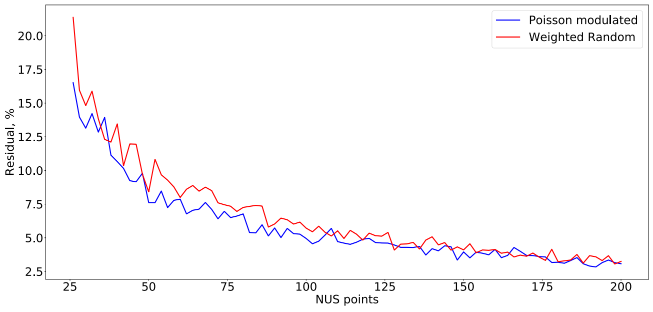

Peak:61

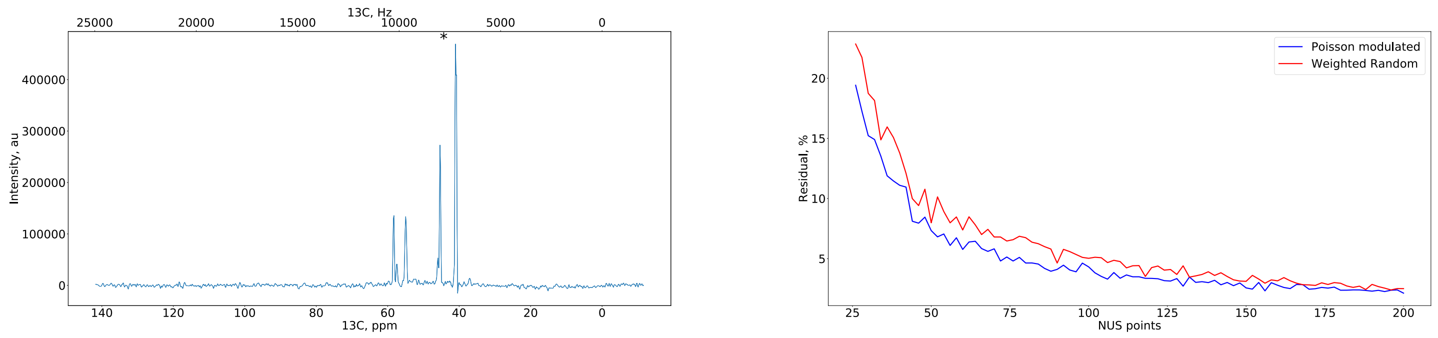

Peak:62

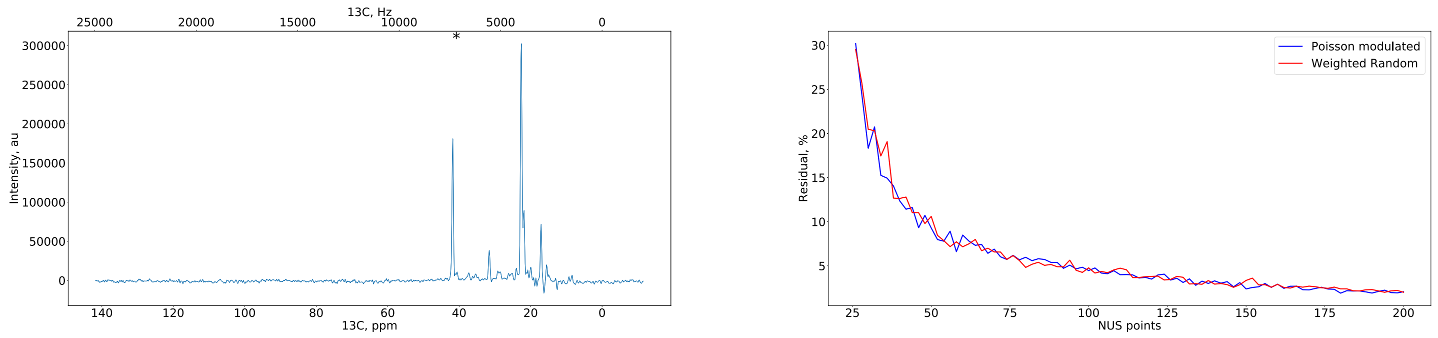

Peak:63

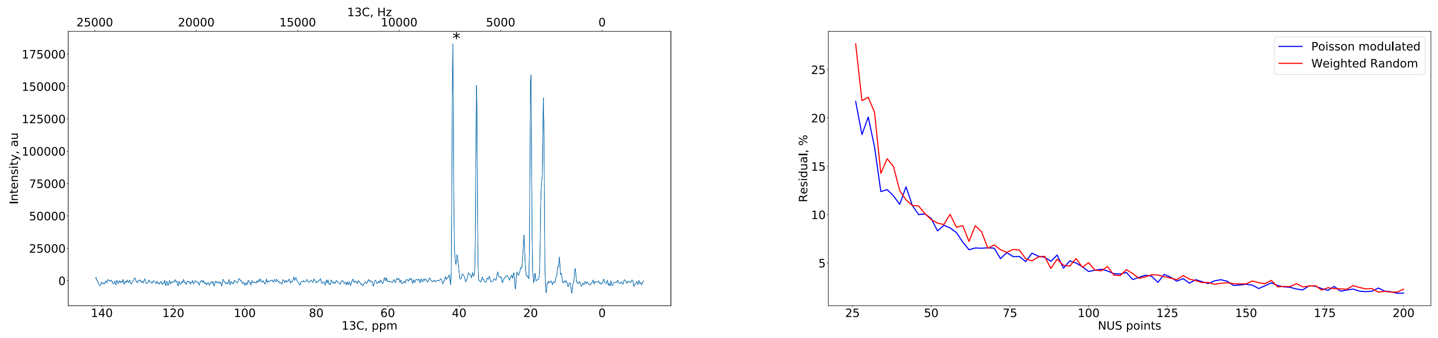

Peak:64

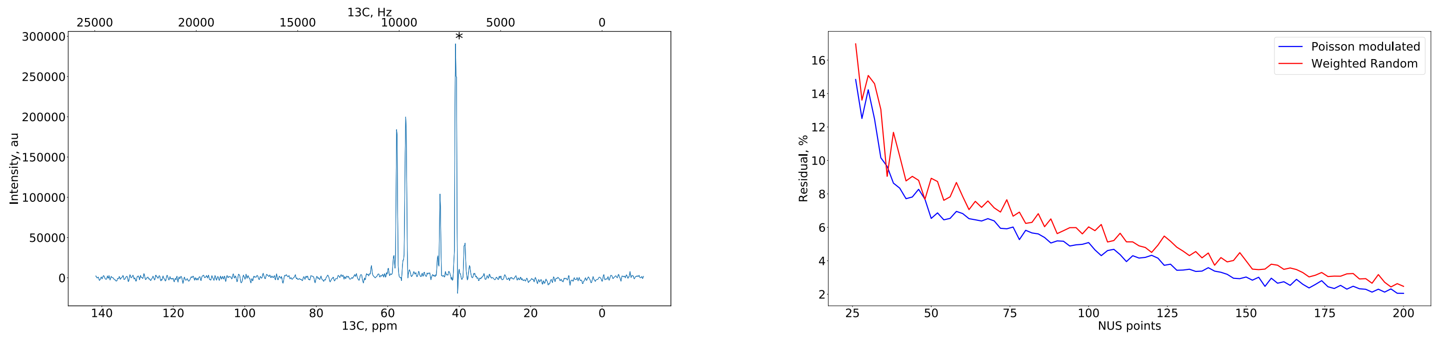

Peak:65

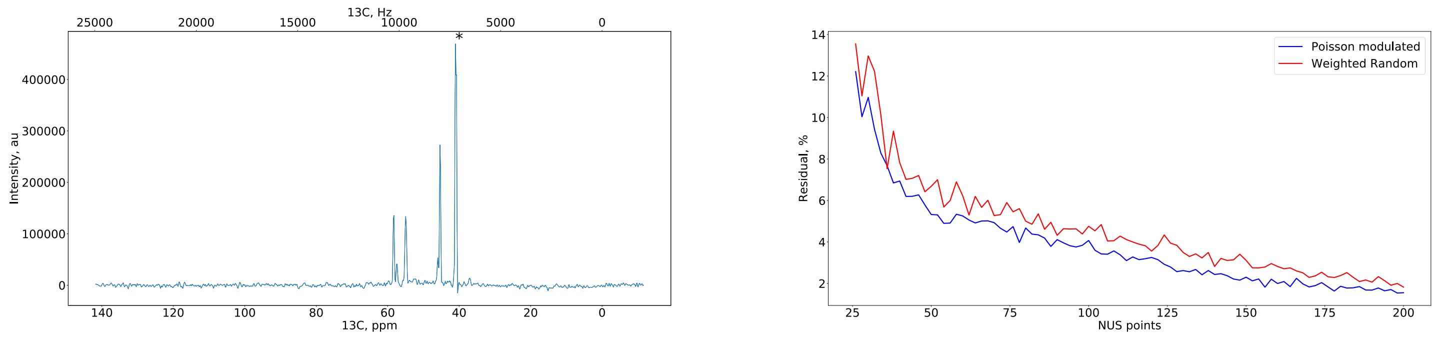

Peak:66

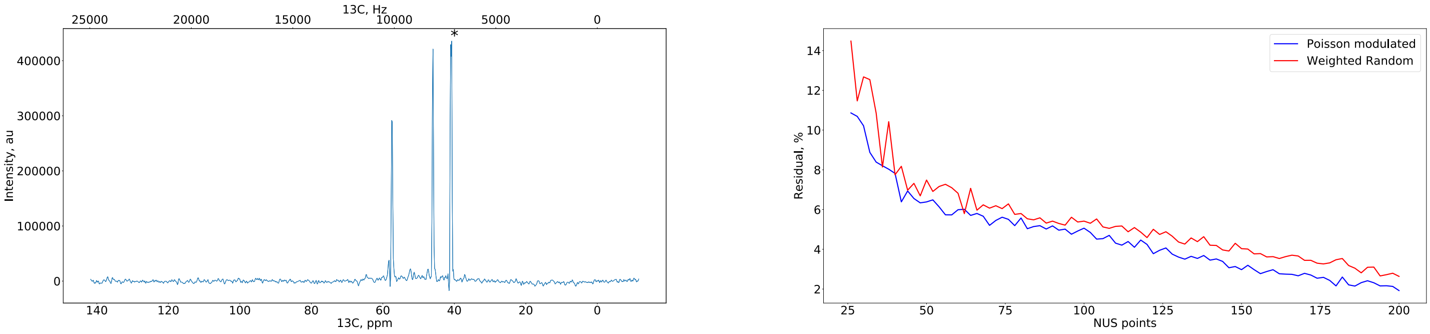

Peak:67

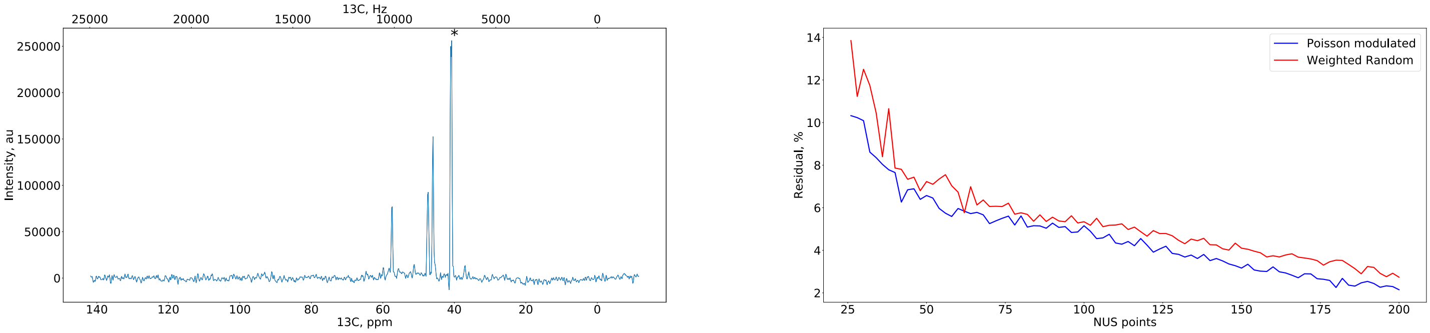

Peak:68

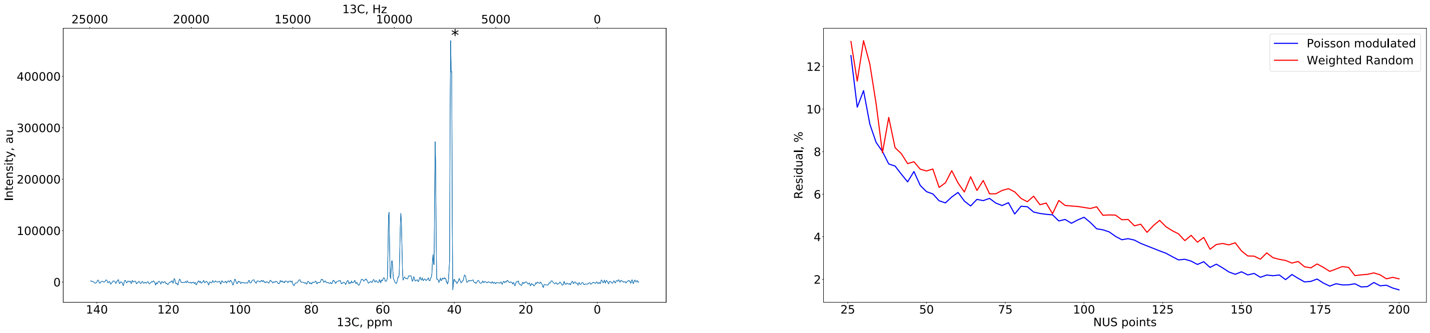

Peak:69

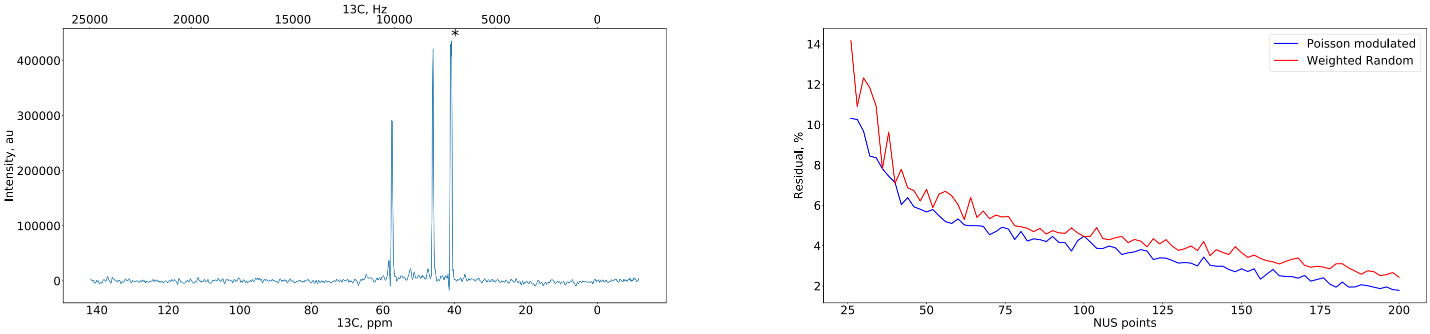

Peak:70

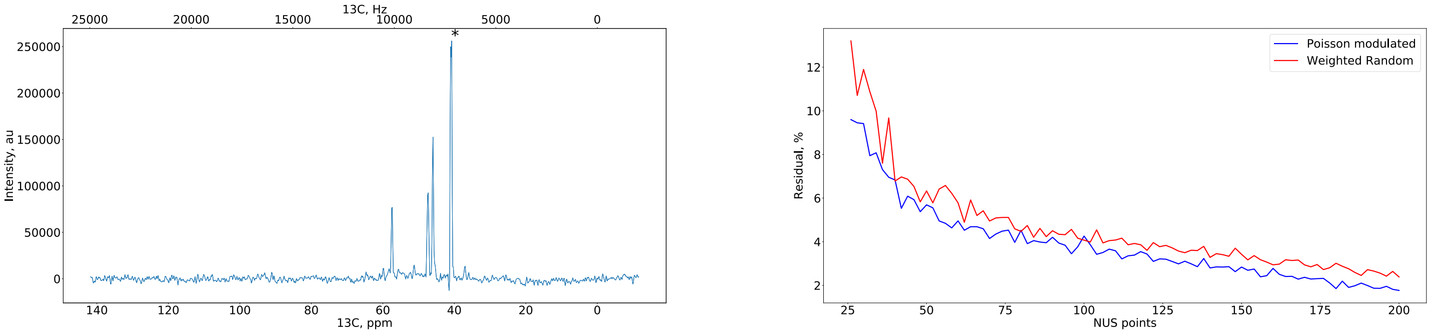

Peak:71

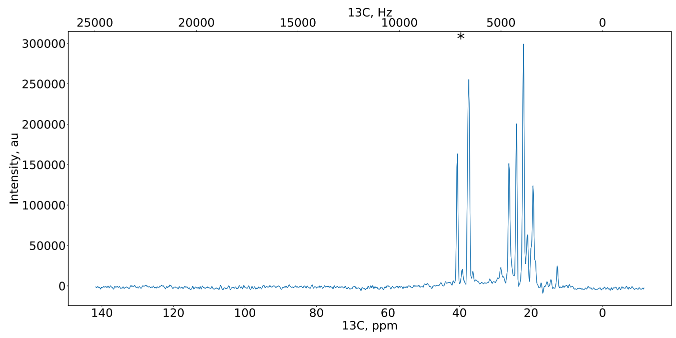

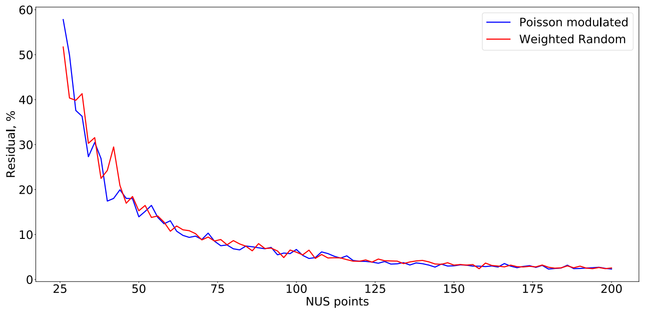

Peak:72

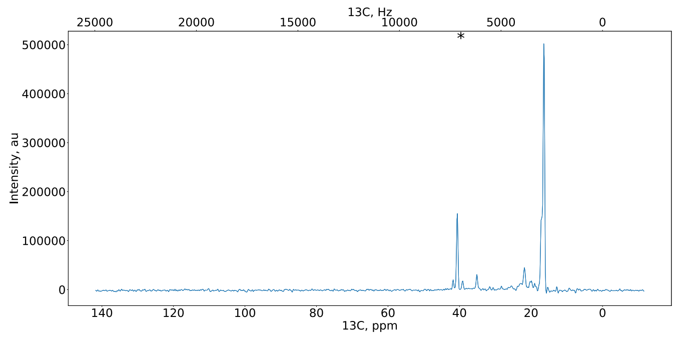

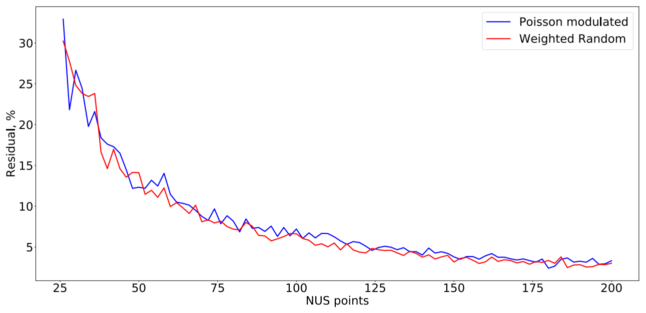

Peak:73

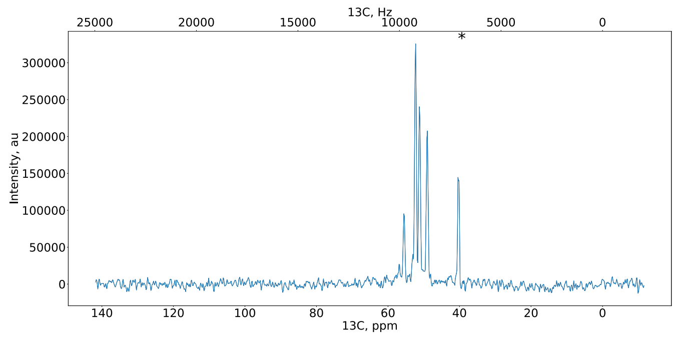

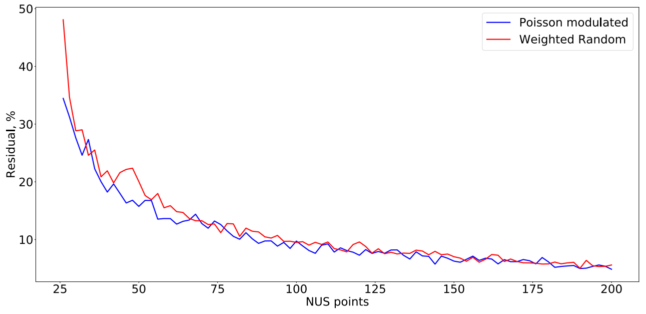

Peak:74

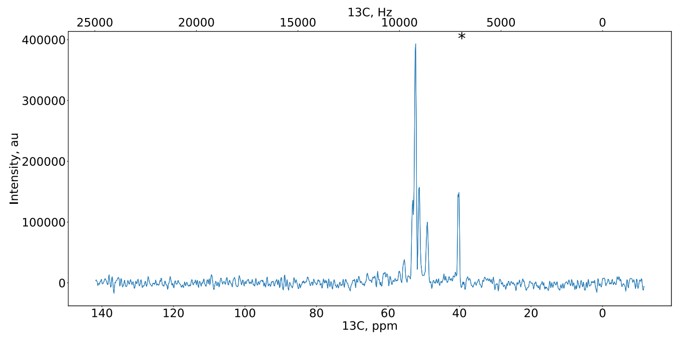

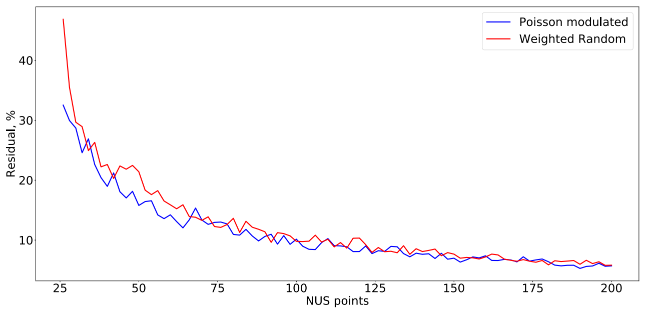

Peak:75

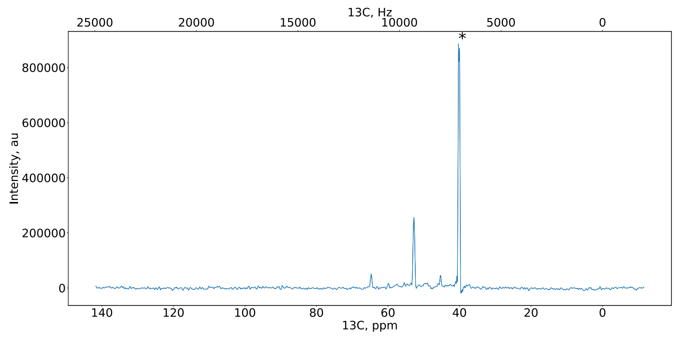

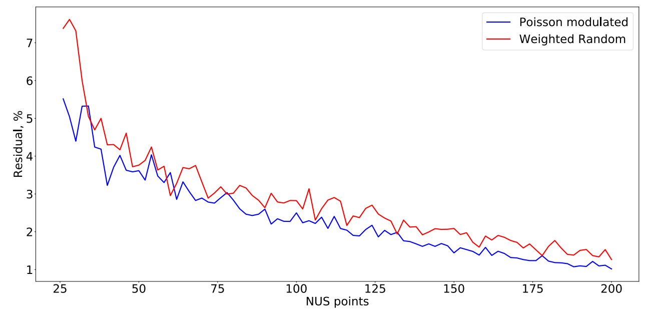

Peak:76

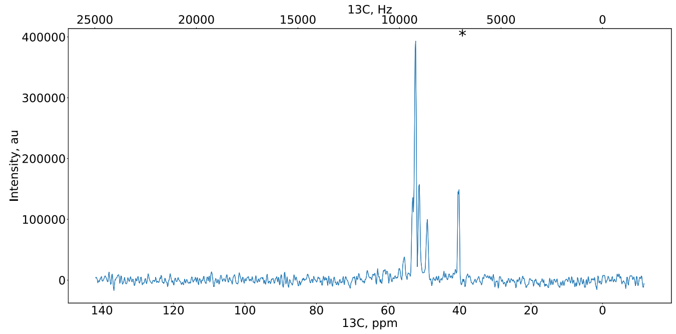

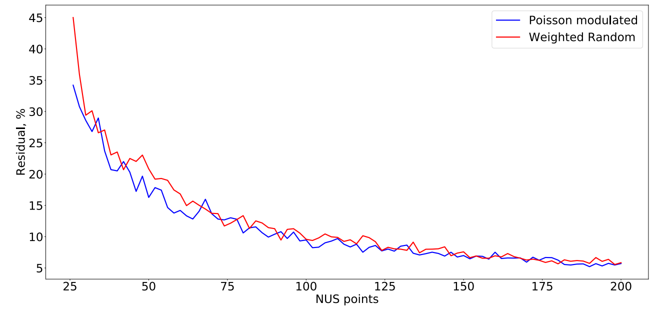

Peak:77

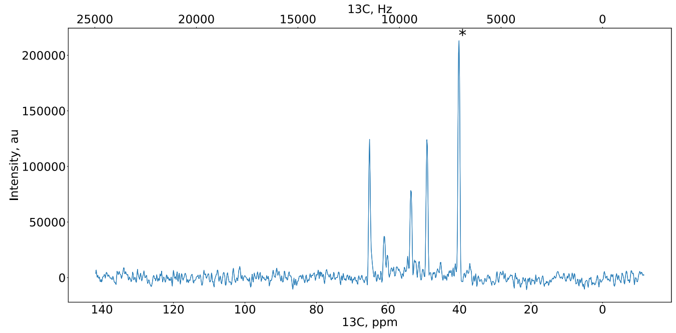

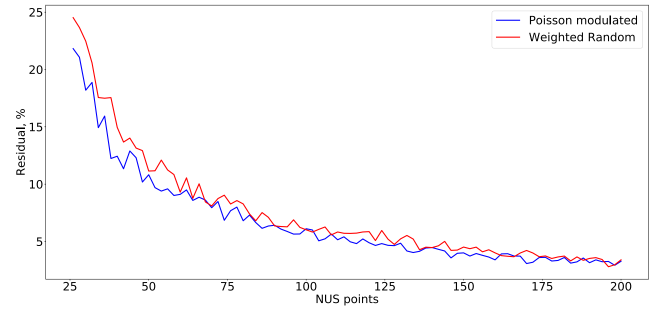

Peak:78

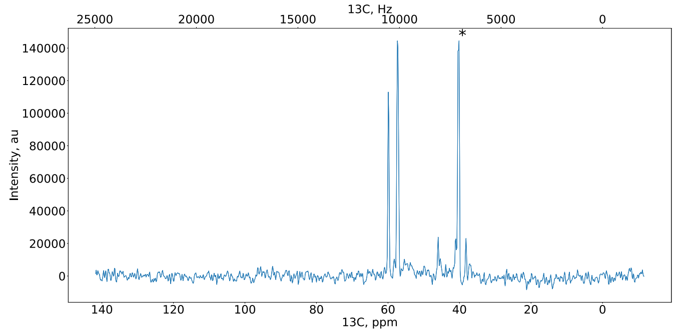

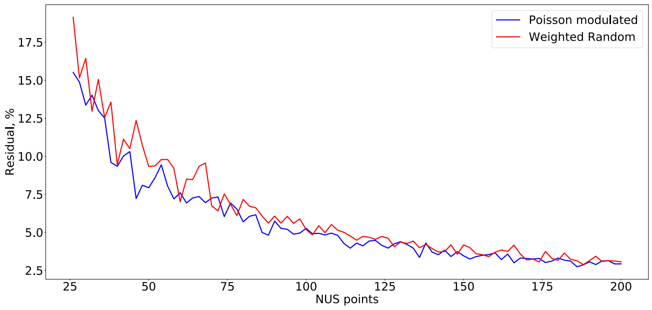

Peak:79

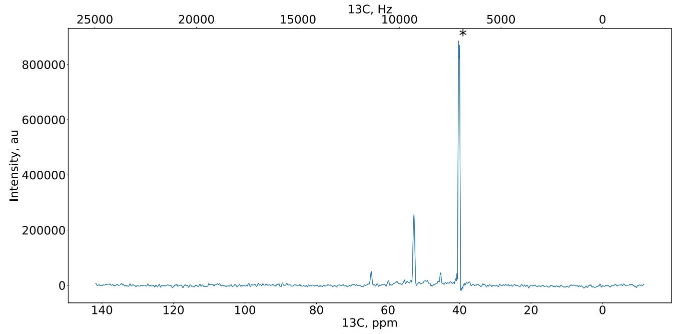

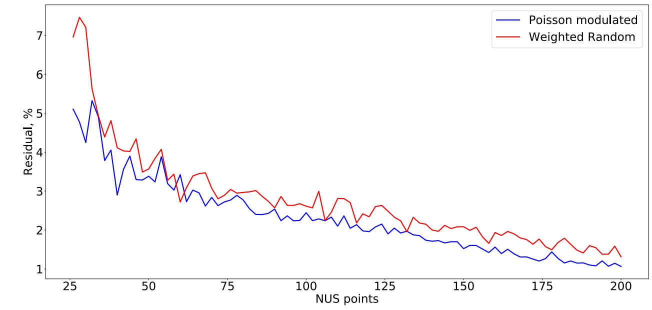

Peak:80

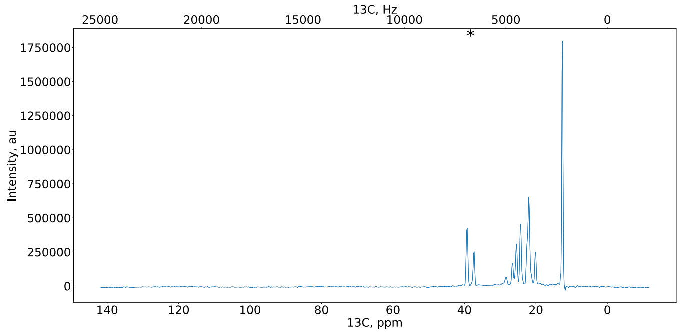

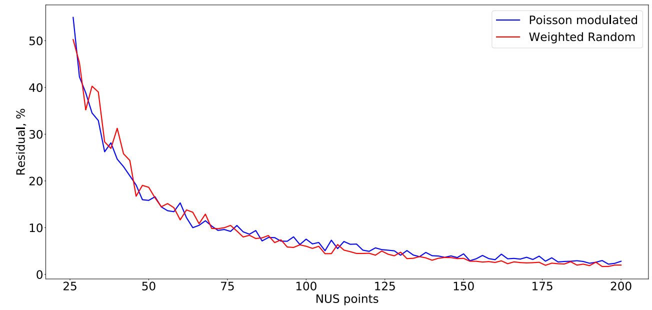

Peak:81

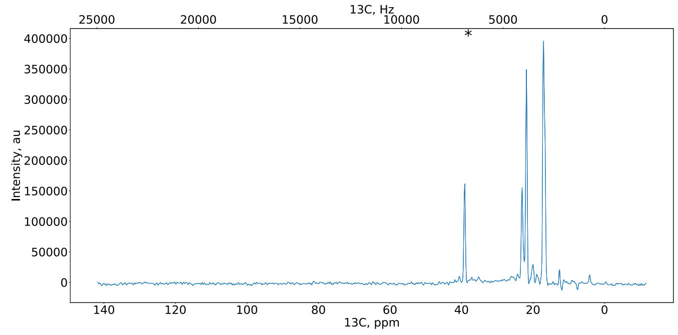

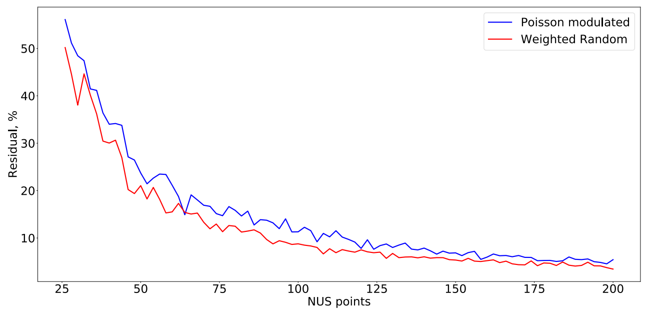

Peak:82

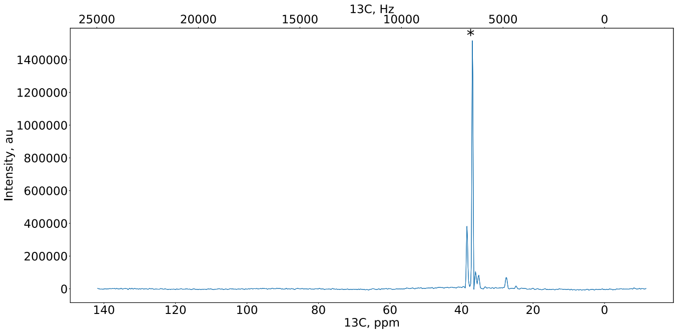

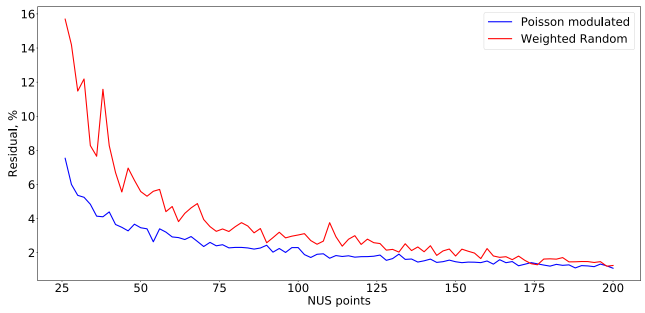

Peak:83

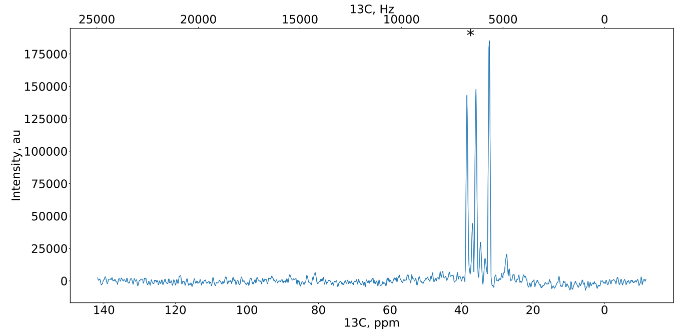

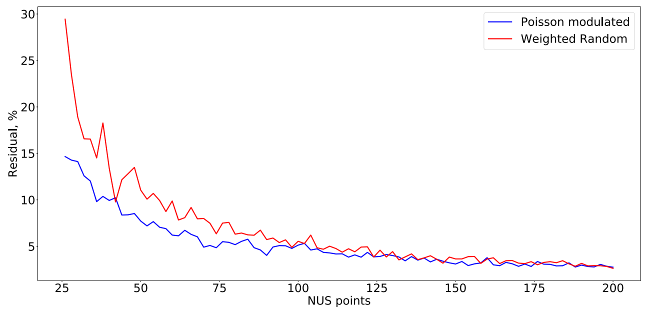

Peak:84

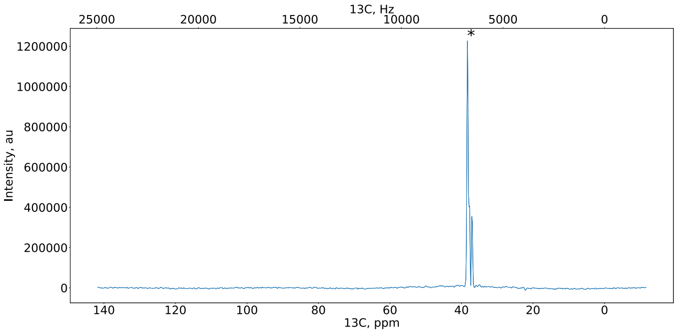

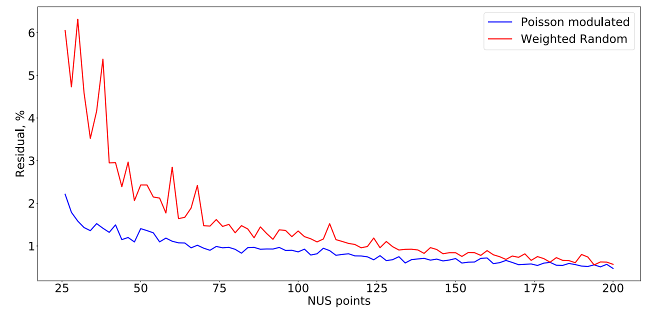

Peak:85

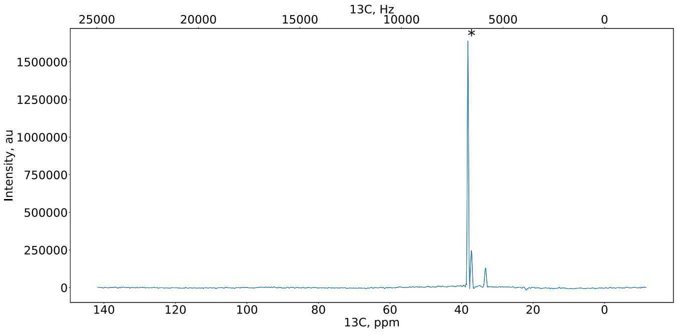

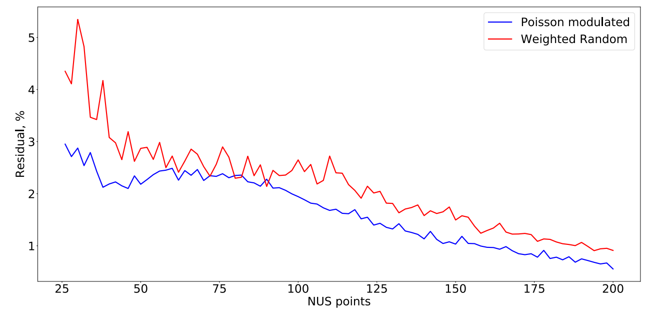

Peak:86

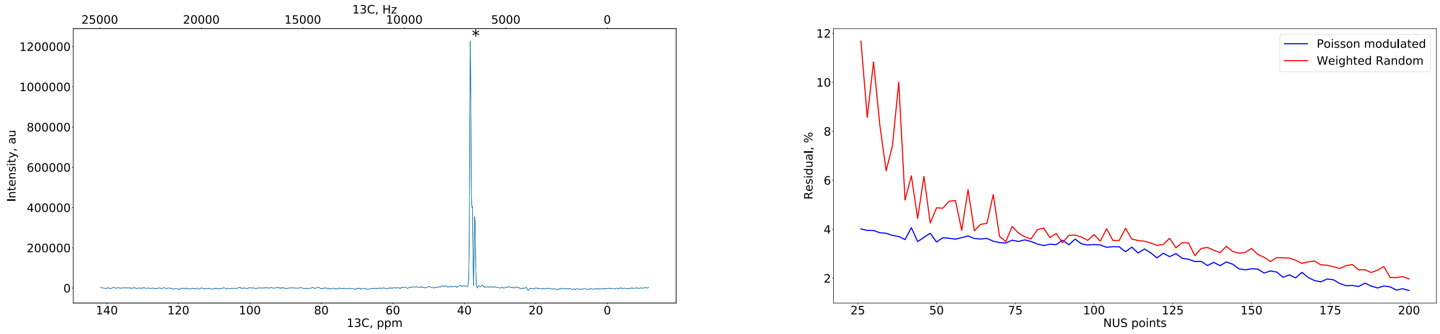

Peak:87

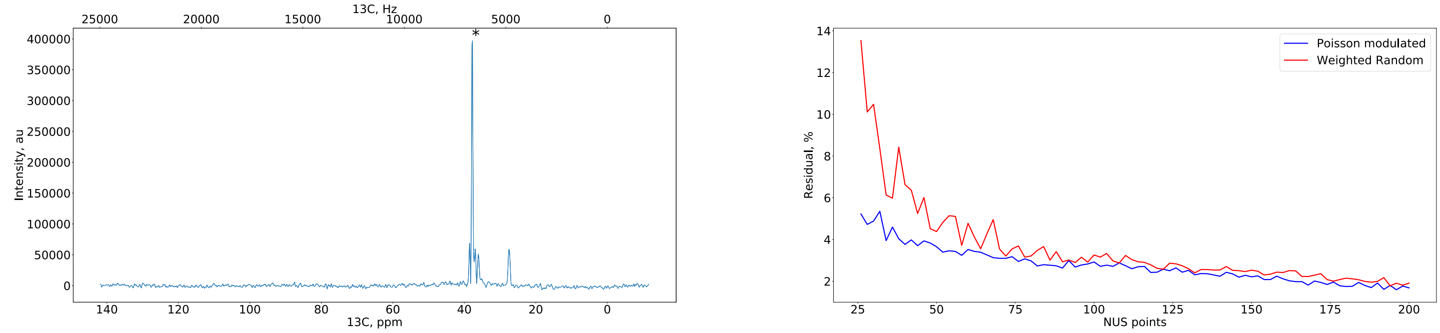

Peak:88

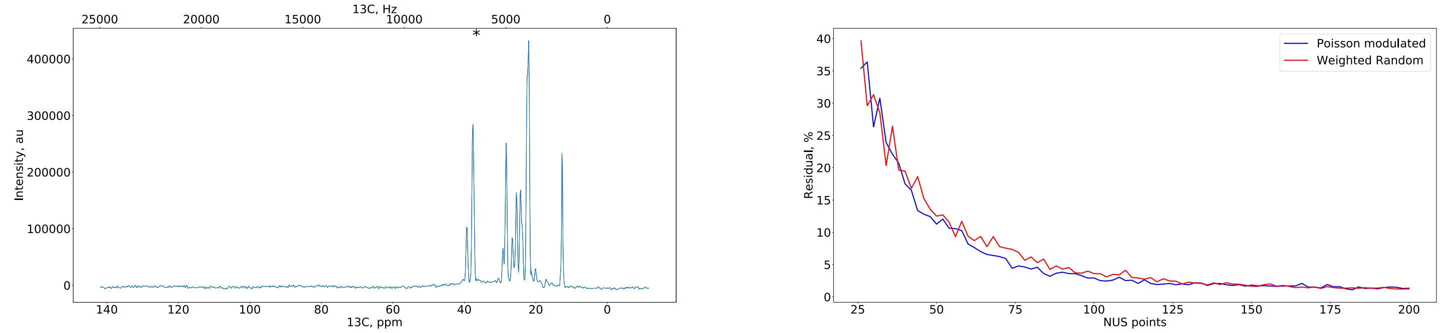

Peak:89

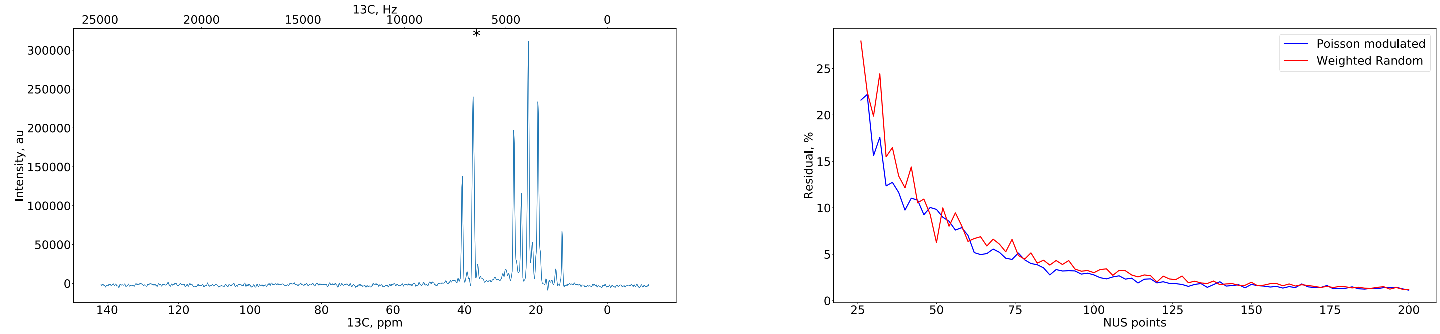

Peak:90

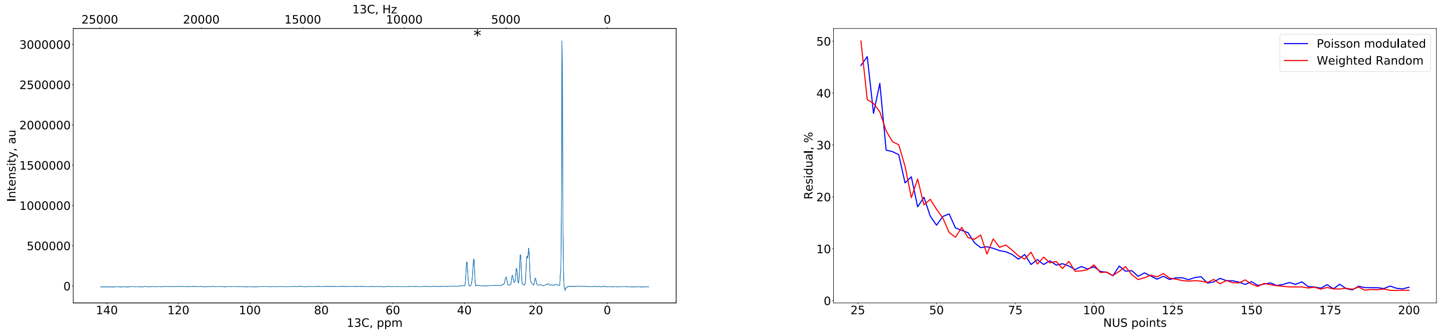

Peak:91

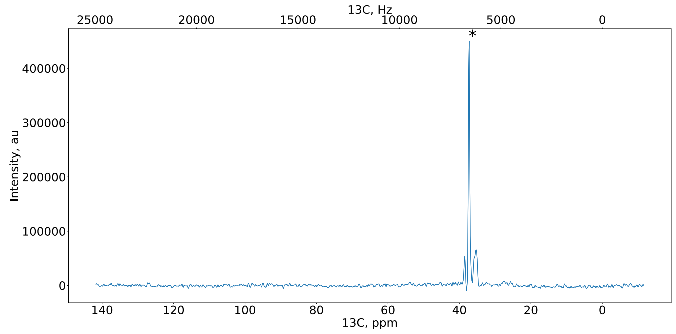

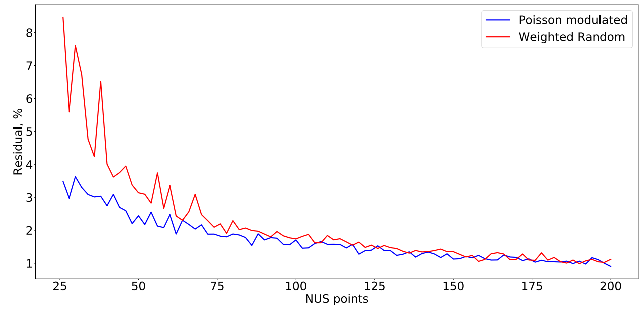

Peak:92

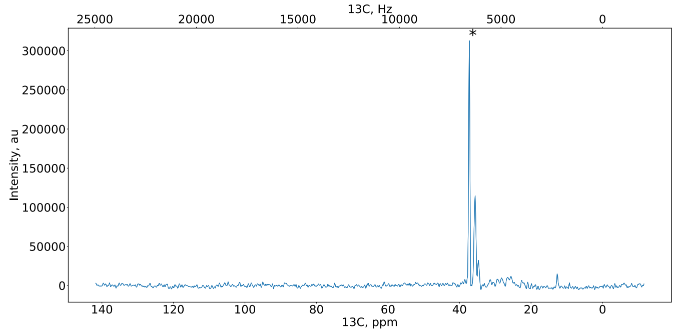

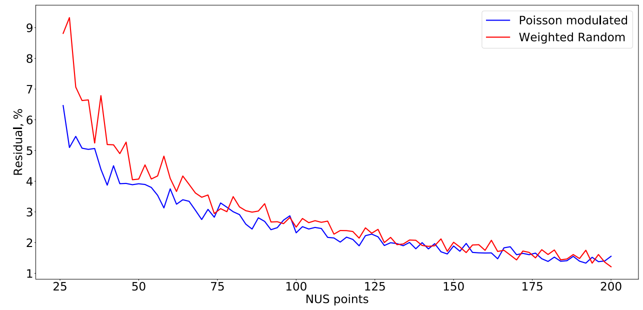

Peak:93

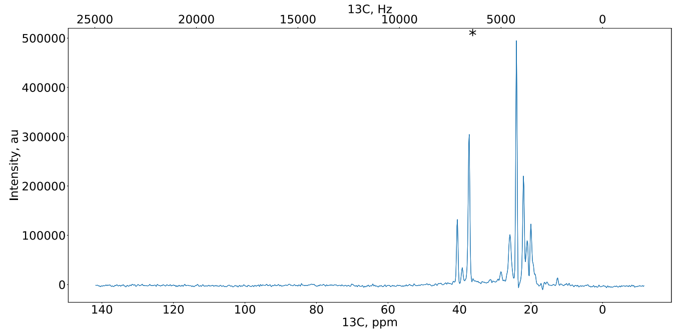

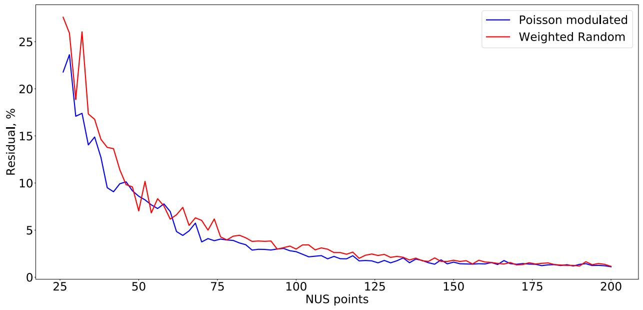

Peak:94

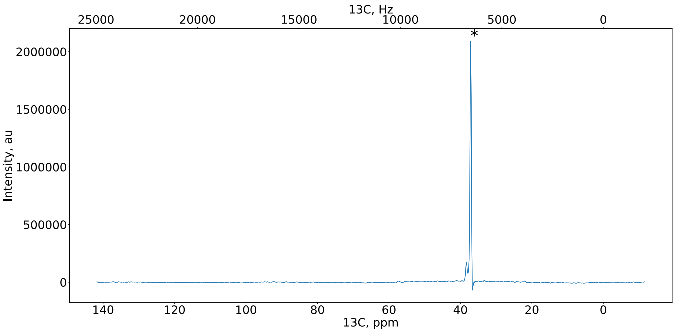

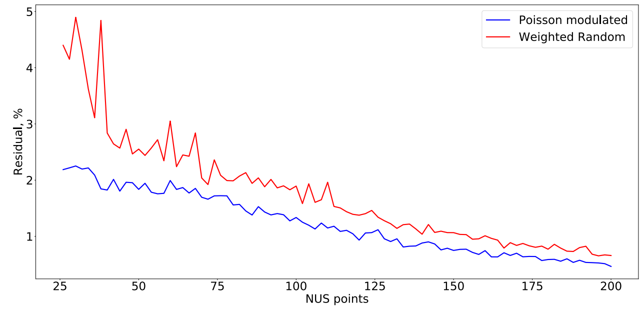

Peak:95

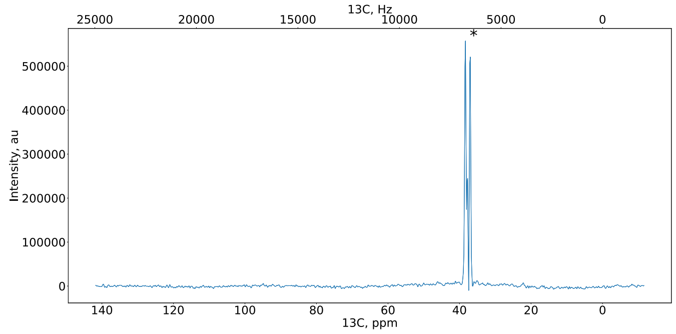

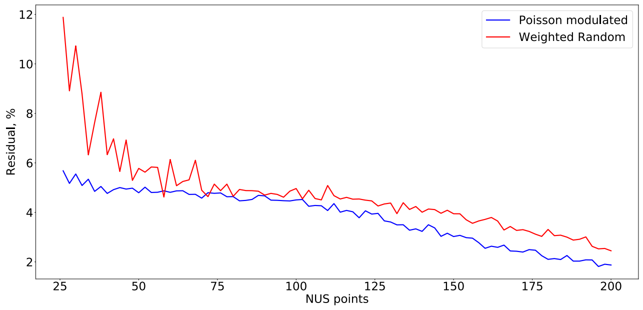

Peak:96

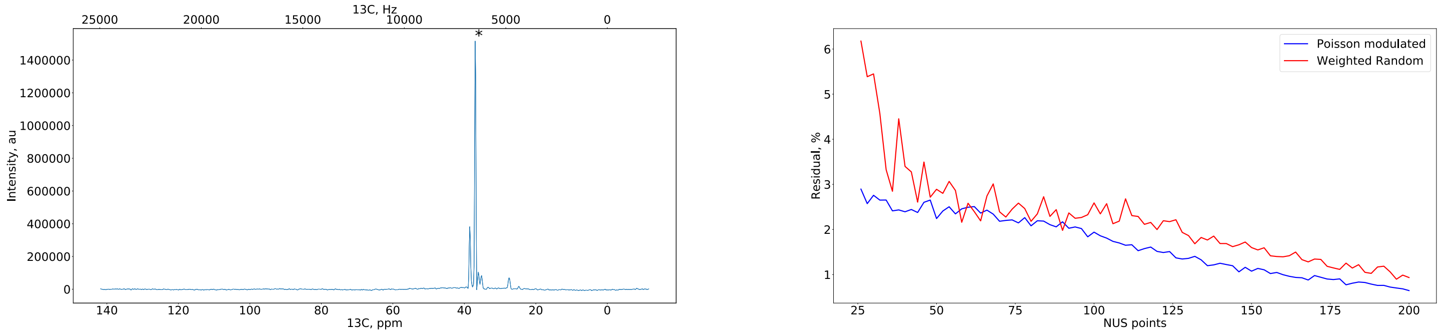

Peak:97

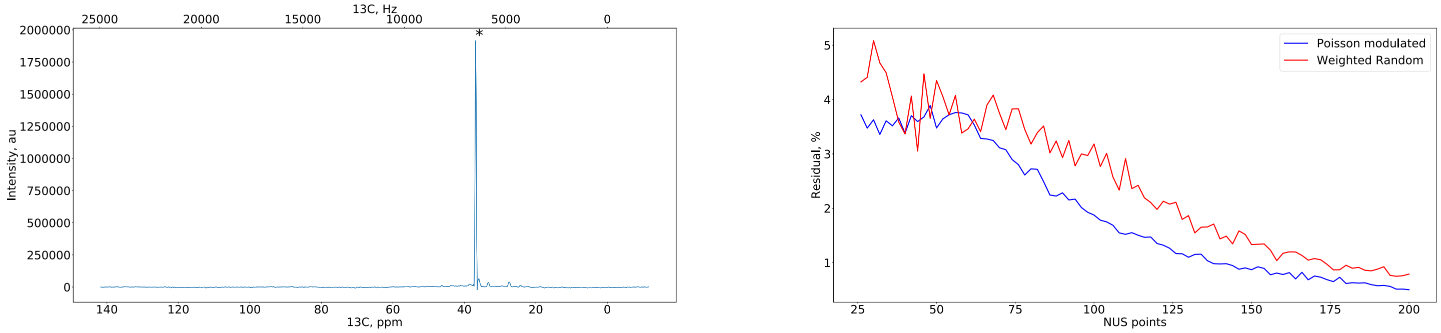

Peak:98

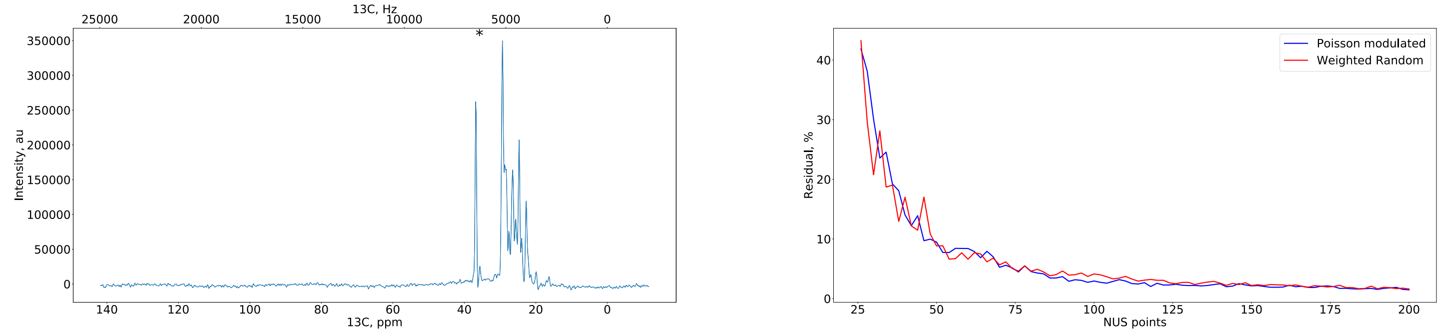

Peak:99

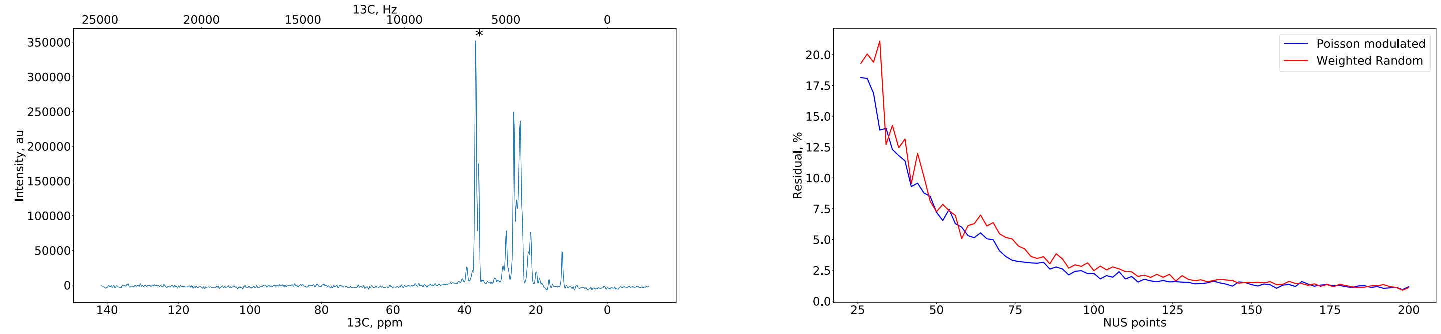

Peak:100

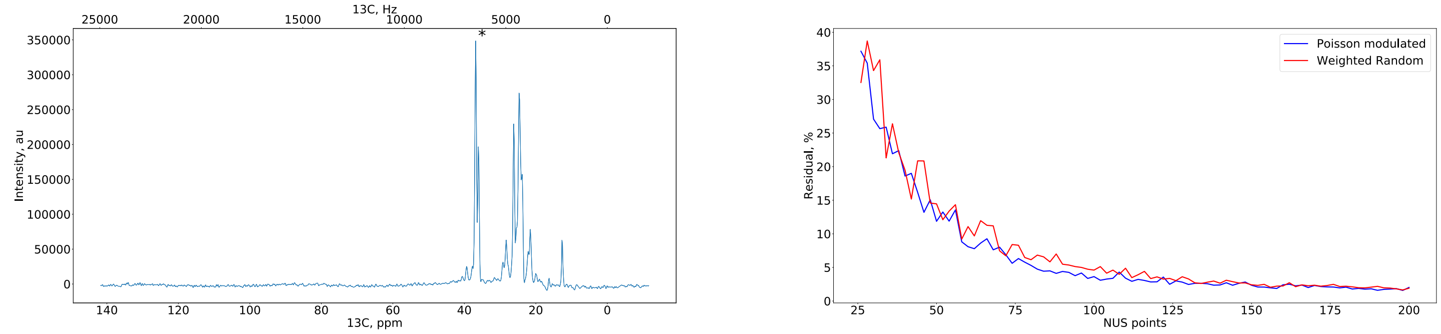

Peak:101

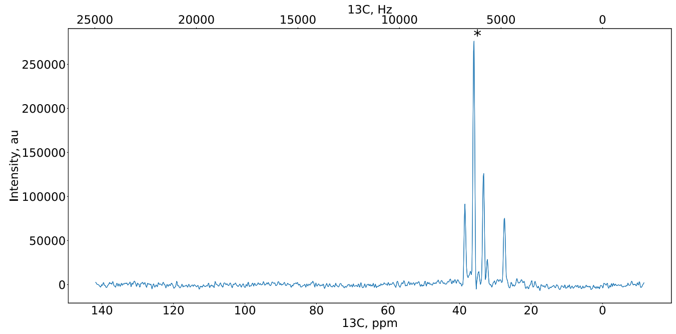

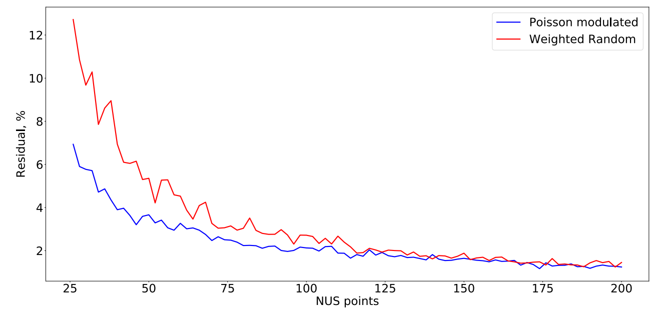

Peak:102

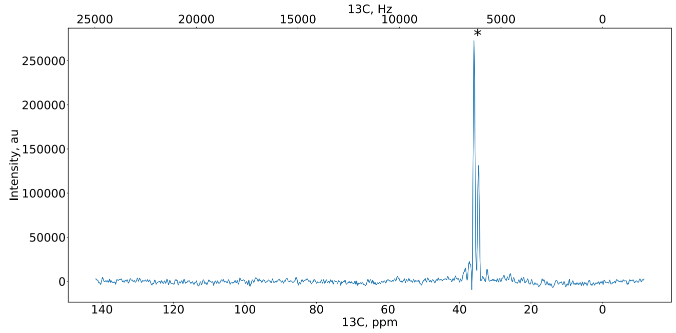

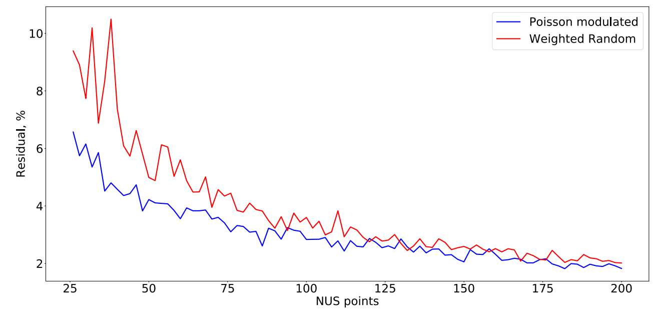

Peak:103

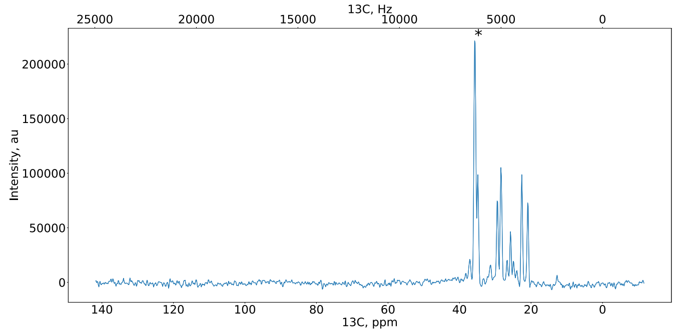

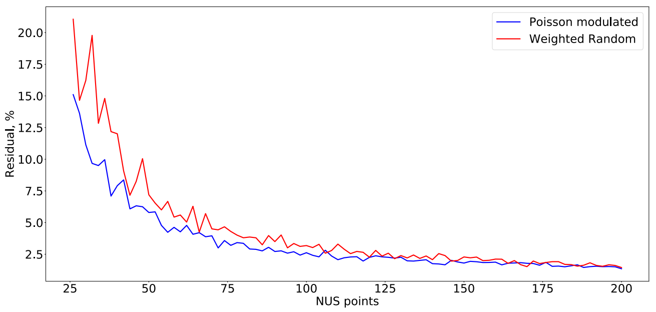

Peak:104

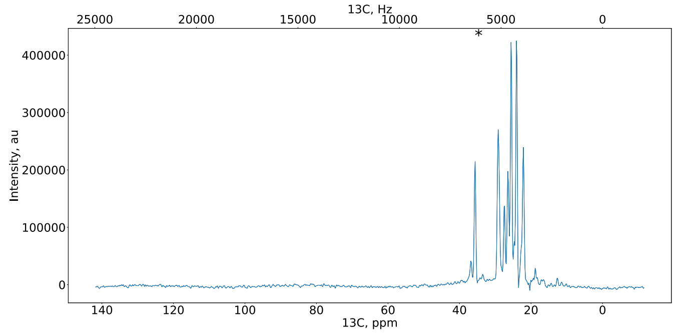

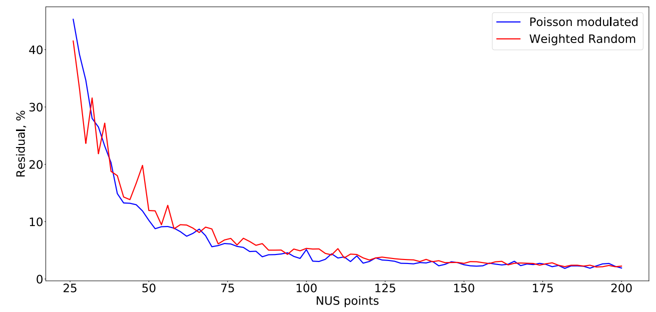

Peak:105

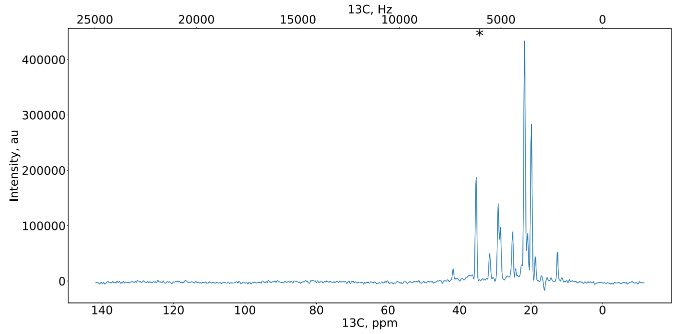

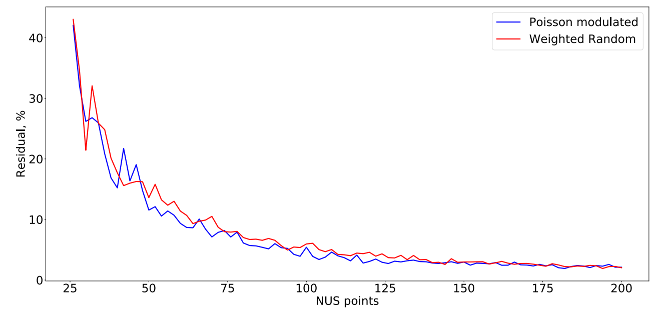

Peak:106

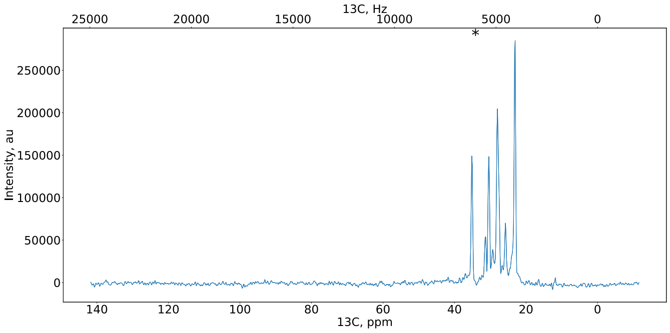

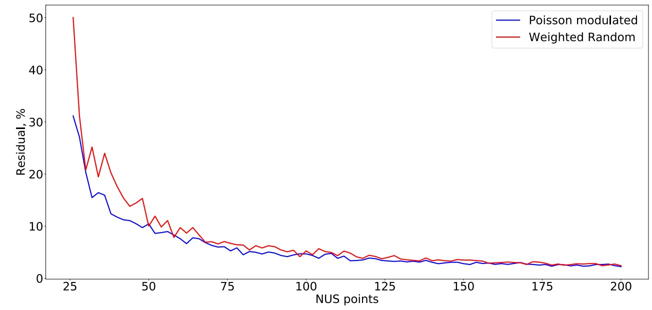

Peak:107

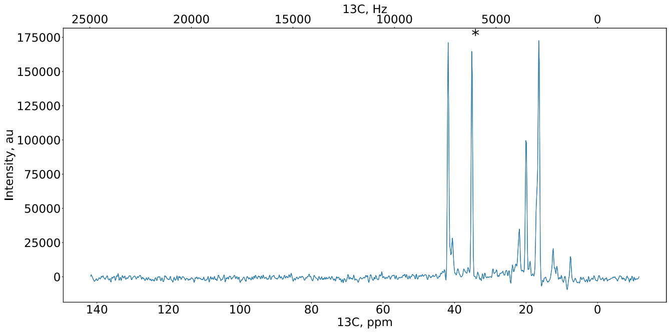

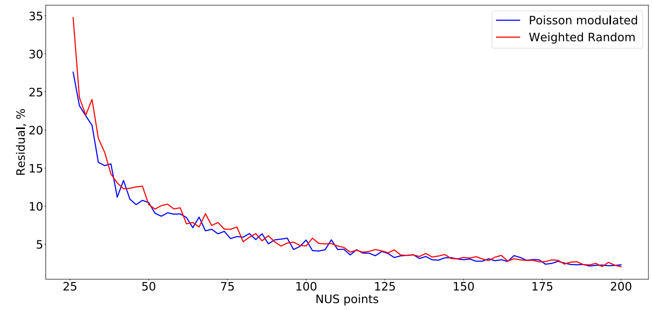

Peak:108

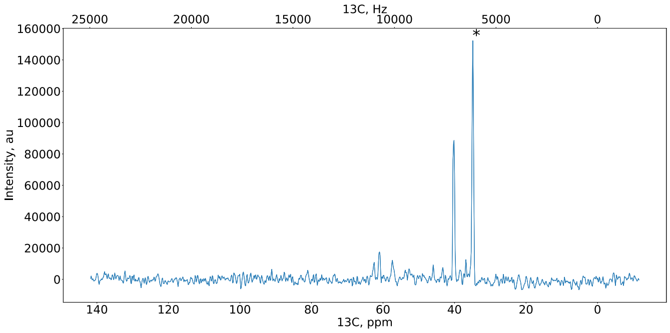

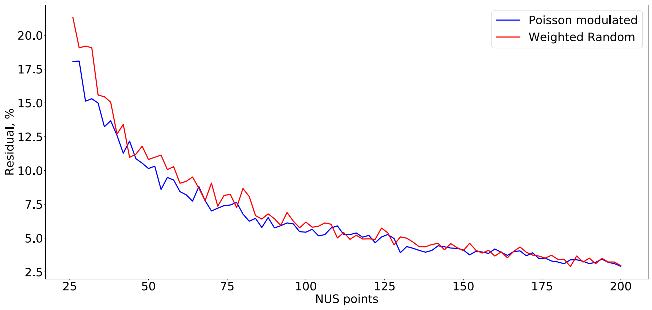

Peak:109

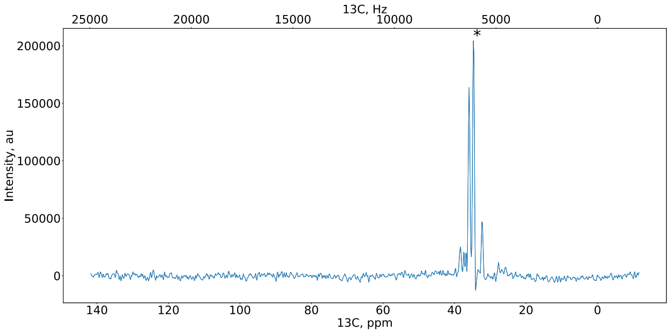

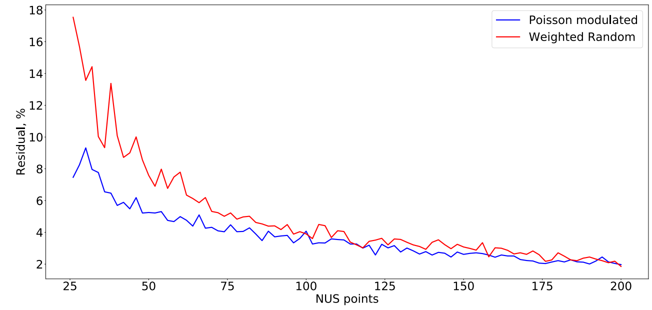

Peak:110

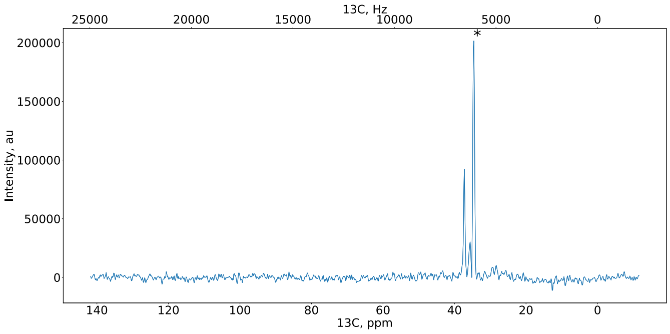

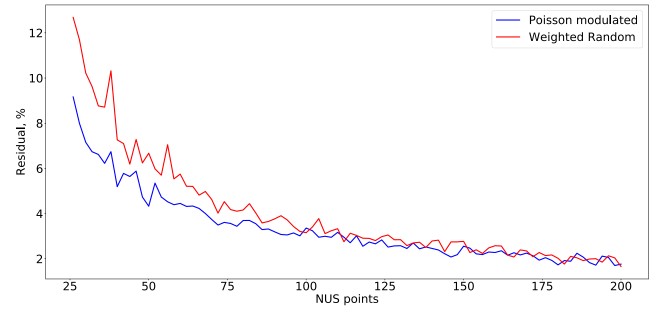

Peak:111

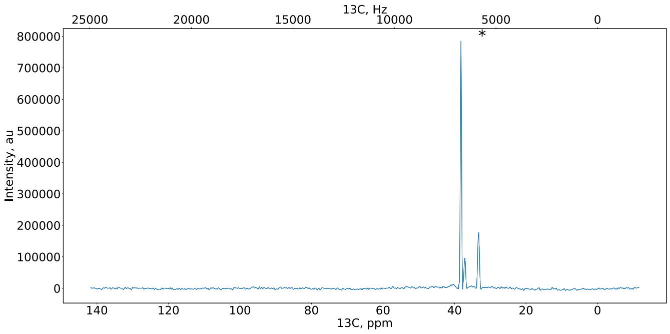

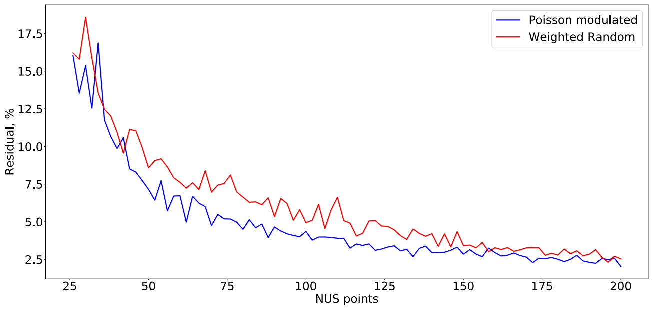

Peak:112

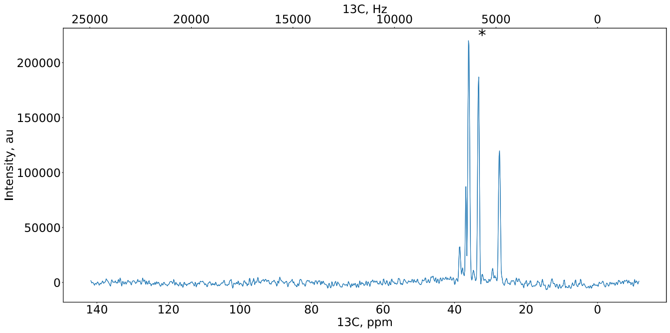

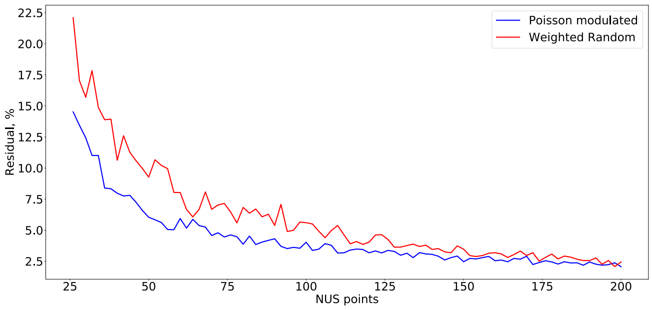

Peak:113

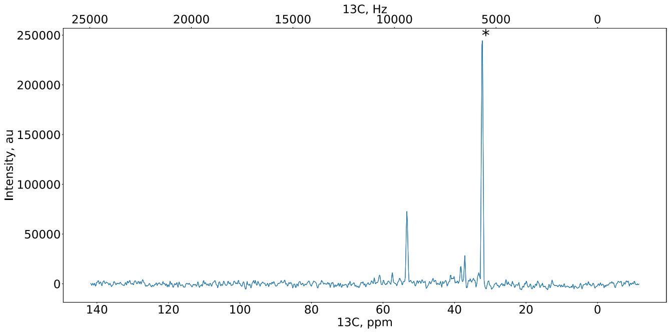

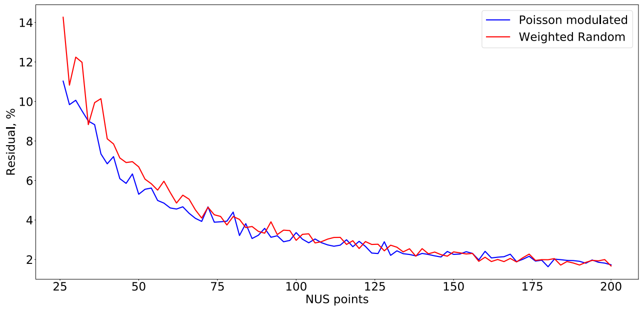

Peak:114

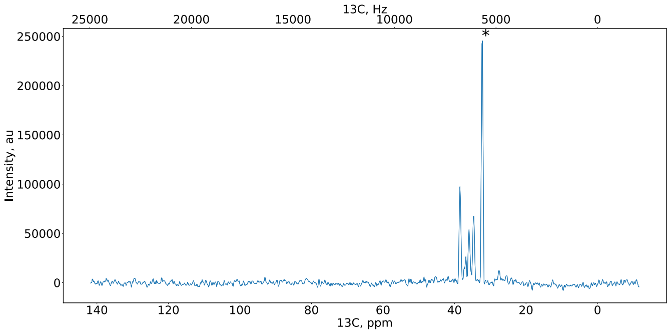

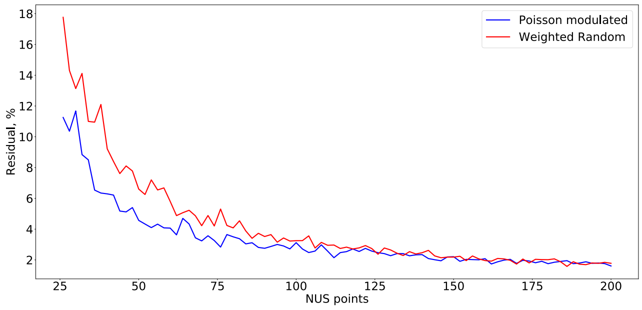

Peak:115

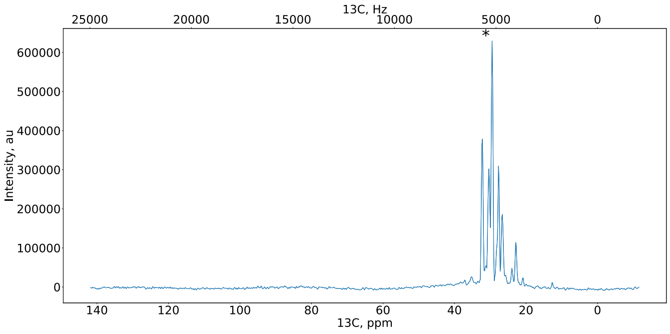

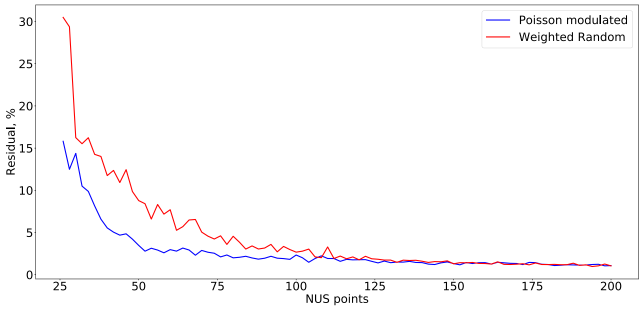

Peak:116

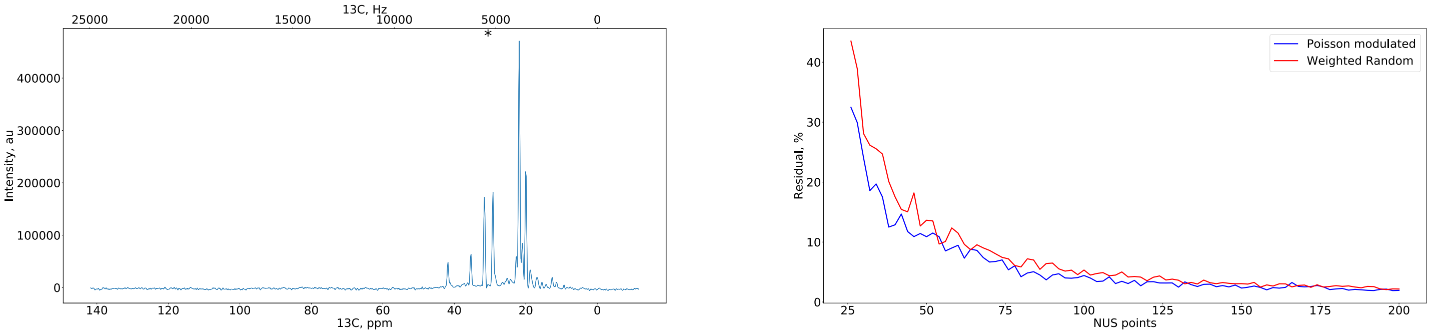

Peak:117

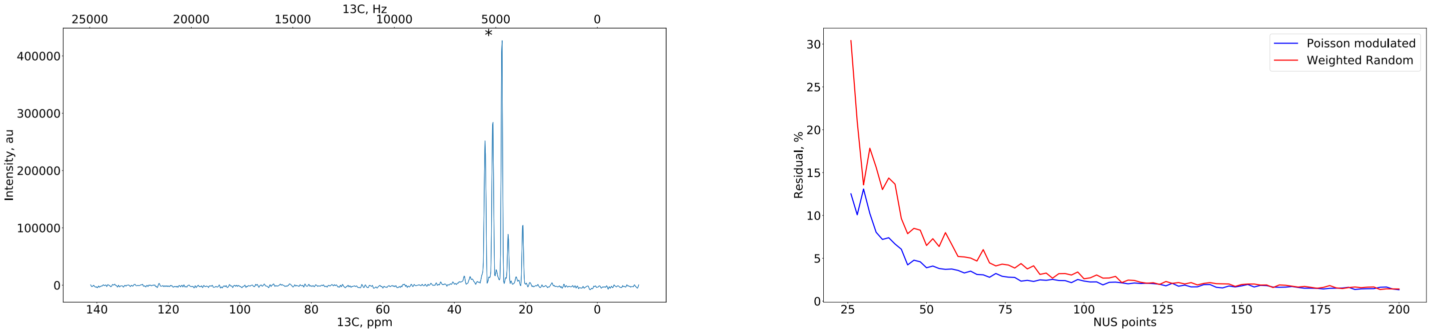

Peak:118

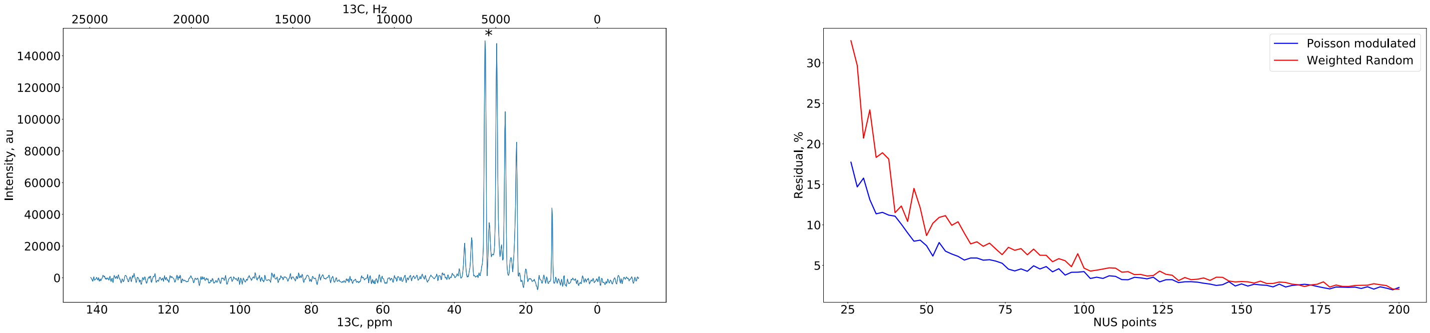

Peak:119

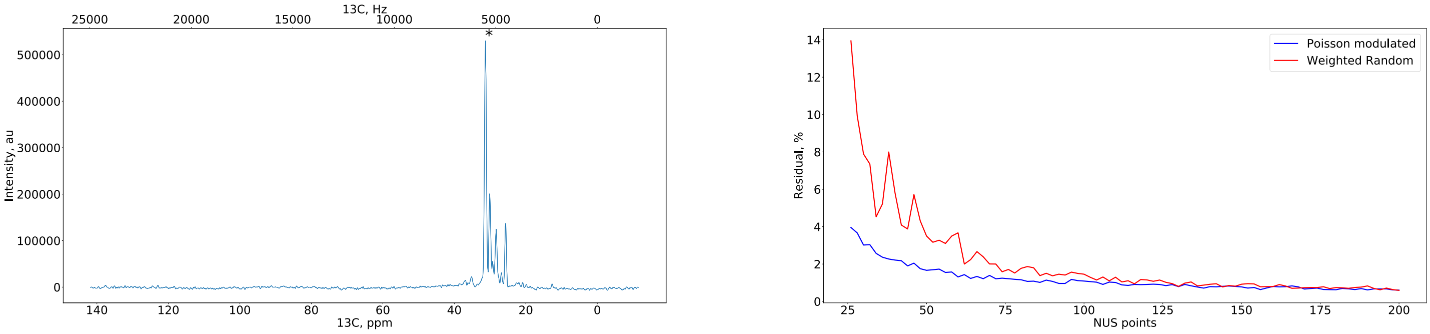

Peak:120

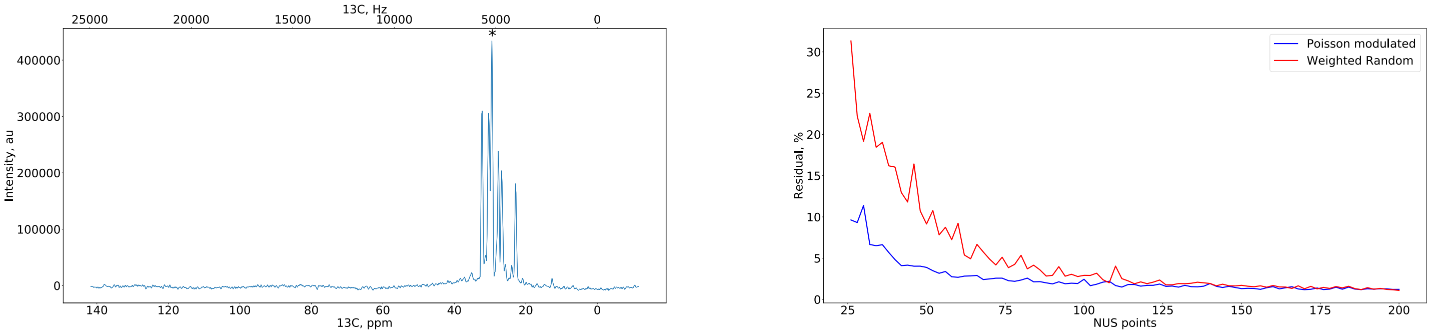

Peak:121

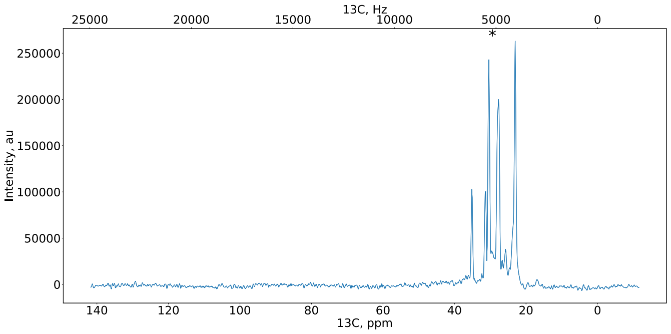

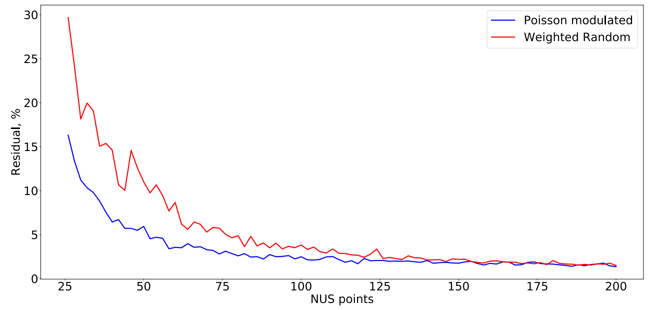

Peak:122

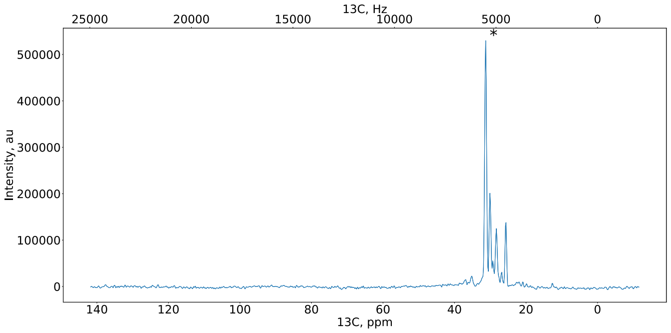

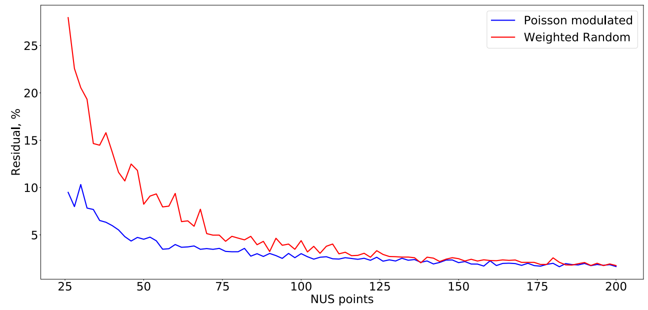

Peak:123

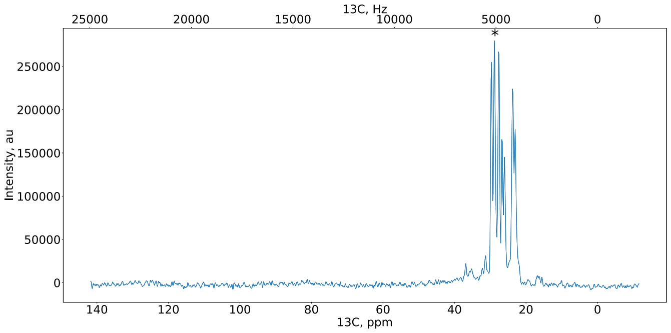

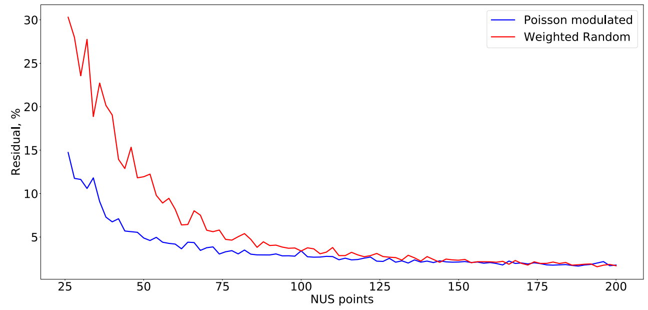

Peak:124

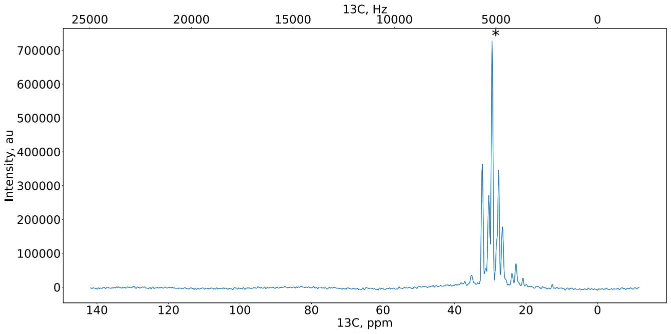

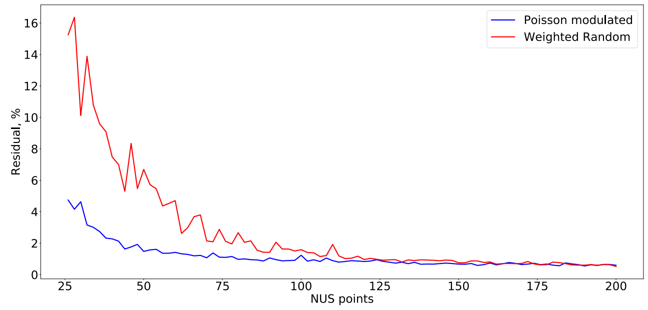

Peak:125

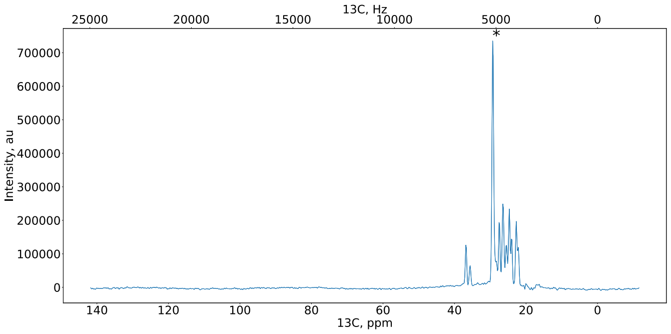

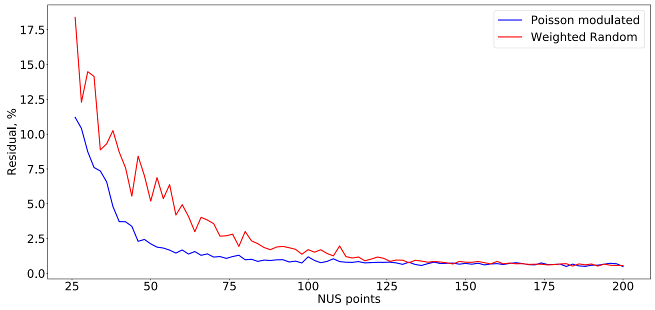

Peak:126

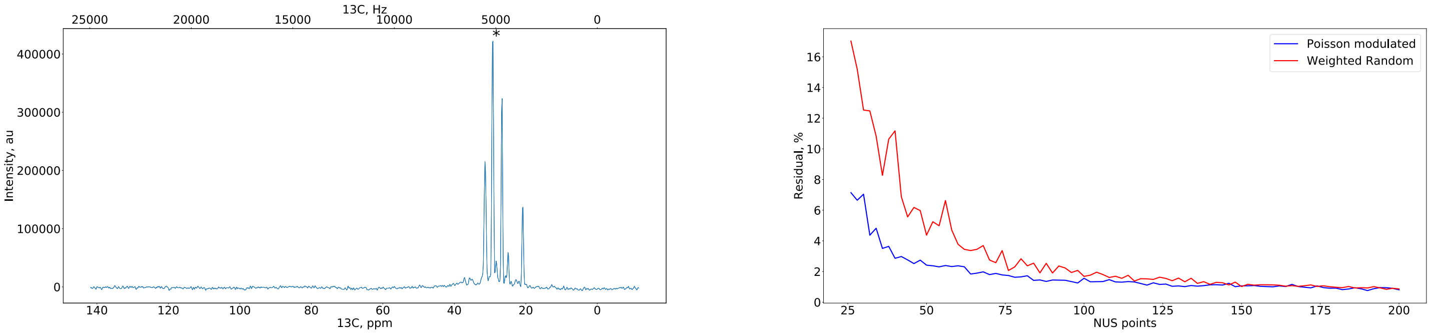

Peak:127

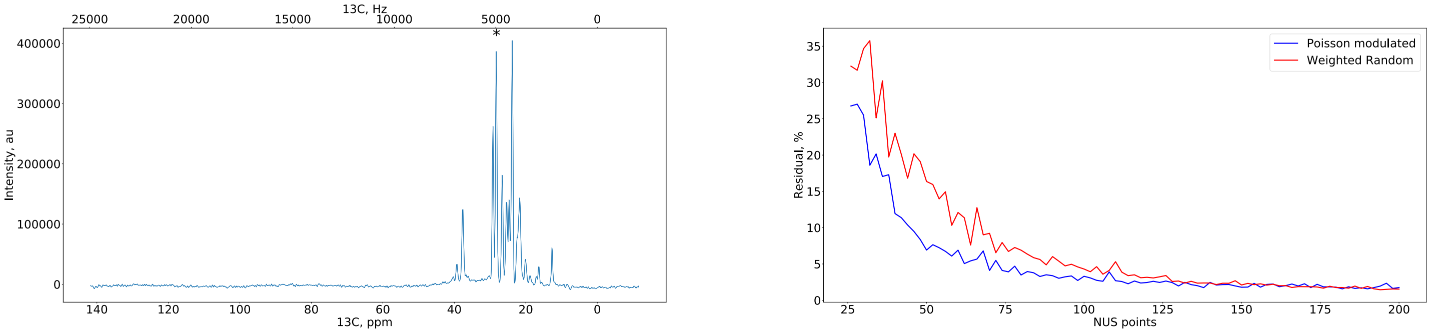

Peak:128

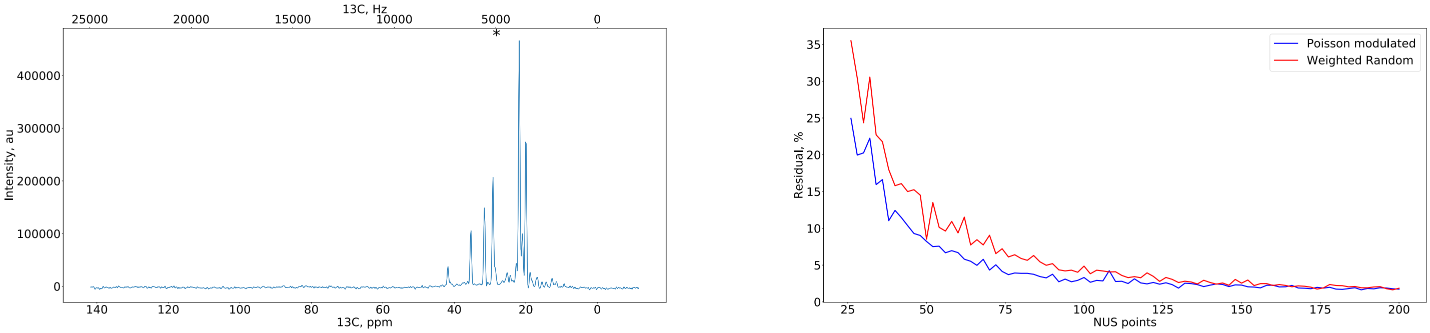

Peak:129

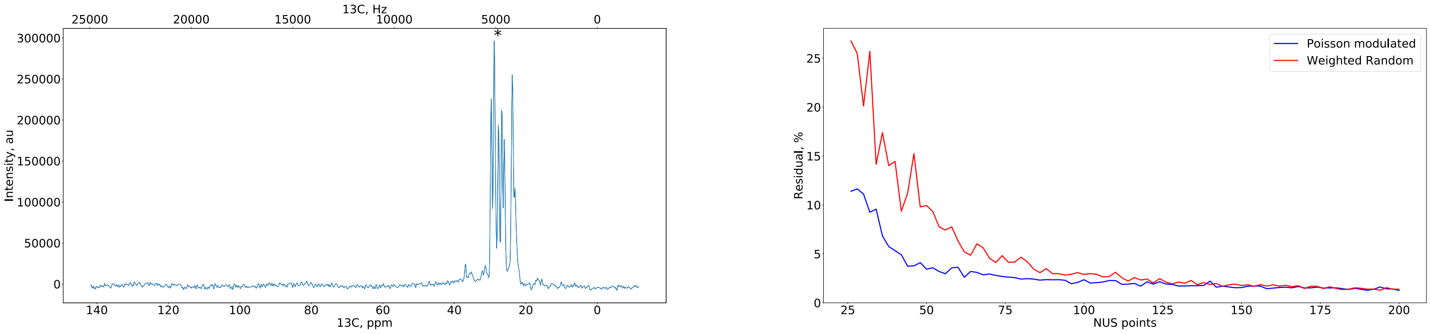

Peak:130

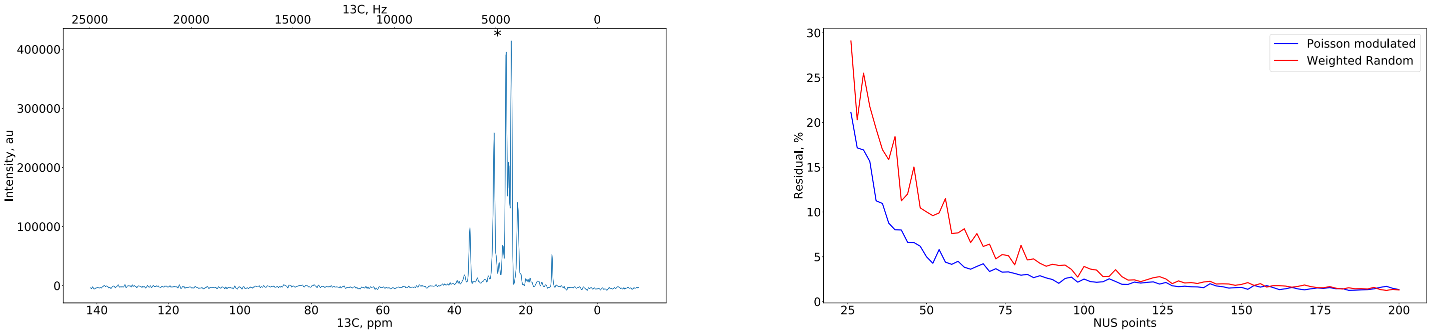

Peak:131

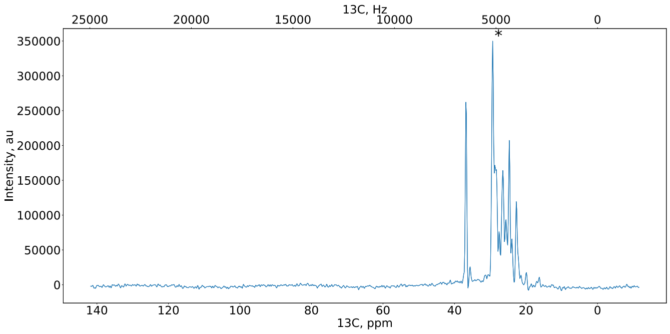

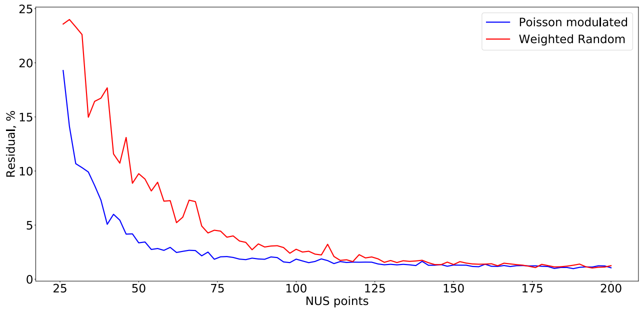

Peak:132

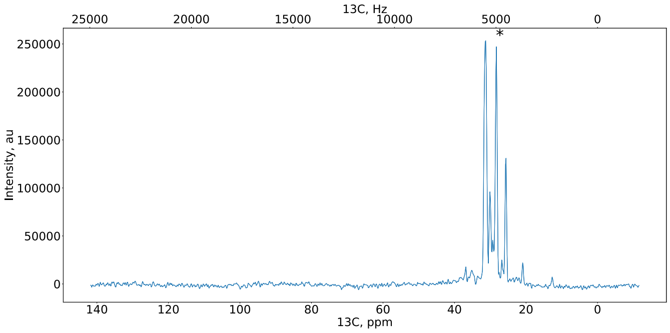

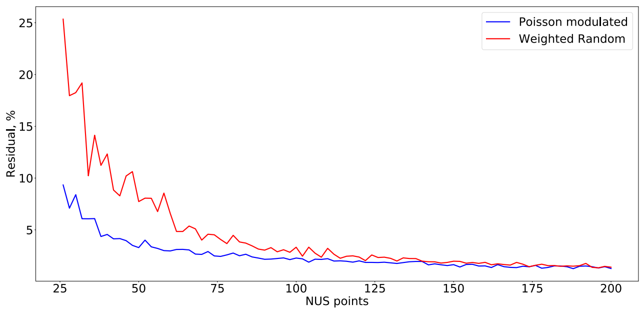

Peak:133

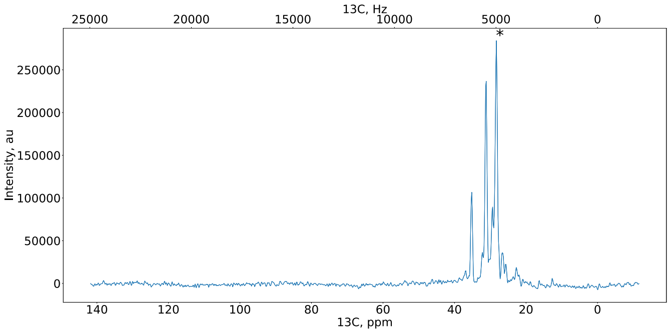

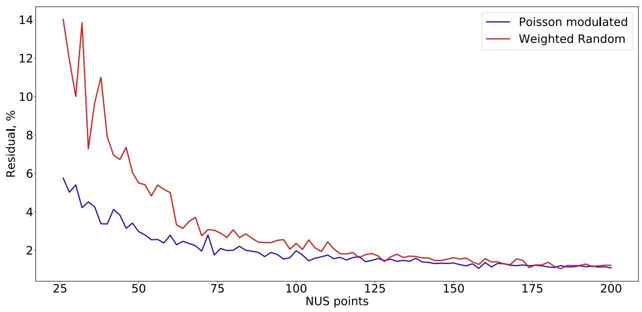

Peak:134

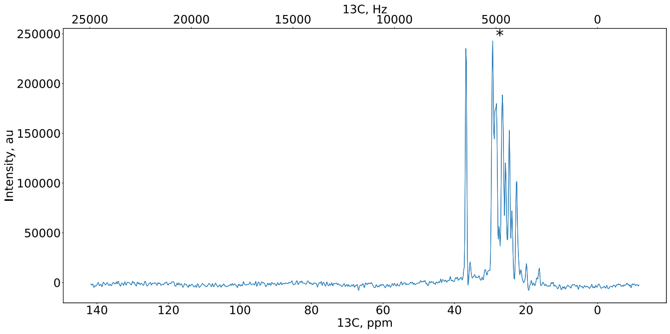

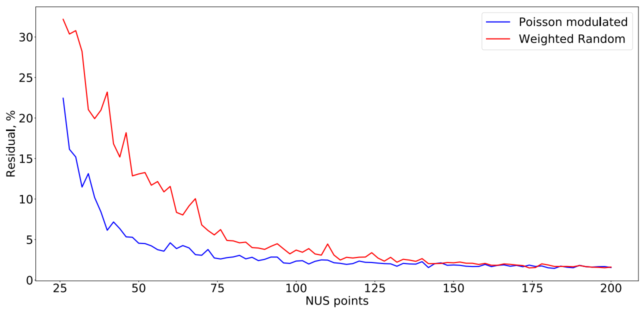

Peak:135

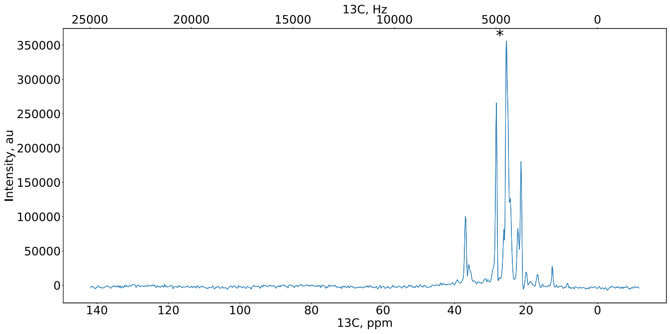

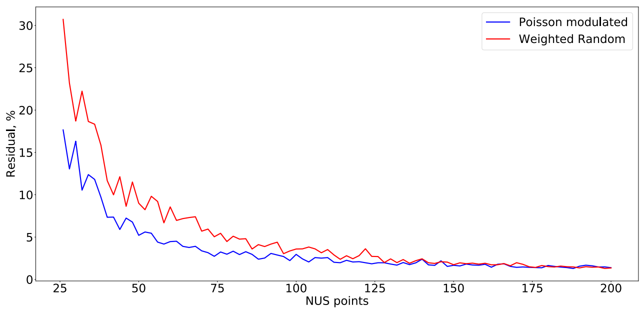

Peak:136

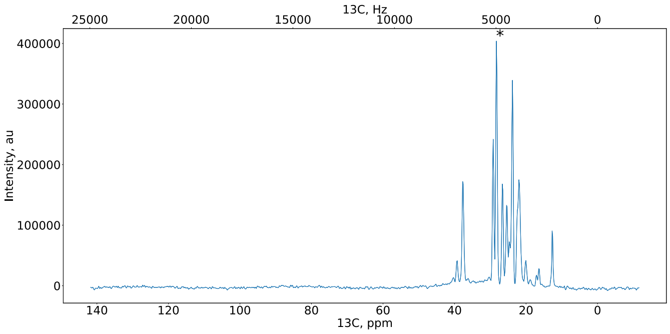

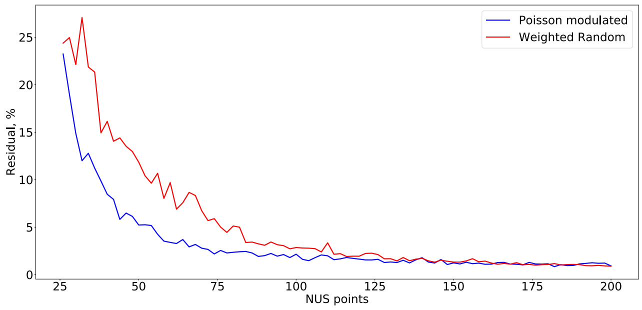

Peak:137

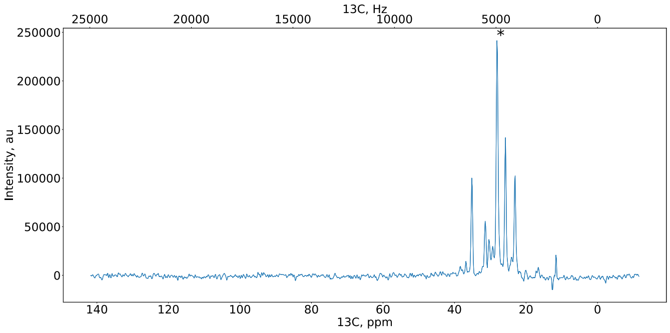

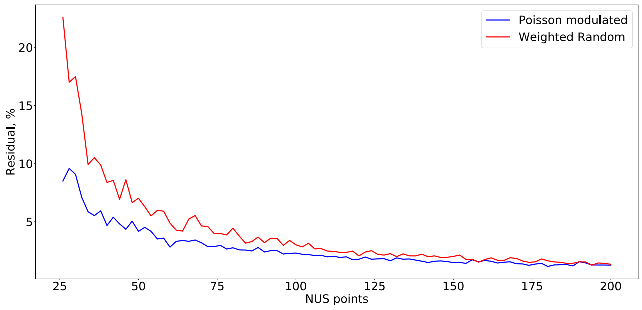

Peak:138

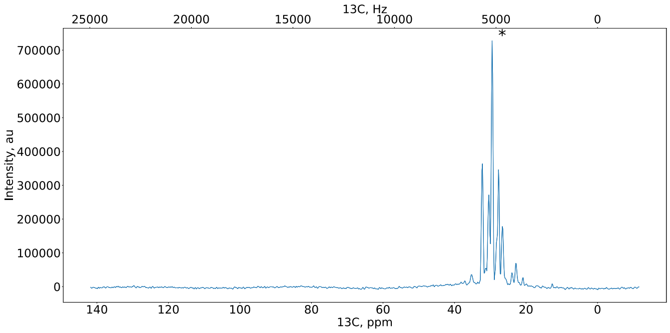

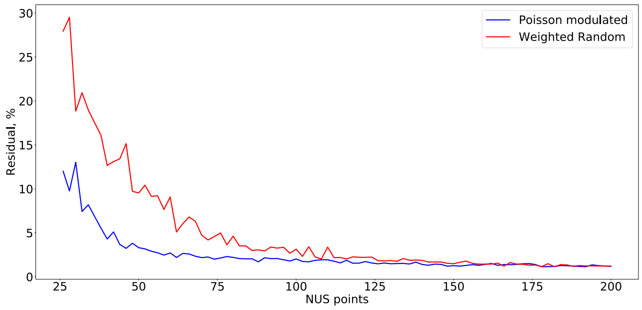

Peak:139

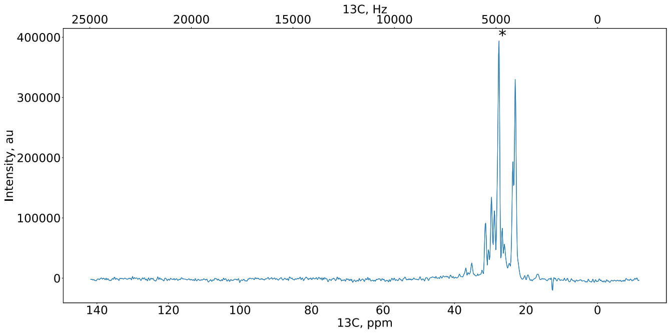

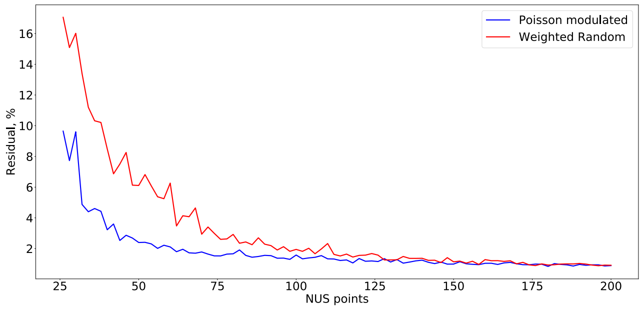

Peak:140

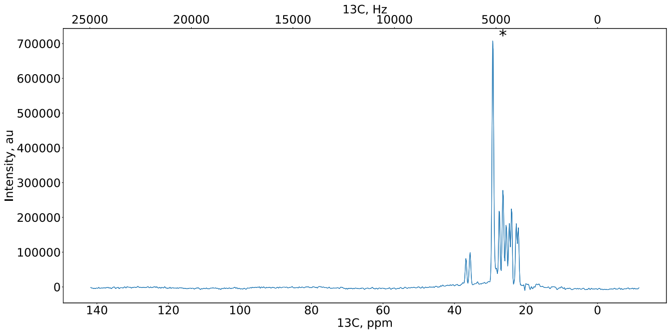

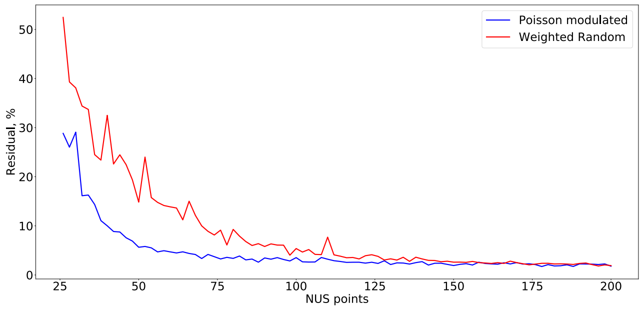

Peak:141

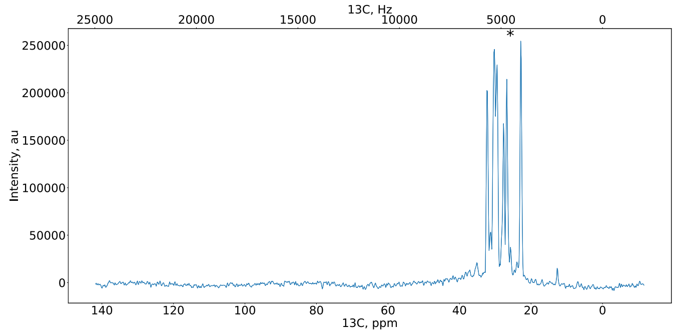

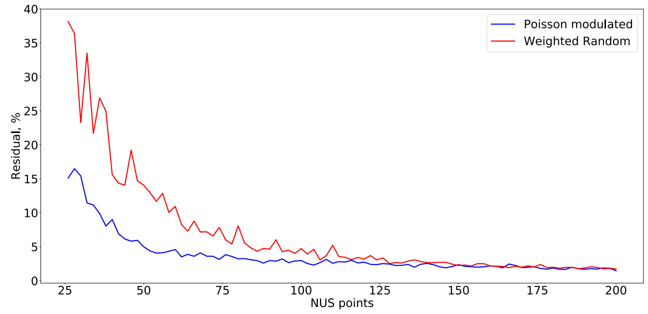

Peak:142

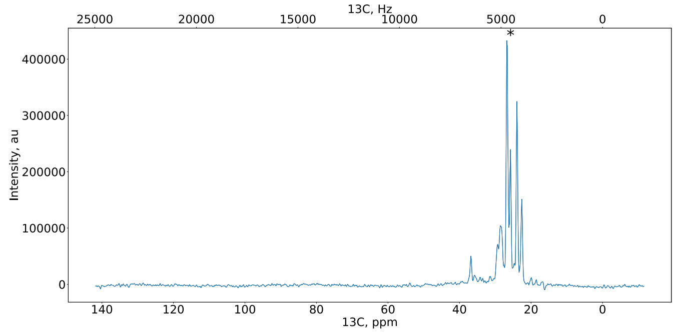

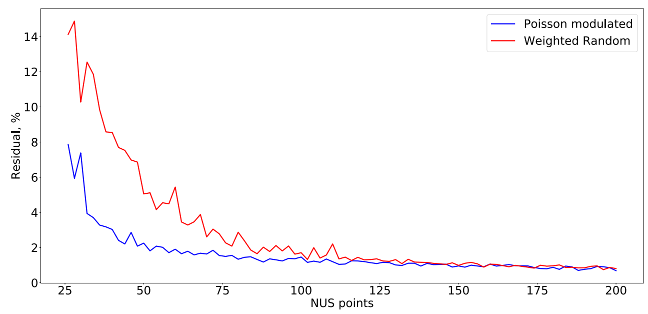

Peak:143

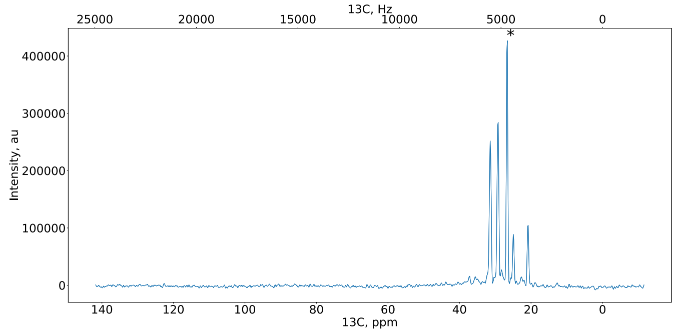

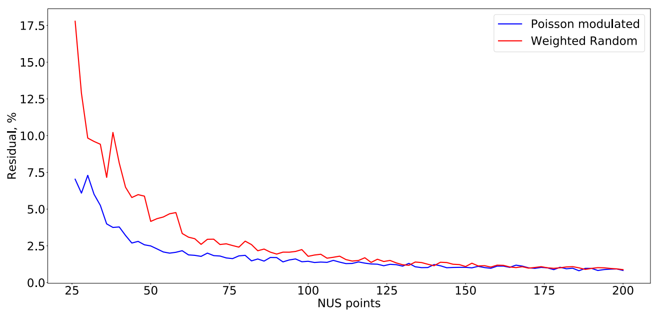

Peak:144

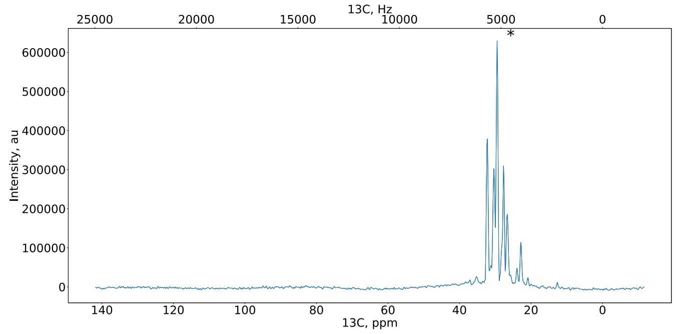

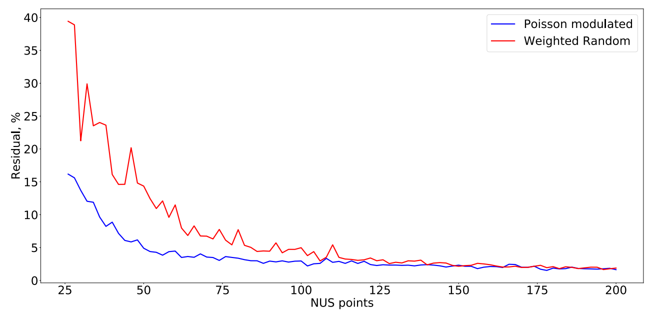

Peak:145

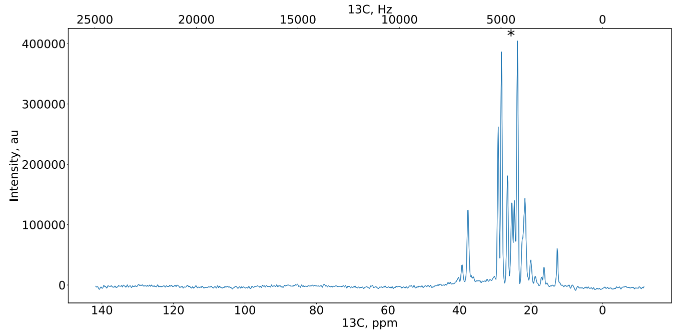

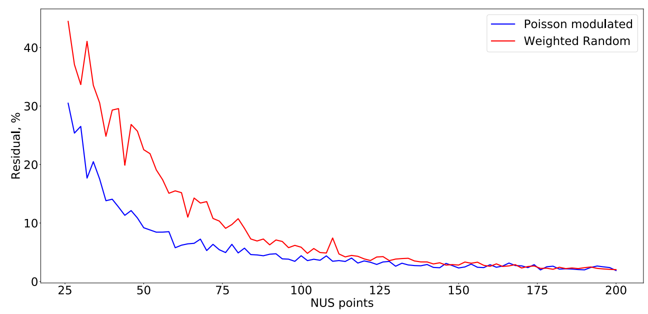

Peak:146

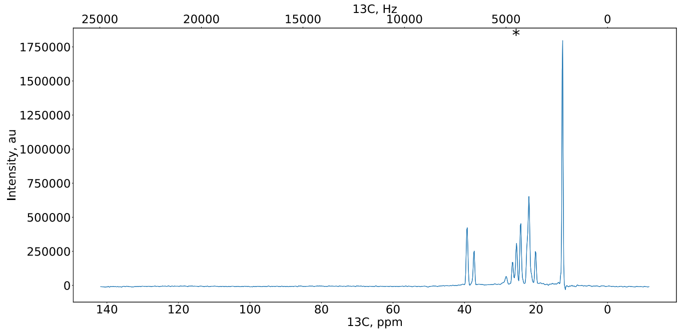

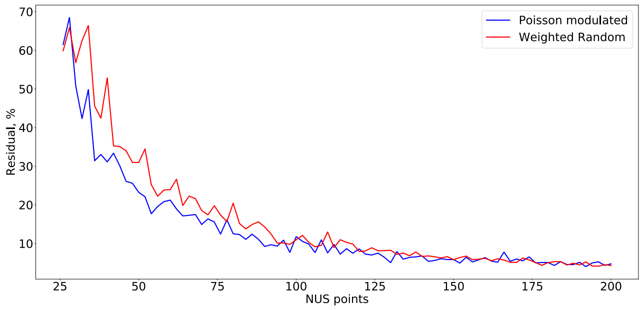

Peak:147

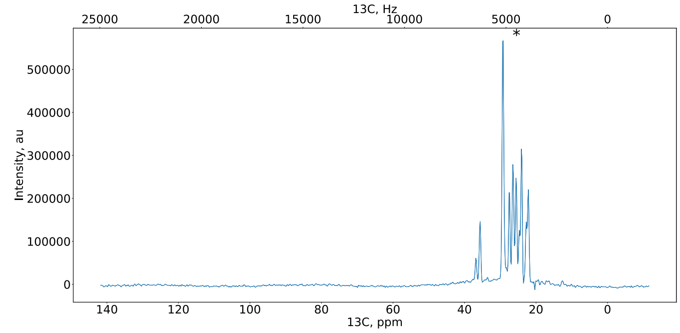

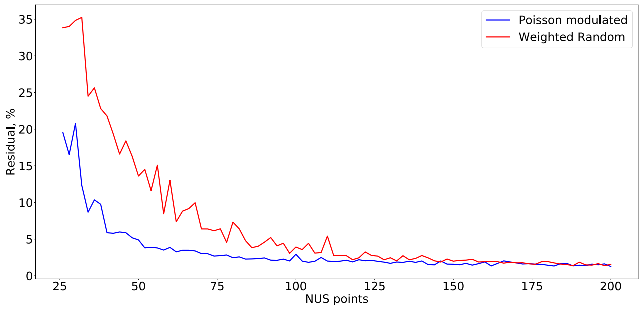

Peak:148

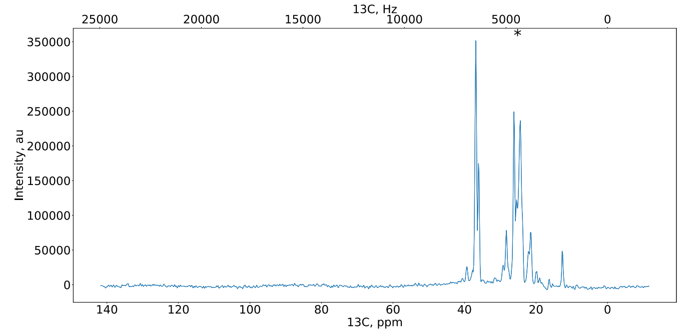

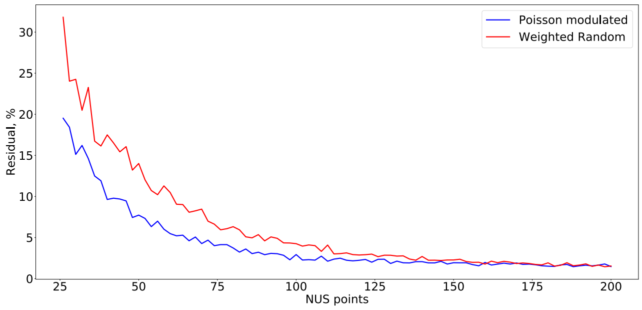

Peak:149

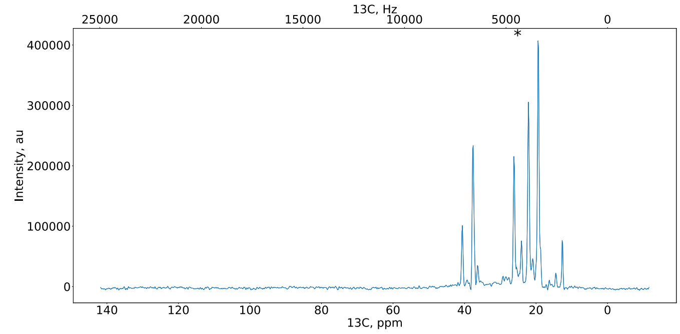

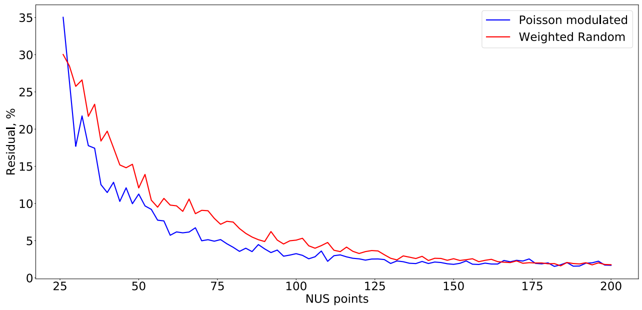

Peak:150

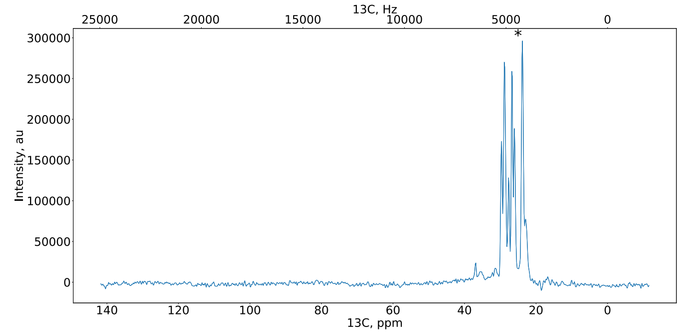

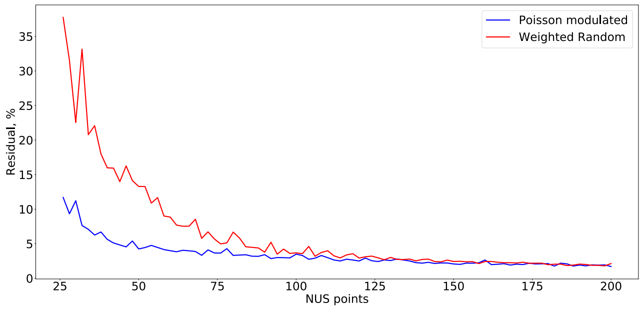

Peak:151

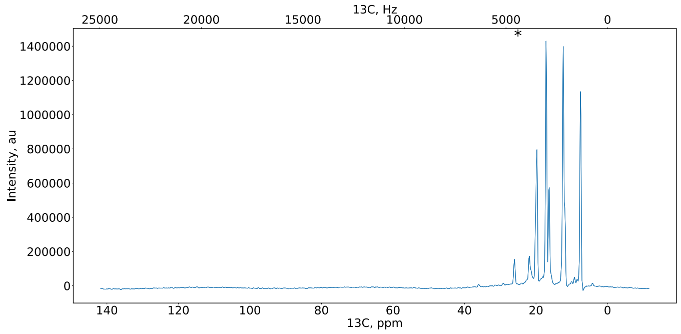

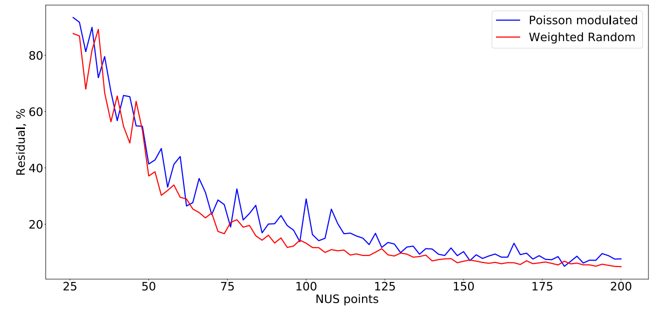

Peak:152

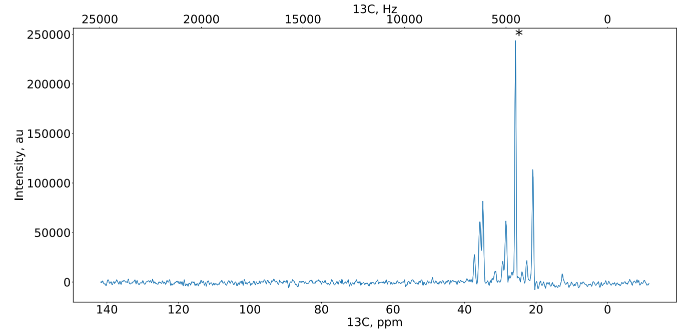

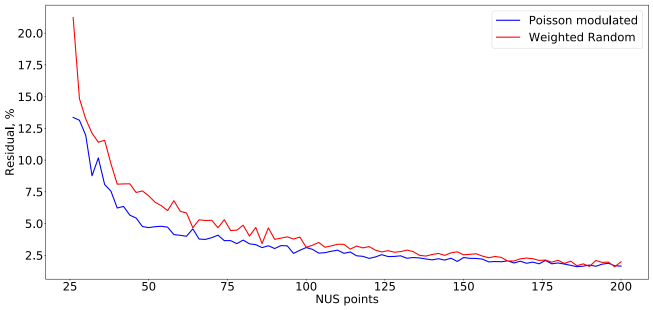

Peak:153

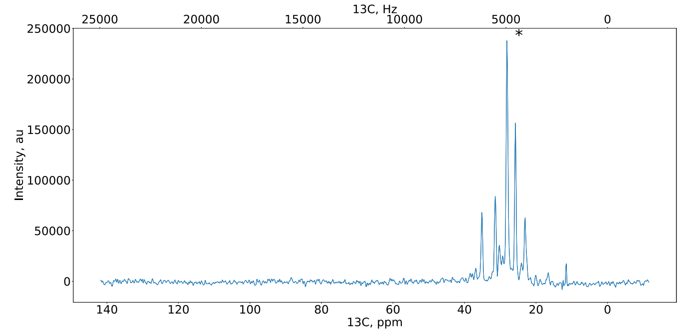

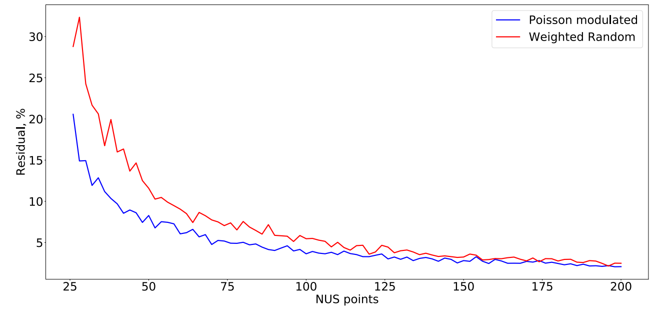

Peak:154

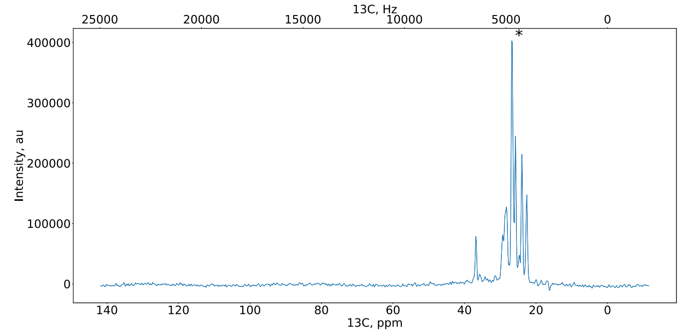

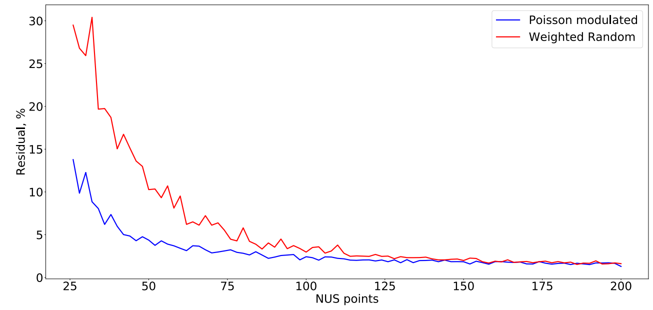

Peak:155

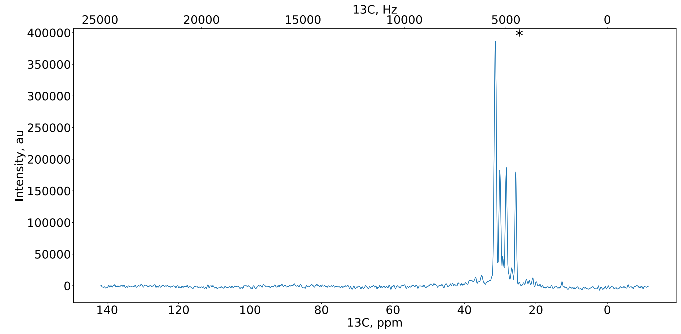

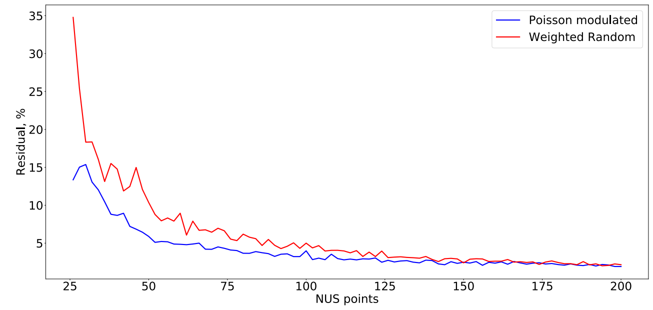

Peak:156

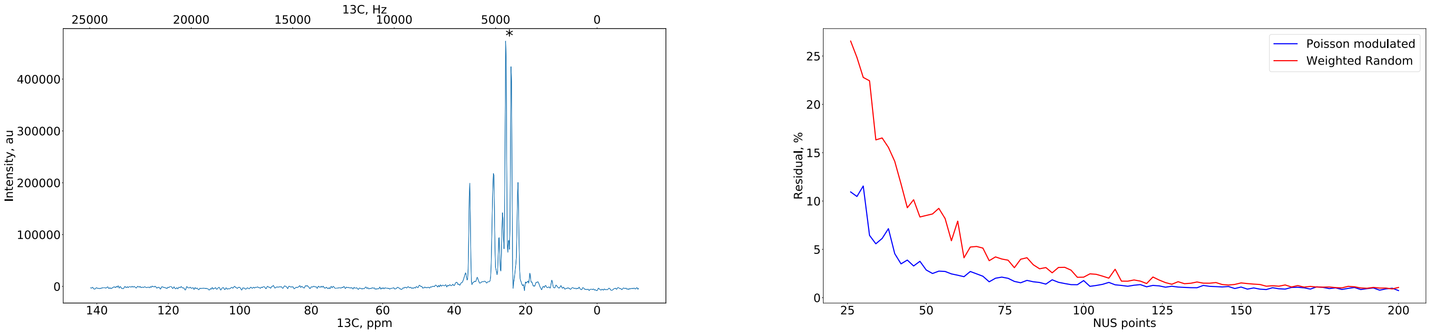

Peak:157

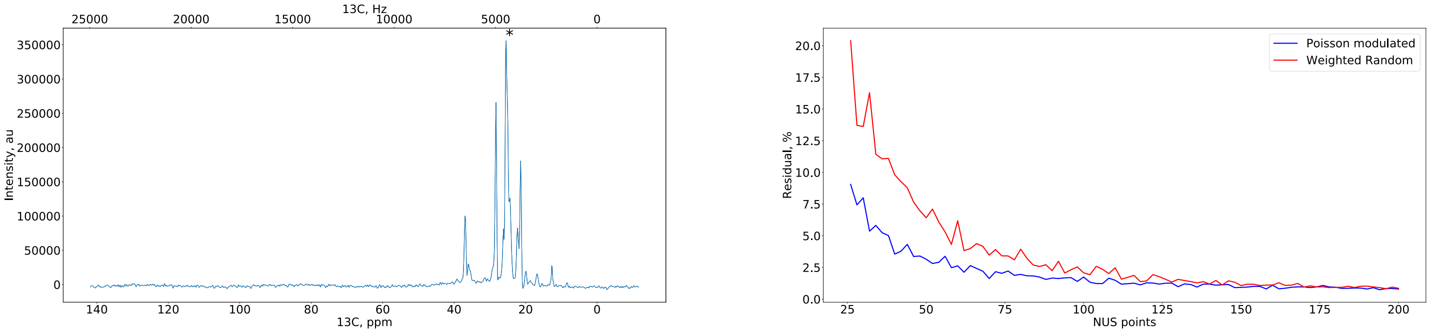

Peak:158

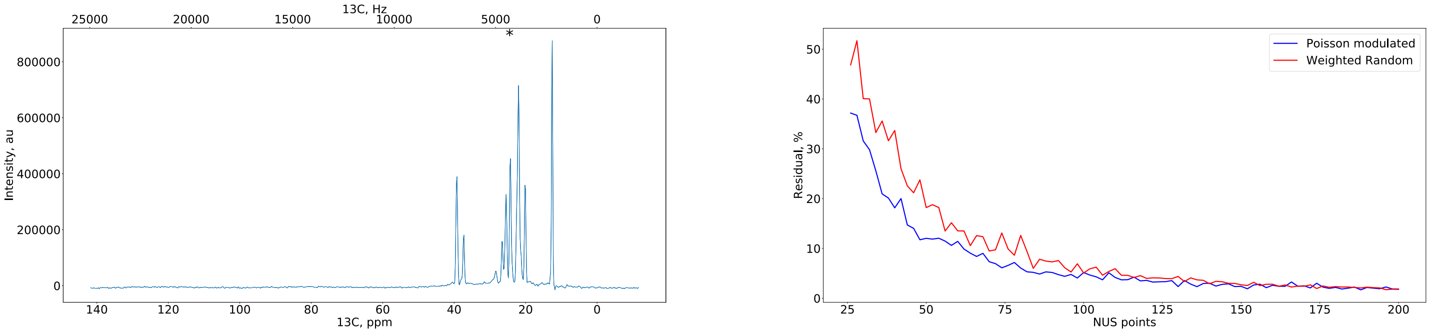

Peak:159

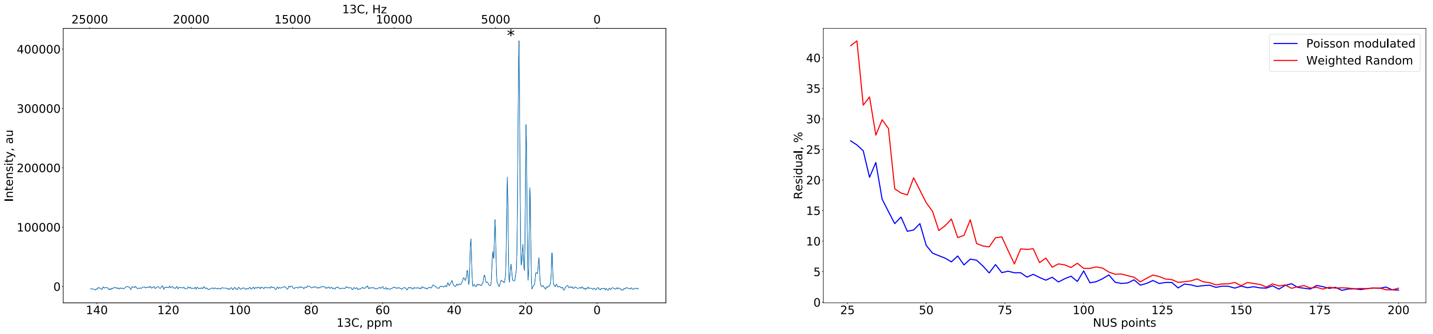

Peak:160

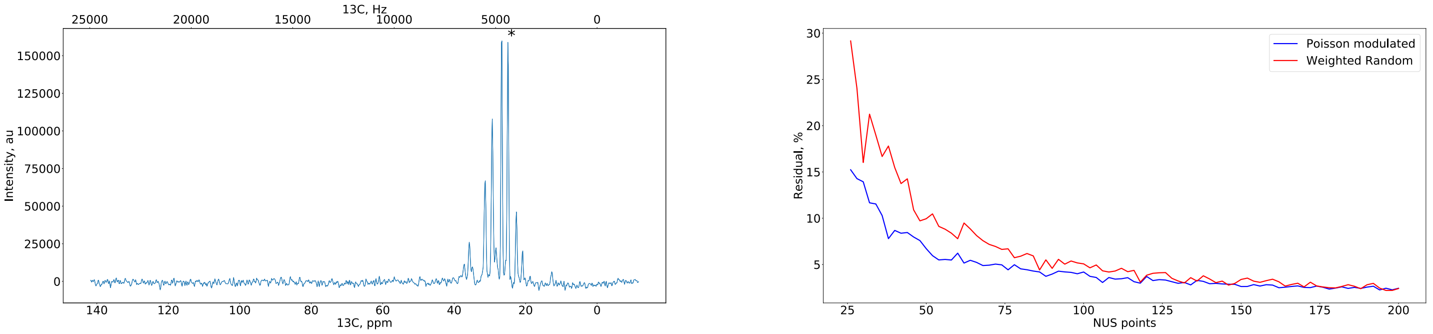

Peak:161

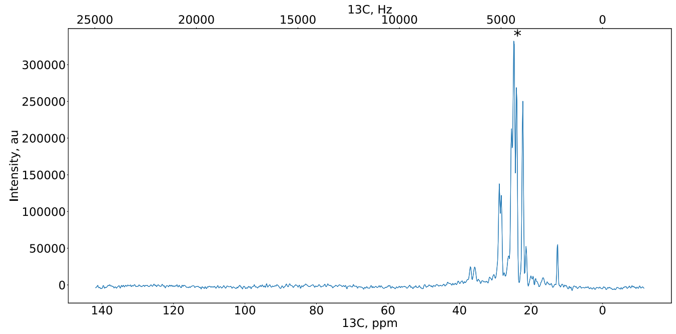

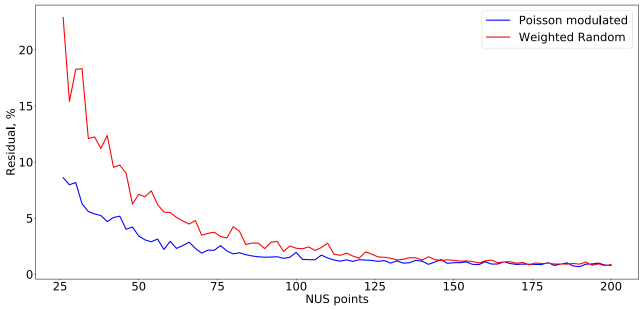

Peak:162

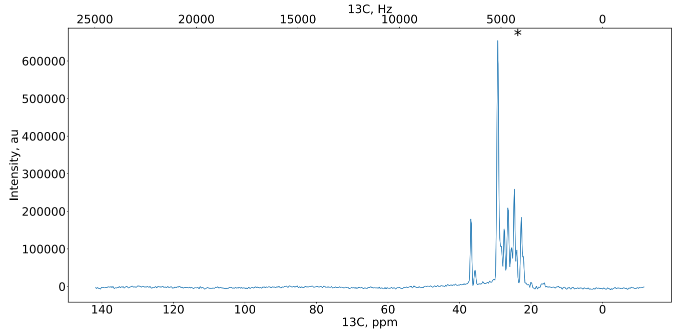

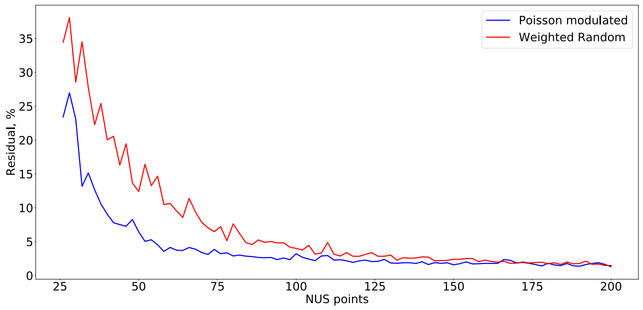

Peak:163

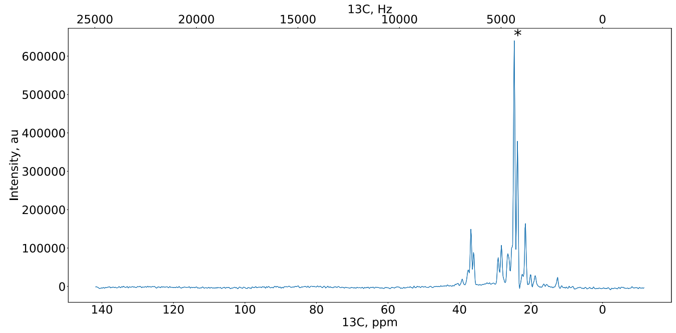

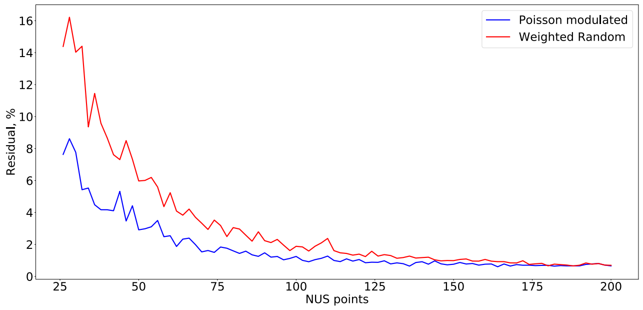

Peak:164

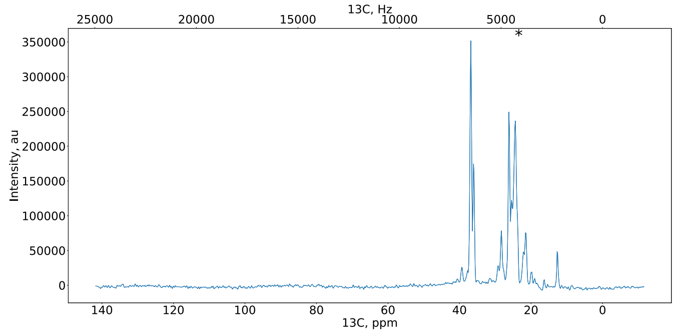

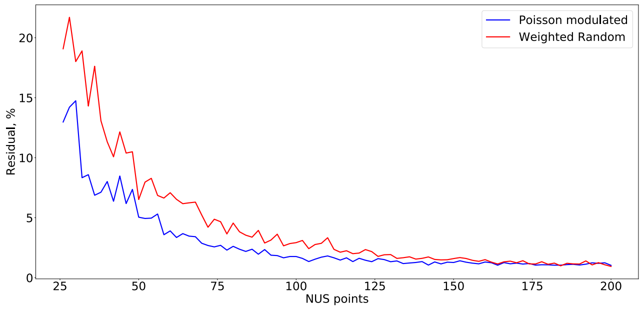

Peak:165

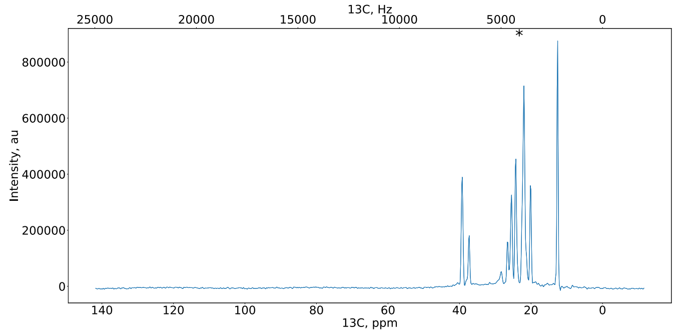

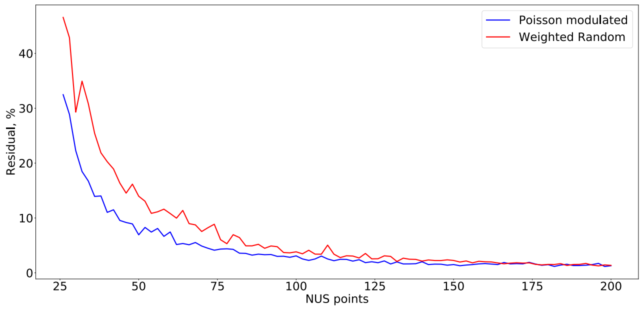

Peak:166

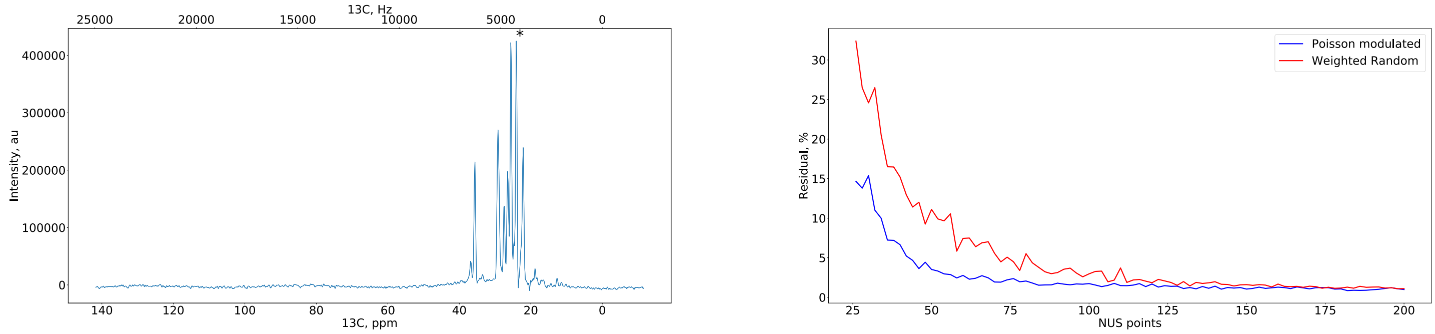

Peak:167

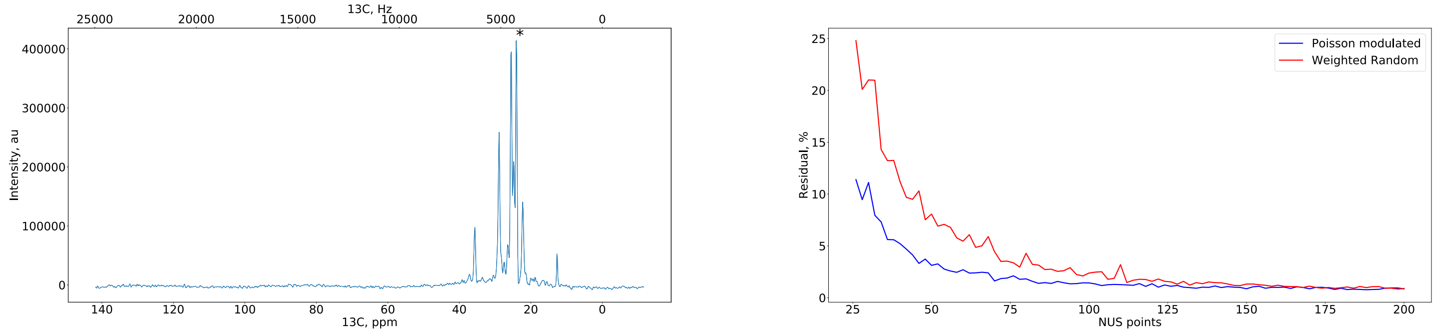

Peak:168

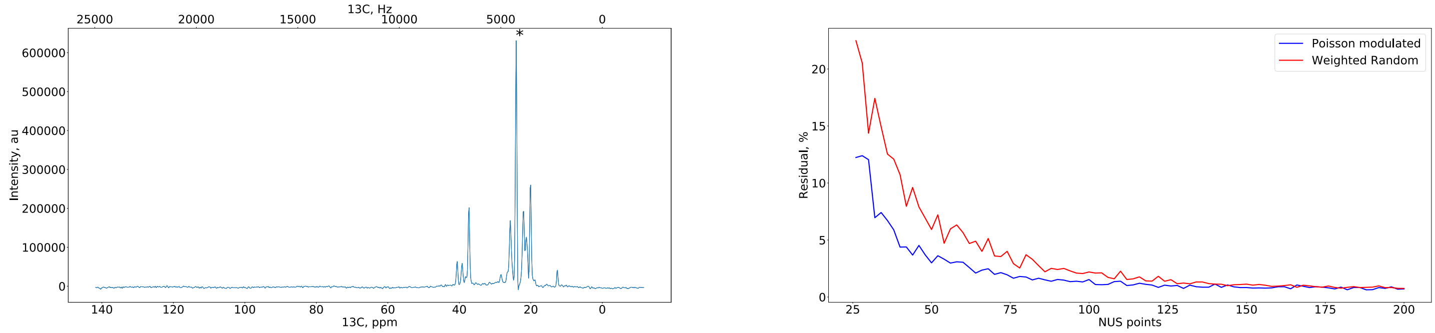

Peak:169

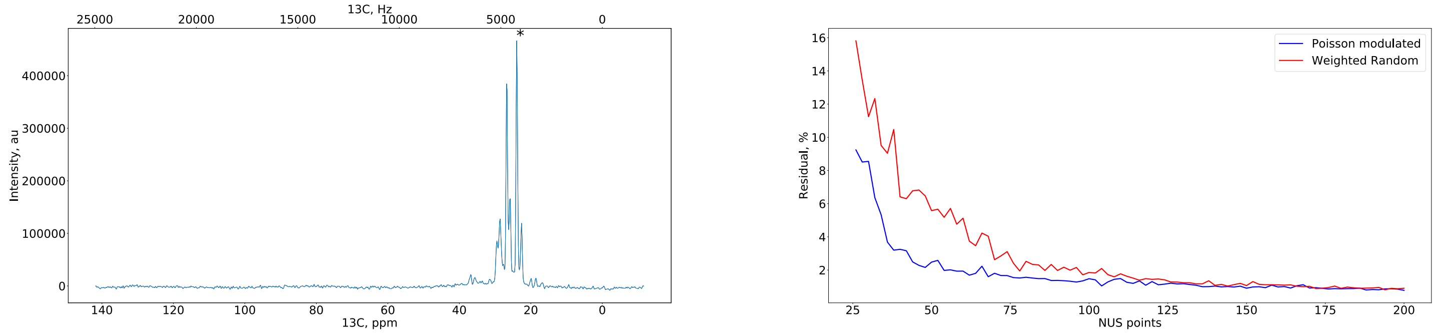

Peak:170

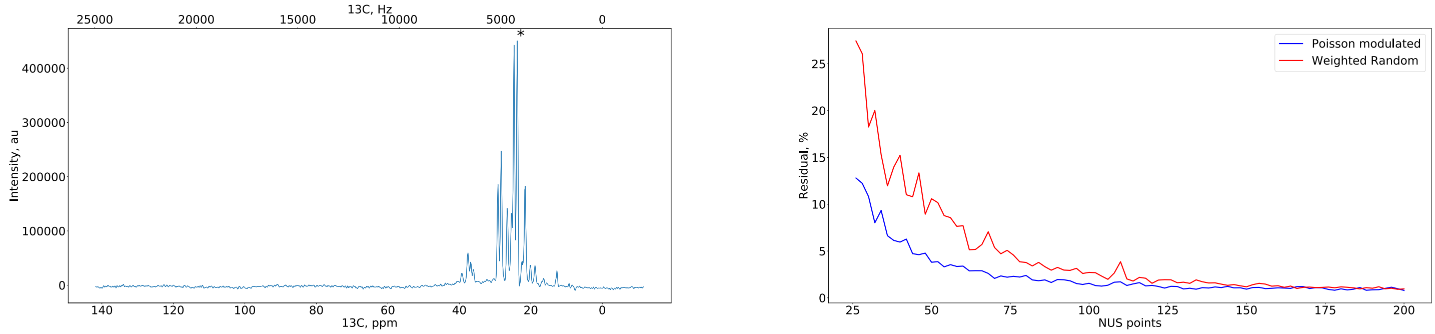

Peak:171

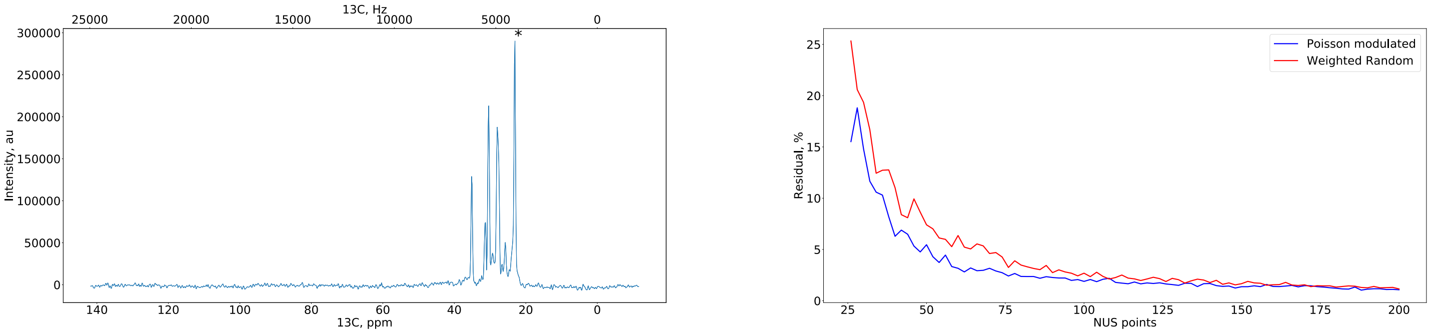

Peak:172

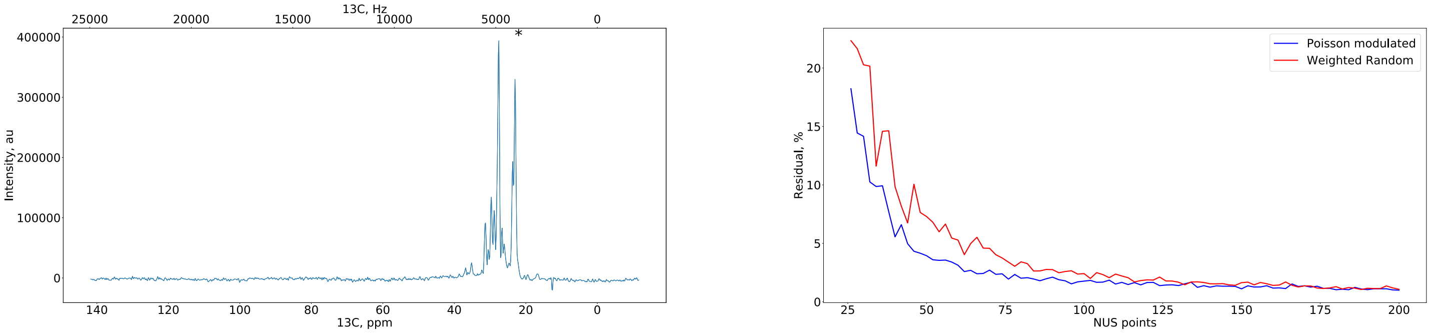

Peak:173

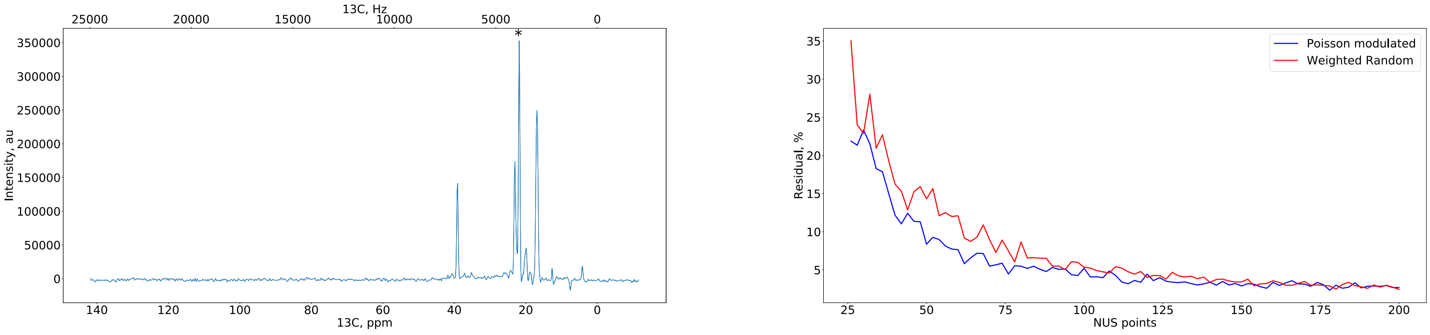

Peak:174

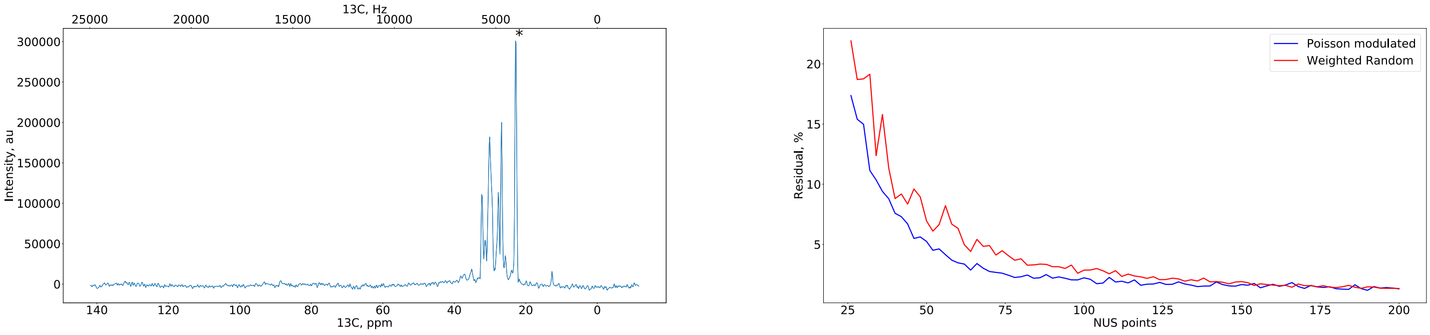

Peak:175

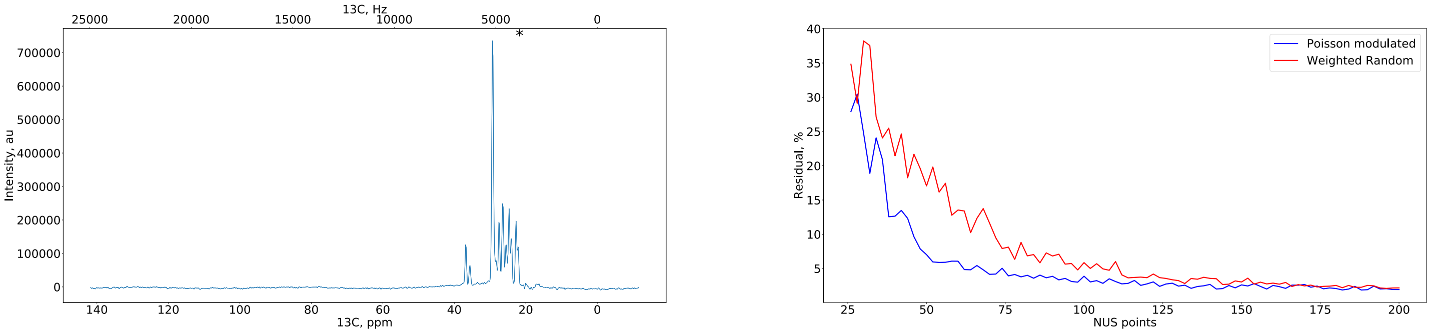

Peak:176

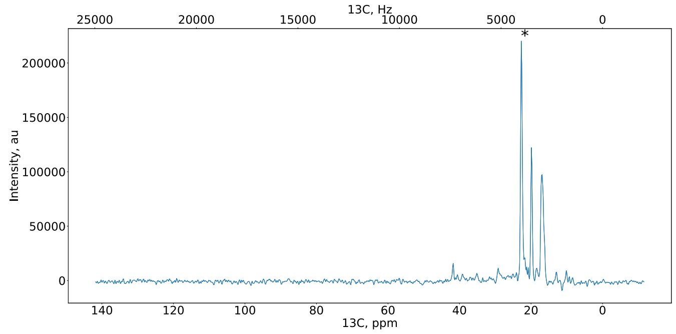

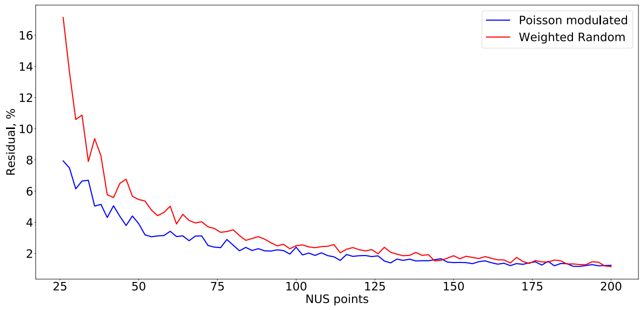

Peak:177

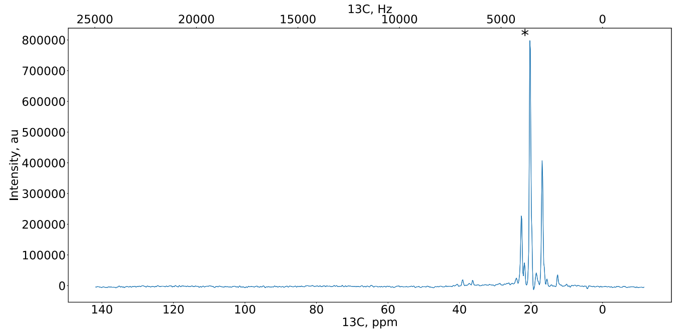

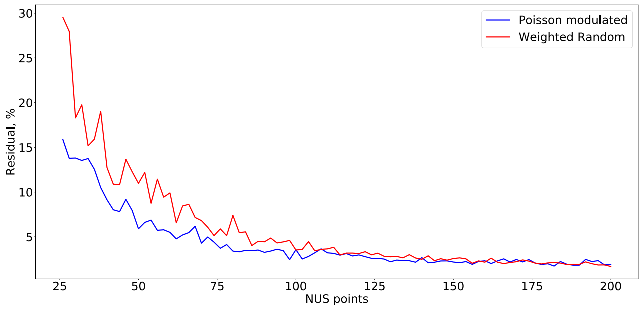

Peak:178

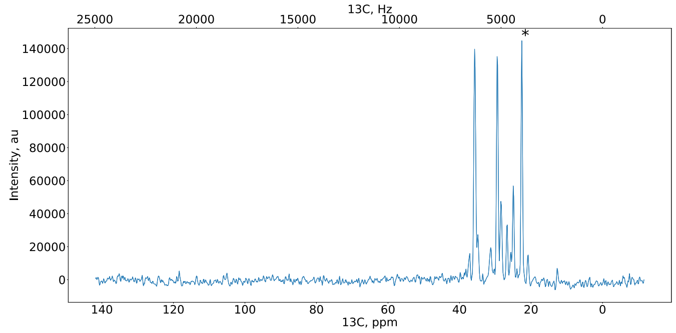

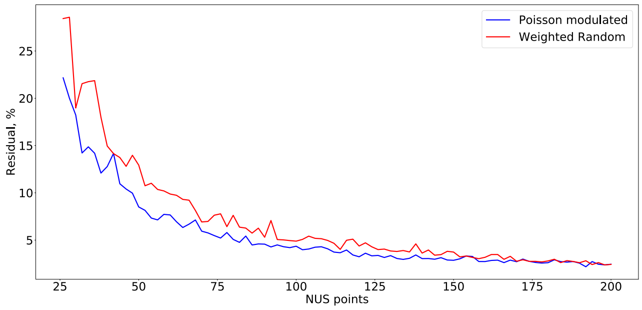

Peak:179

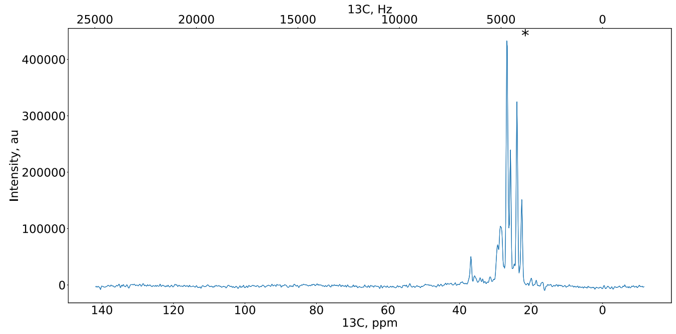

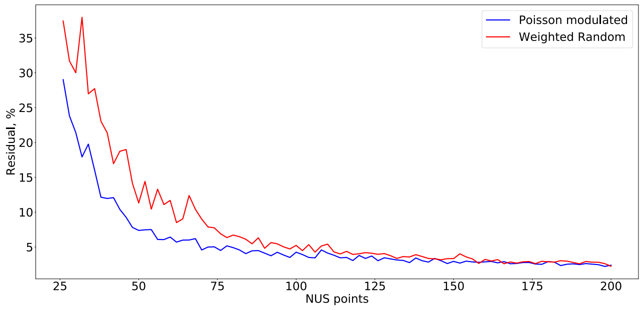

Peak:180

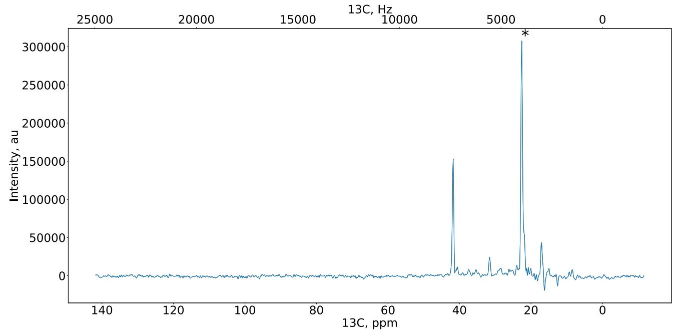

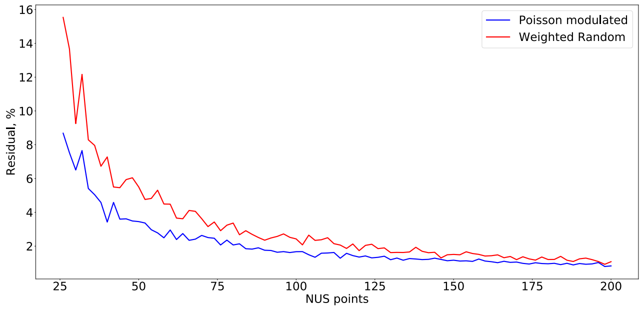

Peak:181

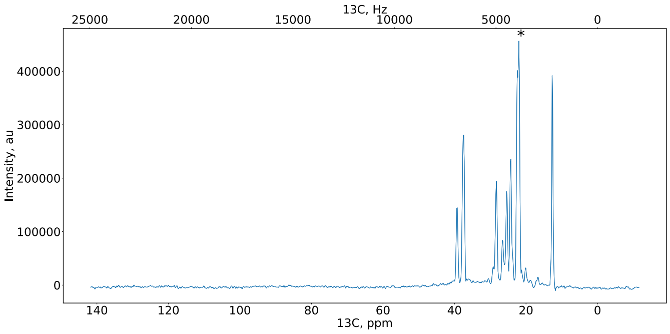

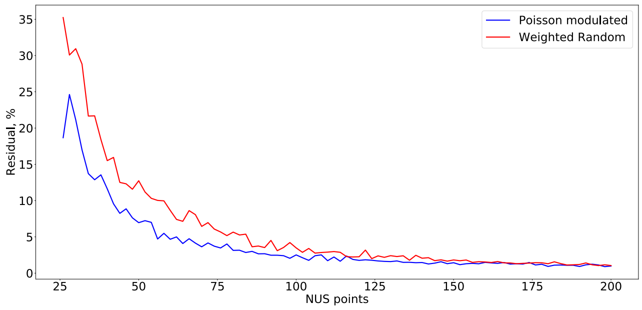

Peak:182

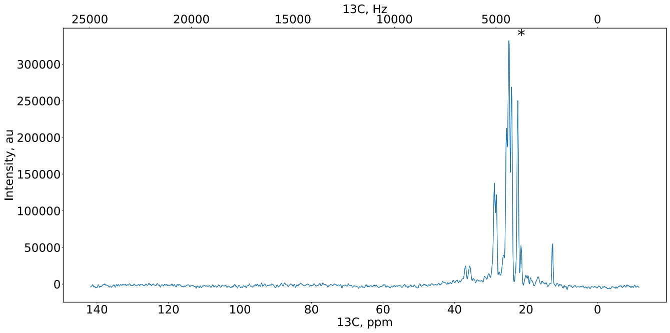

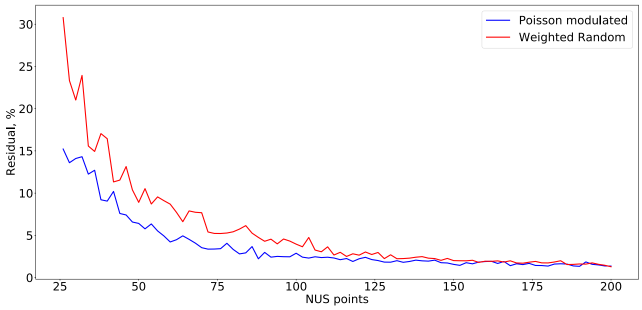

Peak:183

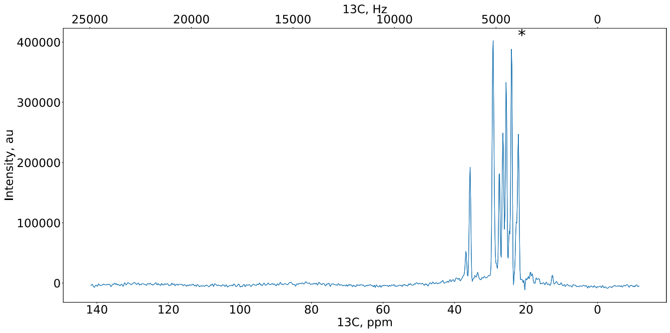

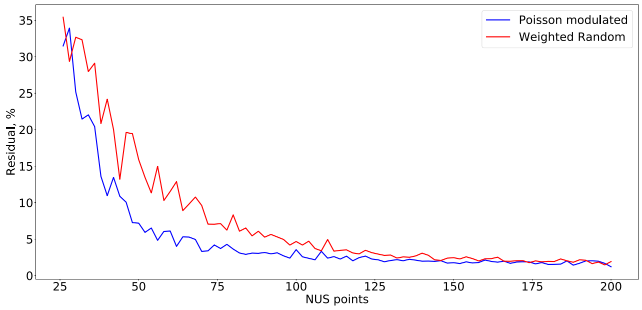

Peak:184

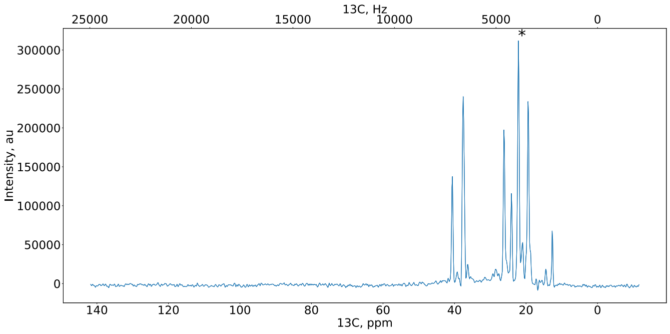

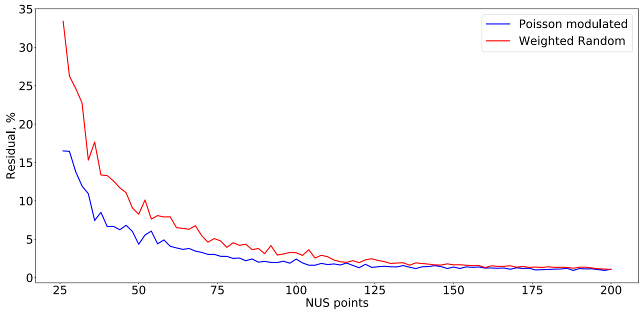

Peak:185

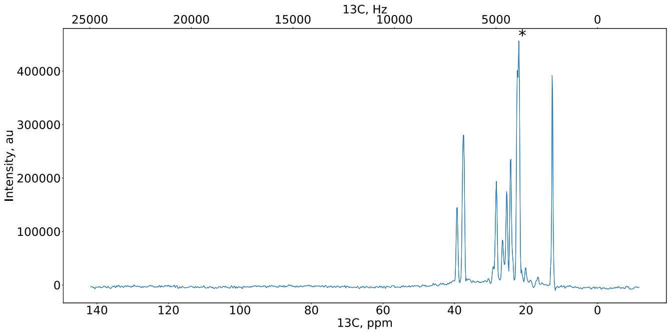

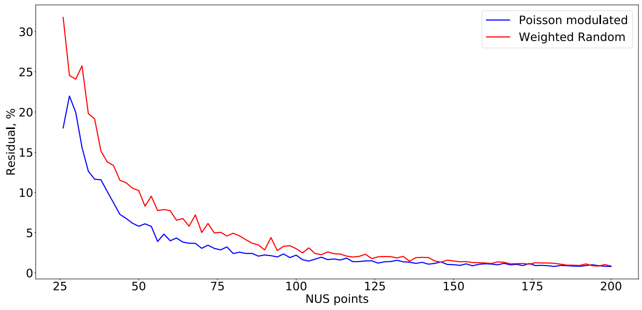

Peak:186

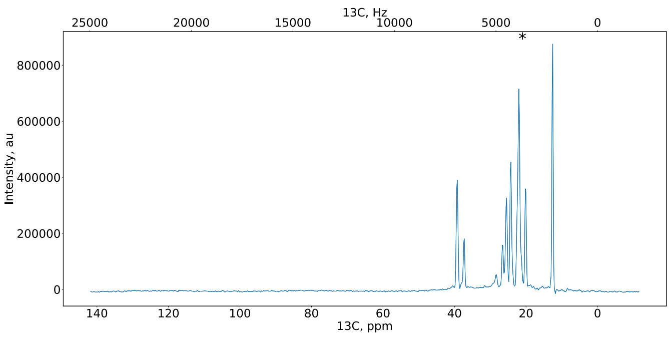

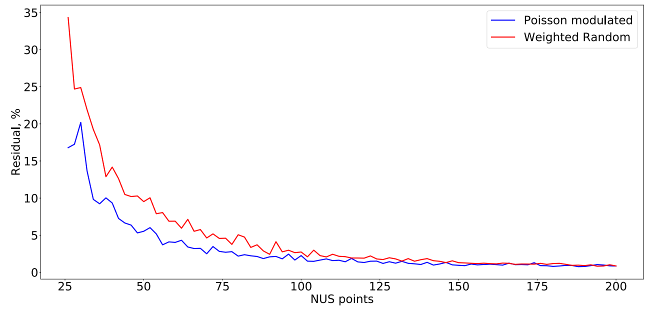

Peak:187

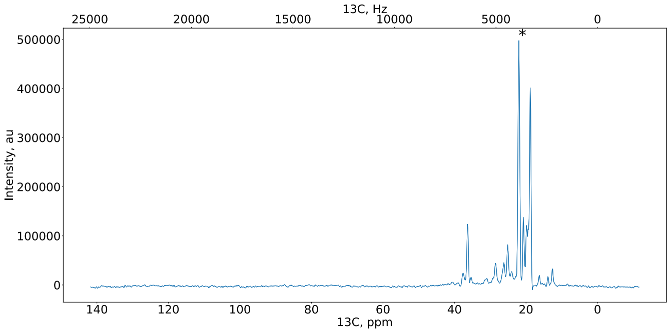

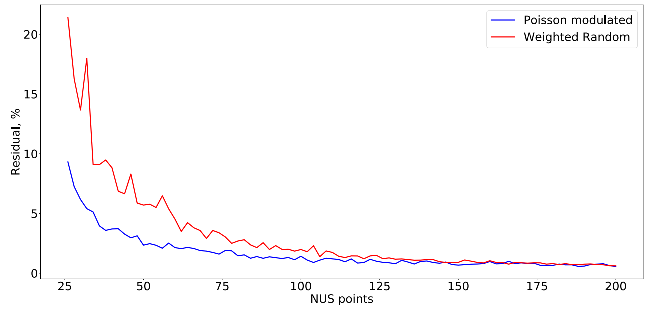

Peak:188

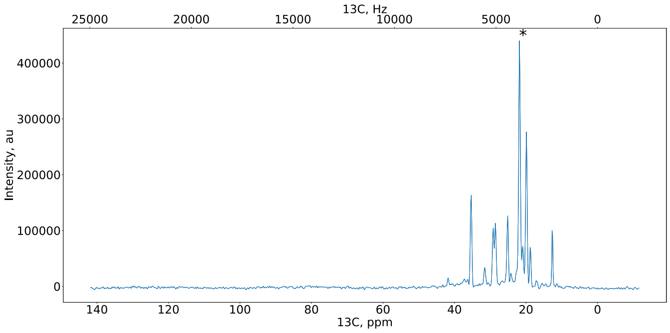

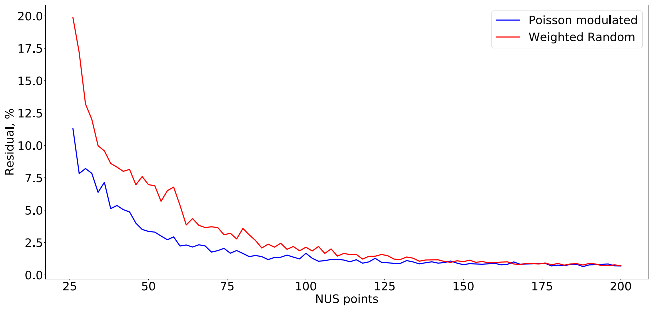

Peak:189

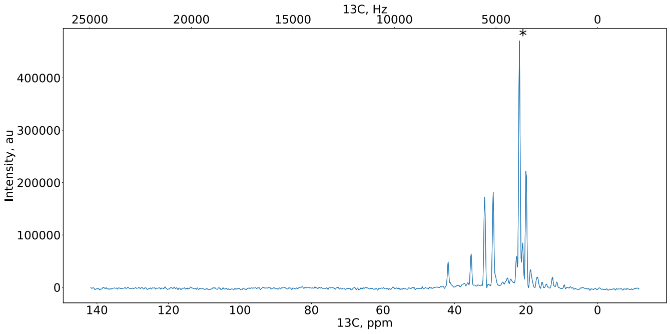

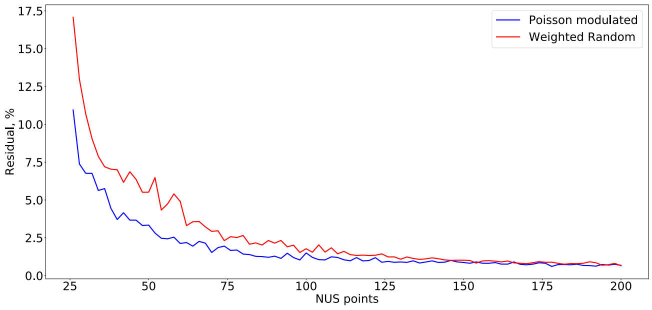

Peak:190

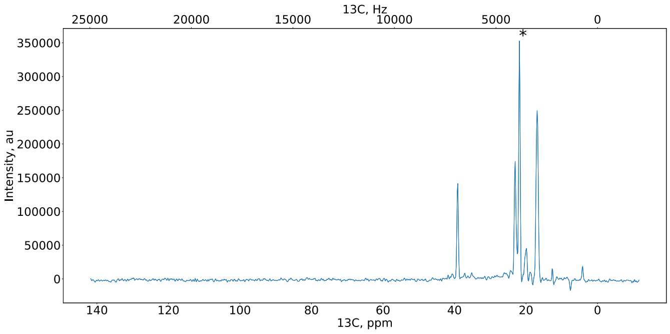

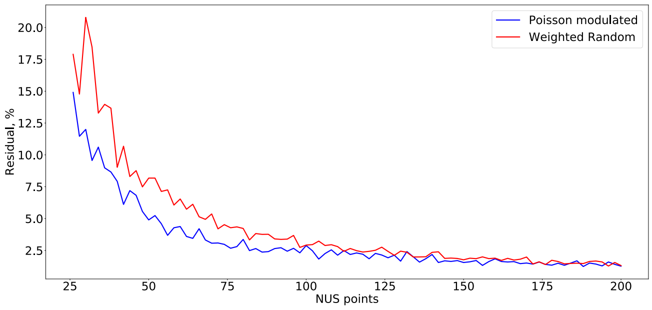

Peak:191

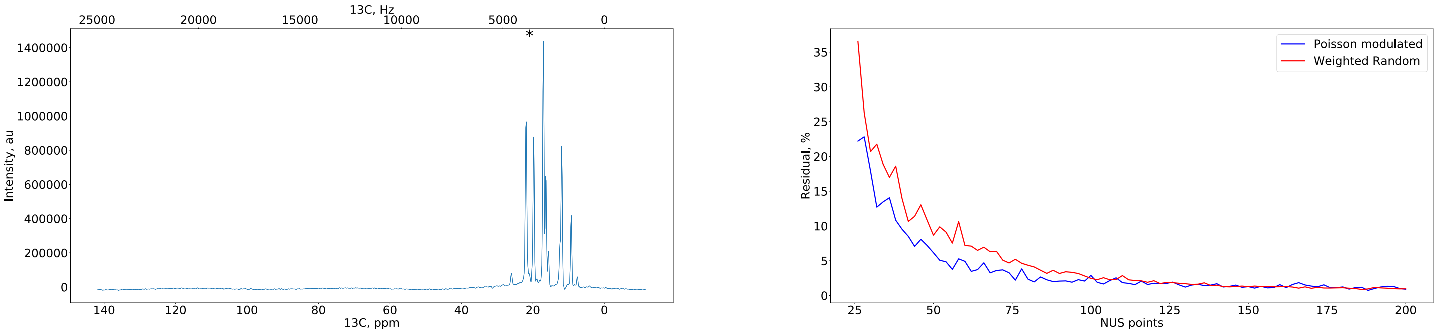

Peak:192

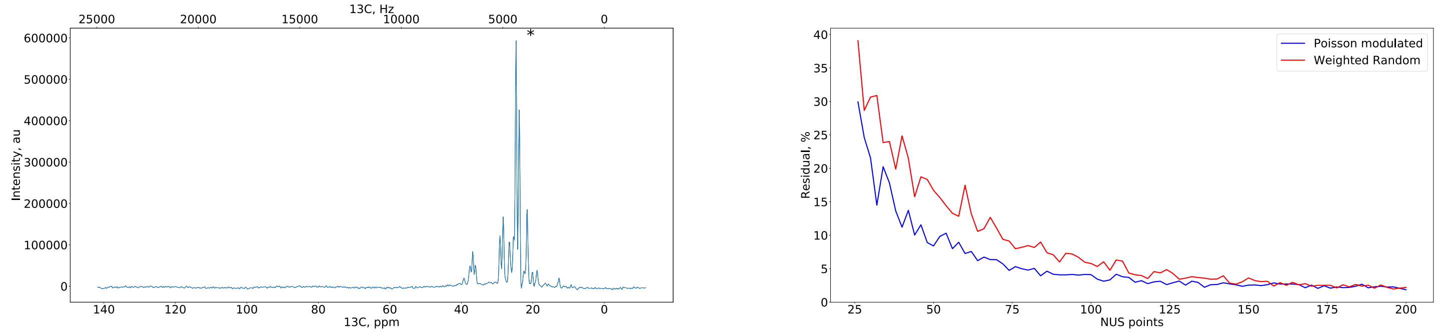

Peak:193

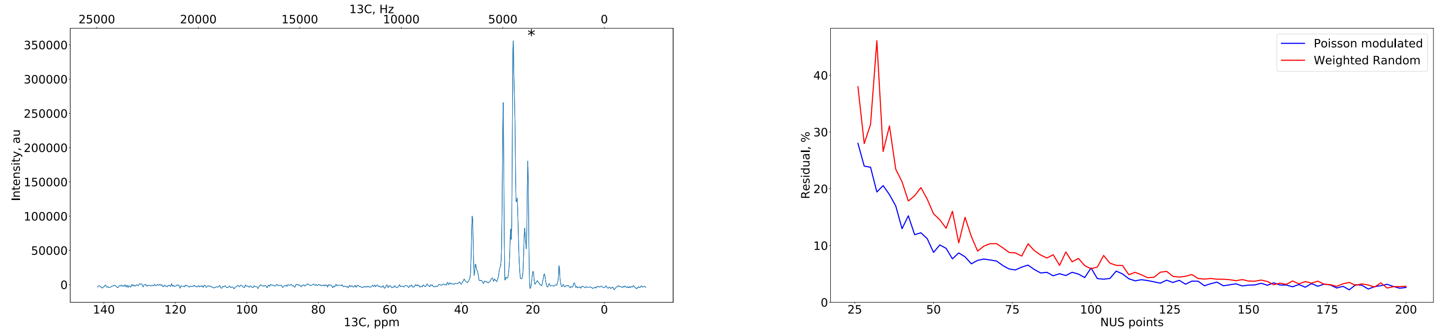

Peak:194

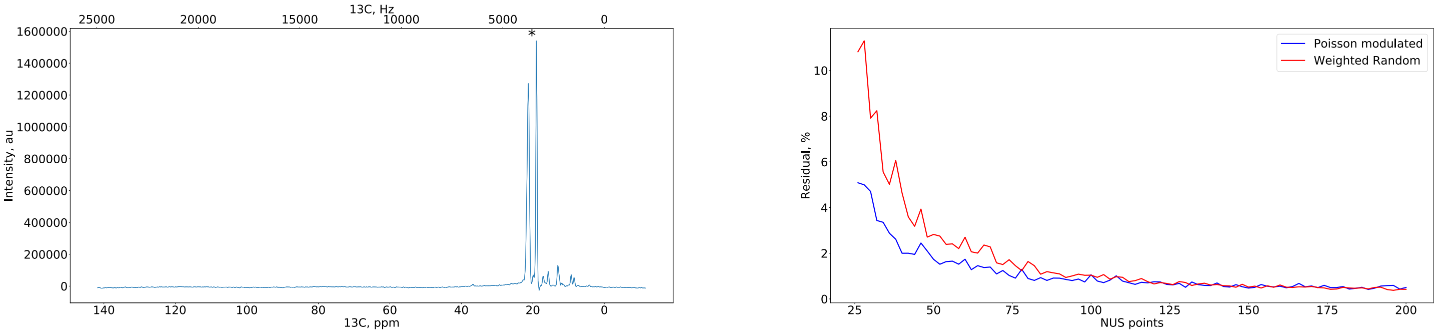

Peak:195

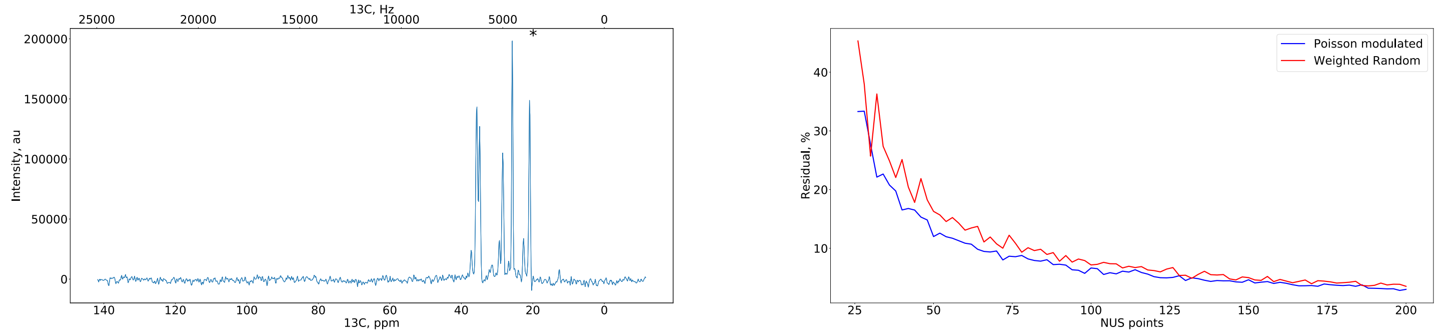

Peak:196

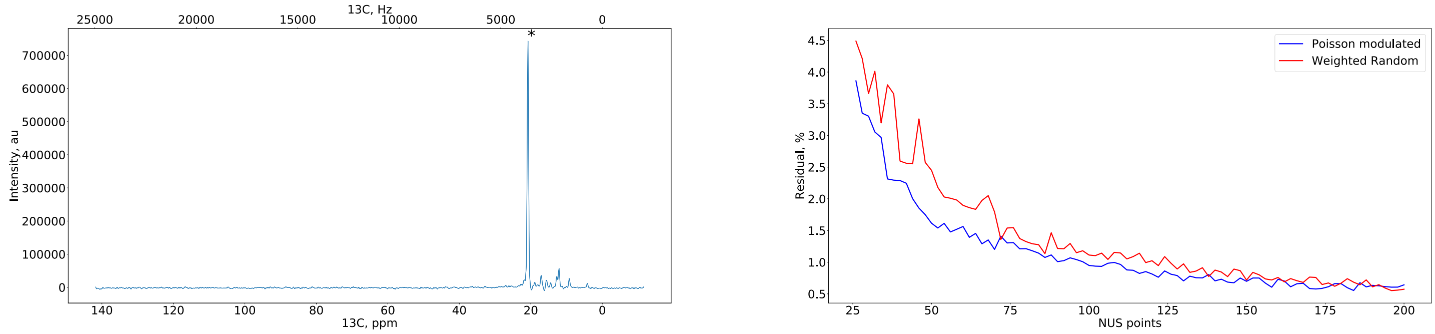

Peak:197

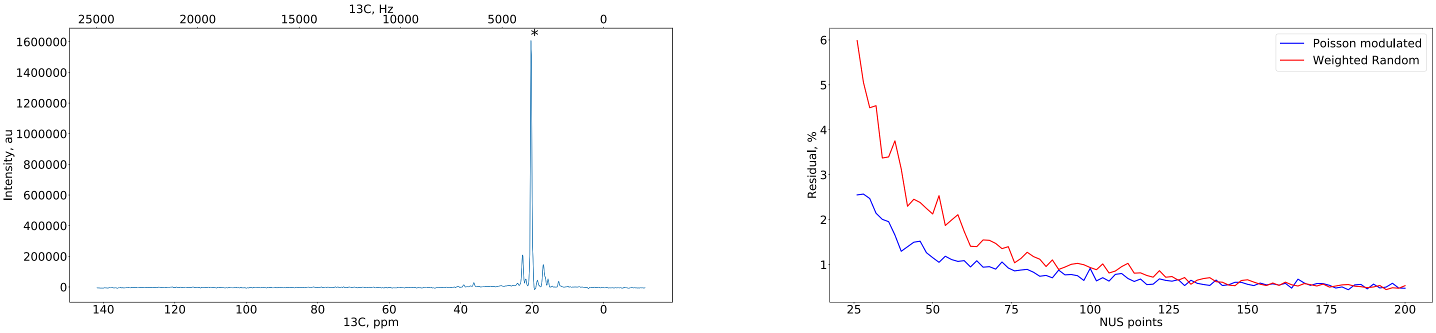

Peak:198

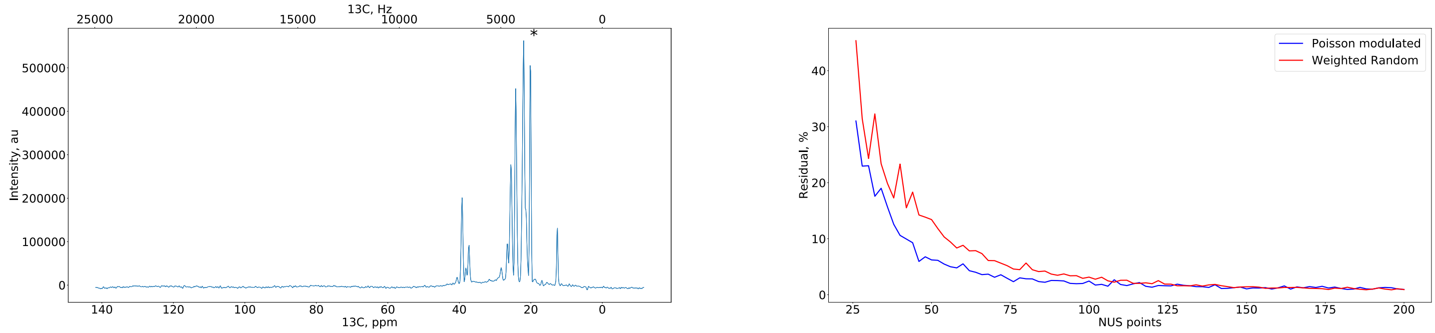

Peak:199

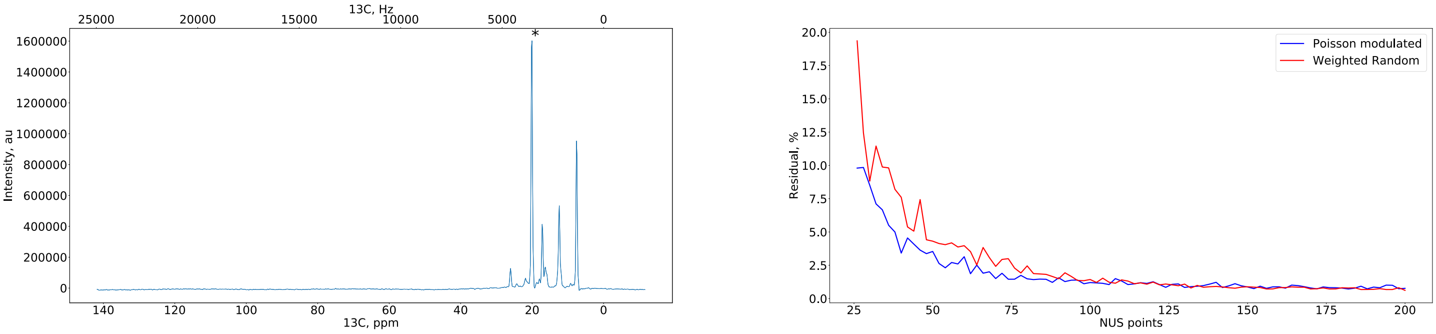

Peak:200

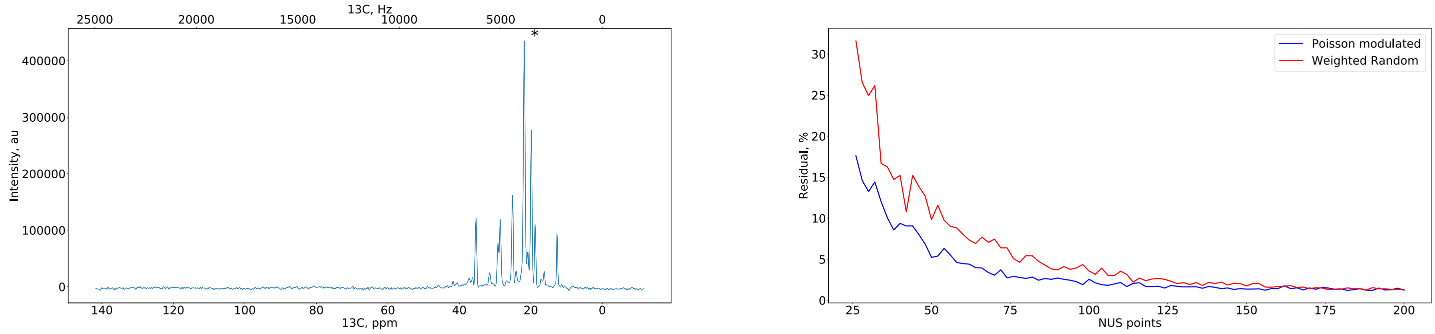

Peak:201

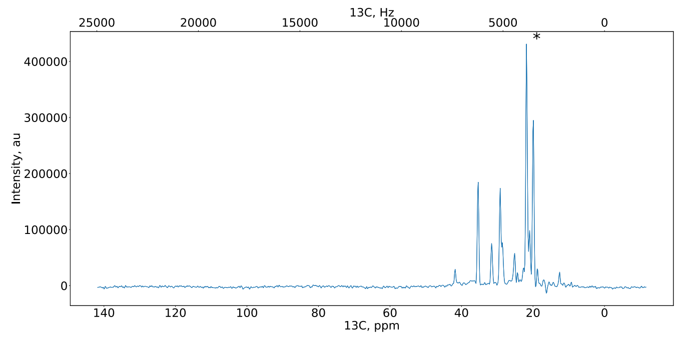

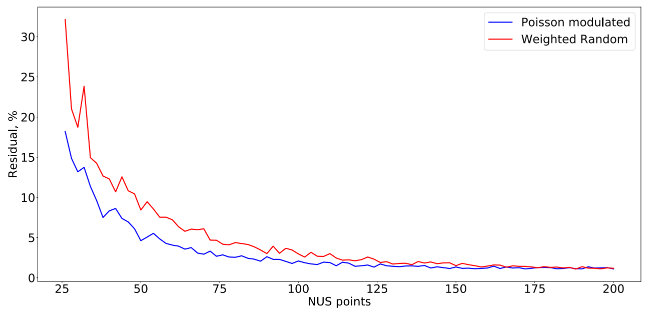

Peak:202

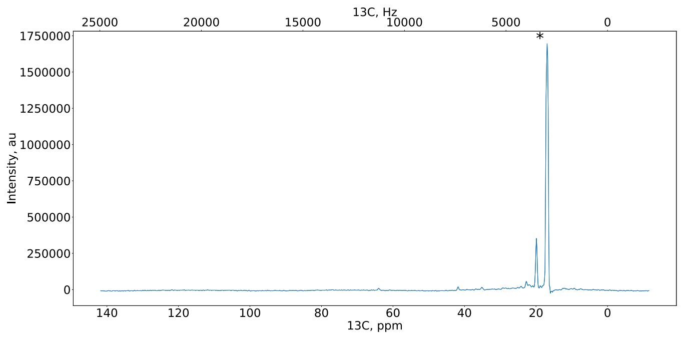

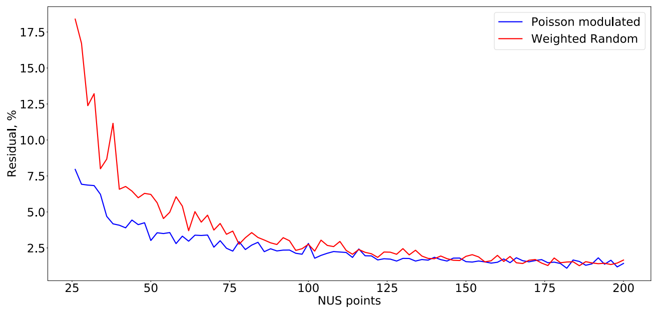

Peak:203

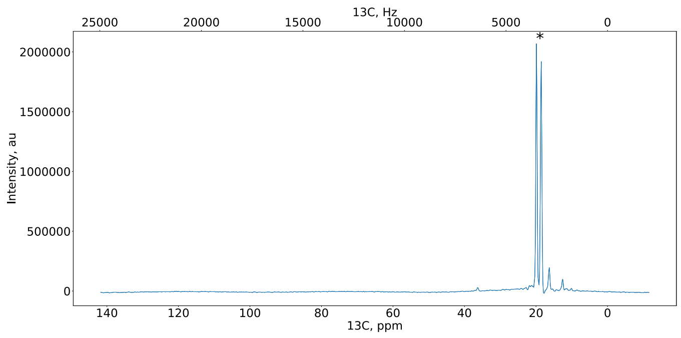

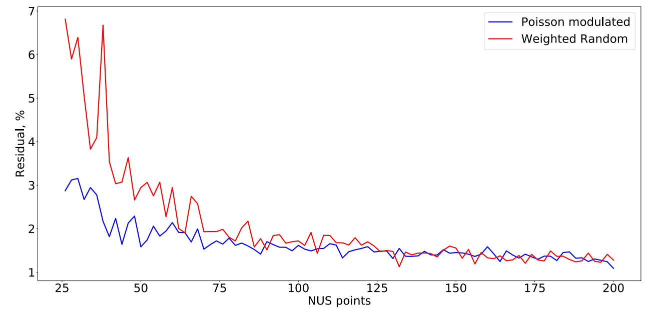

Peak:204

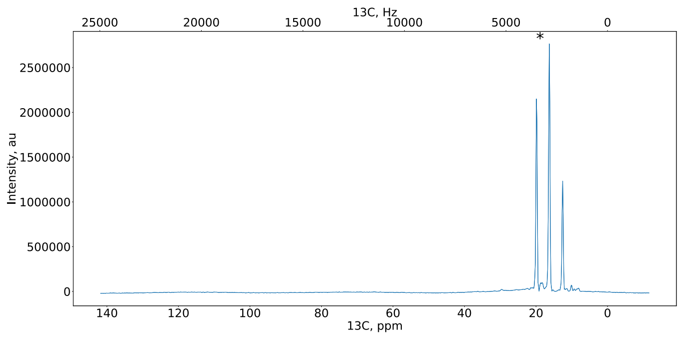

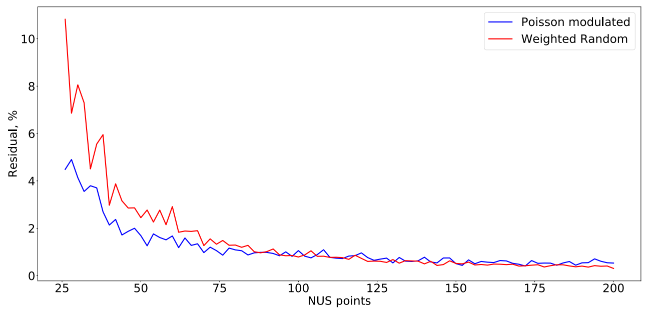

Peak:205

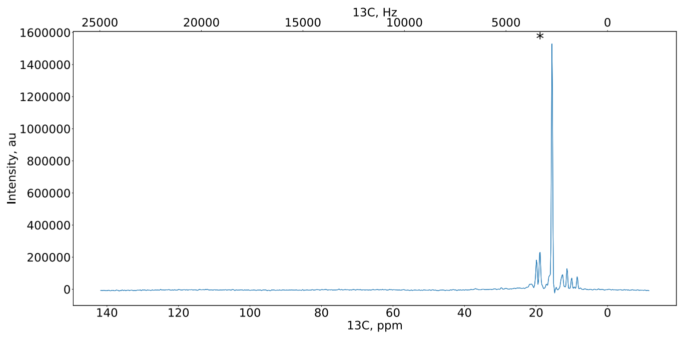

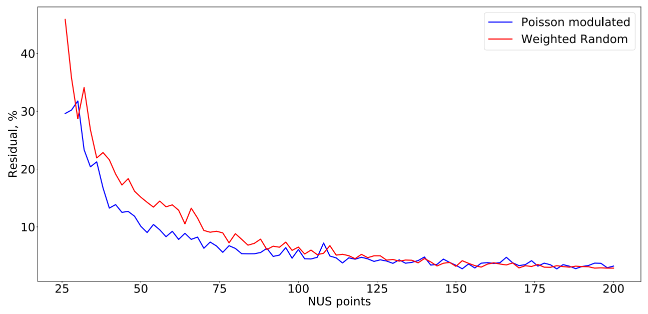

Peak:206

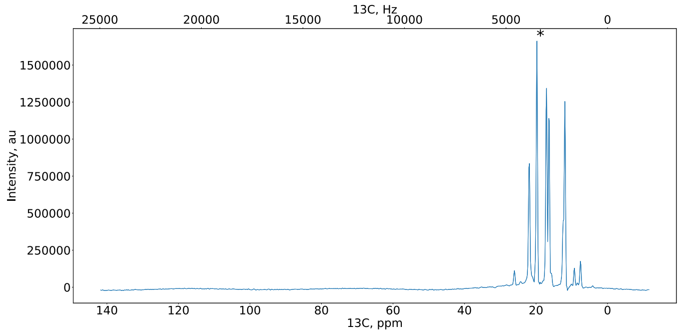

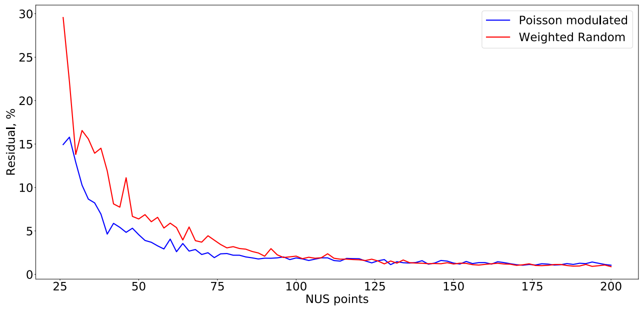

Peak:207

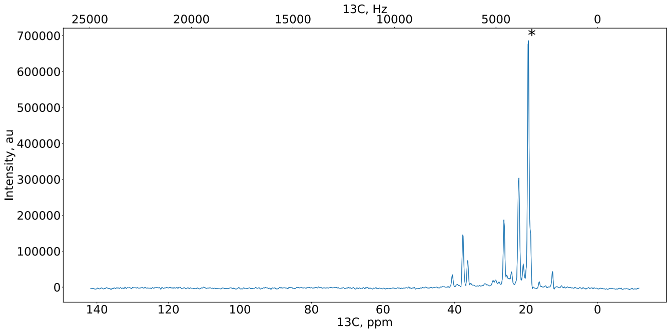

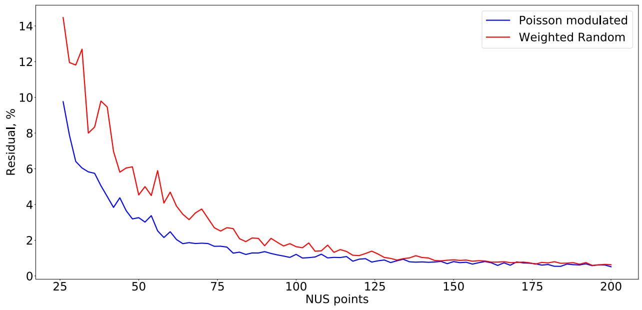

Peak:208

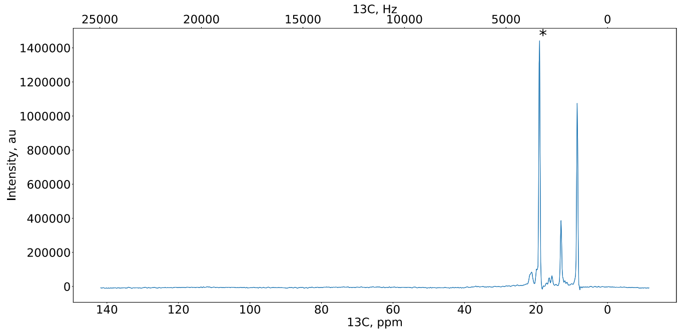

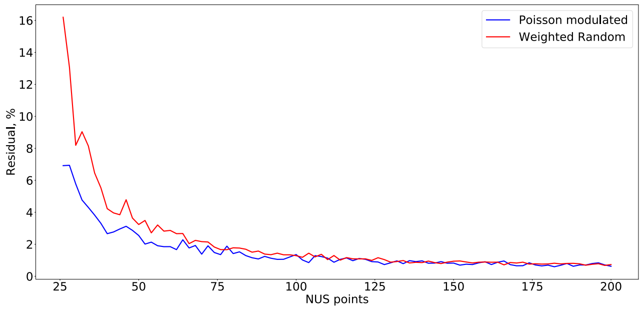

Peak:209

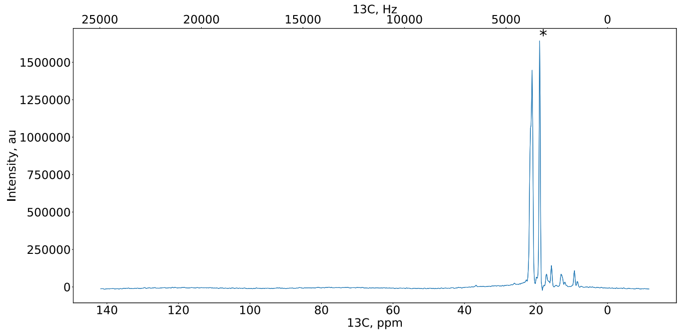

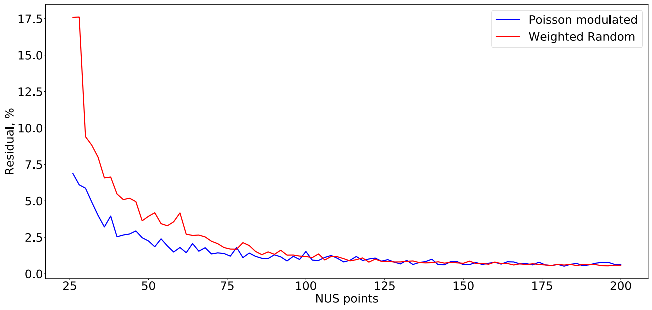

Peak:210

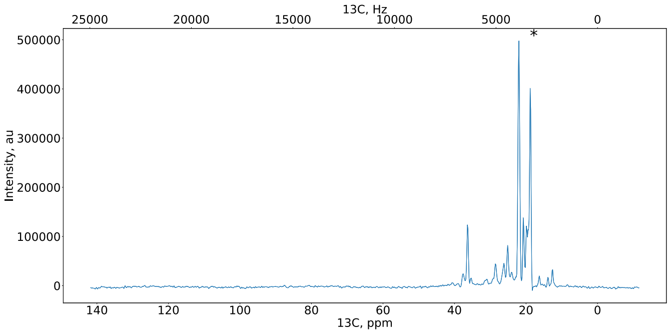

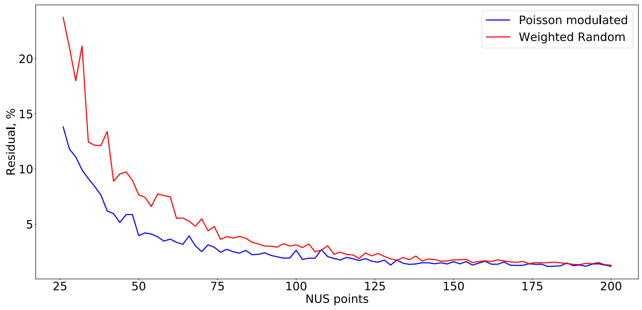

Peak:211

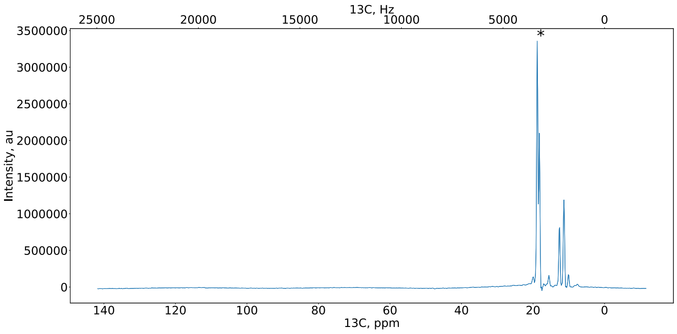

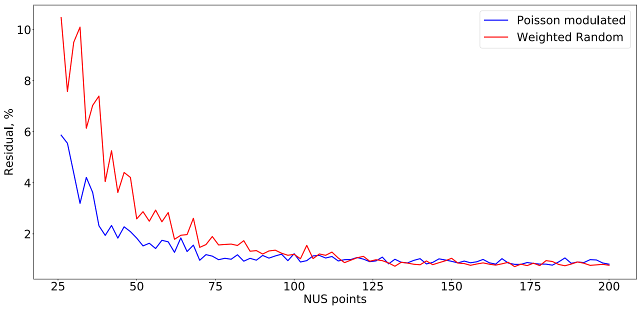

Peak:212

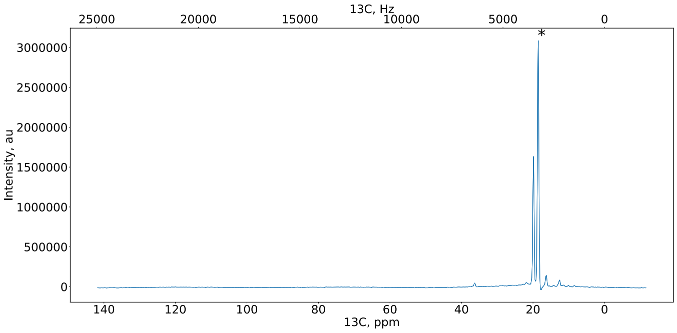

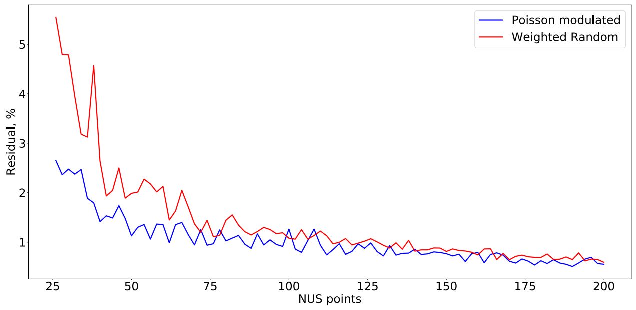

Peak:213

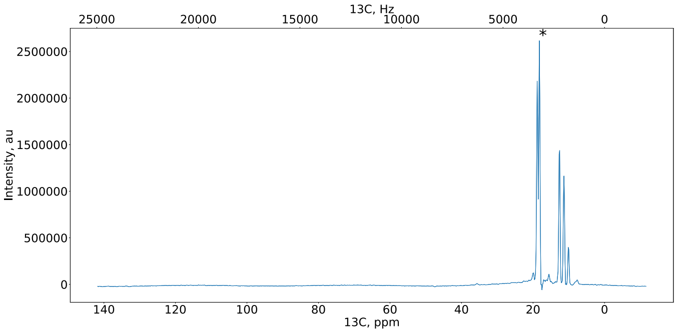

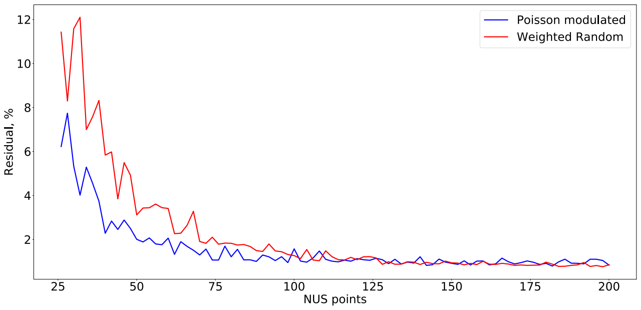

Peak:214

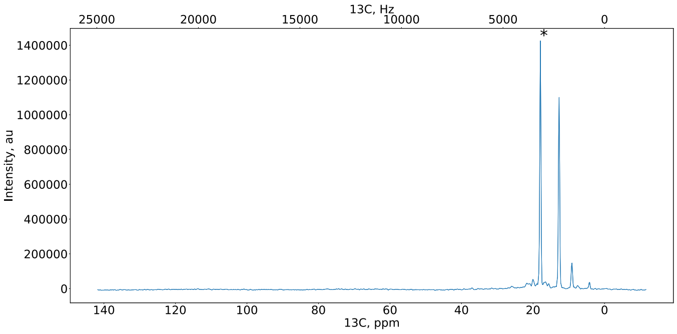

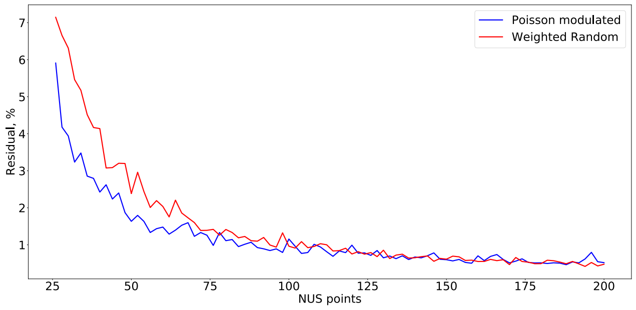

Peak:215

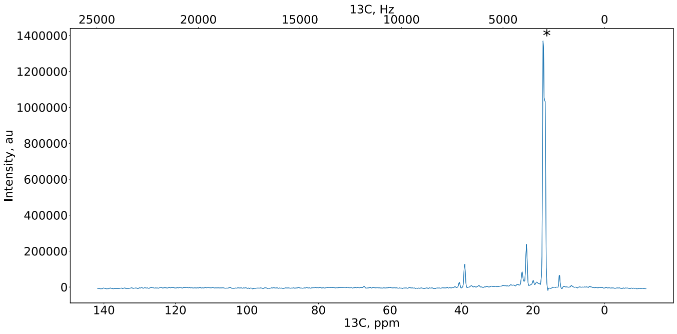

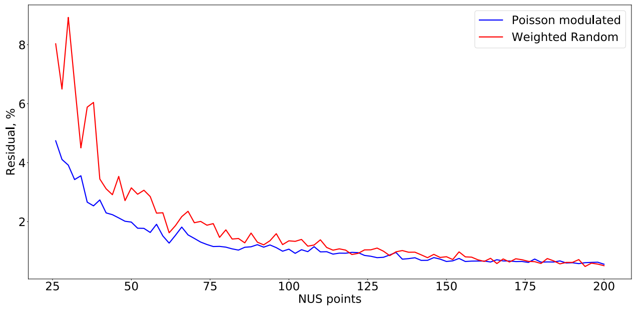

Peak:216

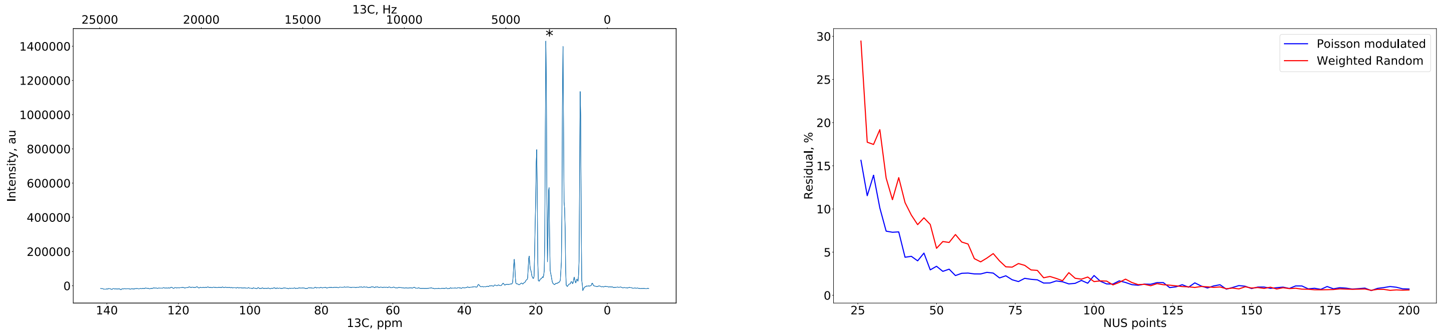

Peak:217

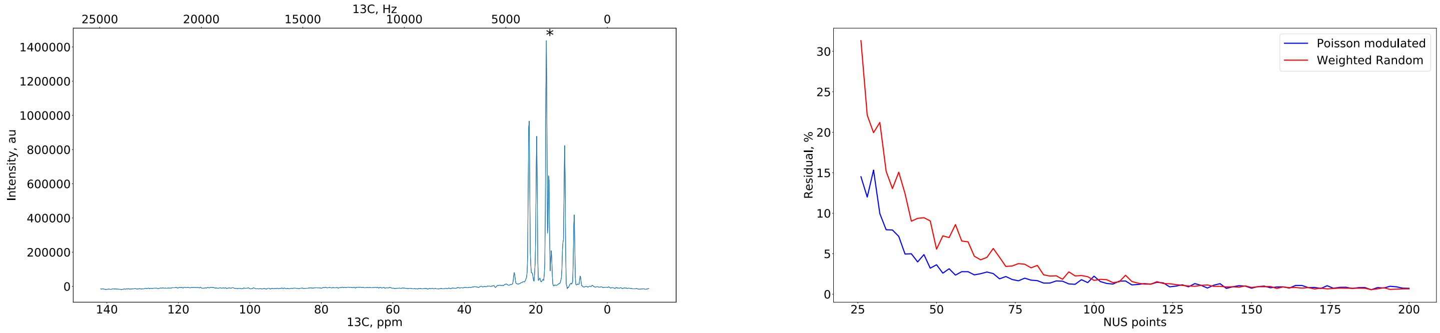

Peak:218

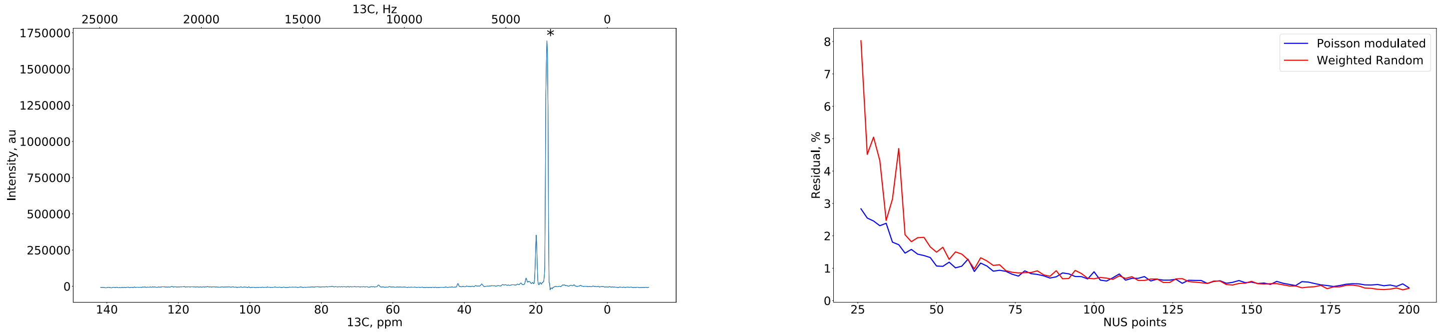

Peak:219

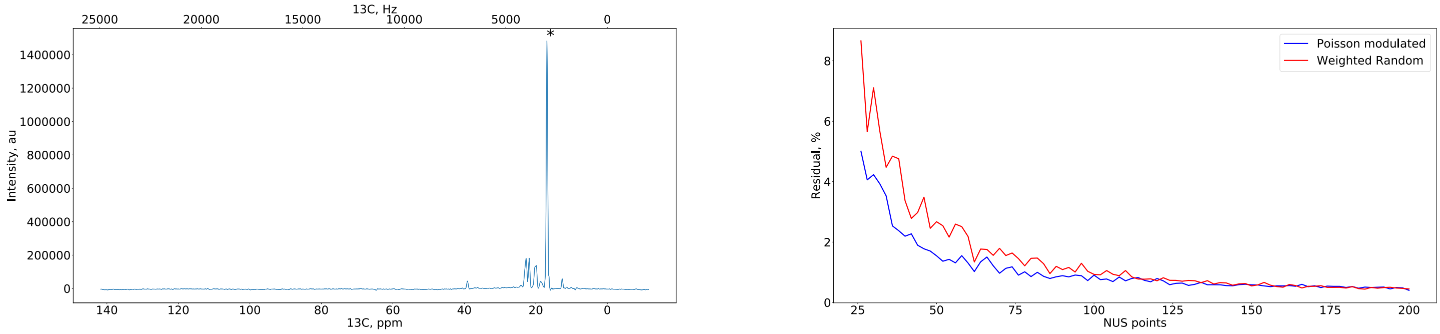

Peak:220

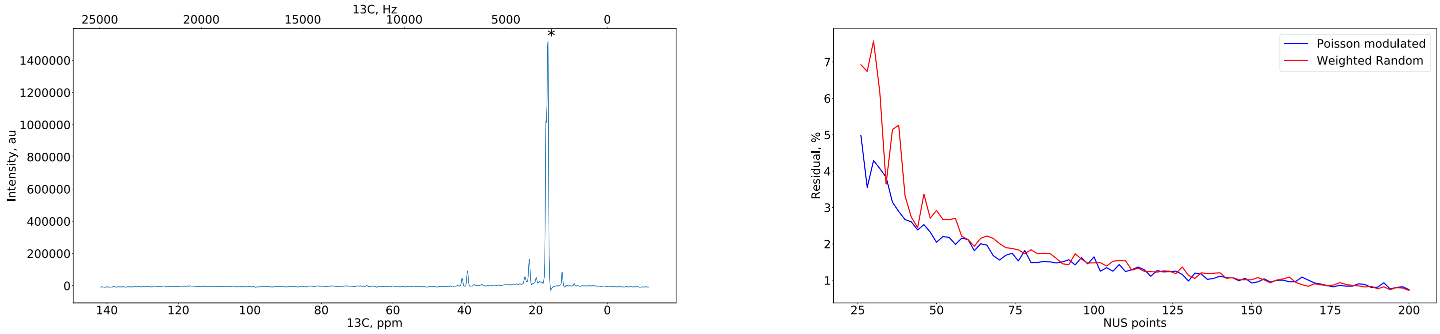

Peak:221

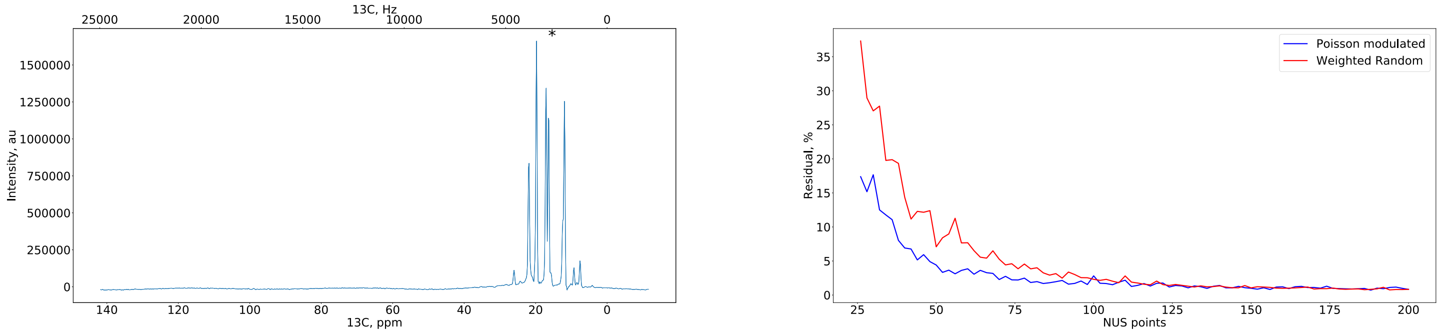

Peak:222

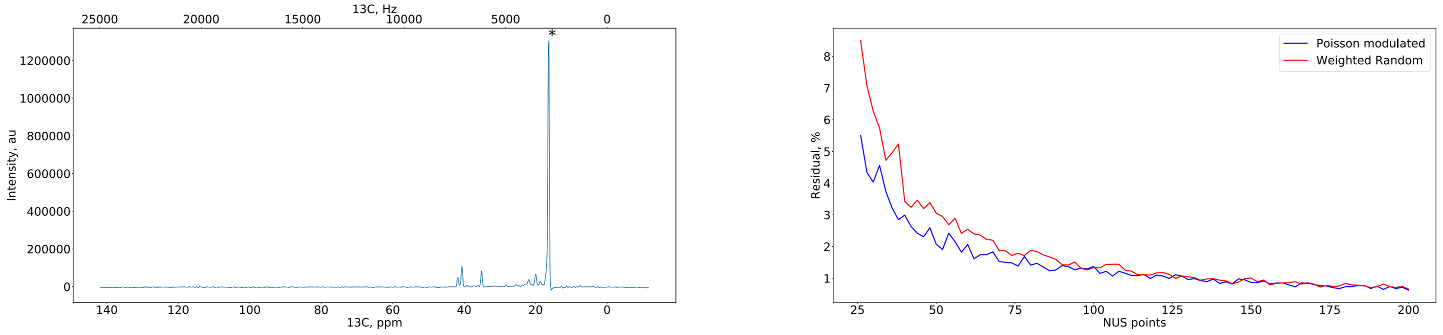

Peak:223

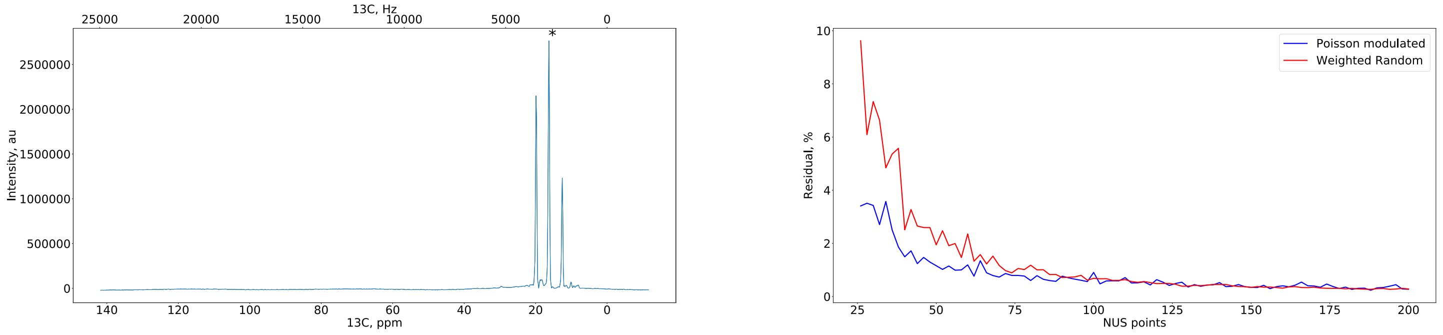

Peak:224

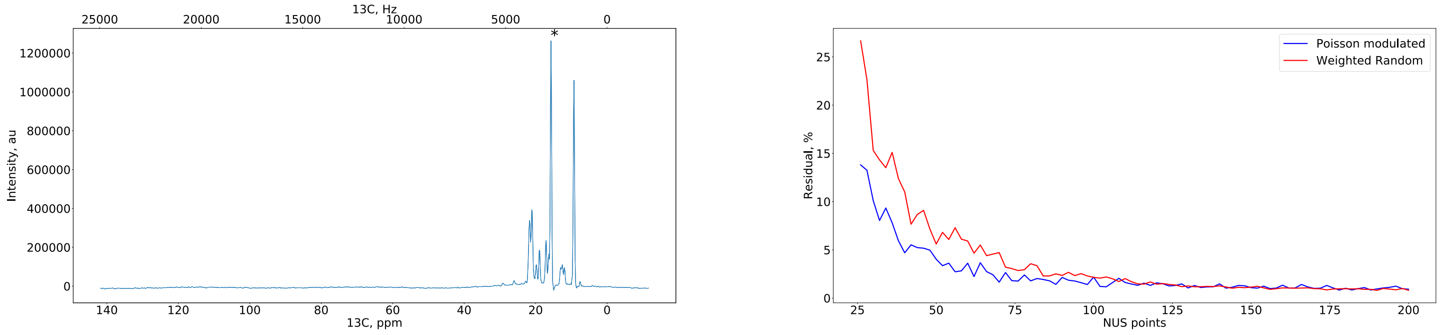

Peak:225

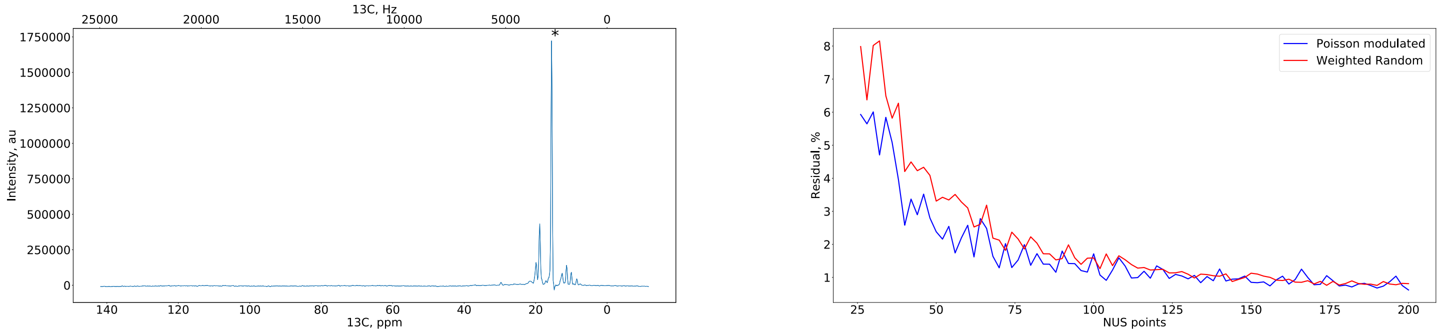

Peak:226

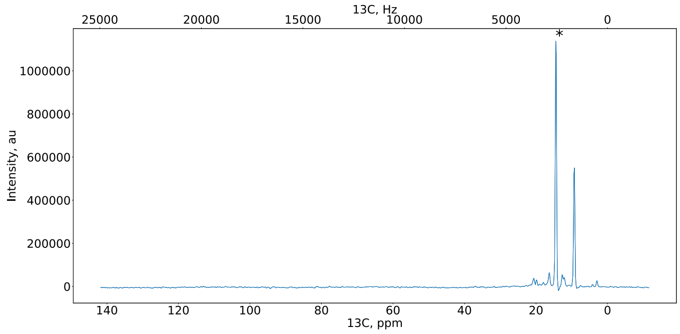

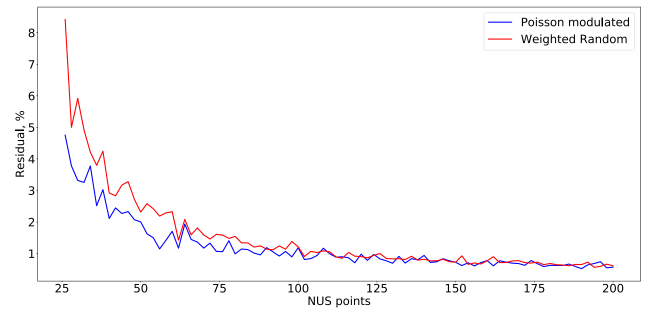

Peak:227

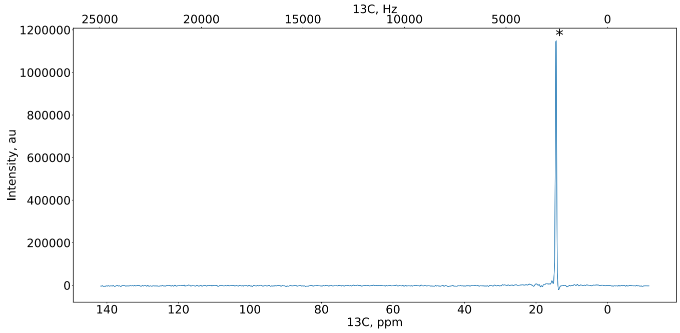

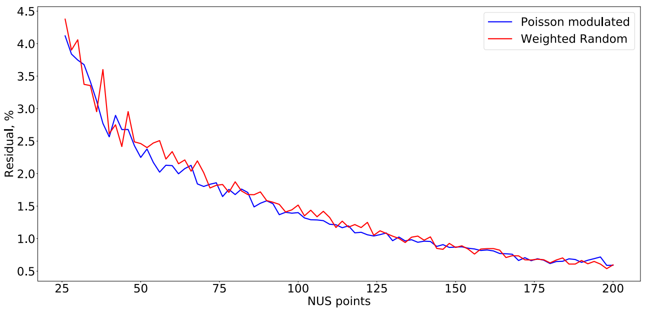

Peak:228

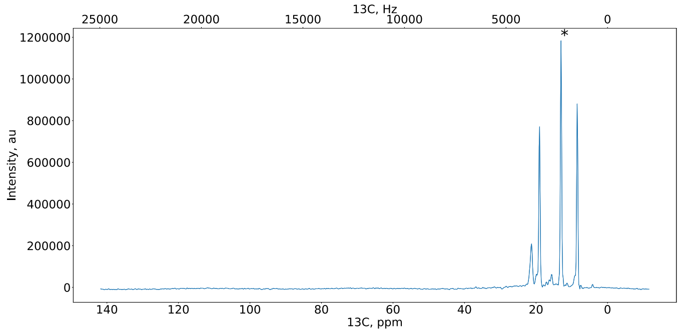

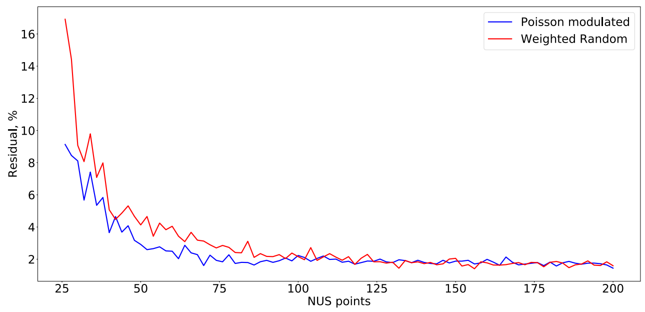

Peak:229

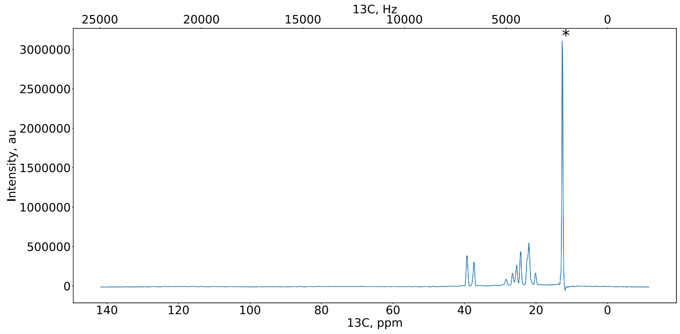

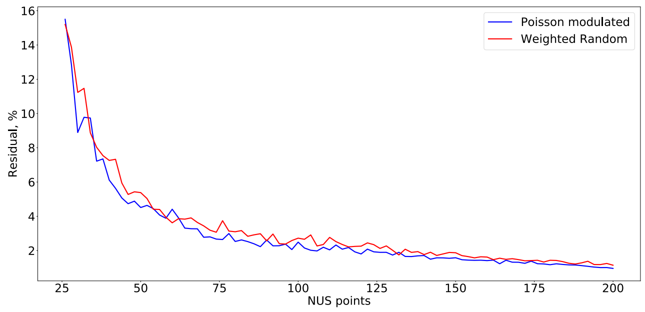

Peak:230

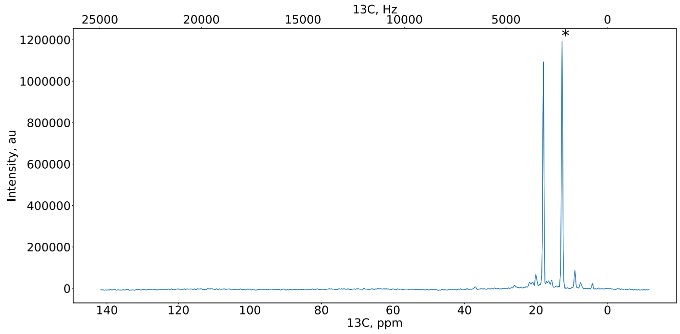

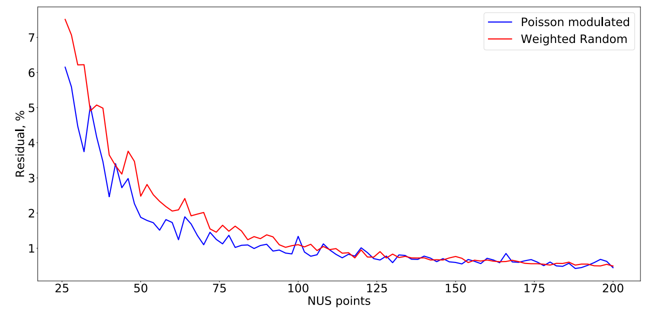

Peak:231

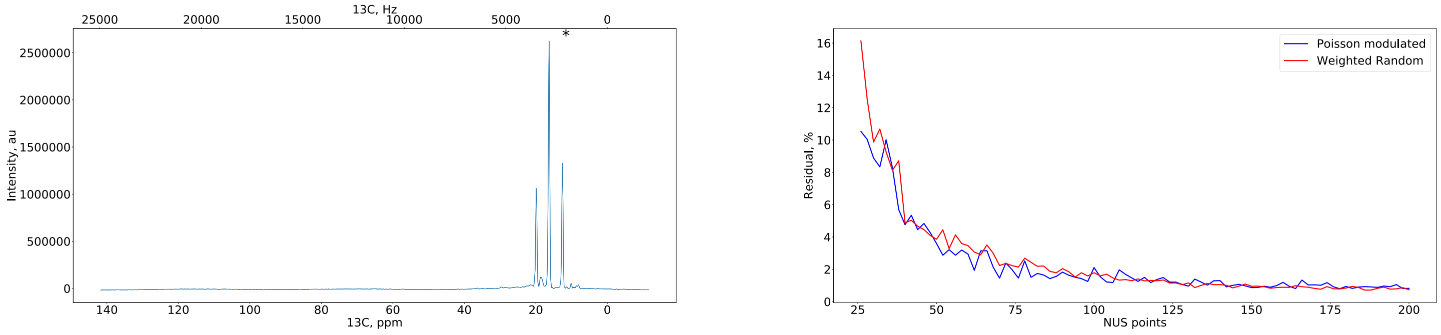

Peak:232

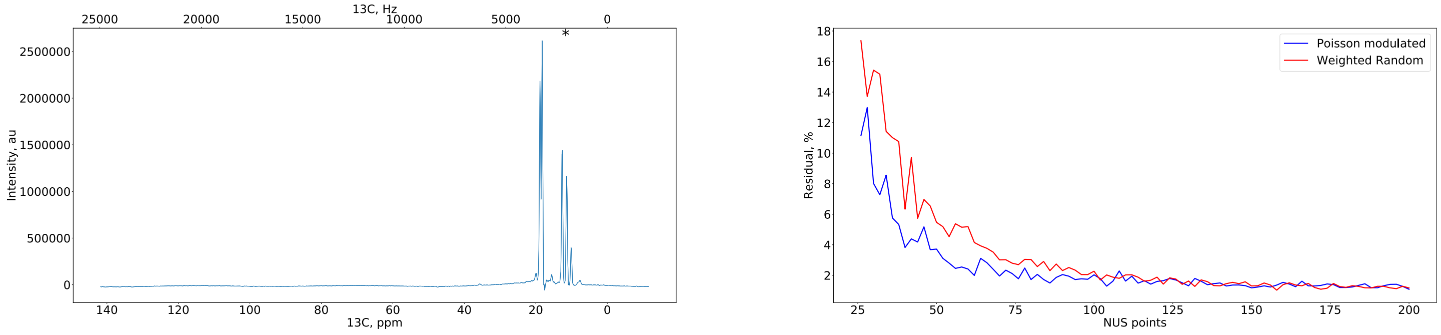

Peak:233

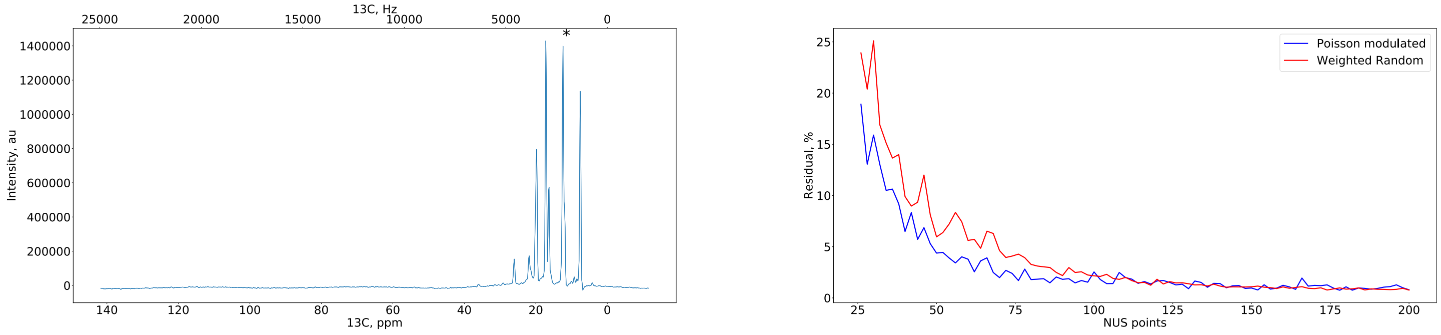

Peak:234

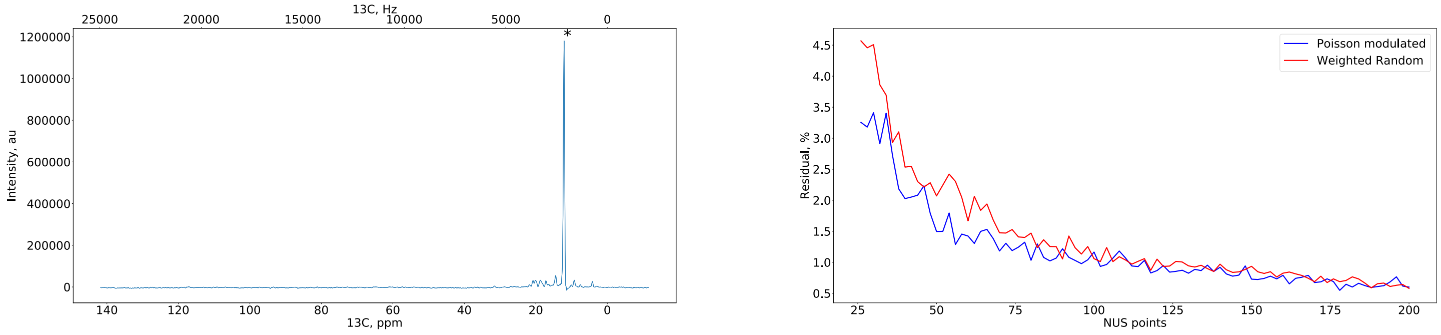

Peak:235

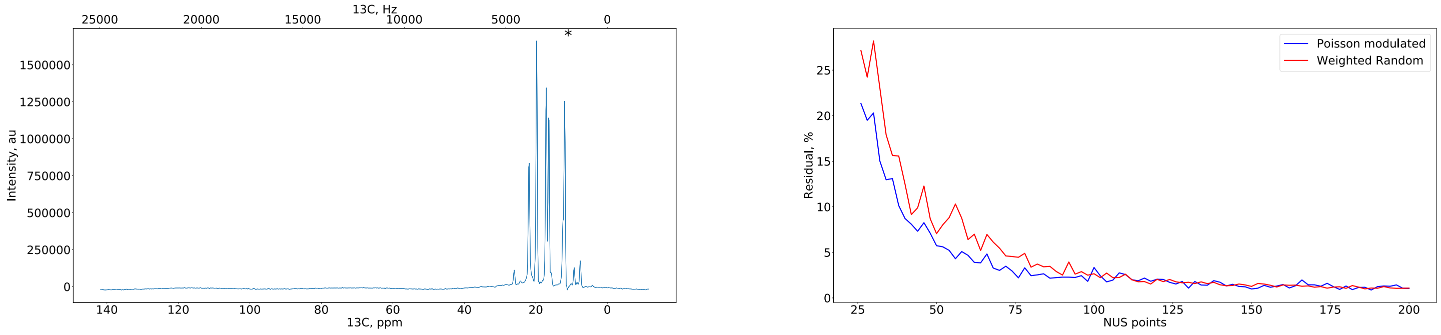

Peak:236

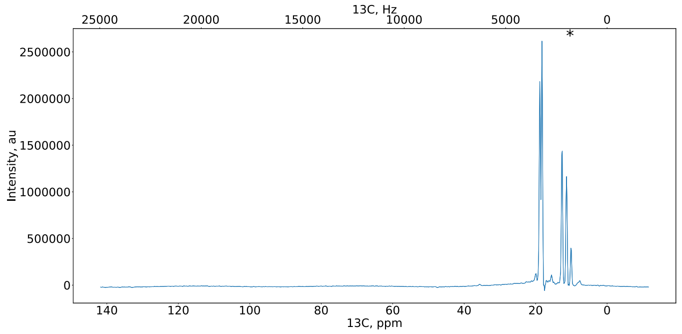

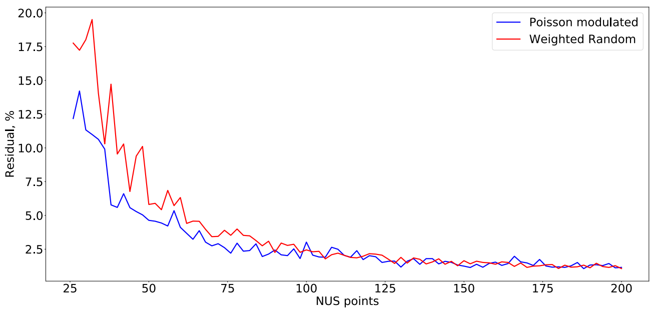

Peak:237

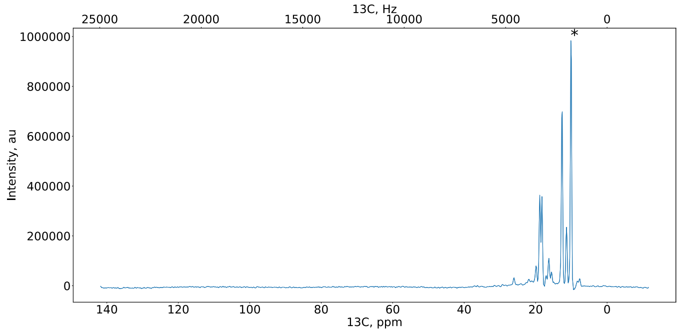

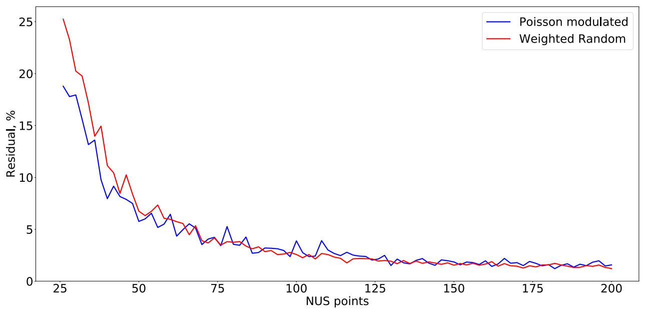

Peak:238

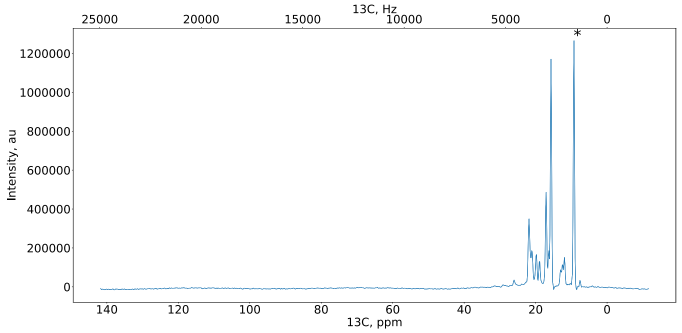

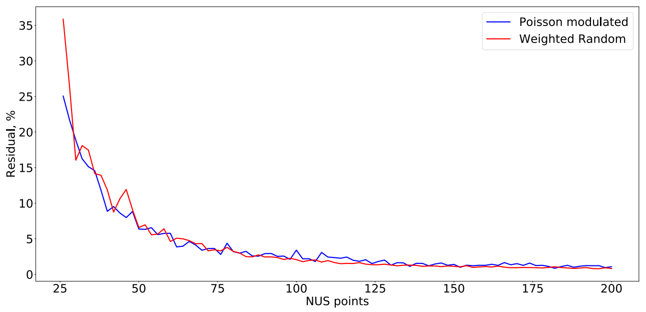

Peak:239

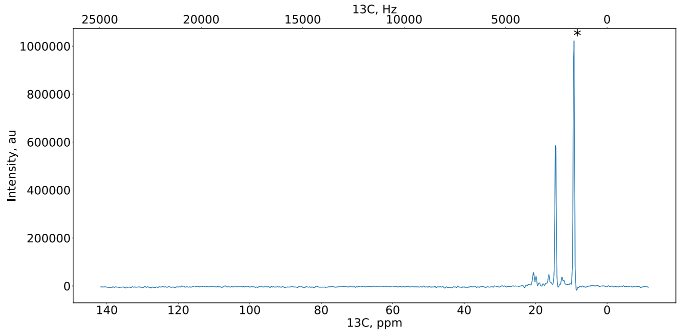

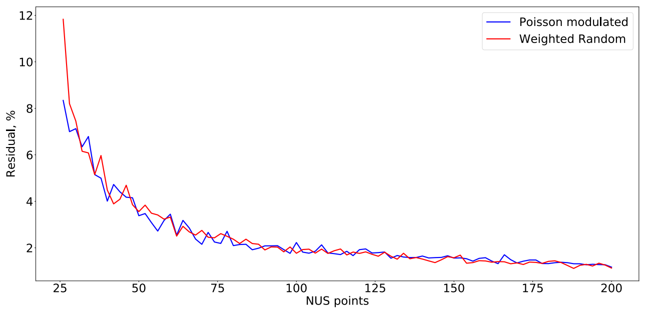

Peak:240

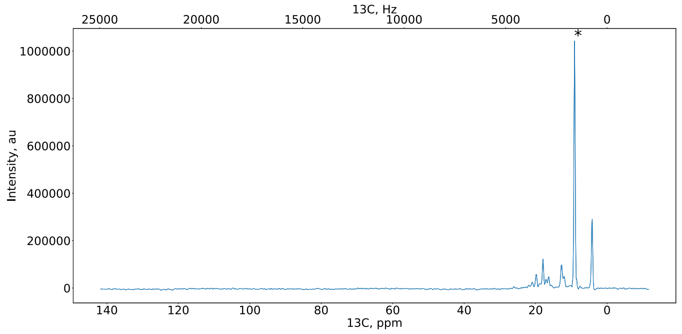

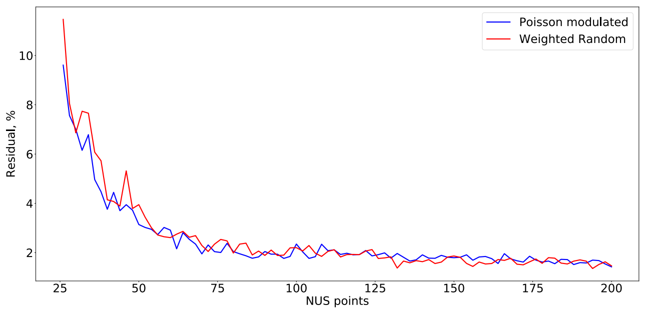

Peak:241

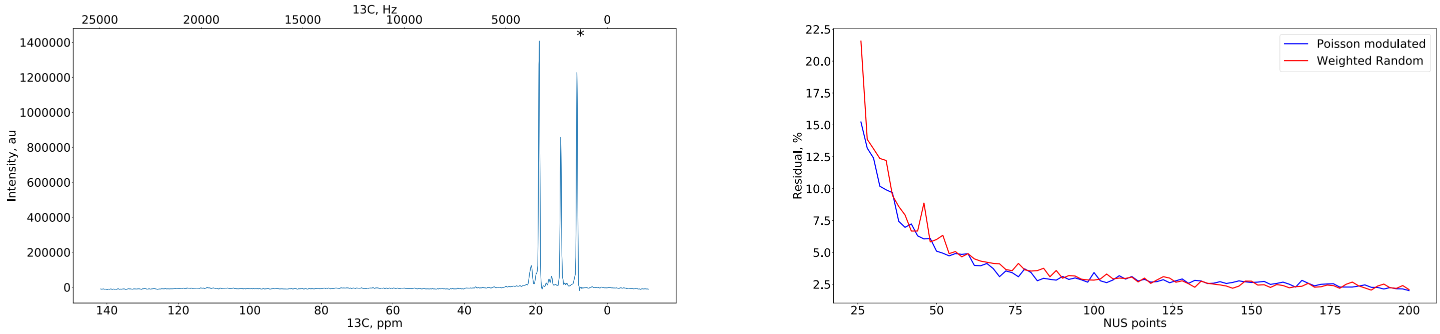

Peak:242

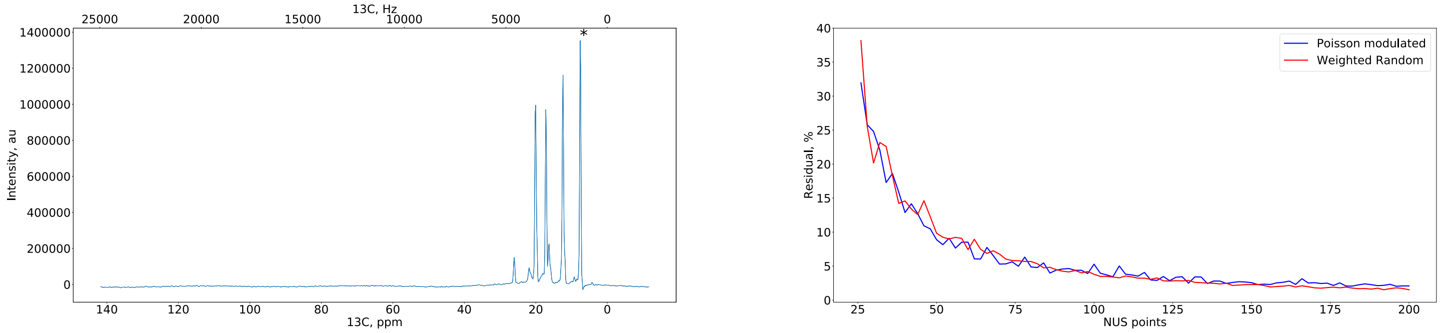

Peak:243

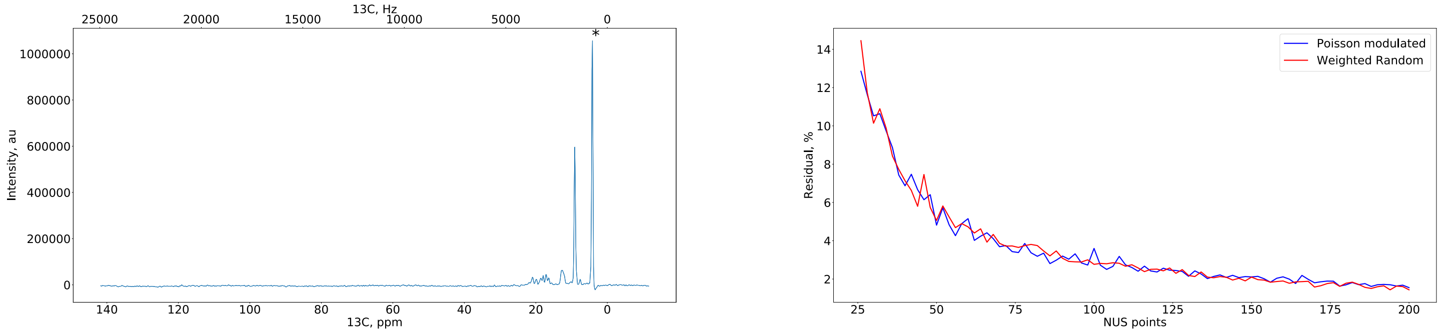

Peak:244

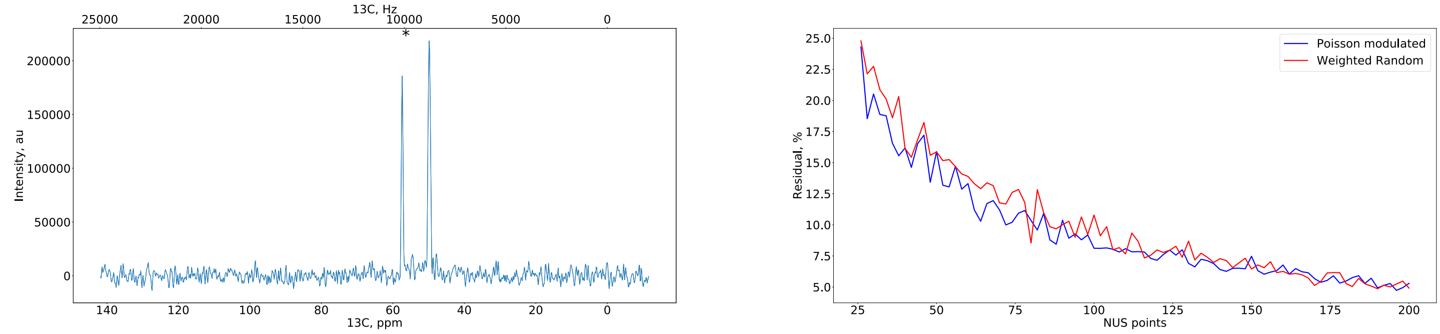

Peak:245

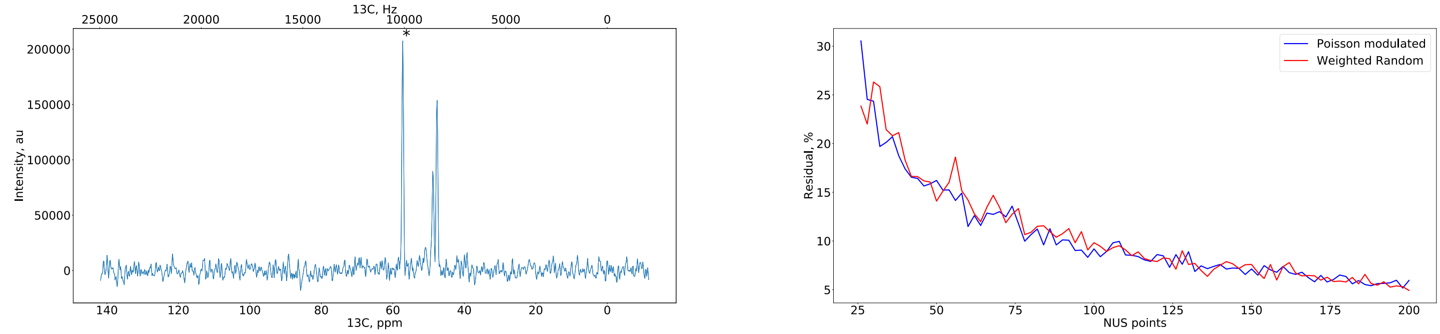

Peak:246

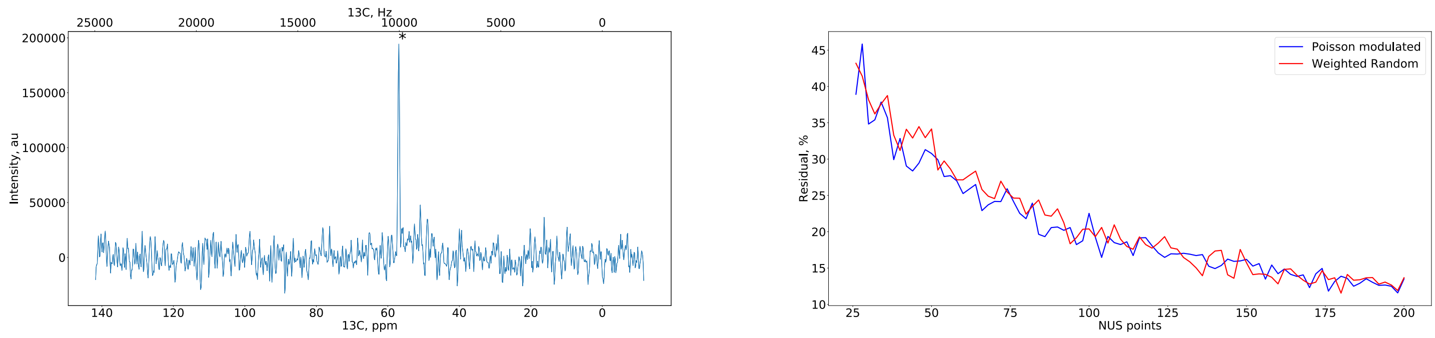

Peak:247

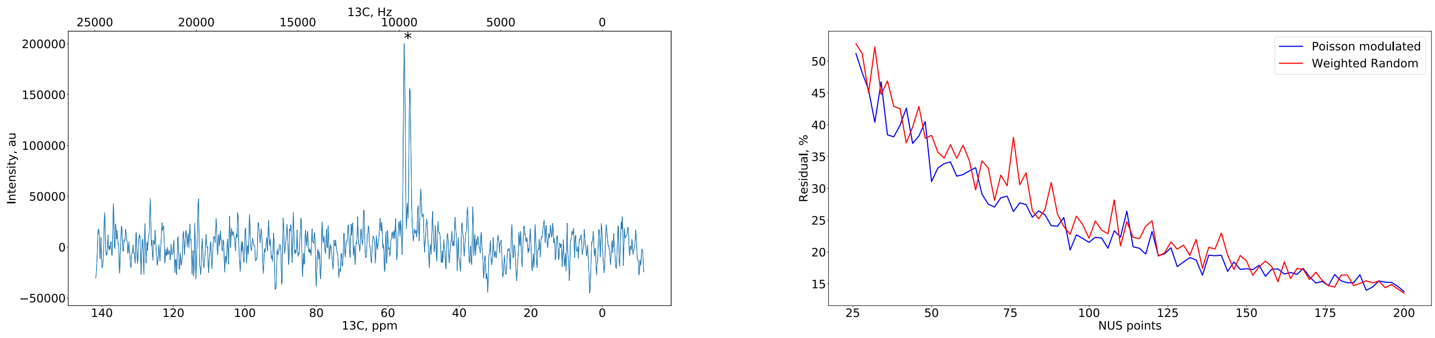

Peak:248

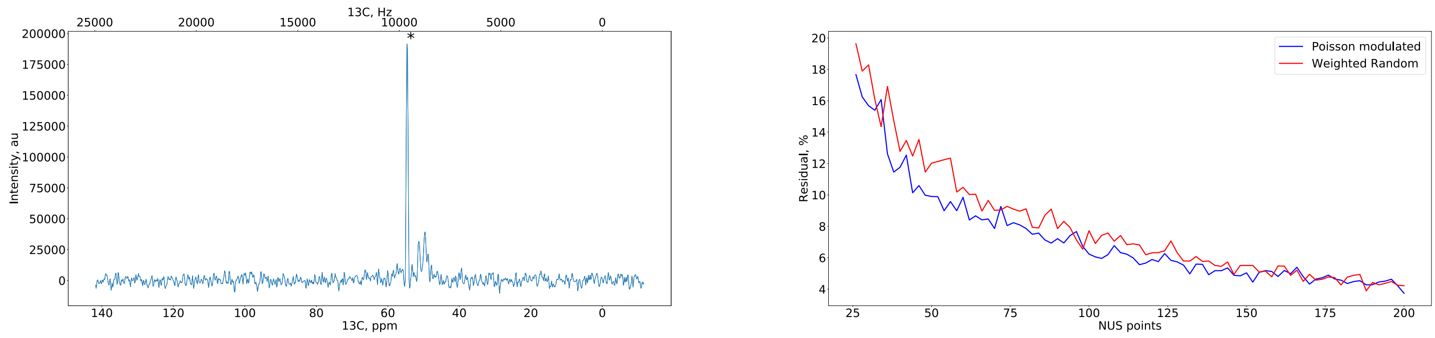

Peak:249

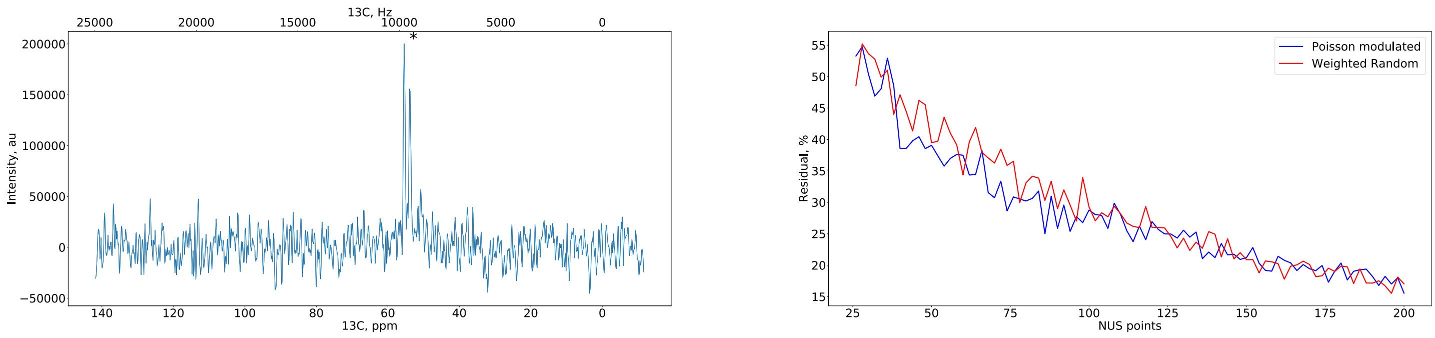

Peak:250

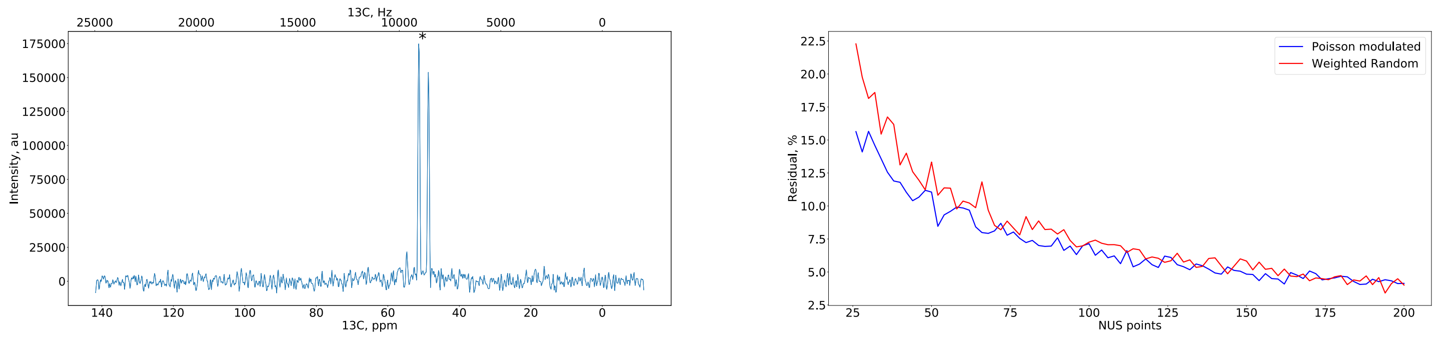

Peak:251

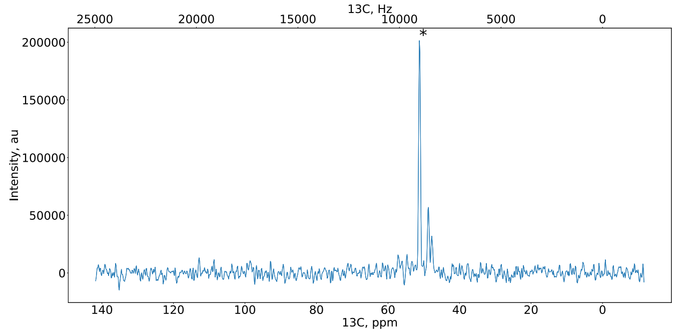

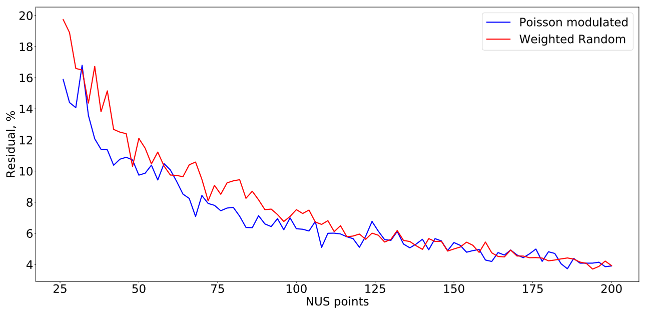

Peak:252

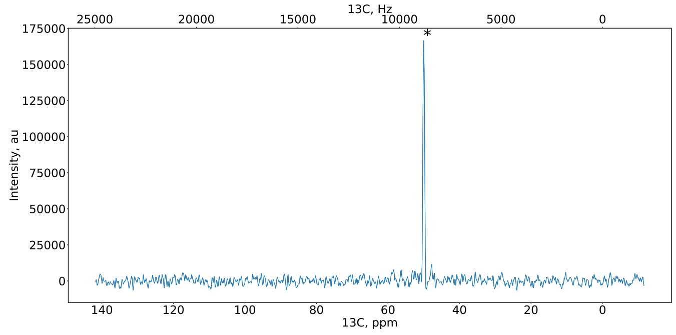

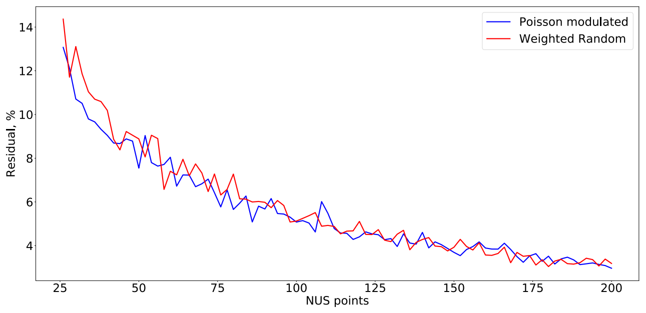

Peak:253

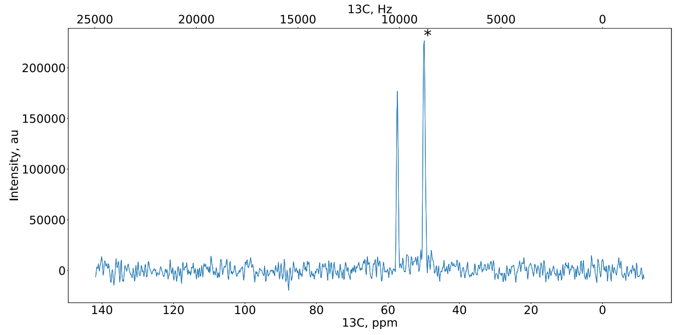

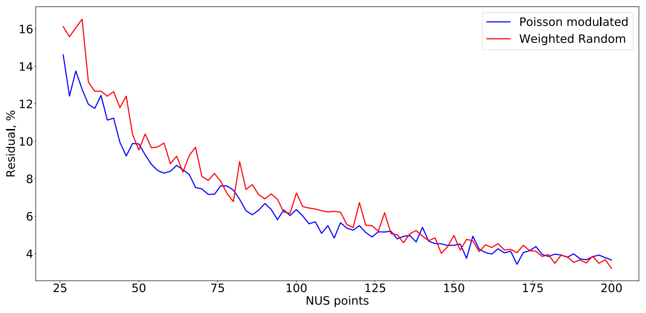

Peak:254

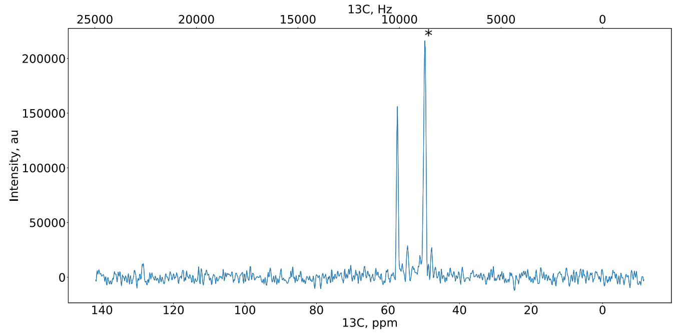

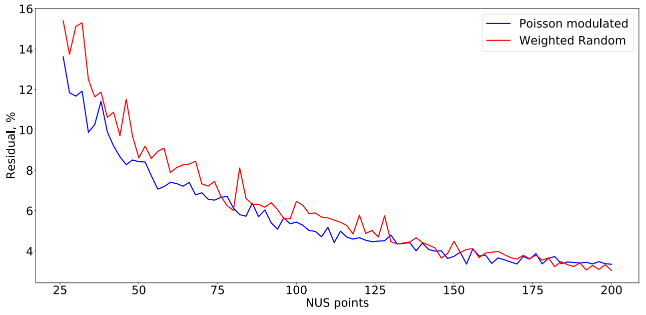

Peak:255

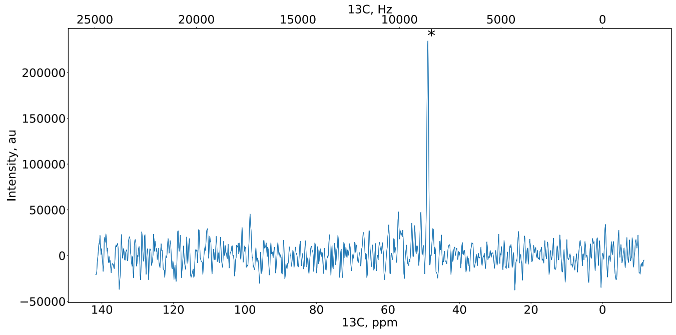

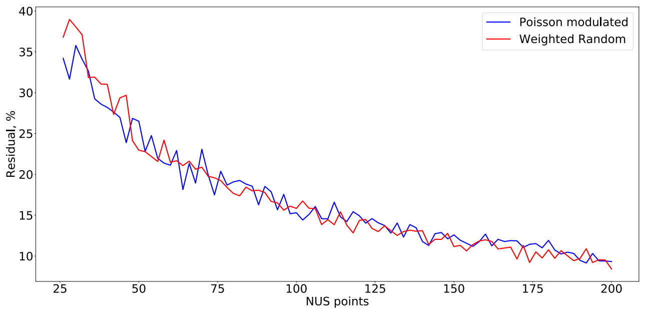

Peak:256

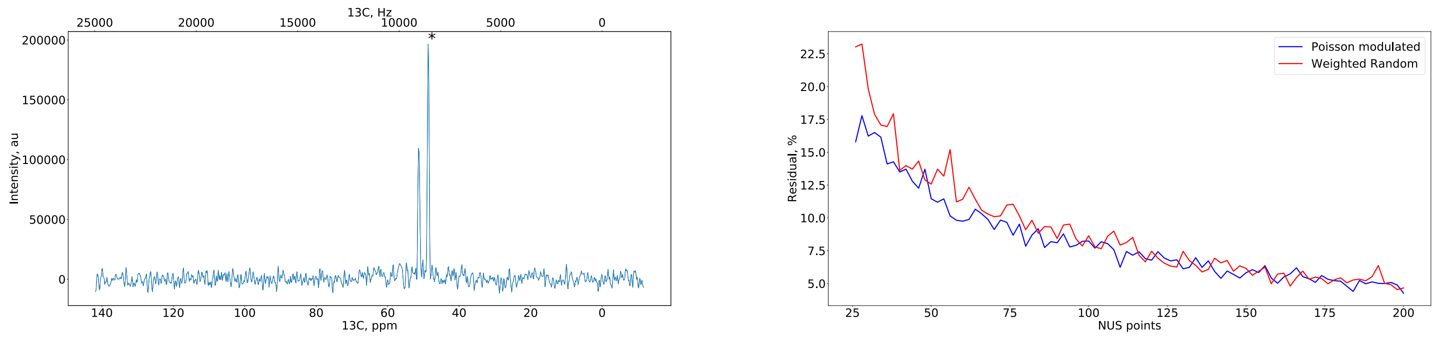

Peak:257

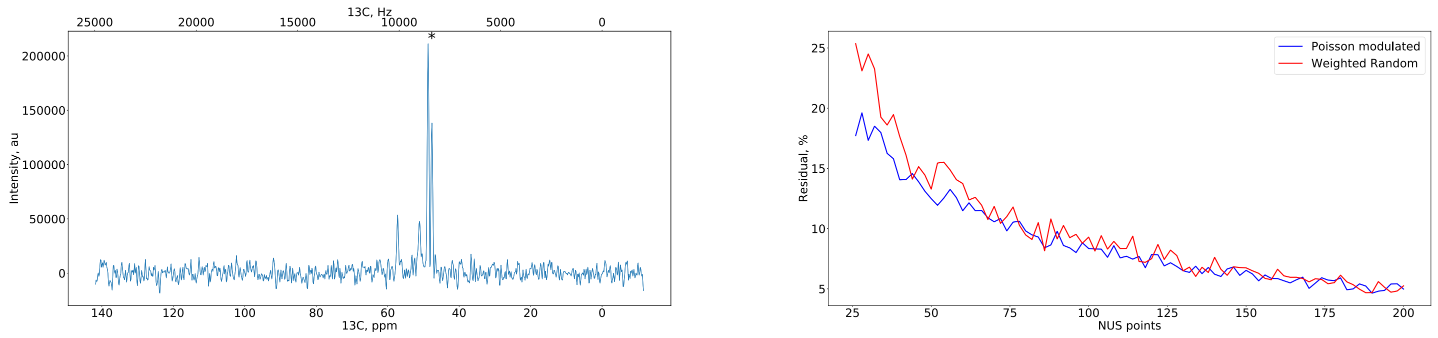

Peak:258

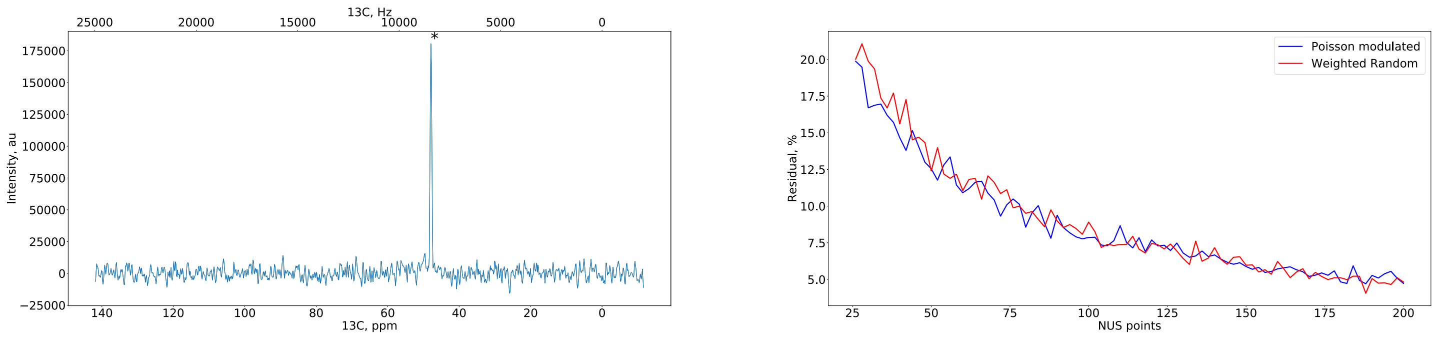

Peak:259

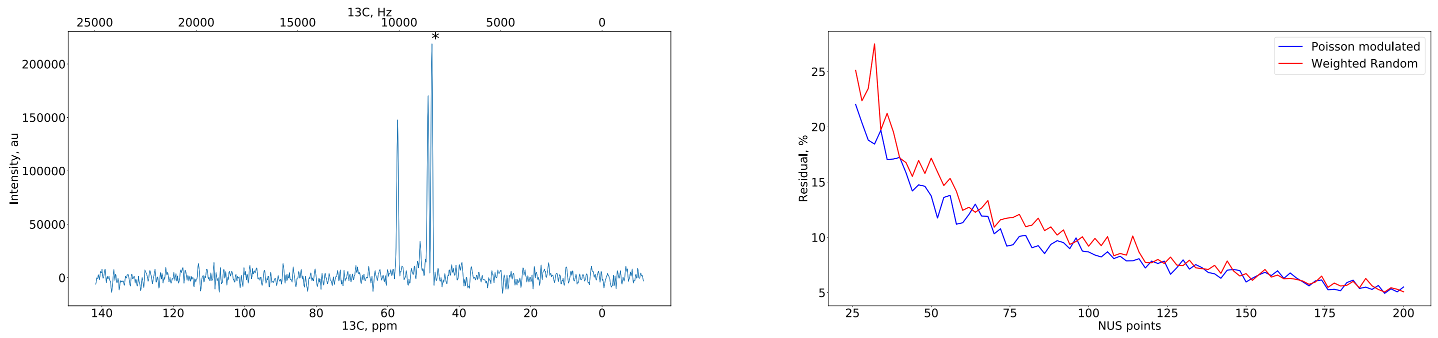

Peak:260

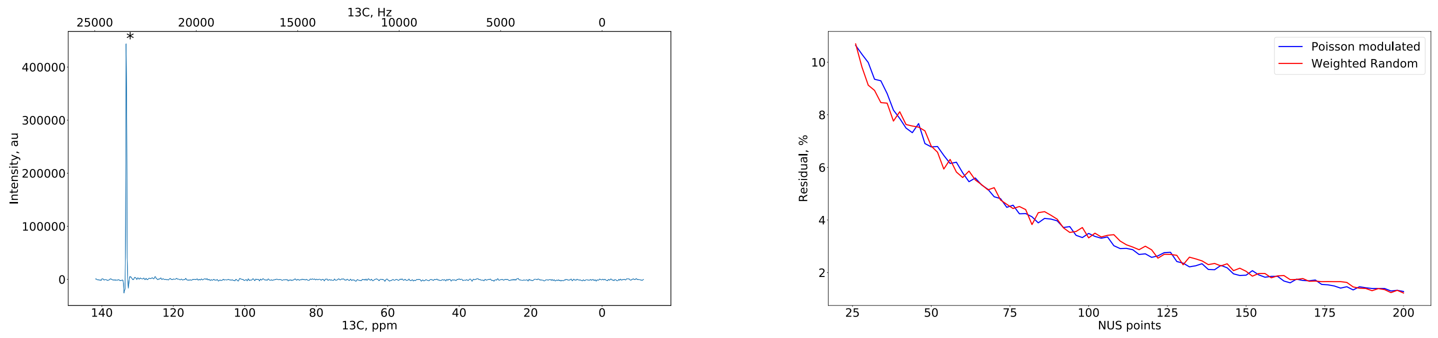

Peak:261

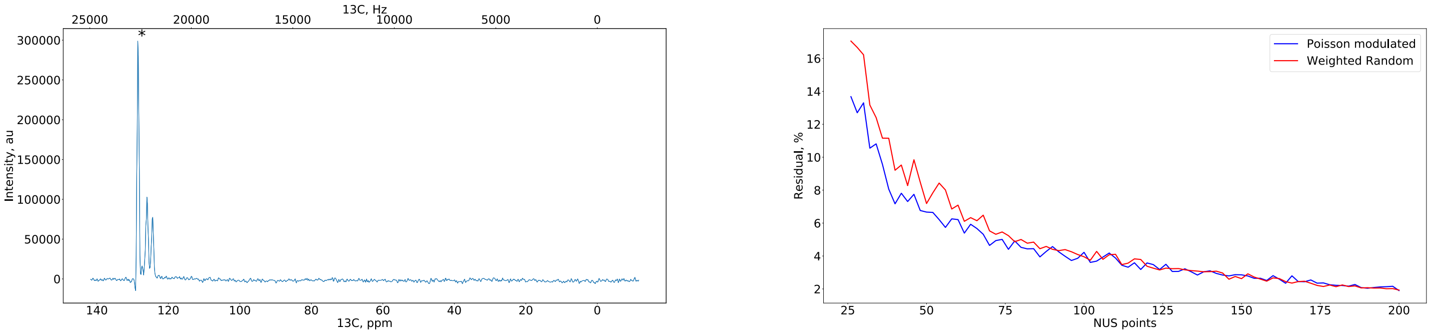

Peak:262

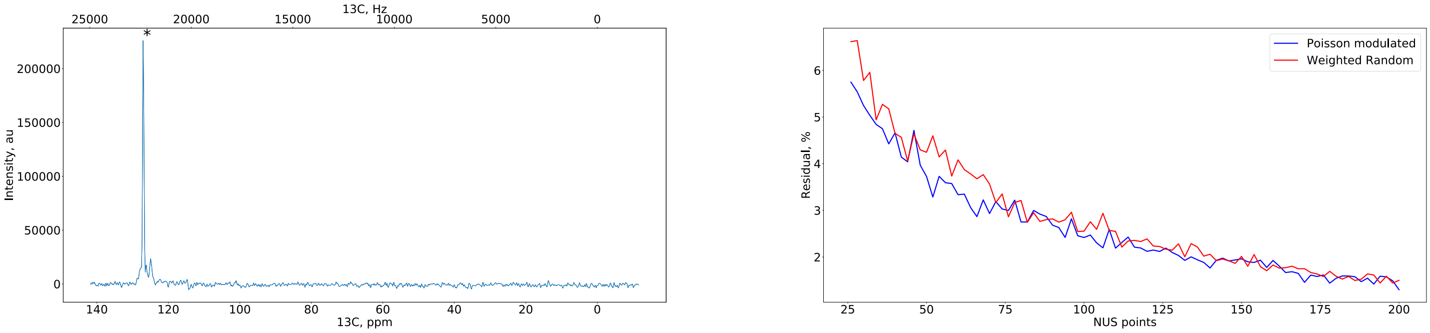

Peak:263

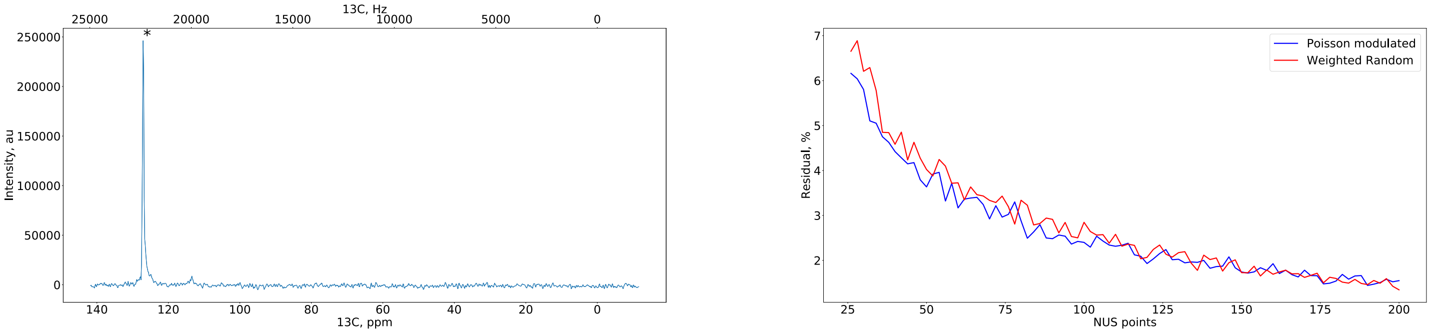

Peak:264

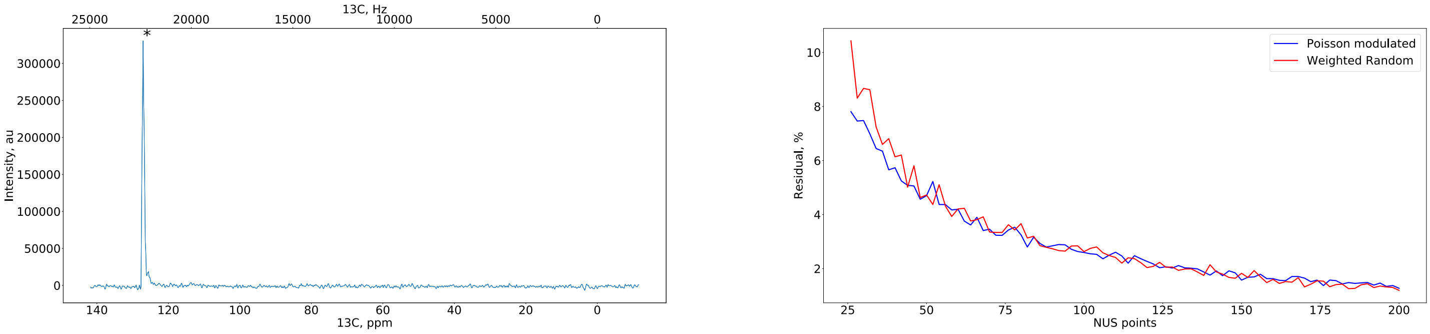

Peak:265

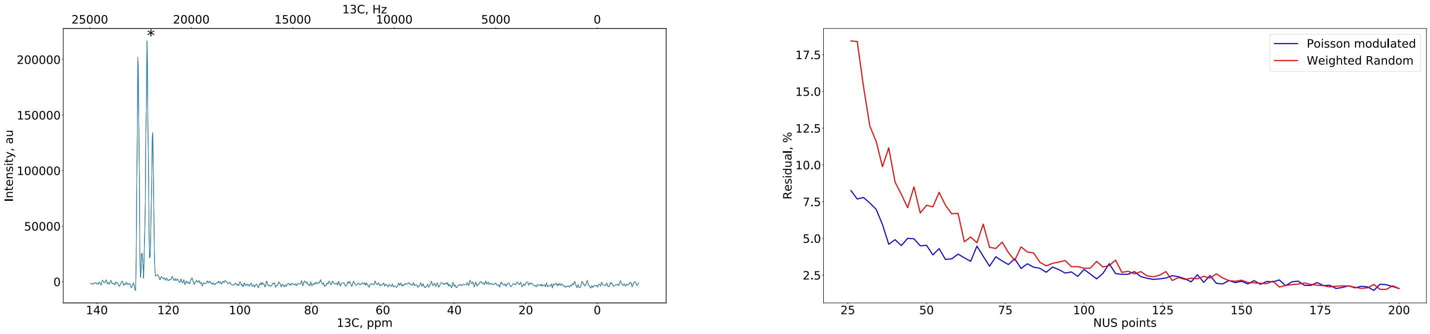

Peak:266

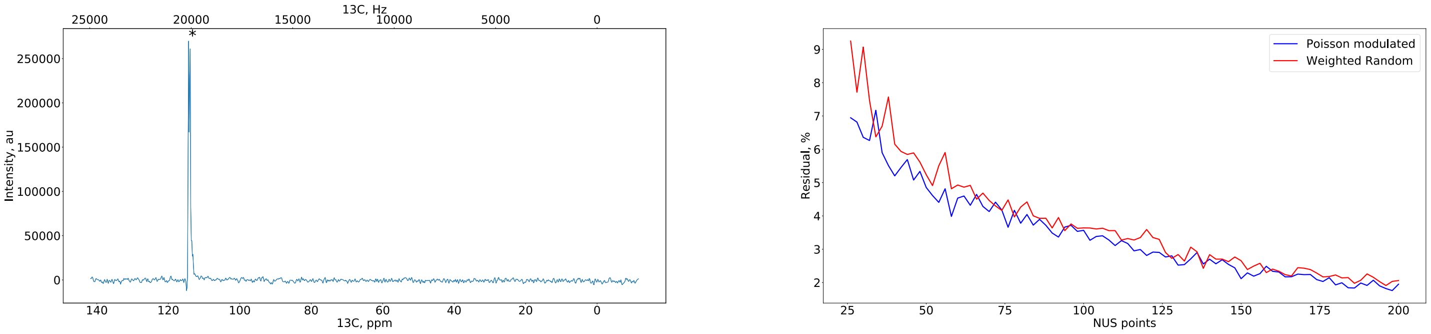

Peak:267

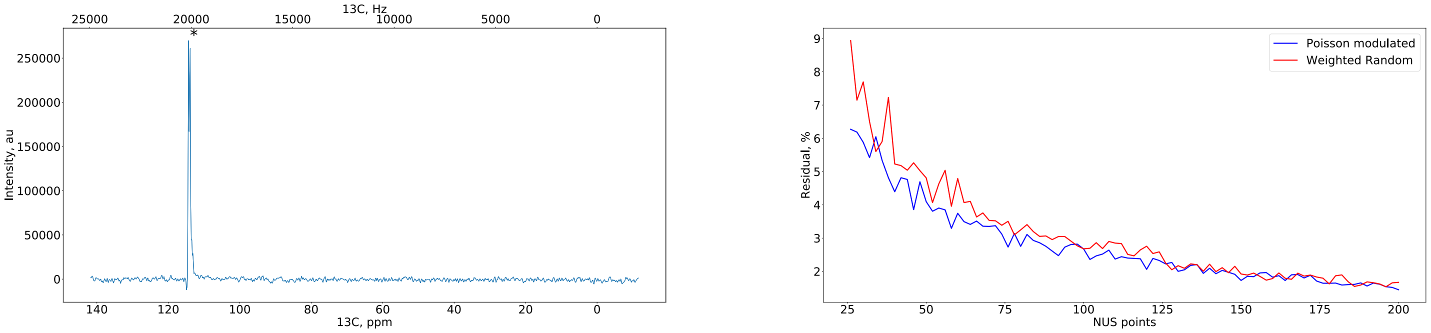

Peak:268

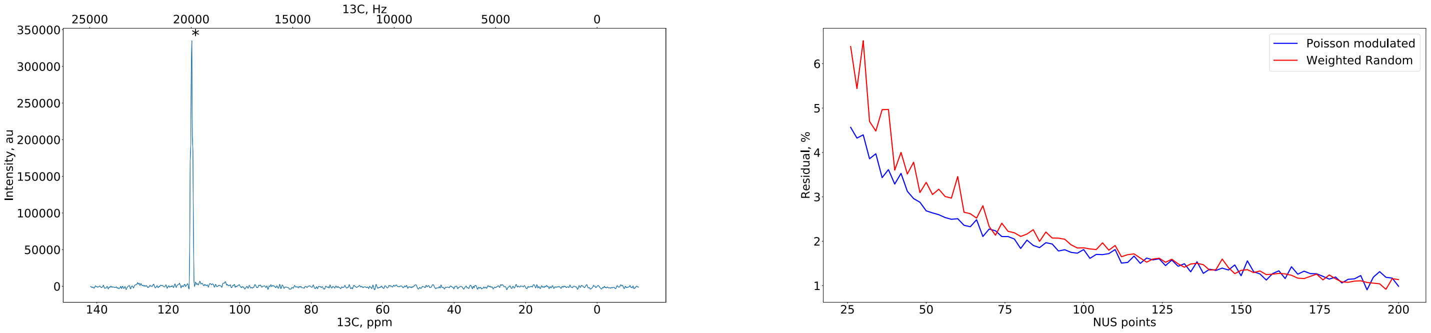

Supplement: Supplementary file 2 — Supplementary file2 (PDF 14484 kb) [file 10858_2021_385_MOESM2_ESM.pdf]
