# Supplementary material for: Clustered sparsity and Poisson-gap sampling
Source: J Biomol NMR. 2021 Nov 5;75(10-12):401–16. doi: 10.1007/s10858-021-00385-7 (PMC8642362; doi:10.1007/s10858-021-00385-7)

Supplementary Information:
Clustered sparsity and Poisson-gap sampling

Paweł Kasprzak[1,2], Mateusz Urbańczyk[1,3], and Krzysztof Kazimierczuk[1]

[1]Centre of New Technologies, University of Warsaw, Banacha 2C, 02-097 Warsaw, Poland
[2]Faculty of Physics, University of Warsaw, Pasteura 5, 02-093 Warsaw, Poland
[3]Institute of Physical Chemistry, Polish Academy of Sciences, Kasprzaka 44/52,01-224 Warsaw, Poland

Analysis of HNCACB spectrum of azurin

Peak1

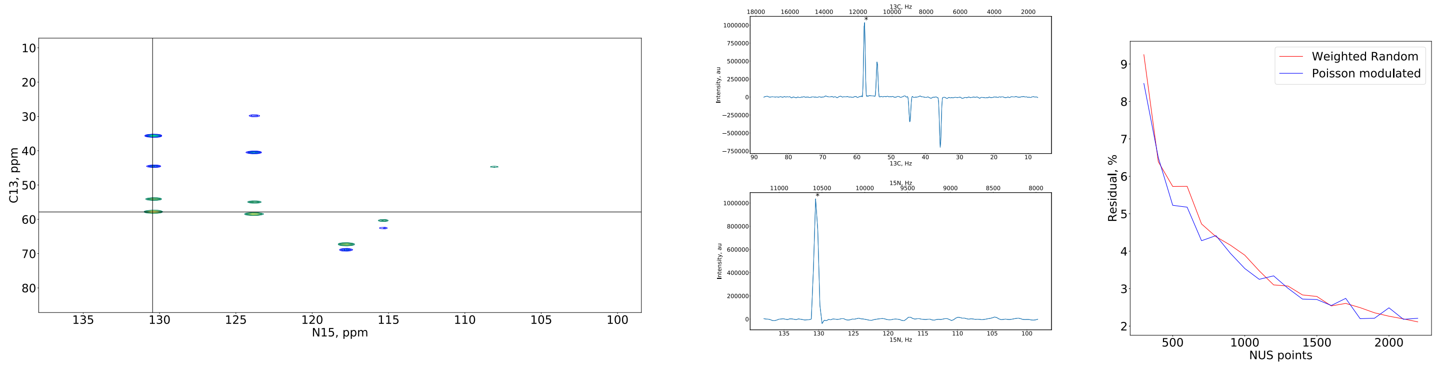

Peak2

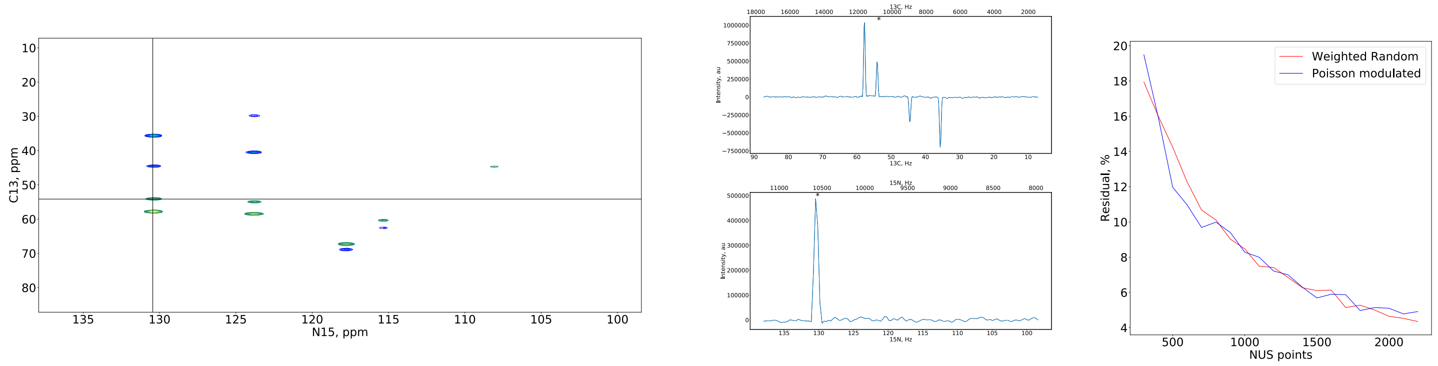

Peak3

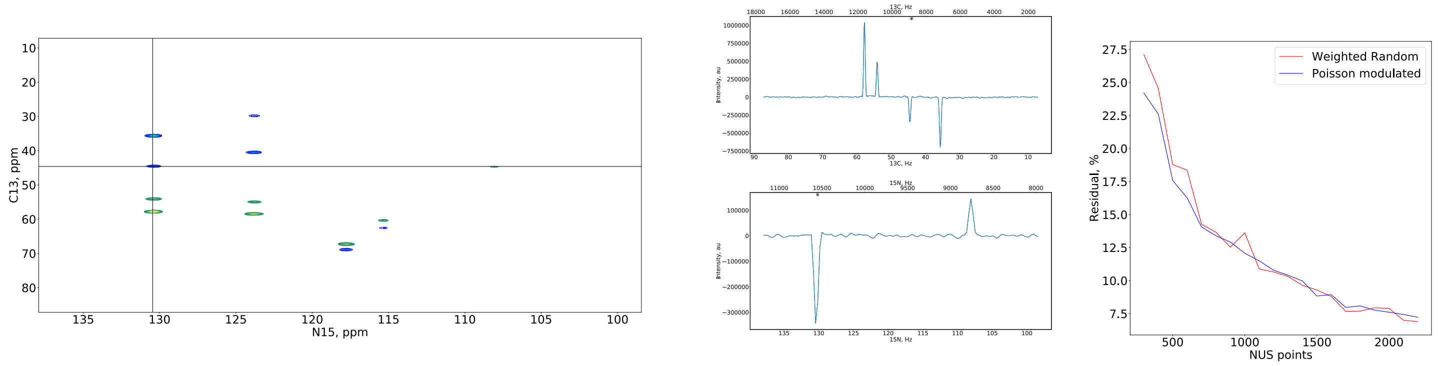

Peak4

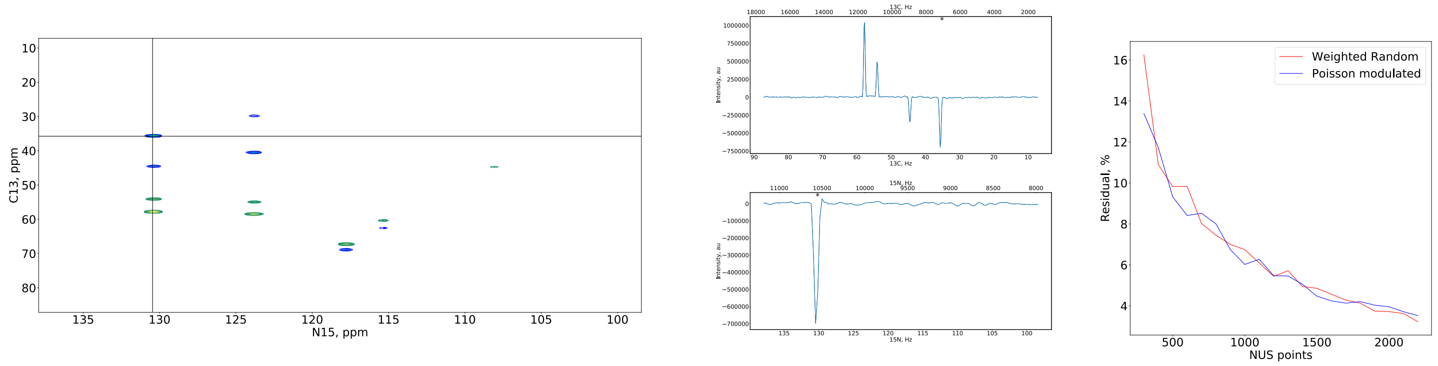

Peak5

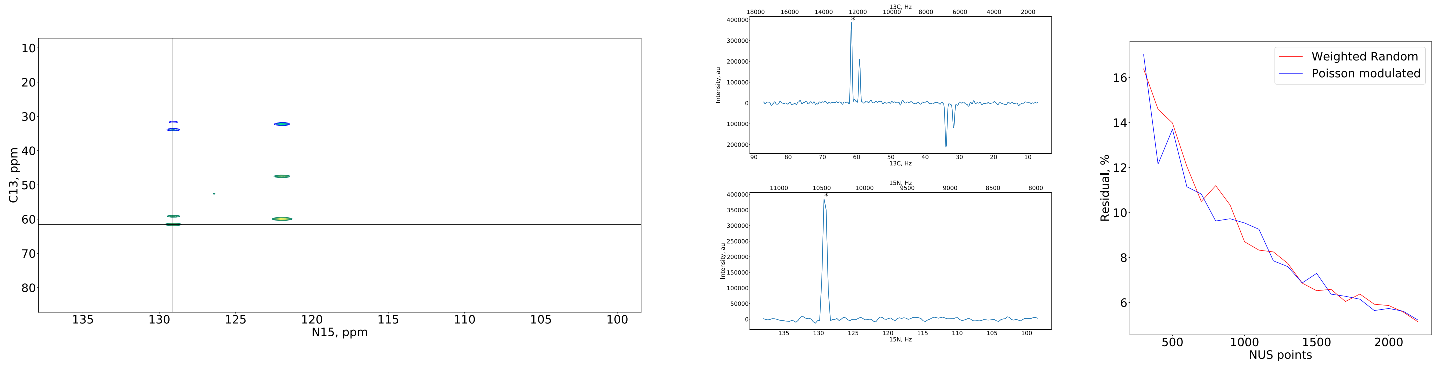

Peak6

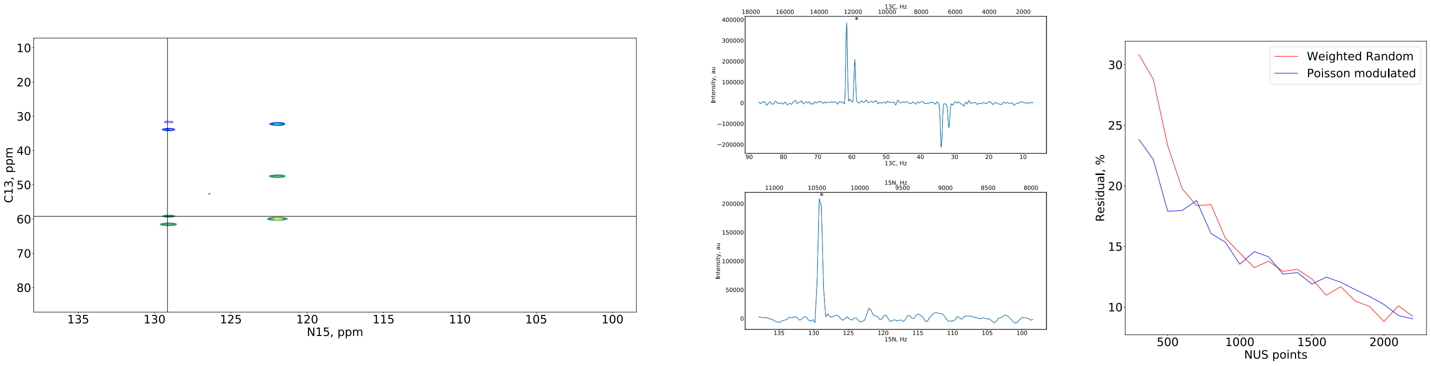

Peak7

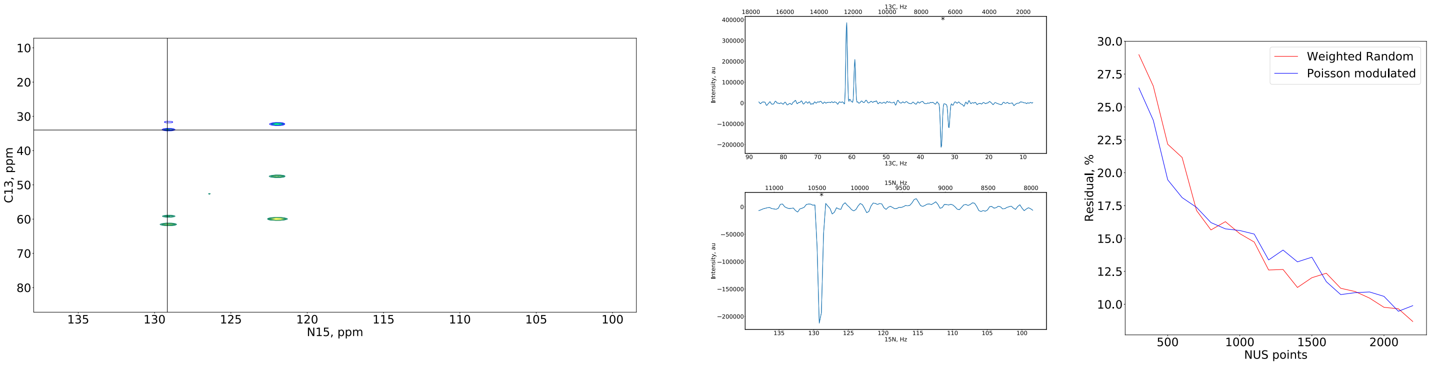

Peak8

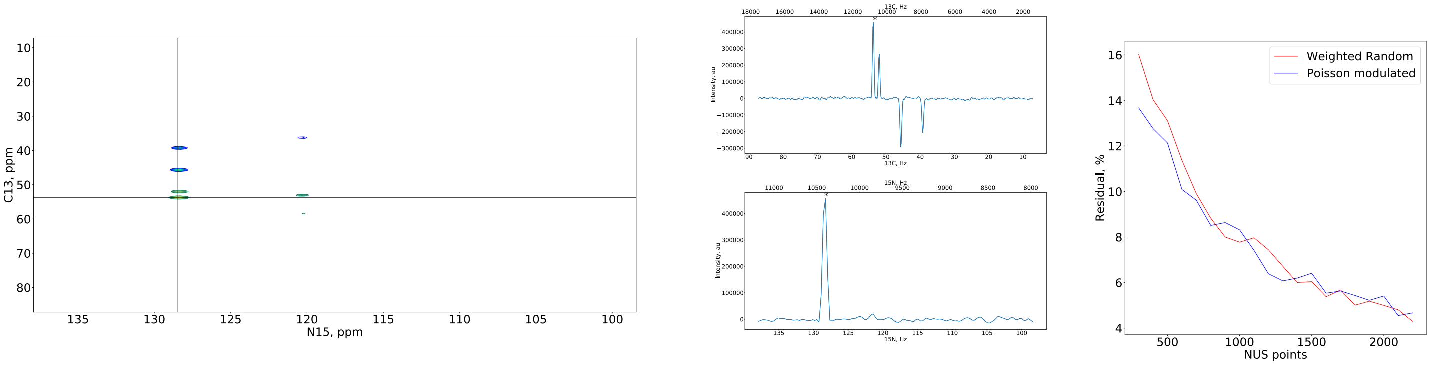

Peak9

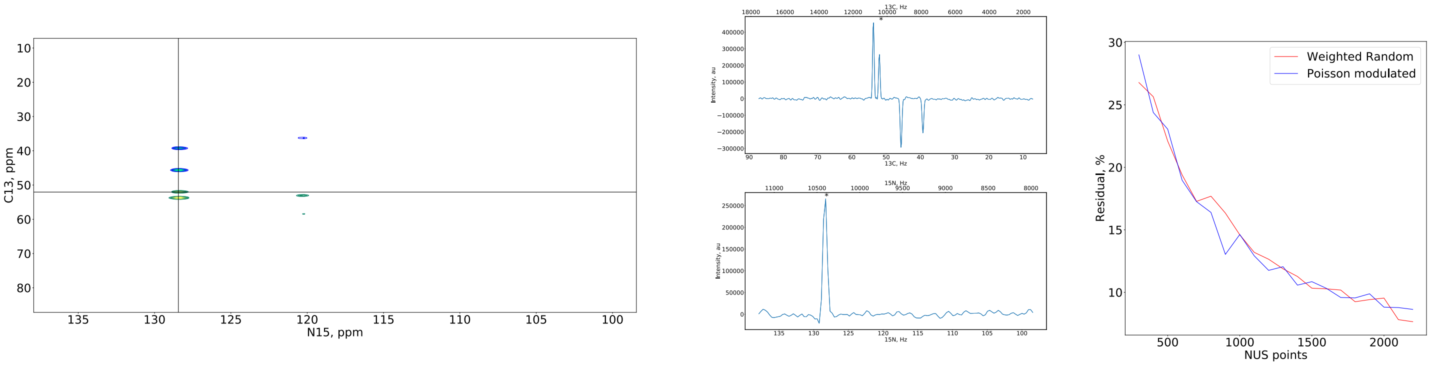

Peak10

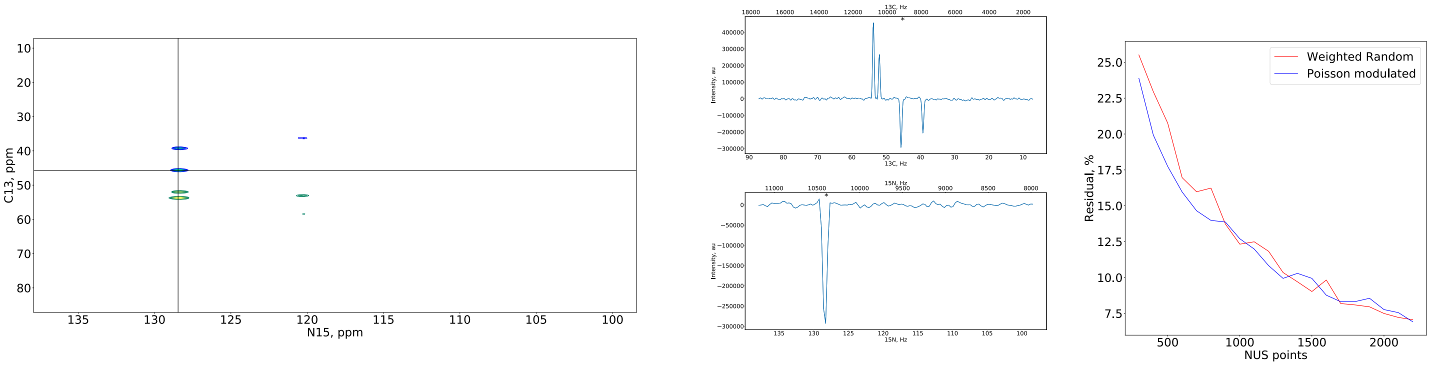

Peak11

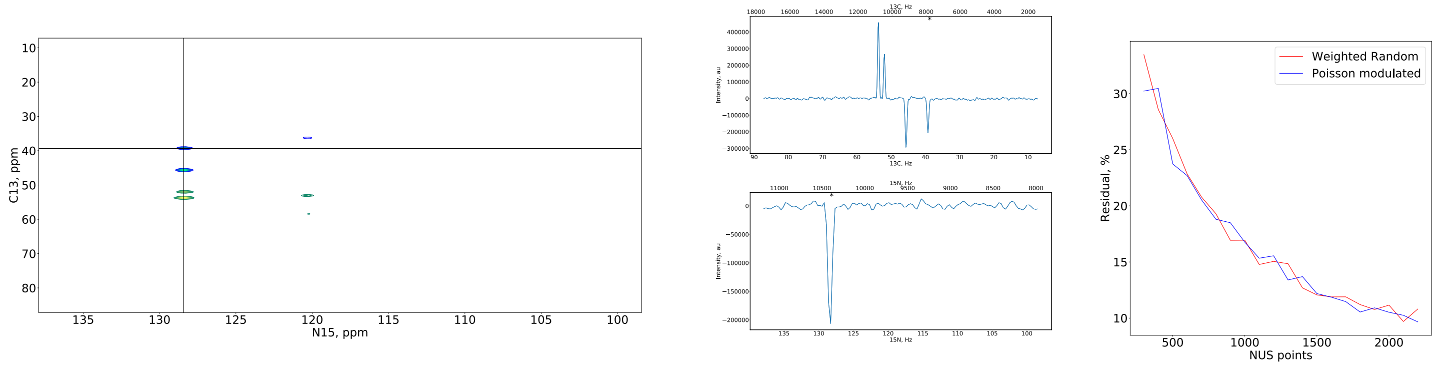

Peak12

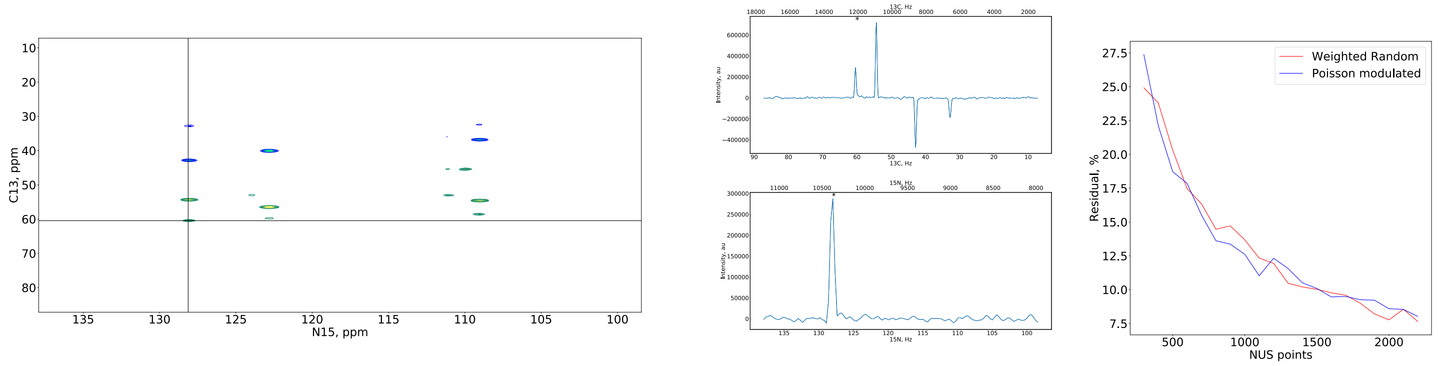

Peak13

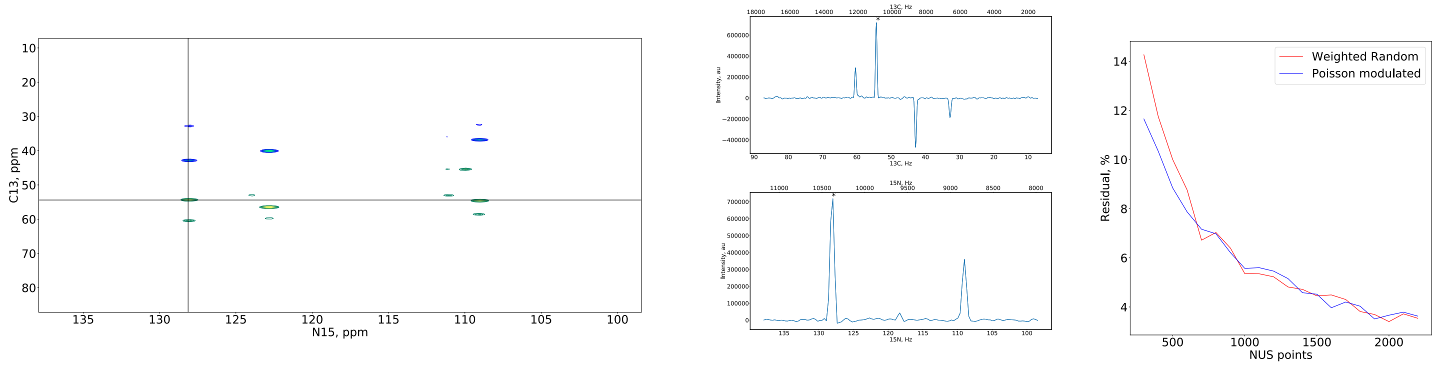

Peak14

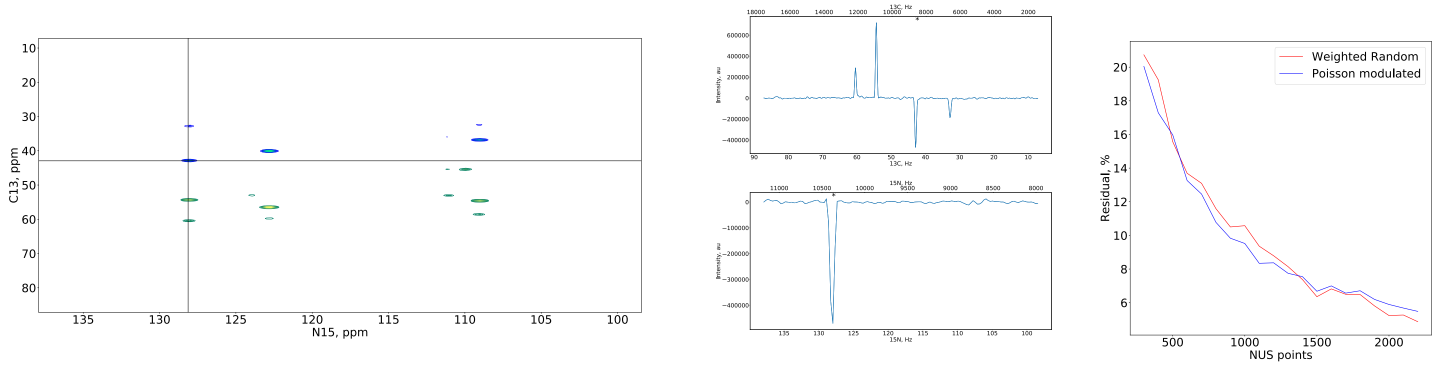

Peak15

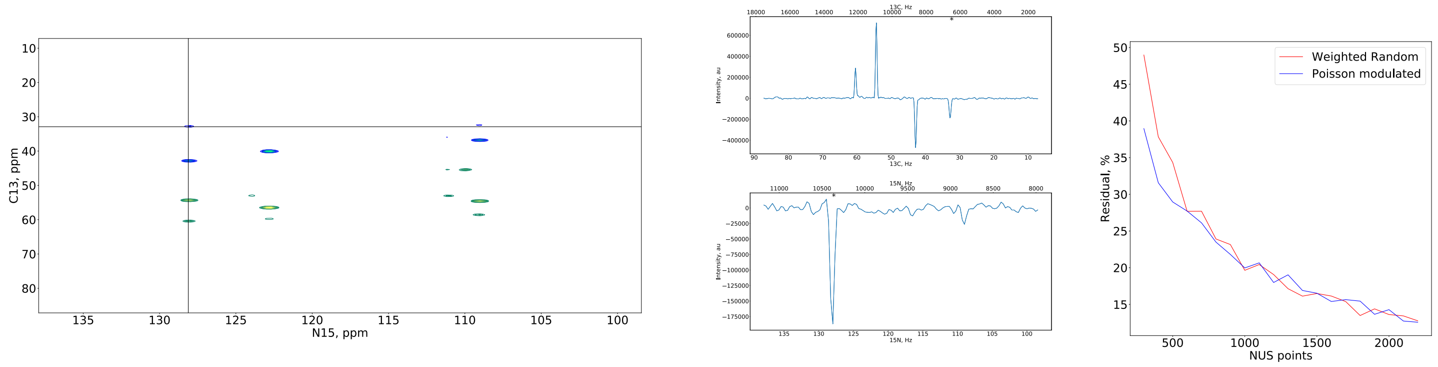

Peak16

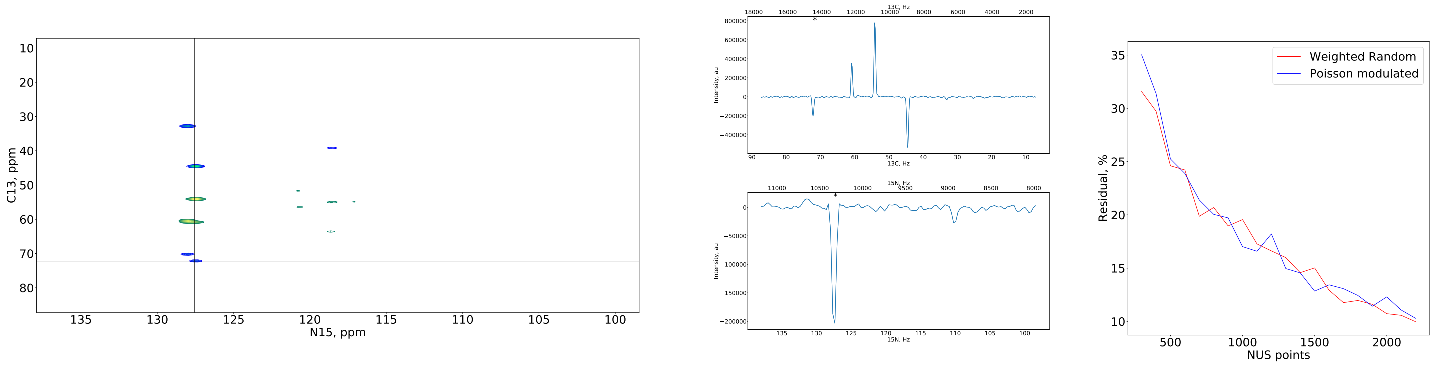

Peak17

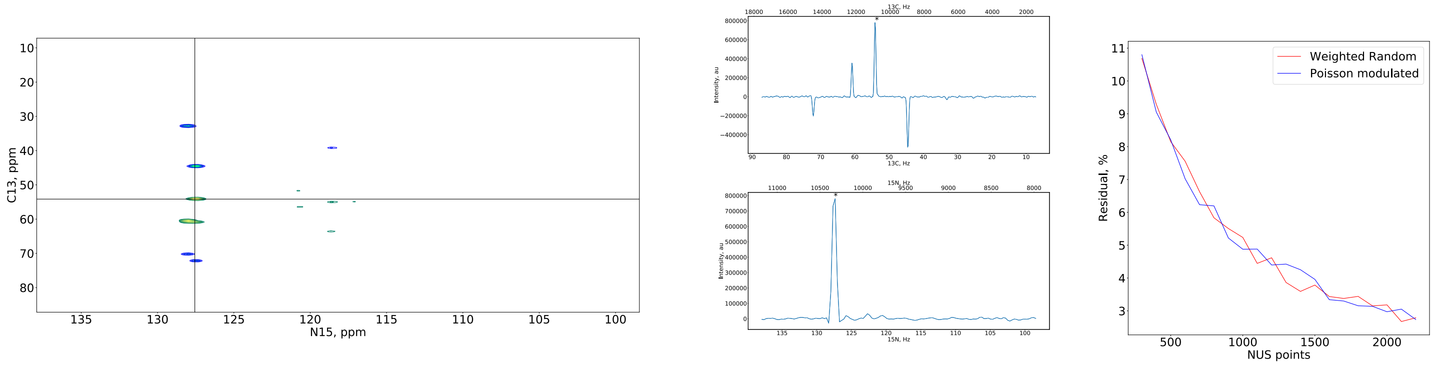

Peak18

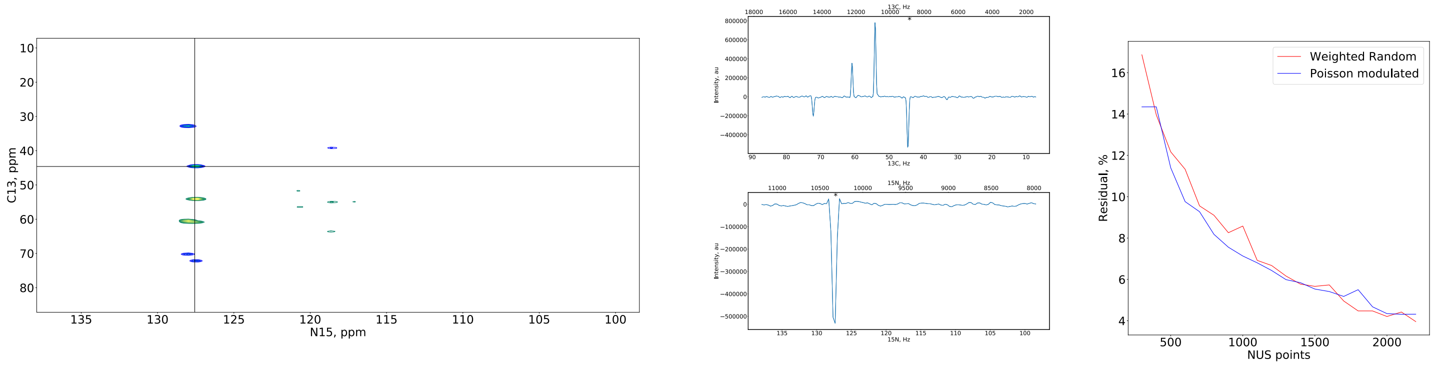

Peak19

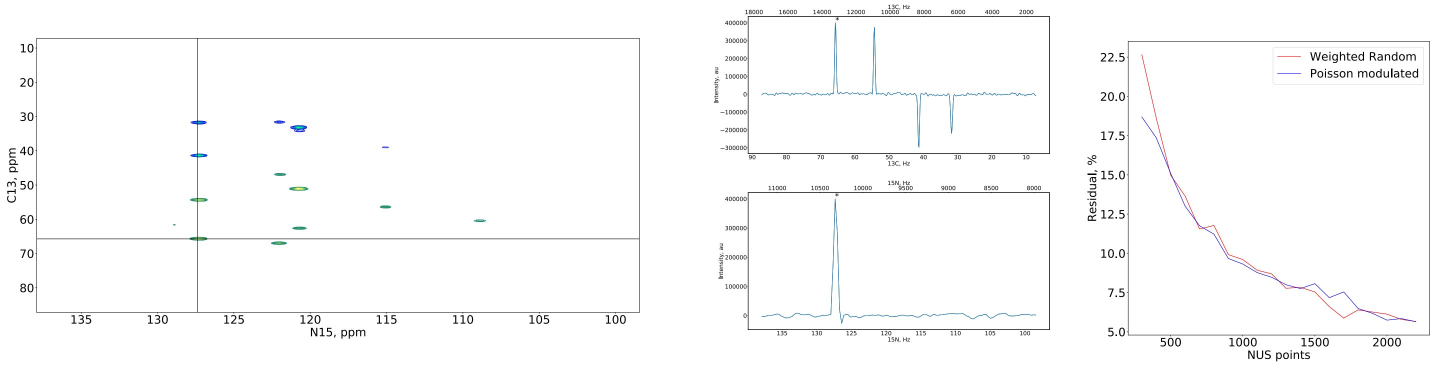

Peak20

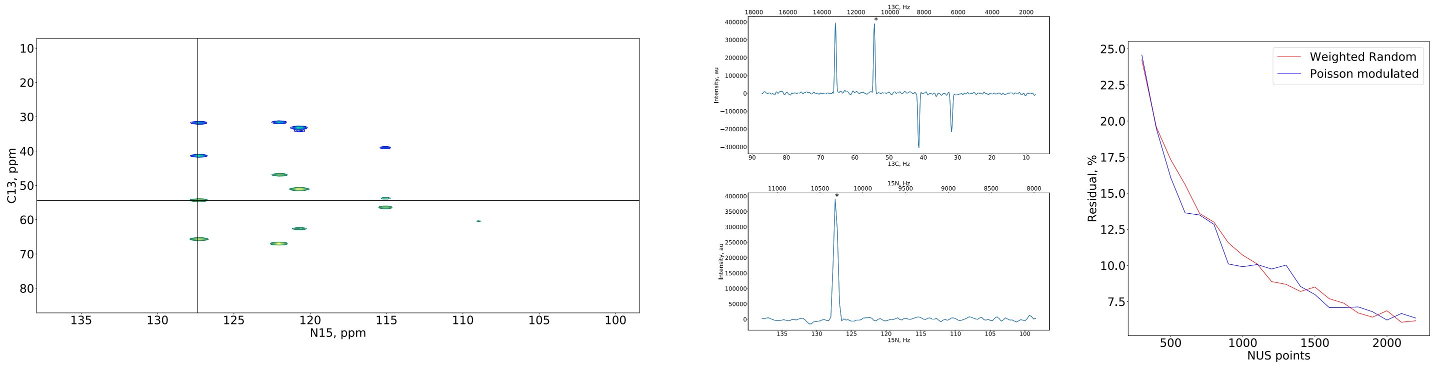

Peak21

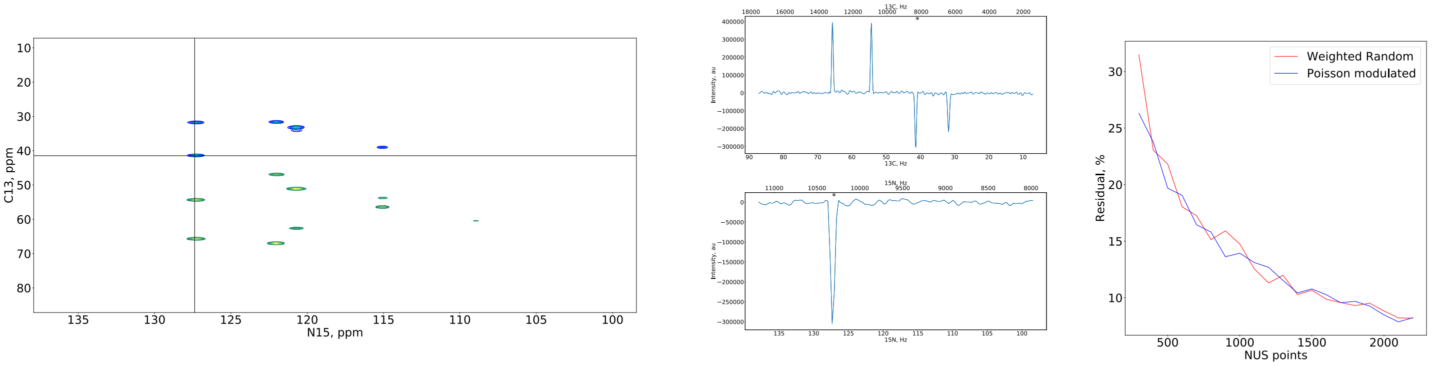

Peak22

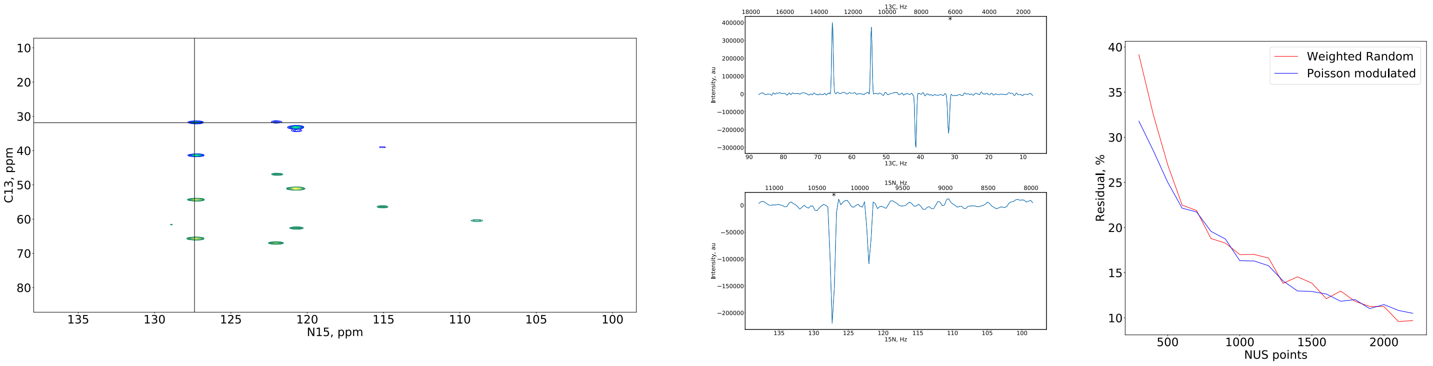

Peak23

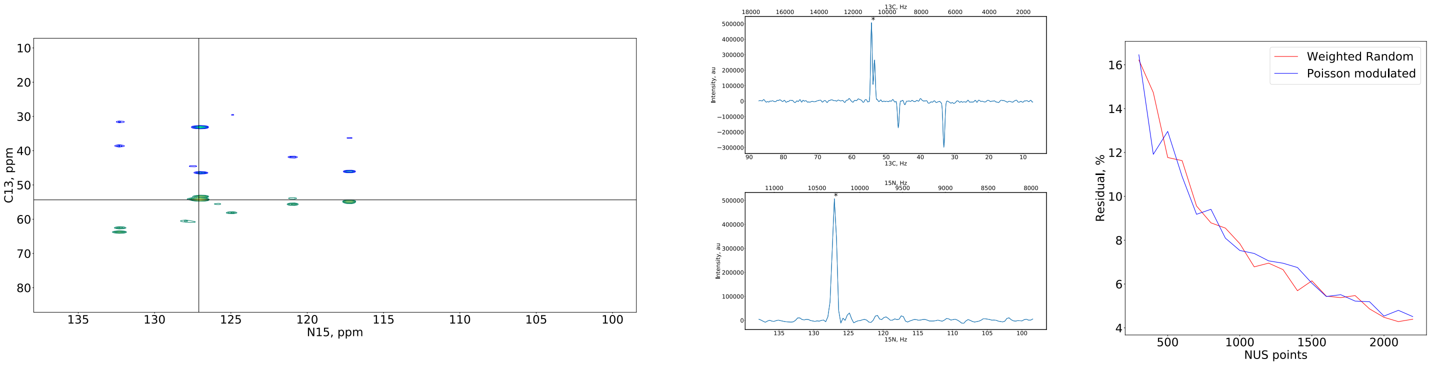

Peak24

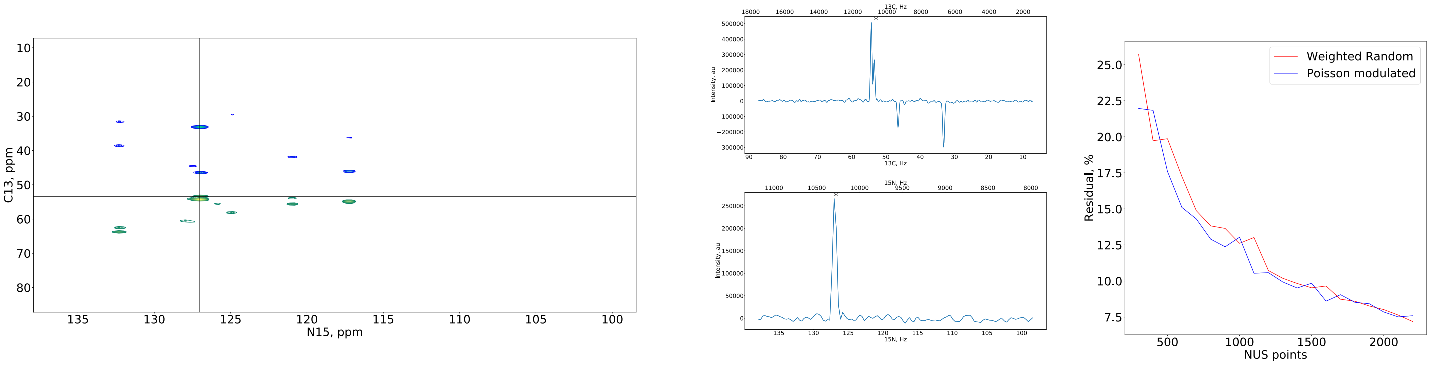

Peak25

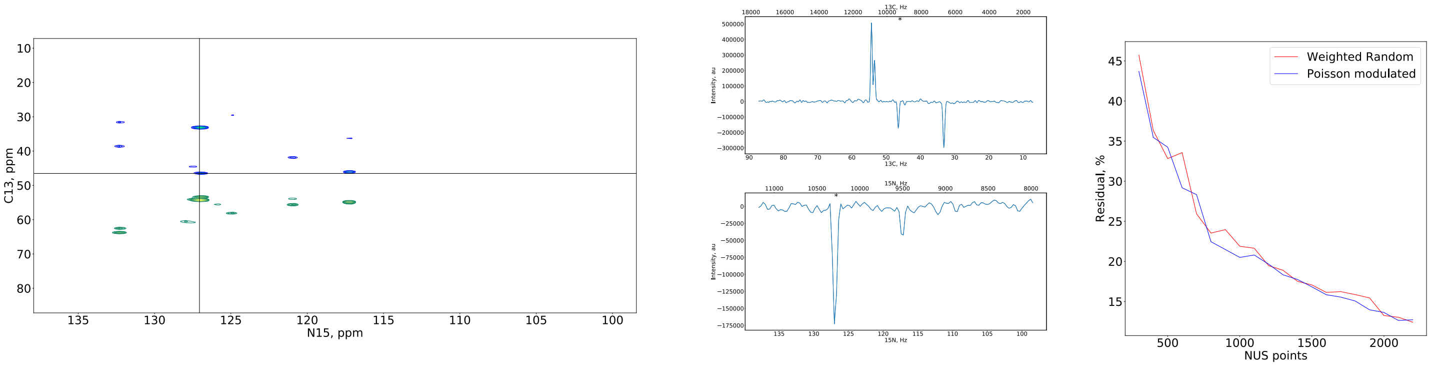

Peak26

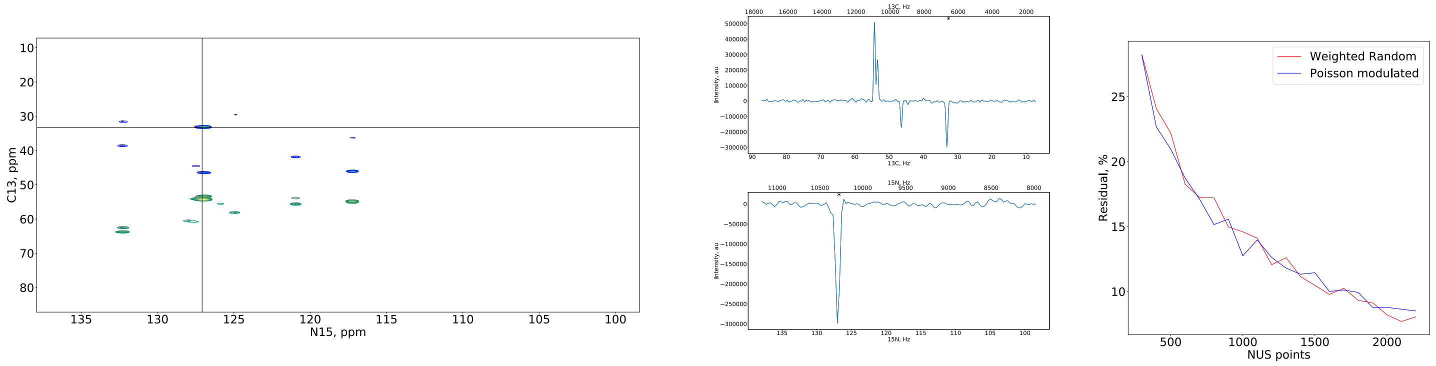

Peak27

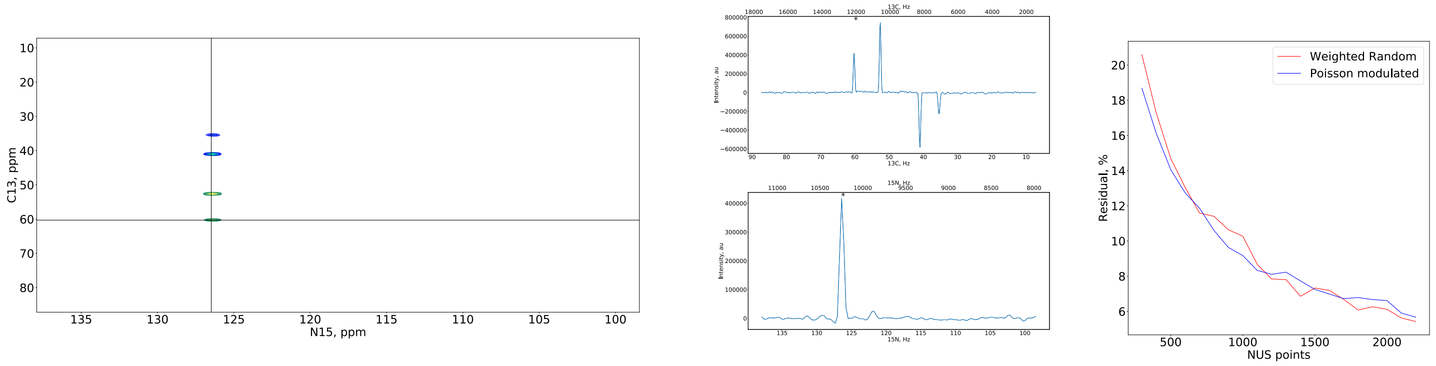

Peak28

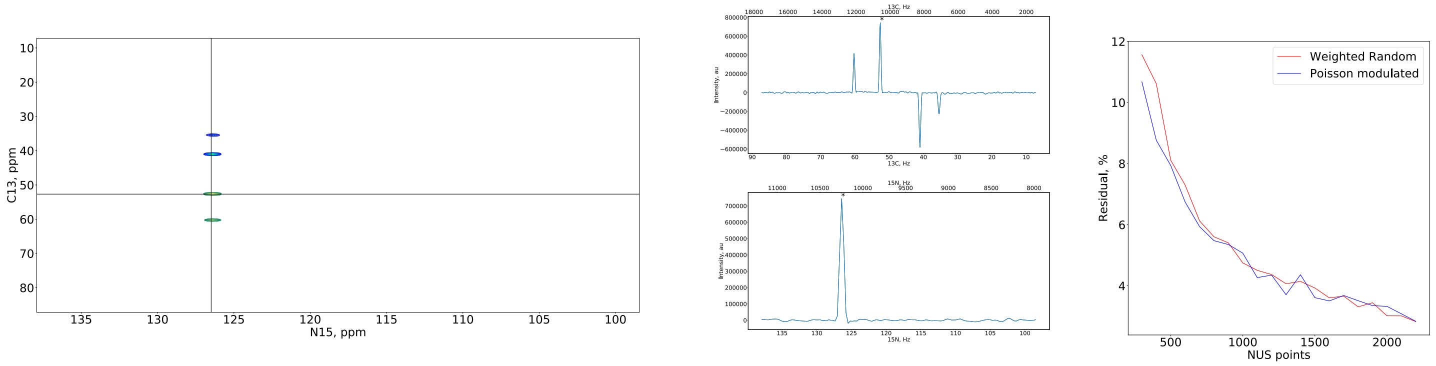

Peak29

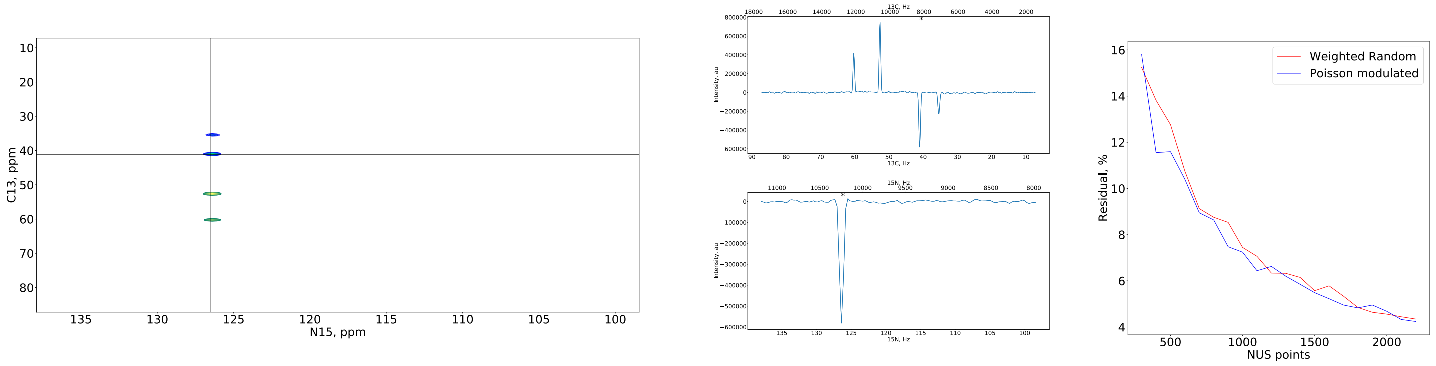

Peak30

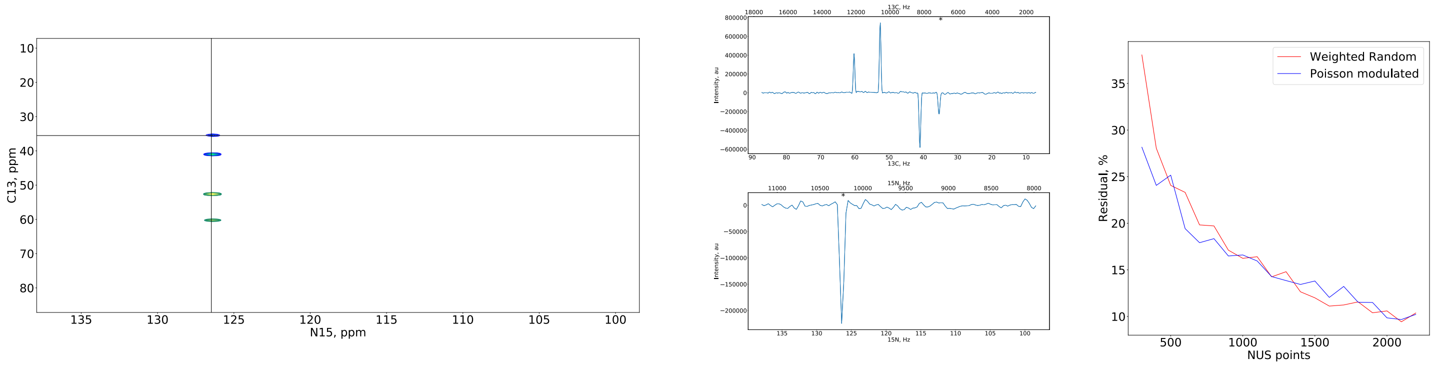

Peak31

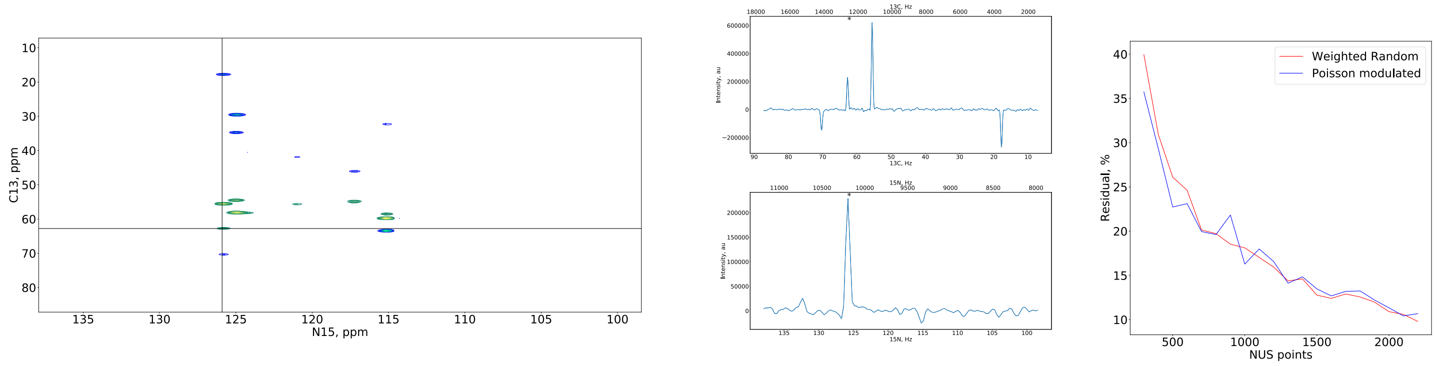

Peak32

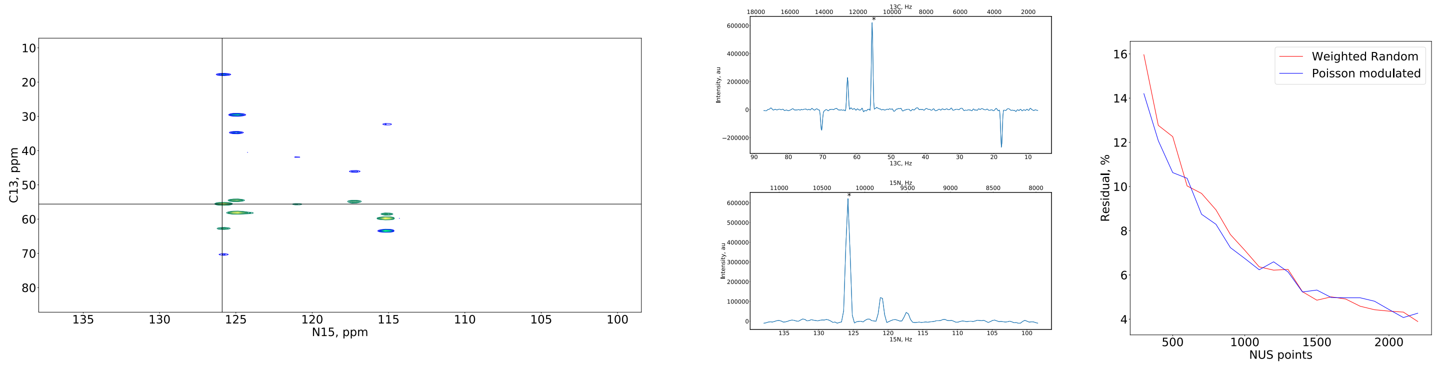

Peak33

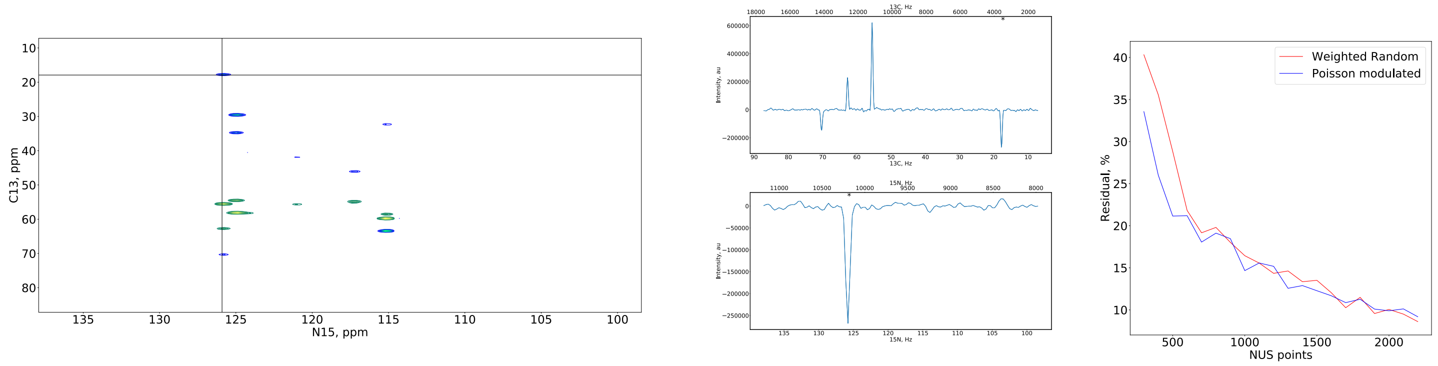

Peak34

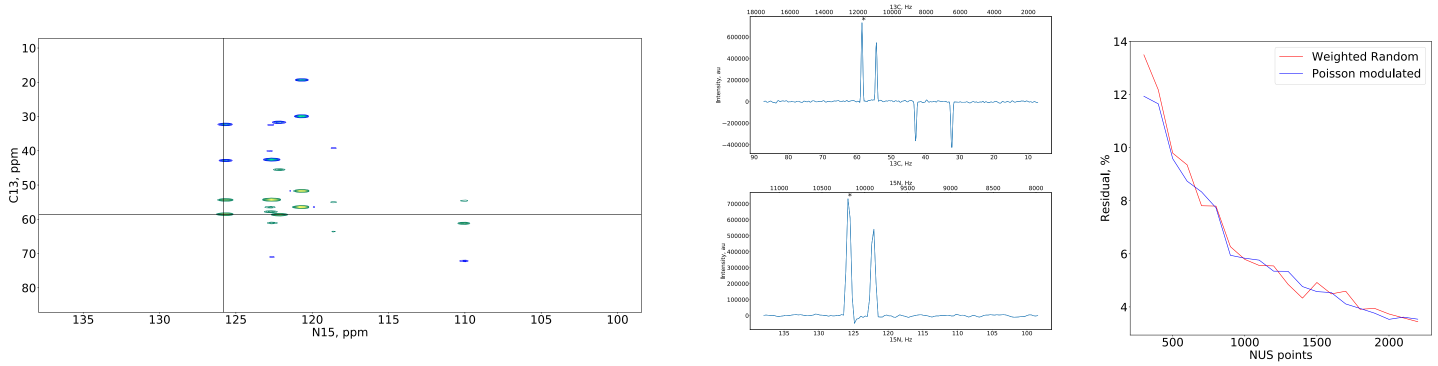

Peak35

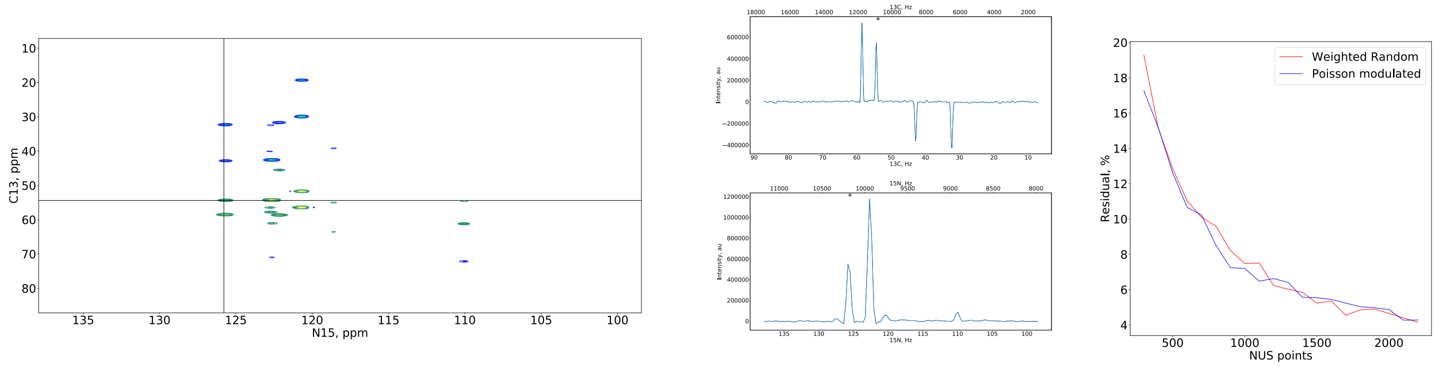

Peak36

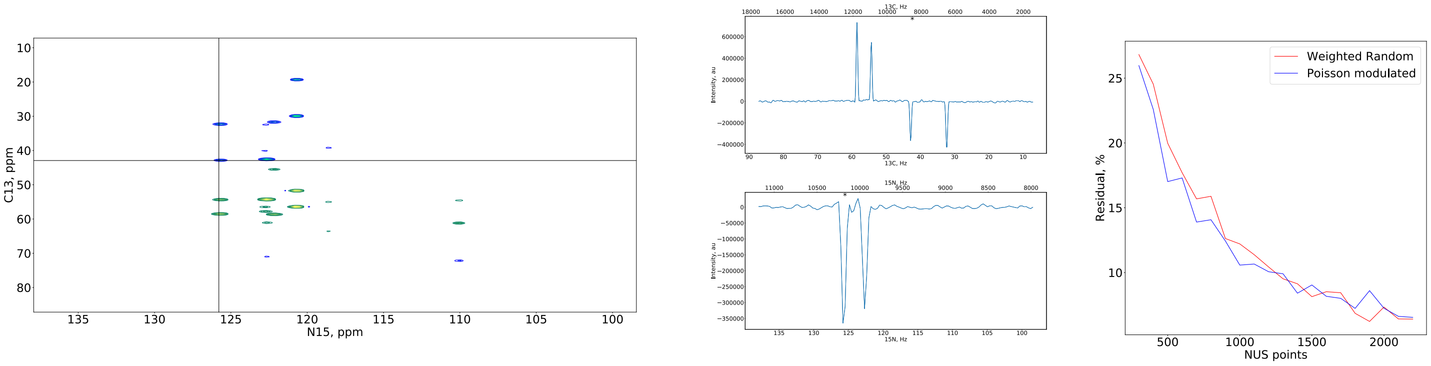

Peak37

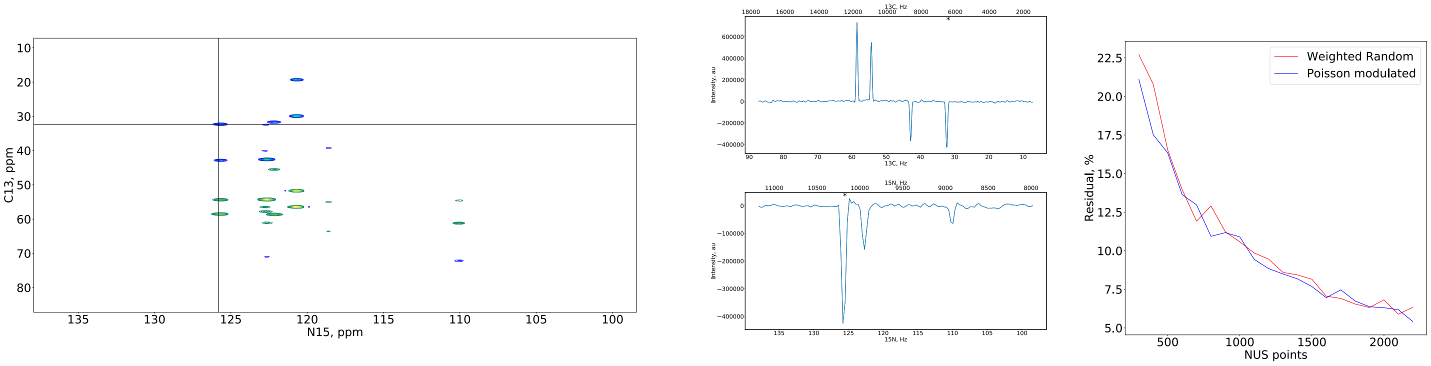

Peak38

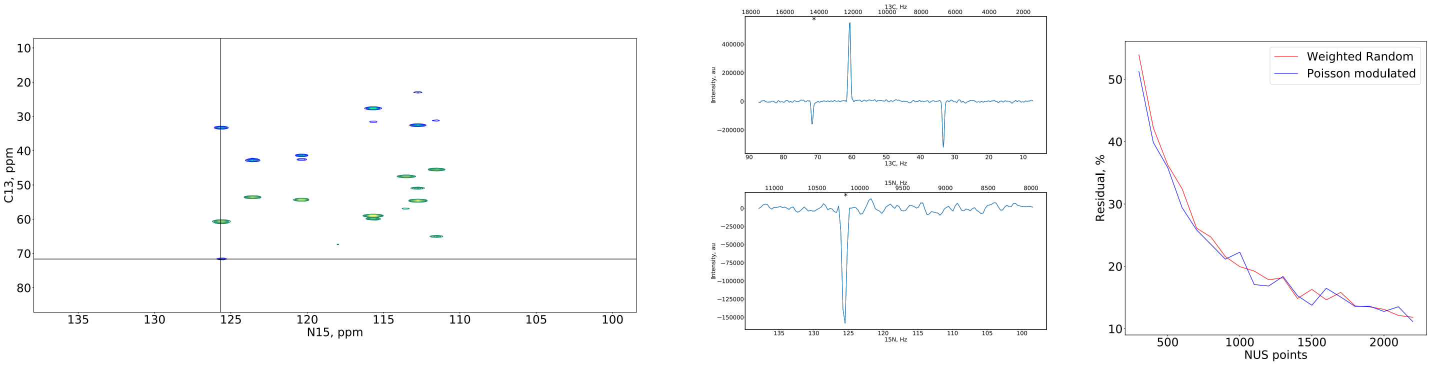

Peak39

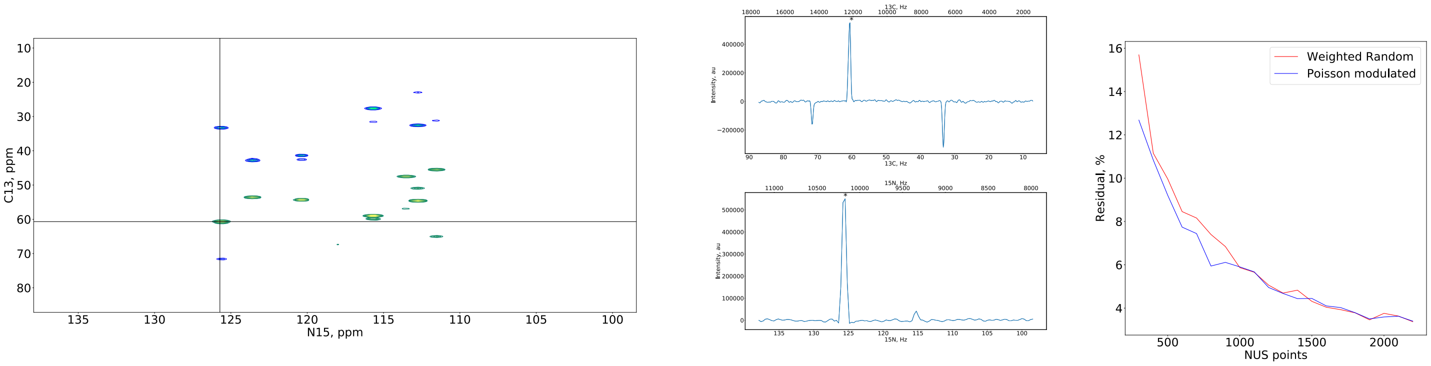

Peak40

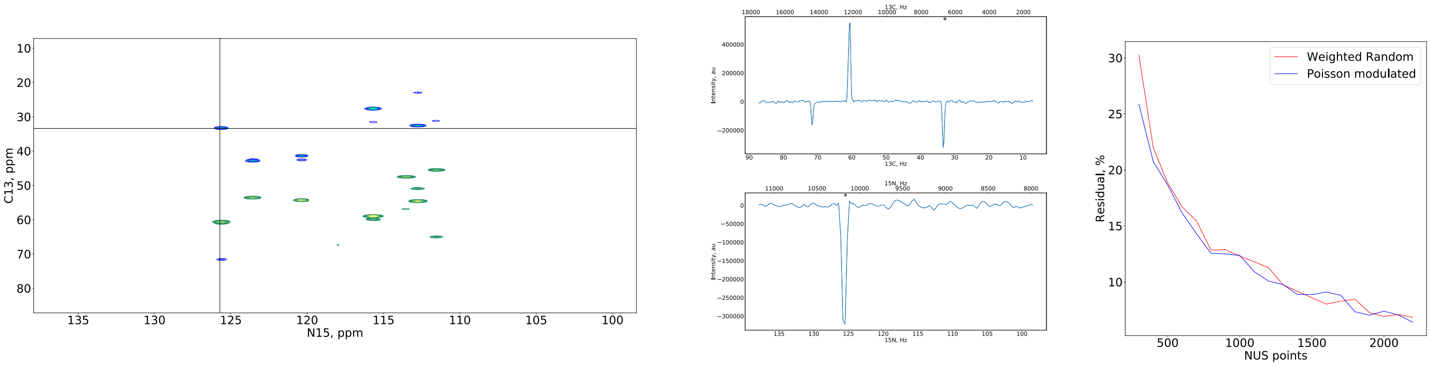

Peak41

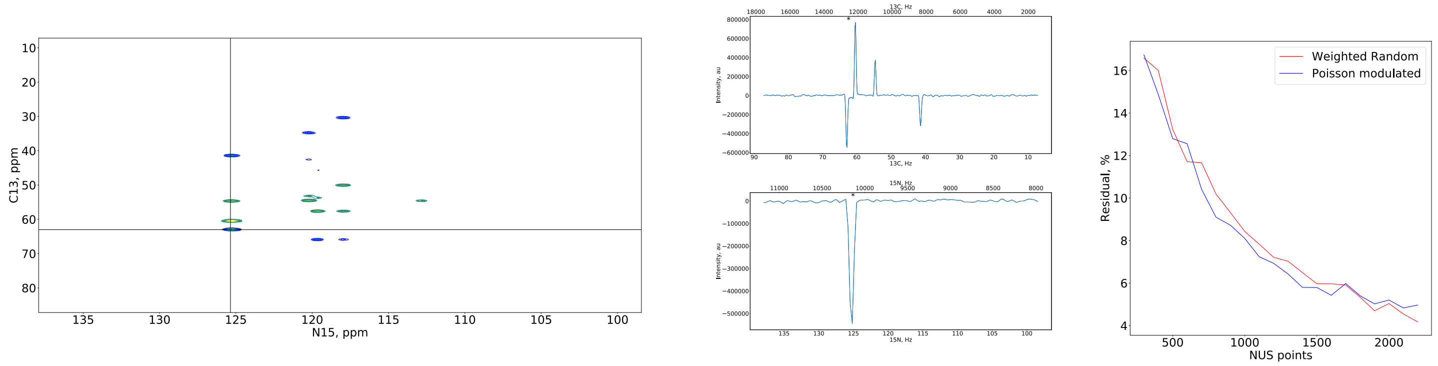

Peak42

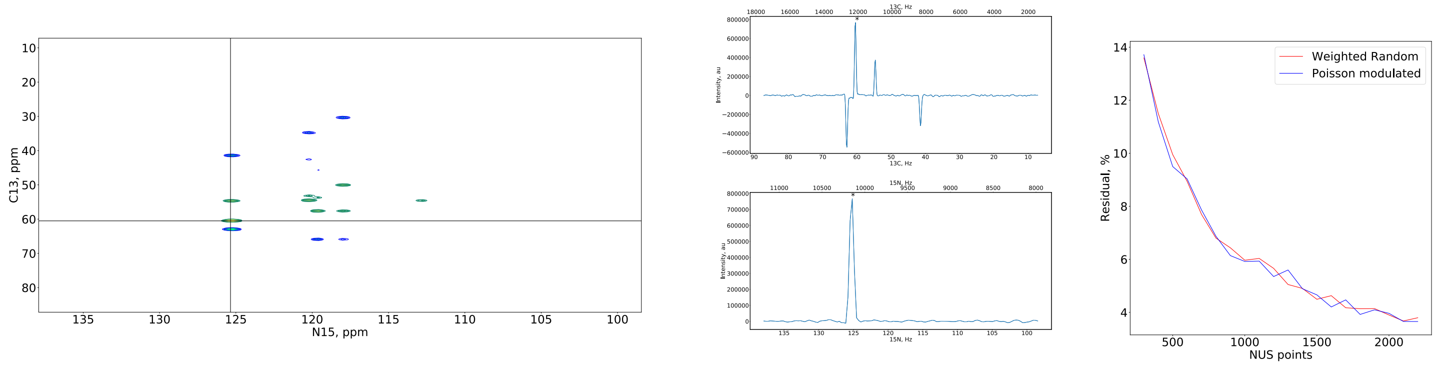

Peak43

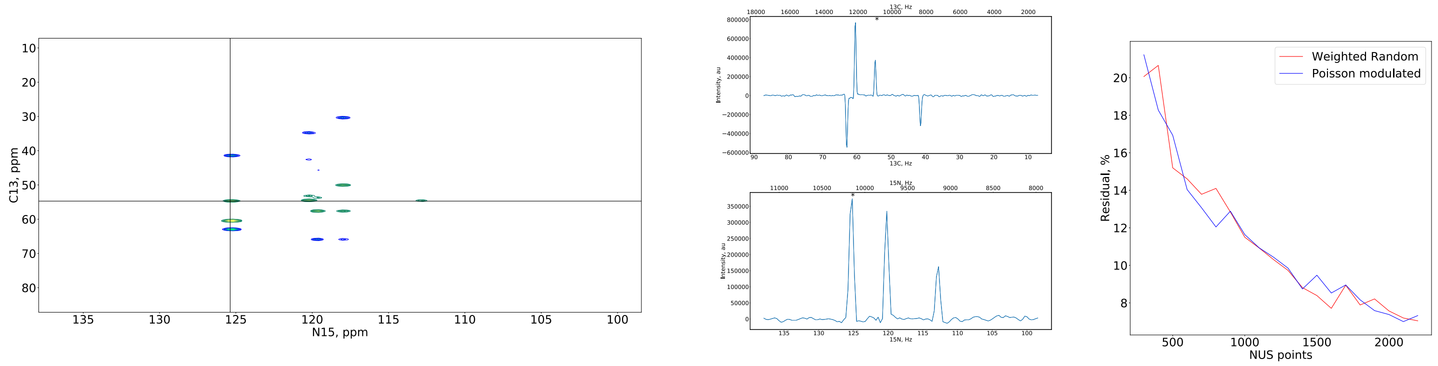

Peak44

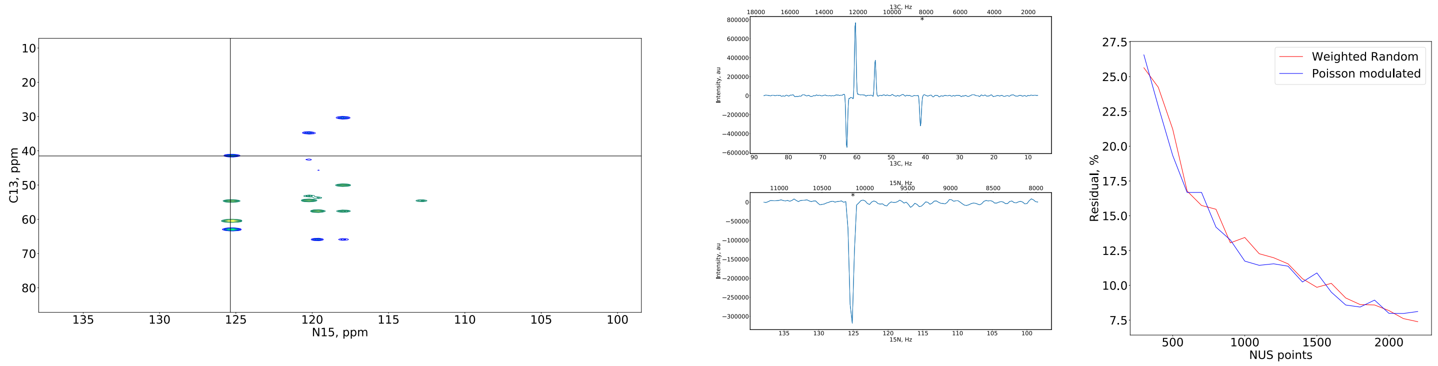

Peak45

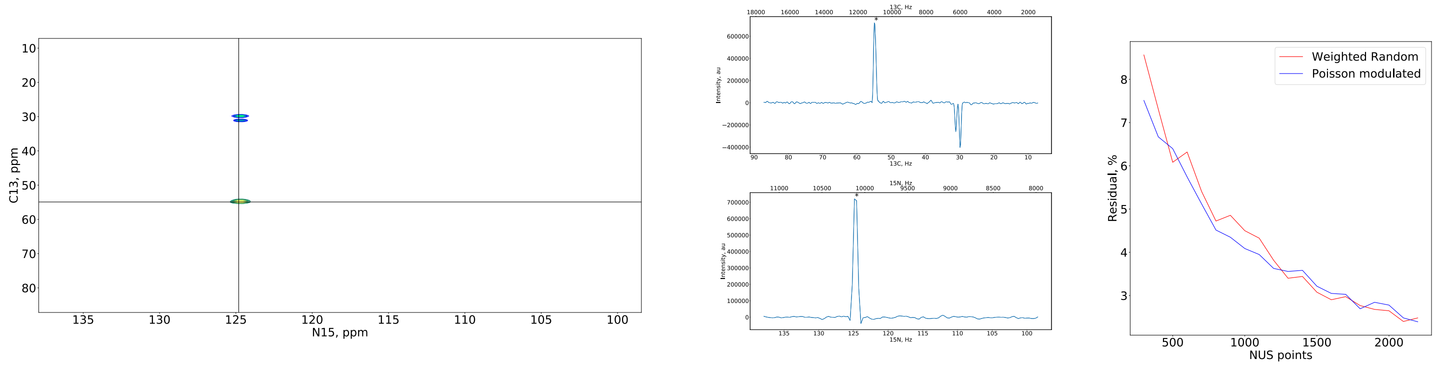

Peak46

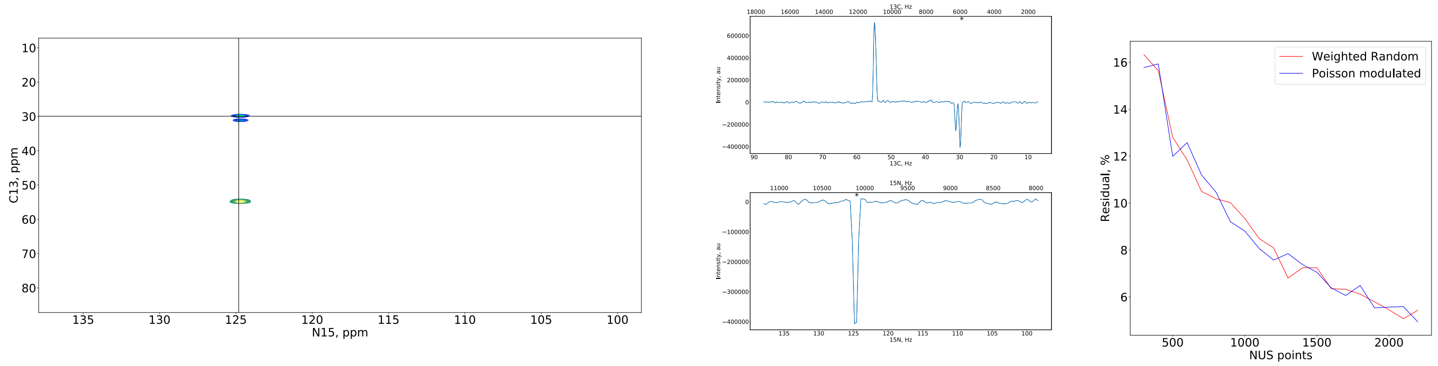

Peak47

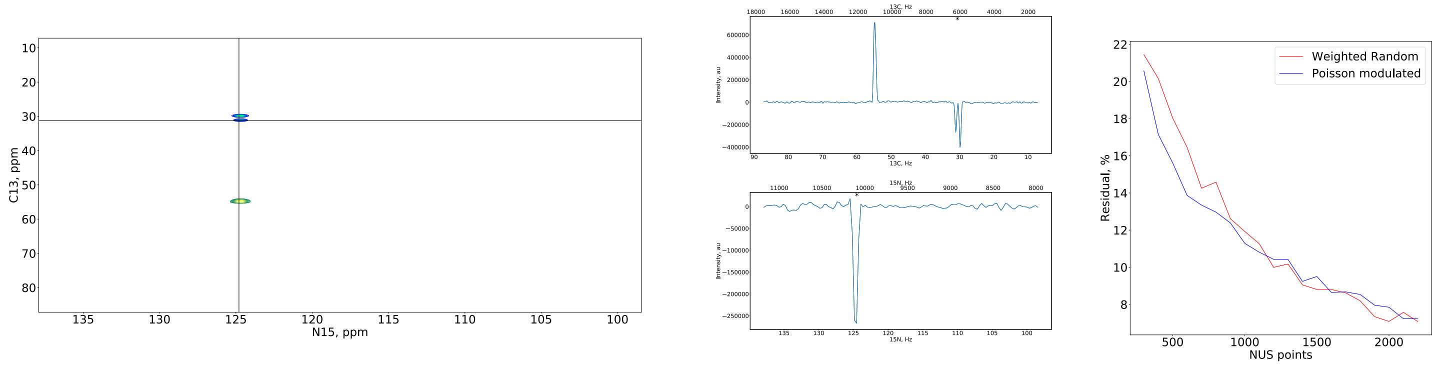

Peak48

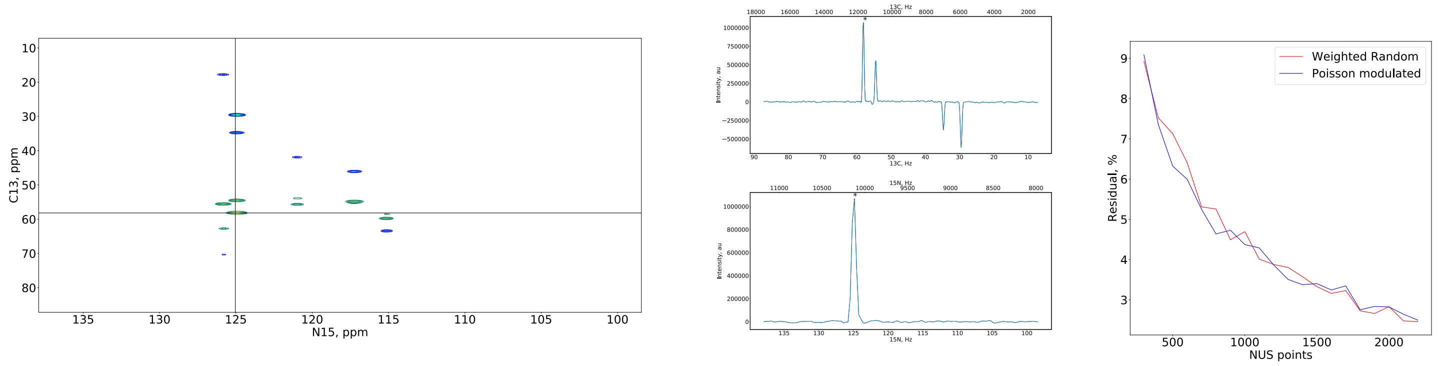

Peak49

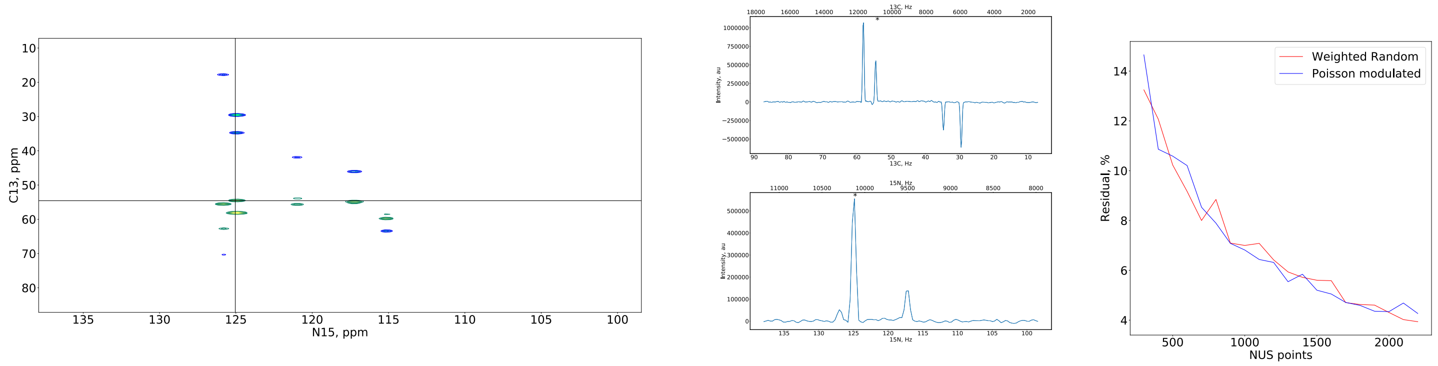

Peak50

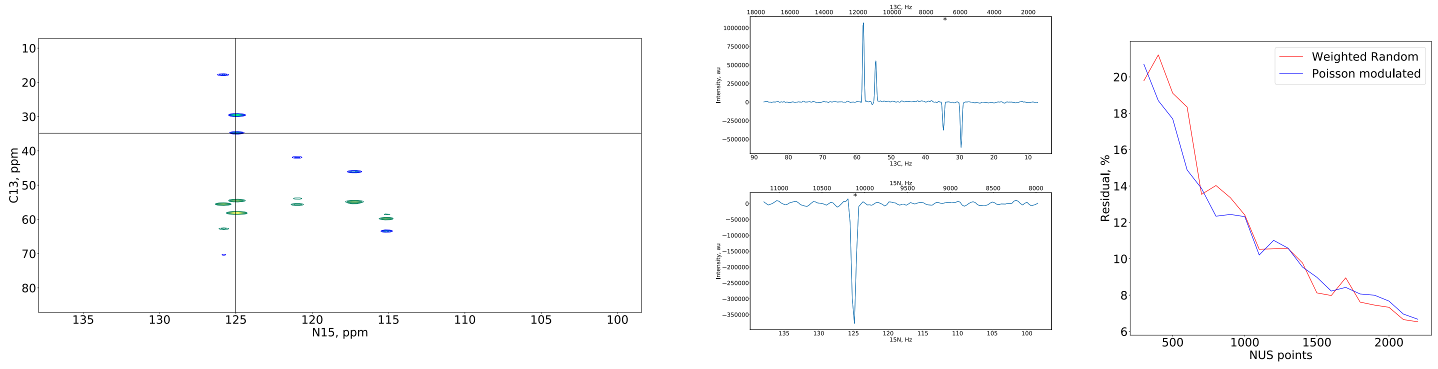

Peak51

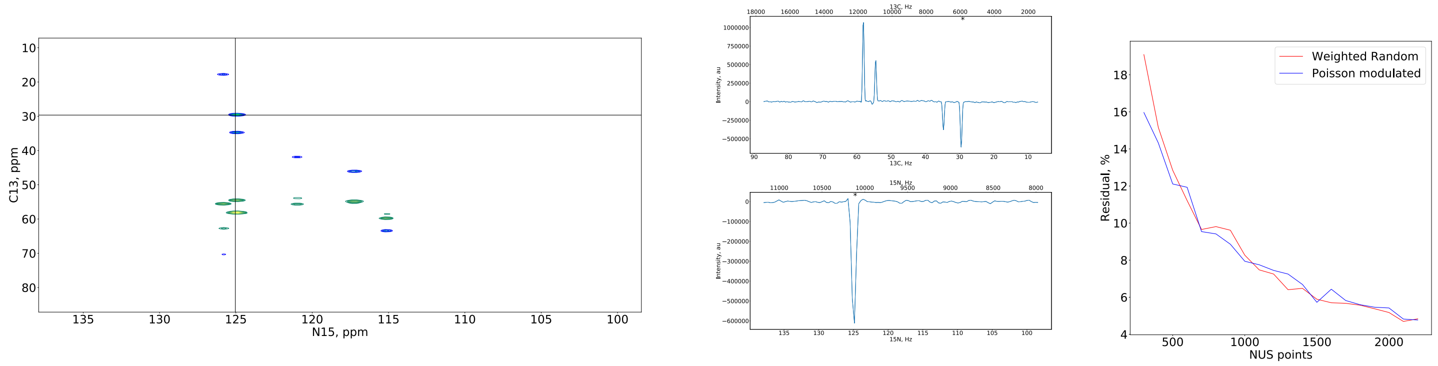

Peak52

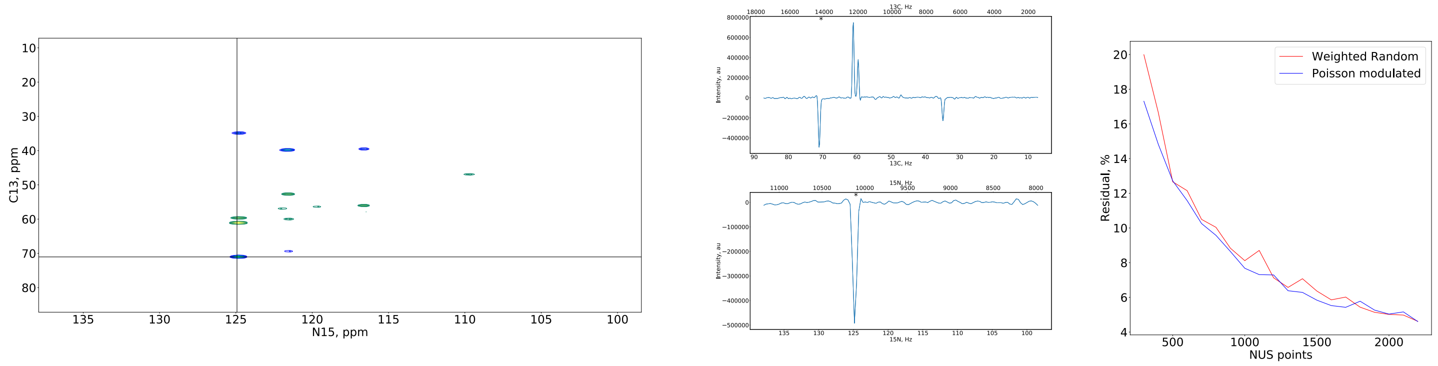

Peak53

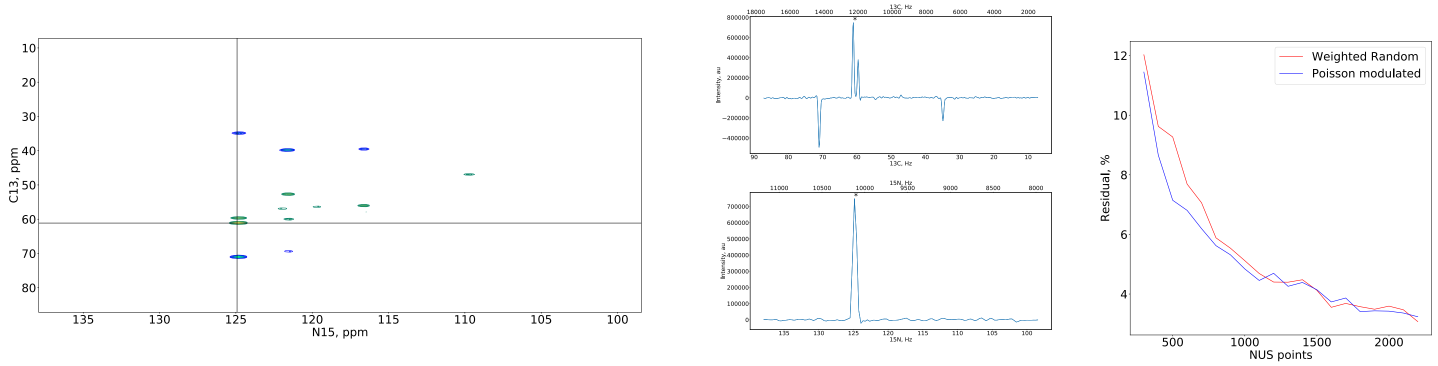

Peak54

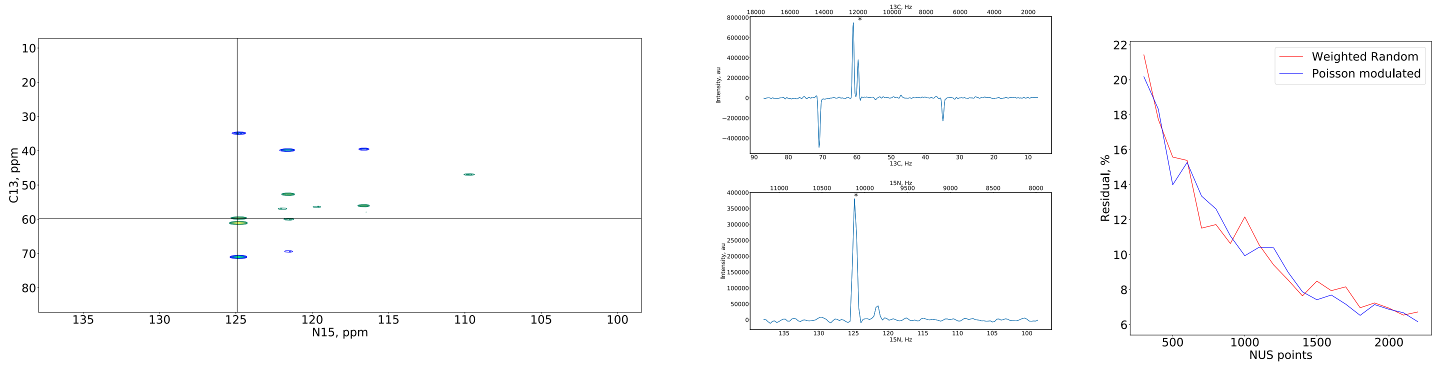

Peak55

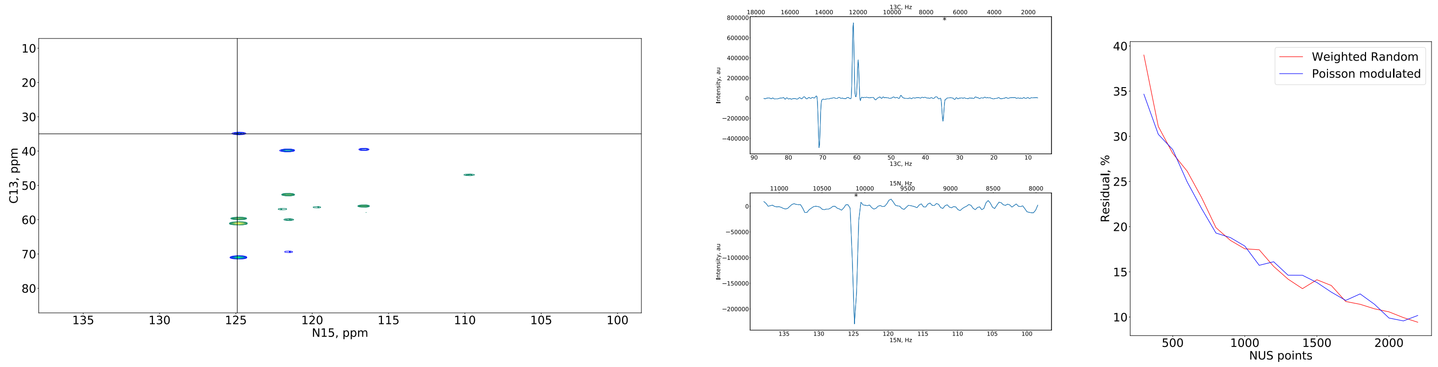

Peak56

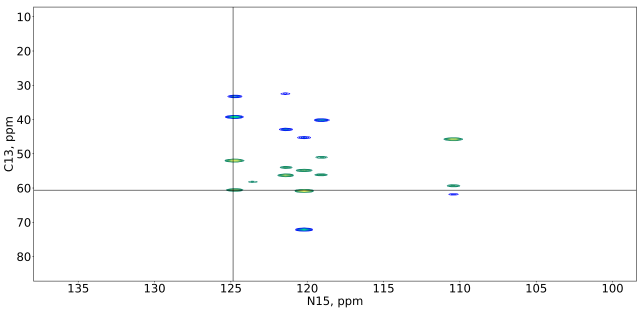

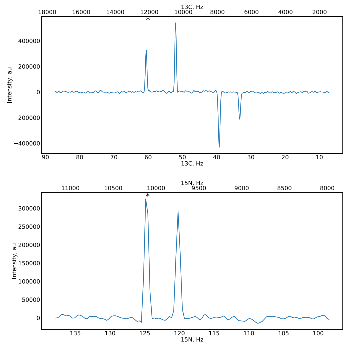

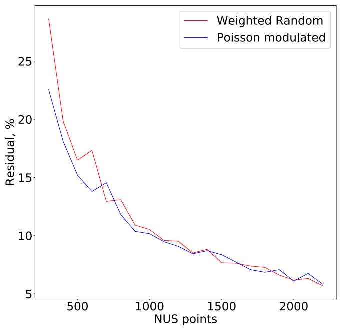

Peak57

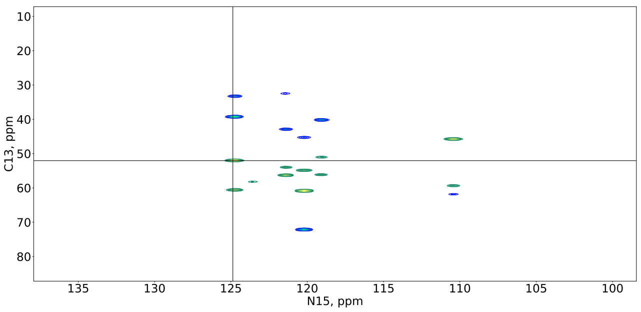

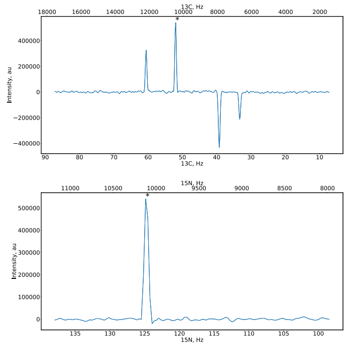

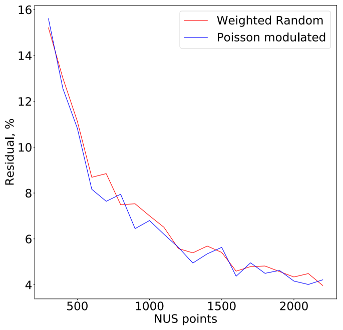

Peak58

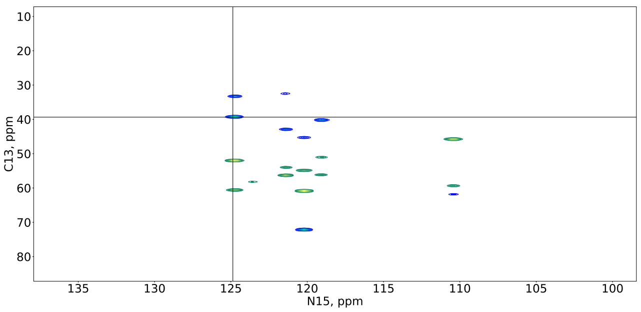

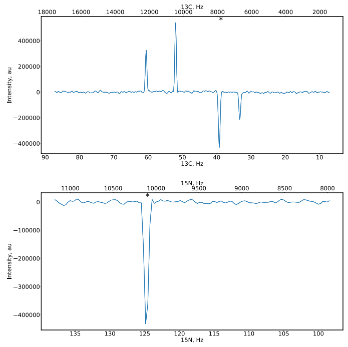

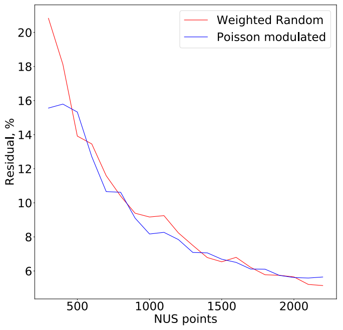

Peak59

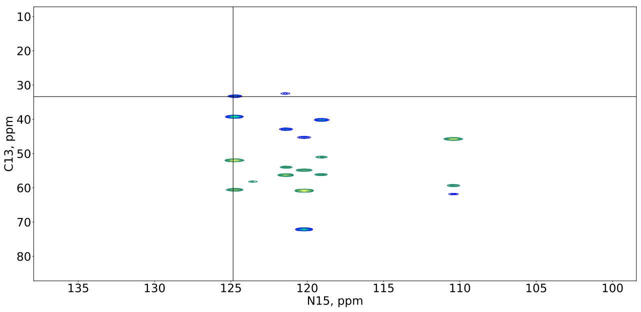

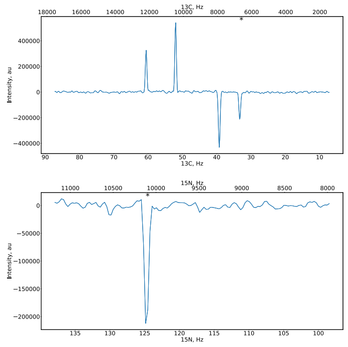

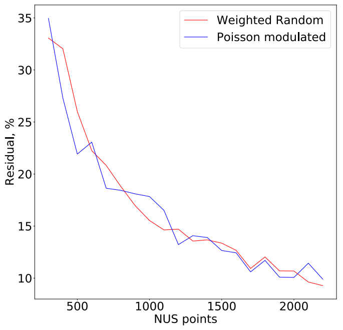

Peak60

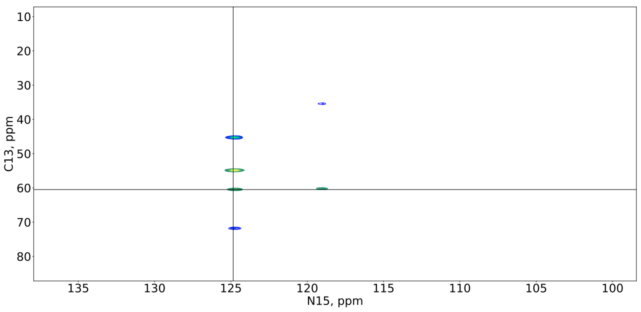

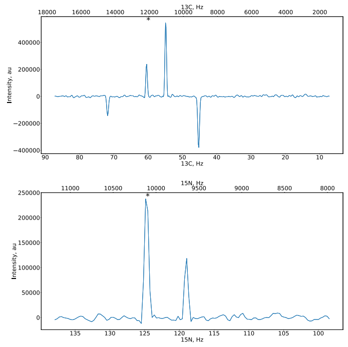

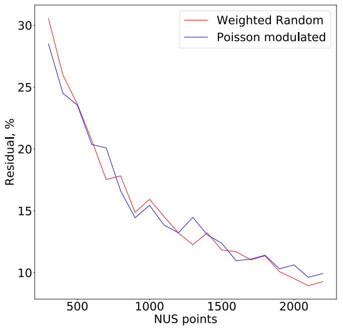

Peak61

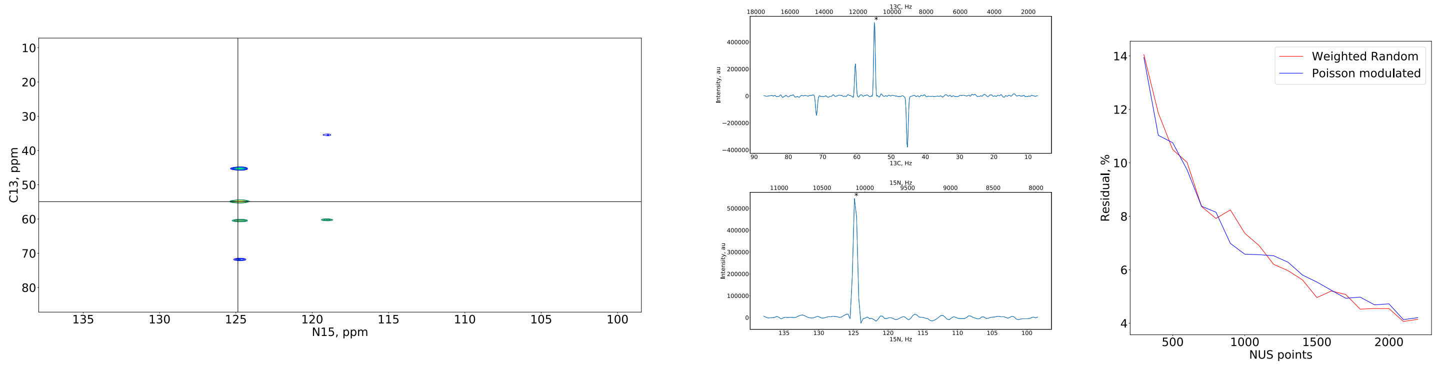

Peak62

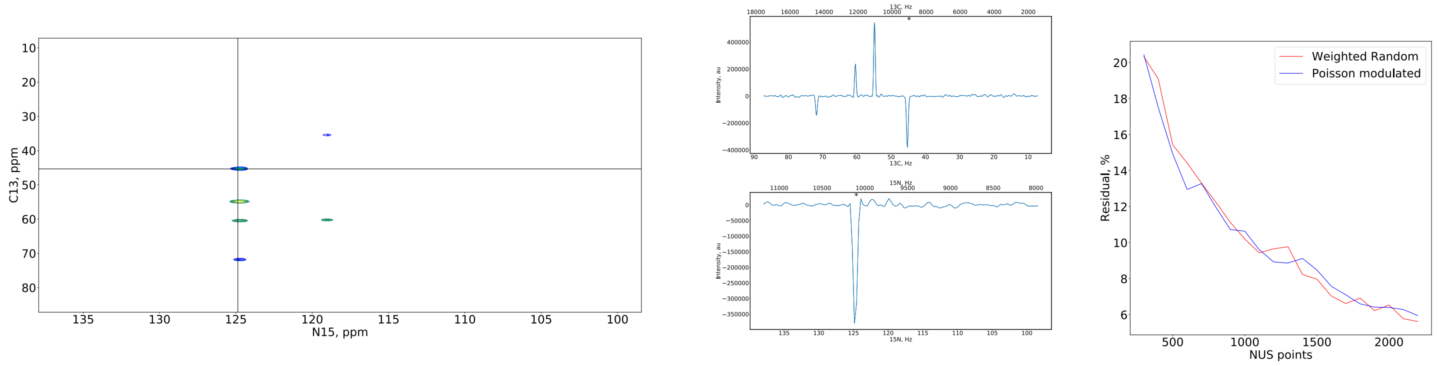

Peak63

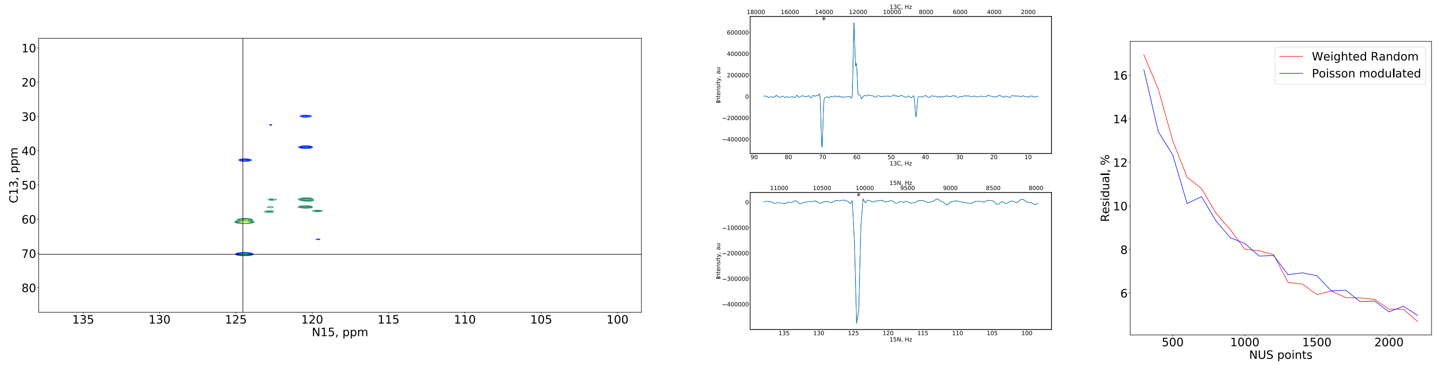

Peak64

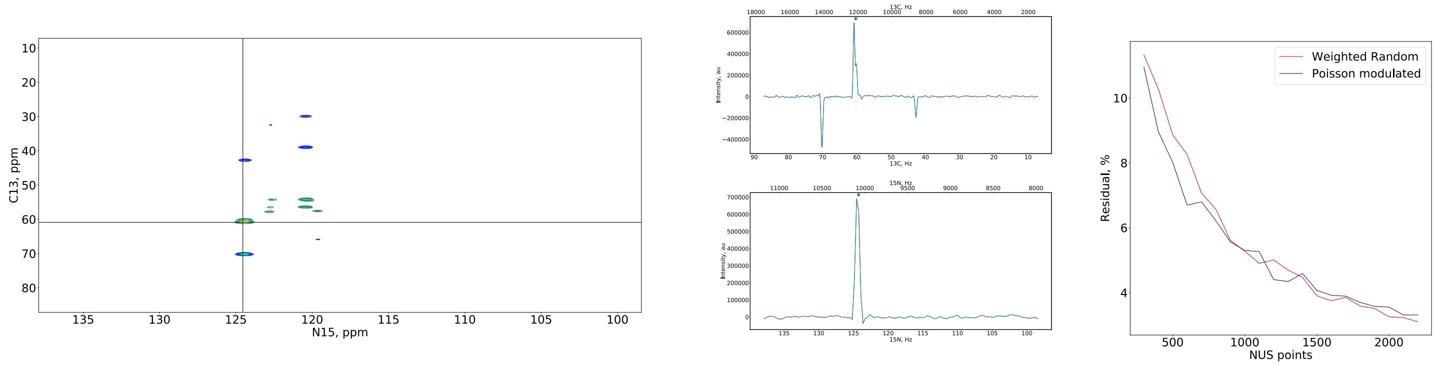

Peak65

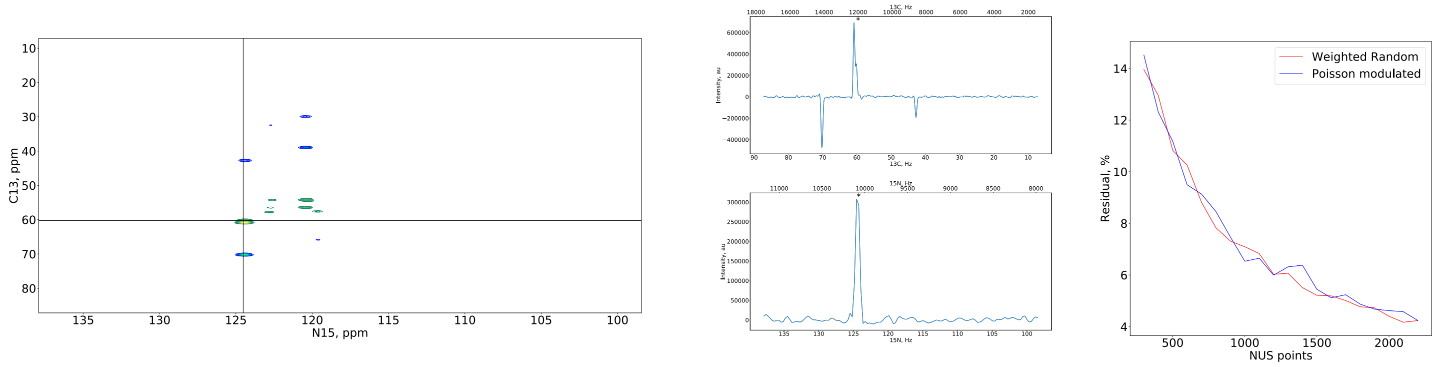

Peak66

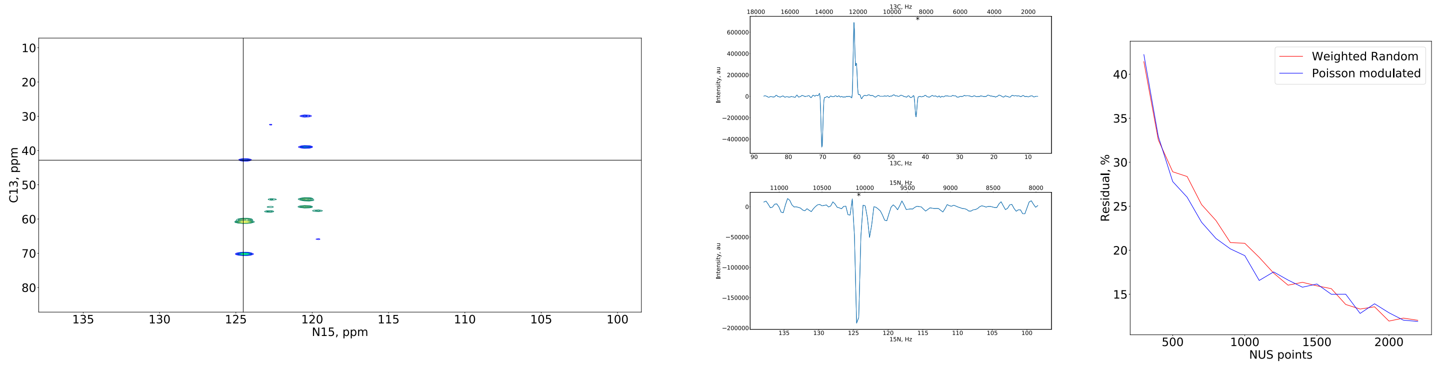

Peak67

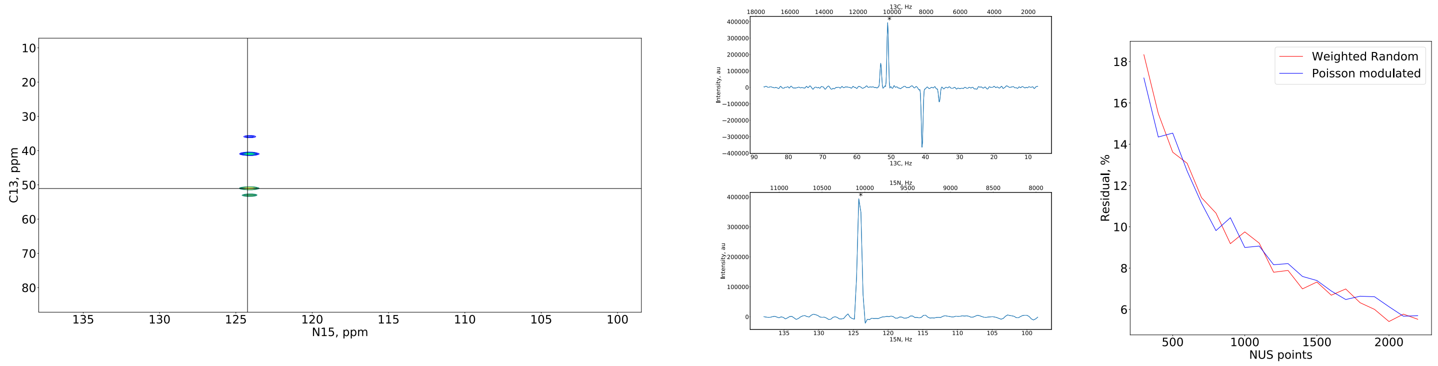

Peak68

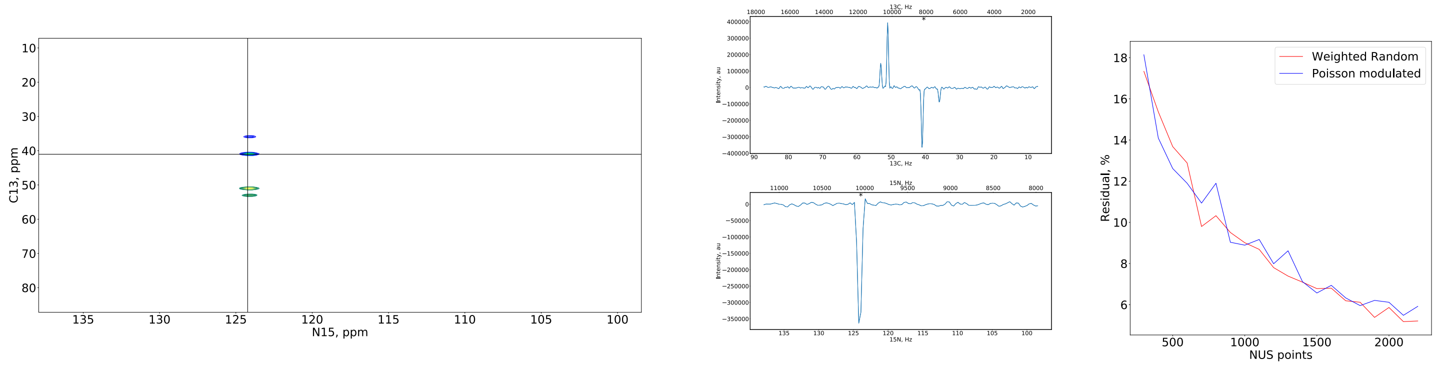

Peak69

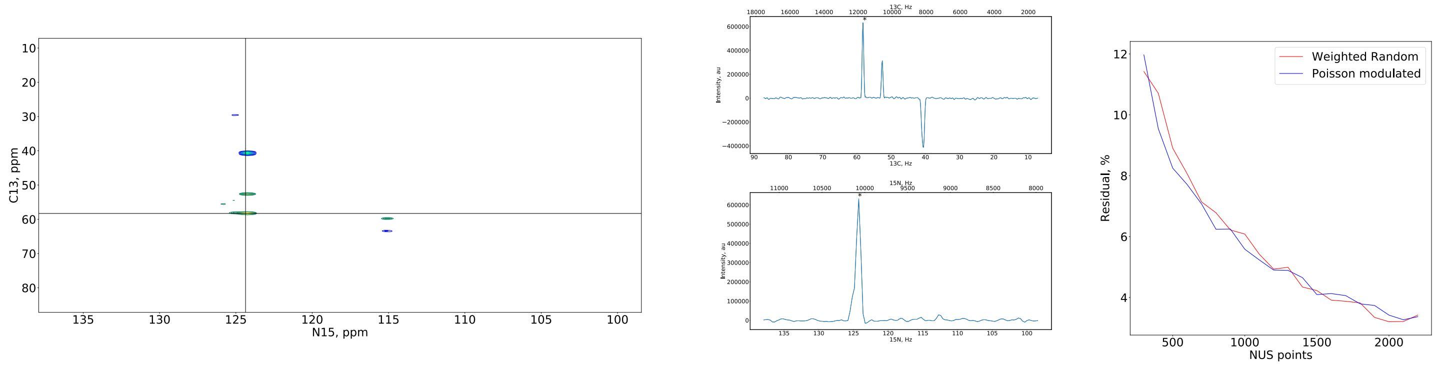

Peak70

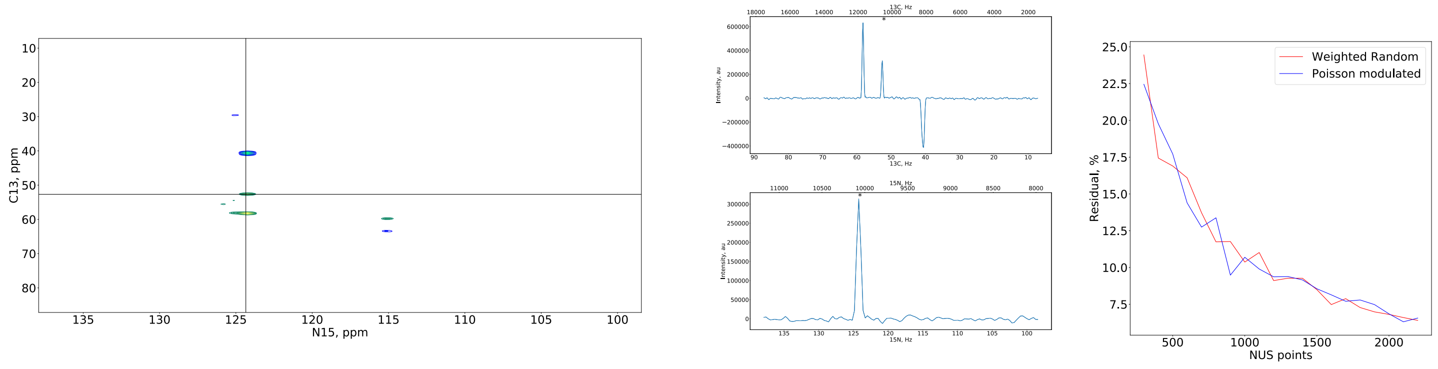

Peak71

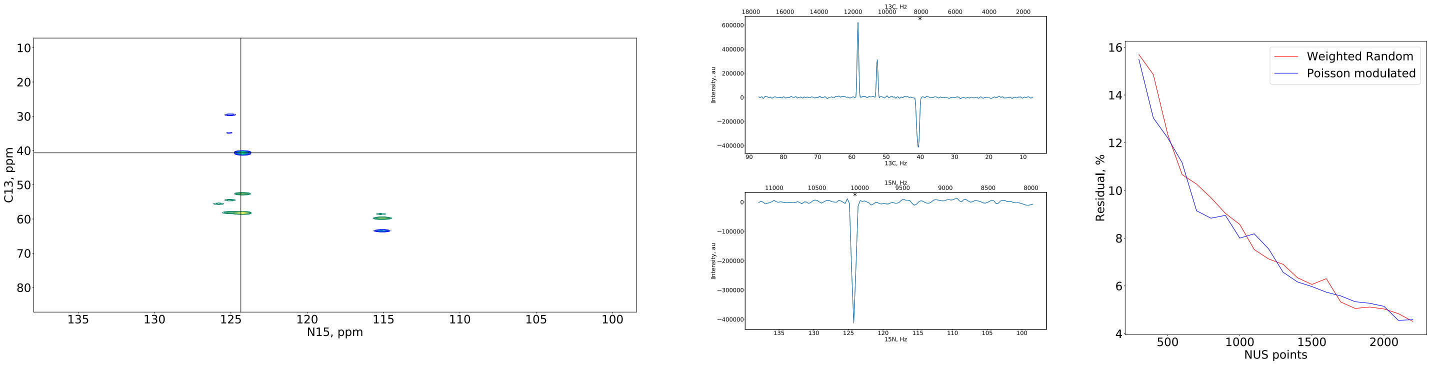

Peak72

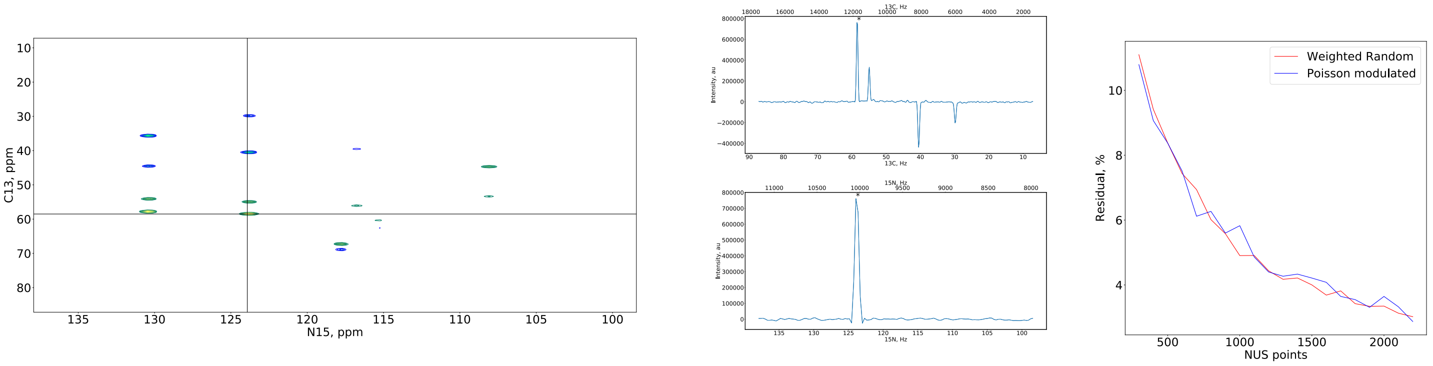

Peak73

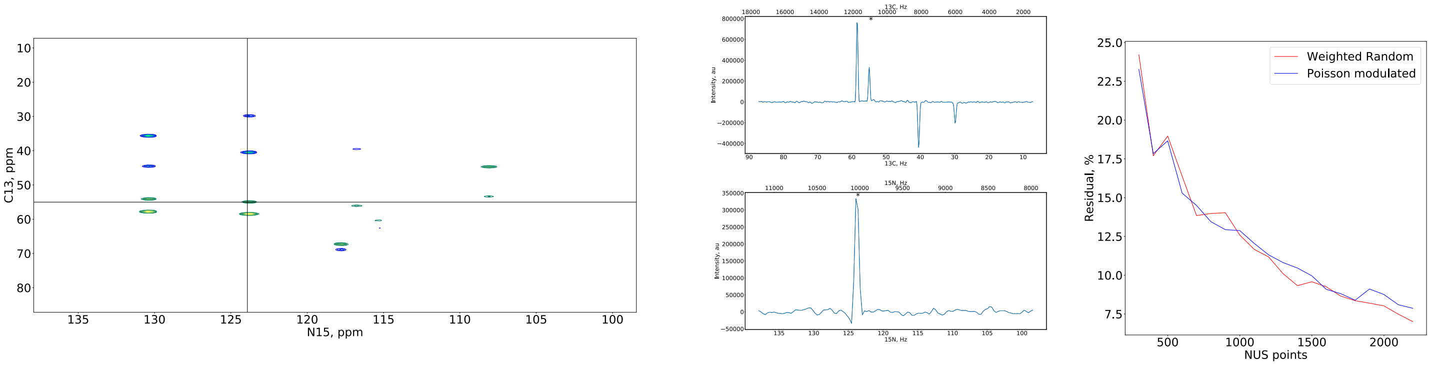

Peak74

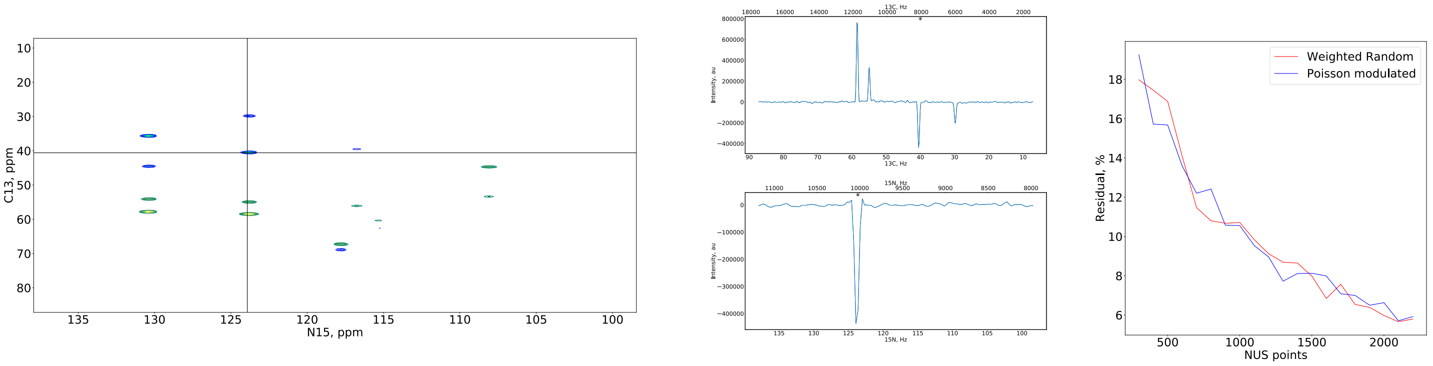

Peak75

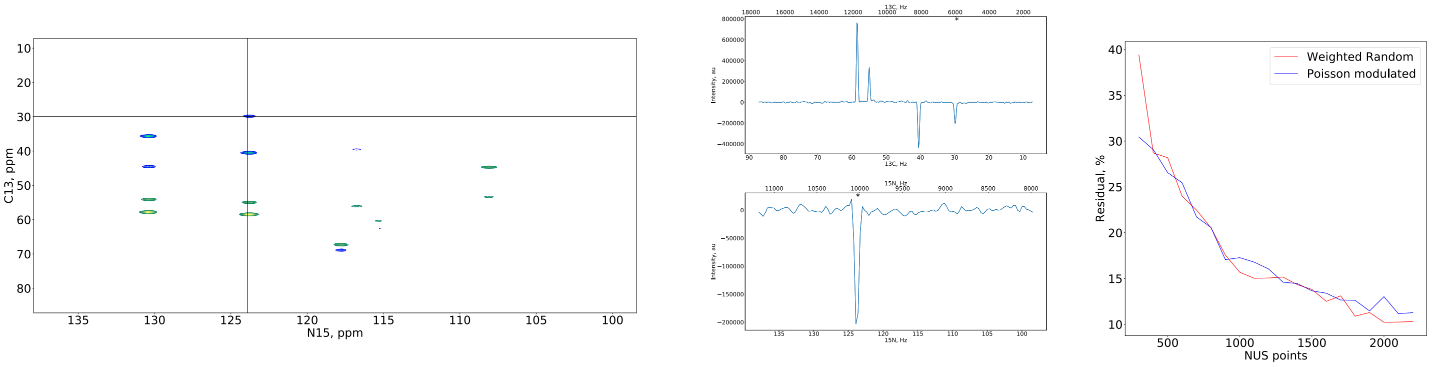

Peak76

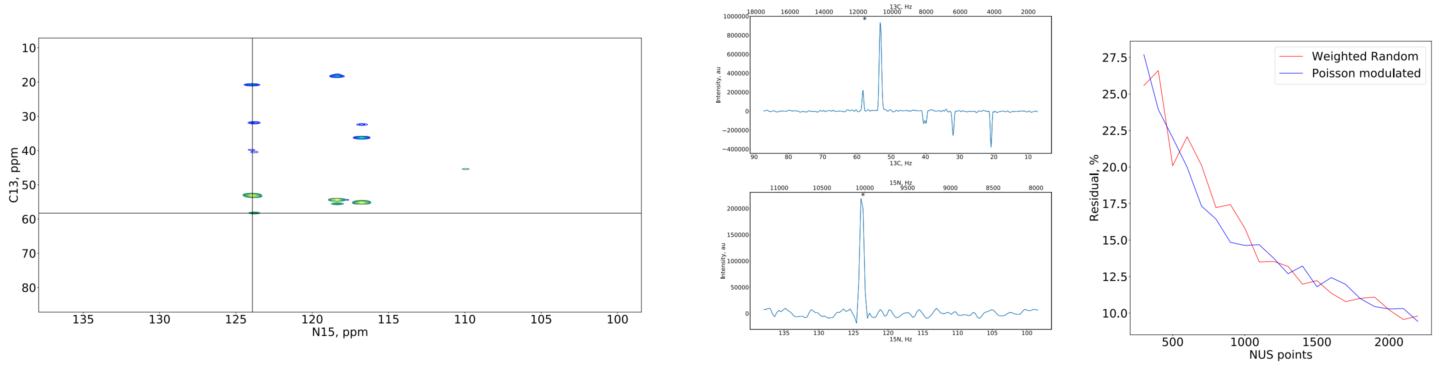

Peak77

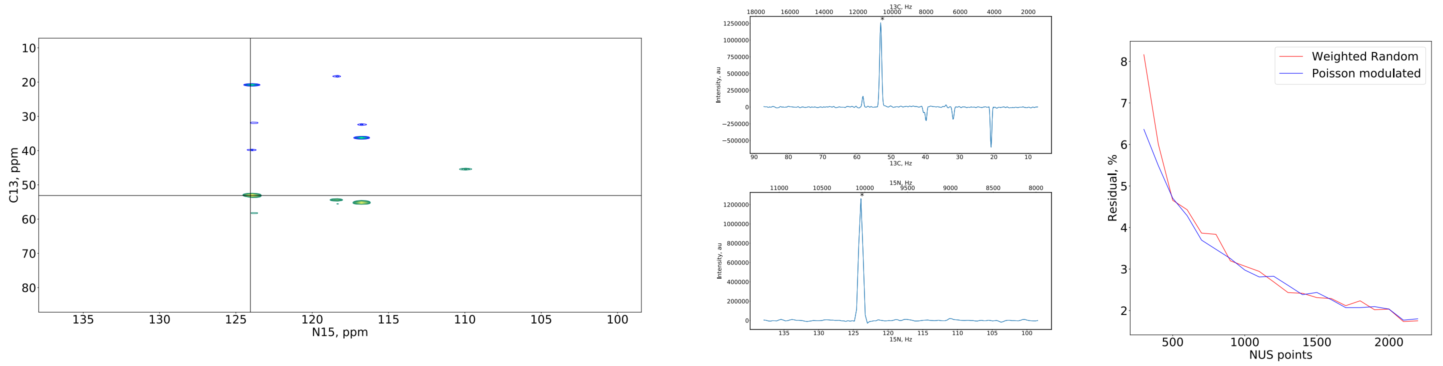

Peak78

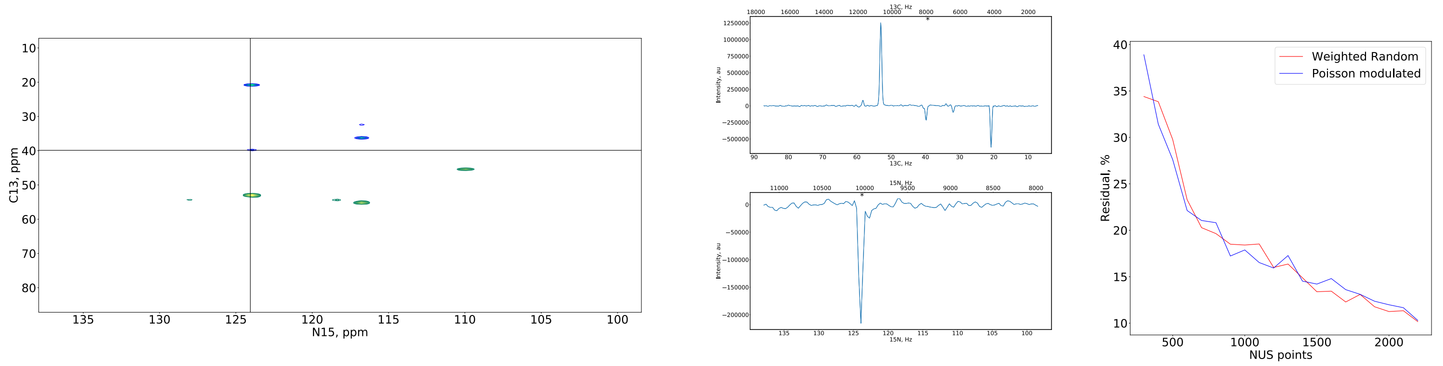

Peak79

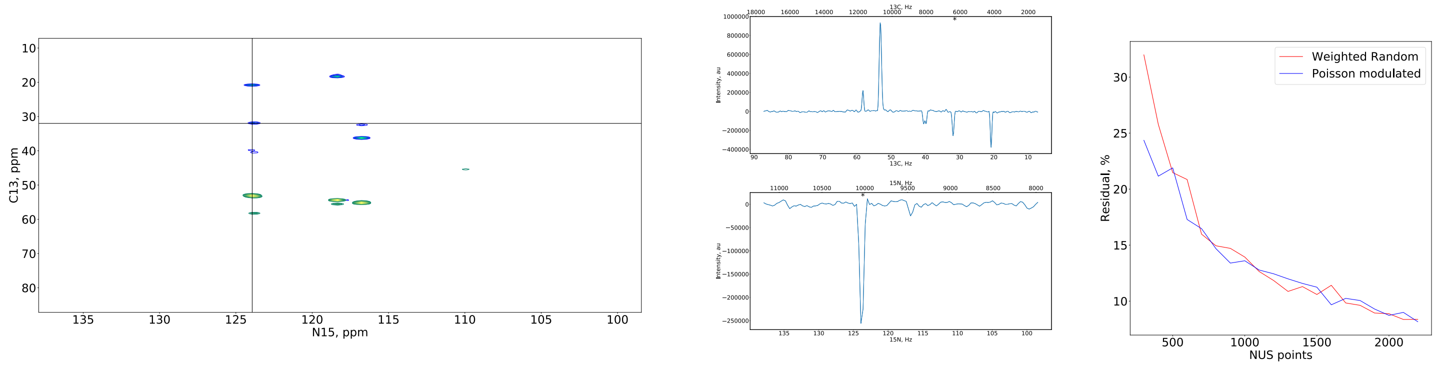

Peak80

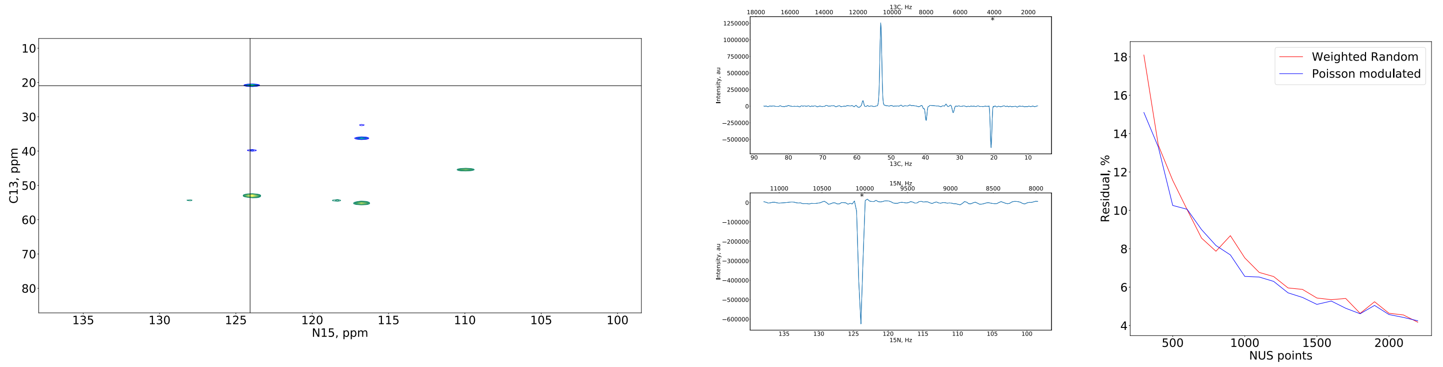

Peak81

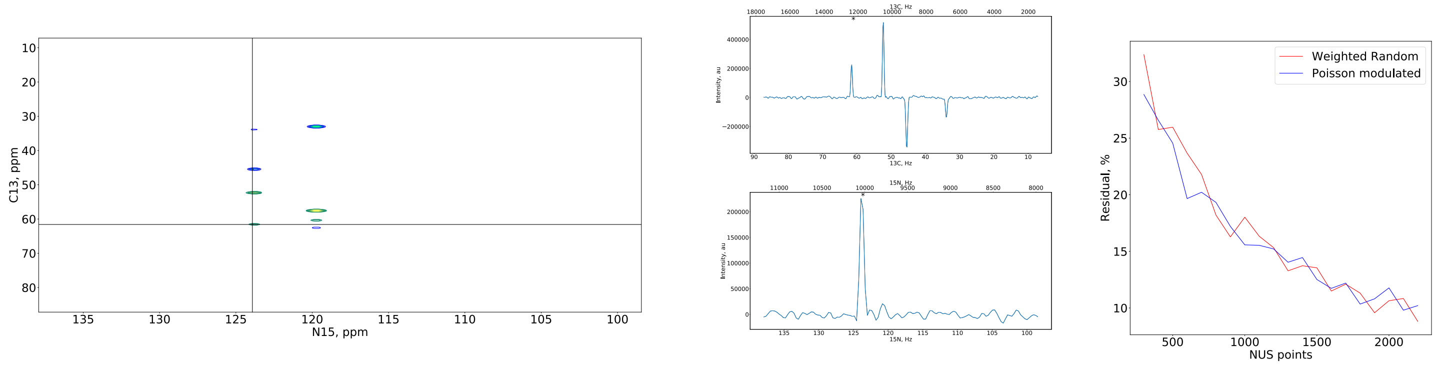

Peak82

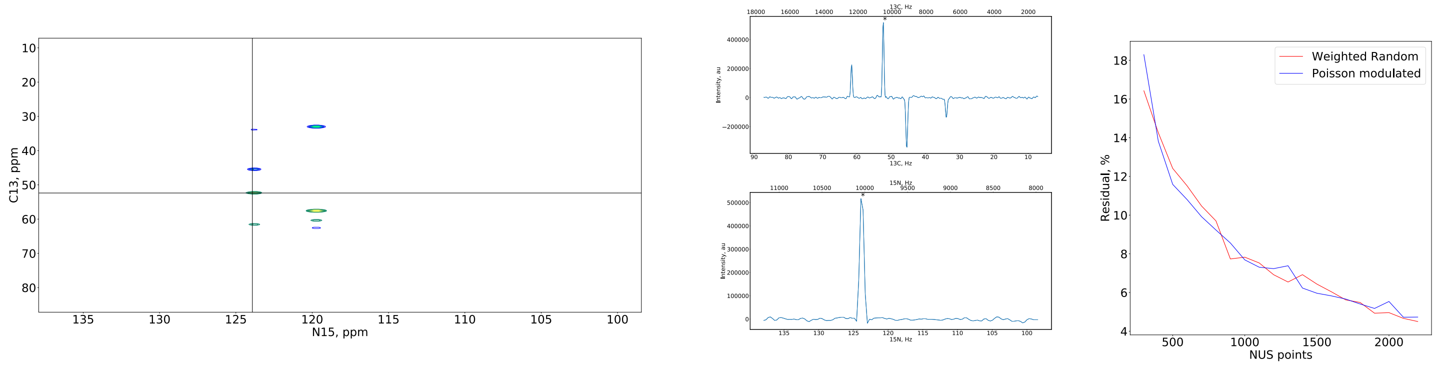

Peak83

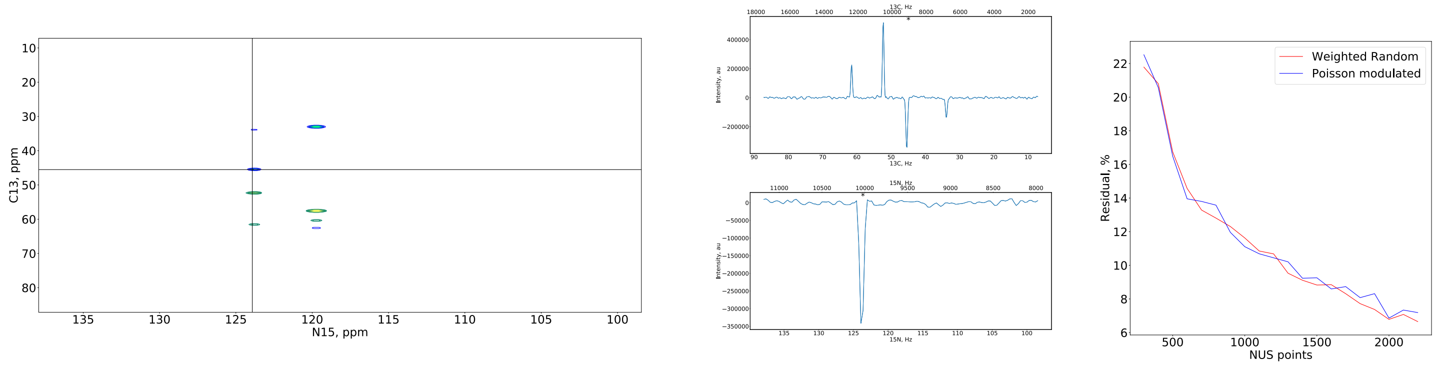

Peak84

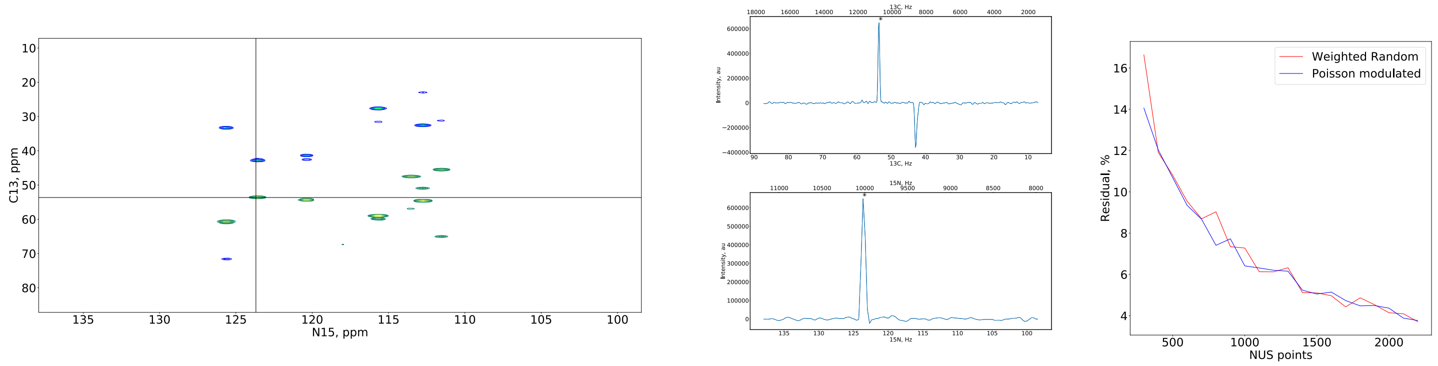

Peak85

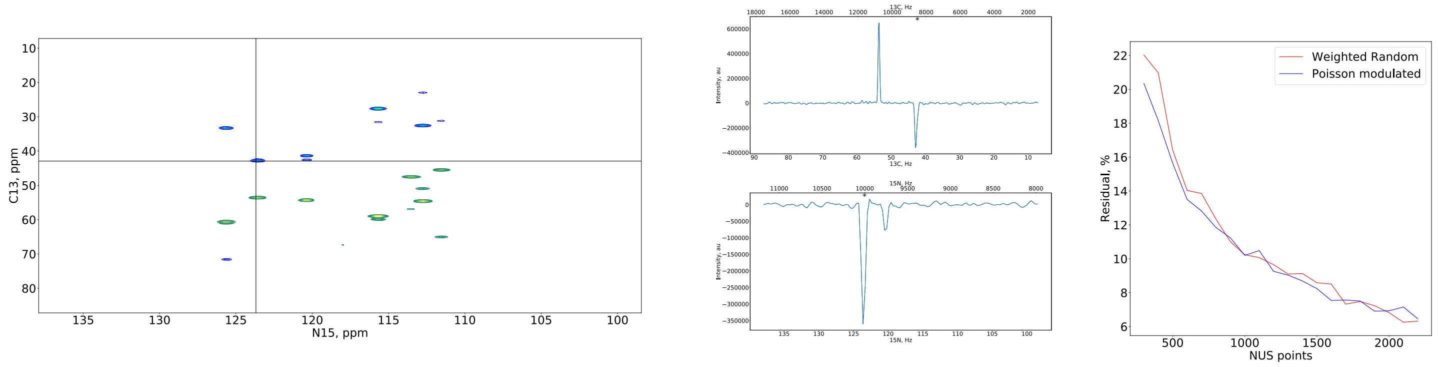

Peak86

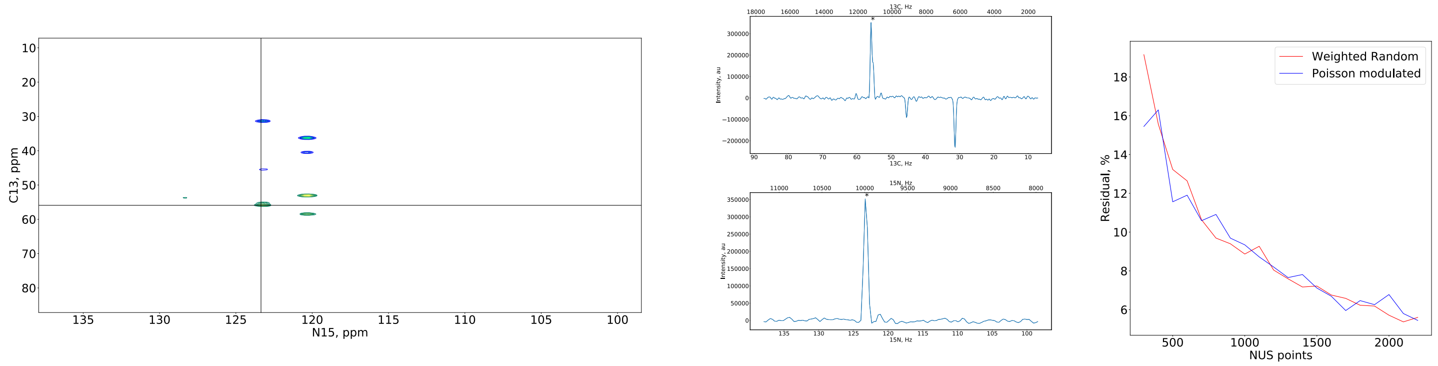

Peak87

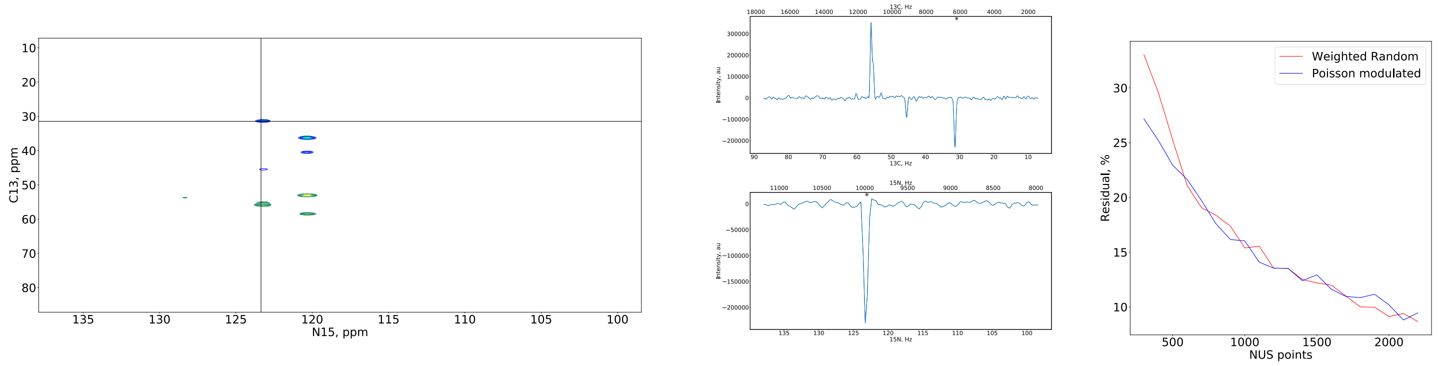

Peak88

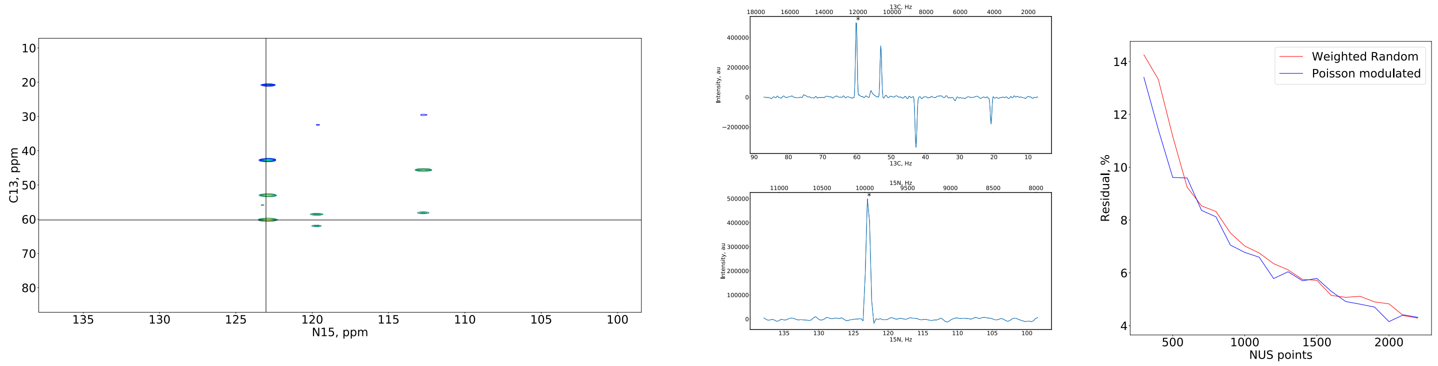

Peak89

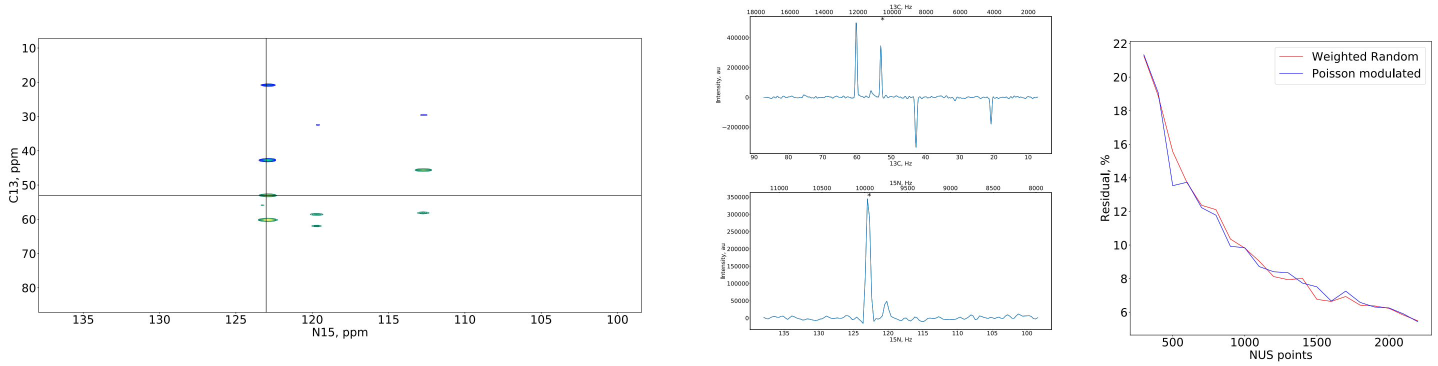

Peak90

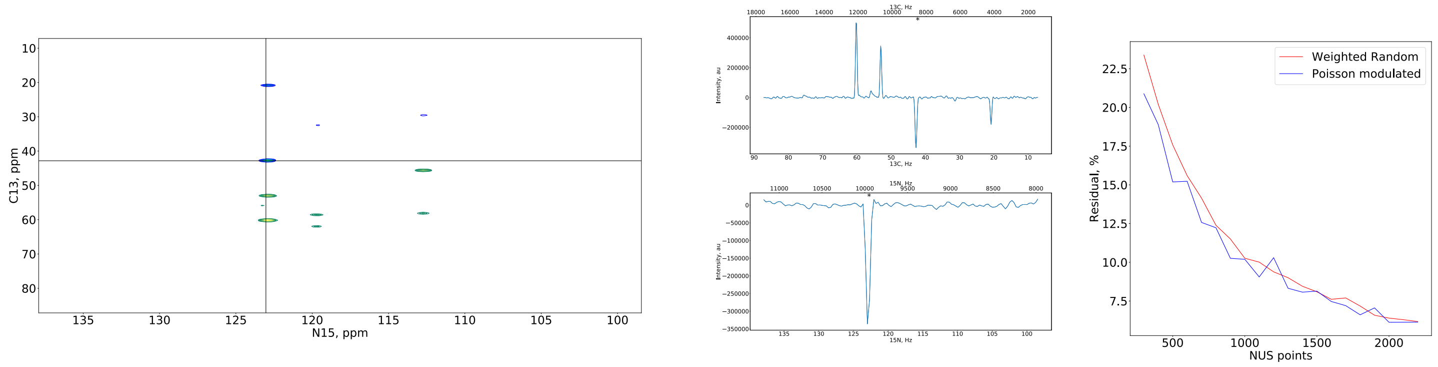

Peak91

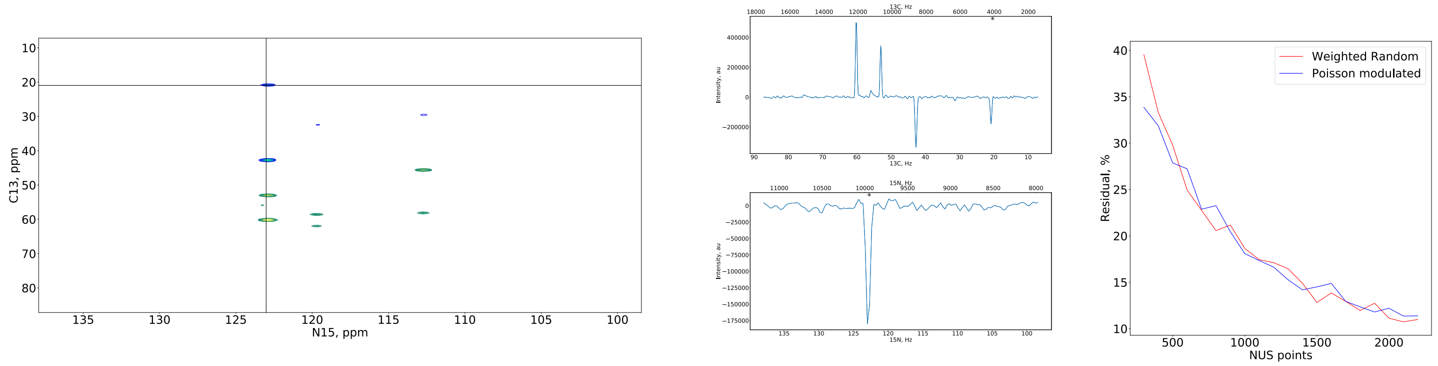

Peak92

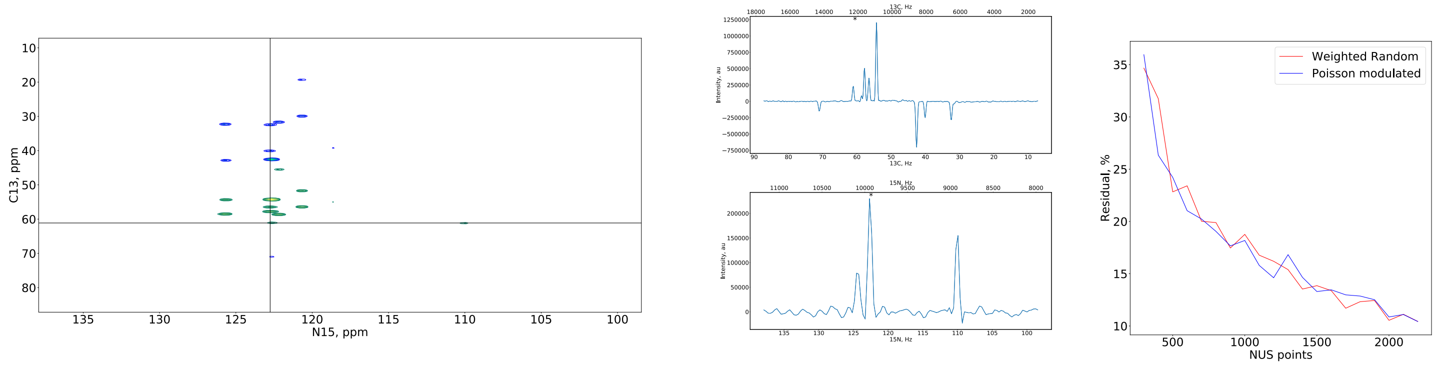

Peak93

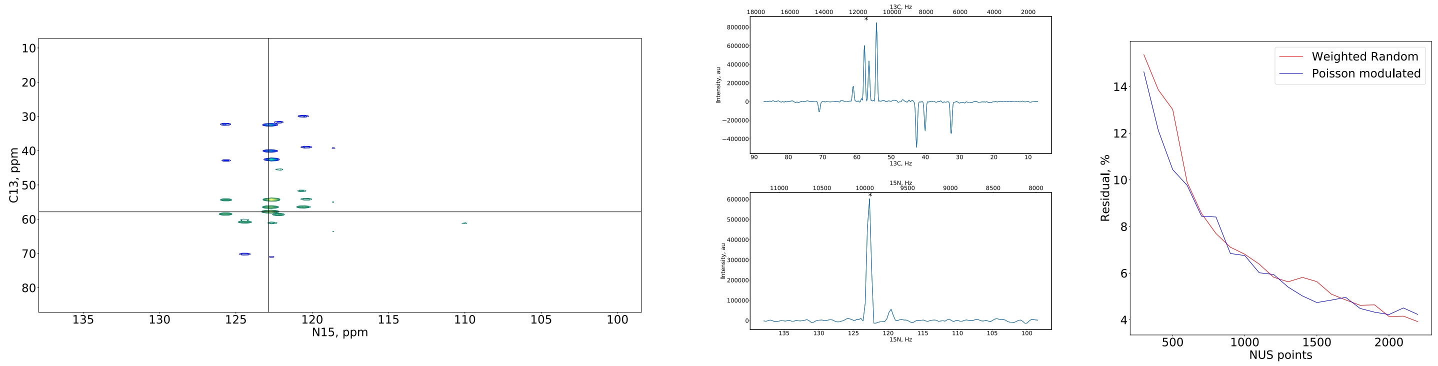

Peak94

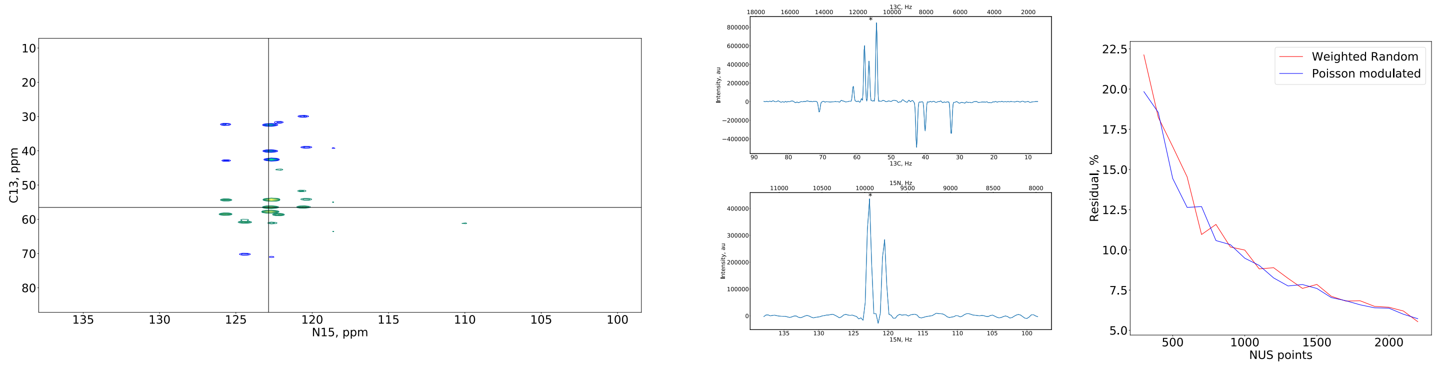

Peak95

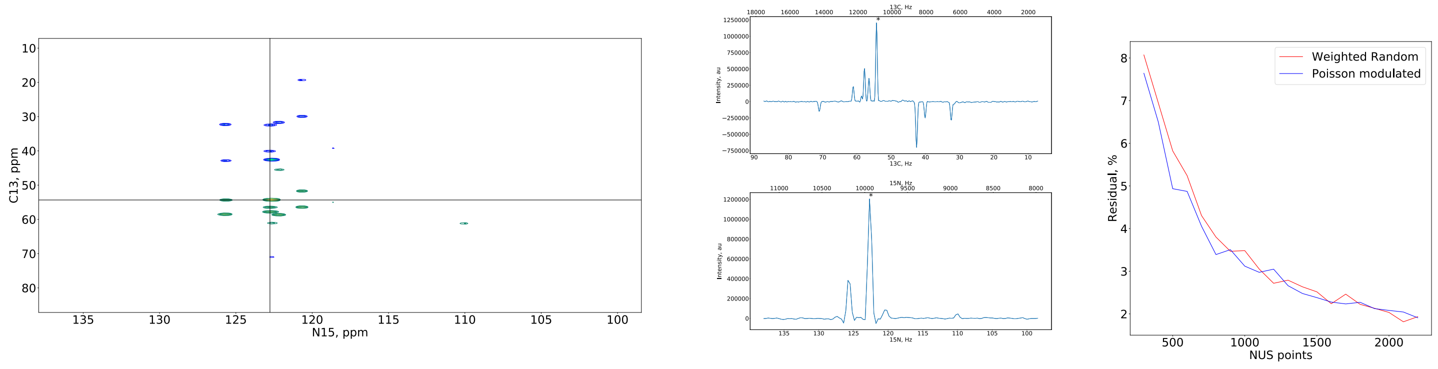

Peak96

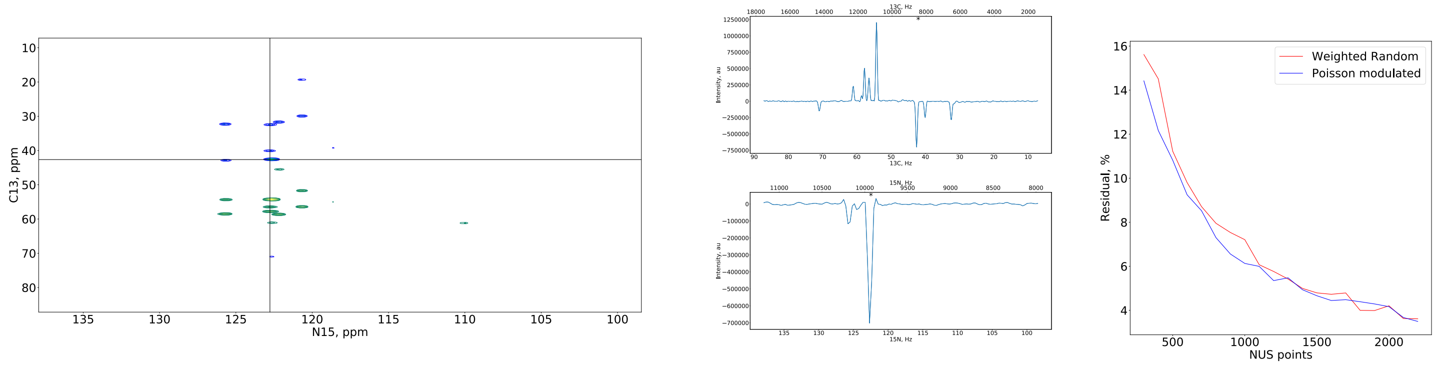

Peak97

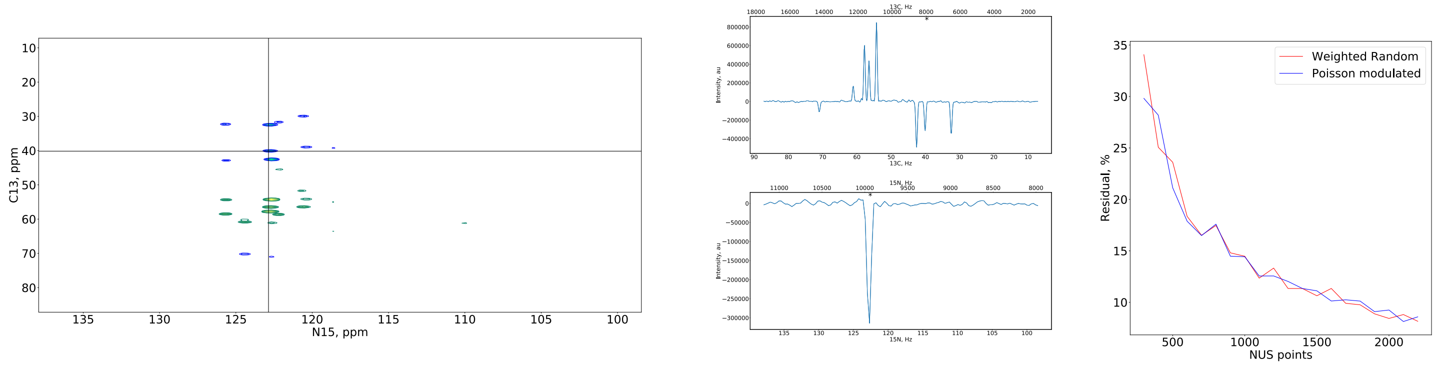

Peak98

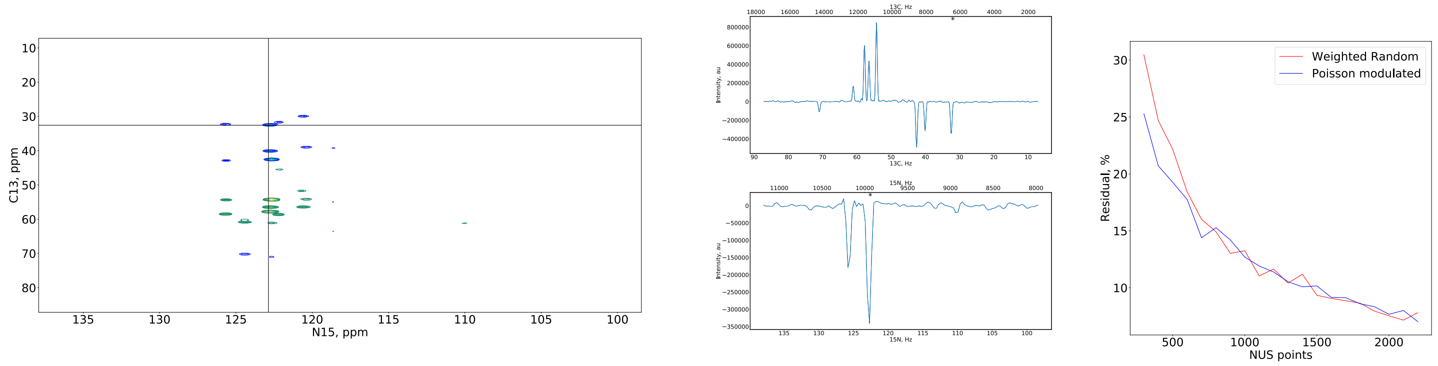

Peak99

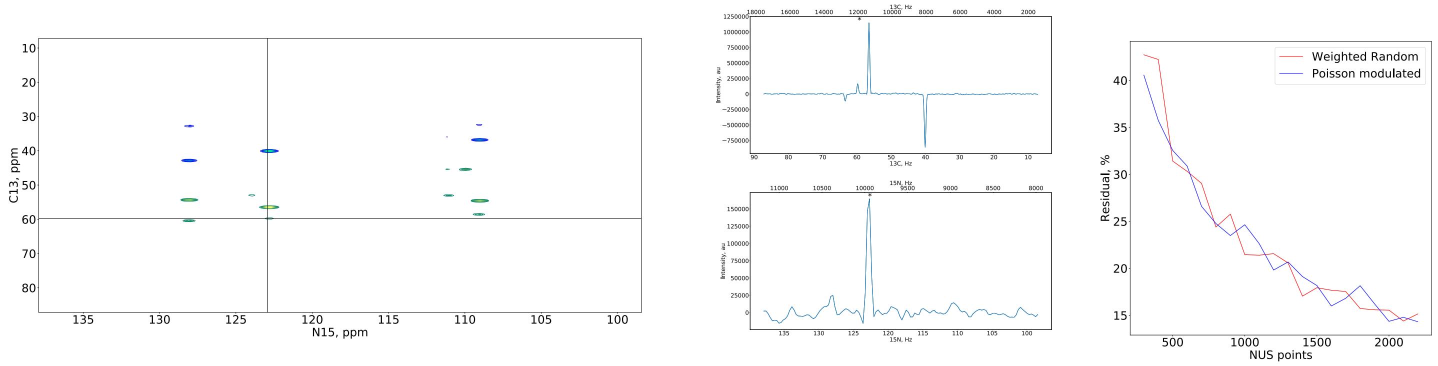

Peak100

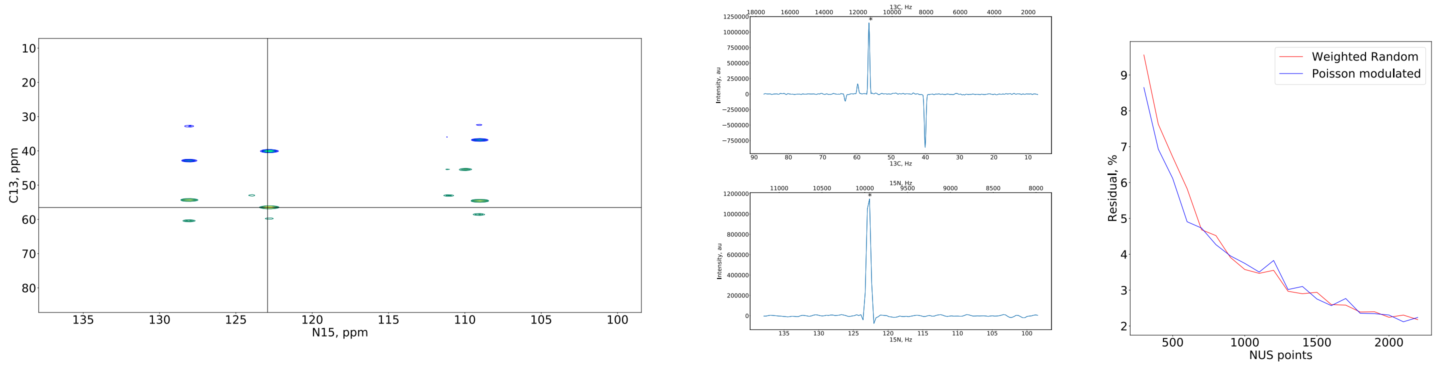

Peak101

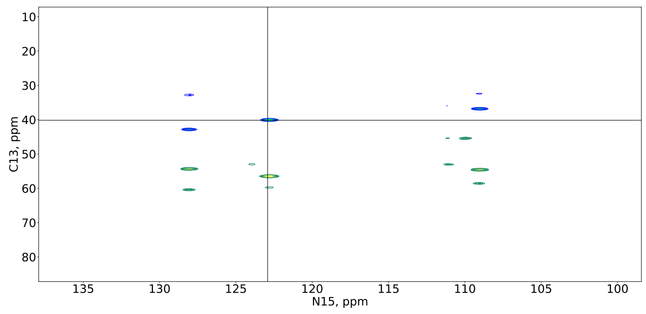

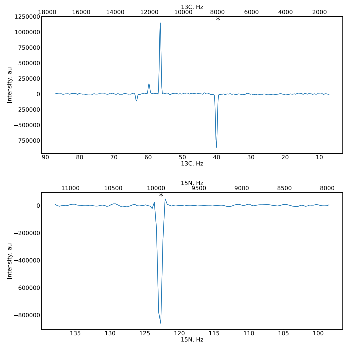

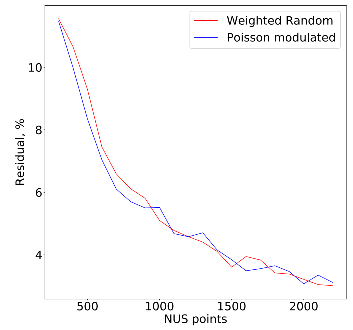

Peak102

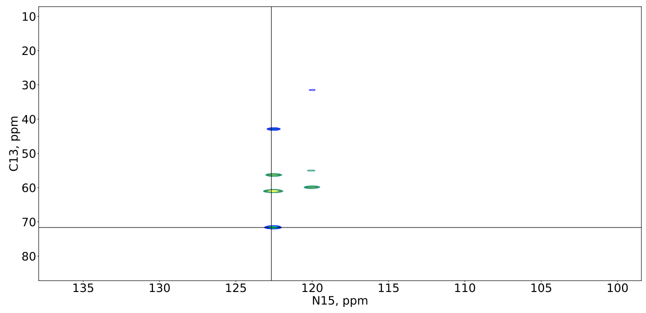

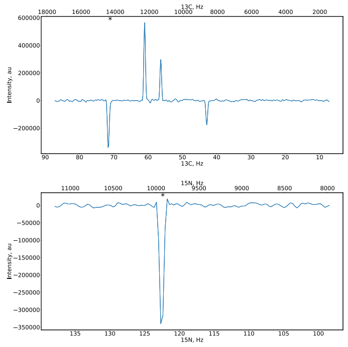

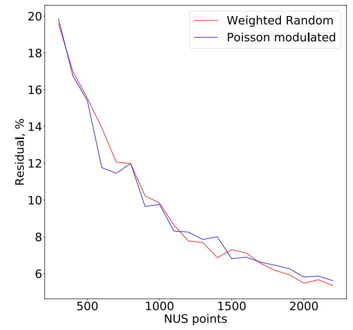

Peak103

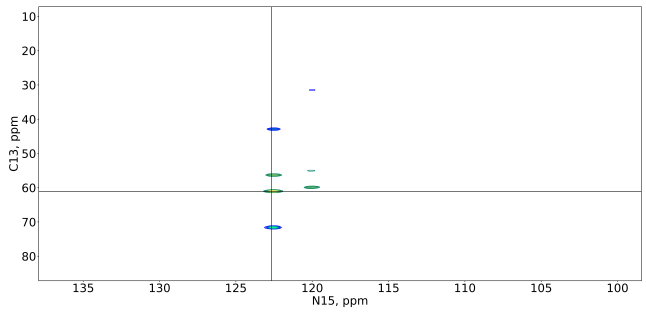

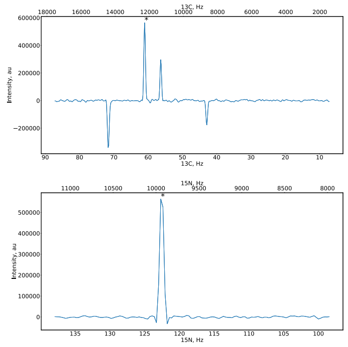

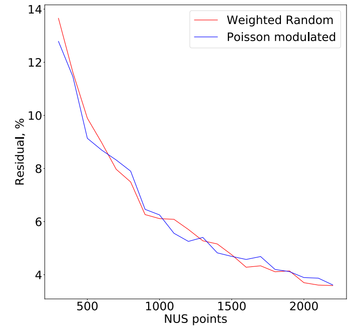

Peak104

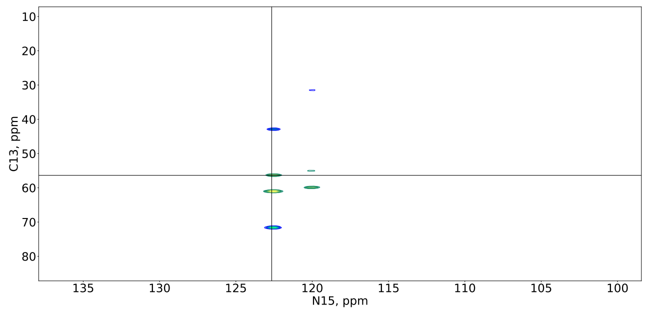

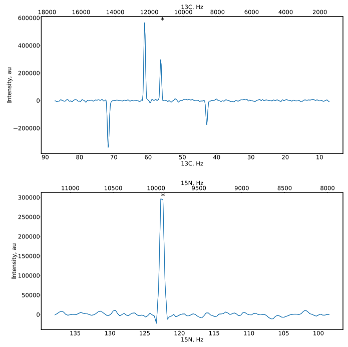

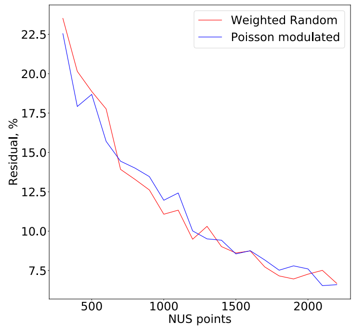

Peak105

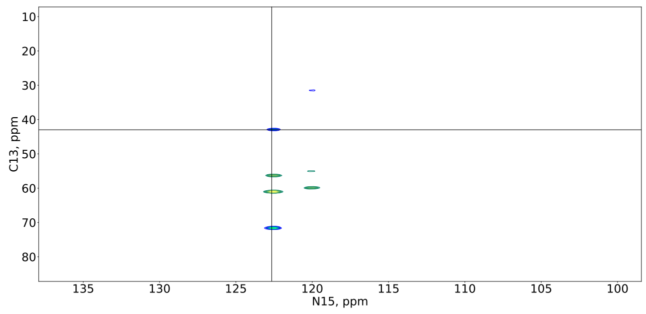

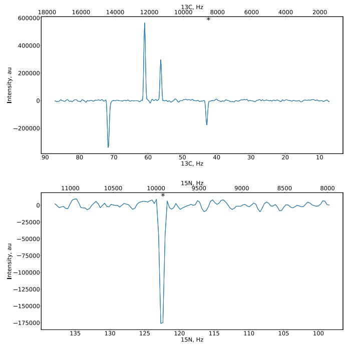

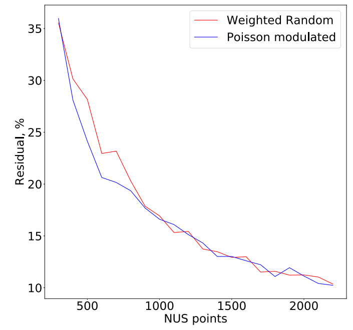

Peak106

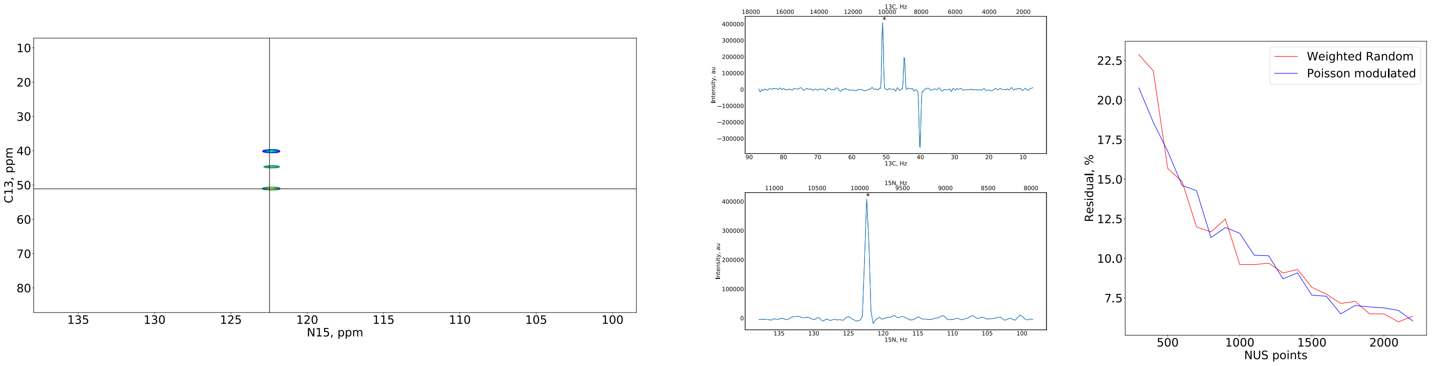

Peak107

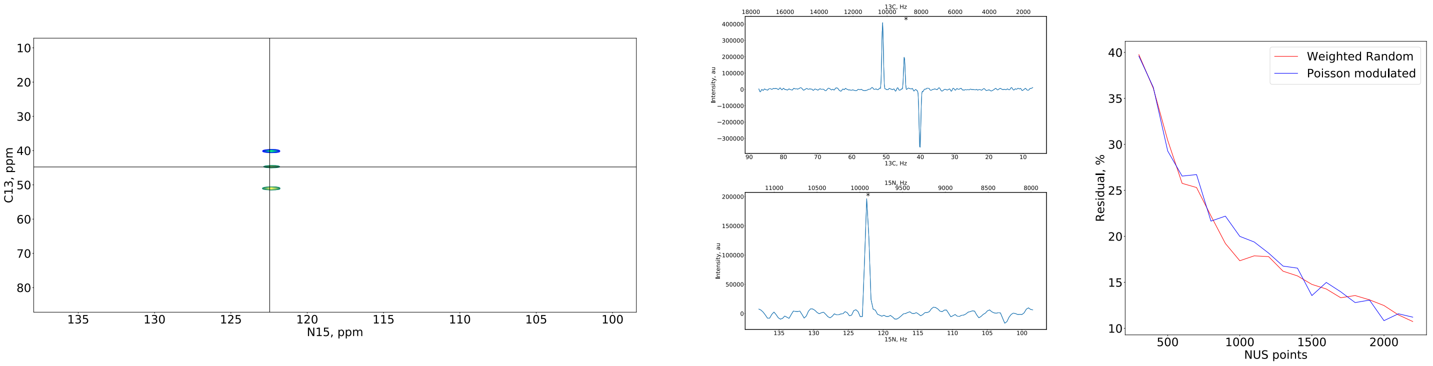

Peak108

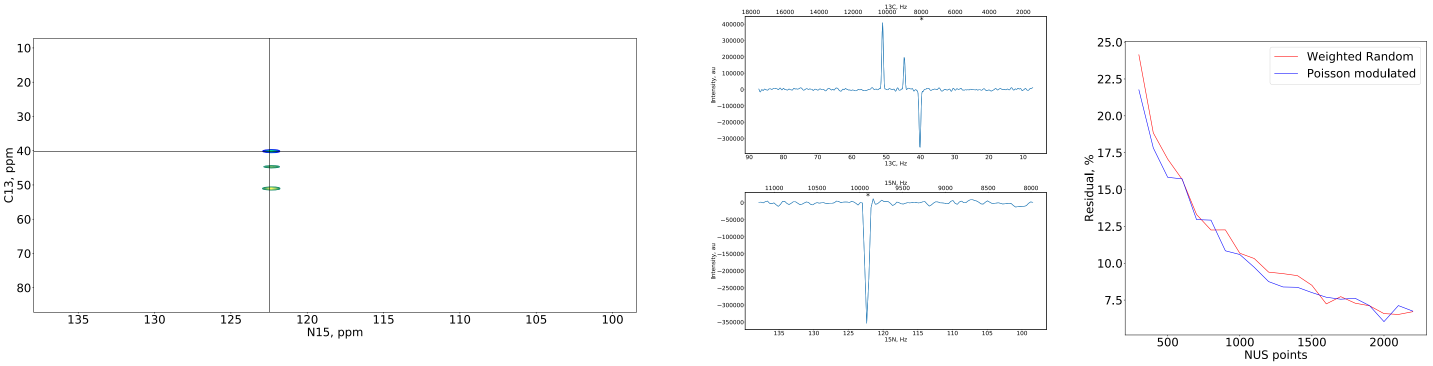

Peak109

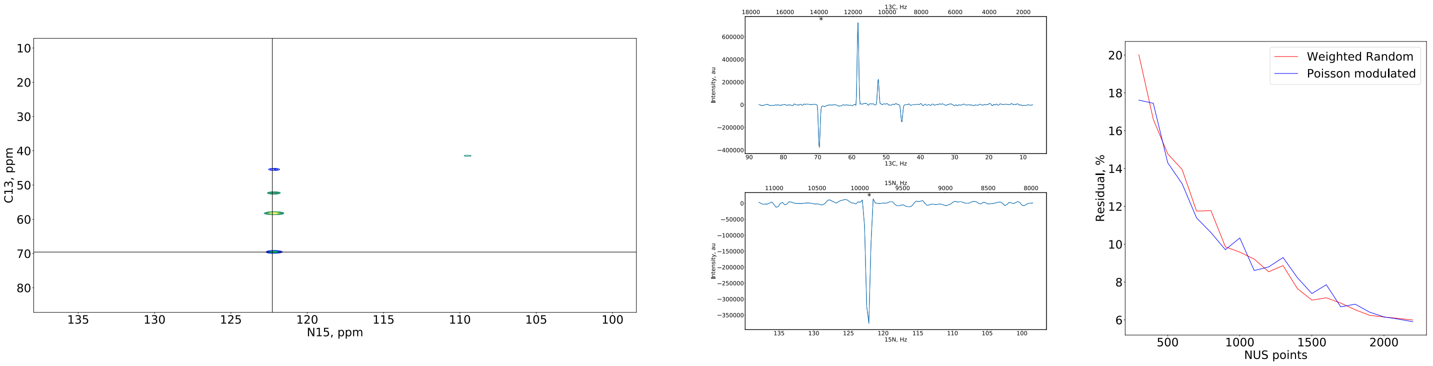

Peak110

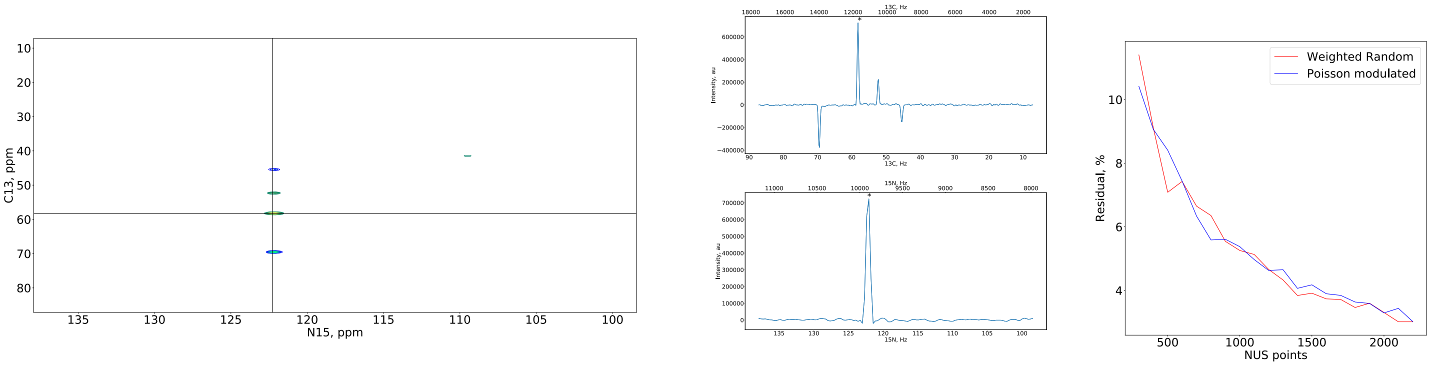

Peak111

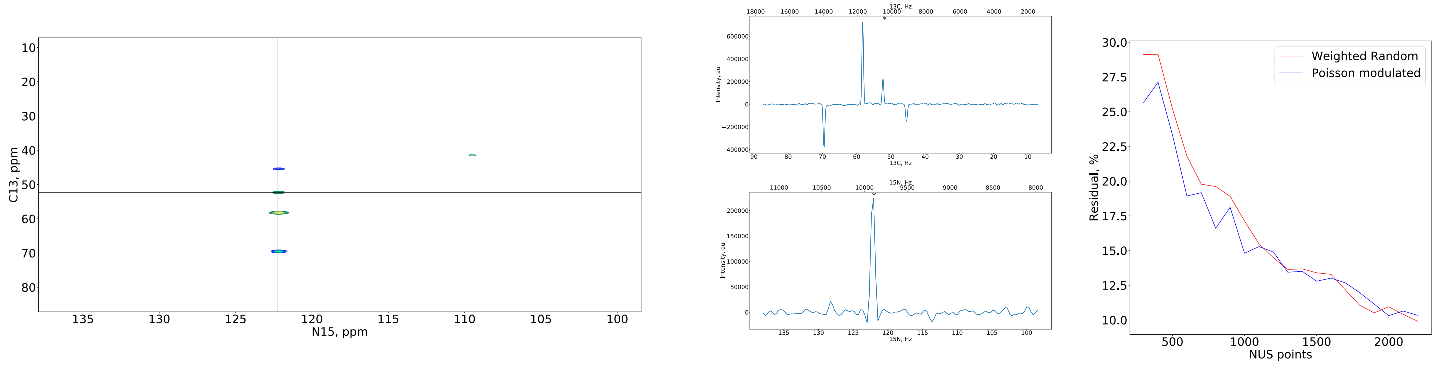

Peak112

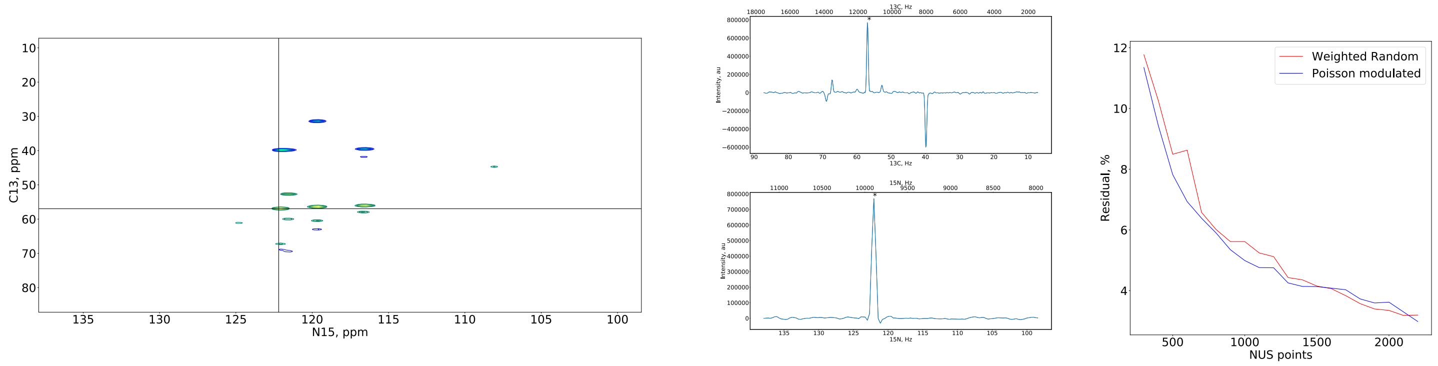

Peak113

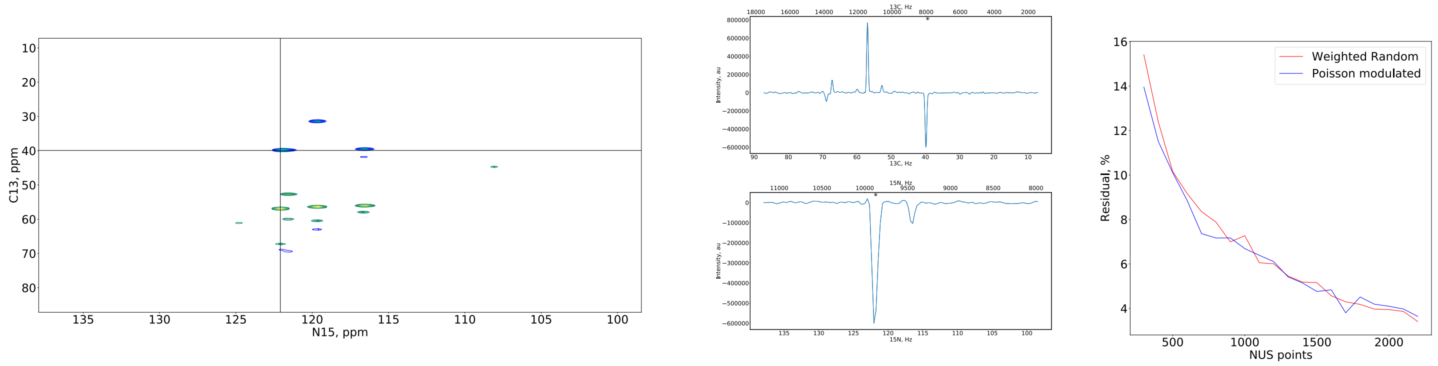

Peak114

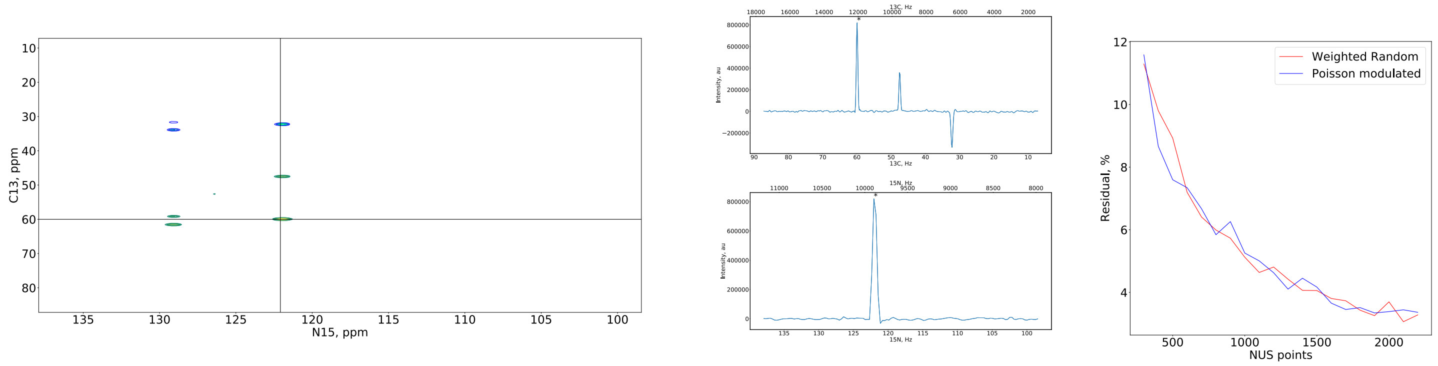

Peak115

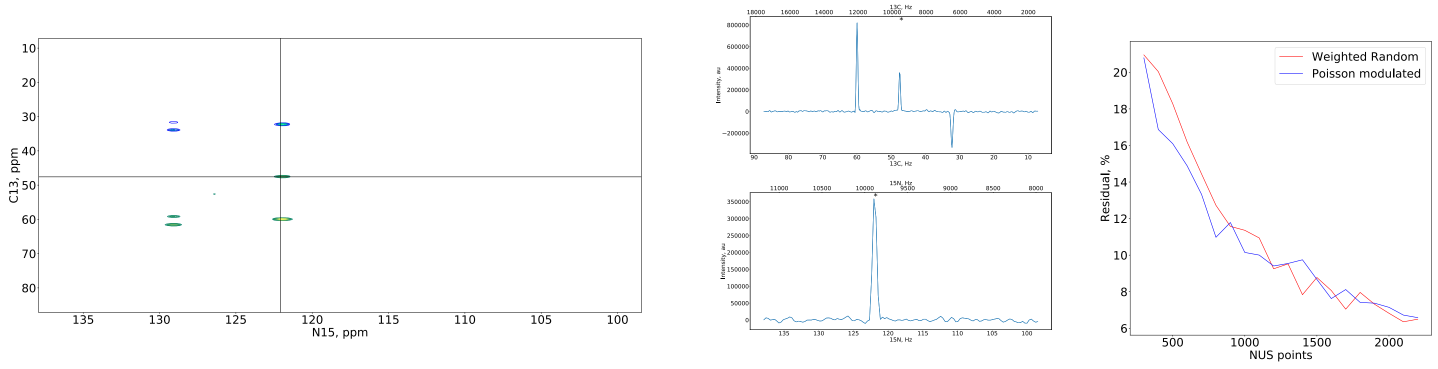

Peak116

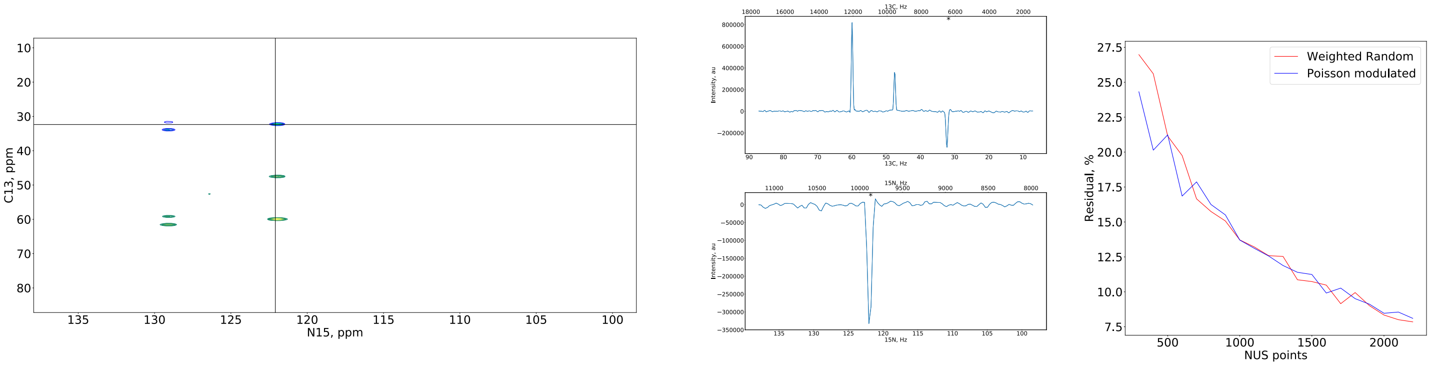

Peak117

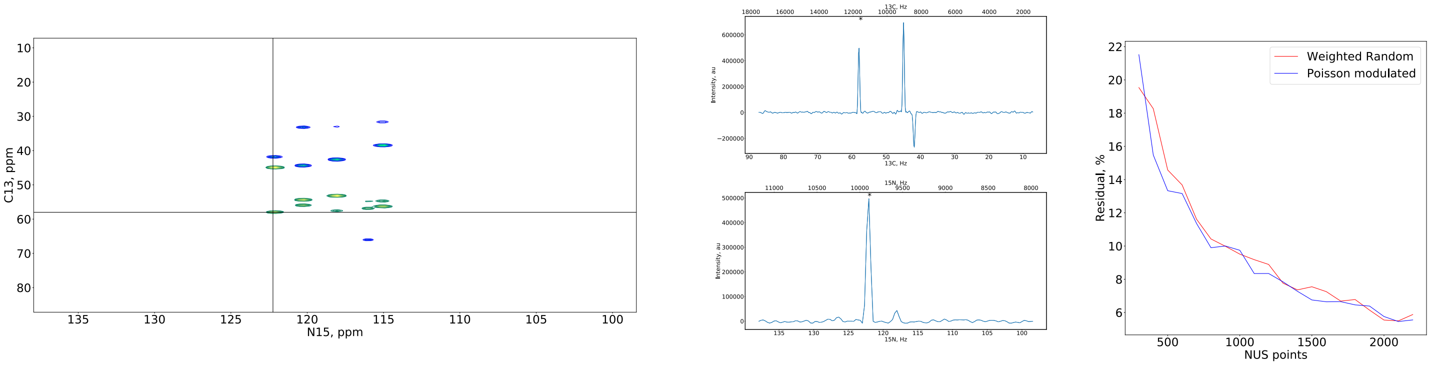

Peak118

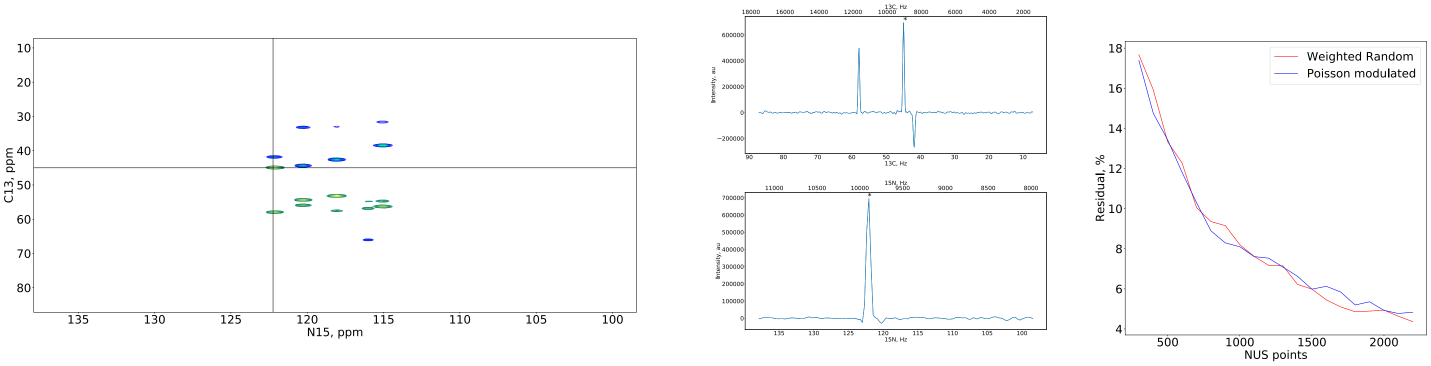

Peak119

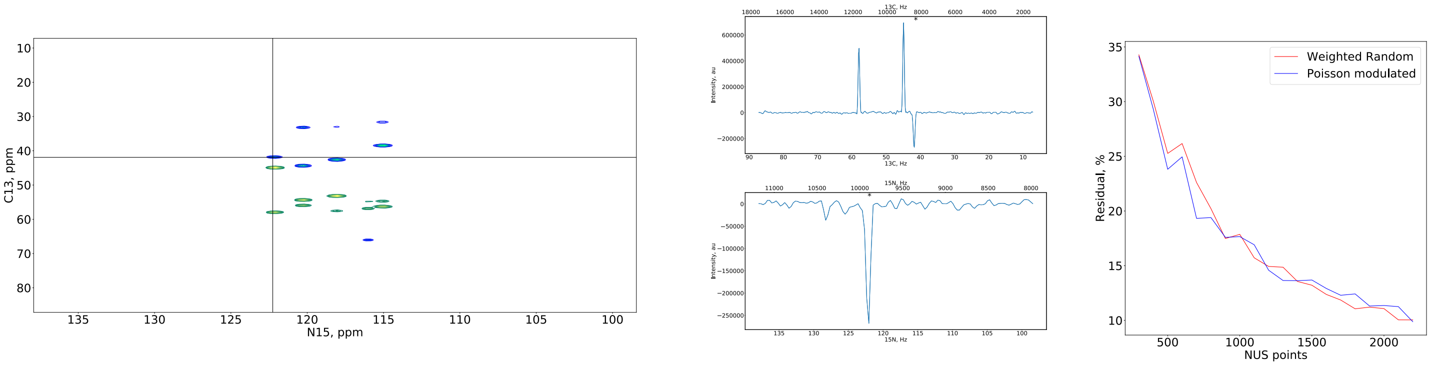

Peak120

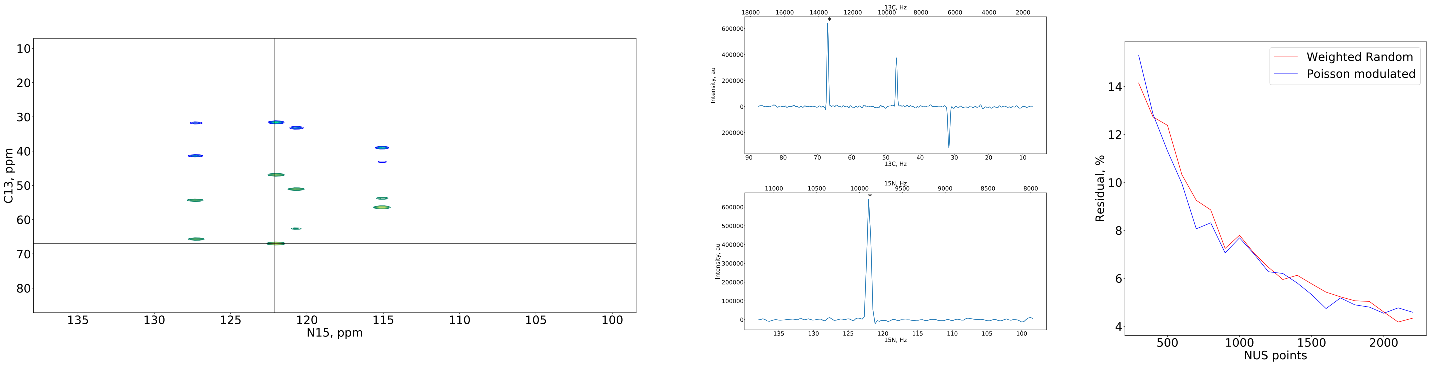

Peak121

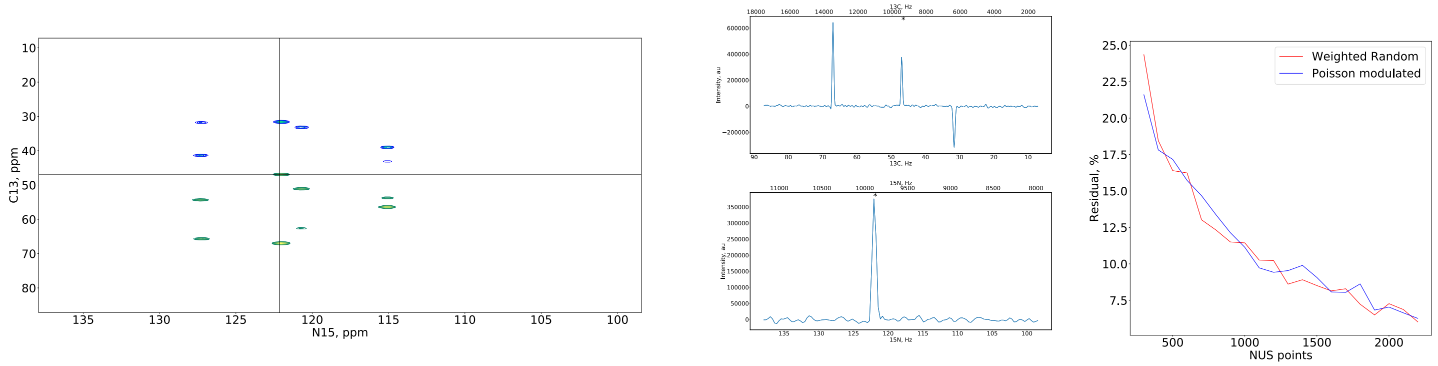

Peak122

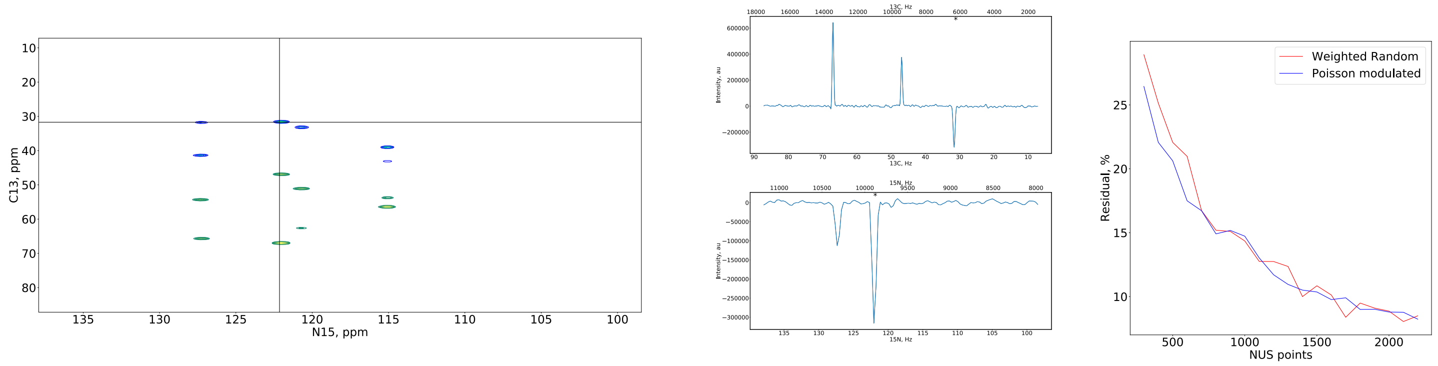

Peak123

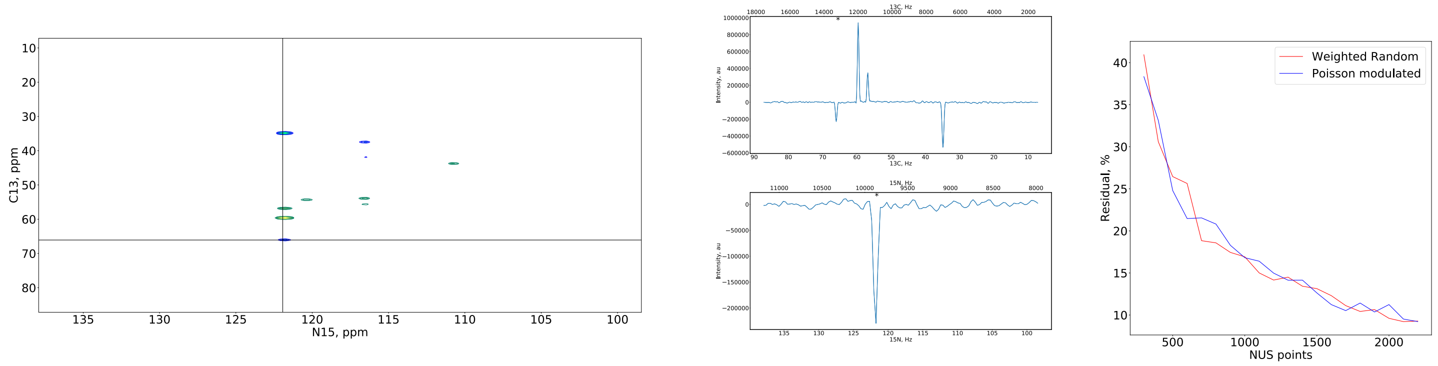

Peak124

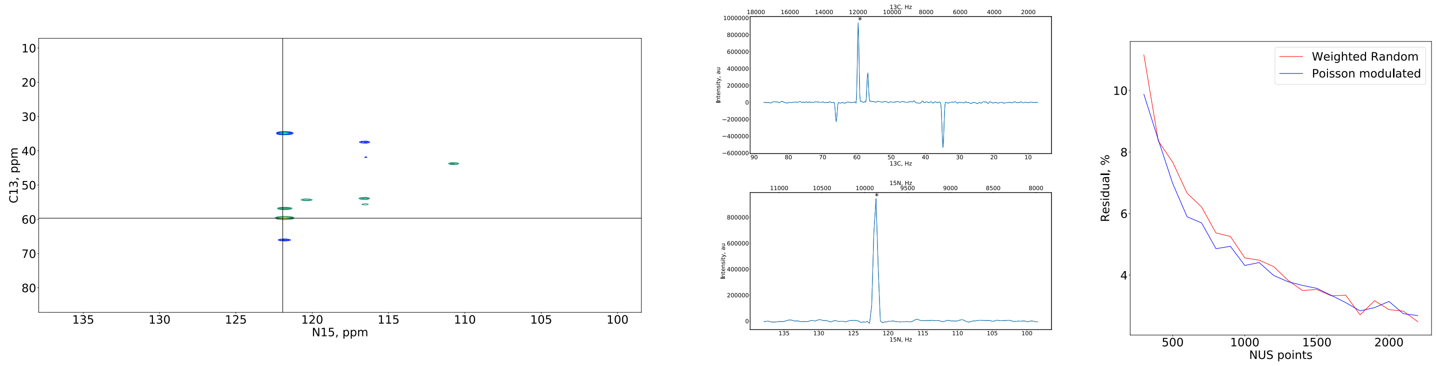

Peak125

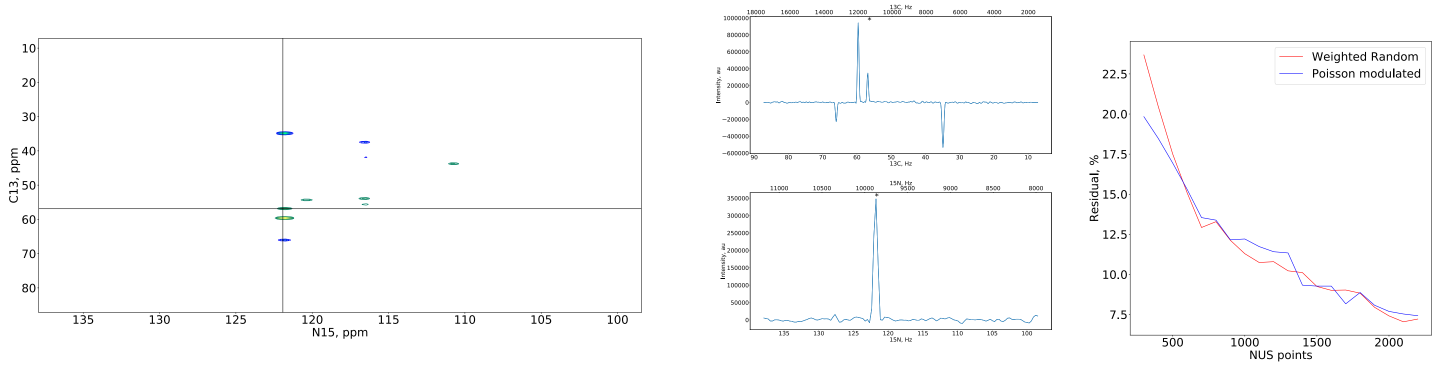

Peak126

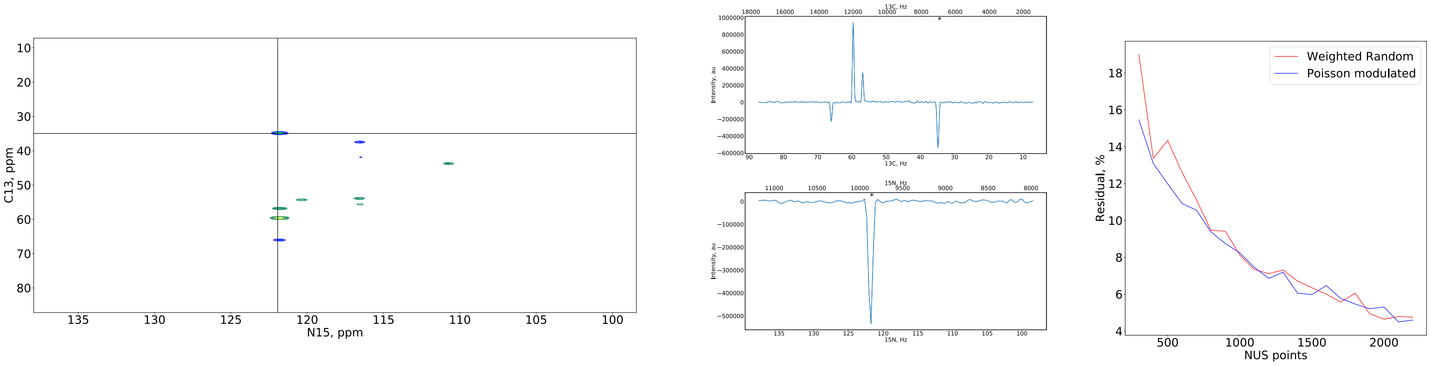

Peak127

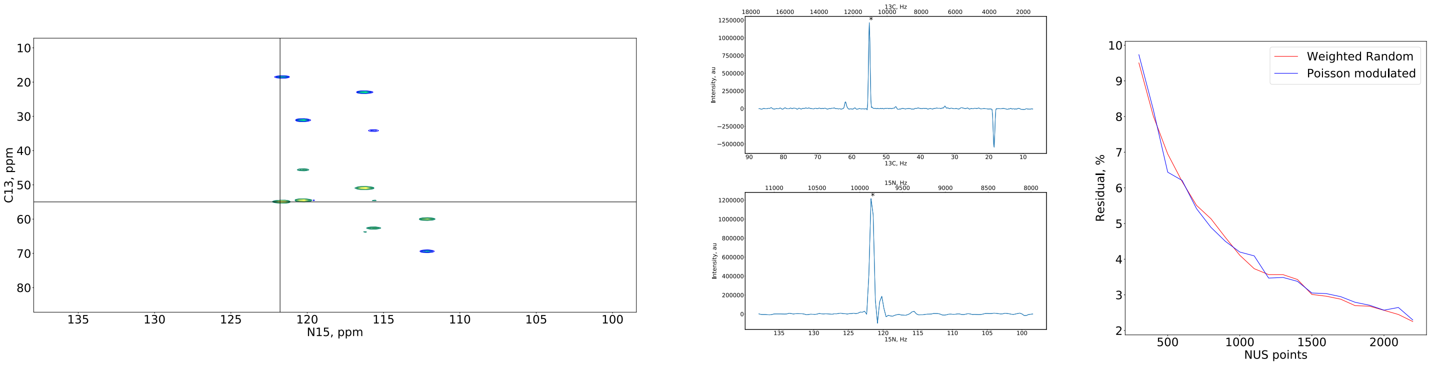

Peak128

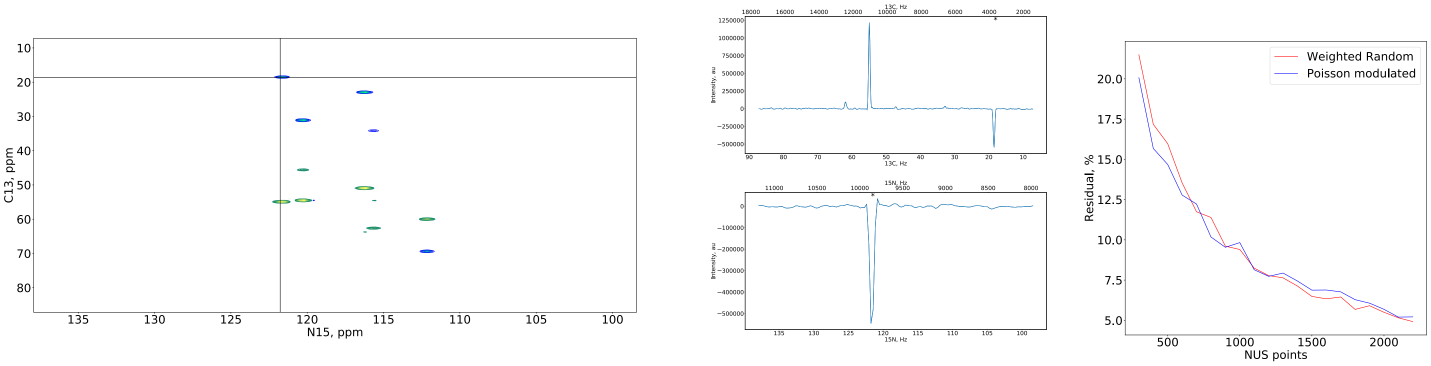

Peak129

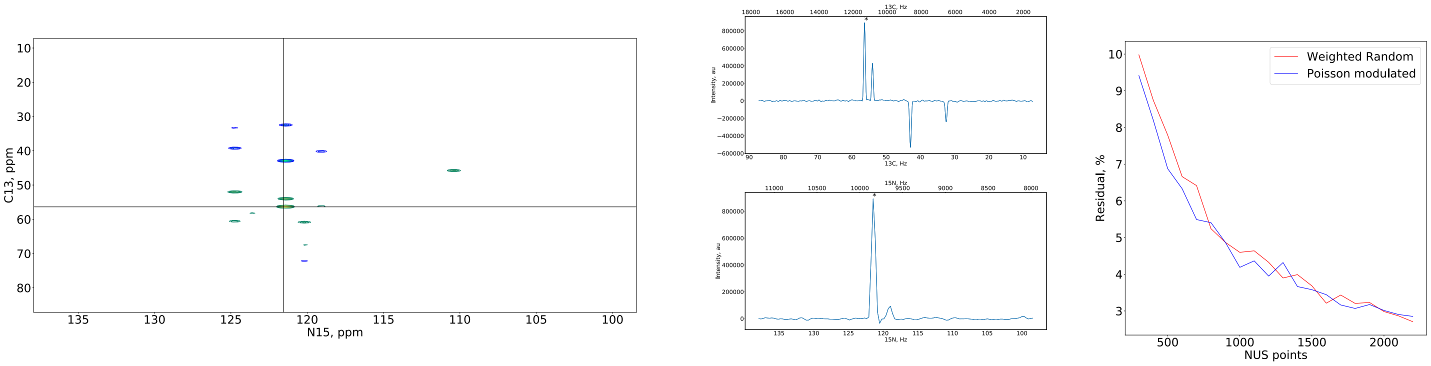

Peak130

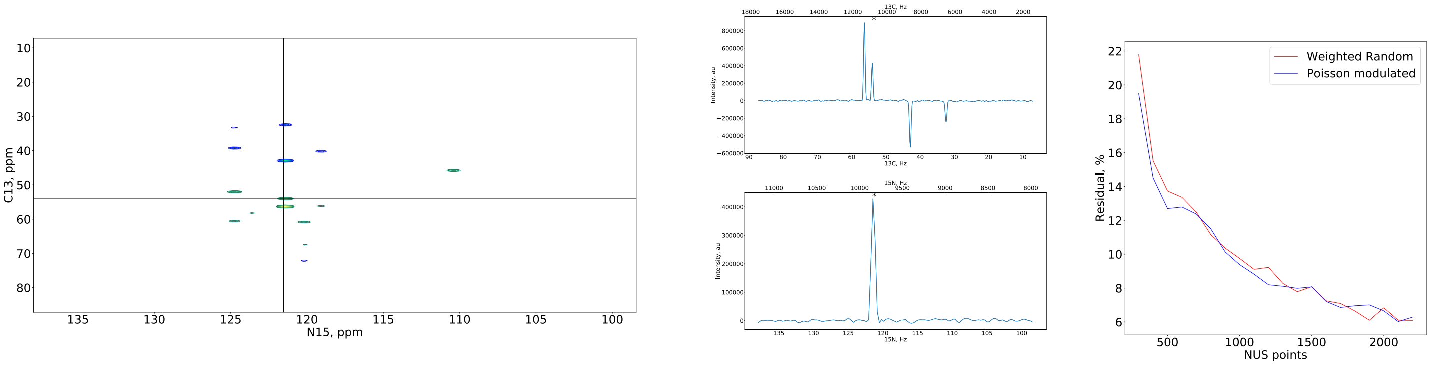

Peak131

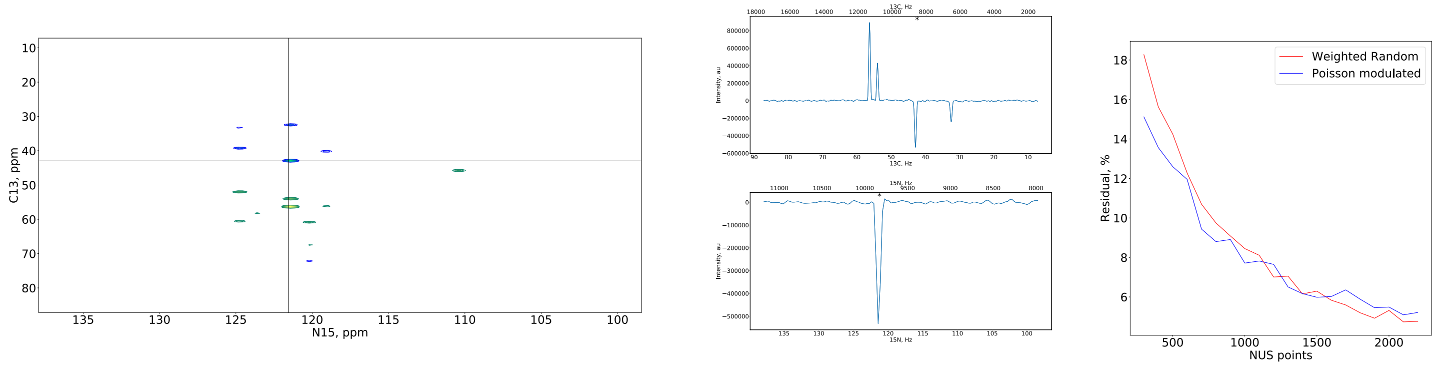

Peak132

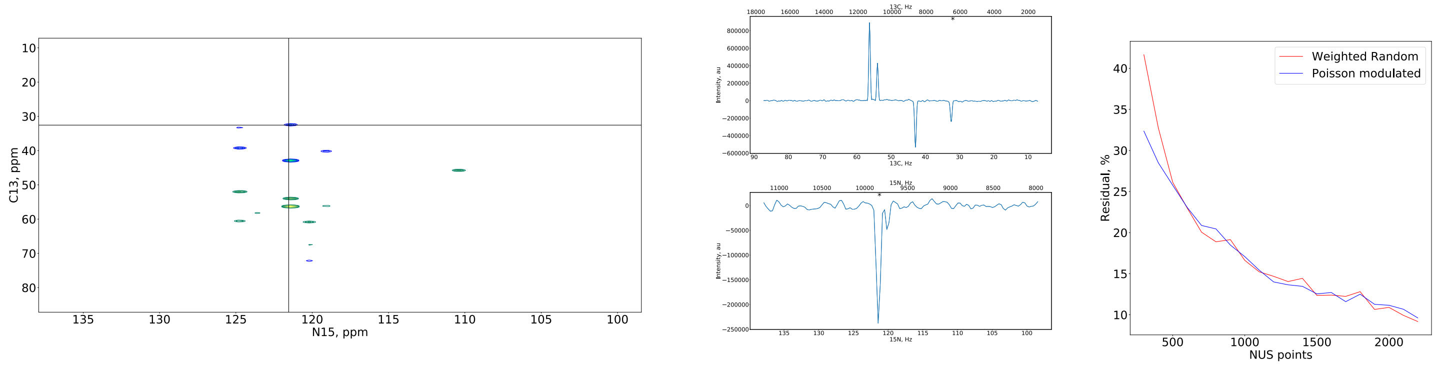

Peak133

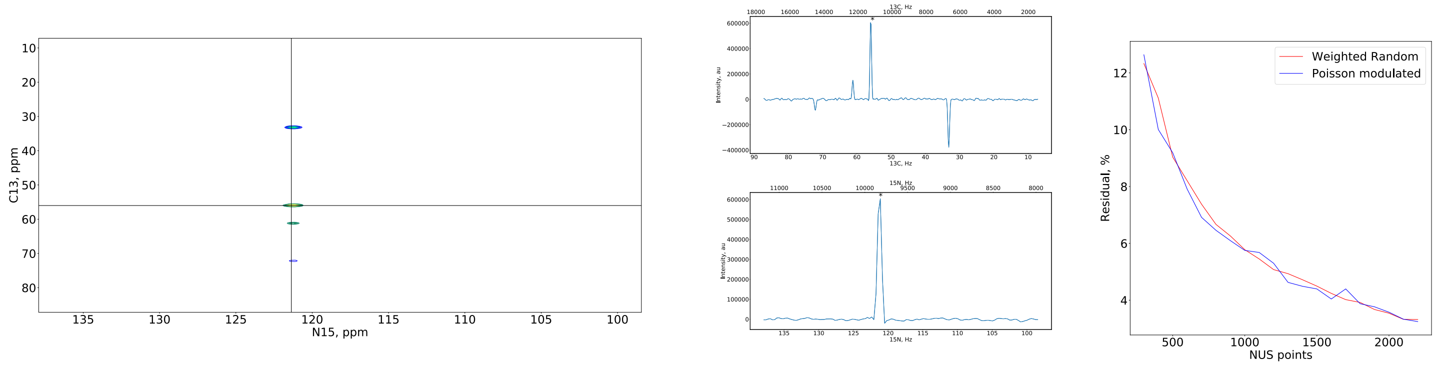

Peak134

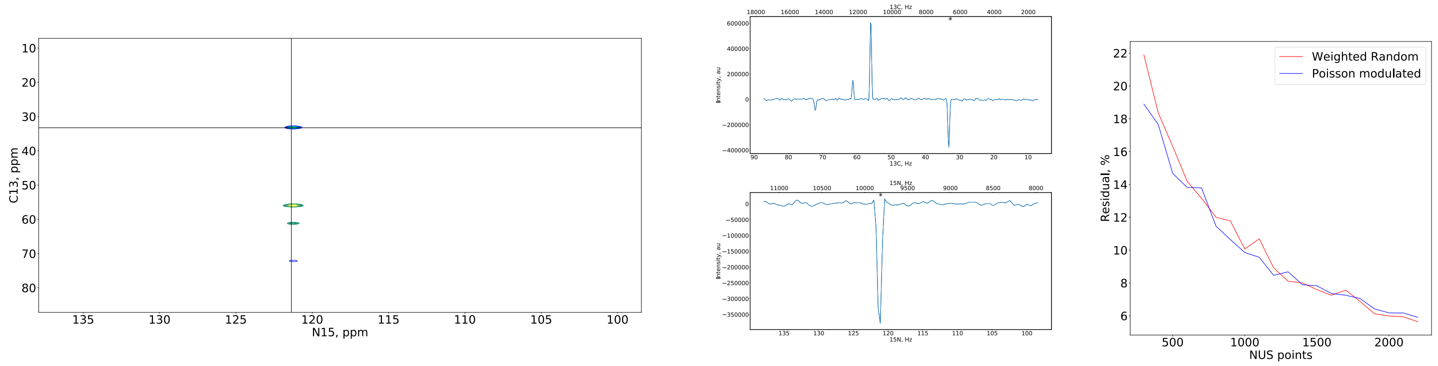

Peak135

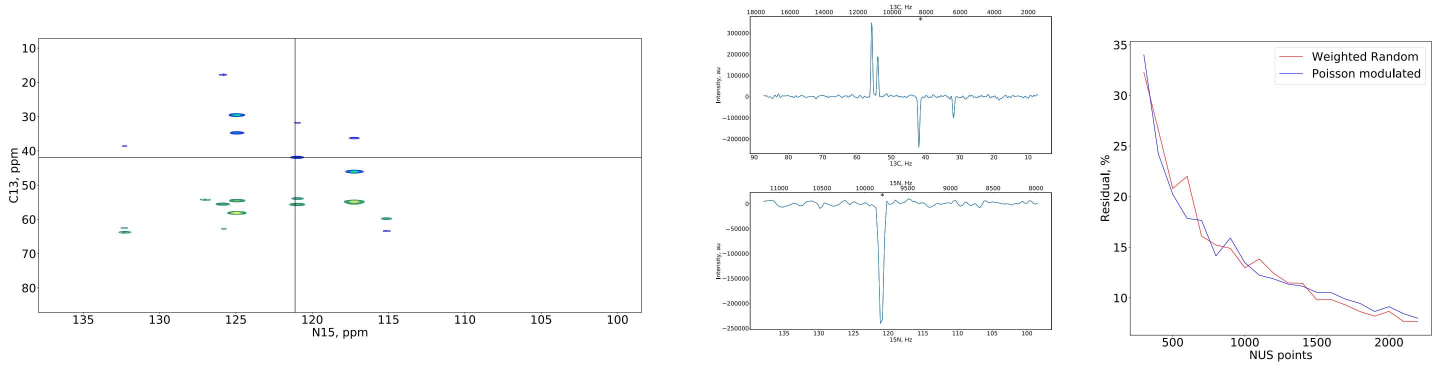

Peak136

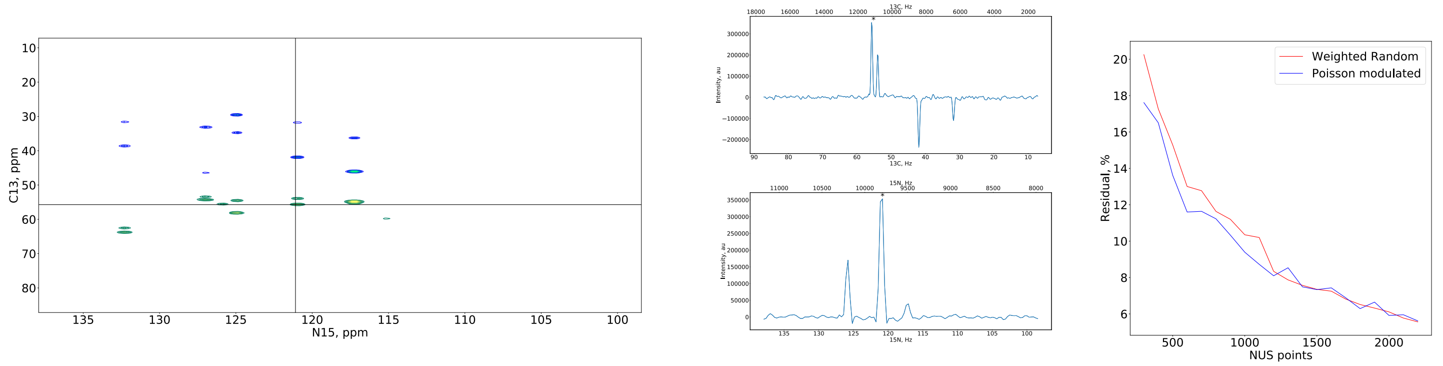

Peak137

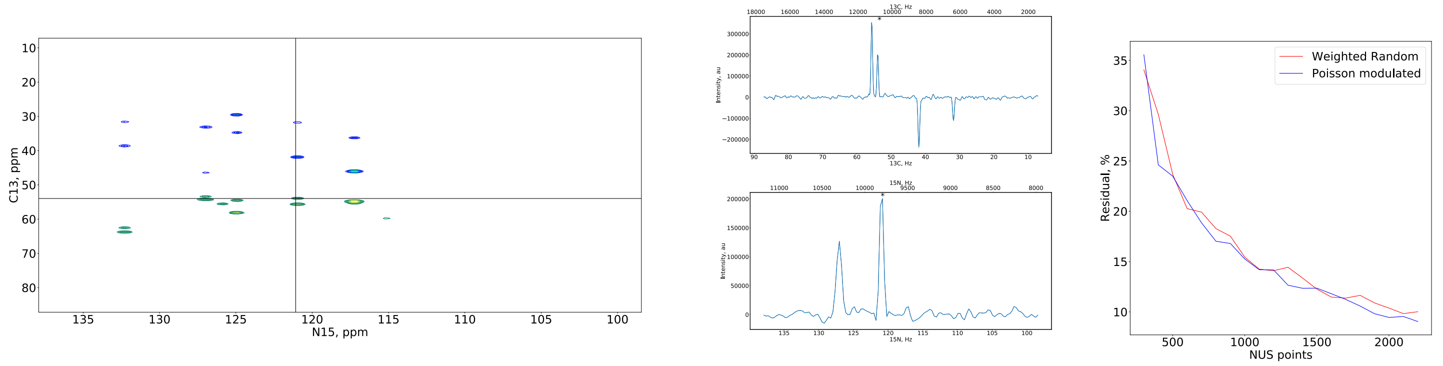

Peak138

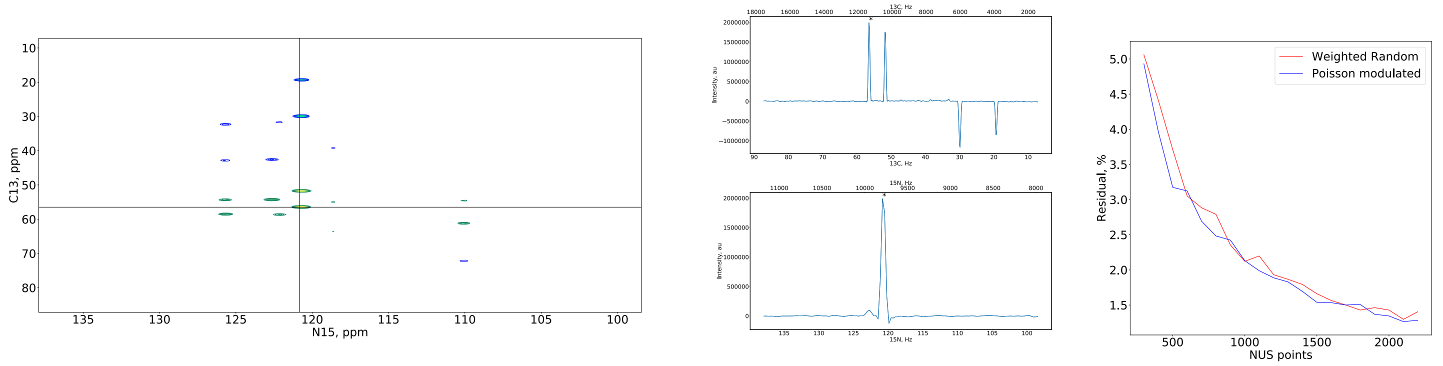

Peak139

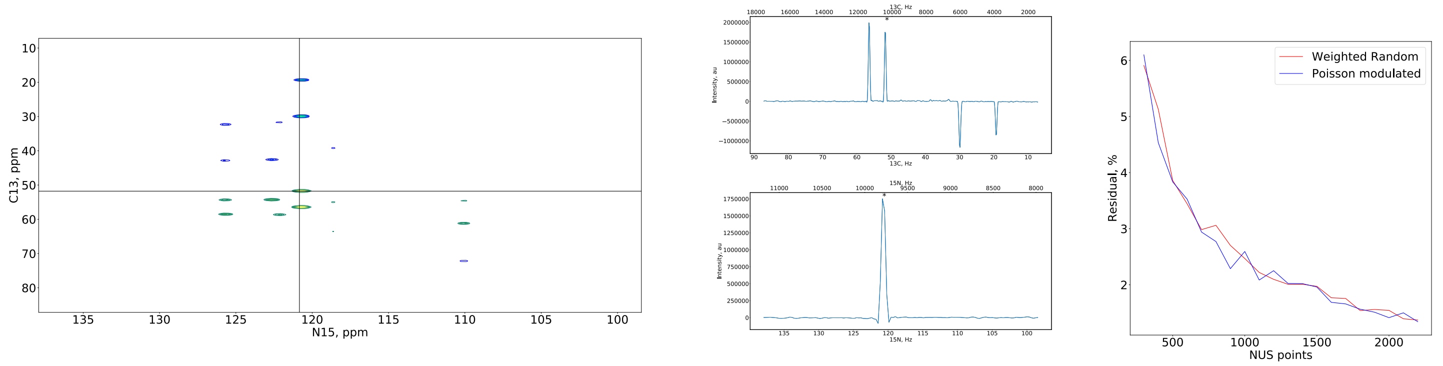

Peak140

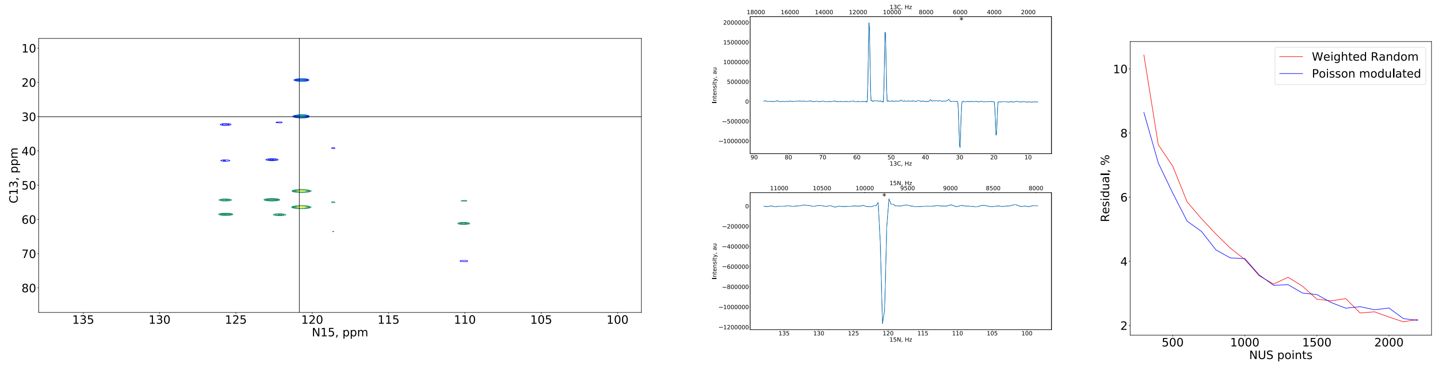

Peak141

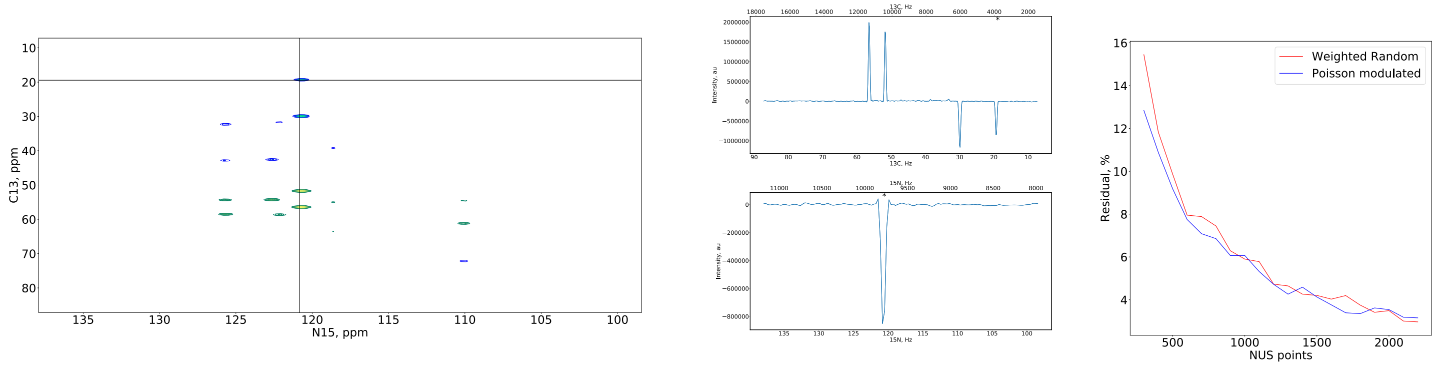

Peak142

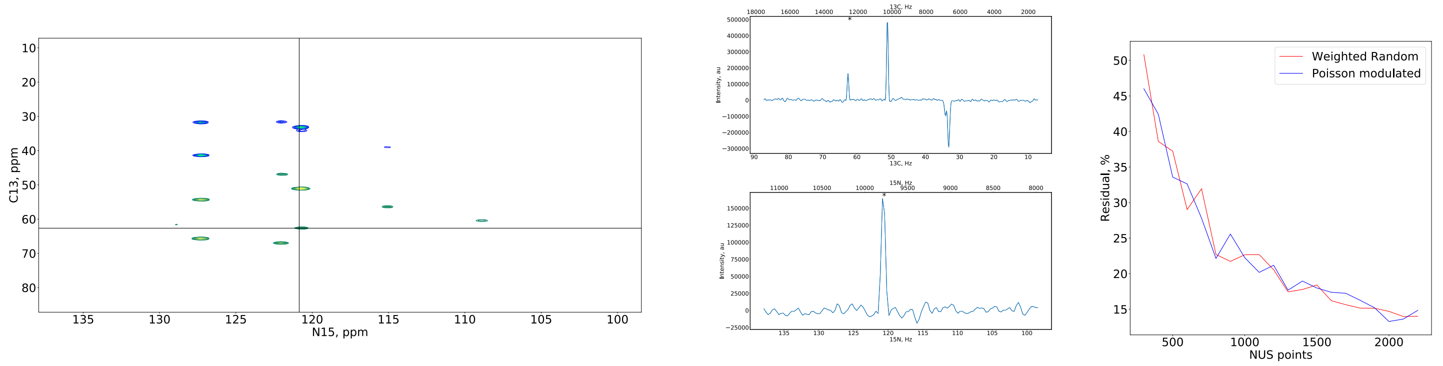

Peak143

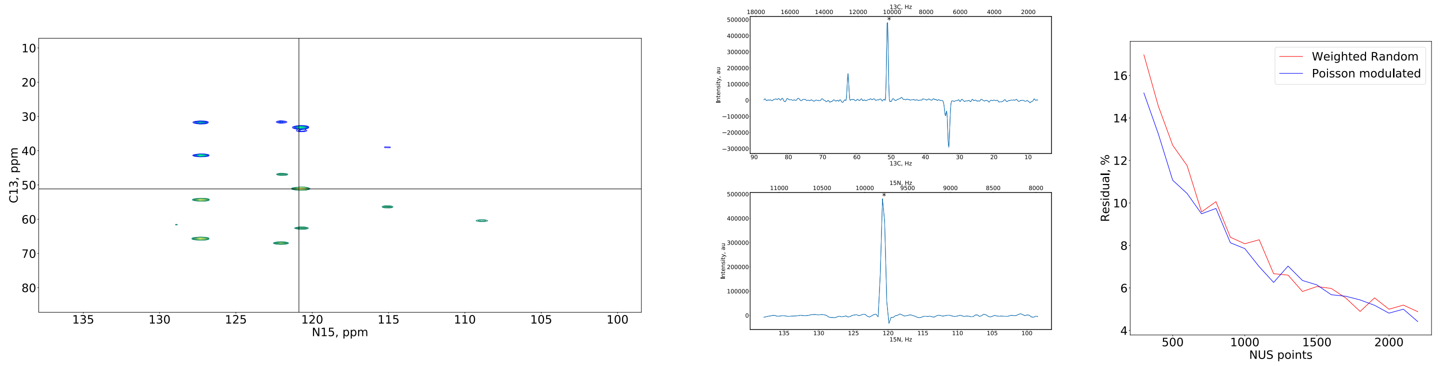

Peak144

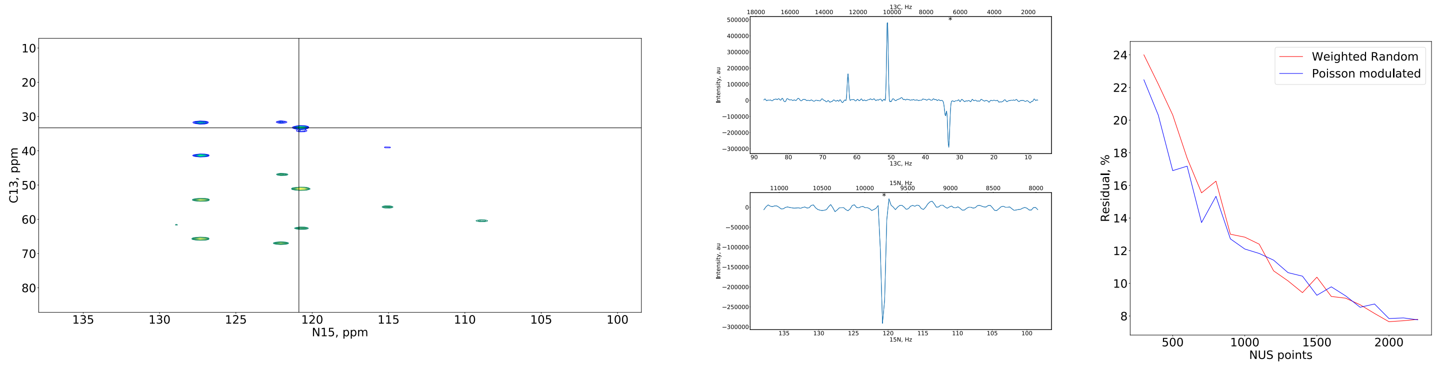

Peak145

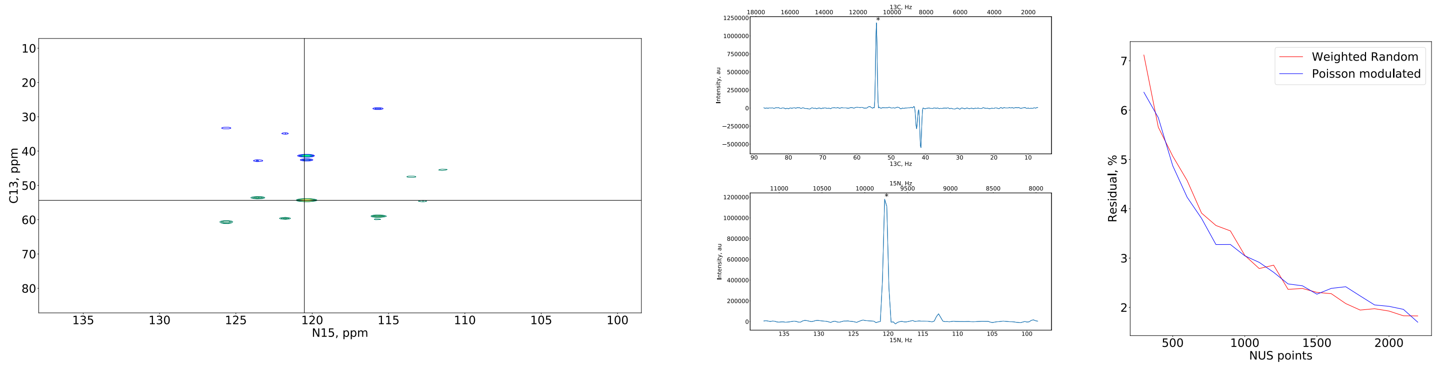

Peak146

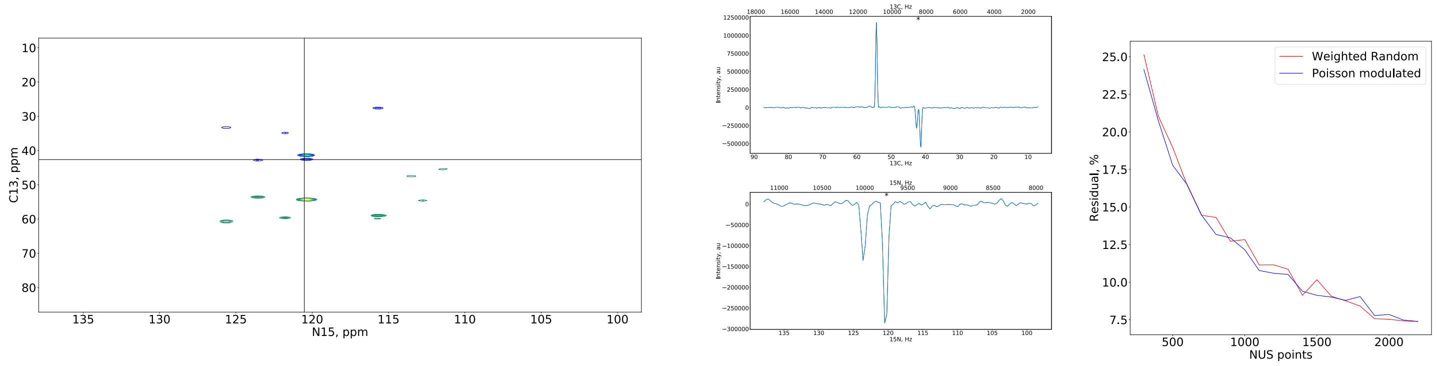

Peak147

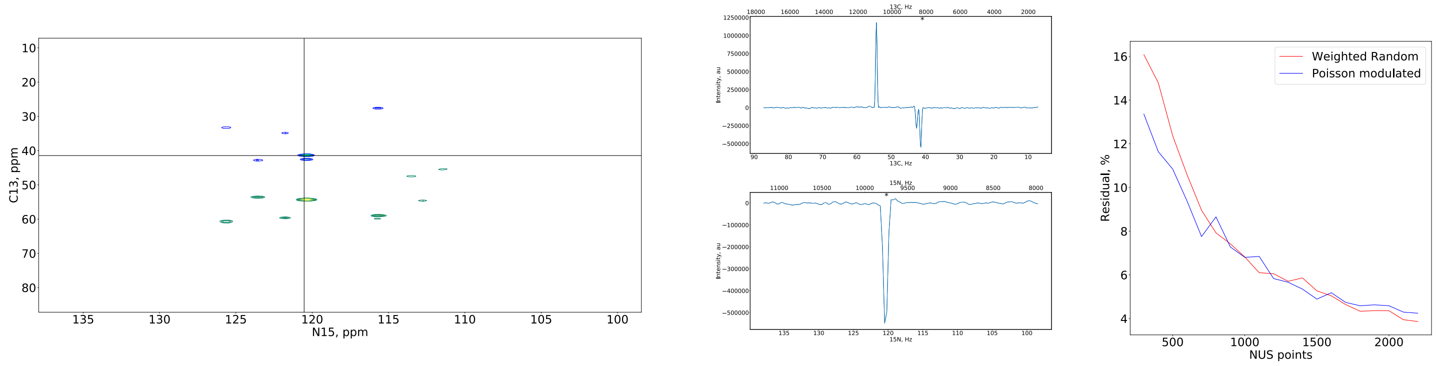

Peak148

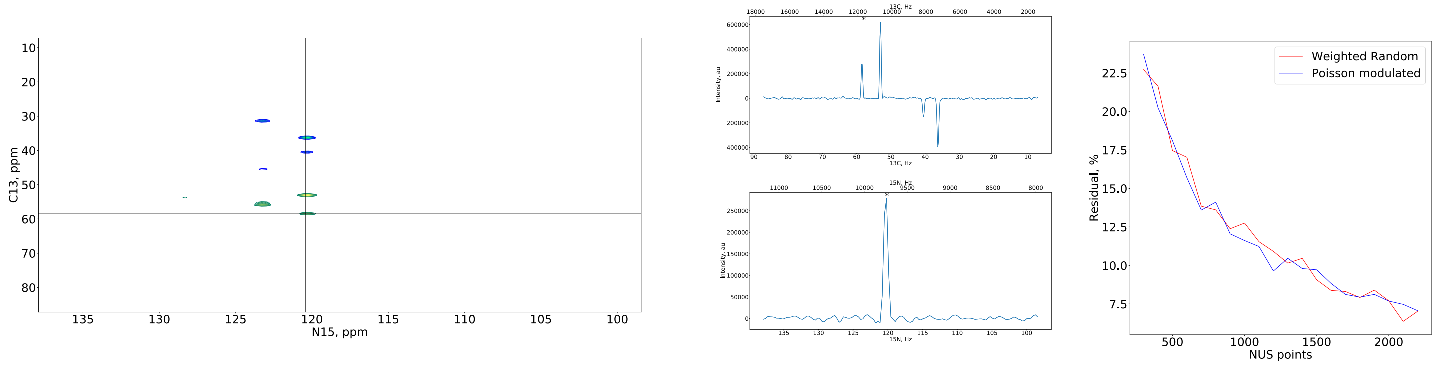

Peak149

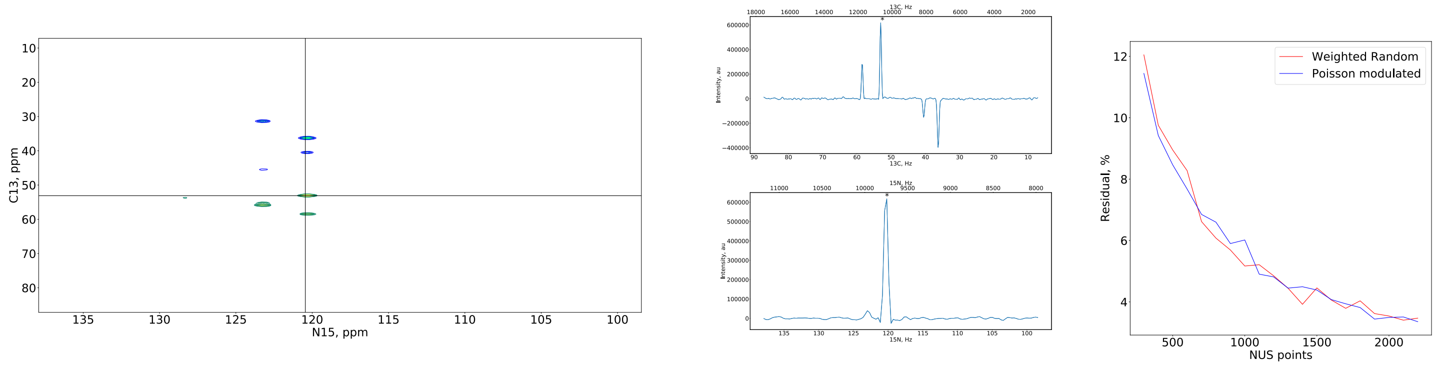

Peak150

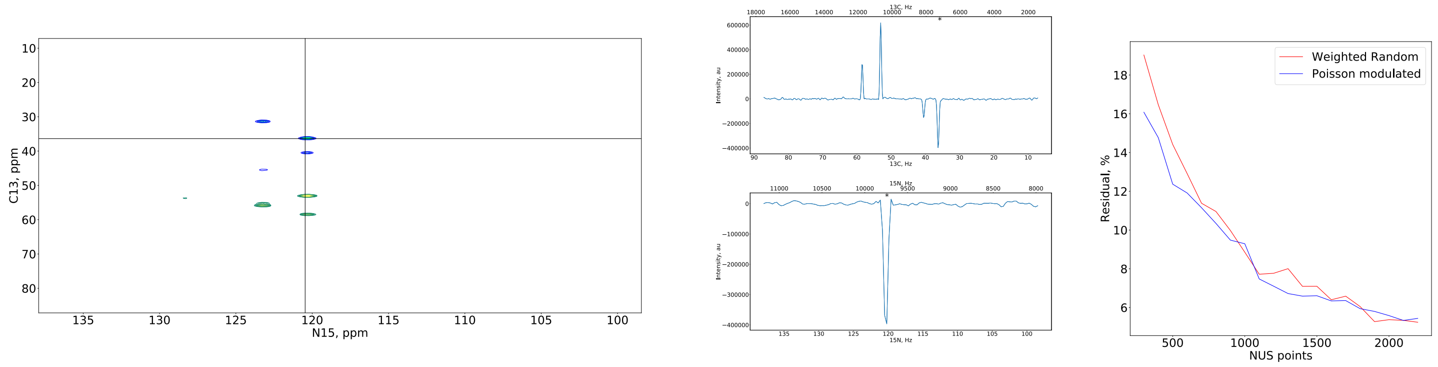

Peak151

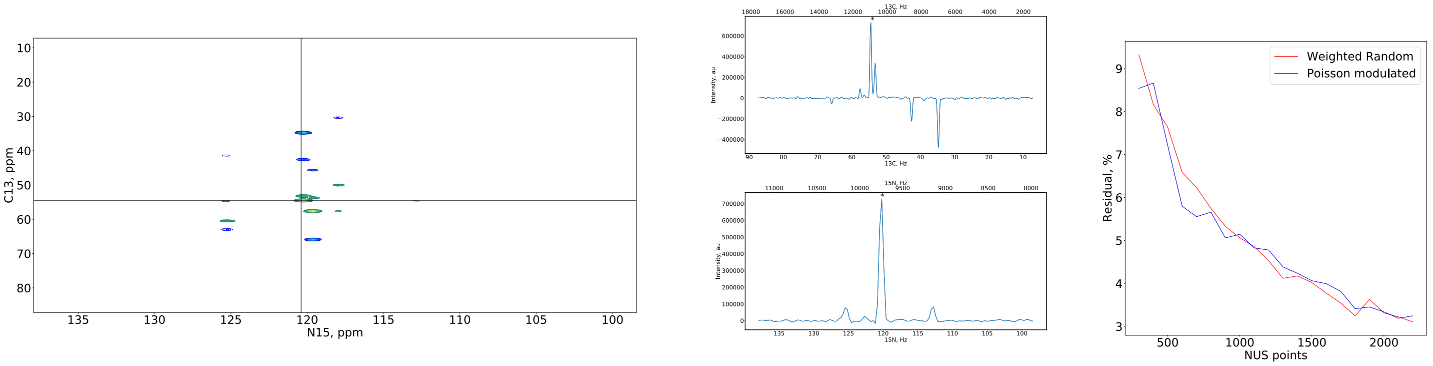

Peak152

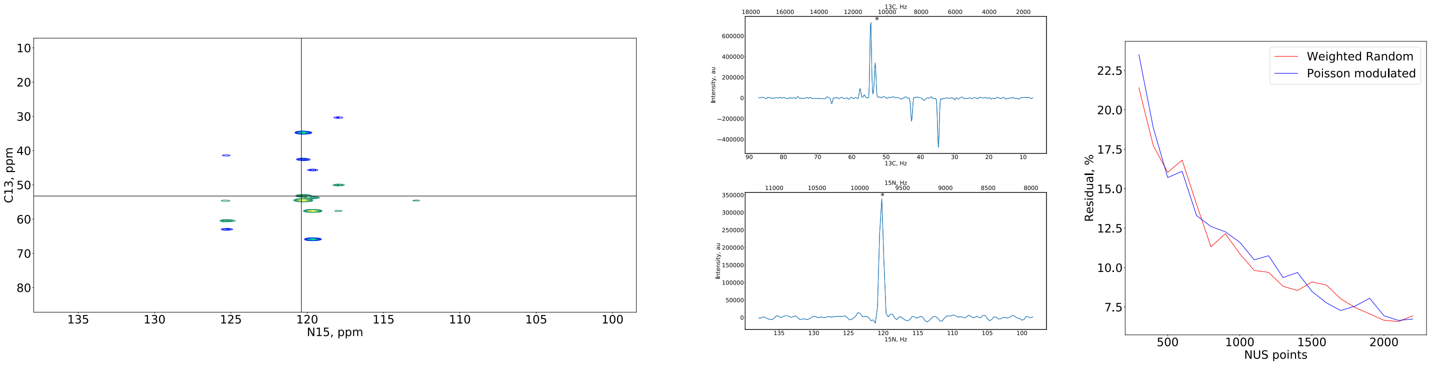

Peak153

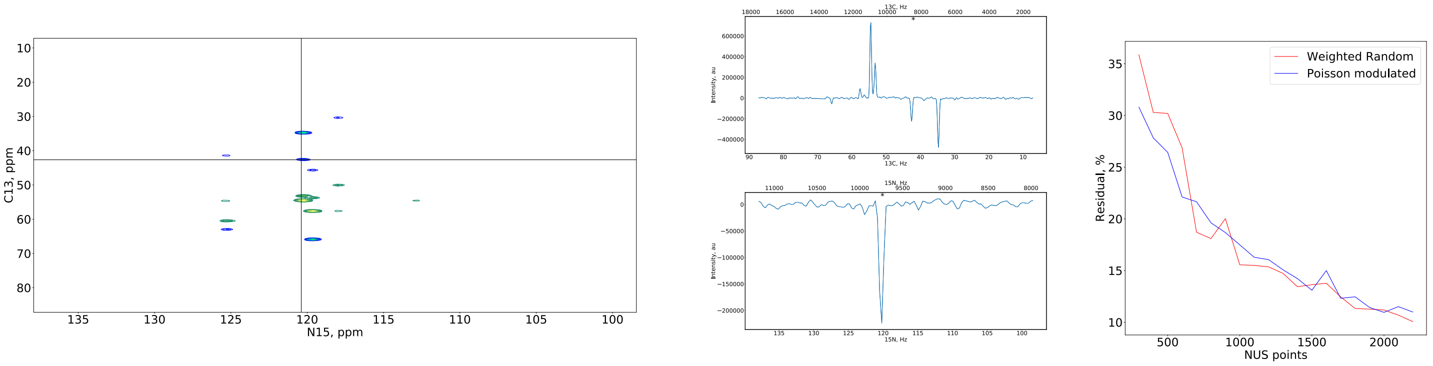

Peak154

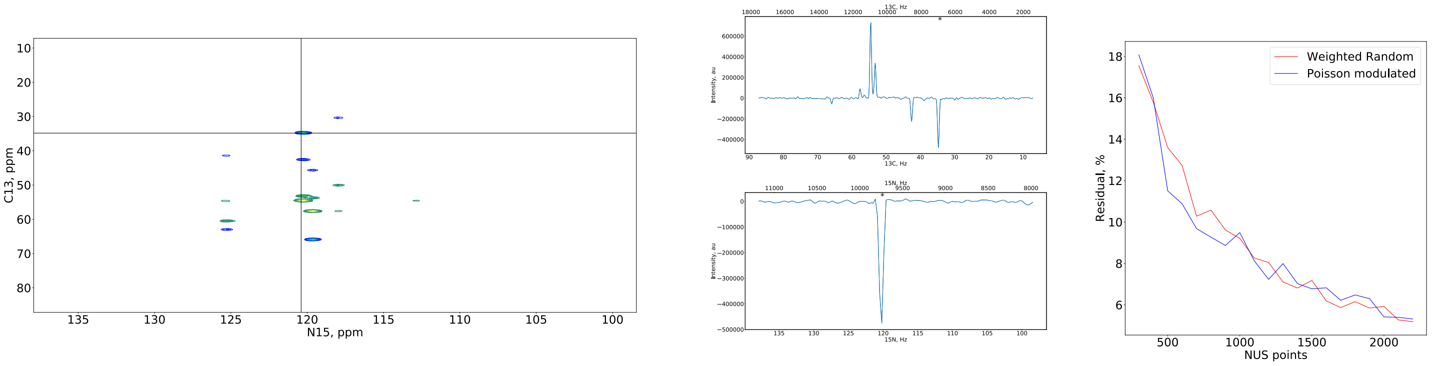

Peak155

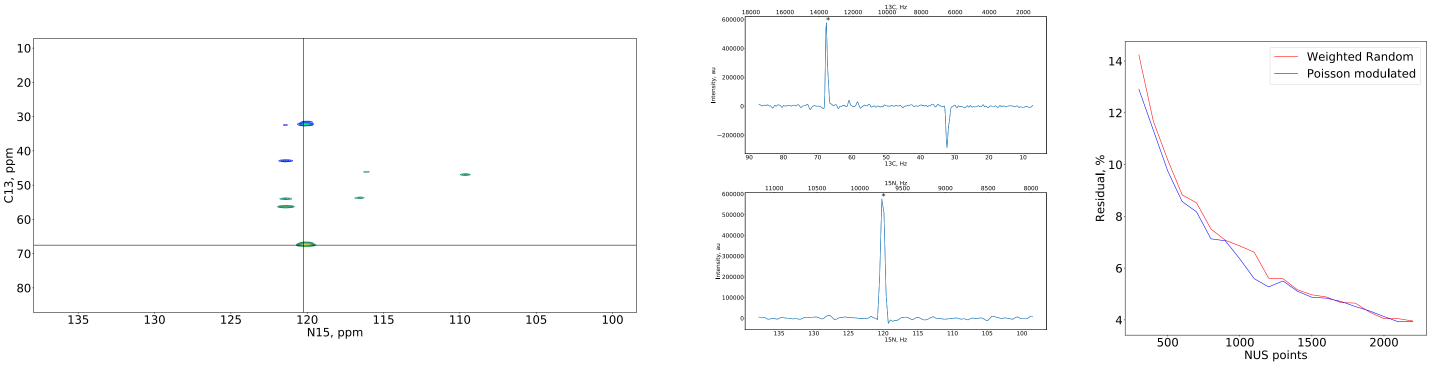

Peak156

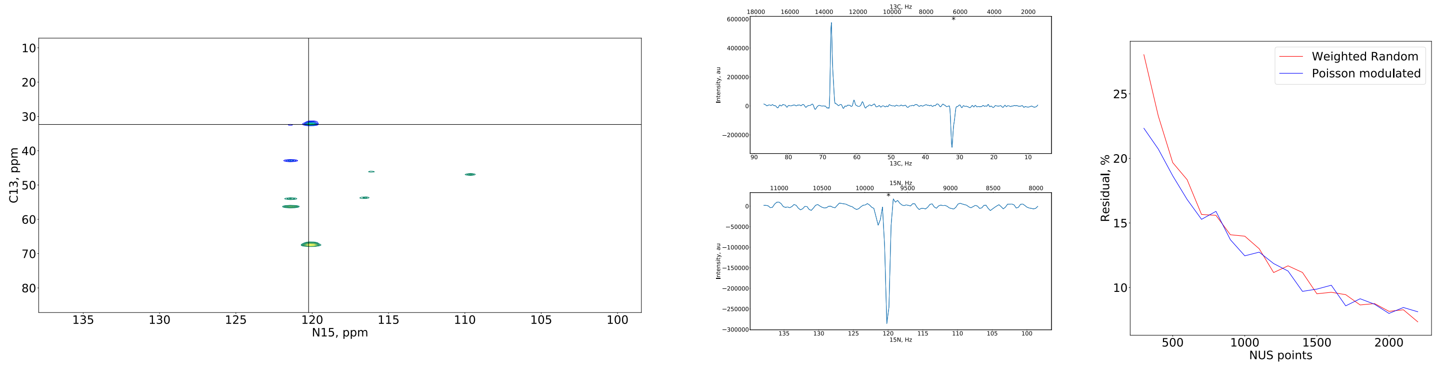

Peak157

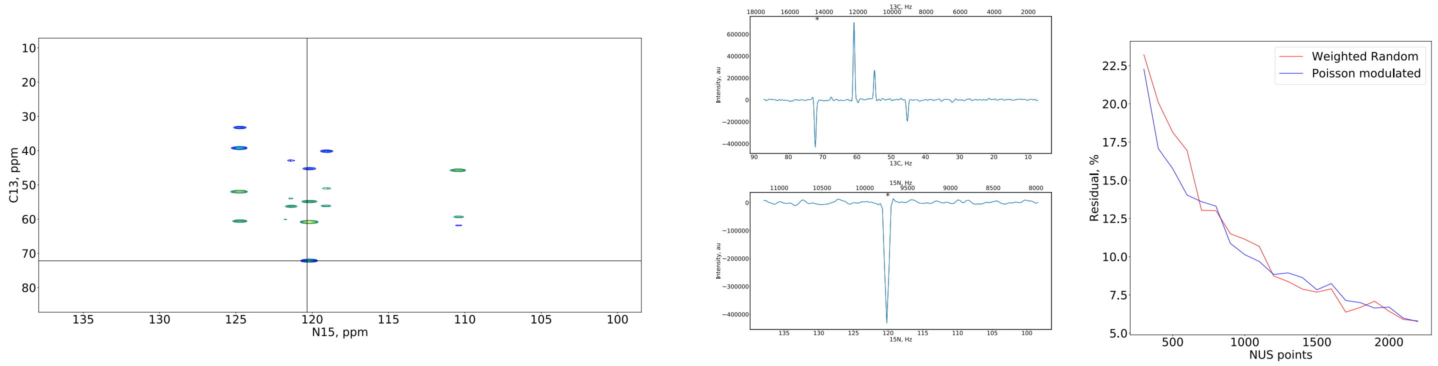

Peak158

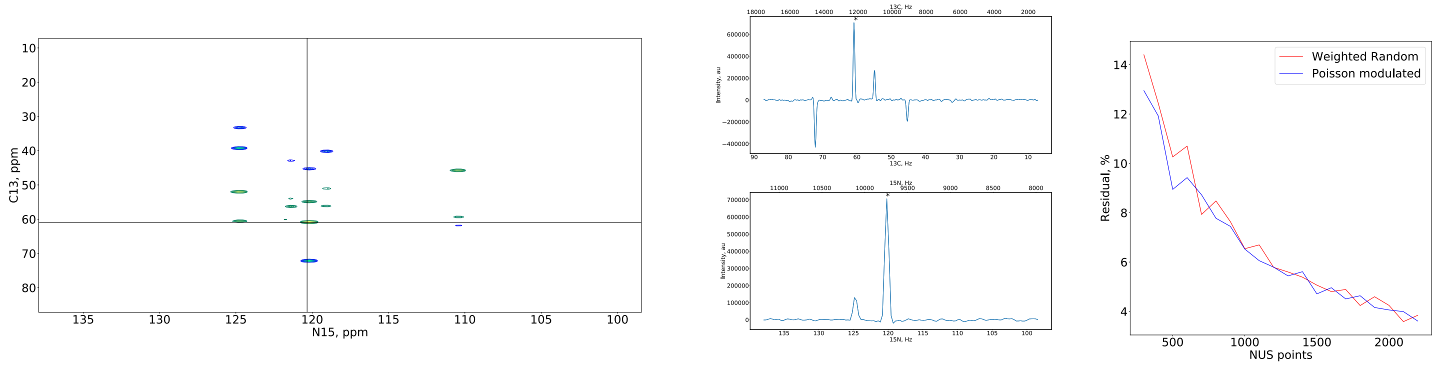

Peak159

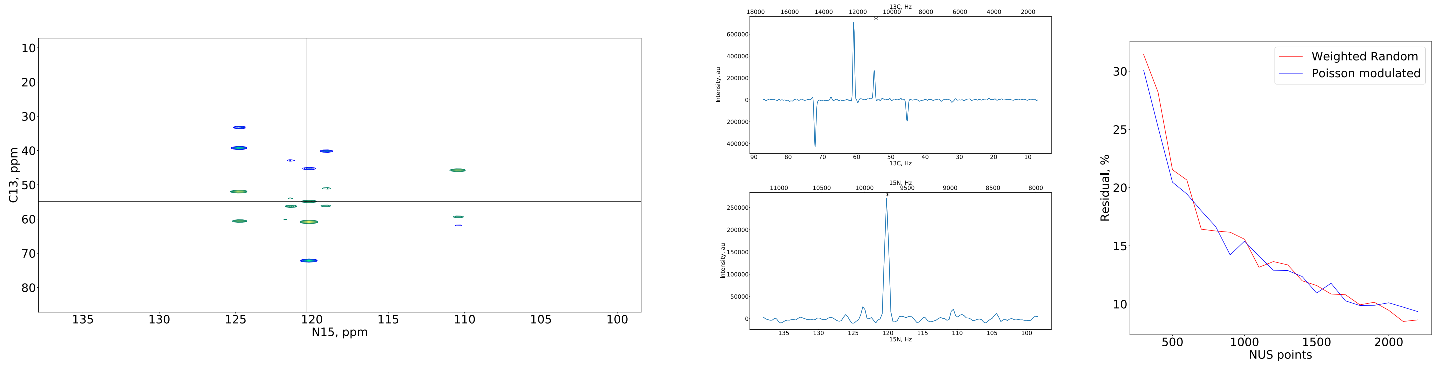

Peak160

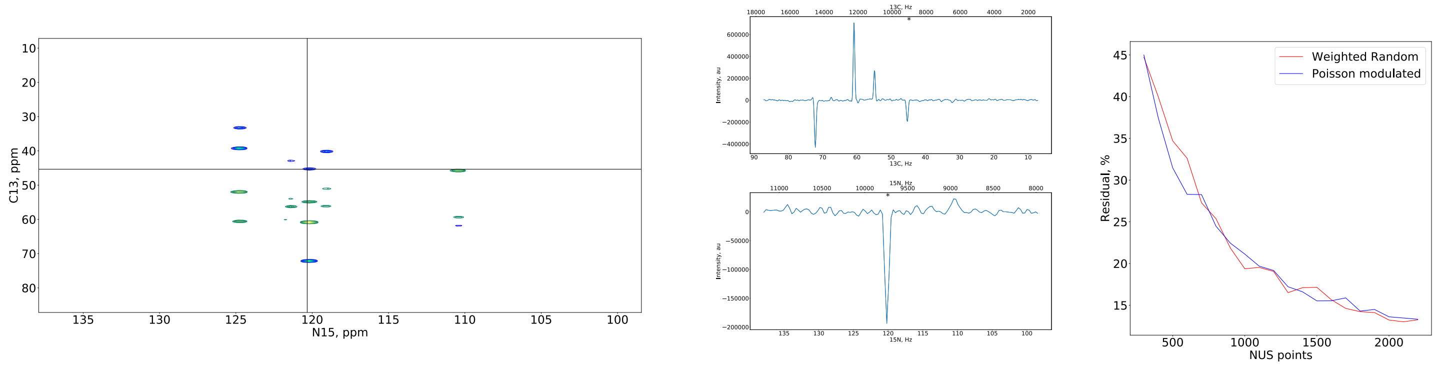

Peak161

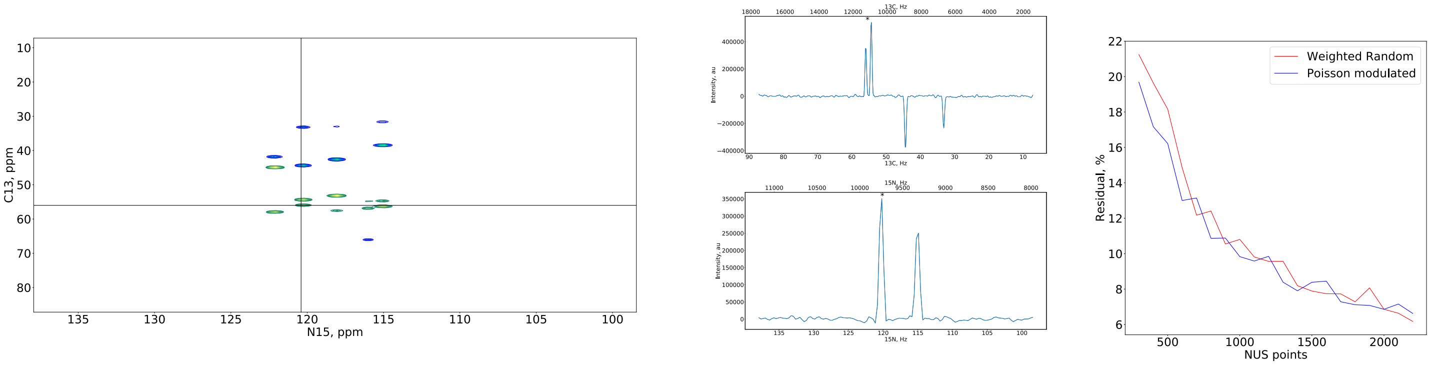

Peak162

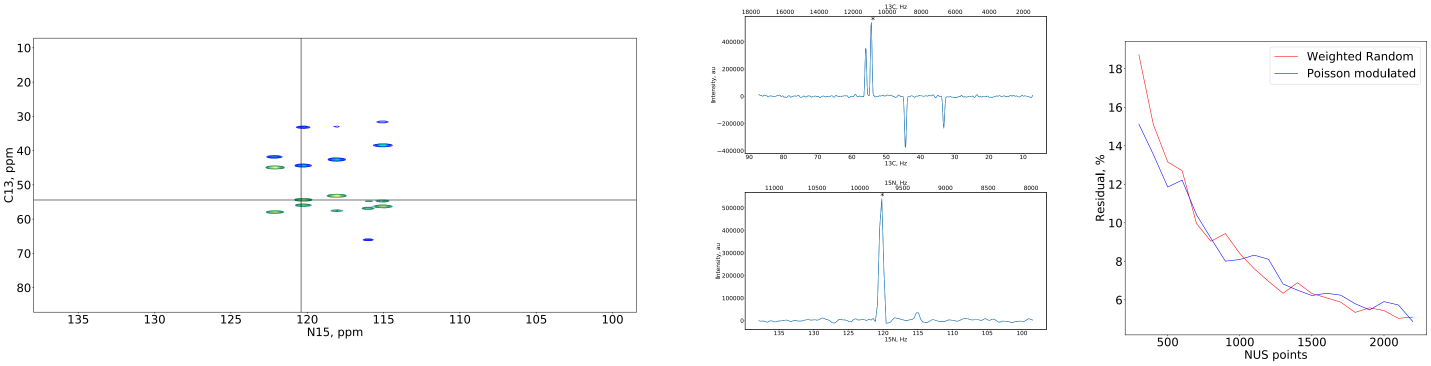

Peak163

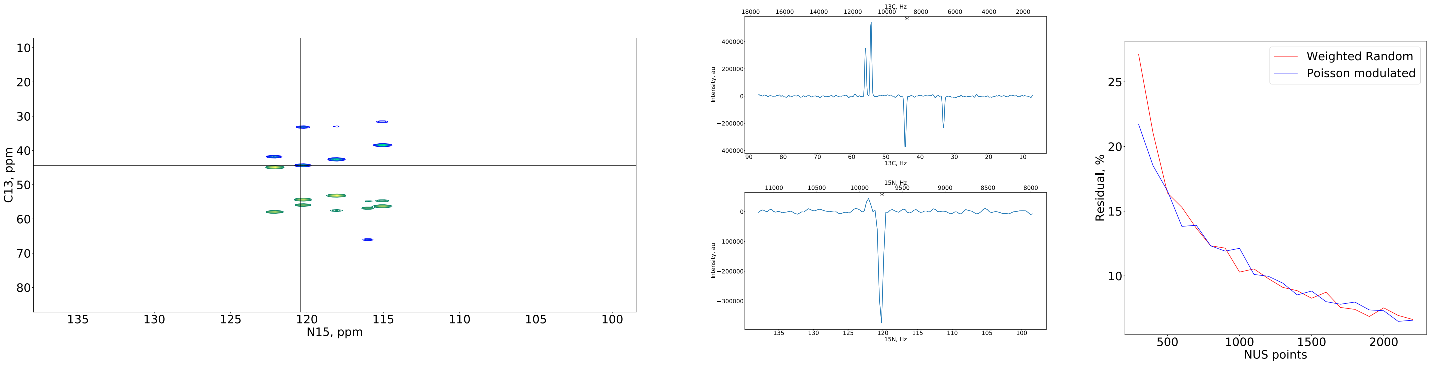

Peak164

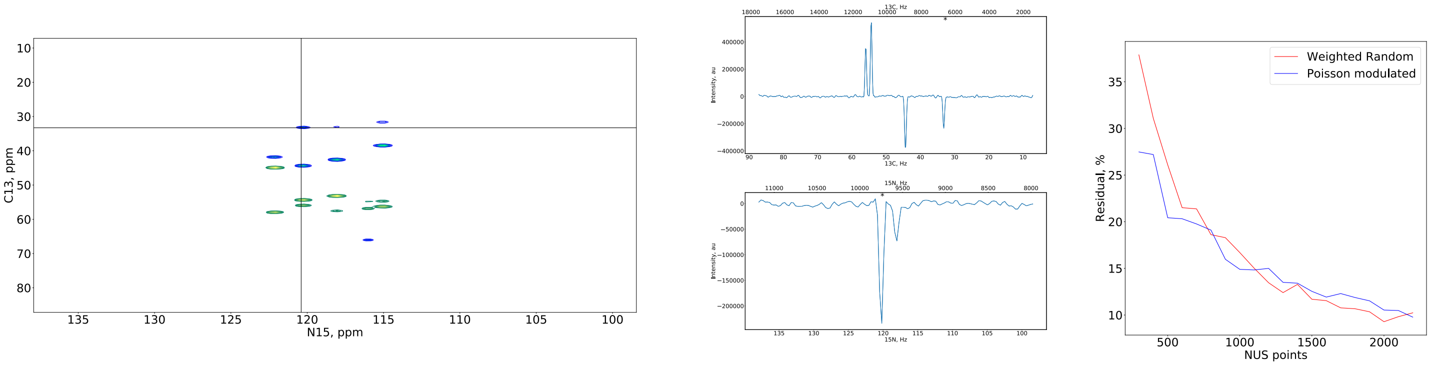

Peak165

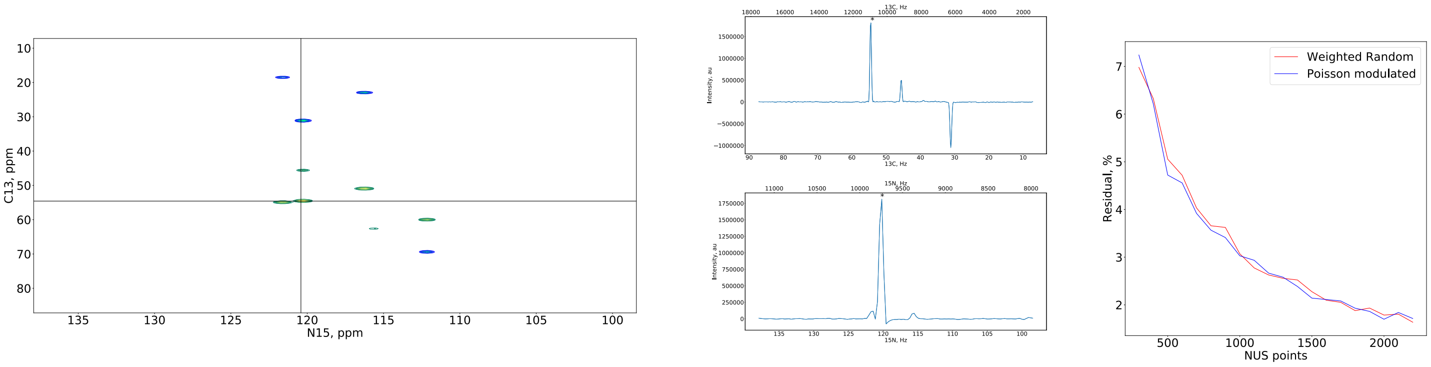

Peak166

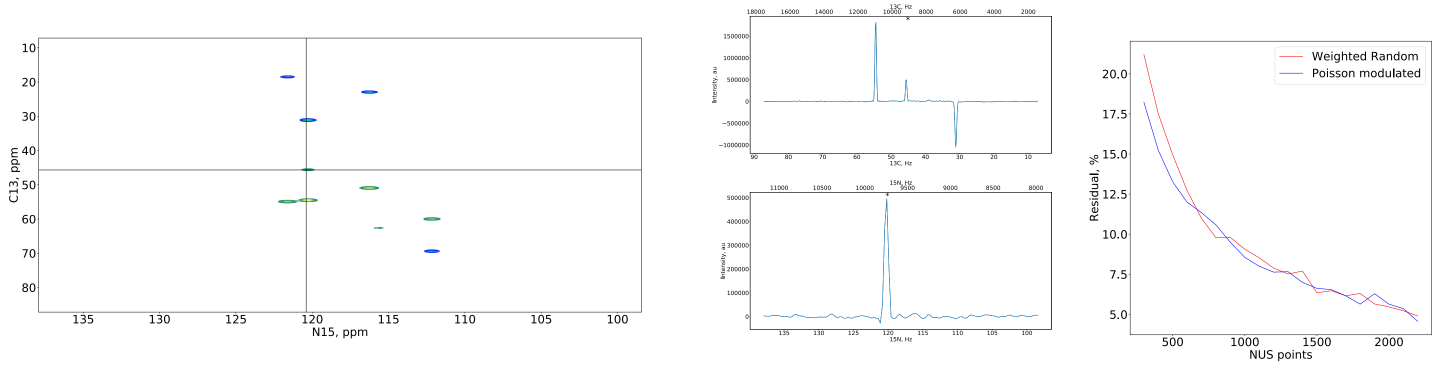

Peak167

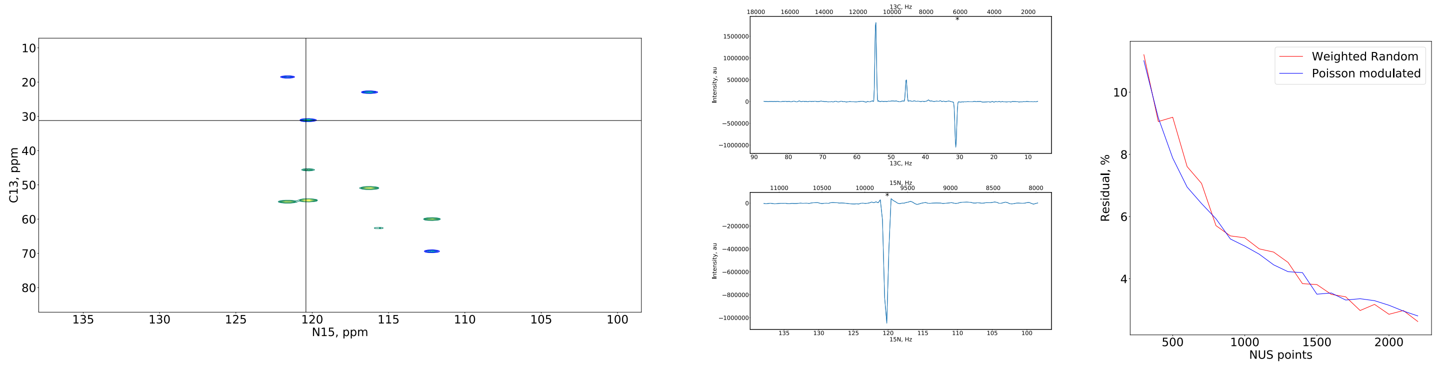

Peak168

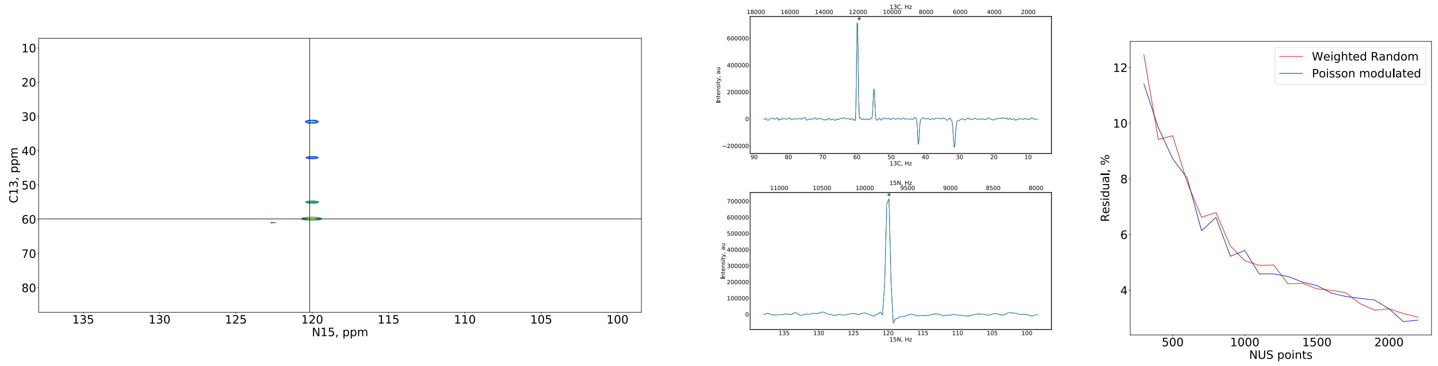

Peak169

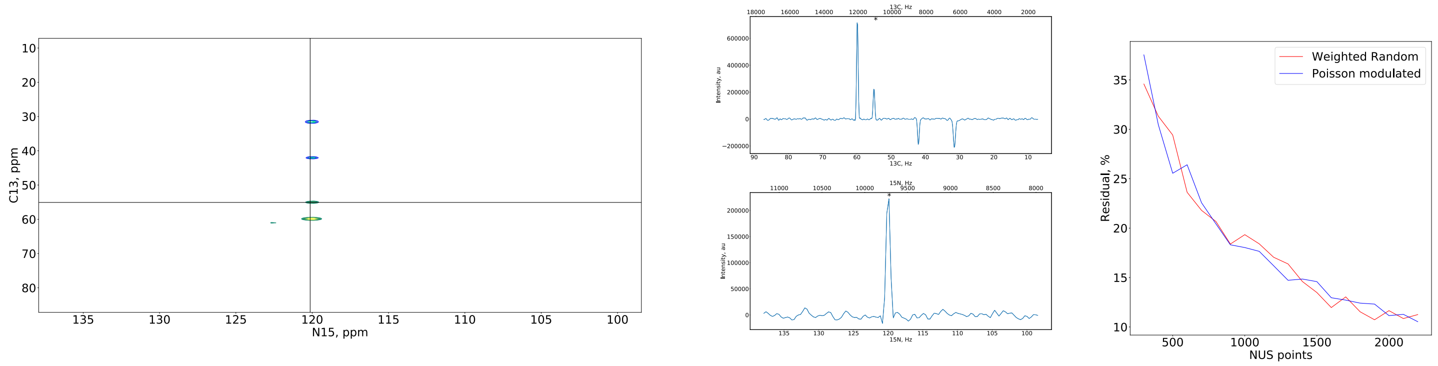

Peak170

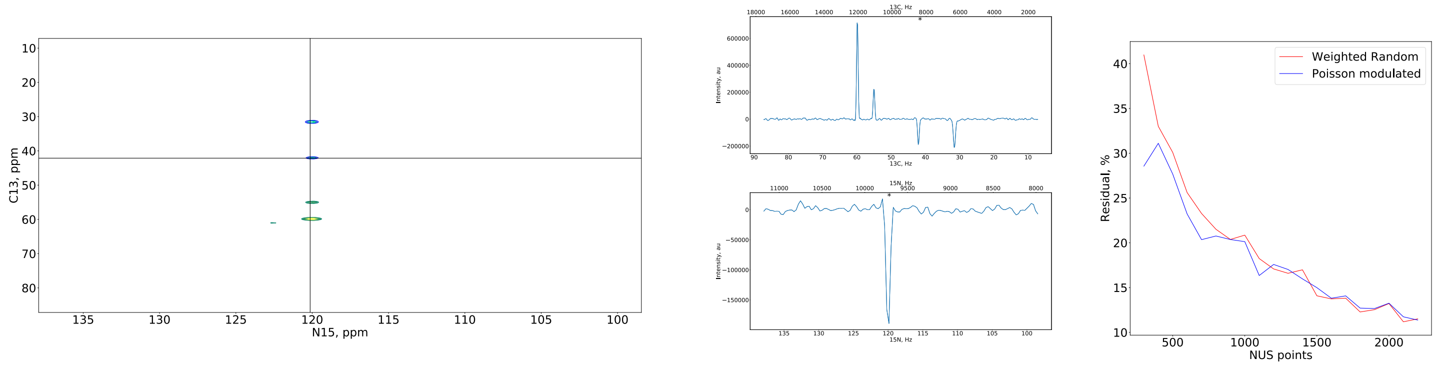

Peak171

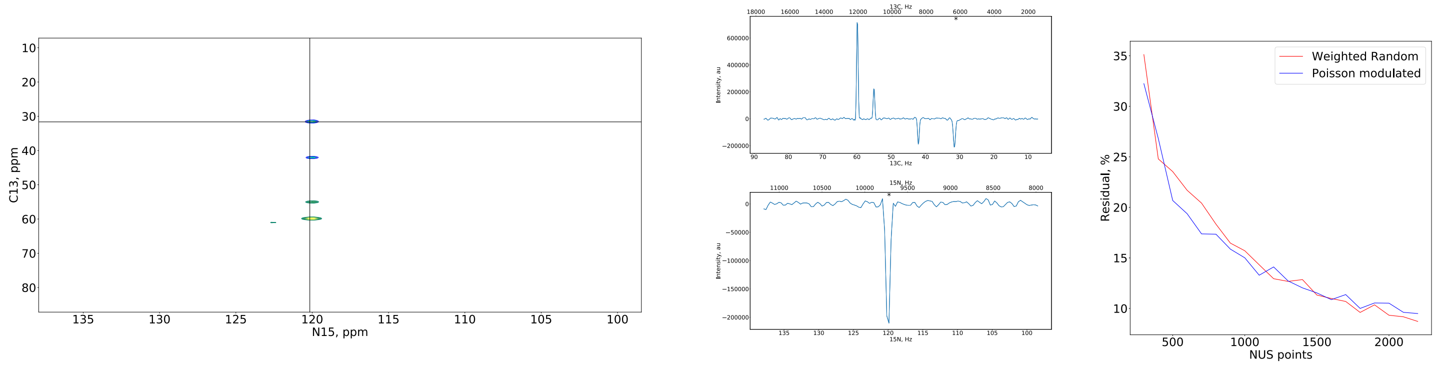

Peak172

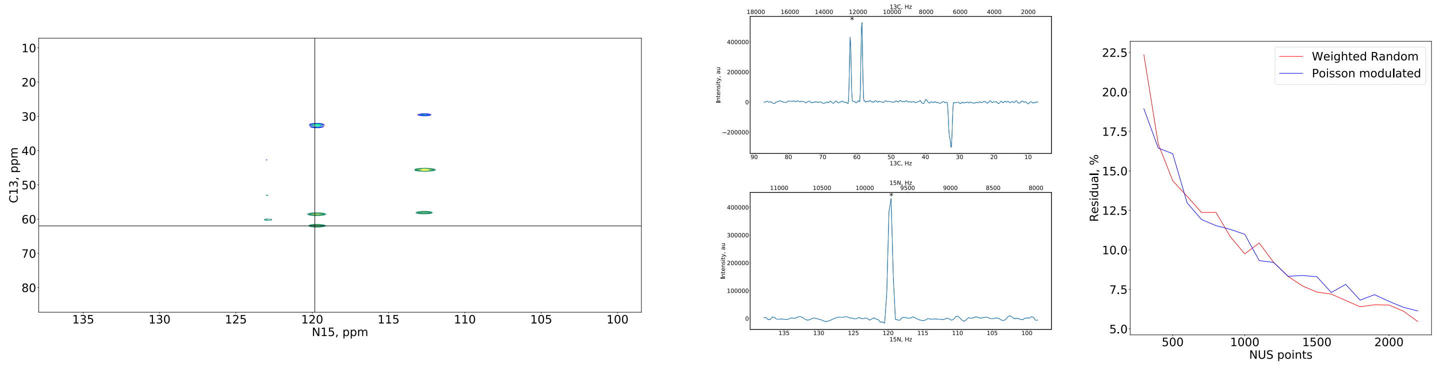

Peak173

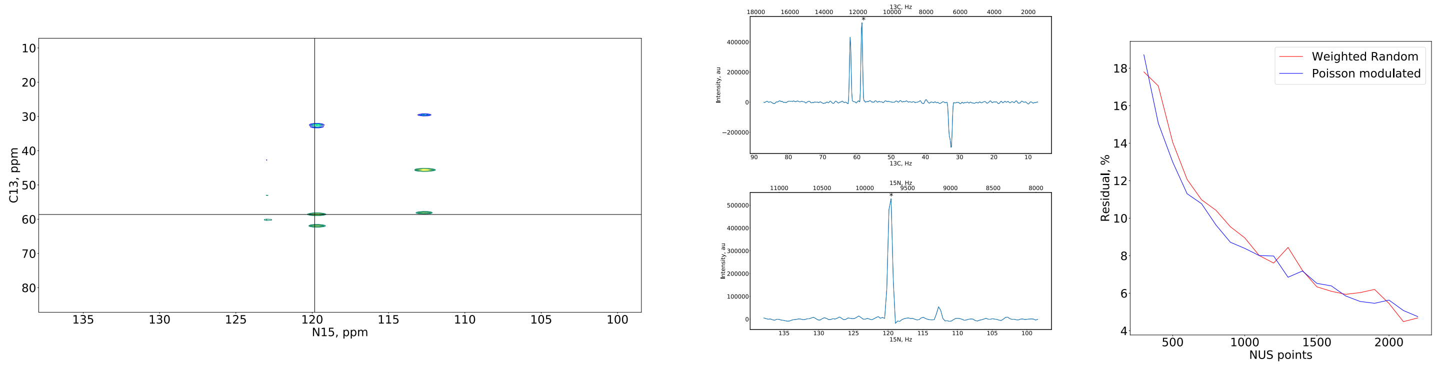

Peak174

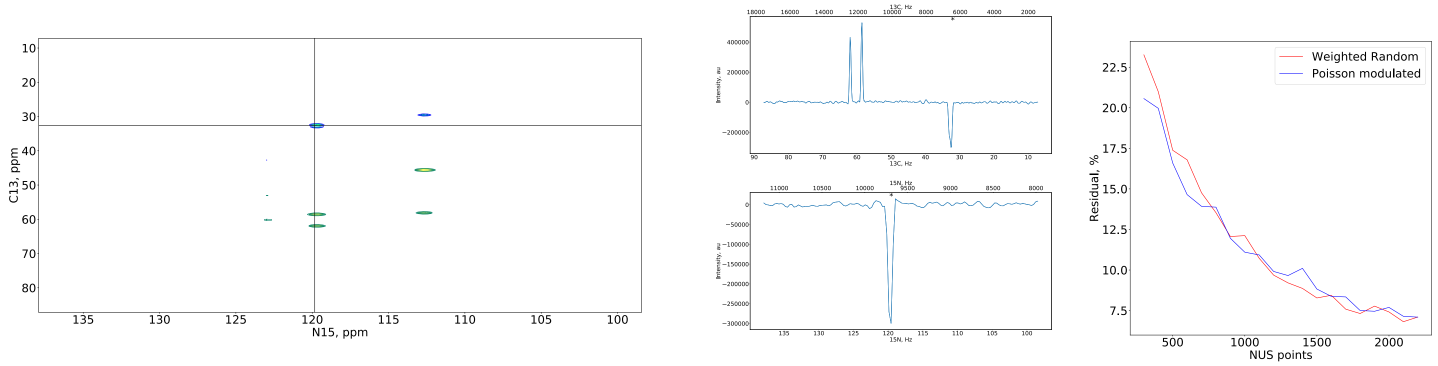

Peak175

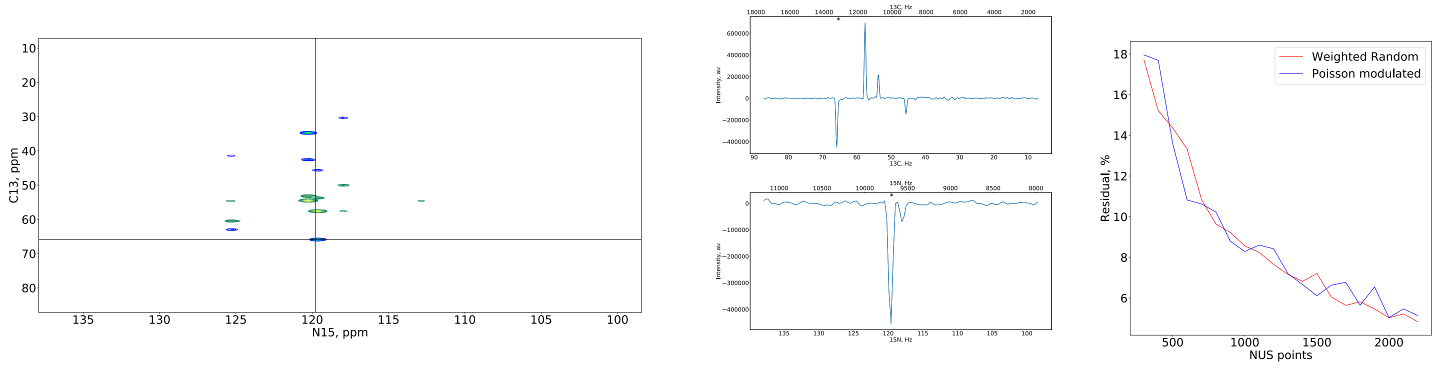

Peak176

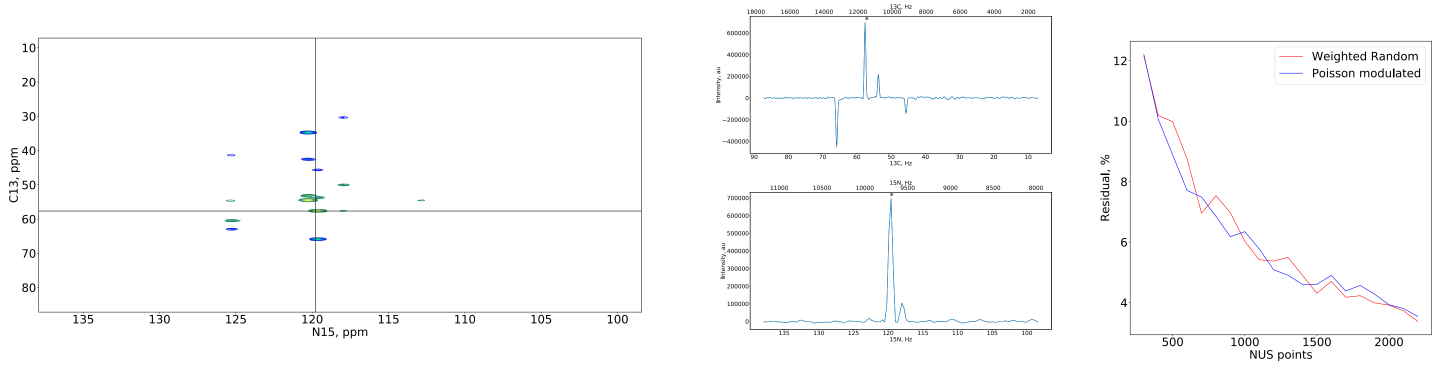

Peak177

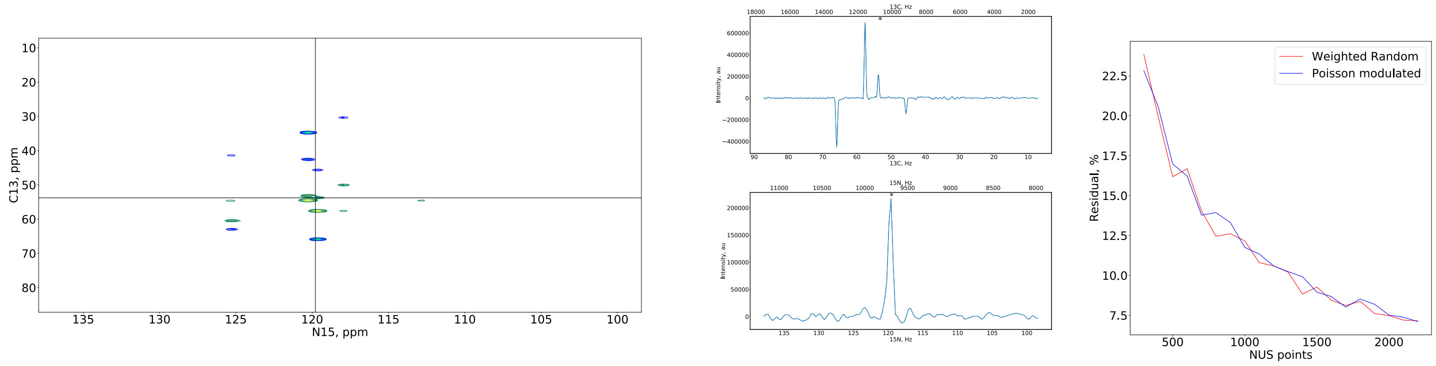

Peak178

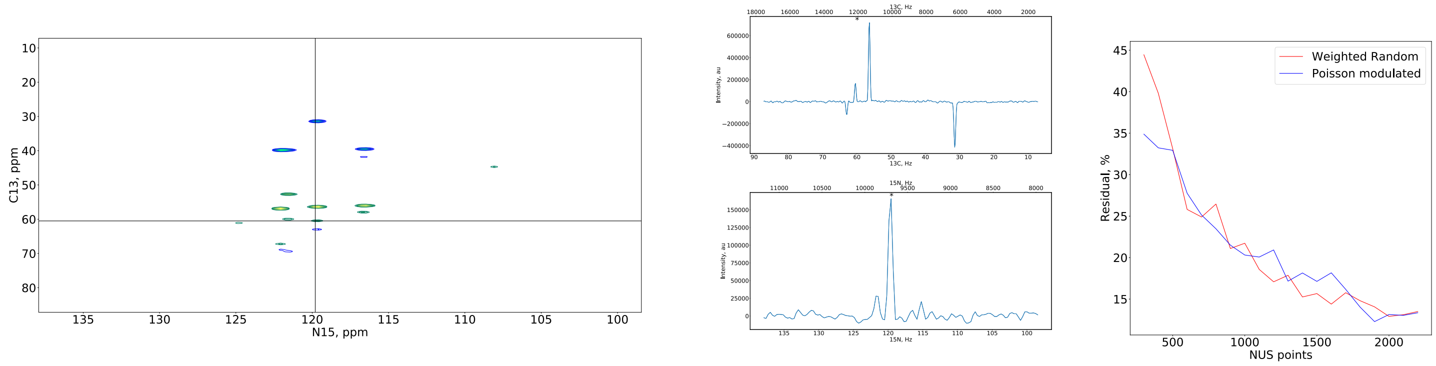

Peak179

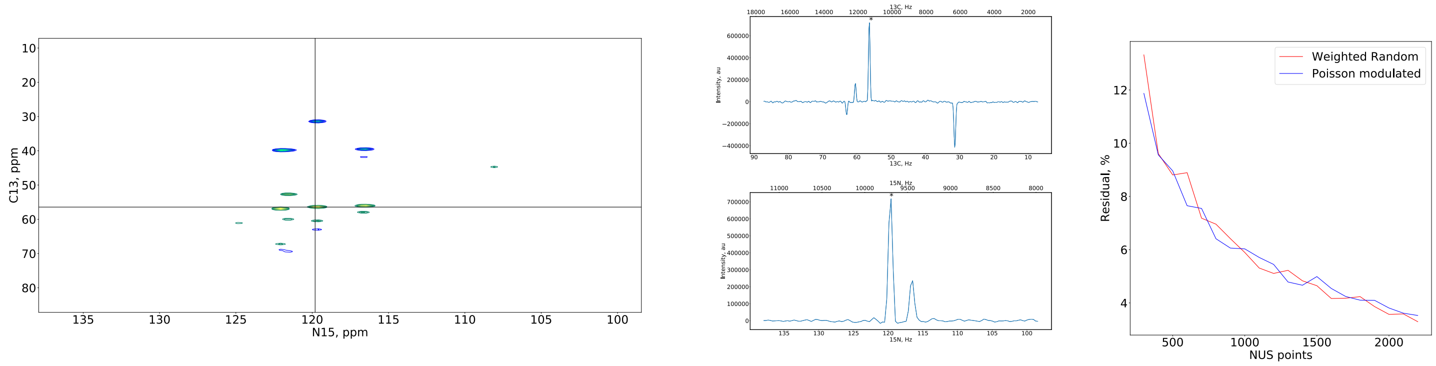

Peak180

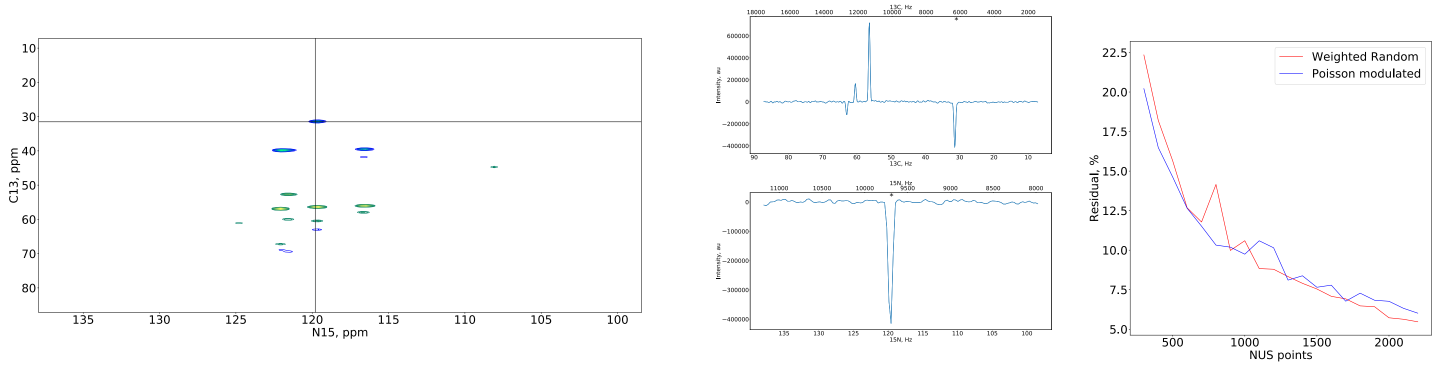

Peak181

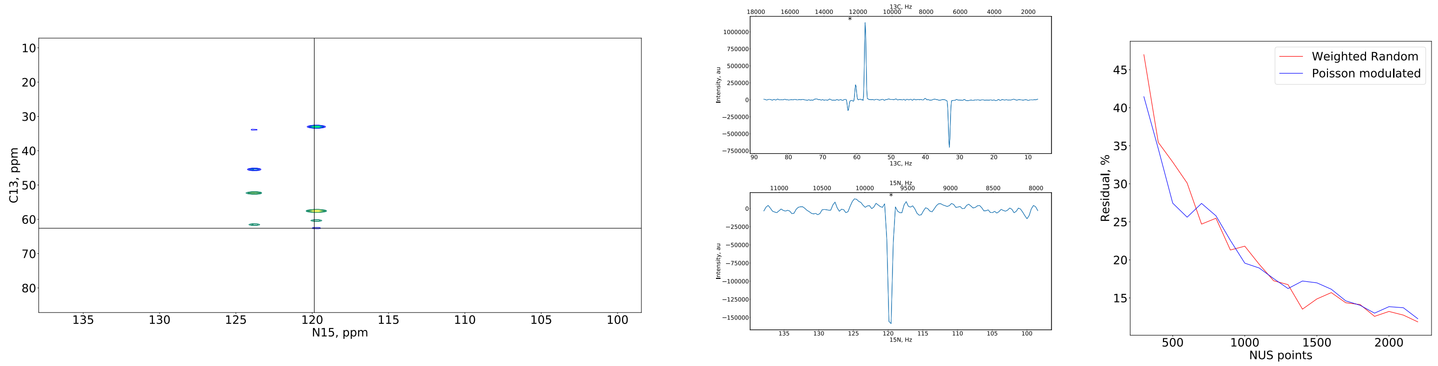

Peak182

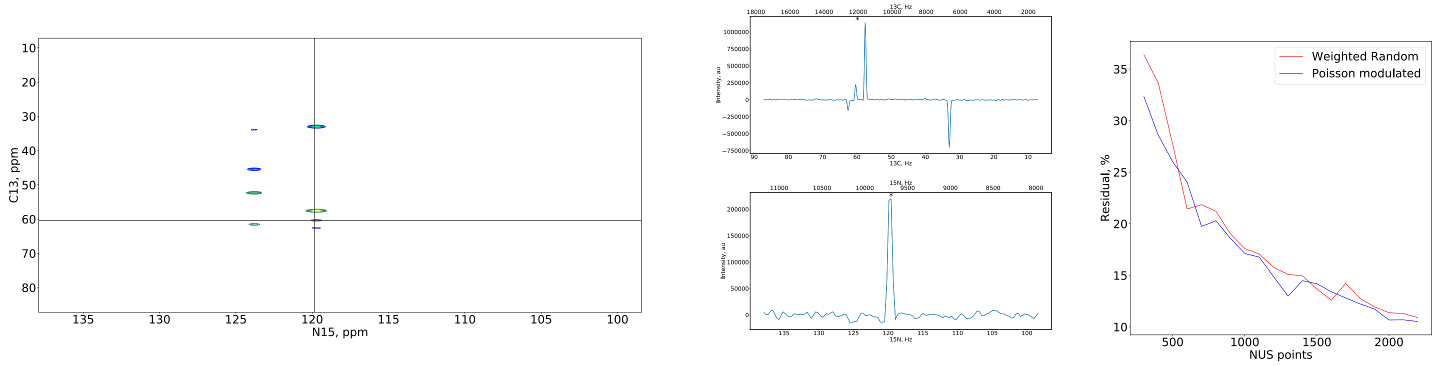

Peak183

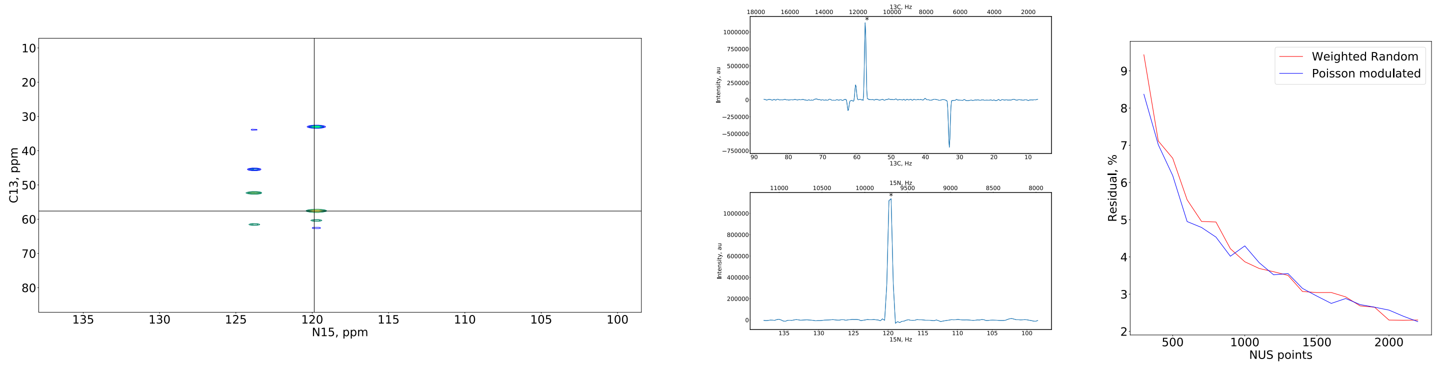

Peak184

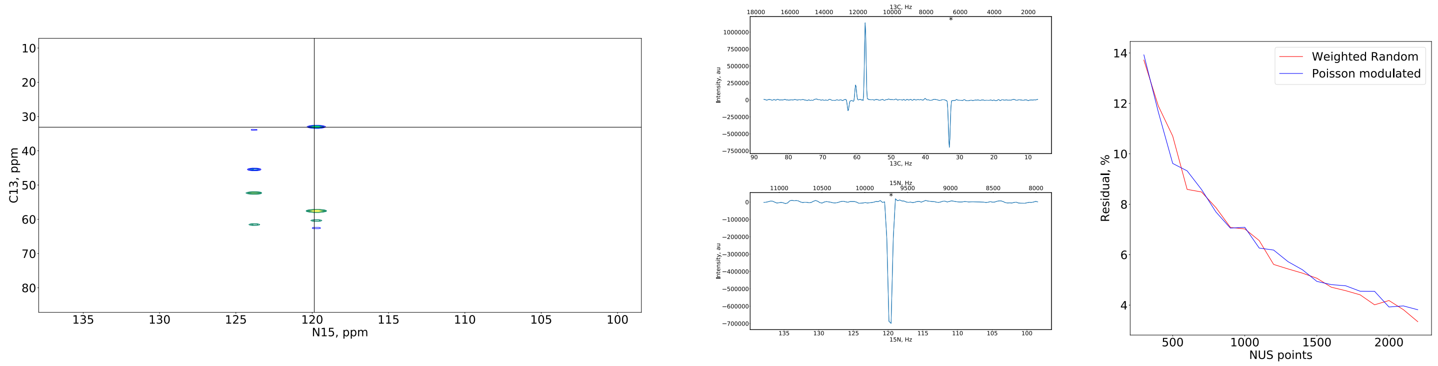

Peak185

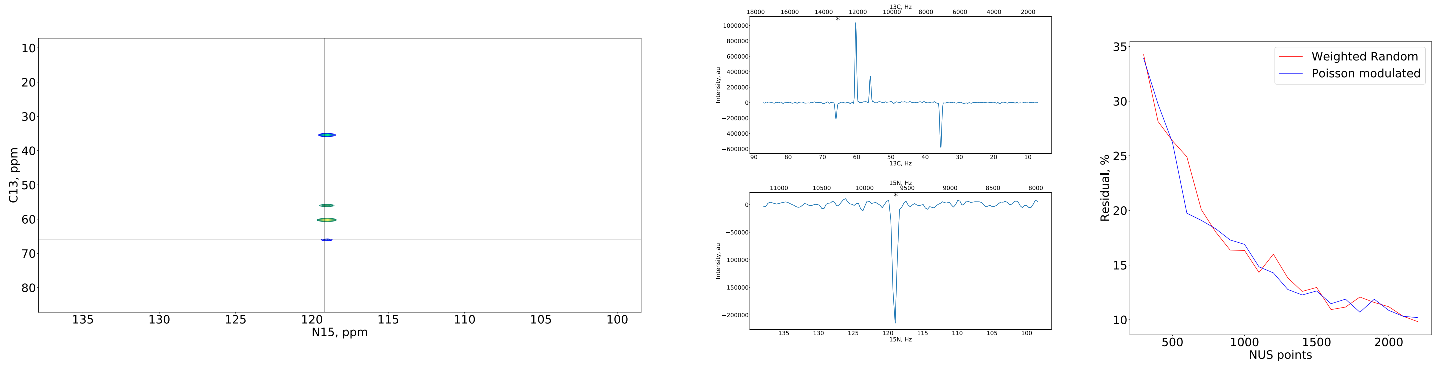

Peak186

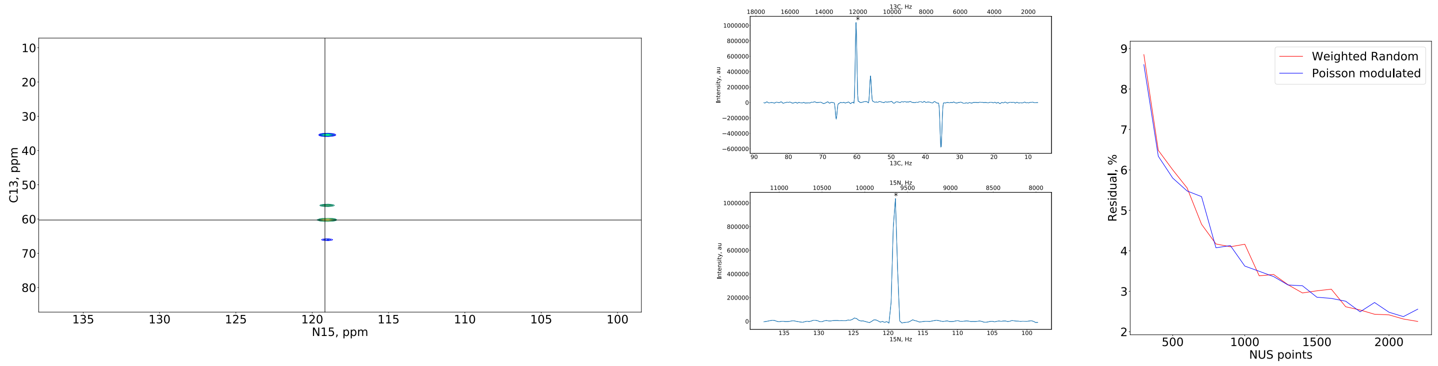

Peak187

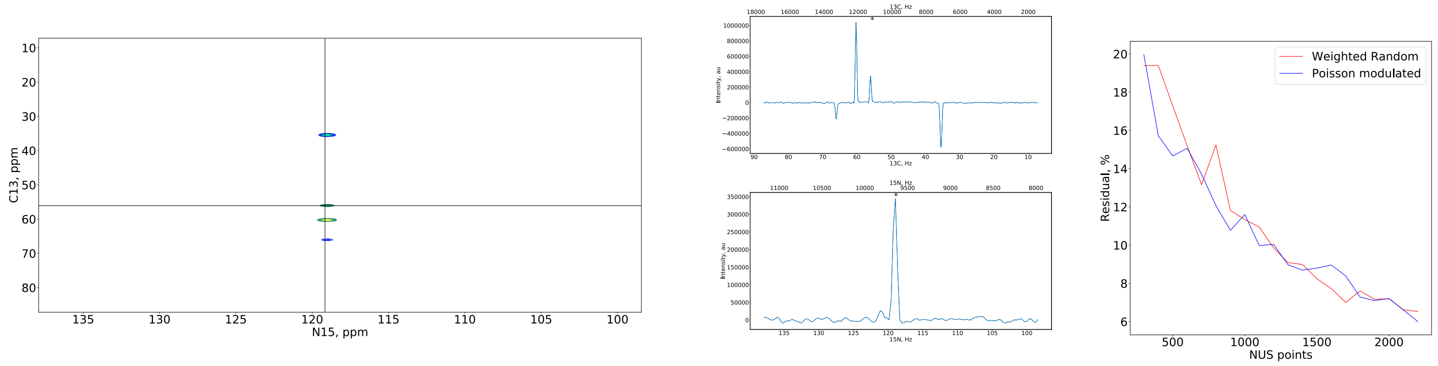

Peak188

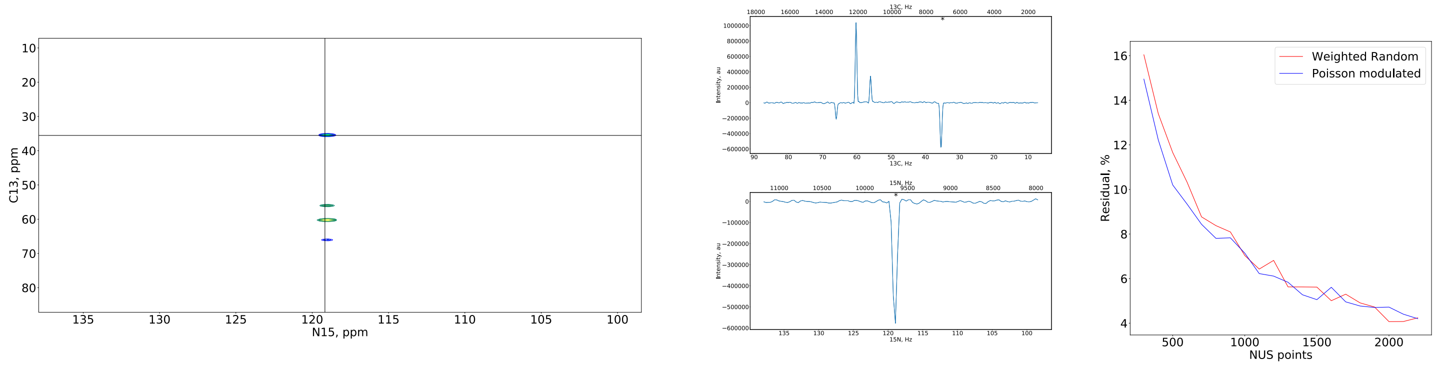

Peak189

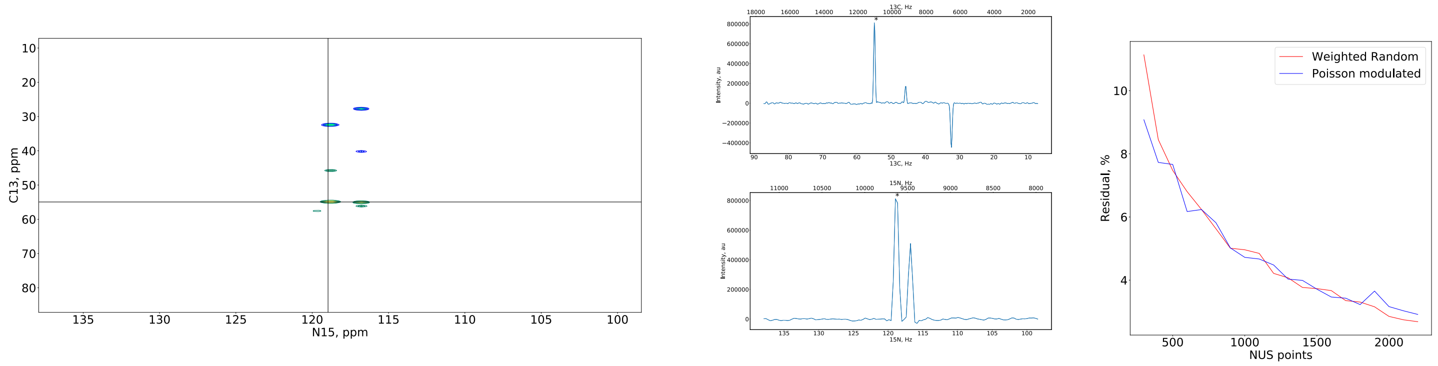

Peak190

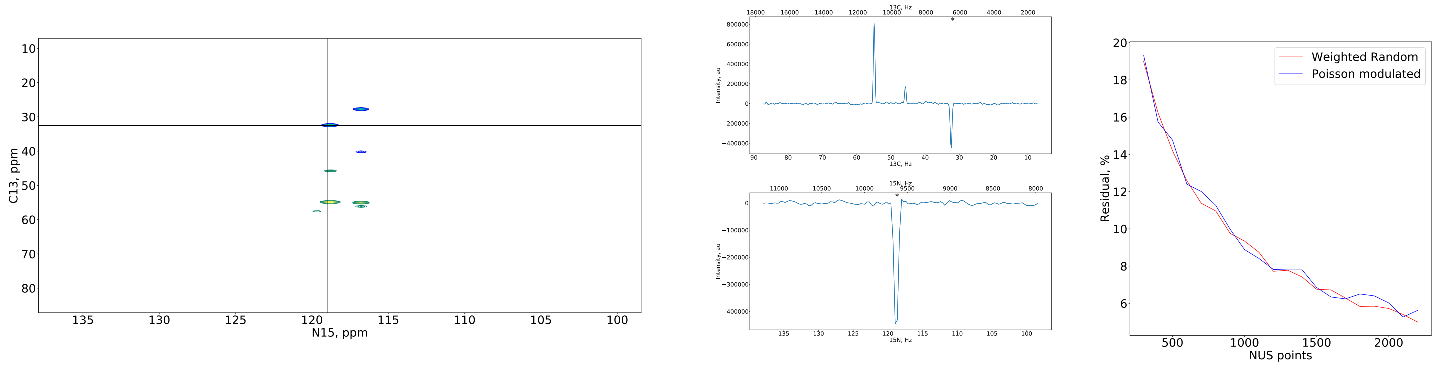

Peak191

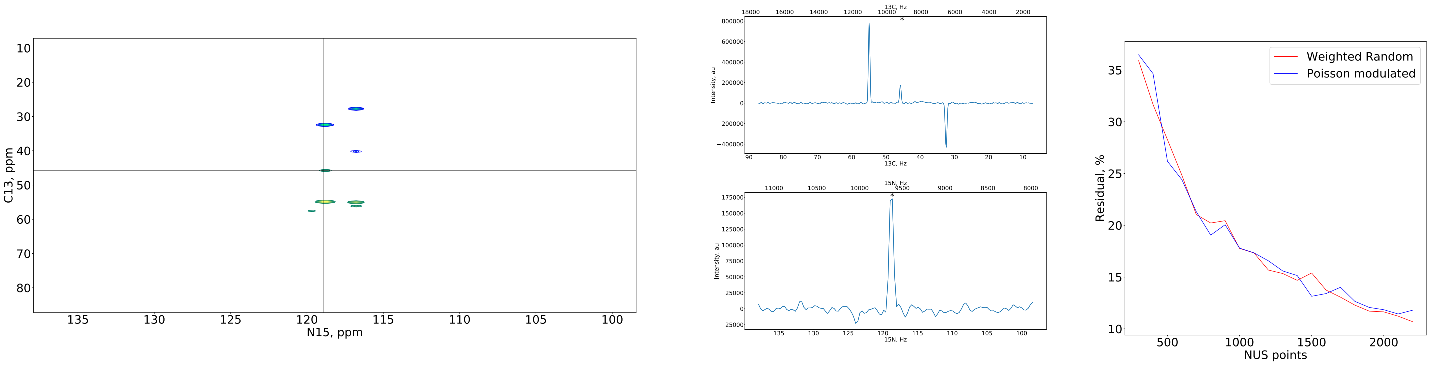

Peak192

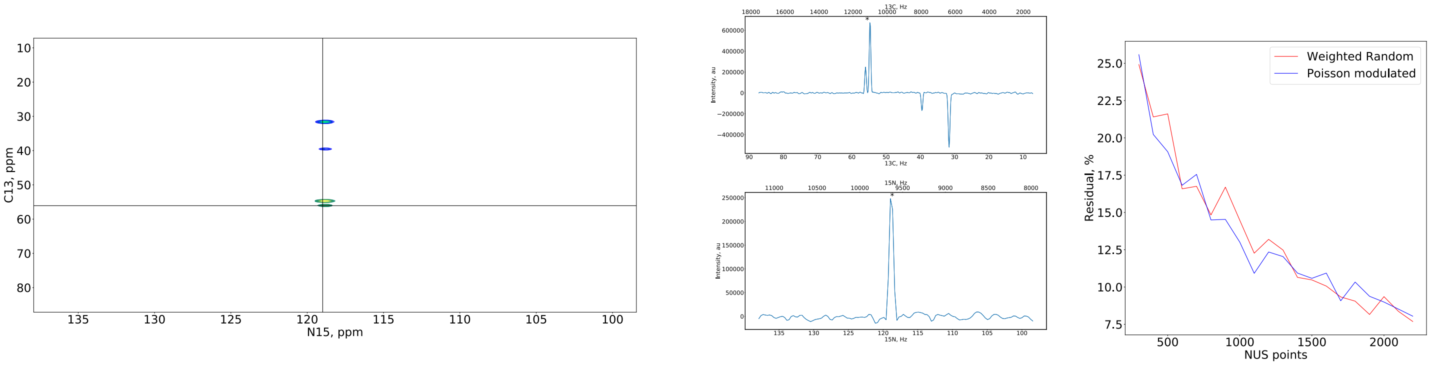

Peak193

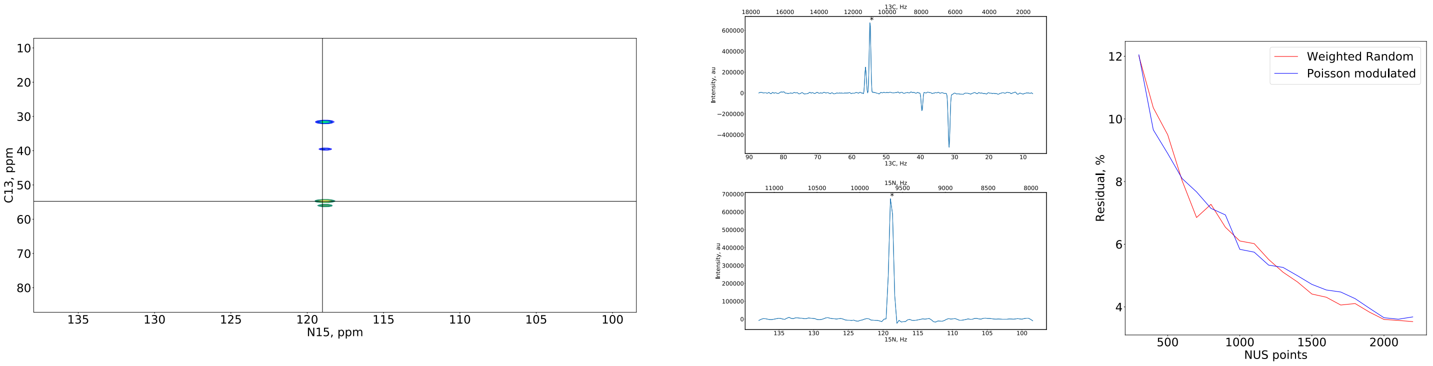

Peak194

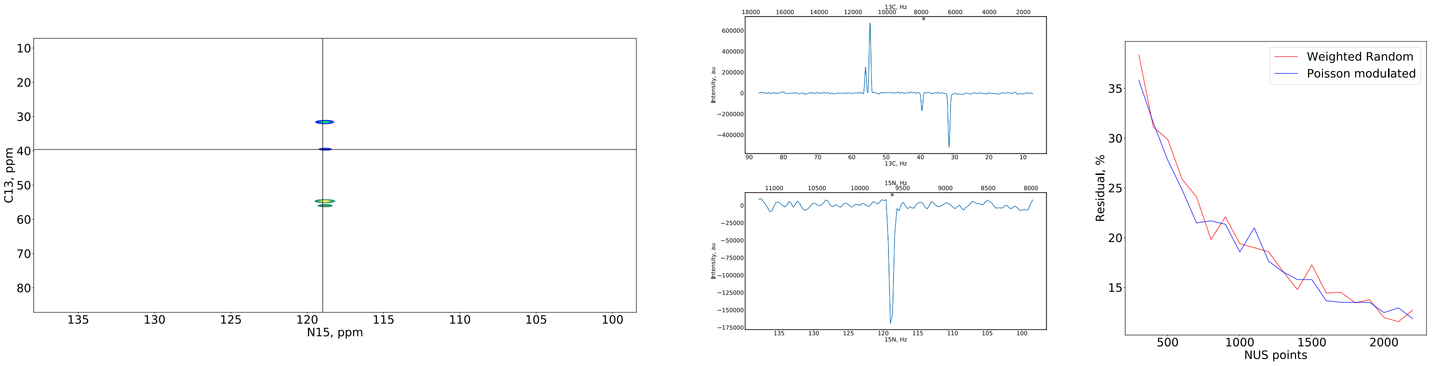

Peak195

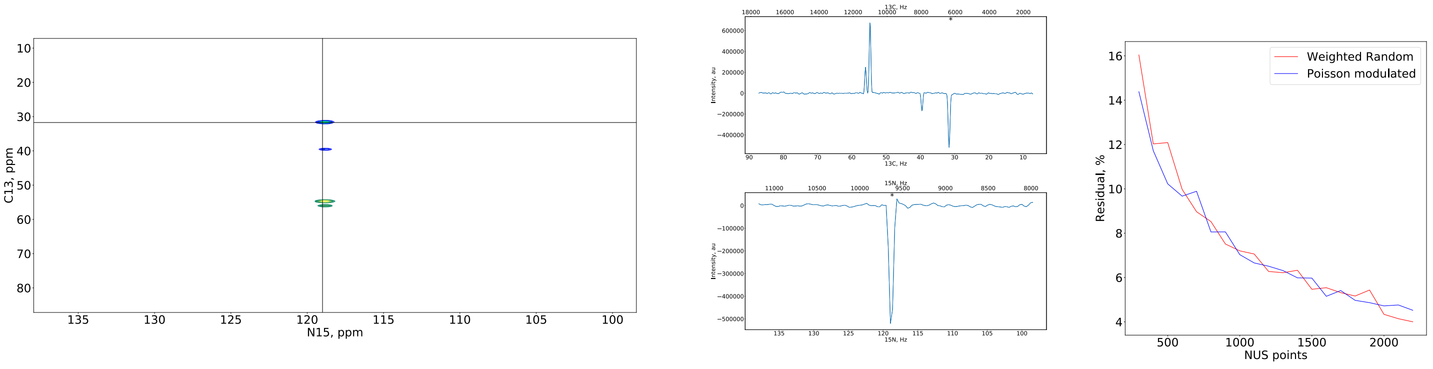

Peak196

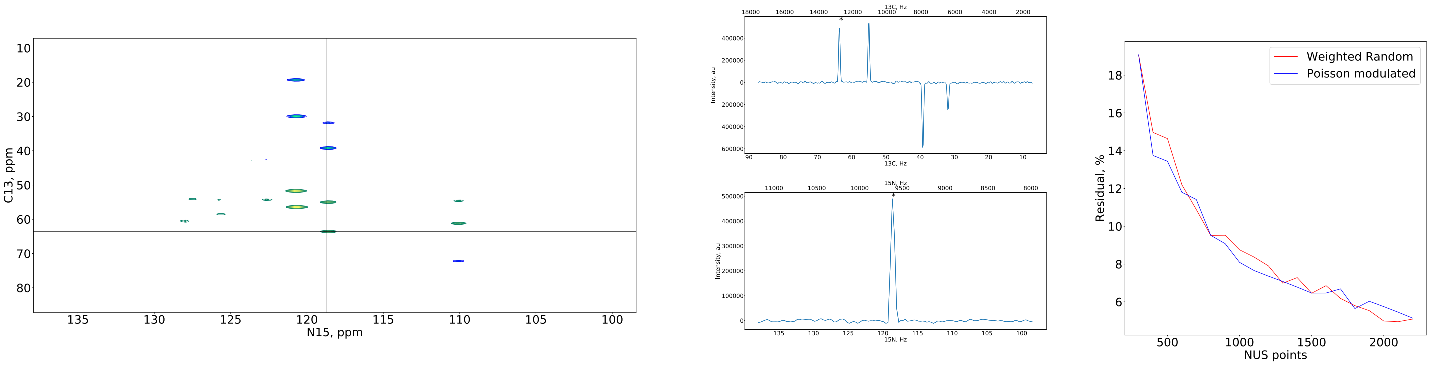

Peak197

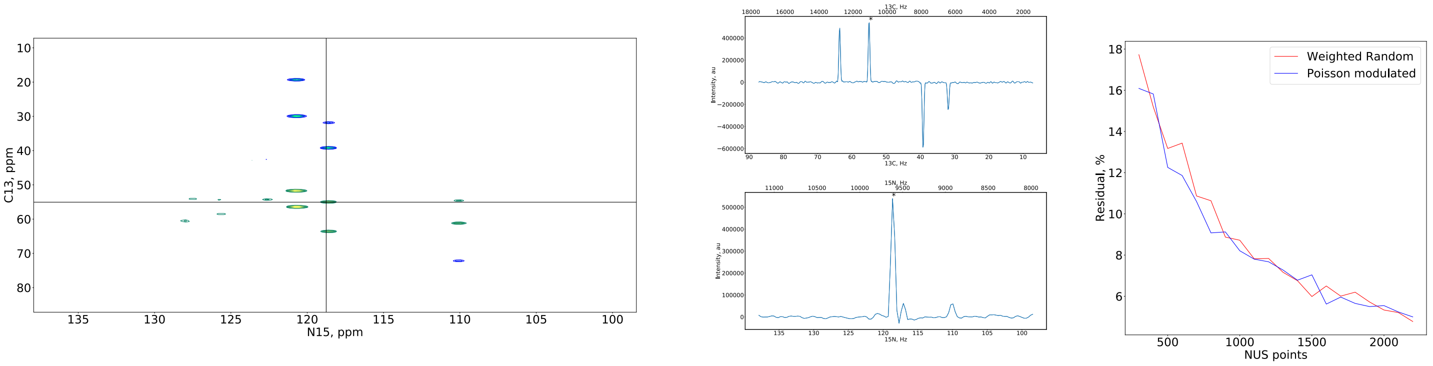

Peak198

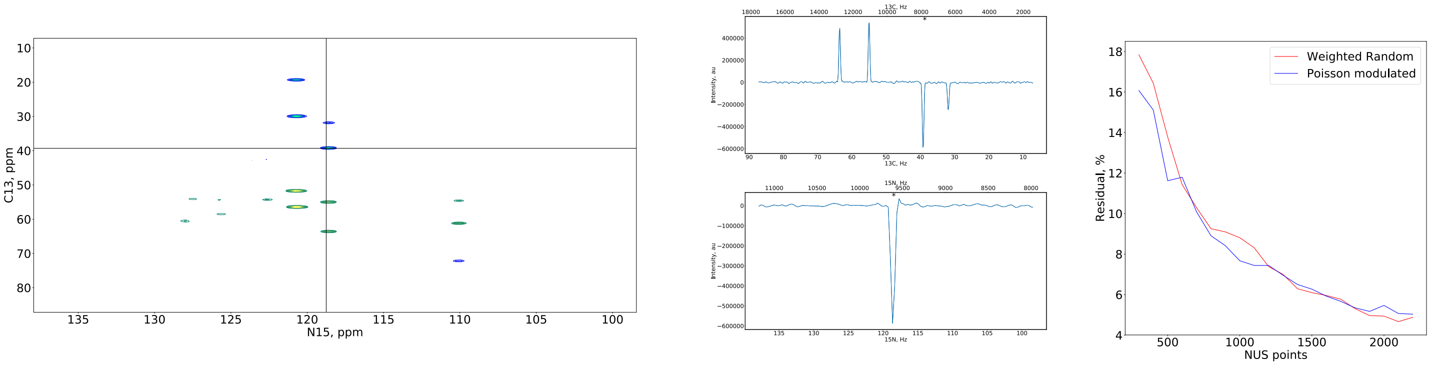

Peak199

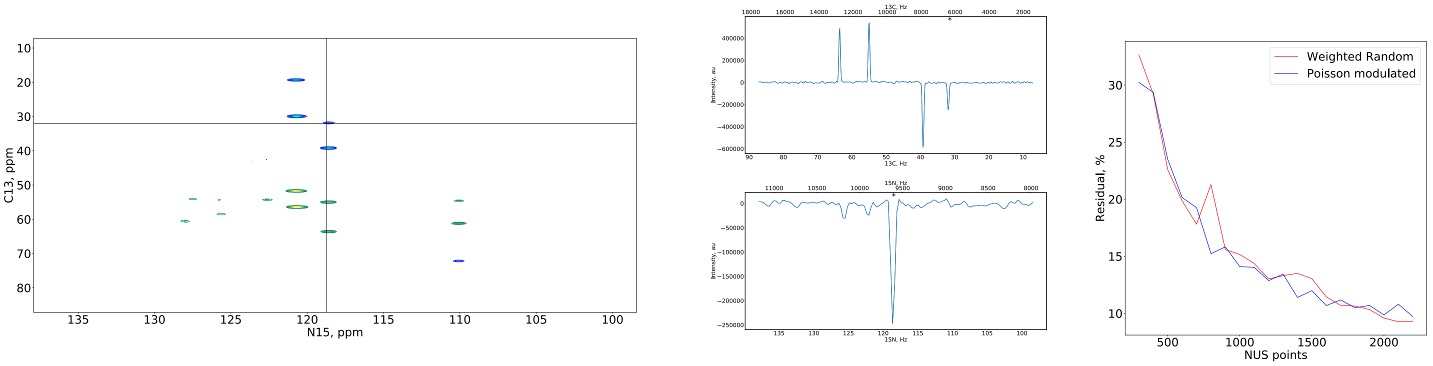

Peak200

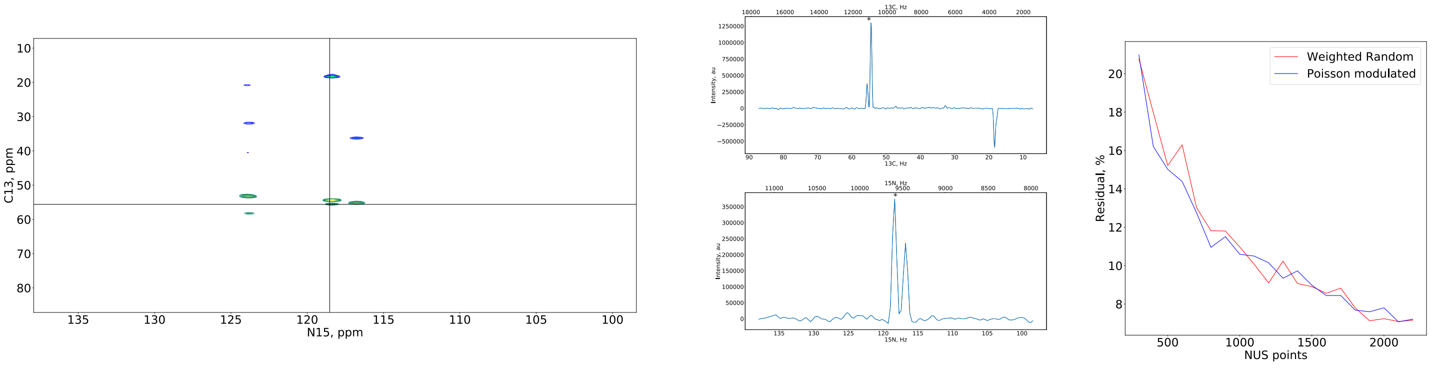

Peak201

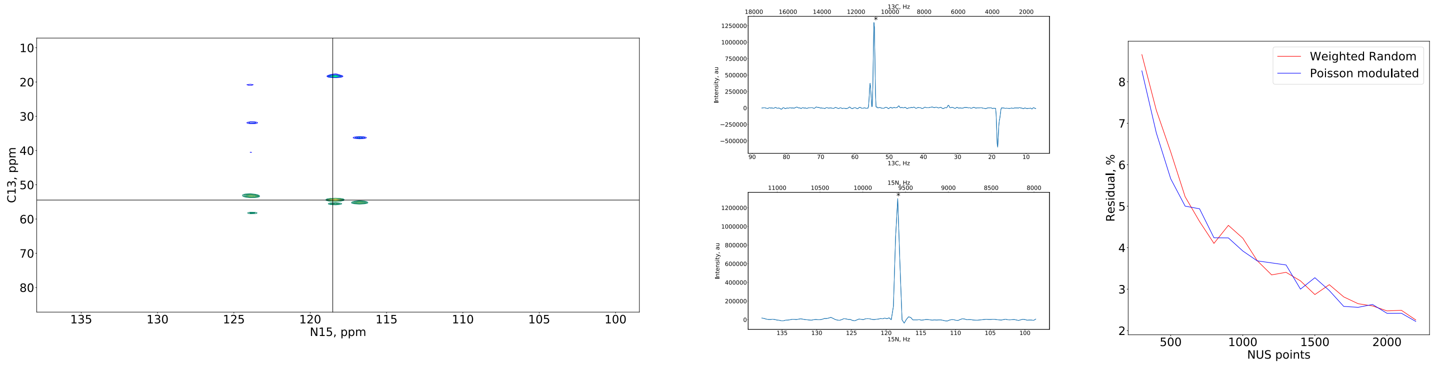

Peak202

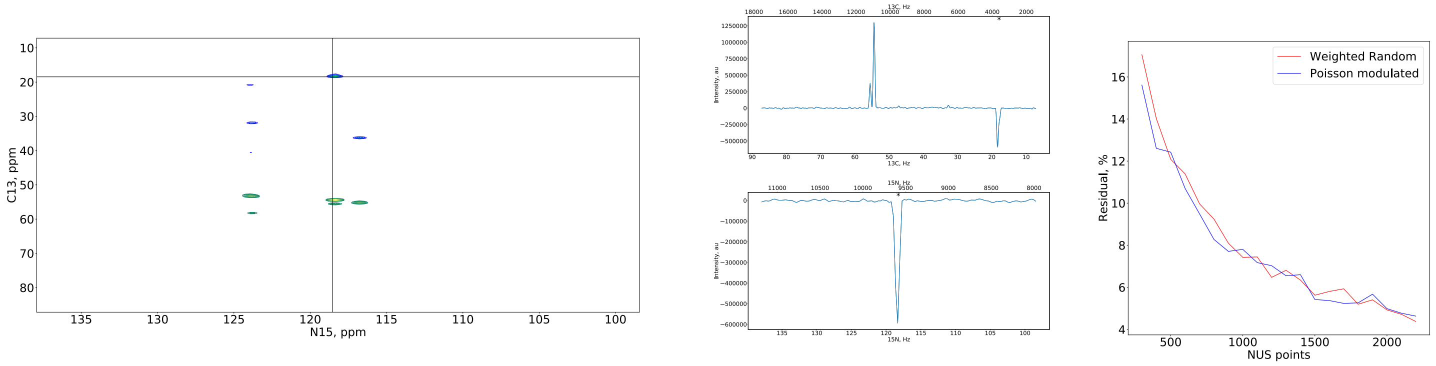

Peak203

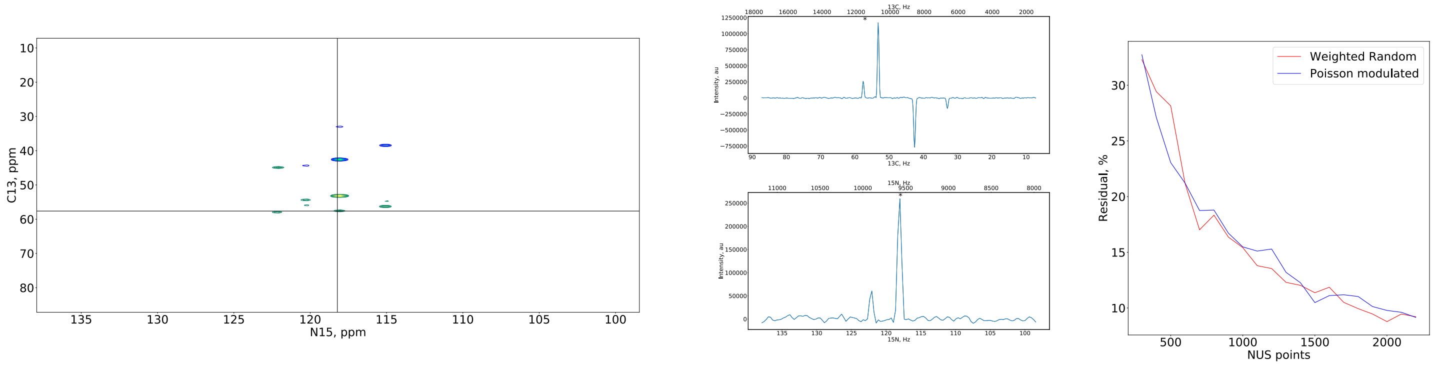

Peak204

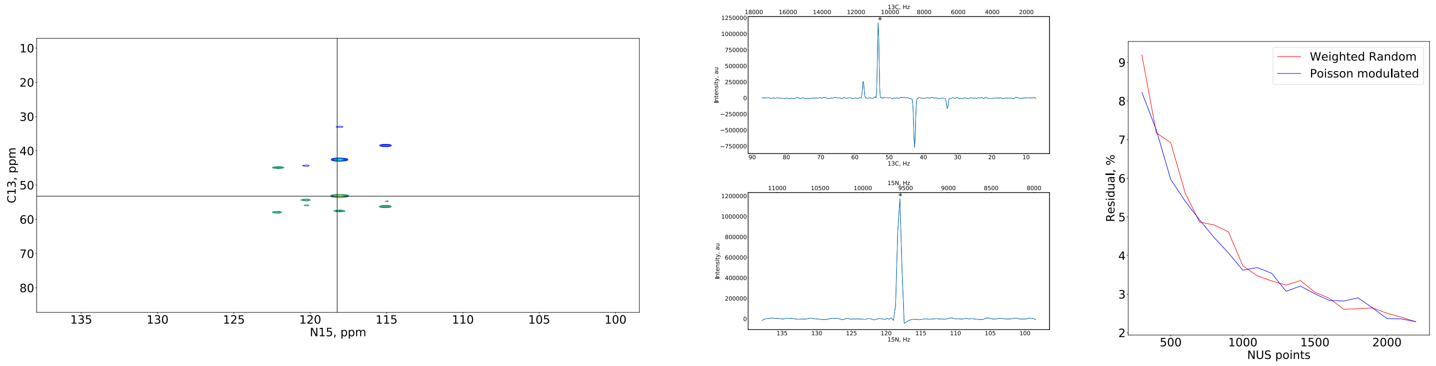

Peak205

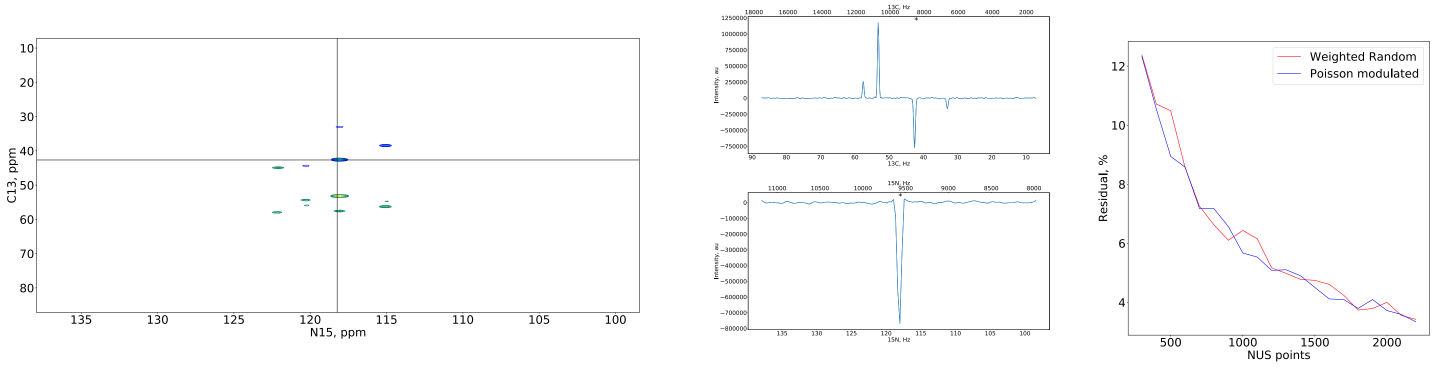

Peak206

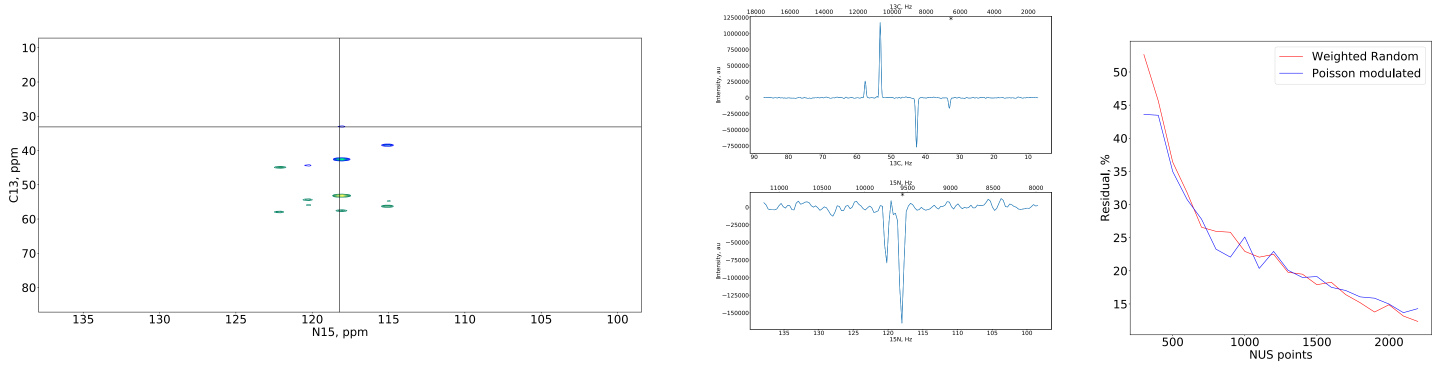

Peak207

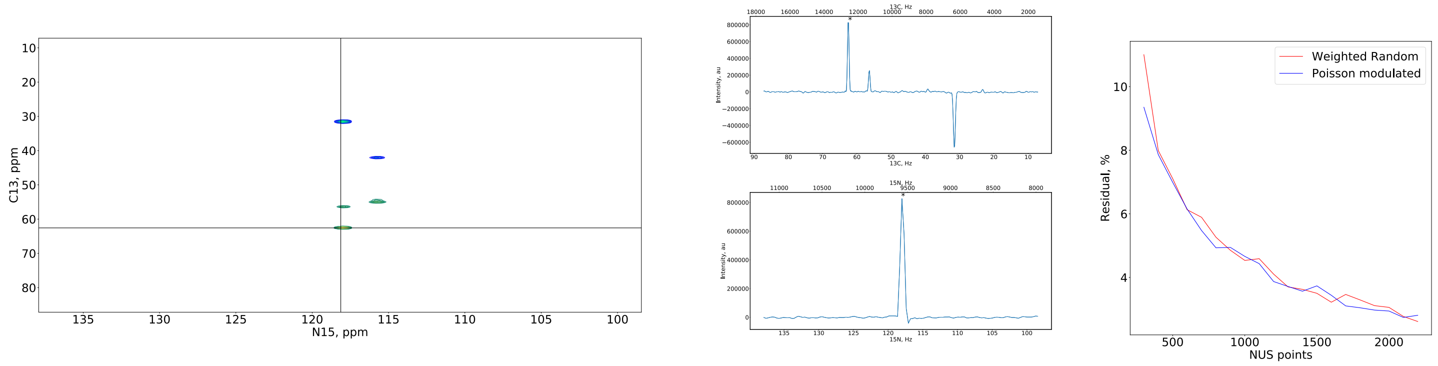

Peak208

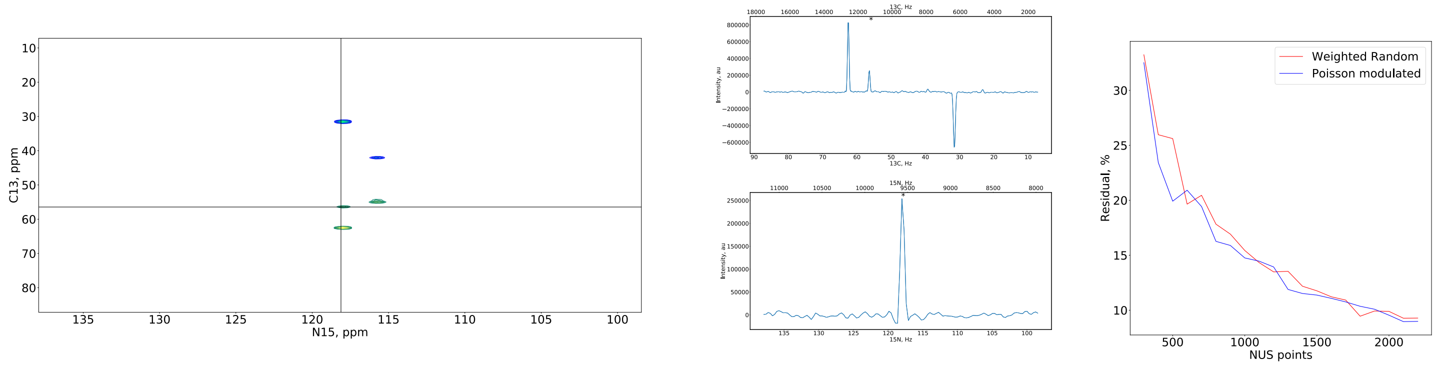

Peak209

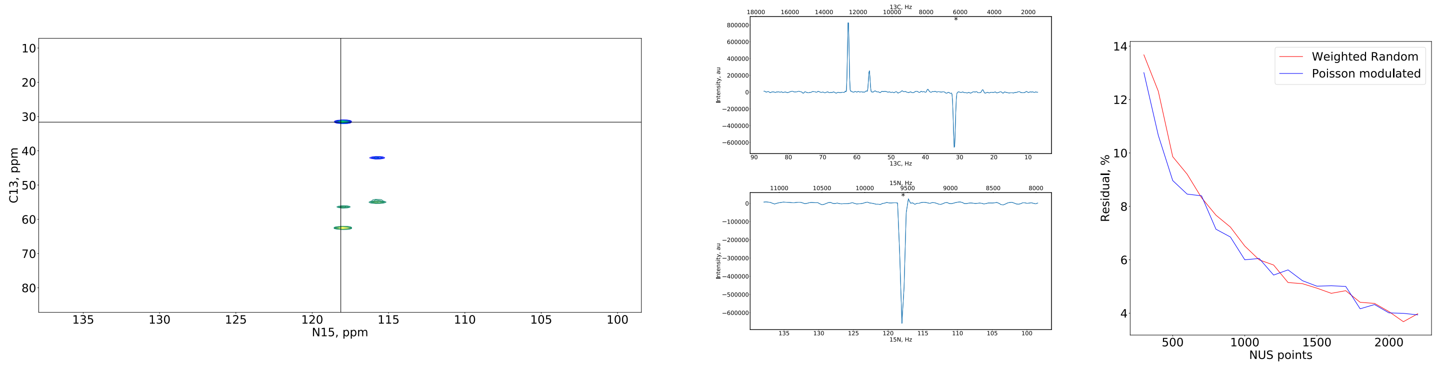

Peak210

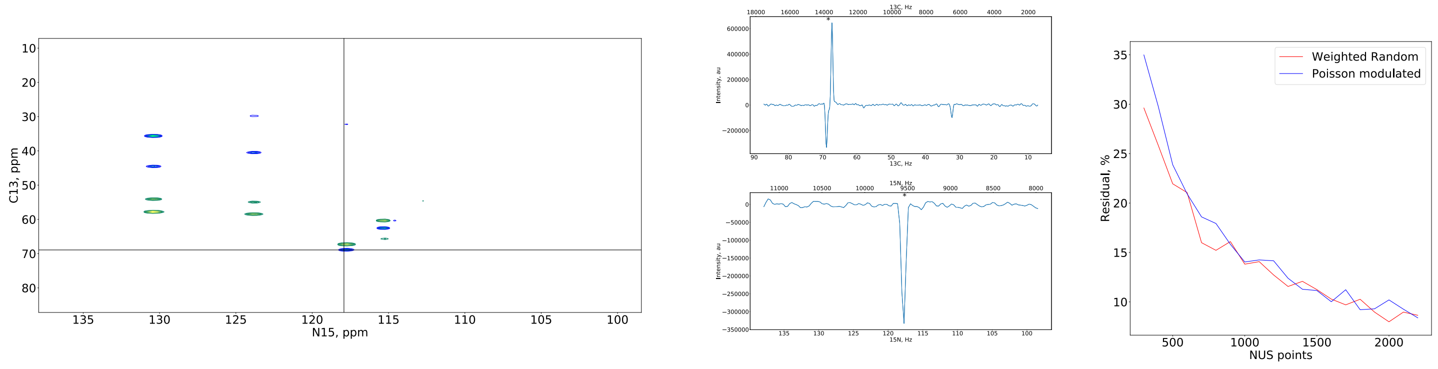

Peak211

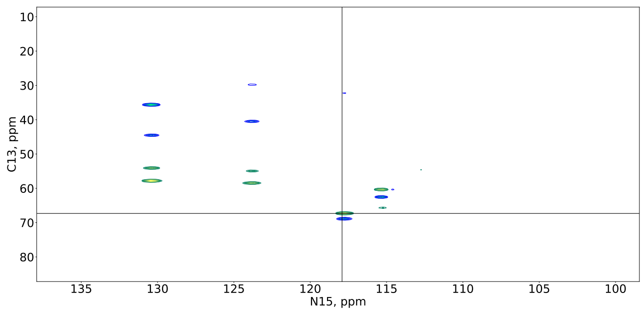

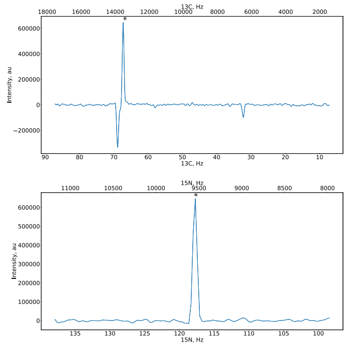

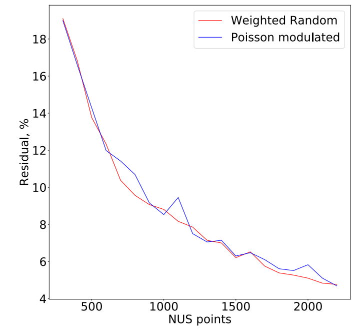

Peak212

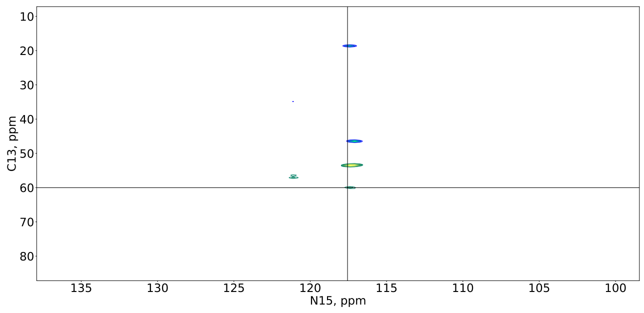

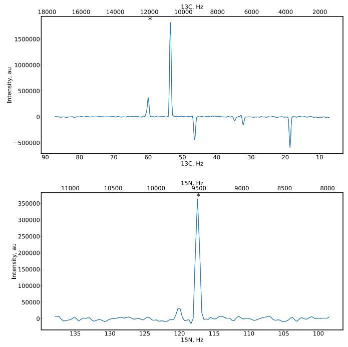

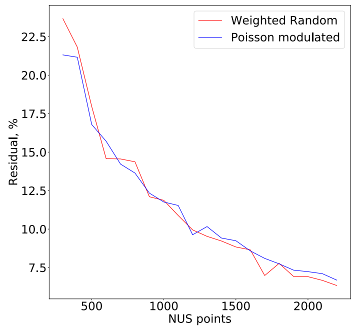

Peak213

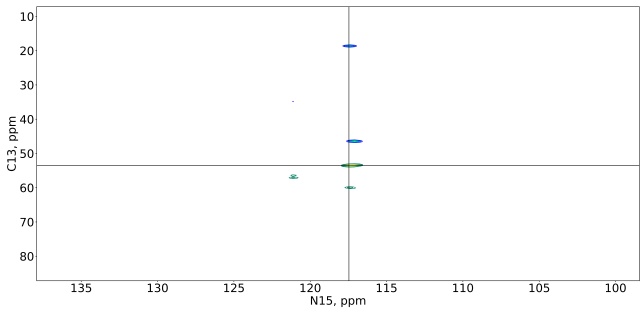

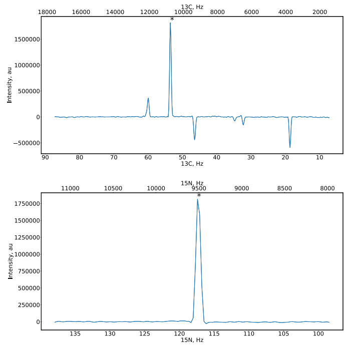

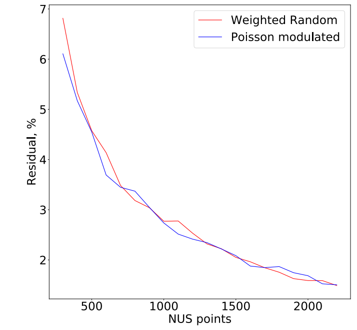

Peak214

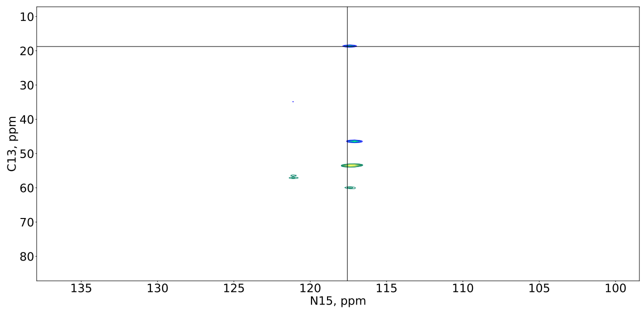

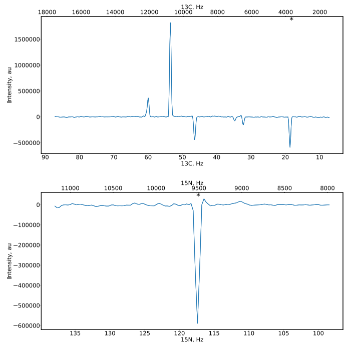

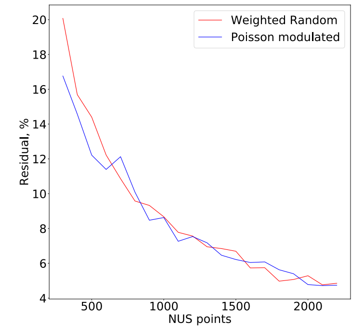

Peak215

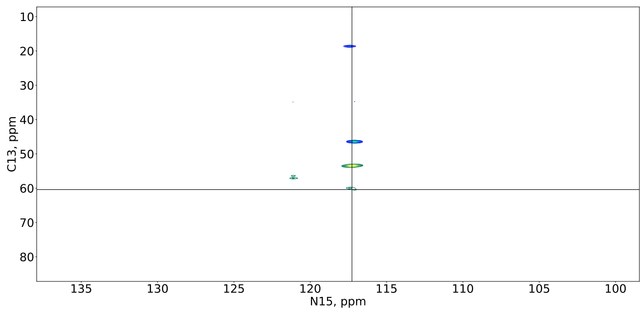

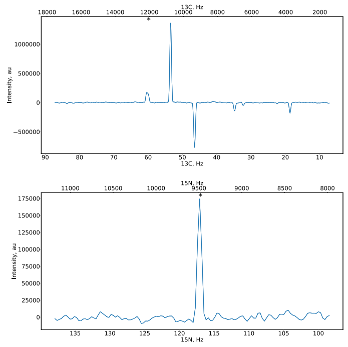

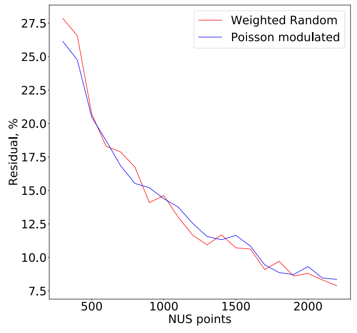

Peak216

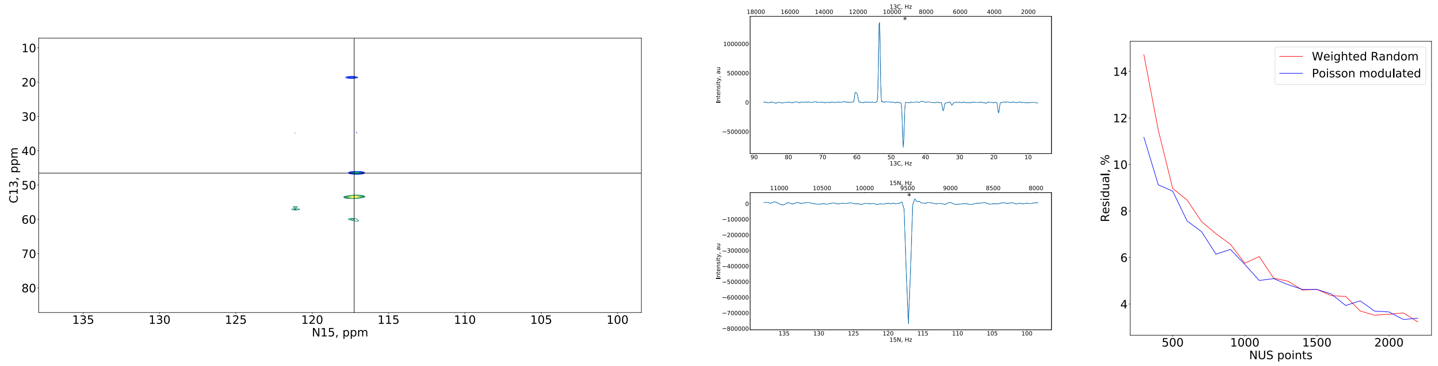

Peak217

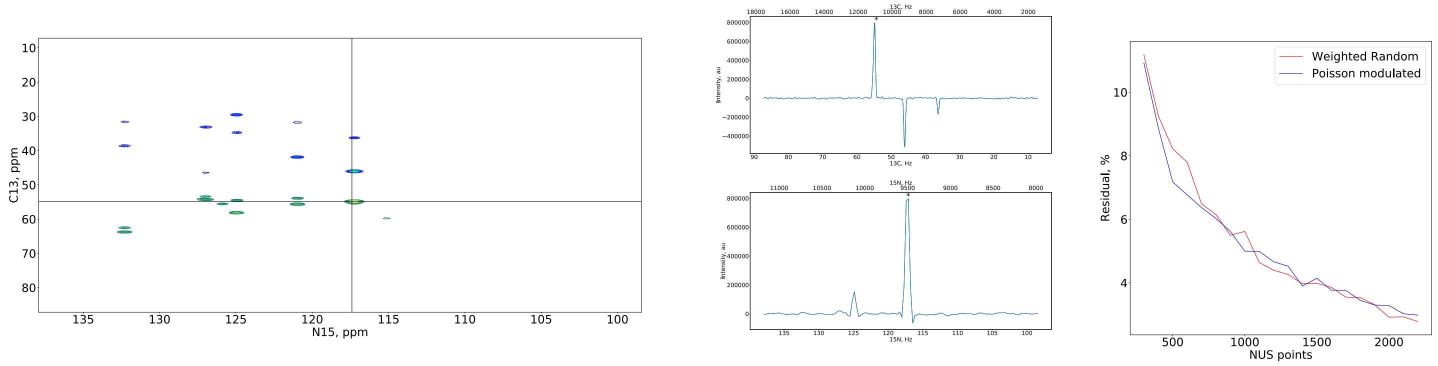

Peak218

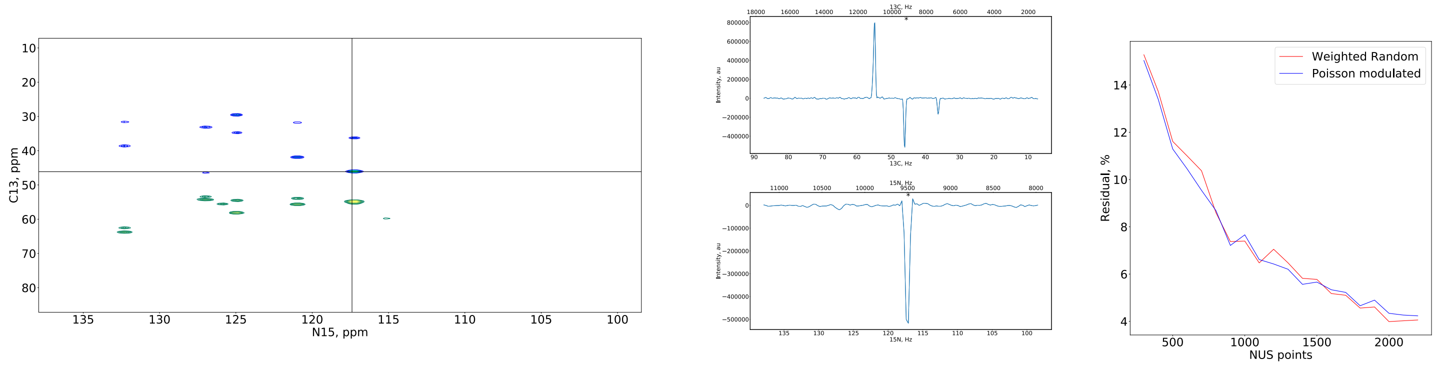

Peak219

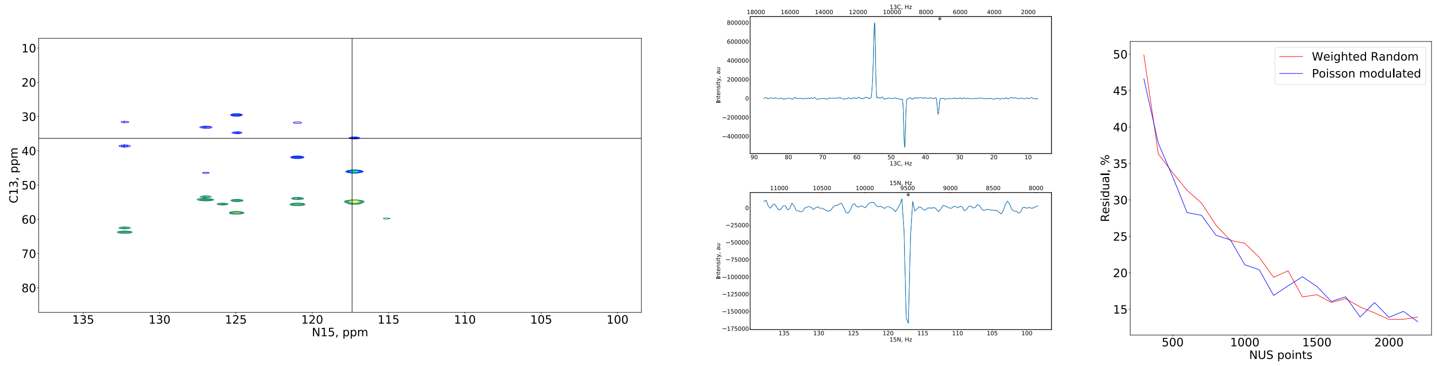

Peak220

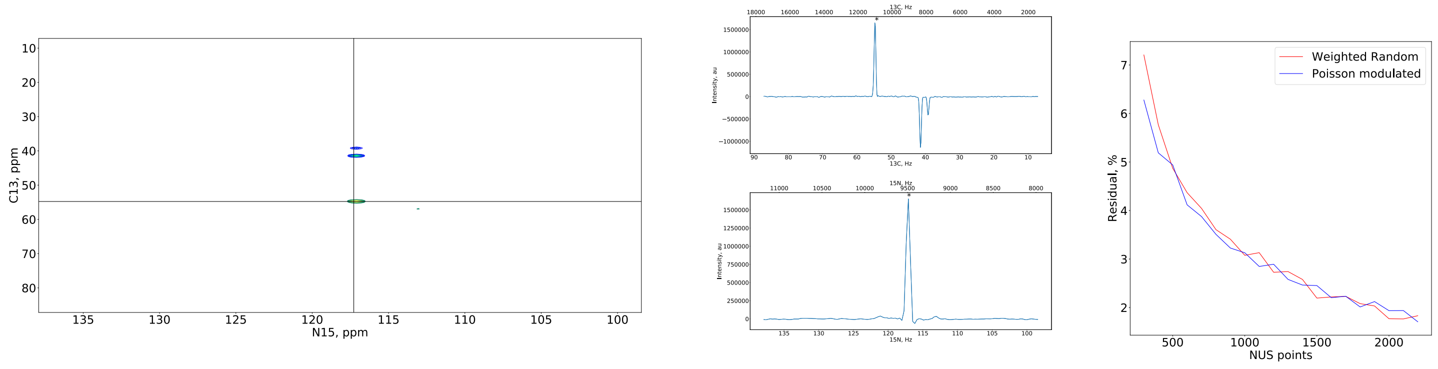

Peak221

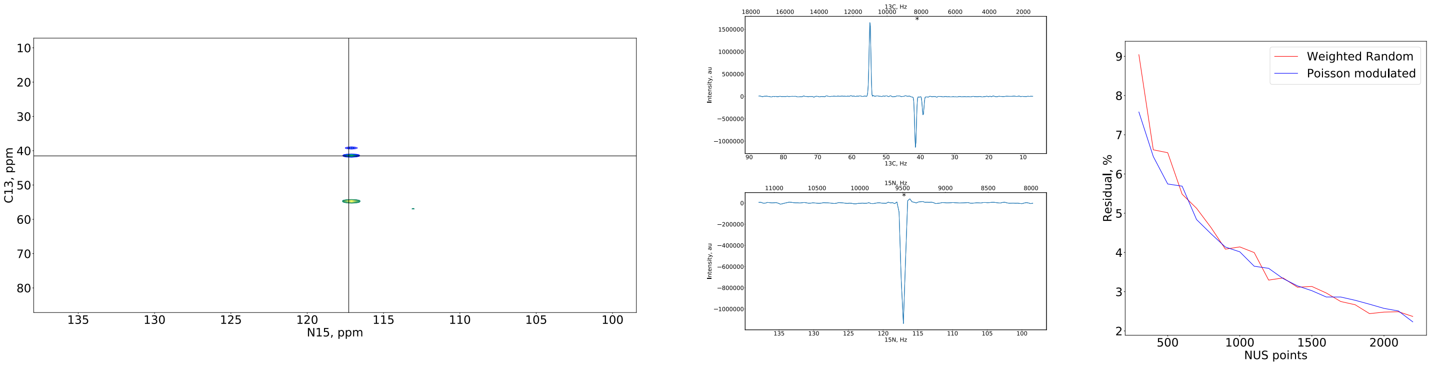

Peak222

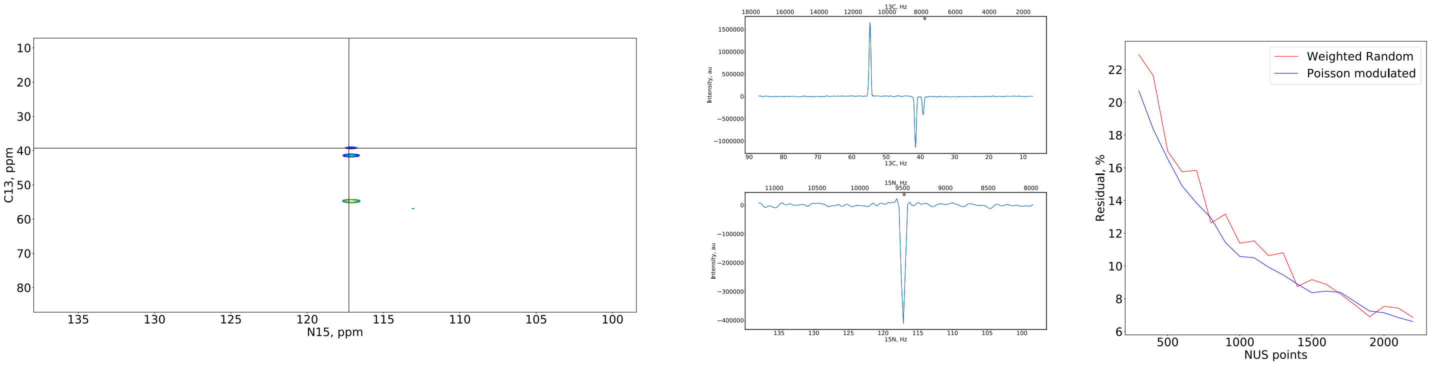

Peak223

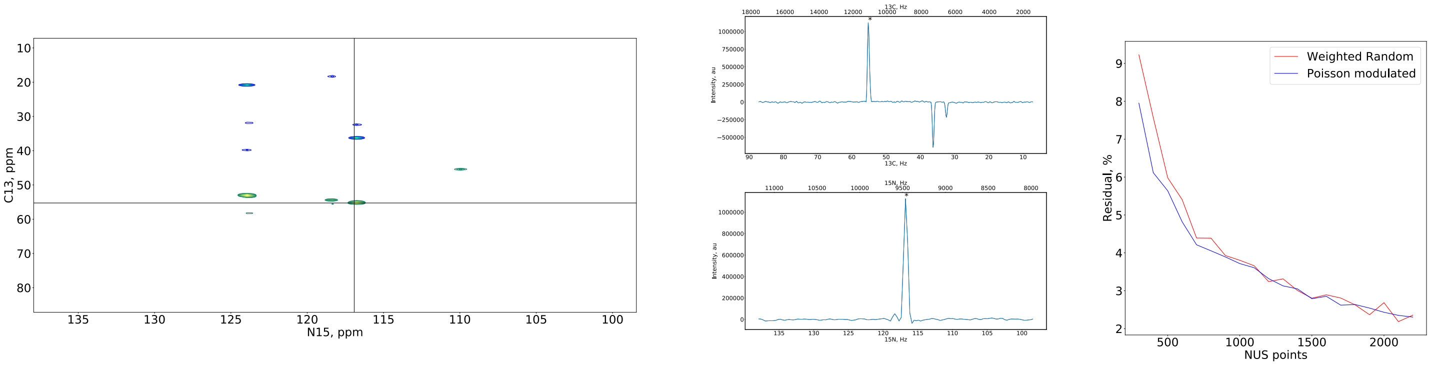

Peak224

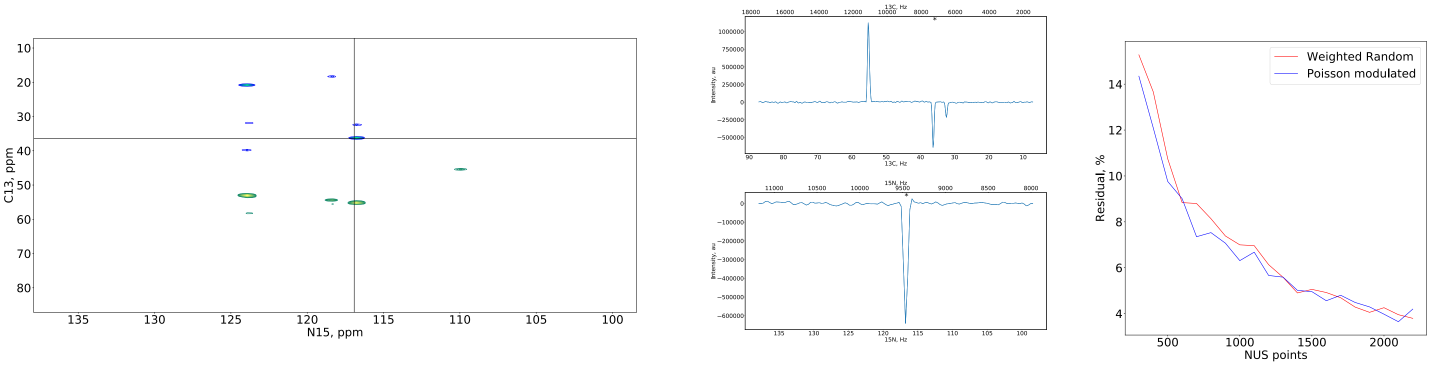

Peak225

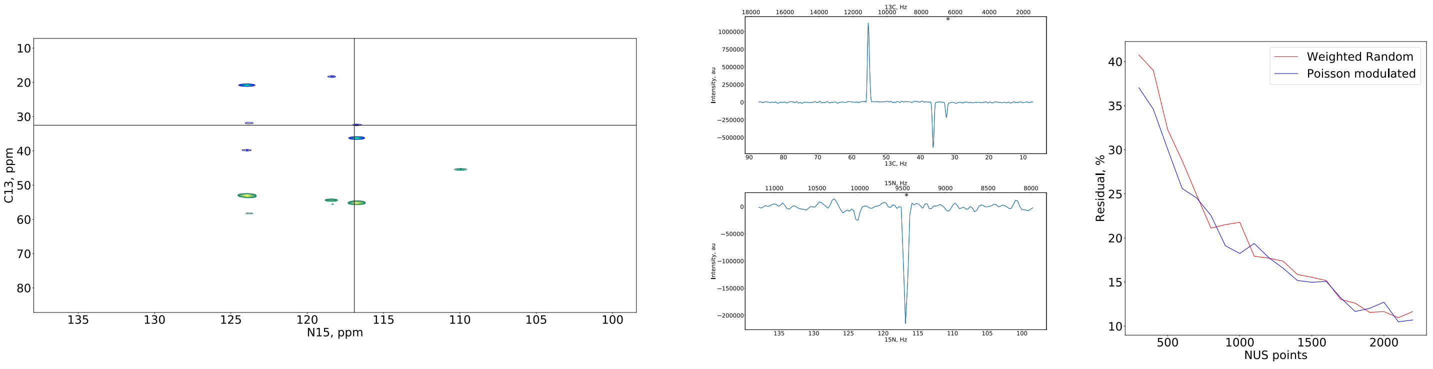

Peak226

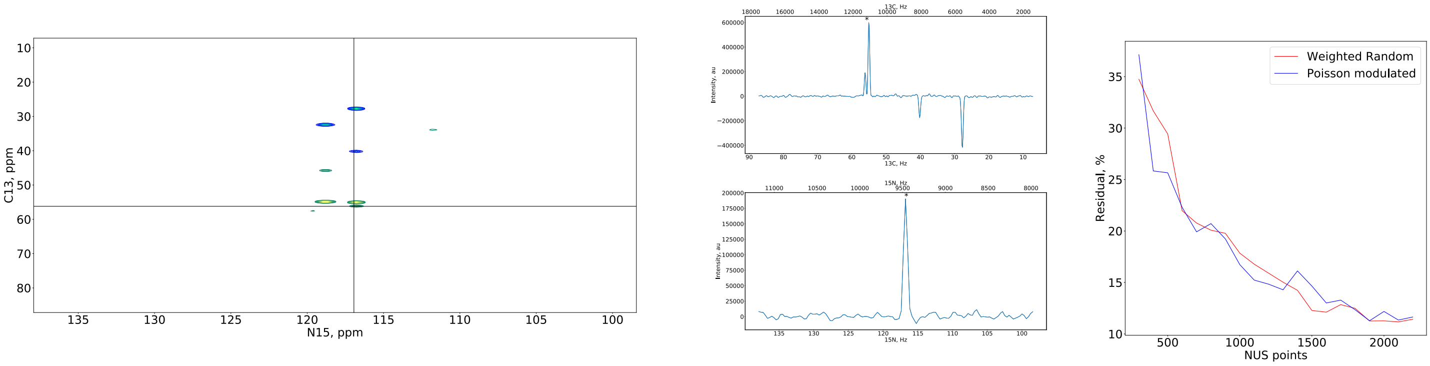

Peak227

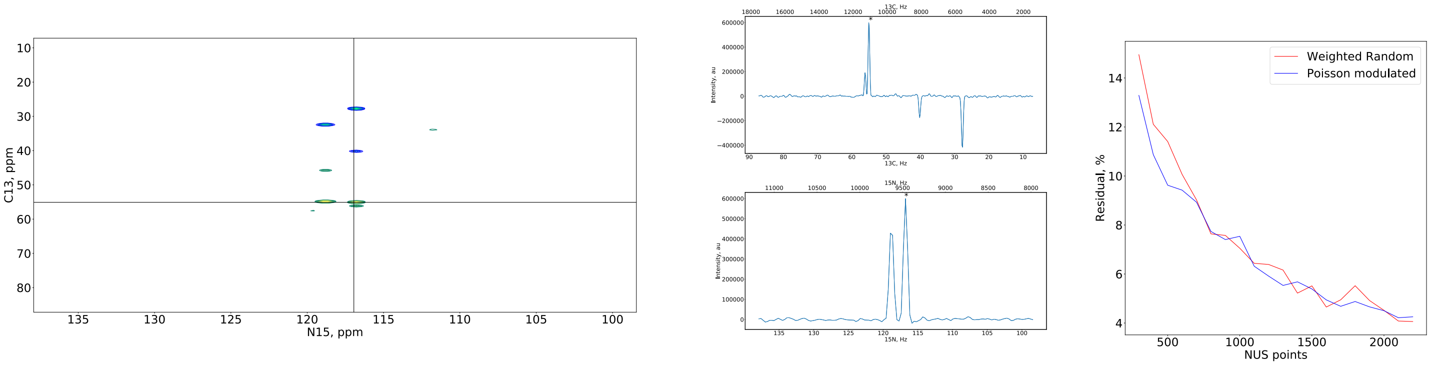

Peak228

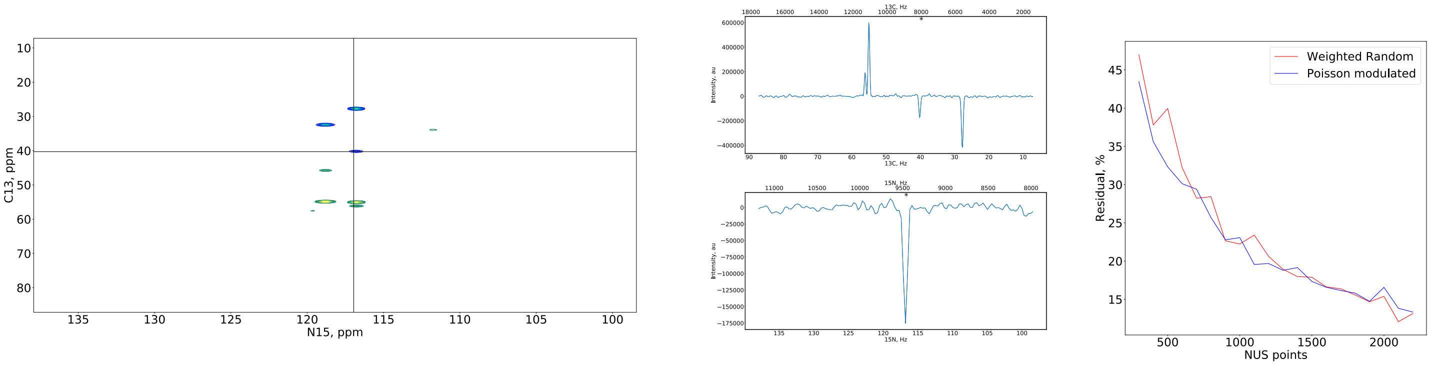

Peak229

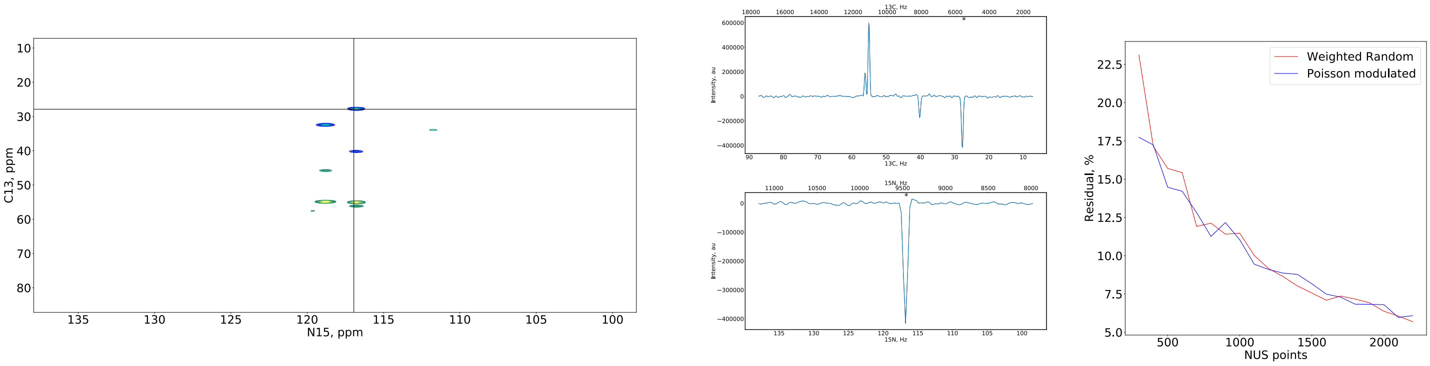

Peak230

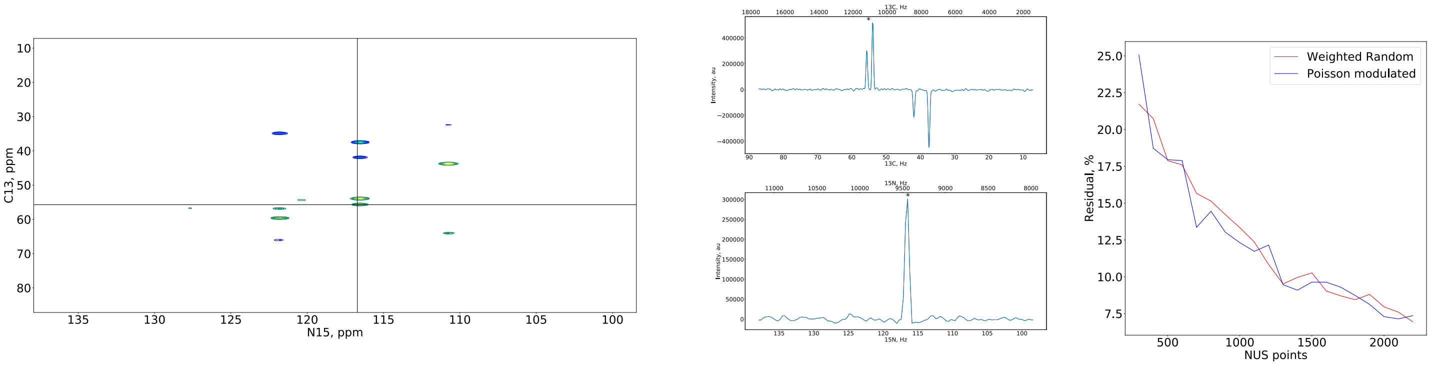

Peak231

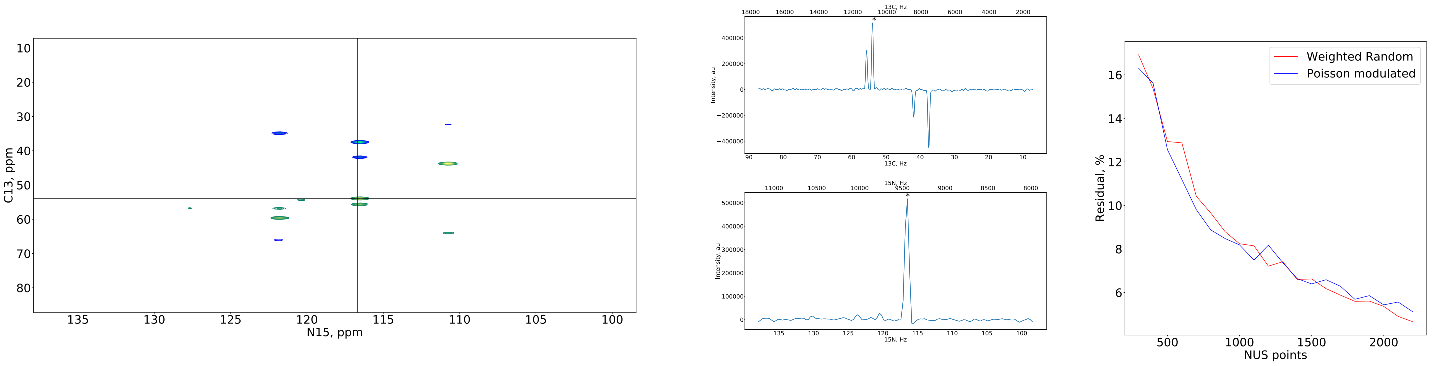

Peak232

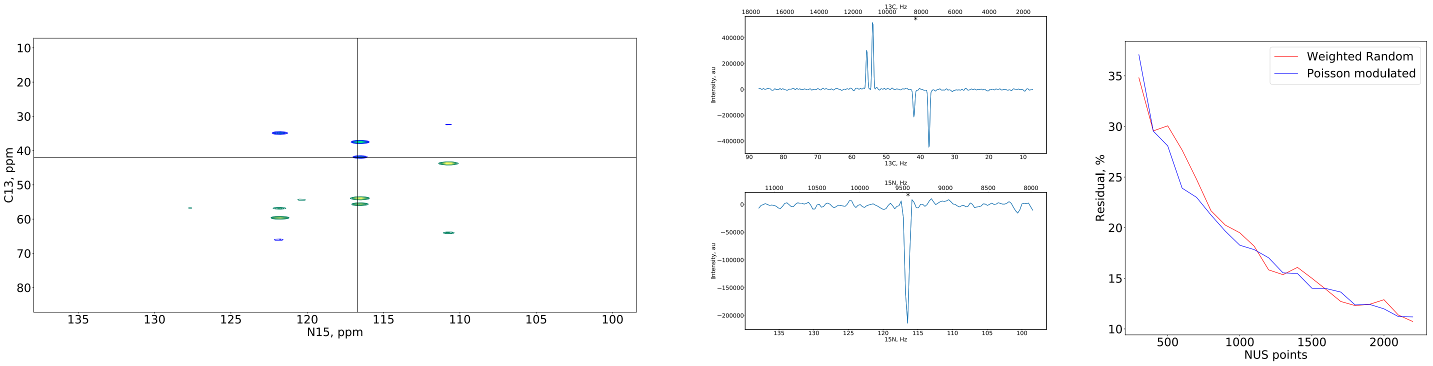

Peak233

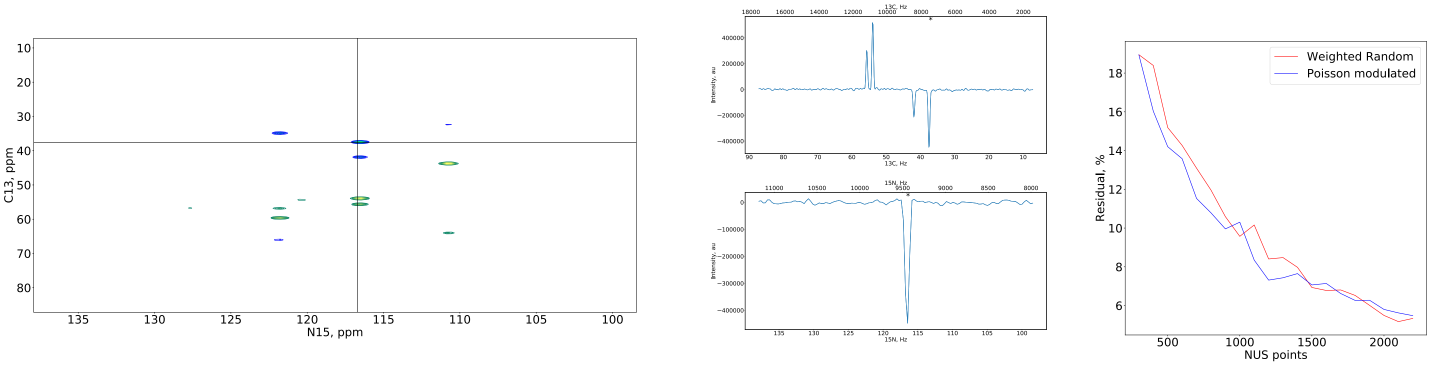

Peak234

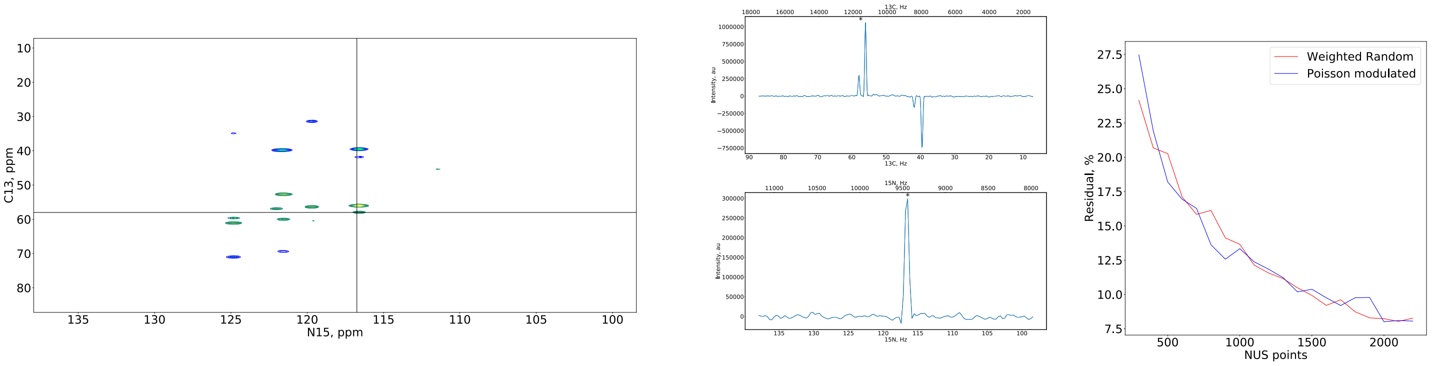

Peak235

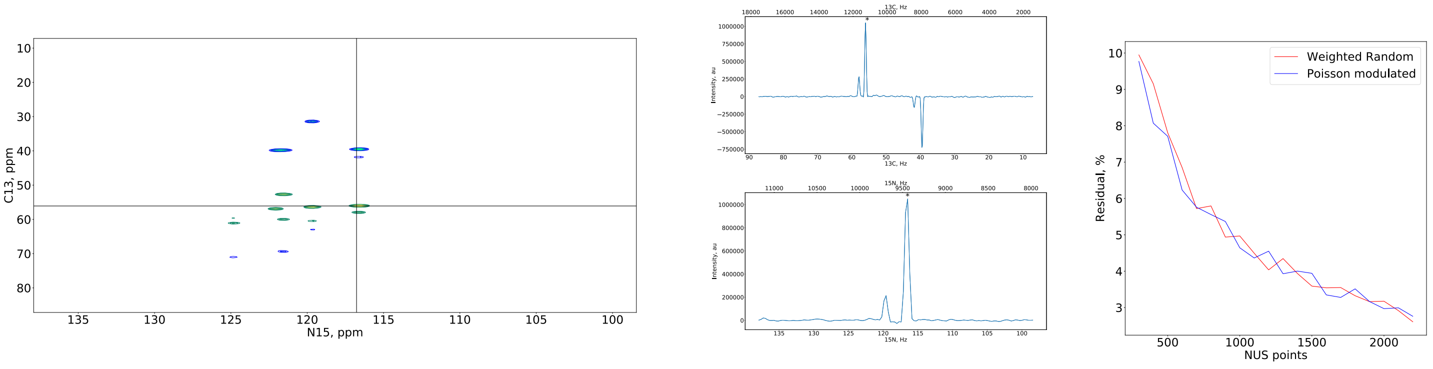

Peak236

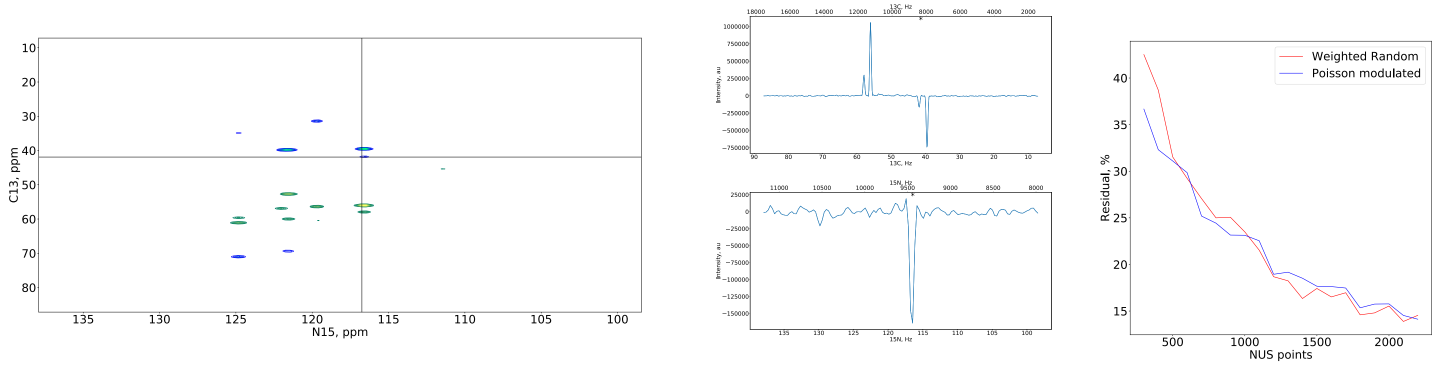

Peak237

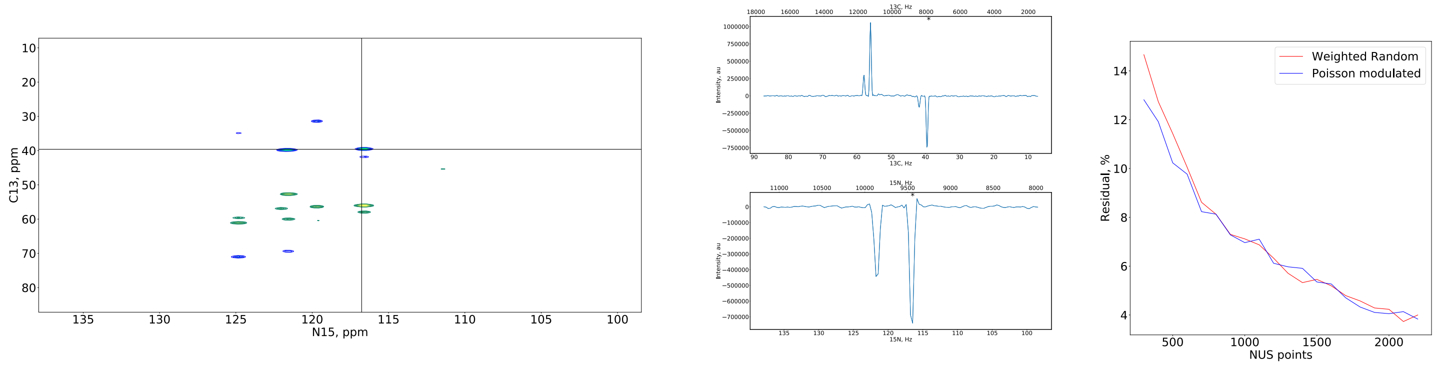

Peak238

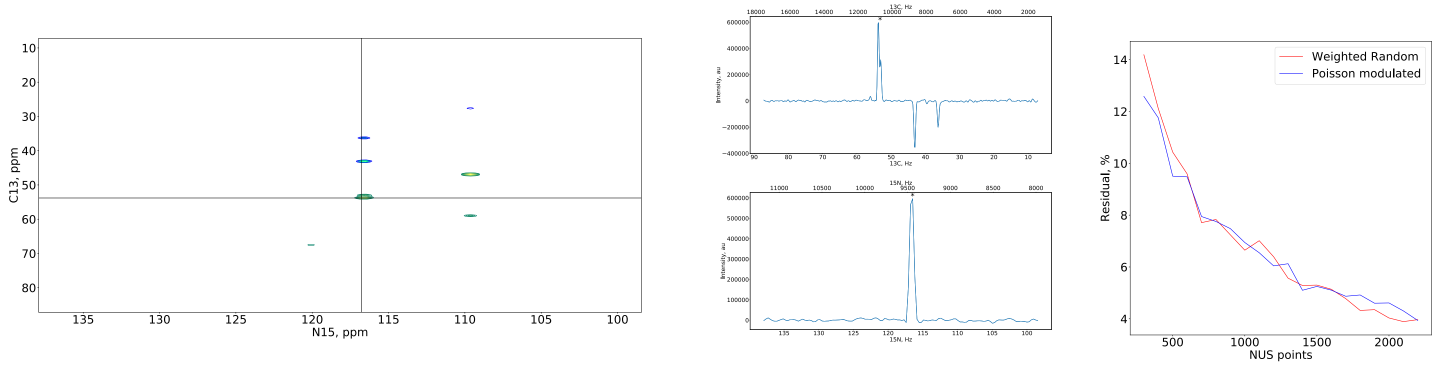

Peak239

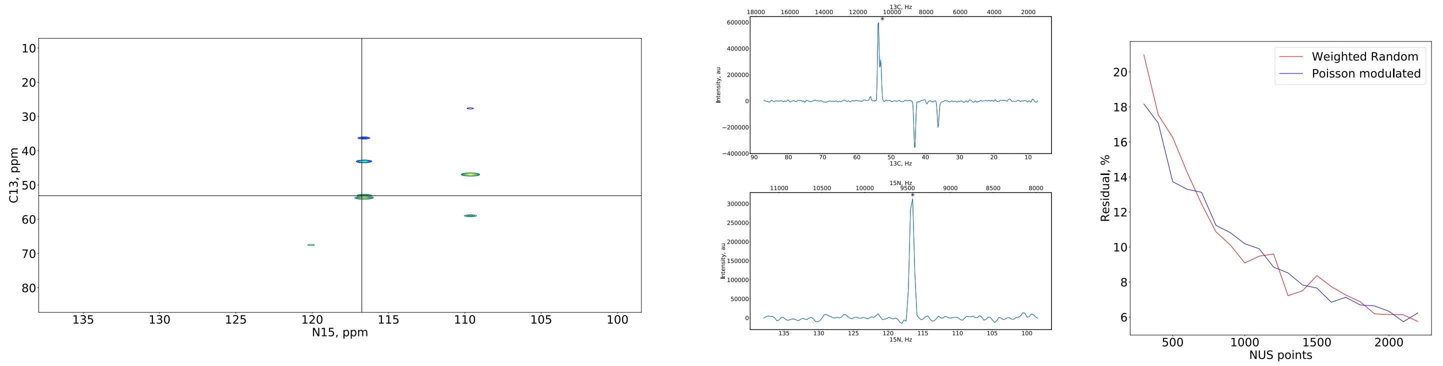

Peak240

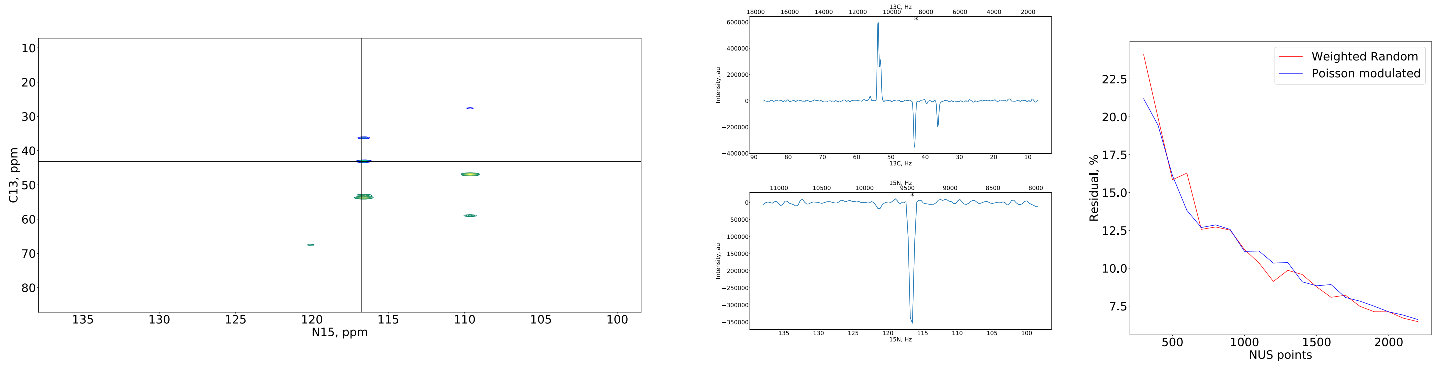

Peak241

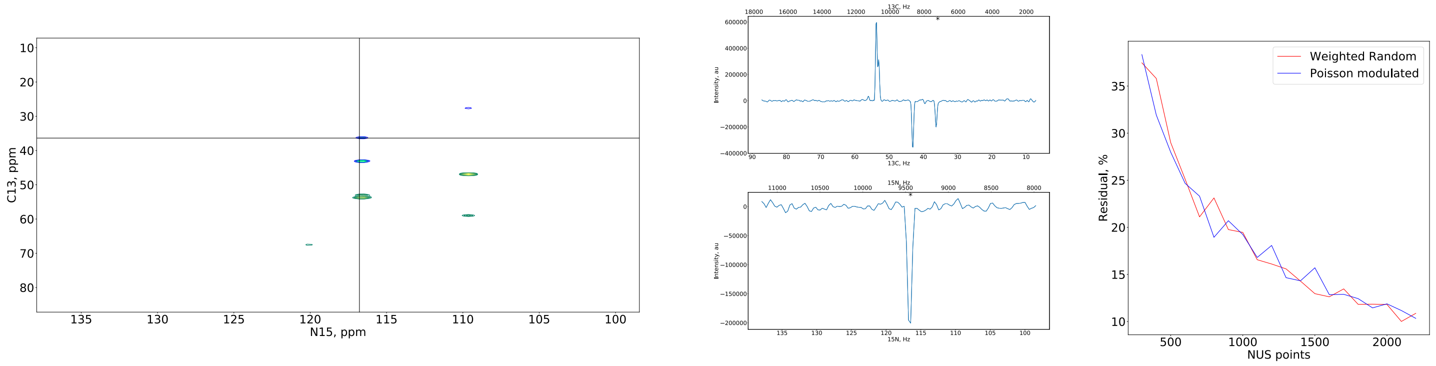

Peak242

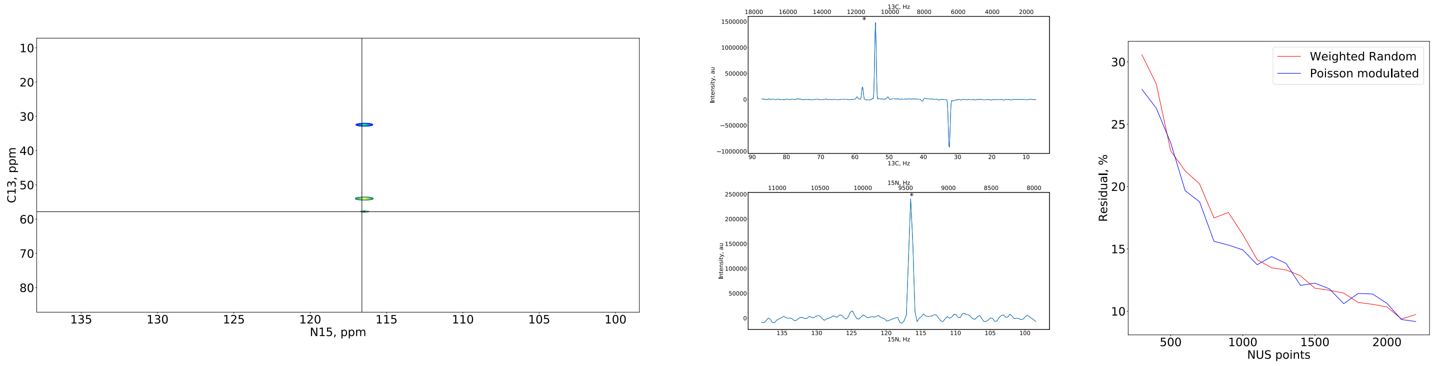

Peak243

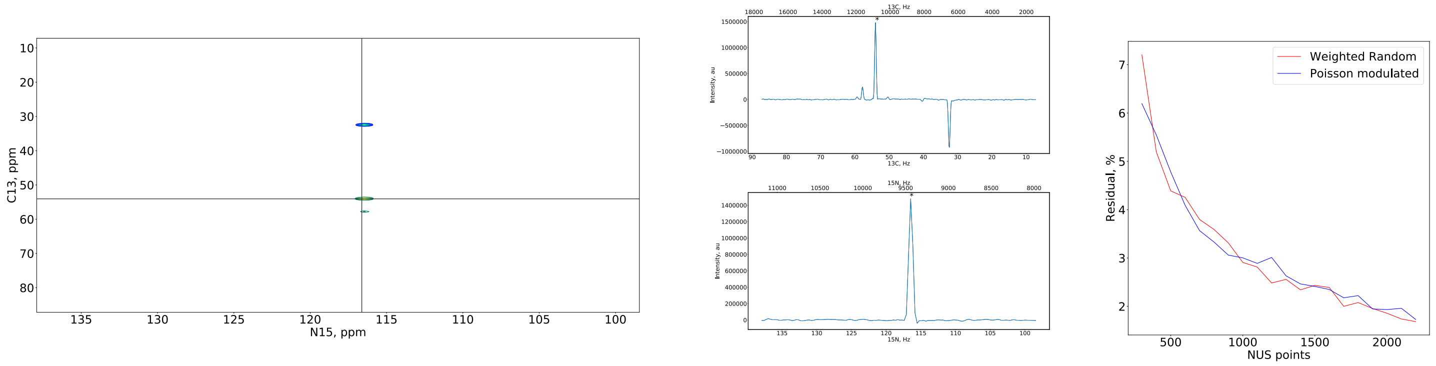

Peak244

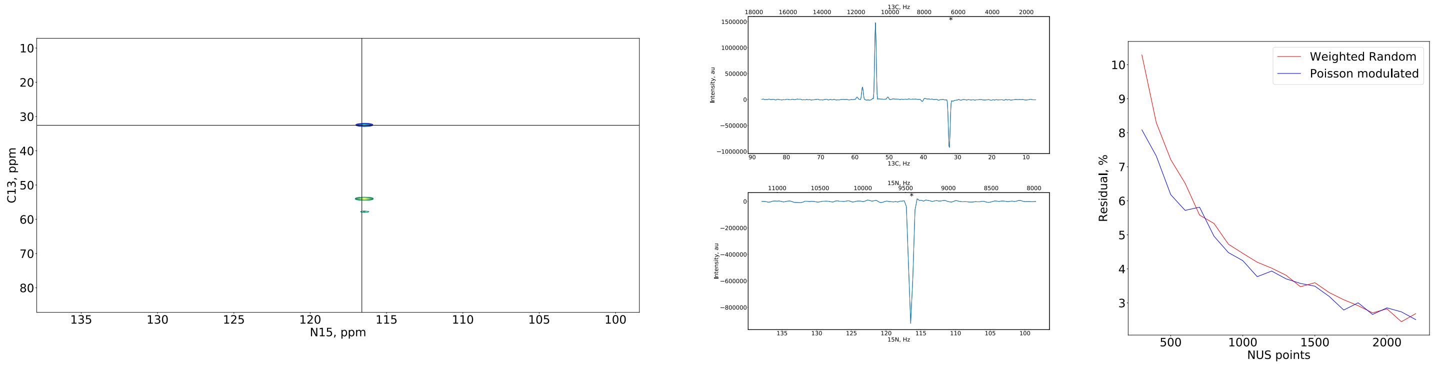

Peak245

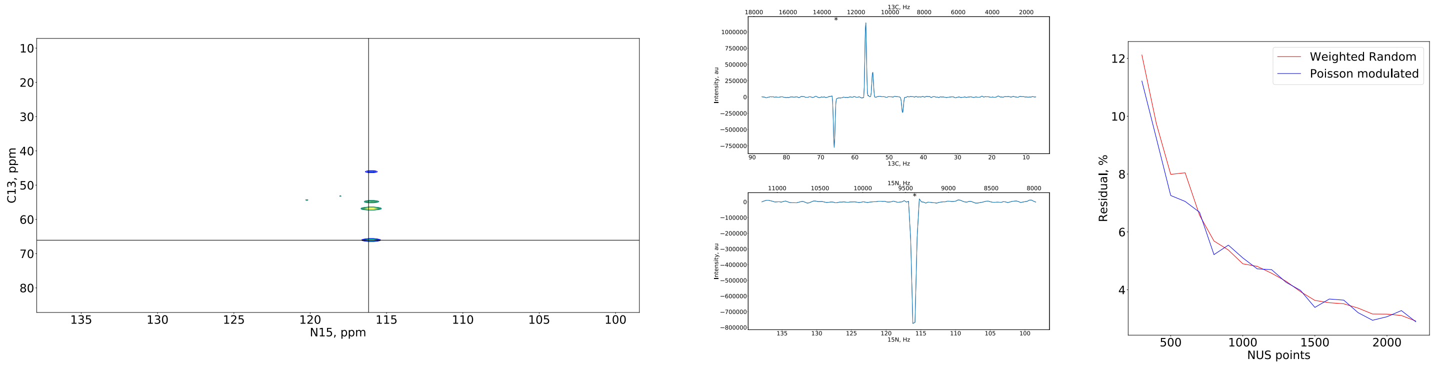

Peak246

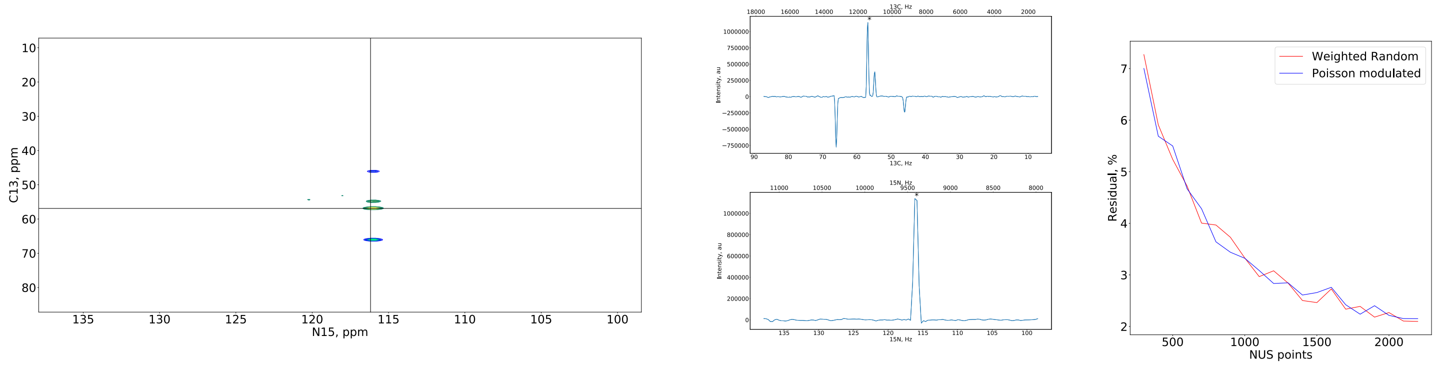

Peak247

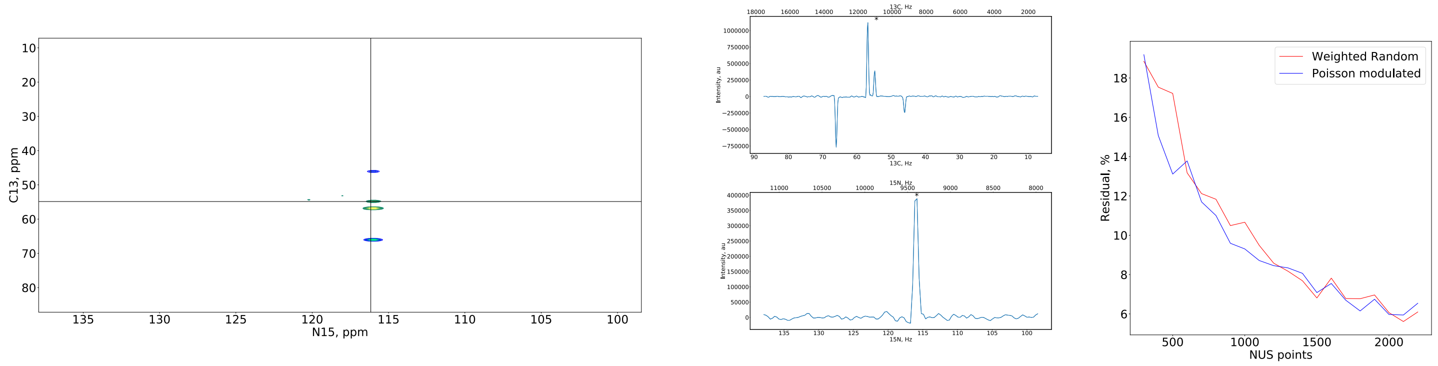

Peak248

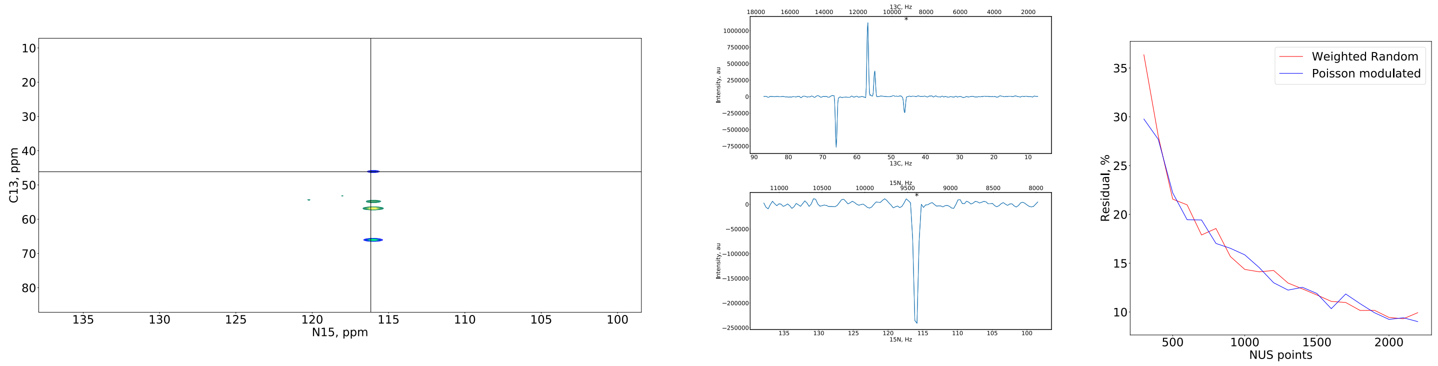

Peak249

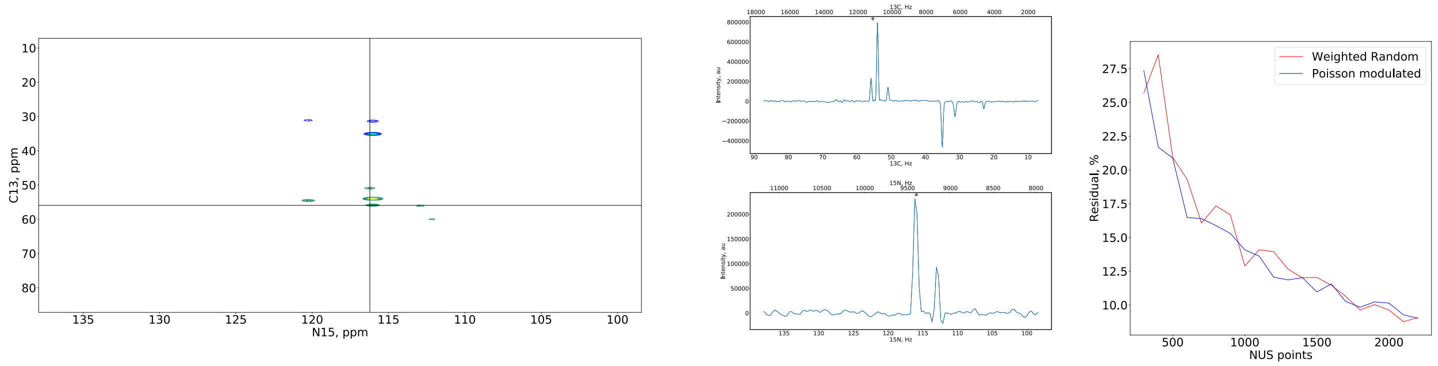

Peak250

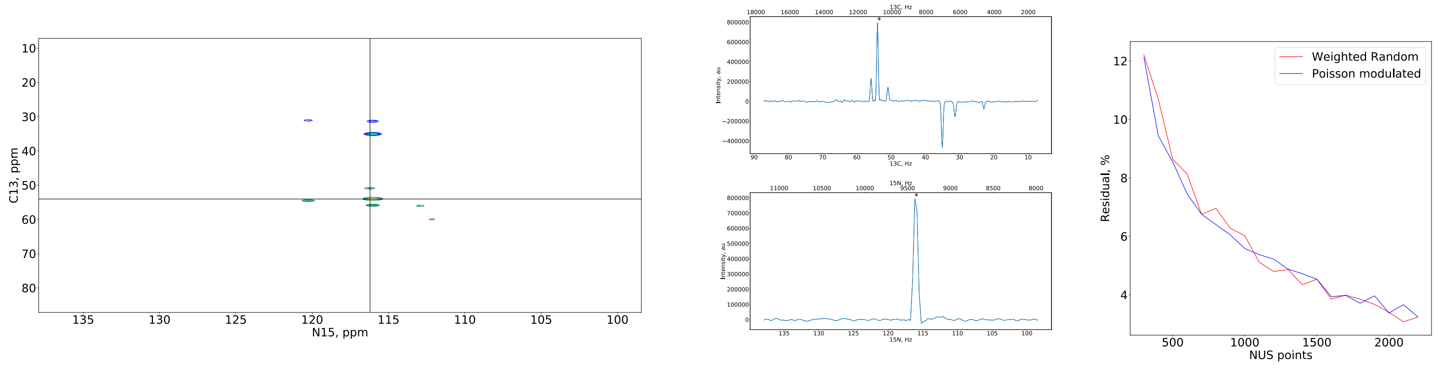

Peak251

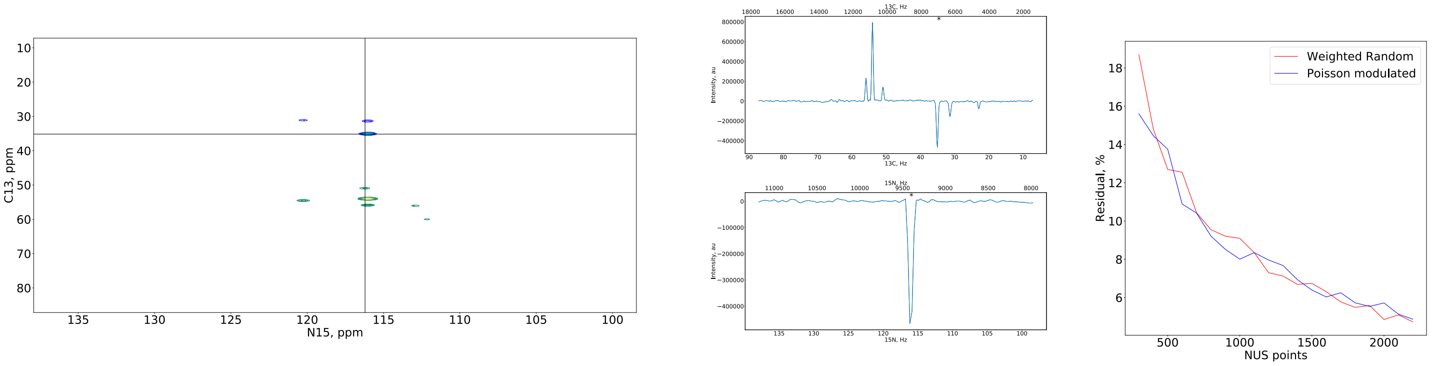

Peak252

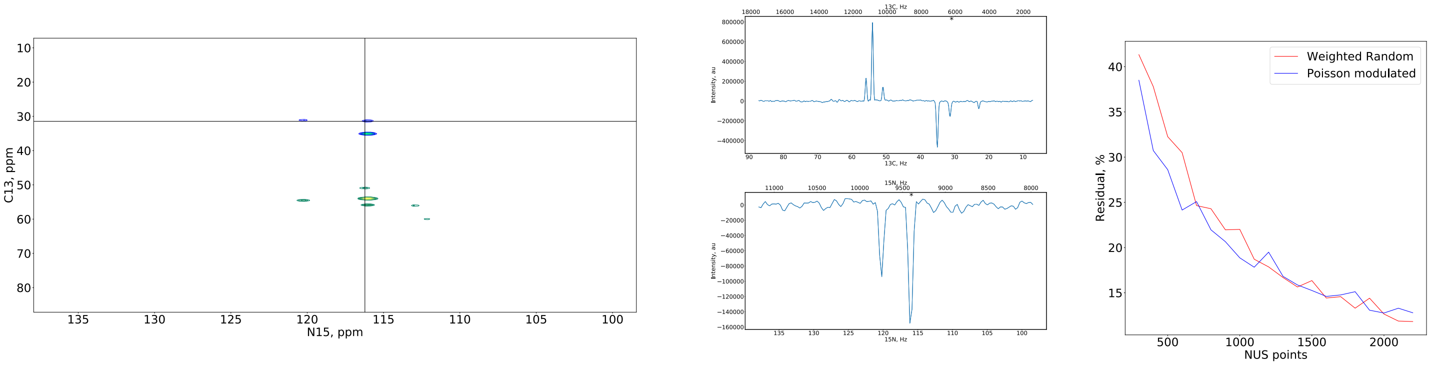

Peak253

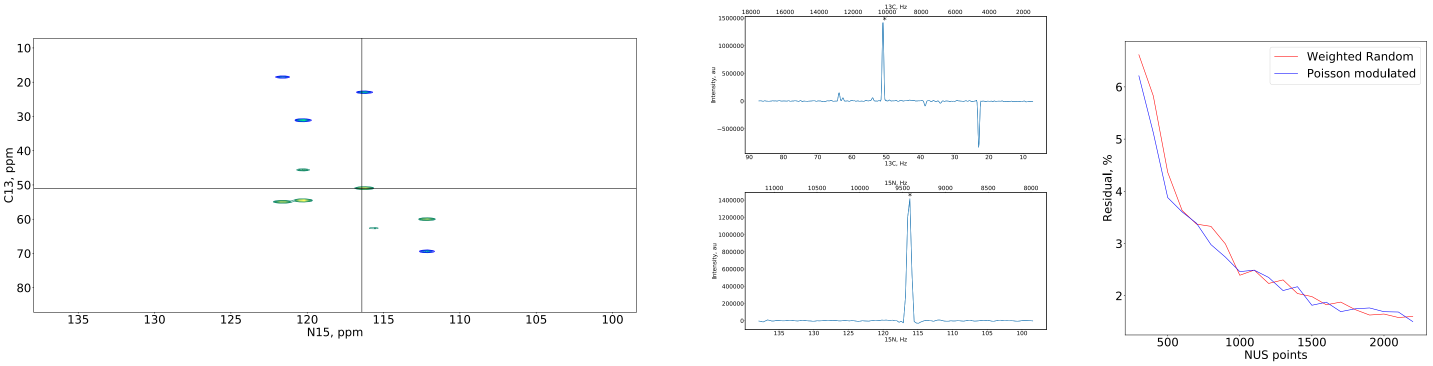

Peak254

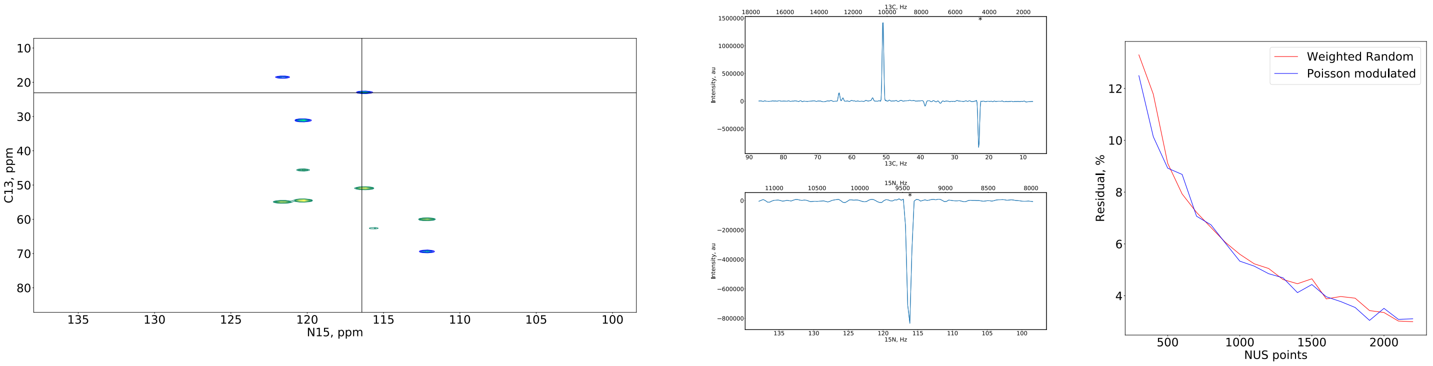

Peak255

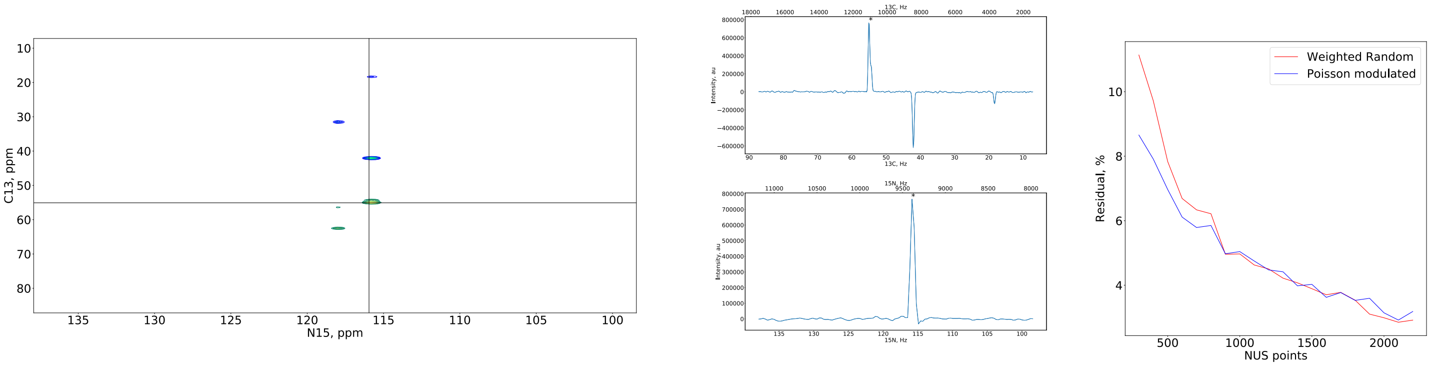

Peak256

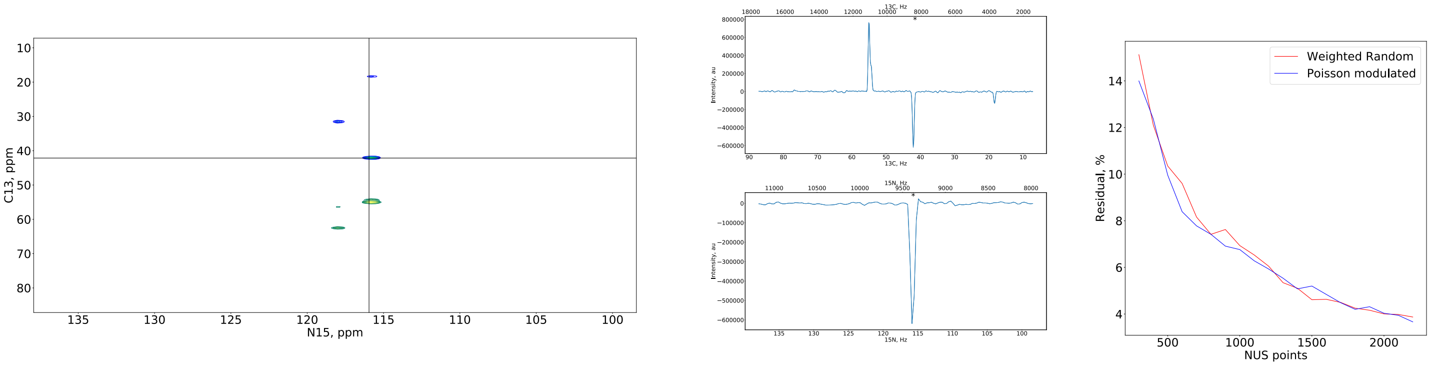

Peak257

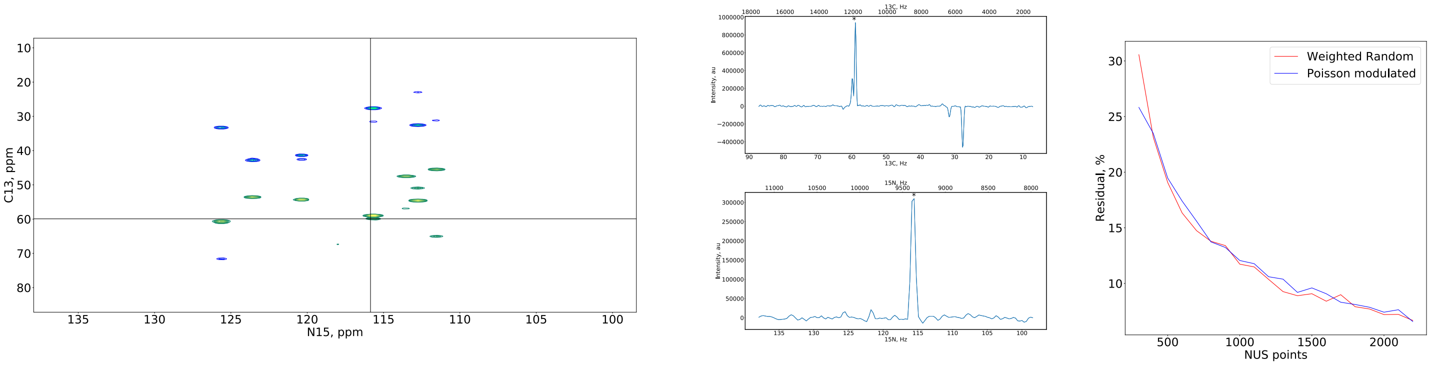

Peak258

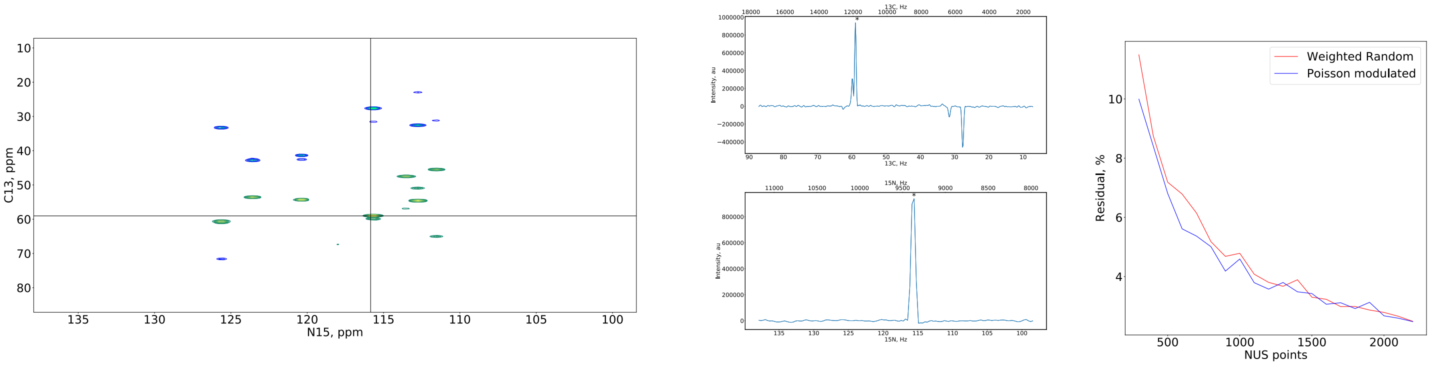

Peak259

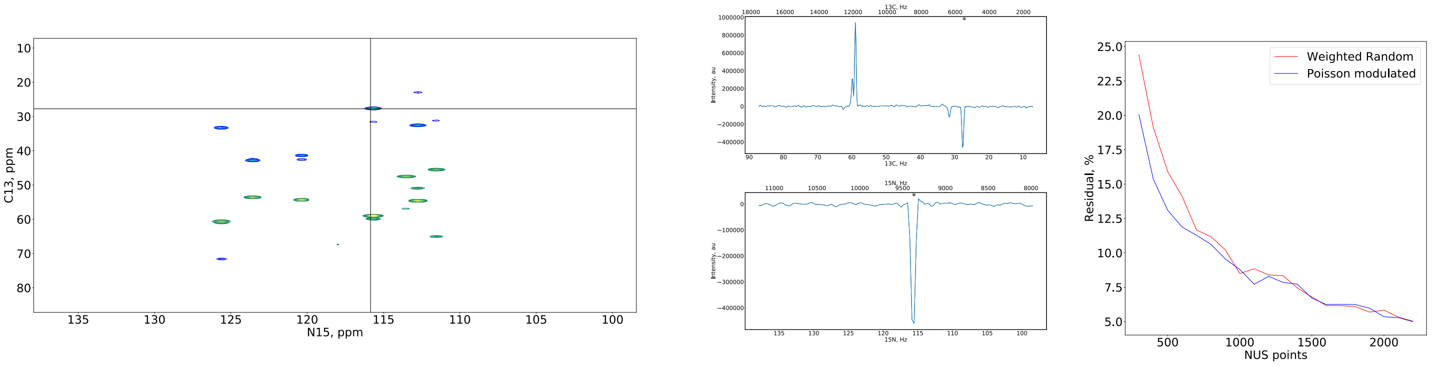

Peak260

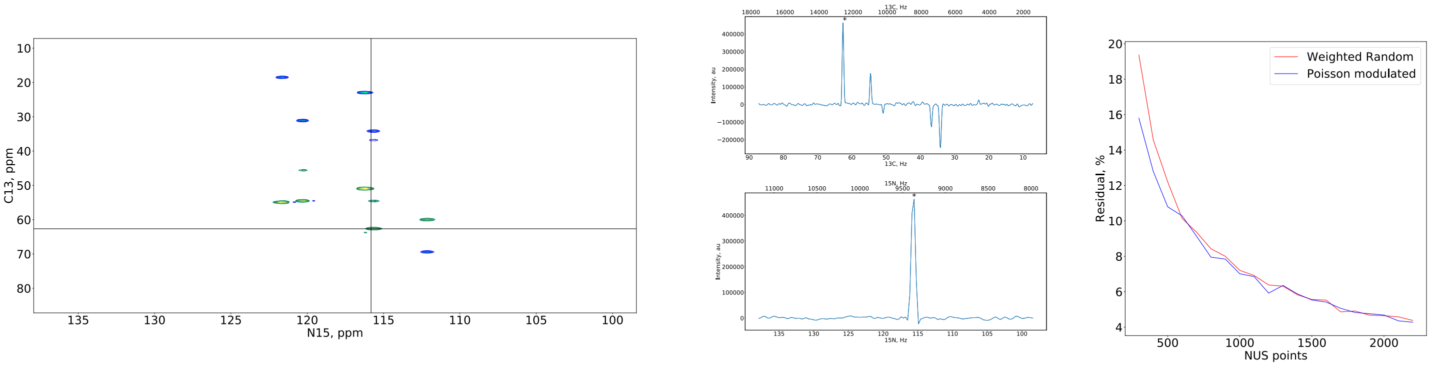

Peak261

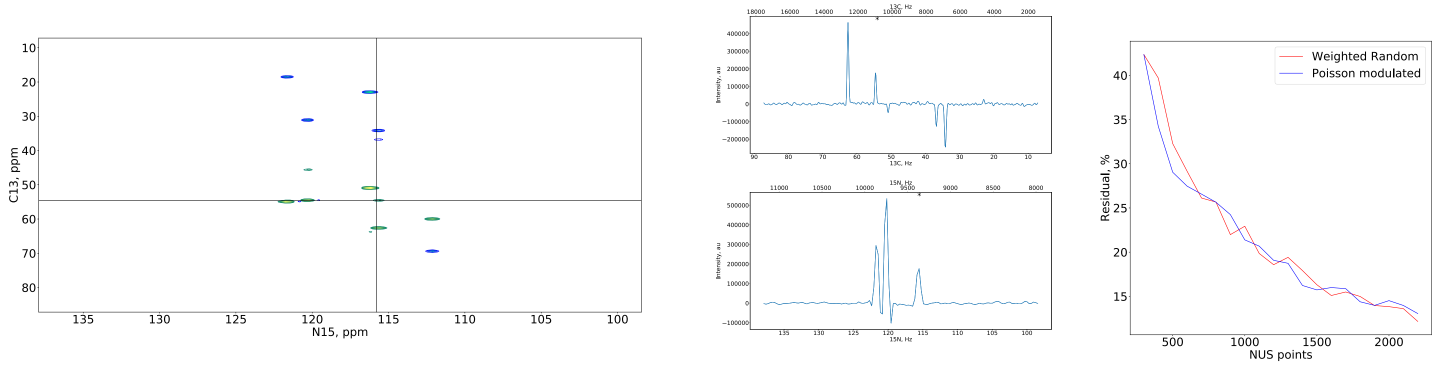

Peak262

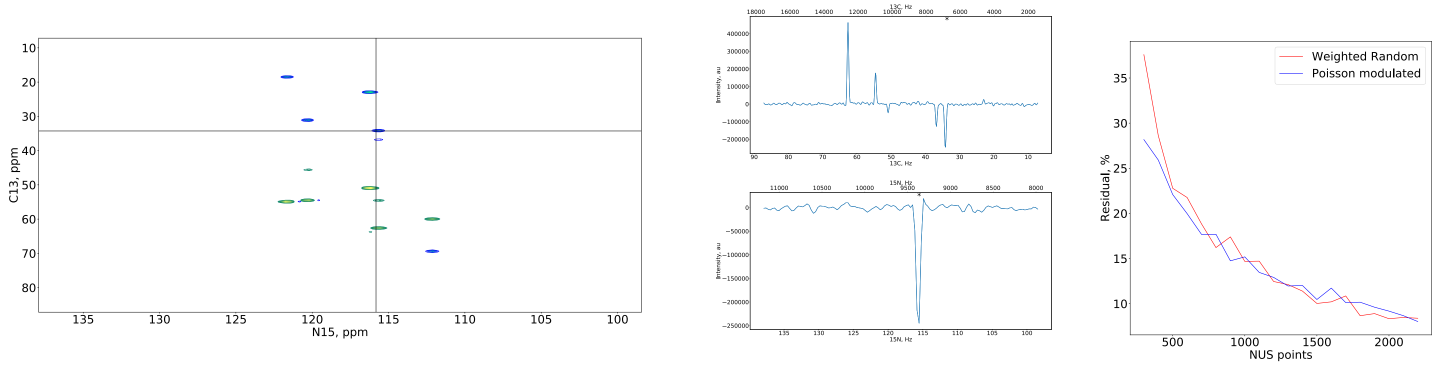

Peak263

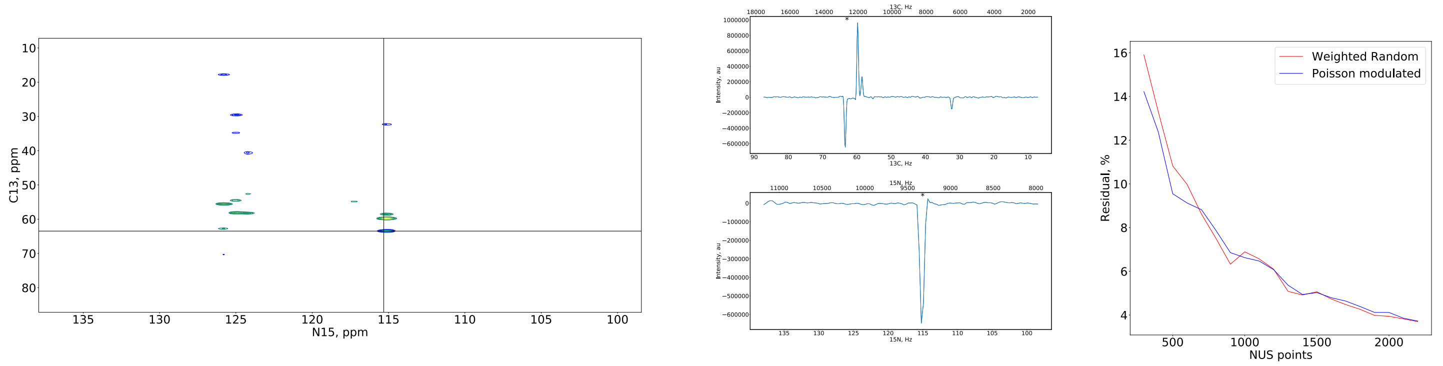

Peak264

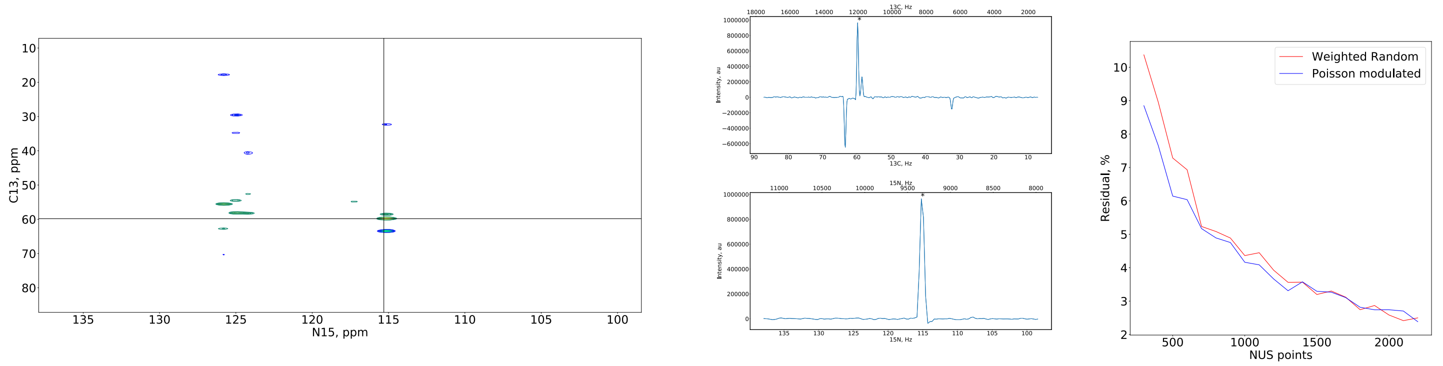

Peak265

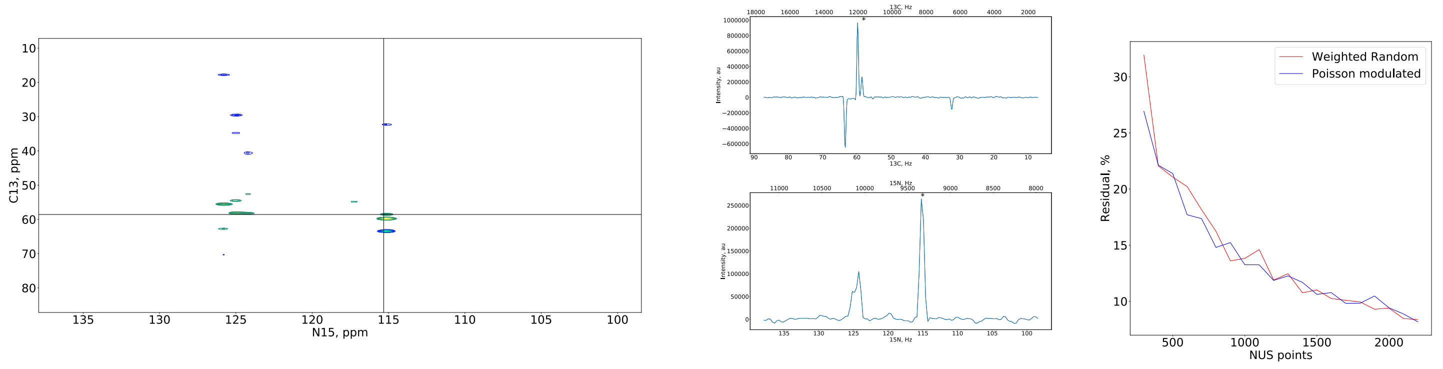

Peak266

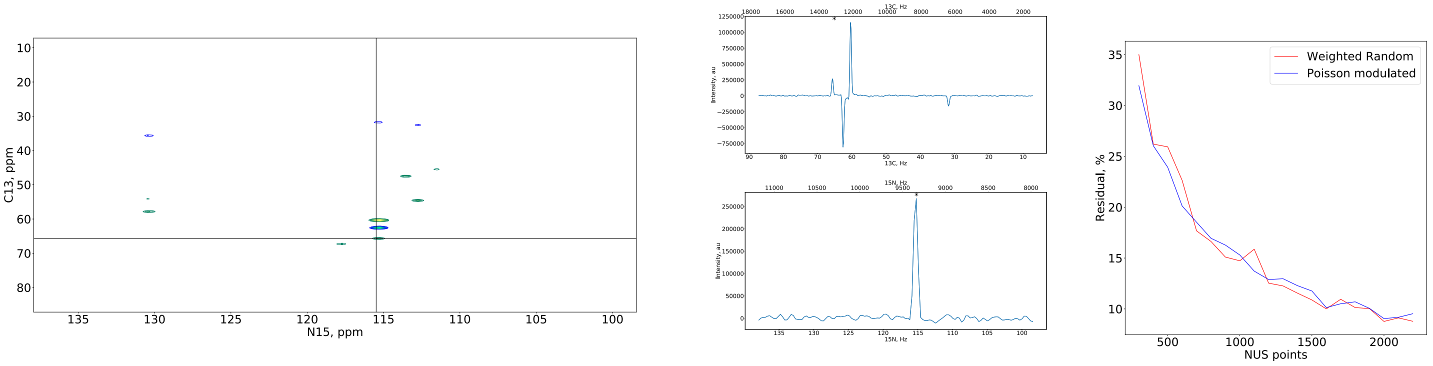

Peak267

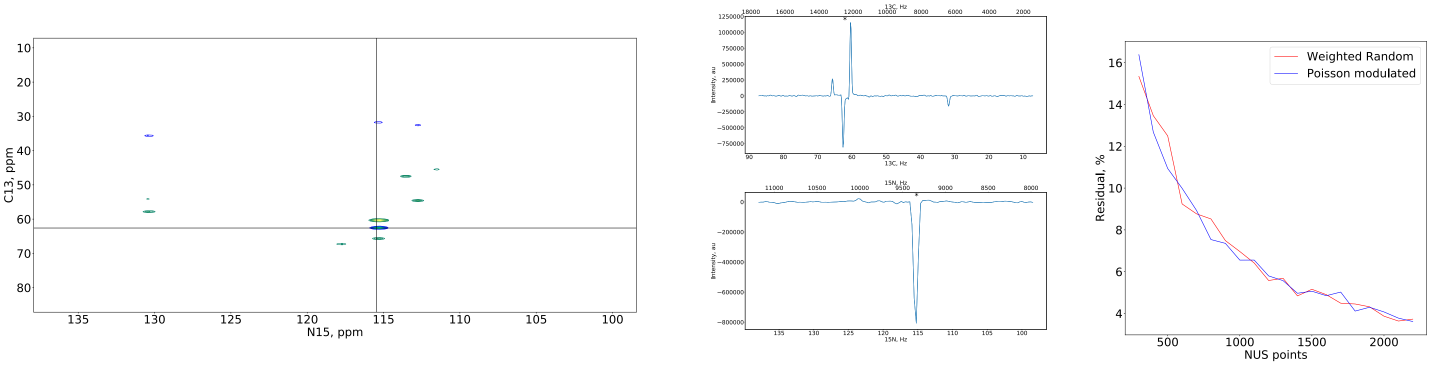

Peak268

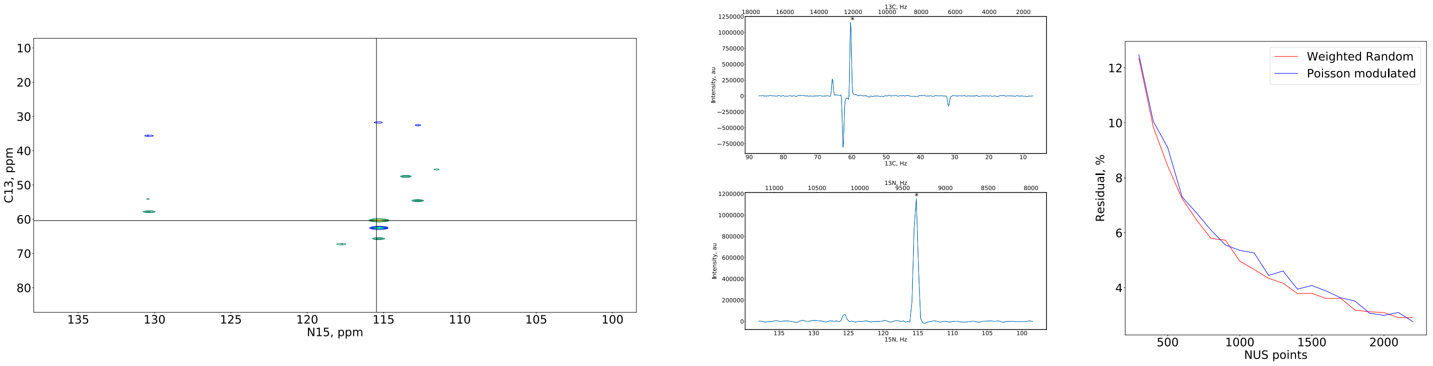

Peak269

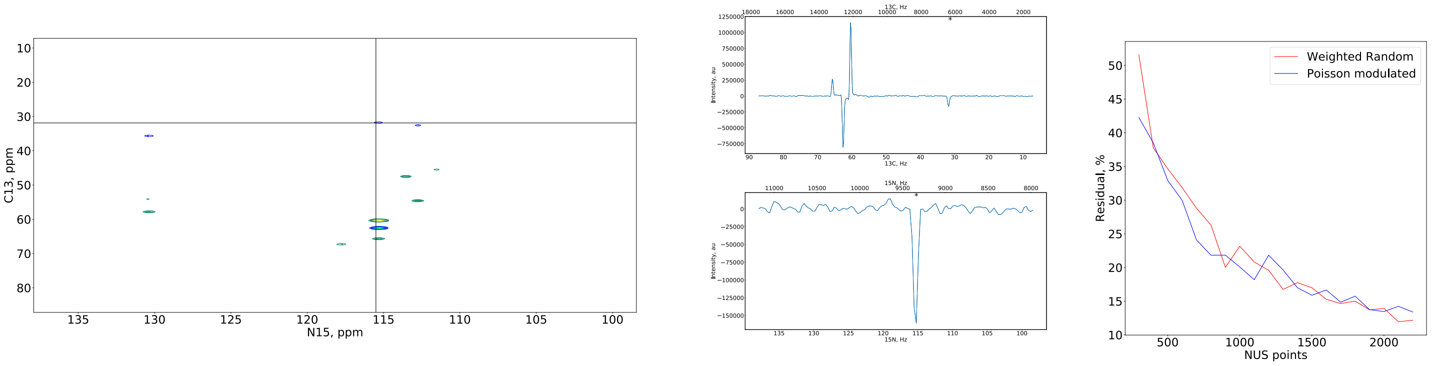

Peak270

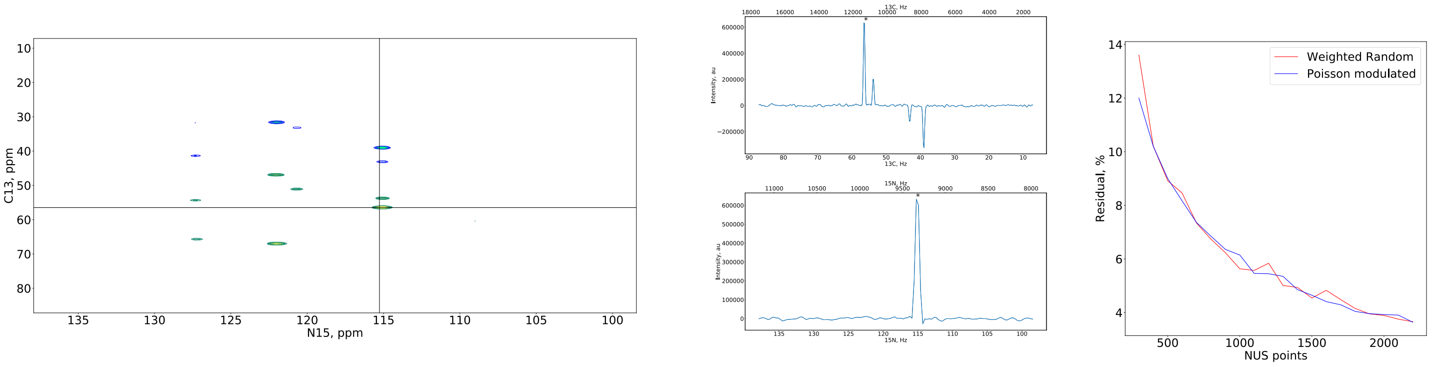

Peak271

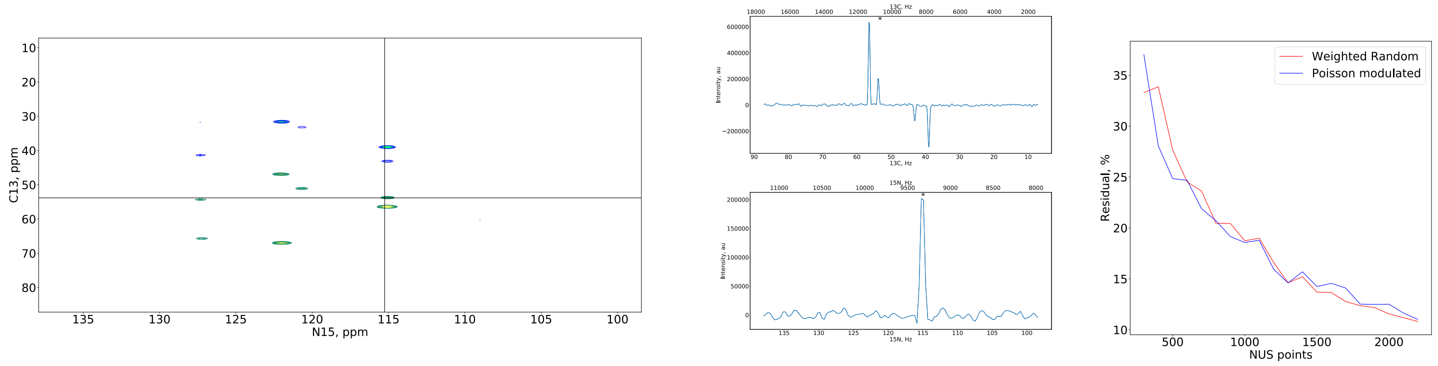

Peak272

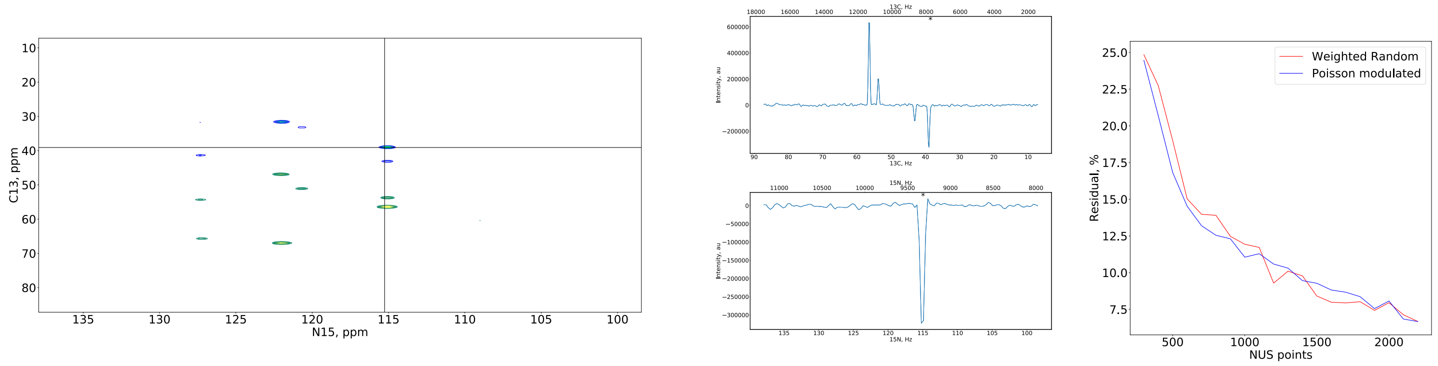

Peak273

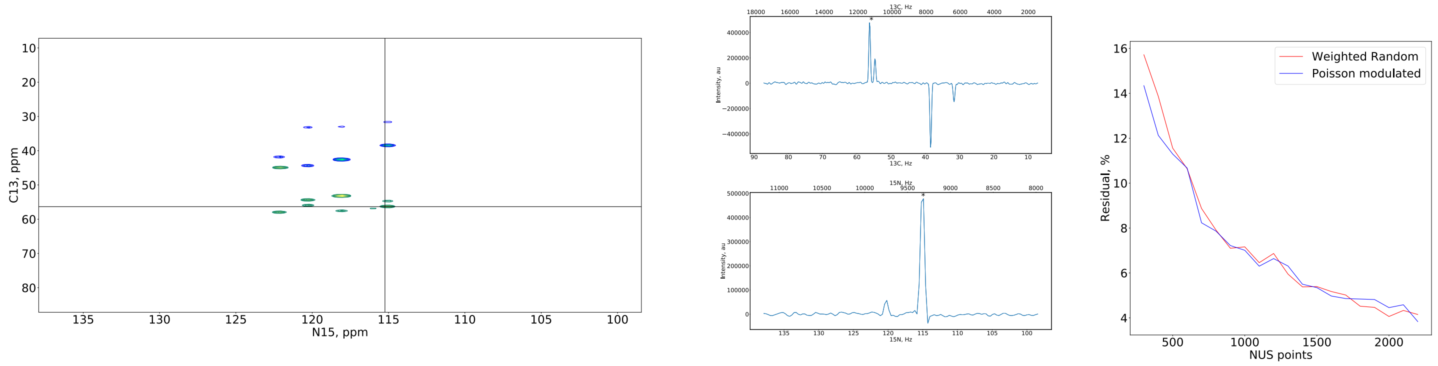

Peak274

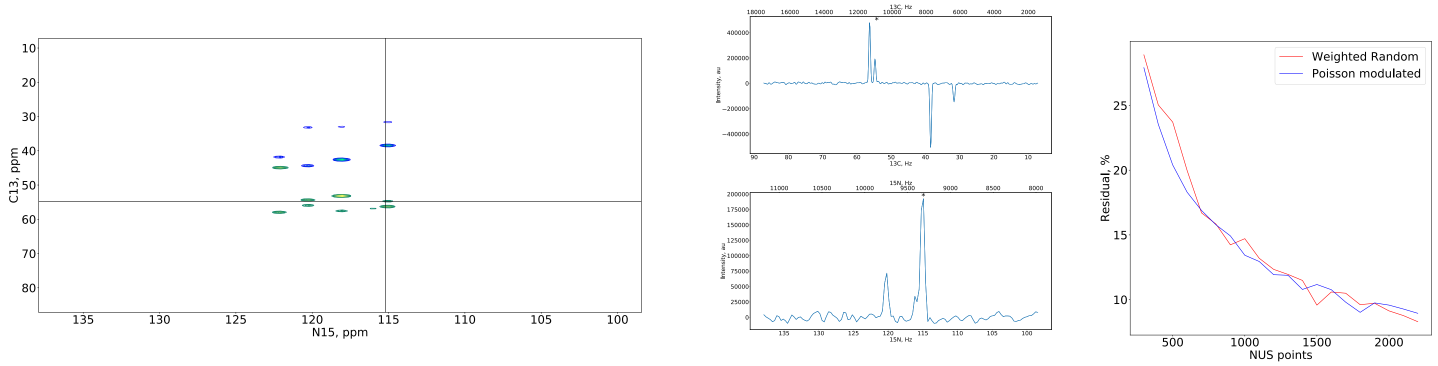

Peak275

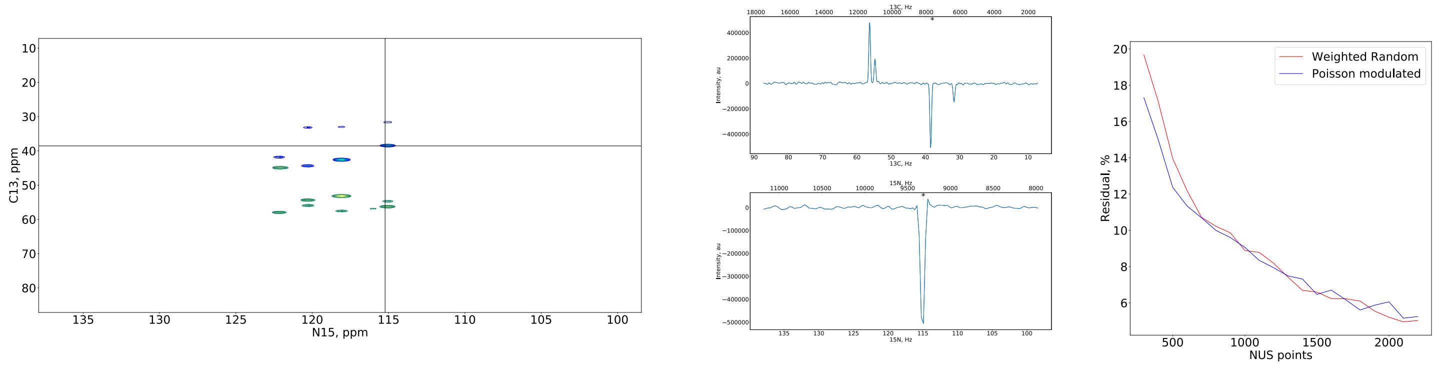

Peak276

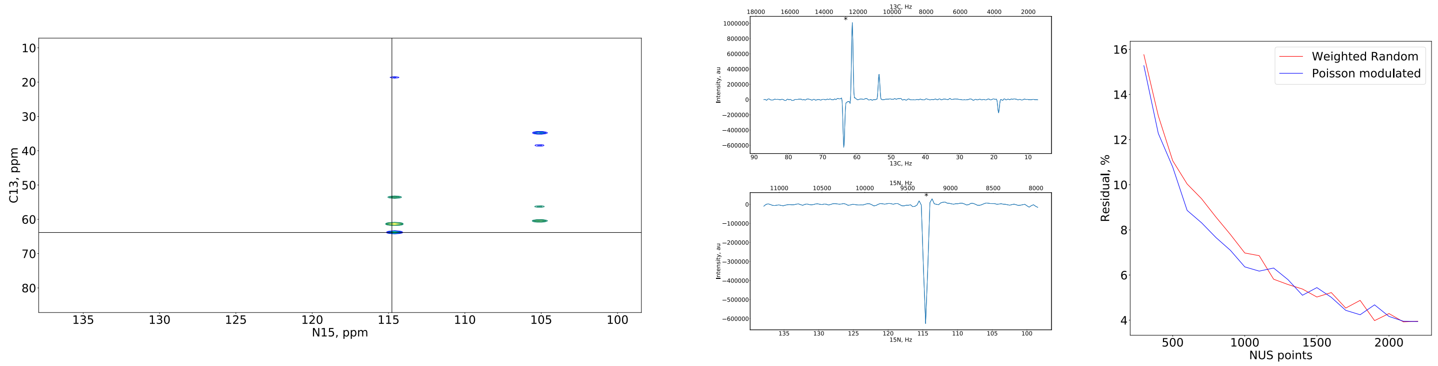

Peak277

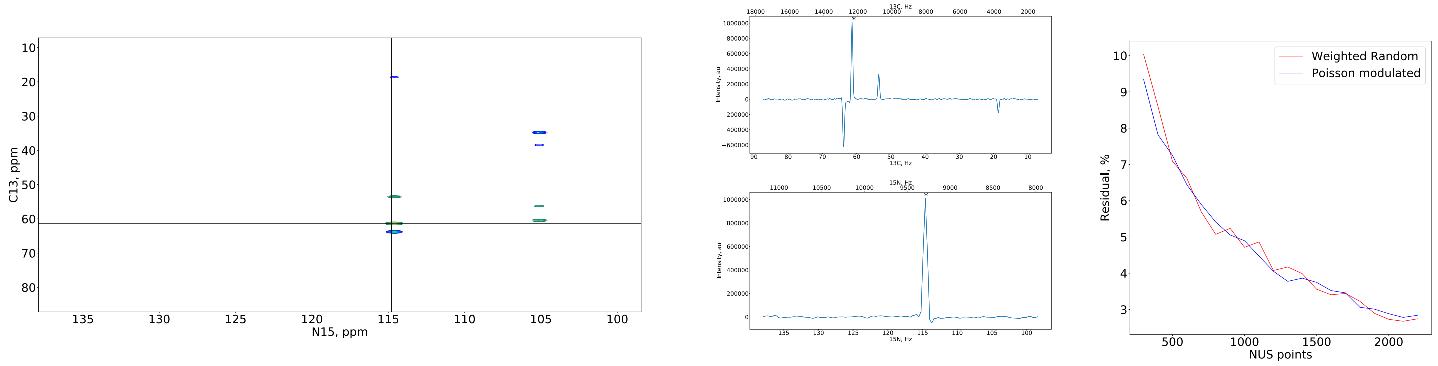

Peak278

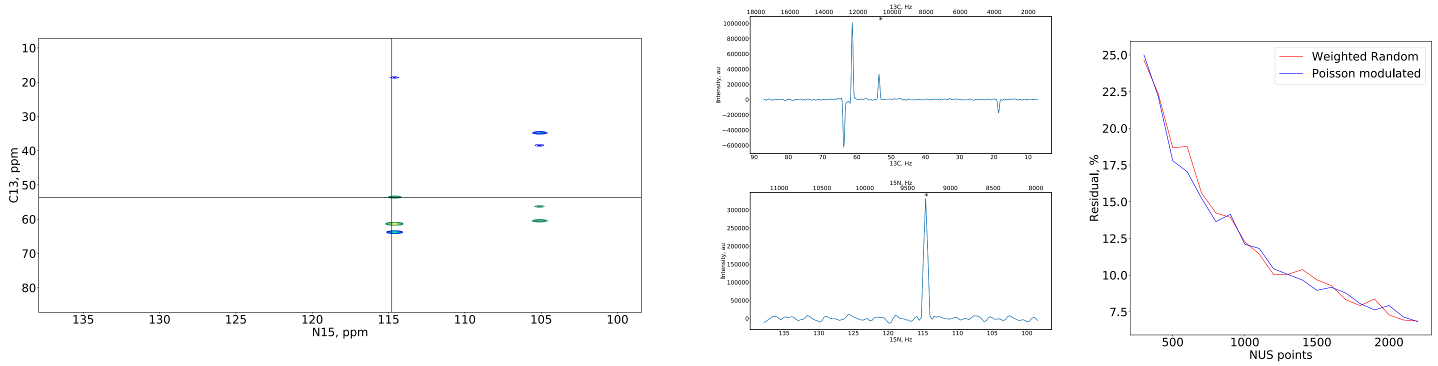

Peak279

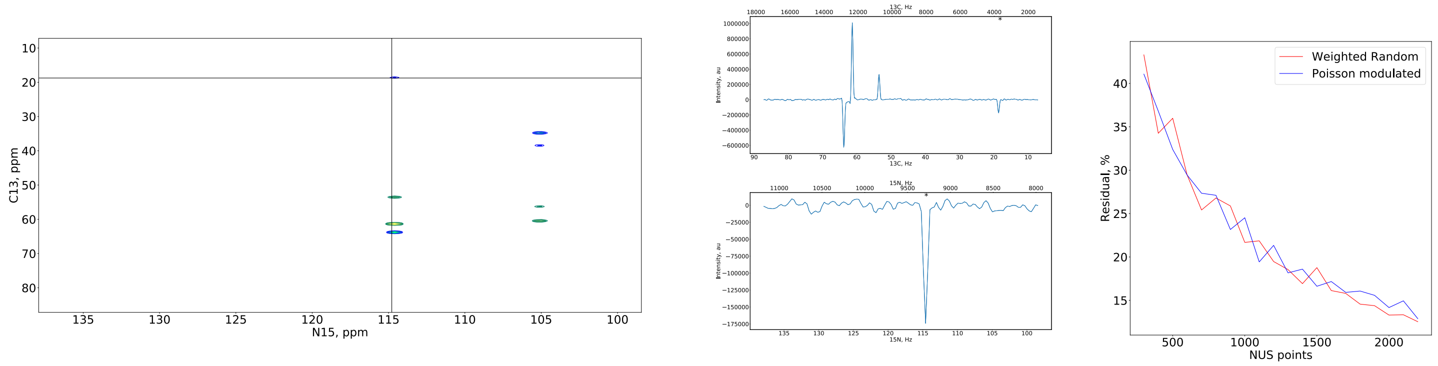

Peak280

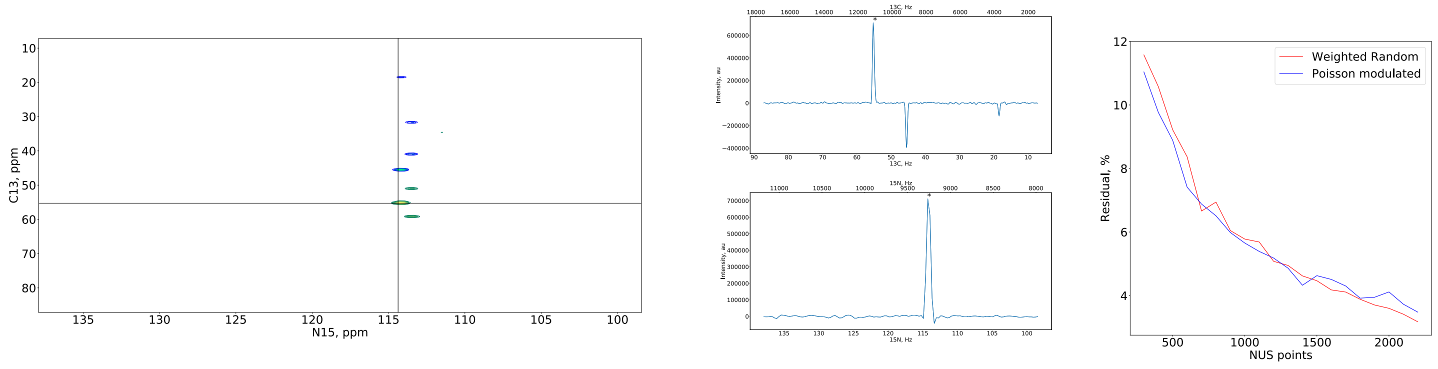

Peak281

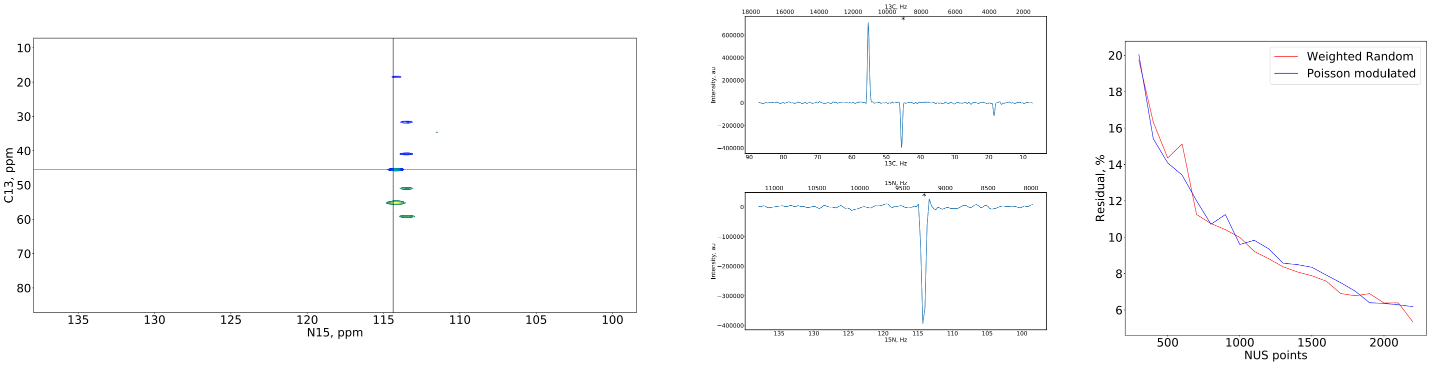

Peak282

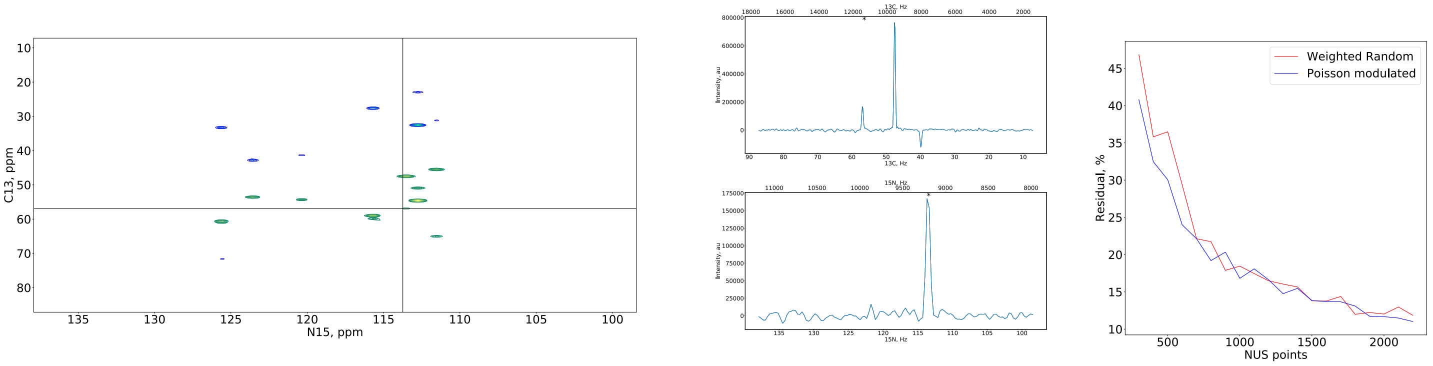

Peak283

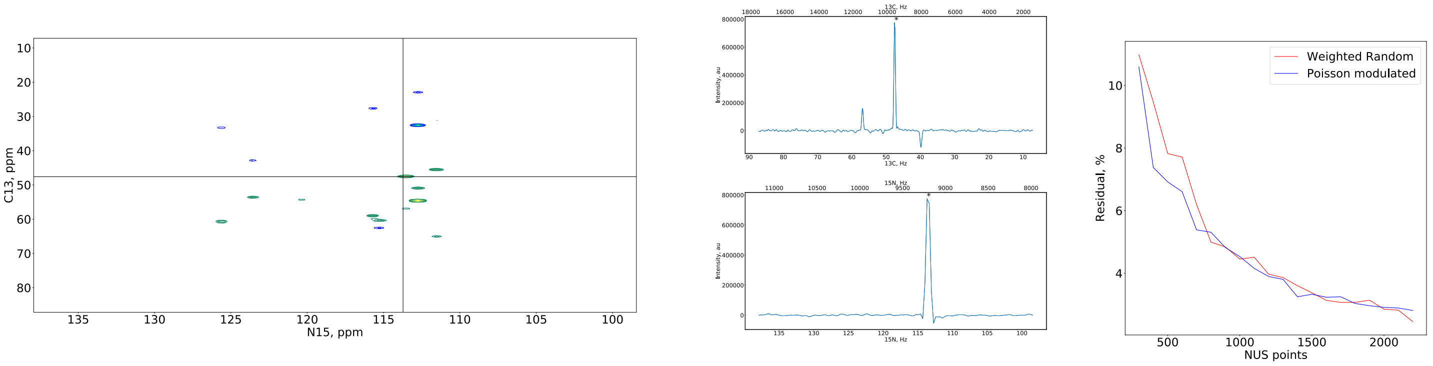

Peak284

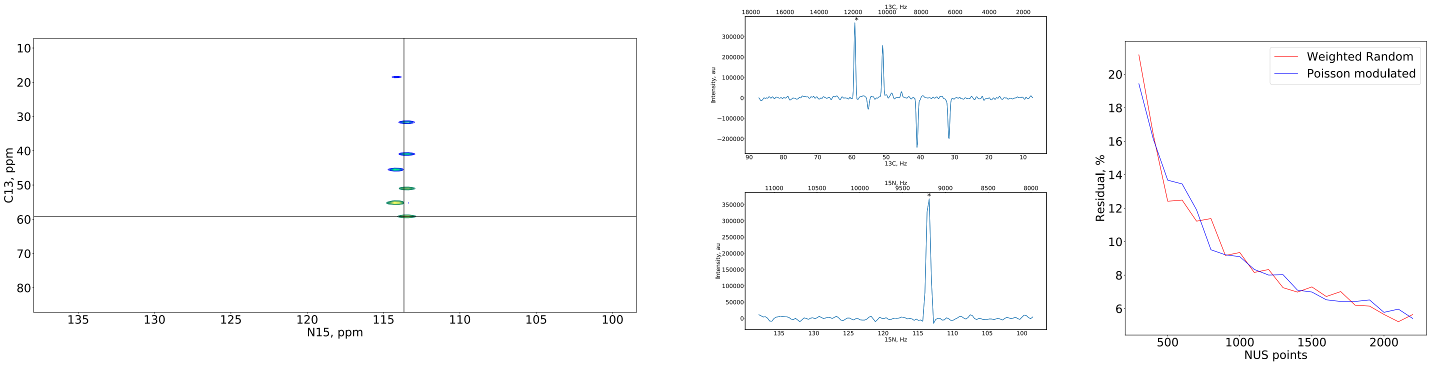

Peak285

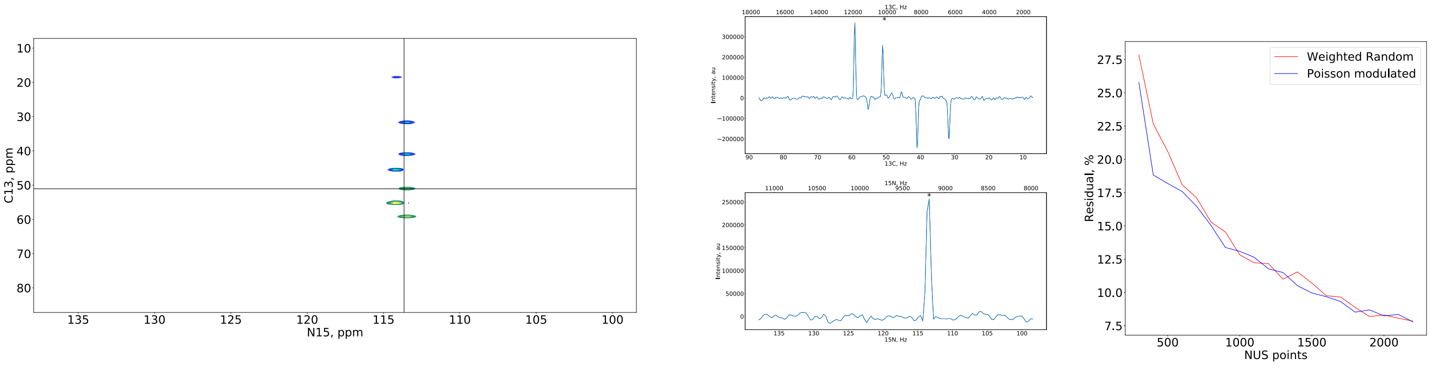

Peak286

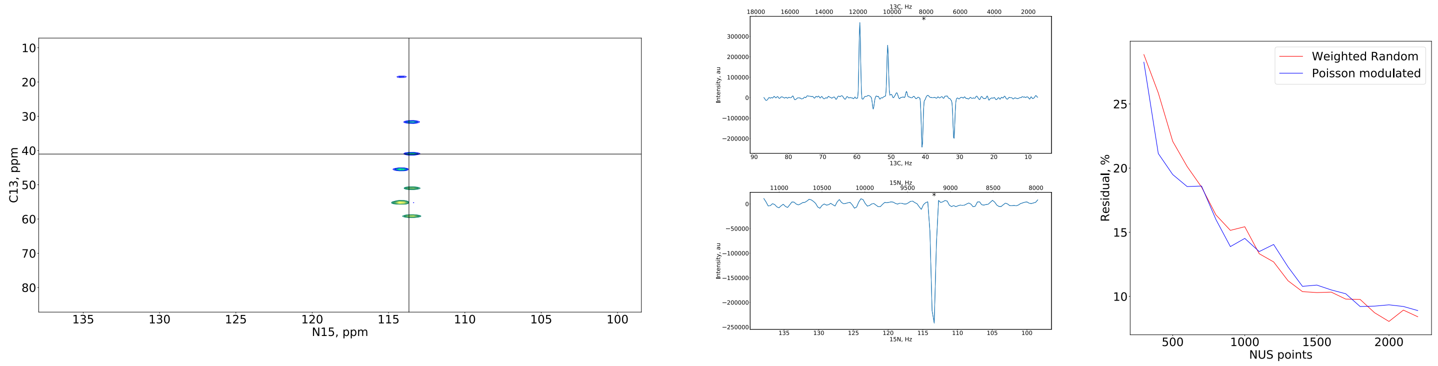

Peak287

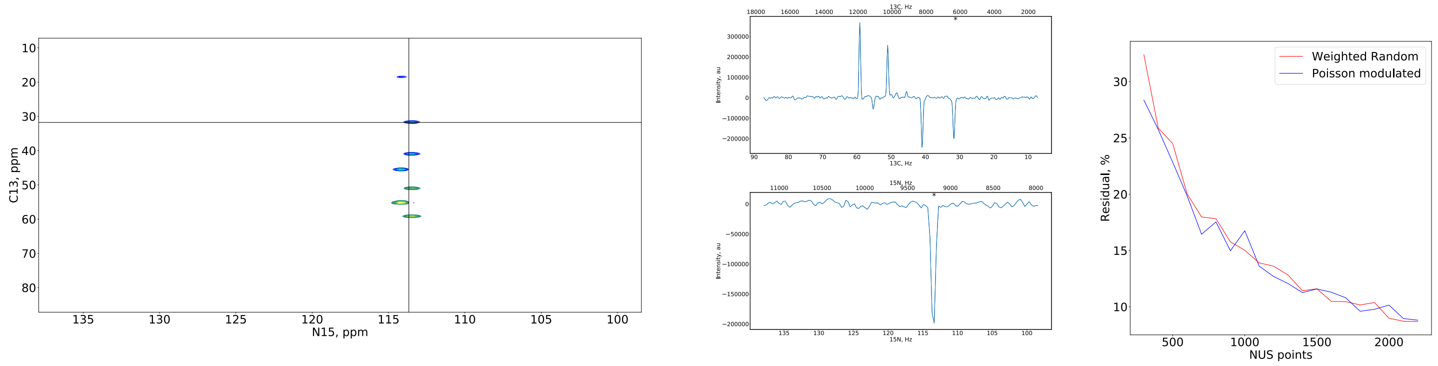

Peak288

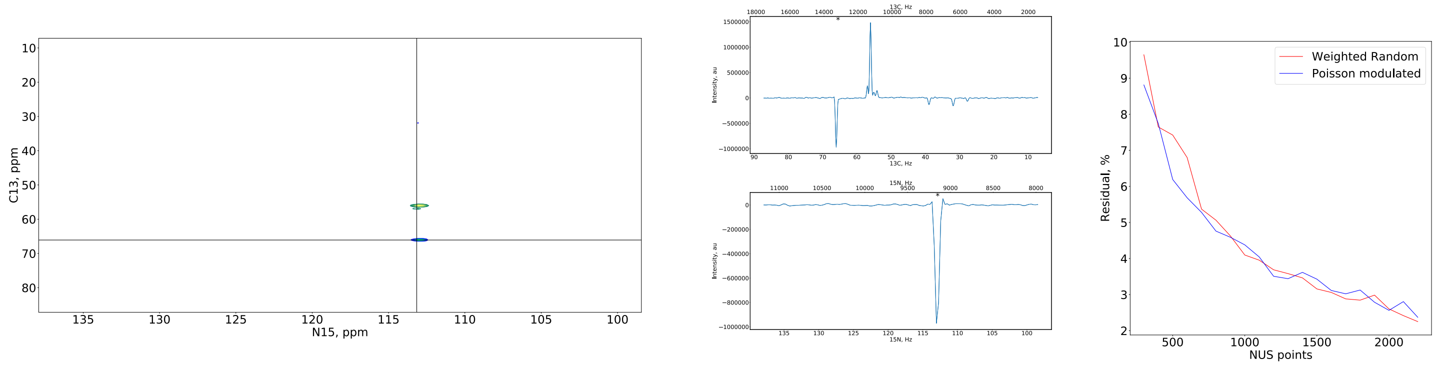

Peak289

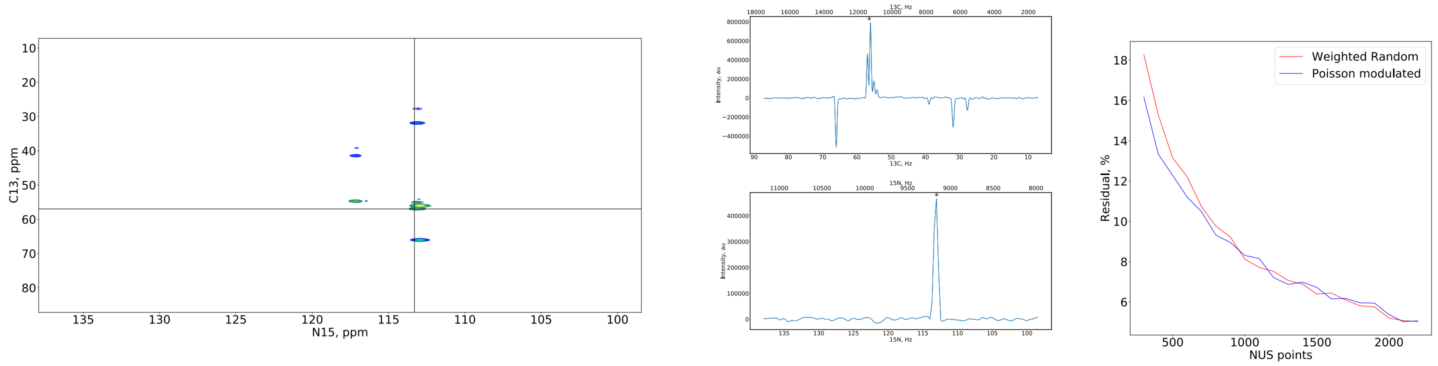

Peak290

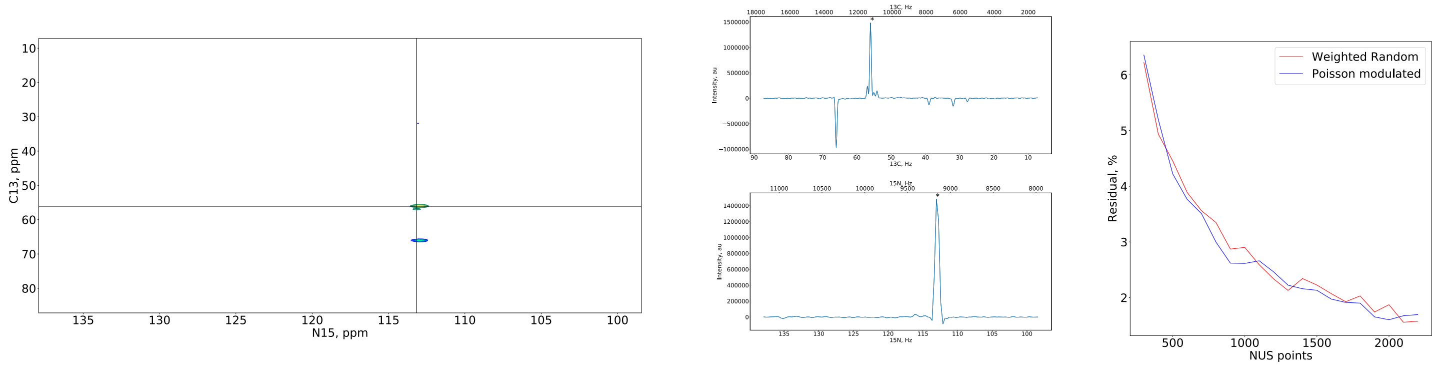

Peak291

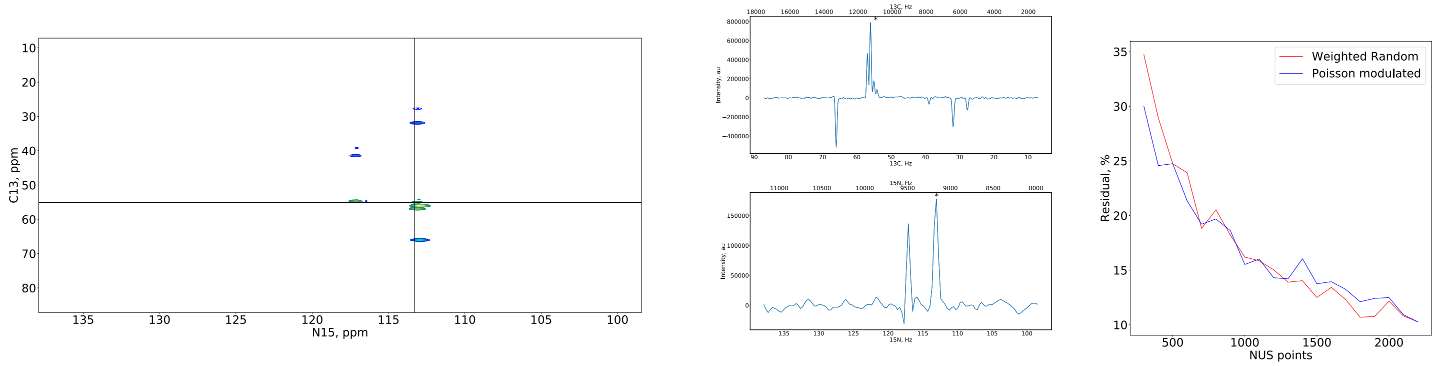

Peak292

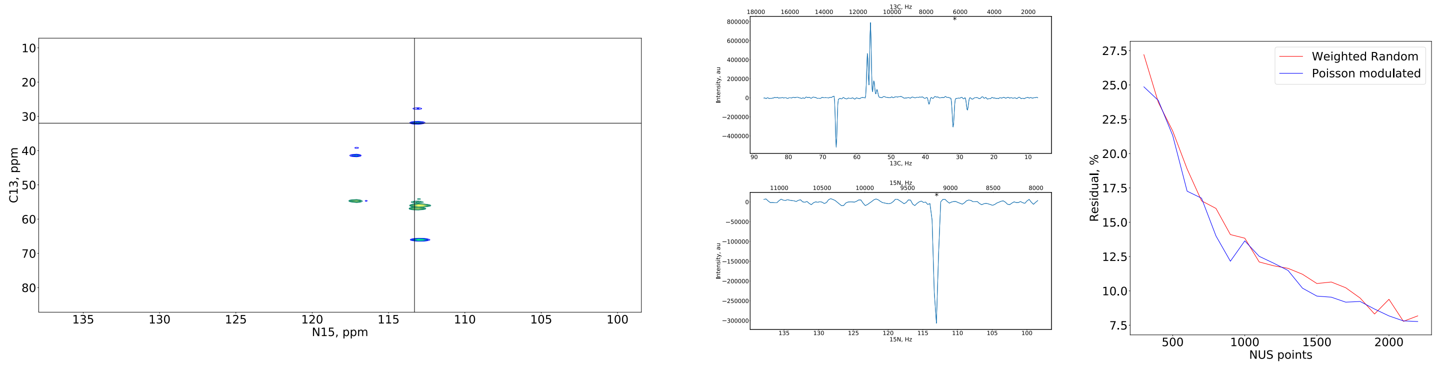

Peak293

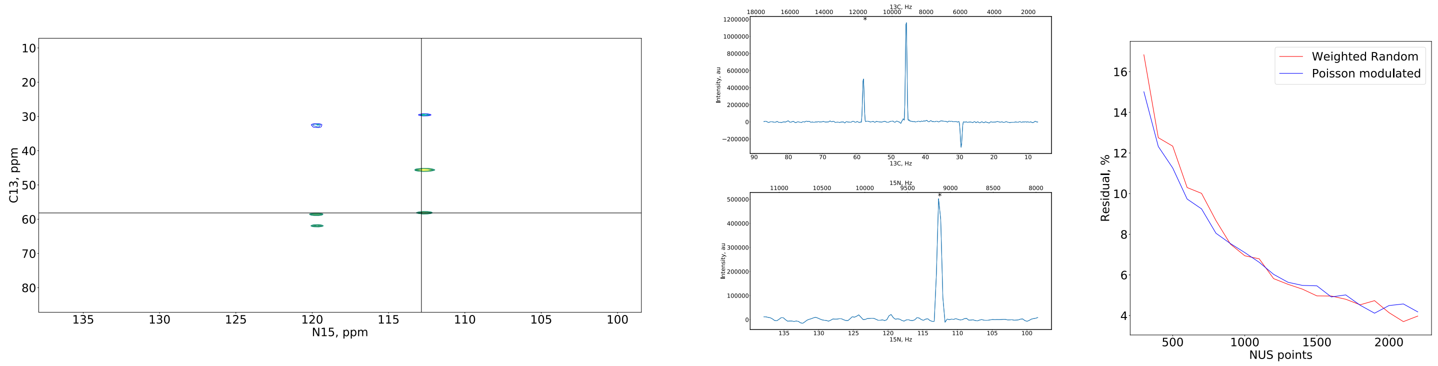

Peak294

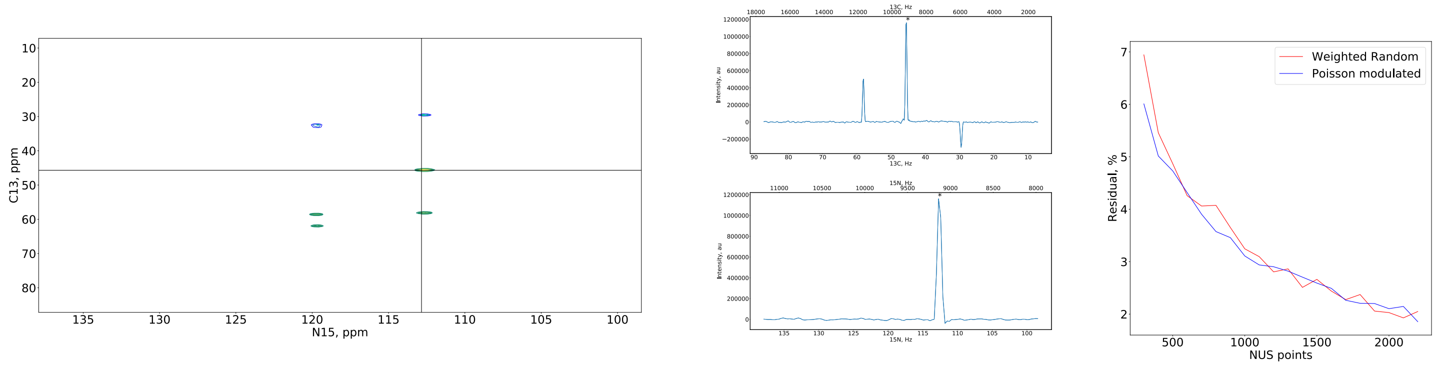

Peak295

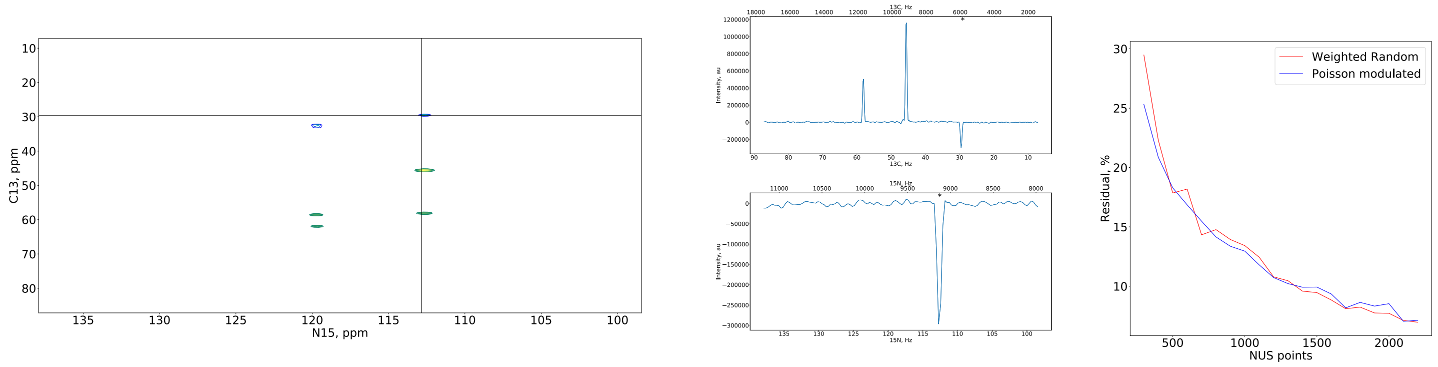

Peak296

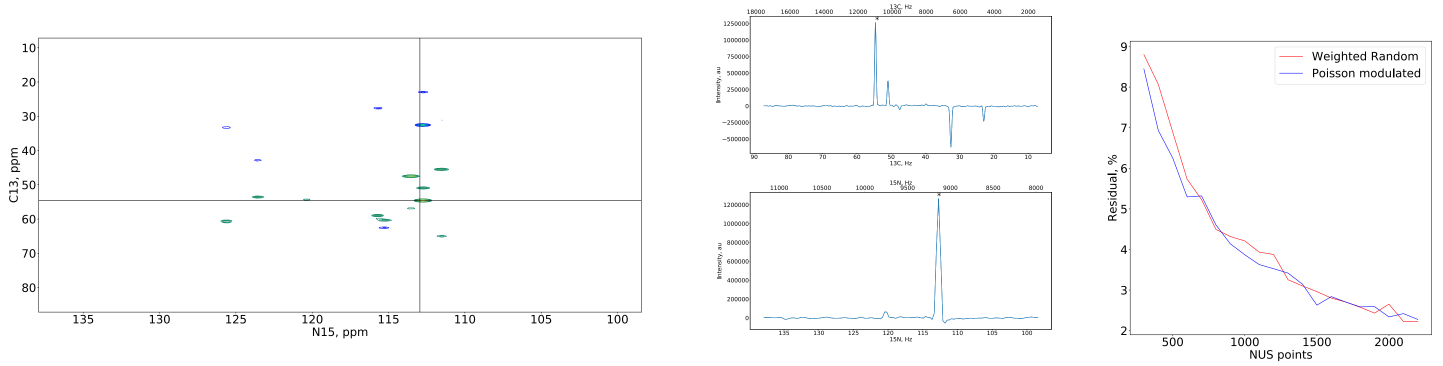

Peak297

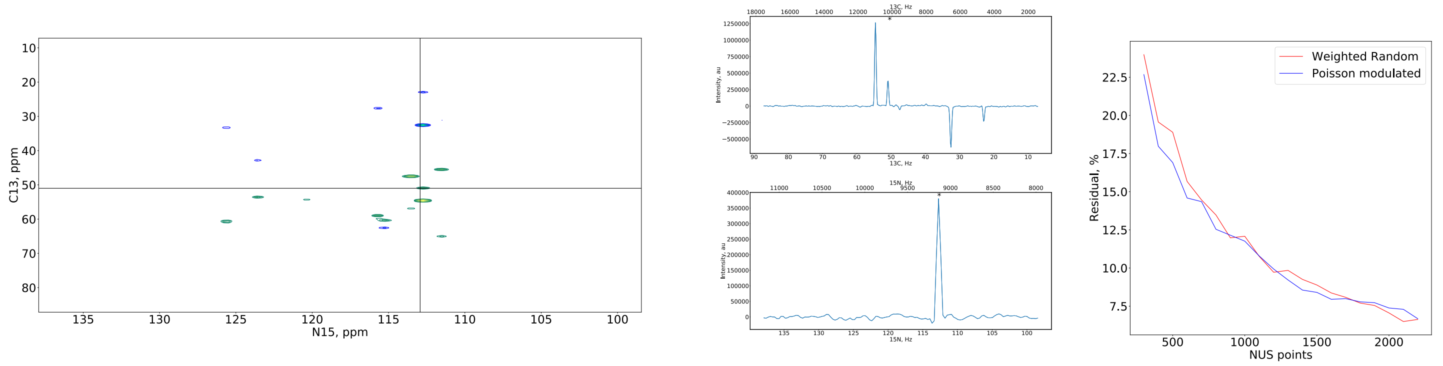

Peak298

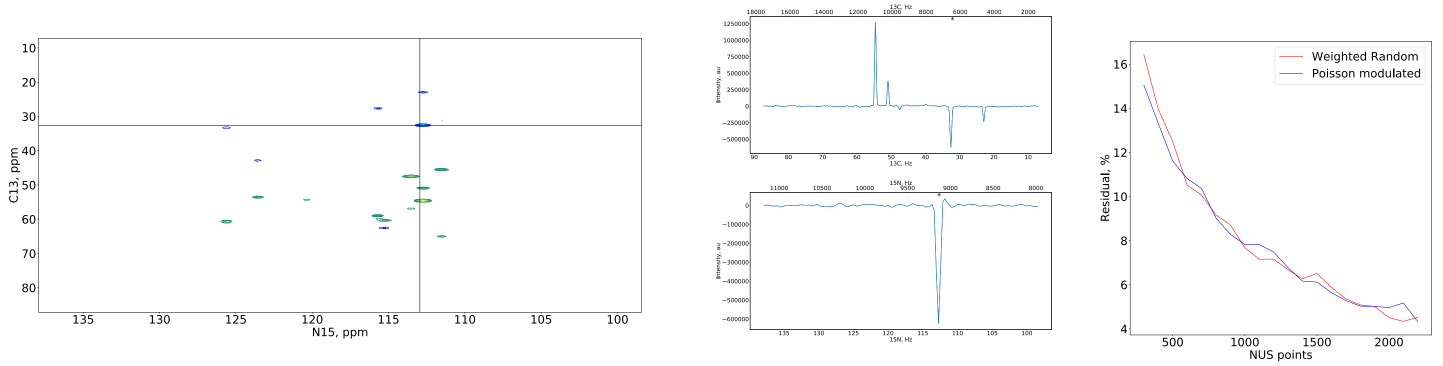

Peak299

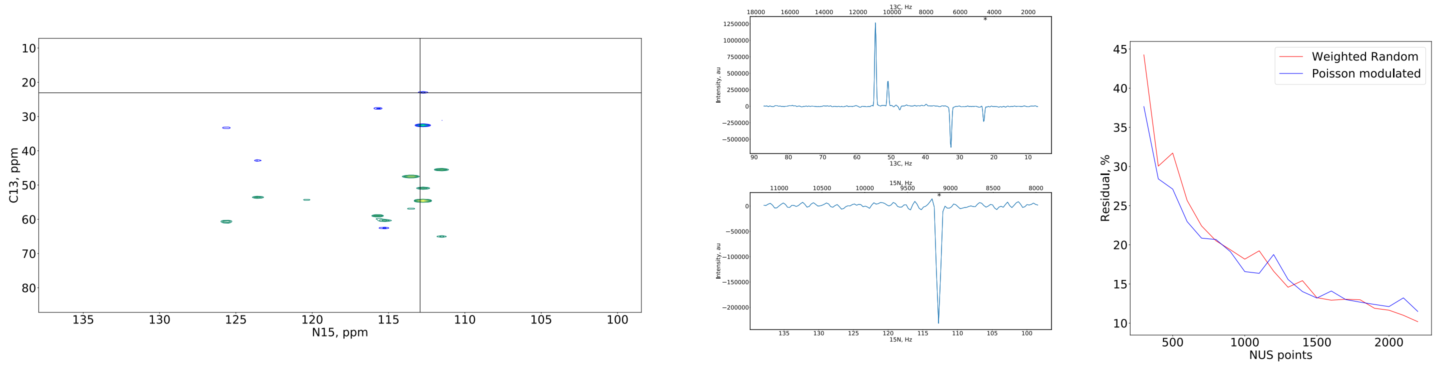

Peak300

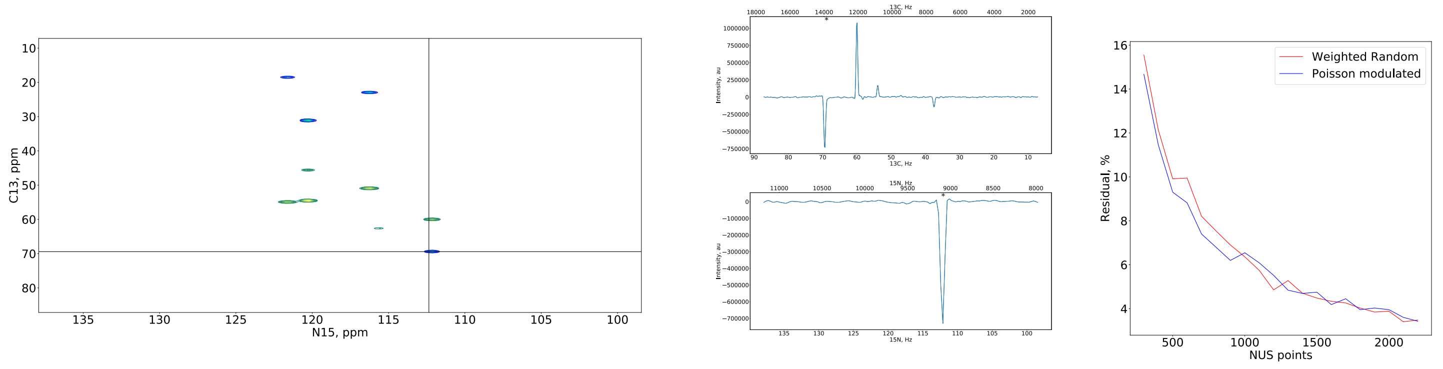

Peak301

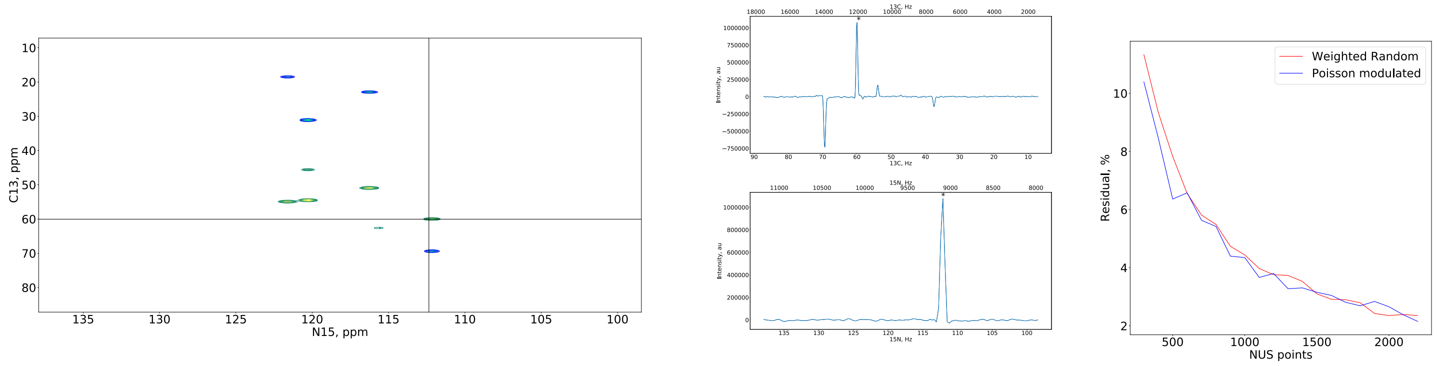

Peak302

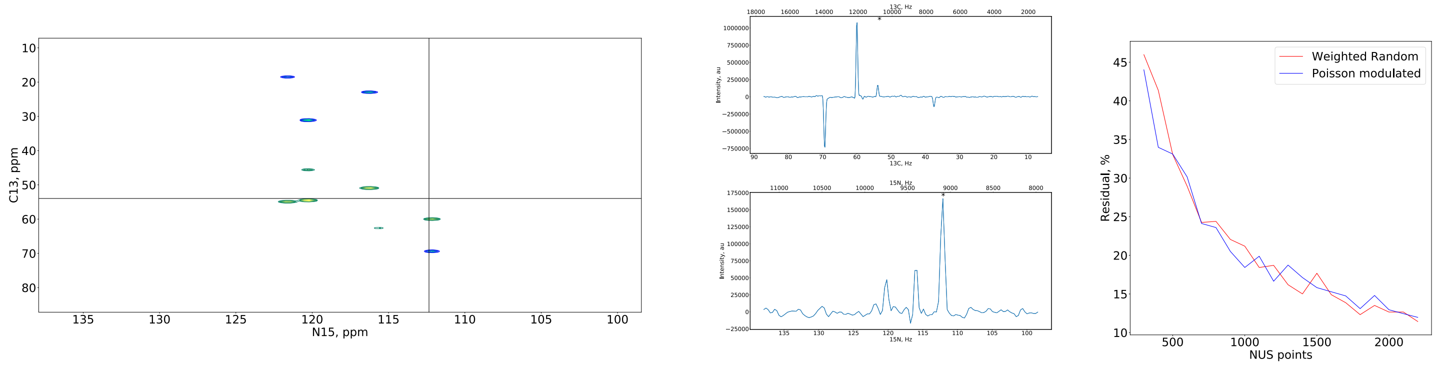

Peak303

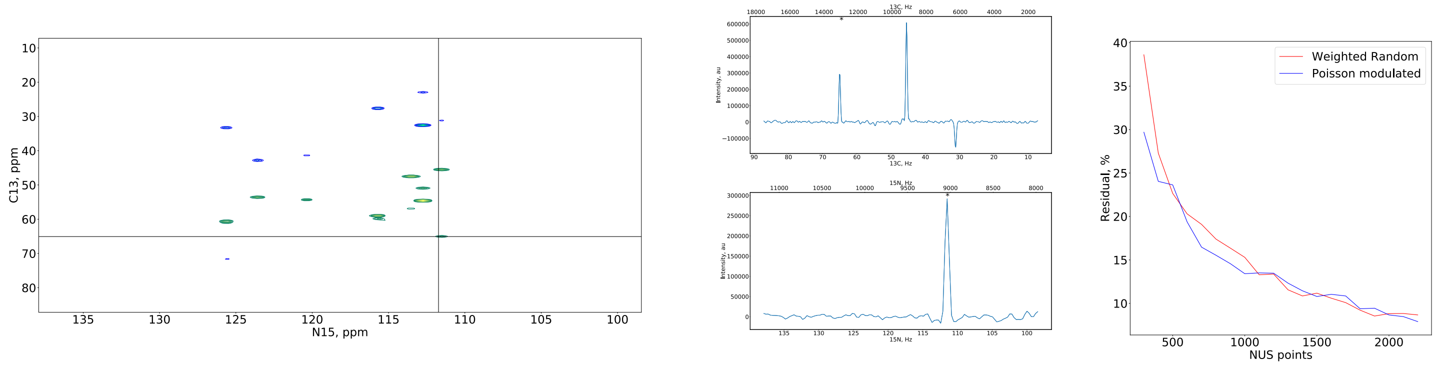

Peak304

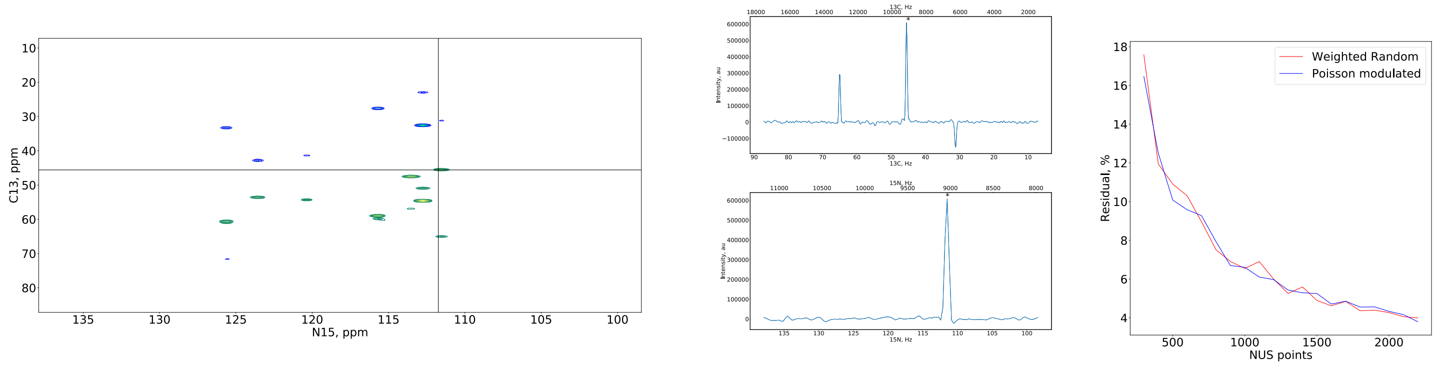

Peak305

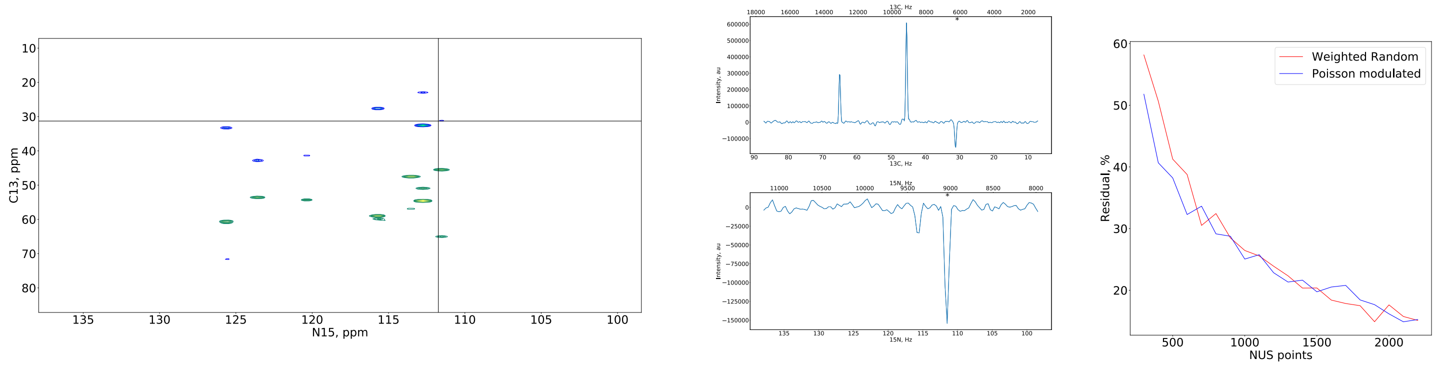

Peak306

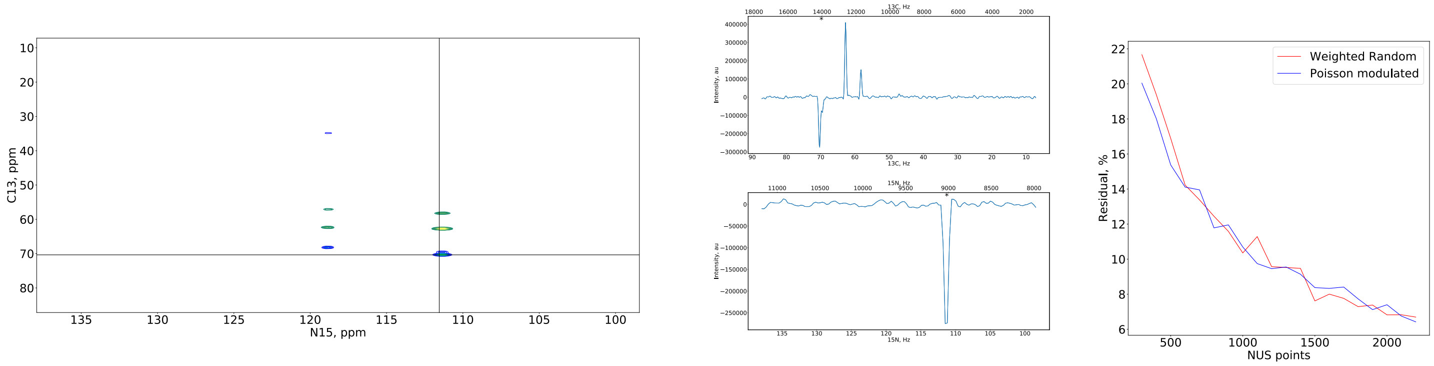

Peak307

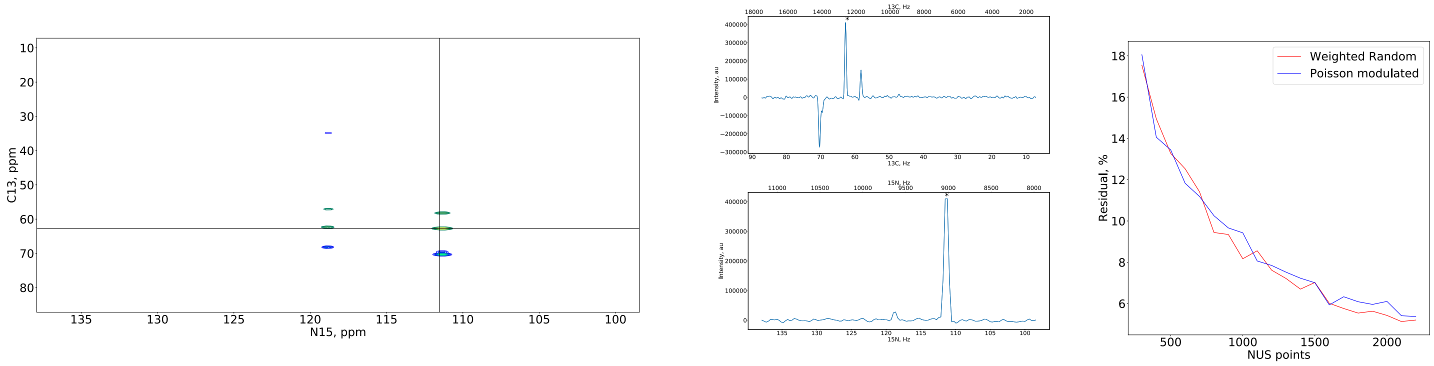

Peak308

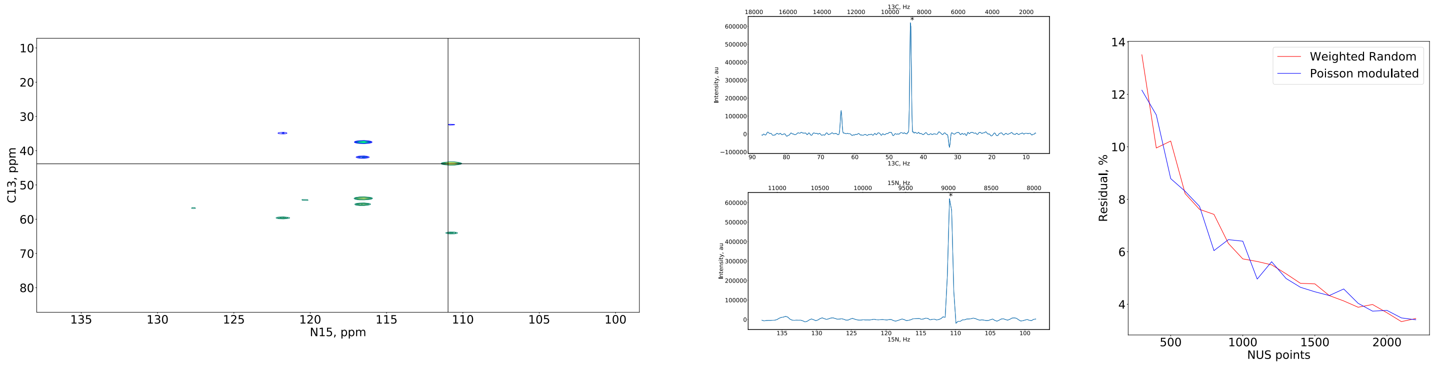

Peak309

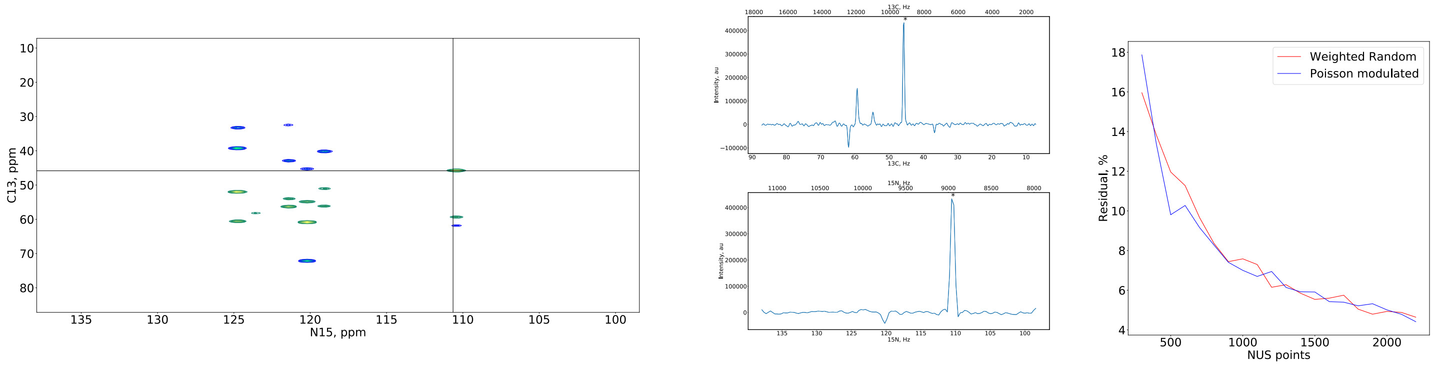

Peak310

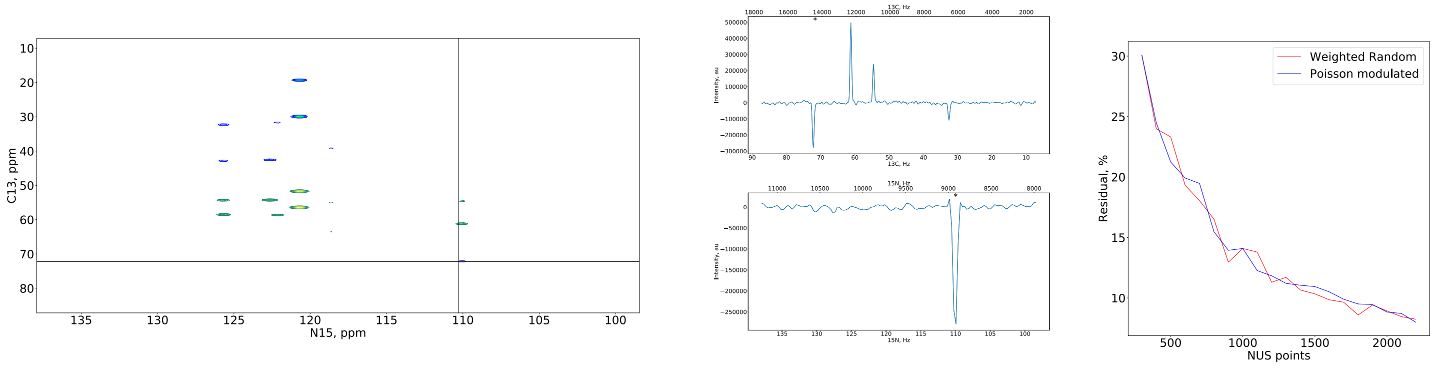

Peak311

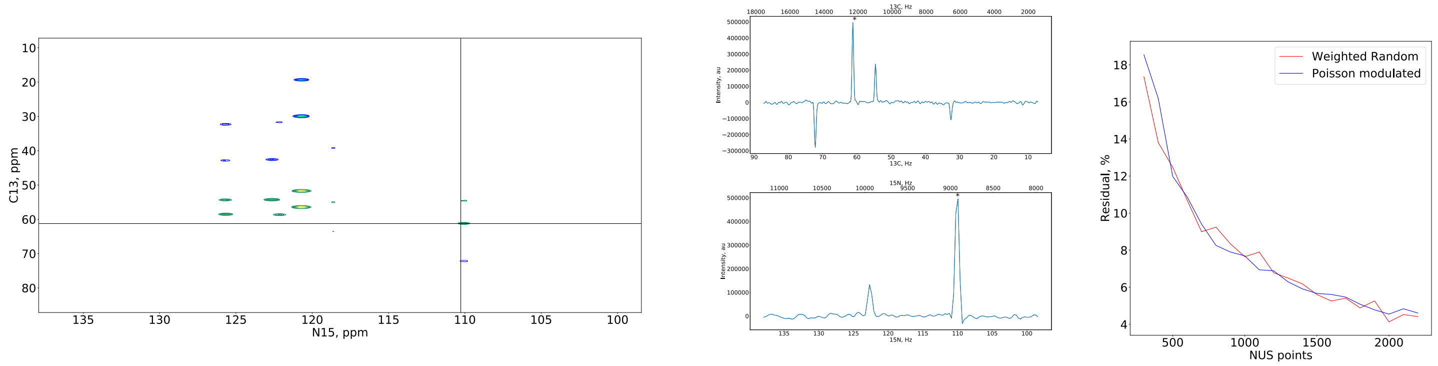

Peak312

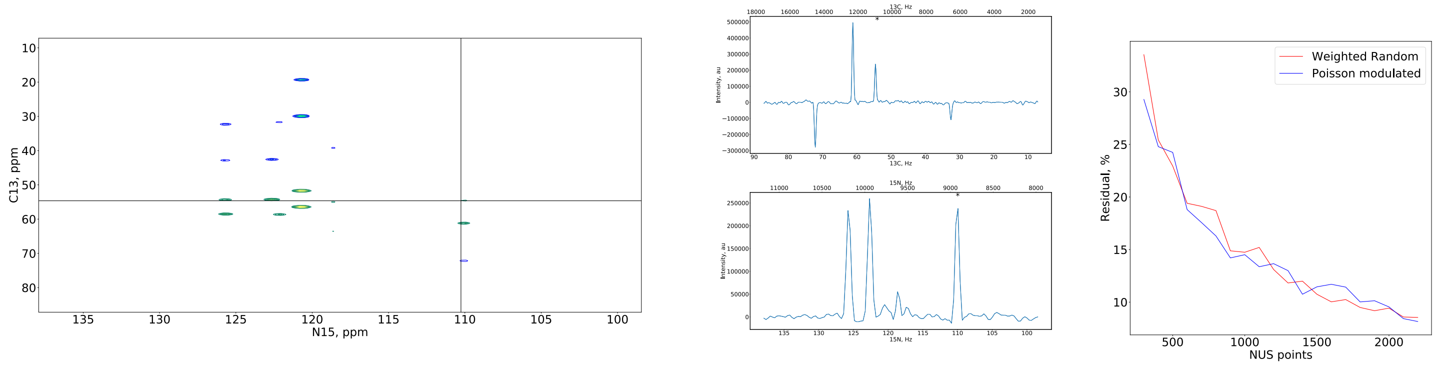

Peak313

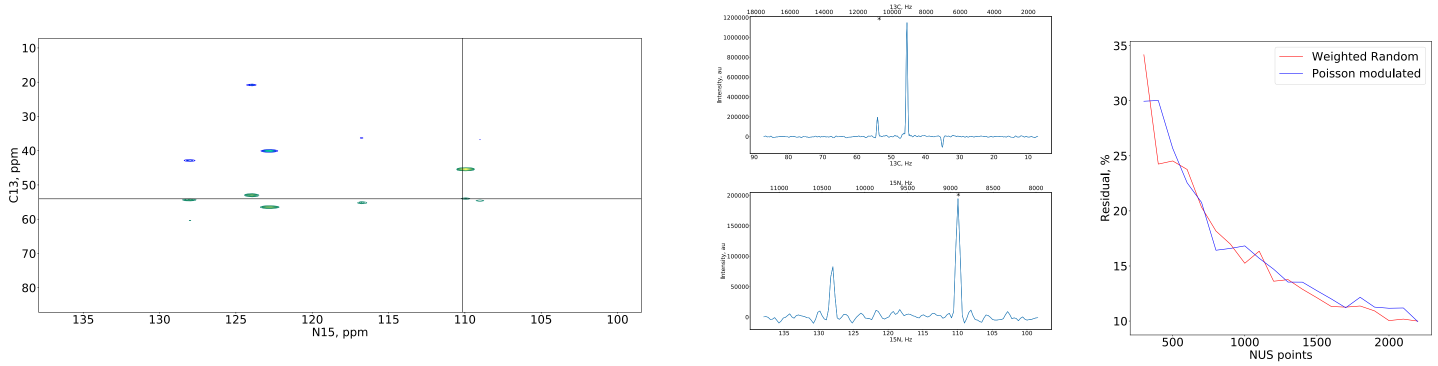

Peak314

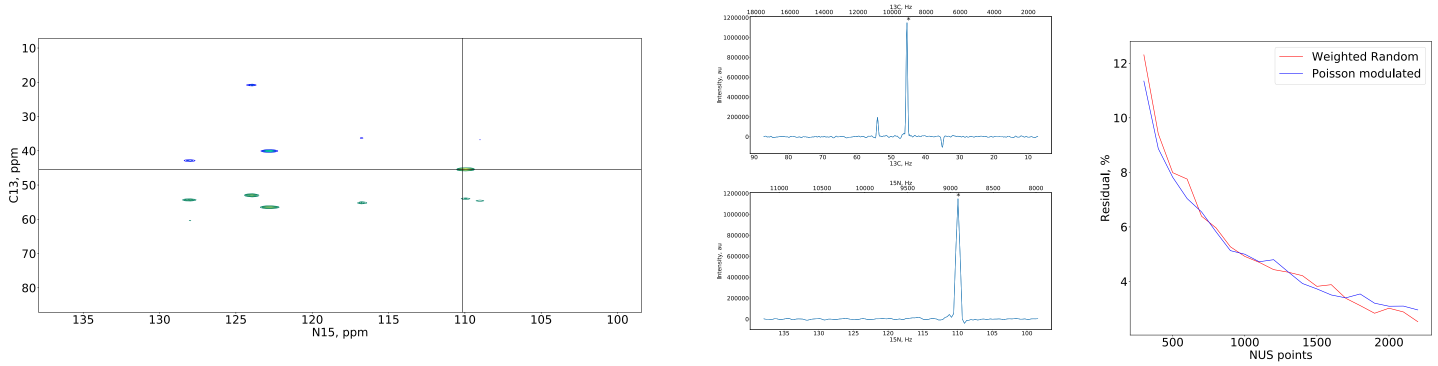

Peak315

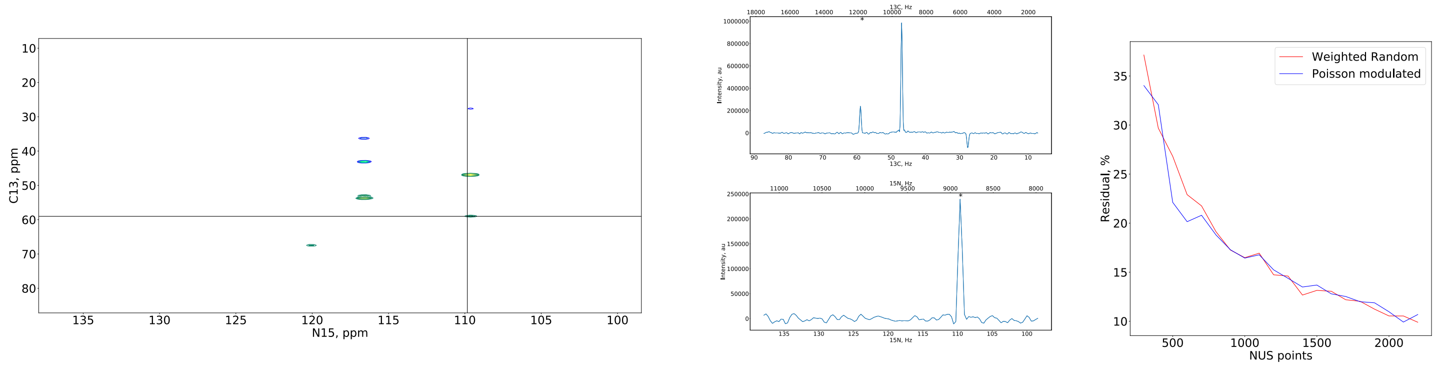

Peak316

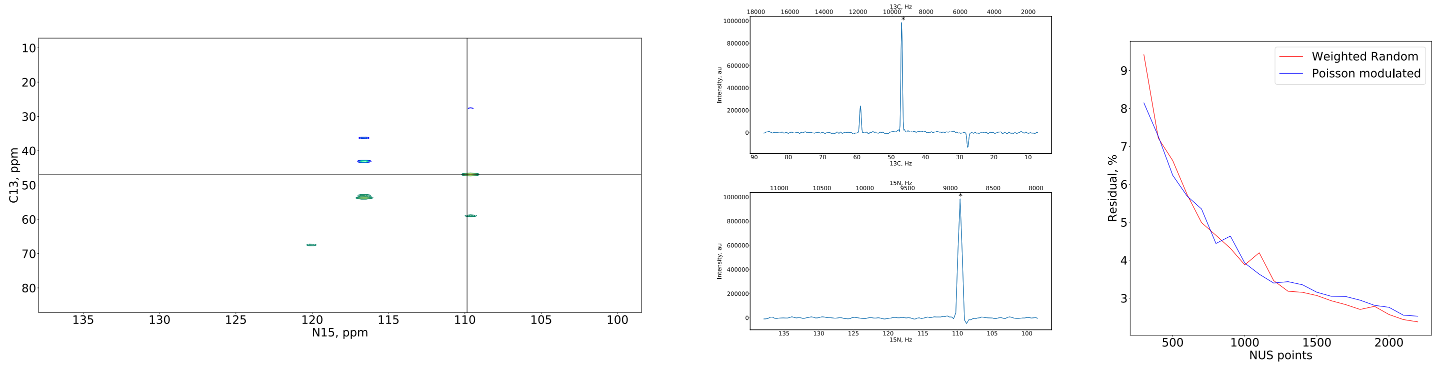

Peak317

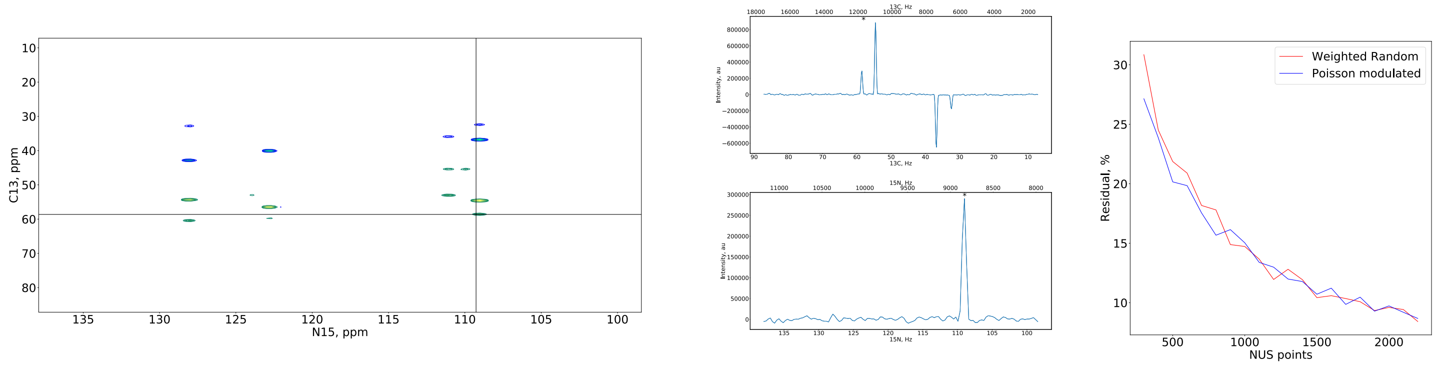

Peak318

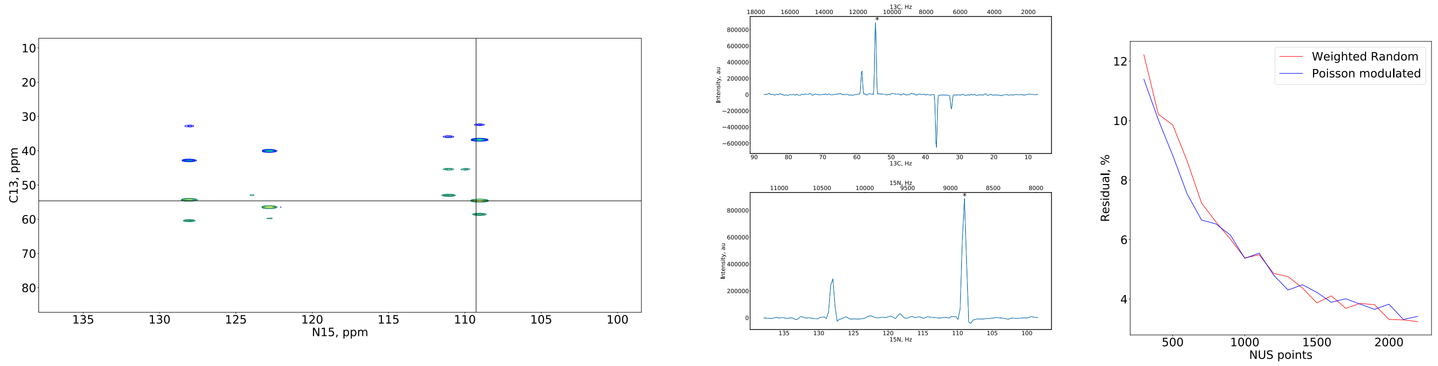

Peak319

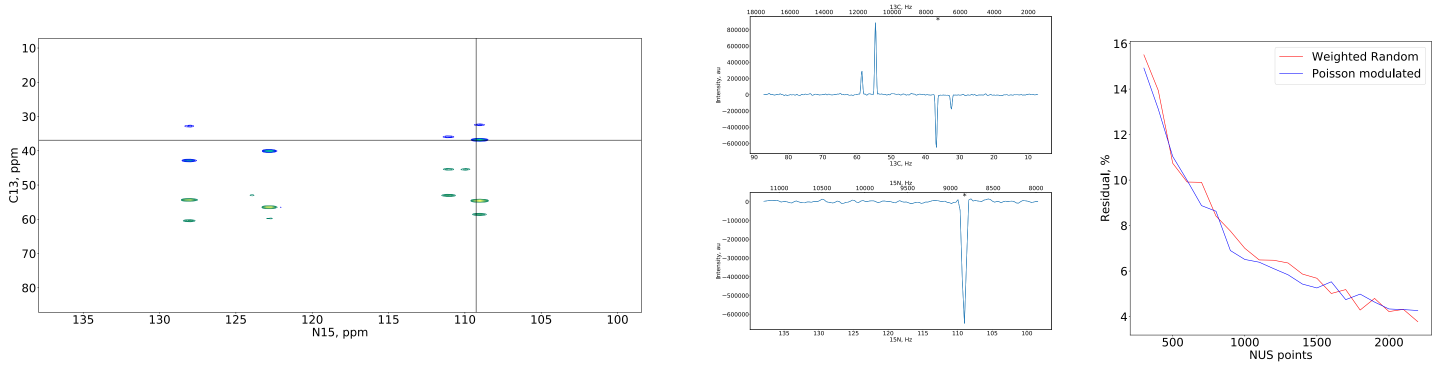

Peak320

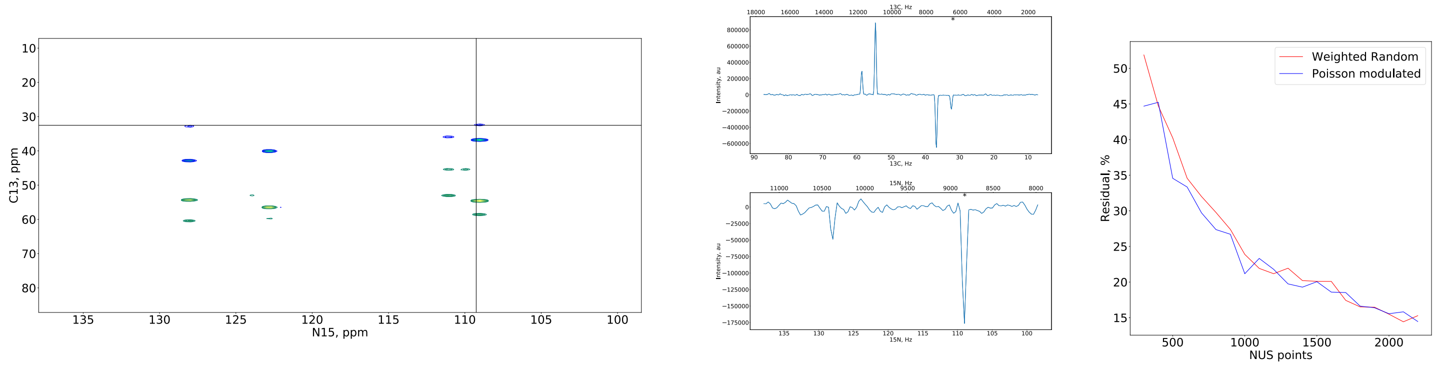

Peak321

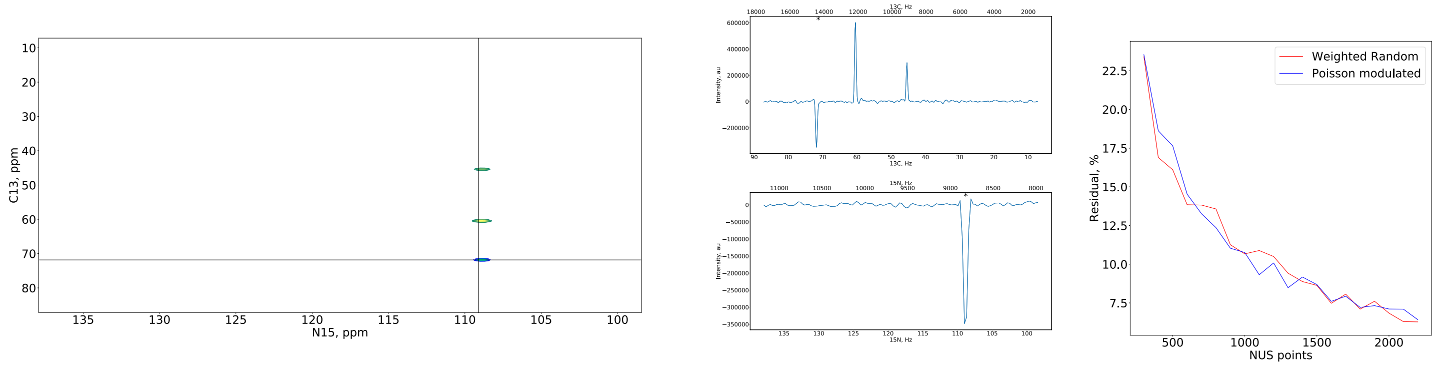

Peak322

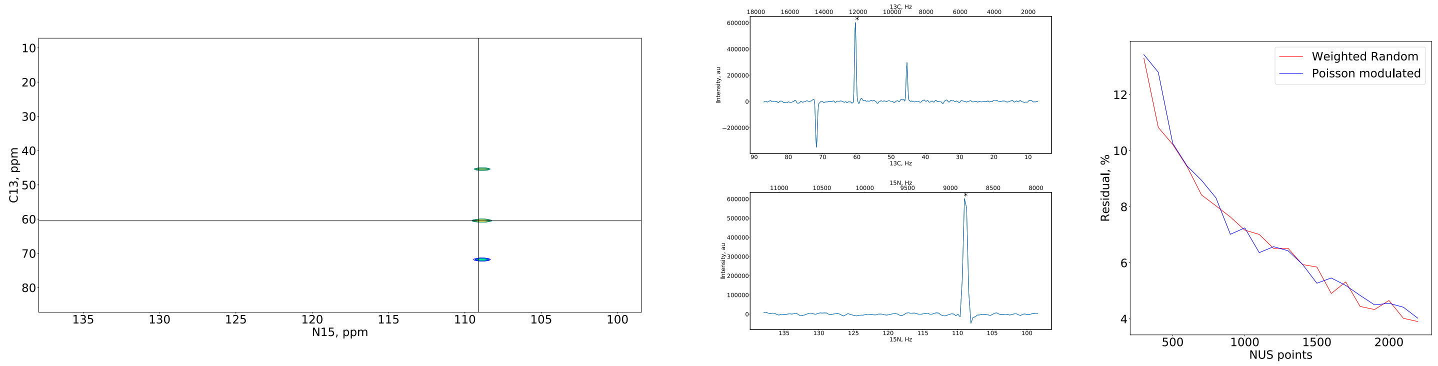

Peak323

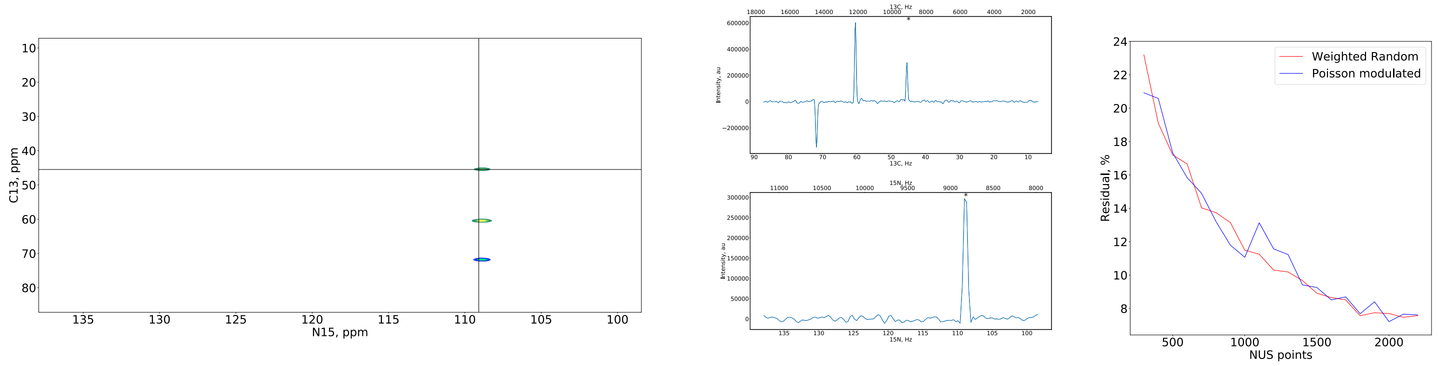

Peak324

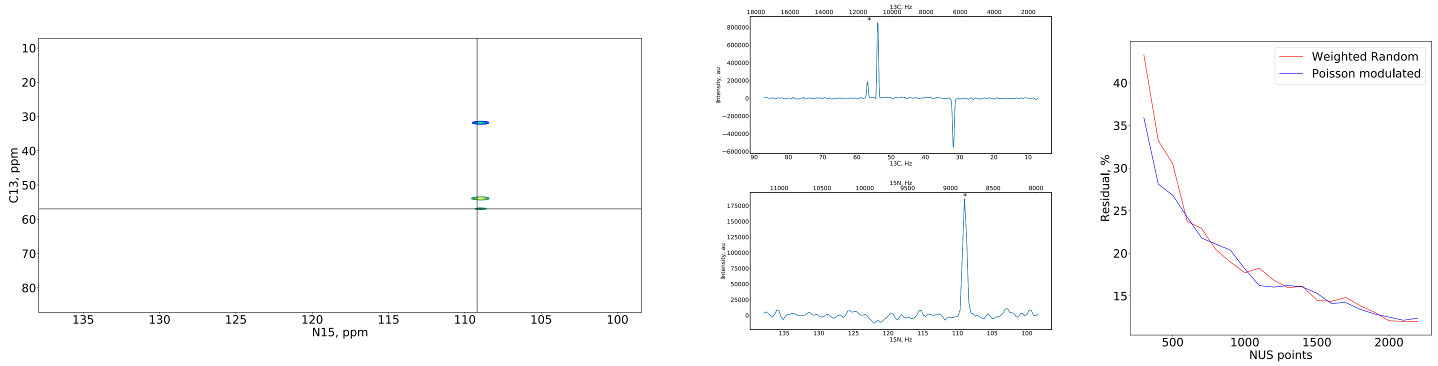

Peak325

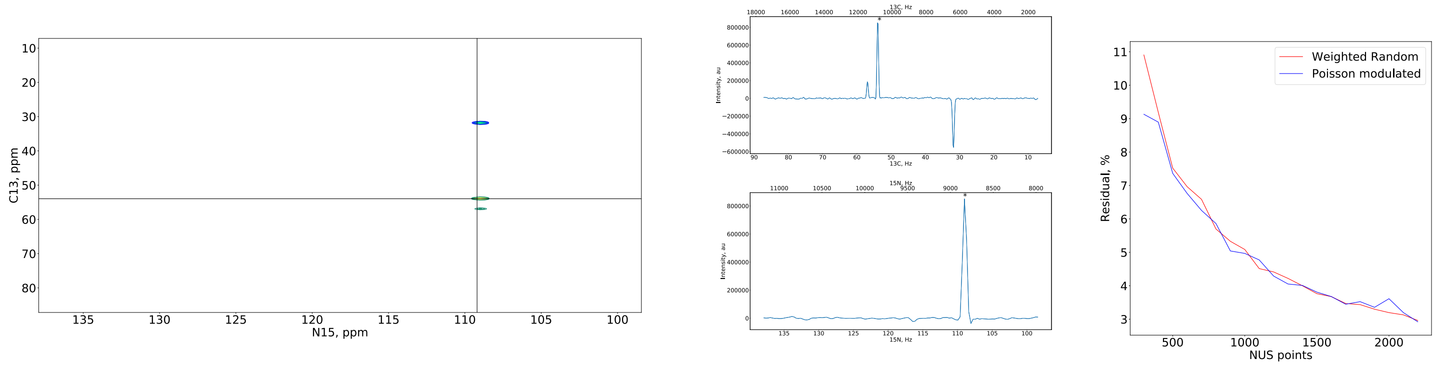

Peak326

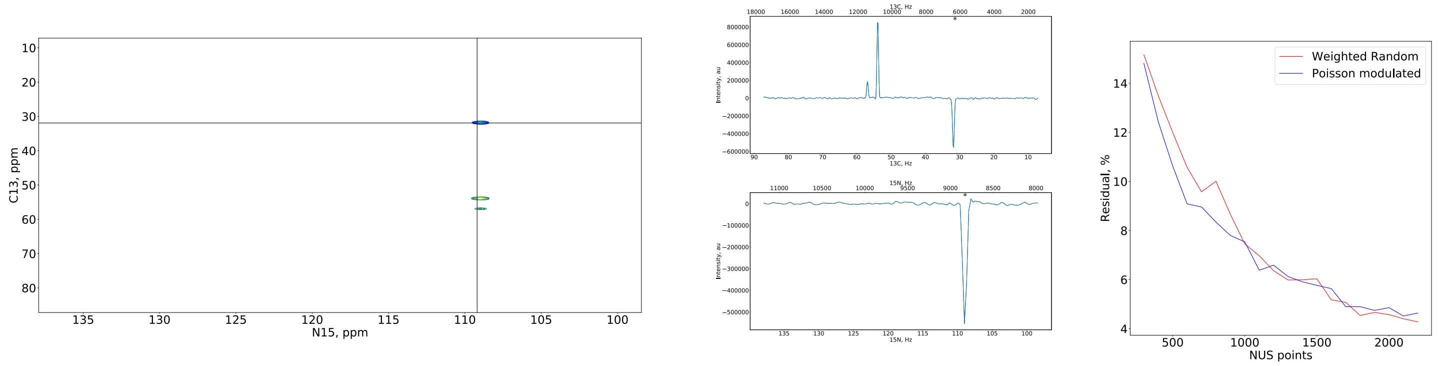

Peak327

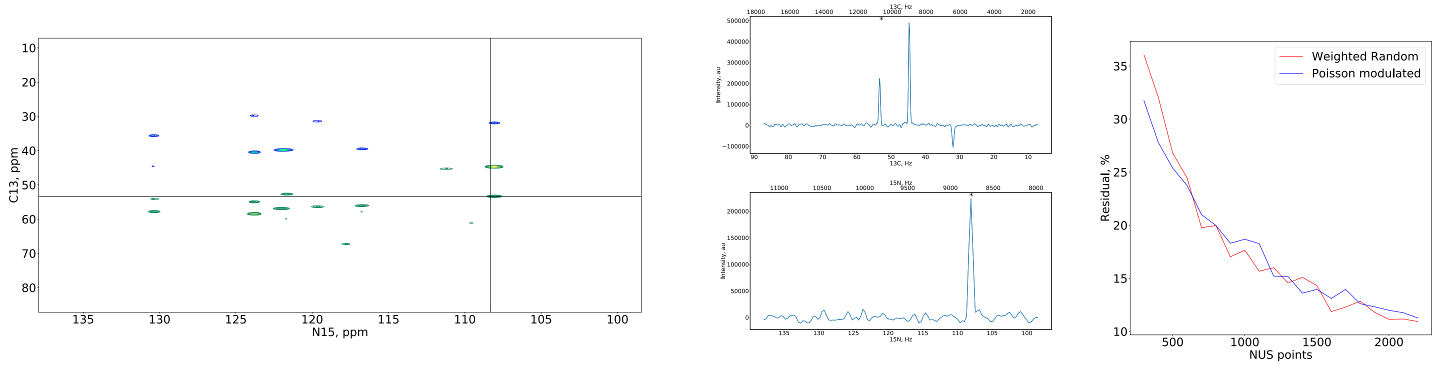

Peak328

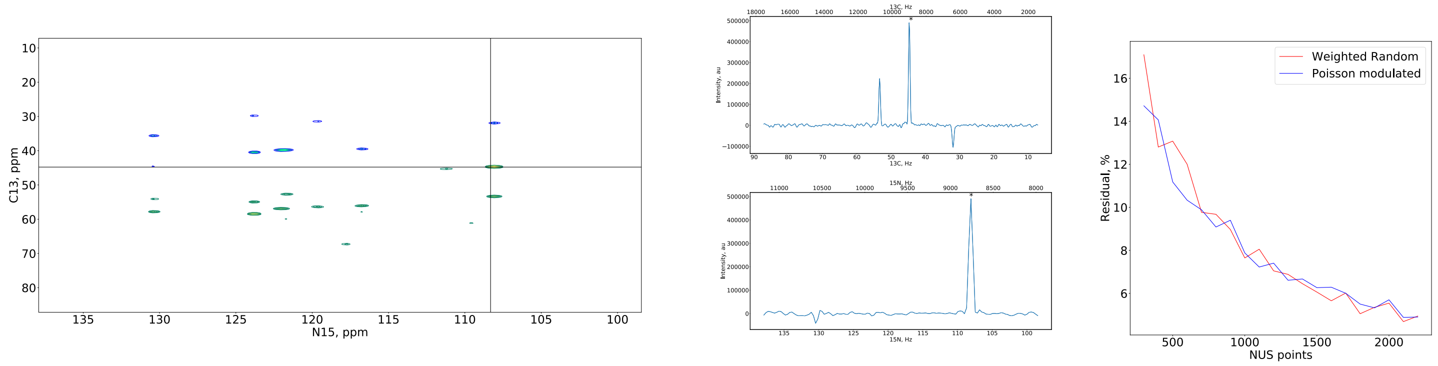

Peak329

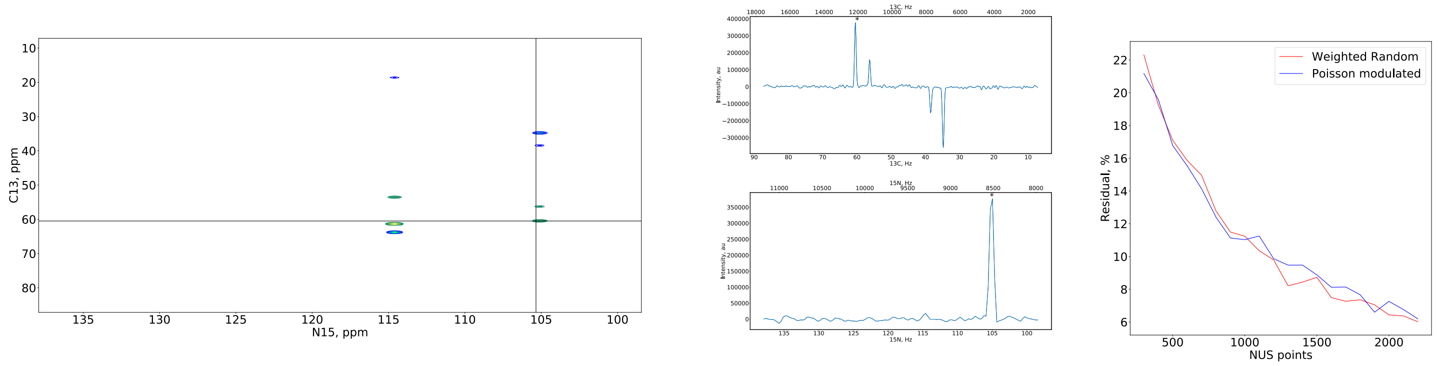

Peak330

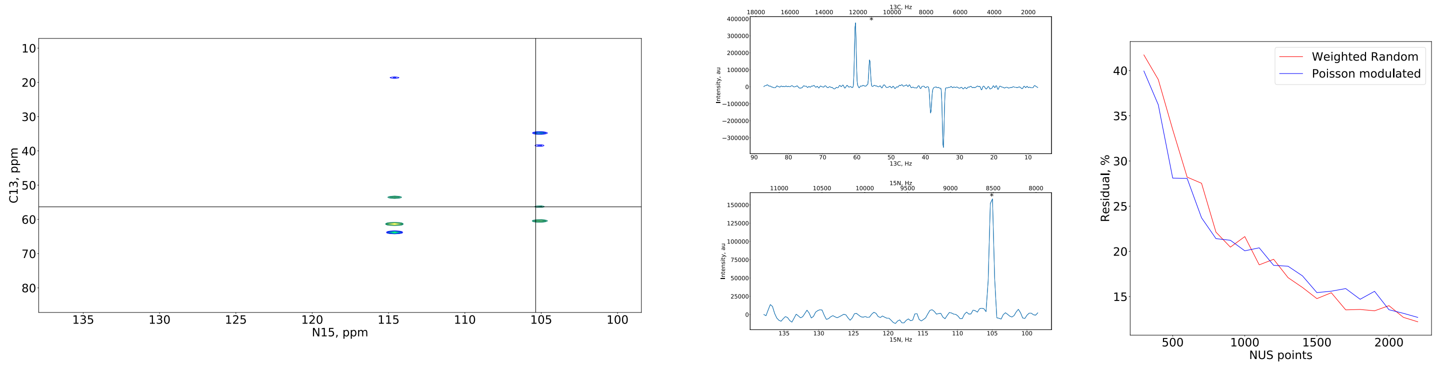

Peak331

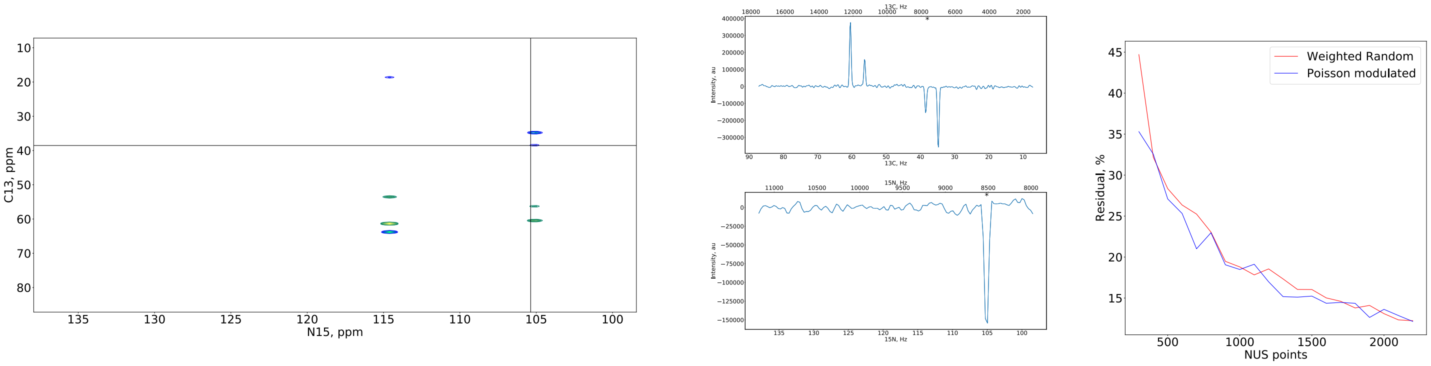

Peak332

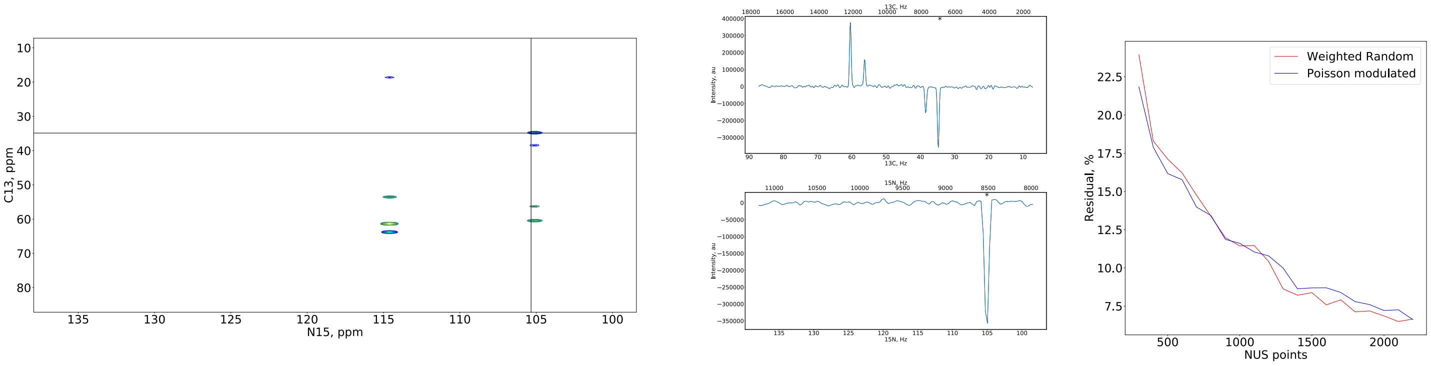

Peak333

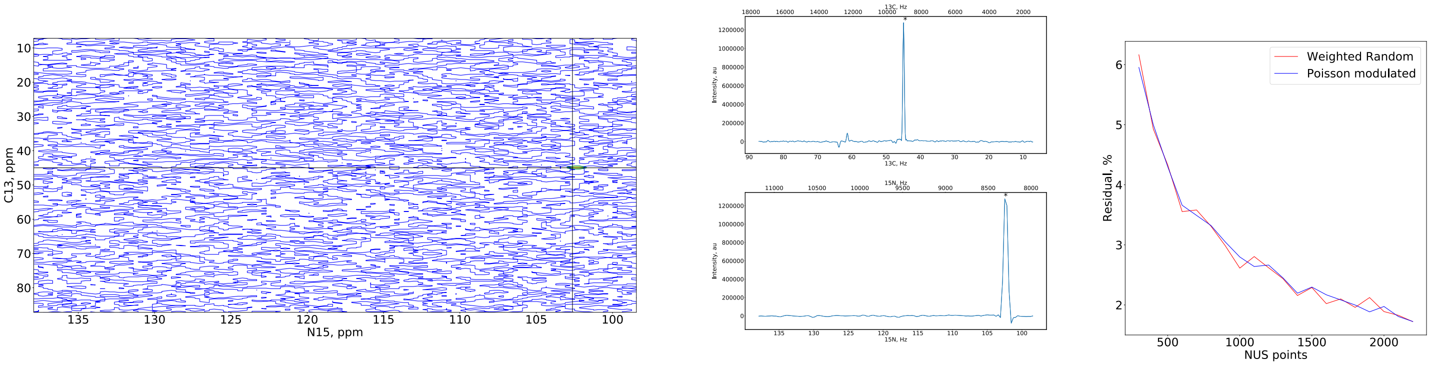

Supplement: Supplementary file 4 — Supplementary file4 (PDF 29610 kb) [file 10858_2021_385_MOESM4_ESM.pdf]
